# Mutational landscape of MCPyV-positive and MCPyV-negative Merkel cell carcinomas with implications for immunotherapy

**DOI:** 10.18632/oncotarget.6494

**Published:** 2015-12-07

**Authors:** Gerald Goh, Trent Walradt, Vladimir Markarov, Astrid Blom, Nadeem Riaz, Ryan Doumani, Krista Stafstrom, Ata Moshiri, Lola Yelistratova, Jonathan Levinsohn, Timothy A. Chan, Paul Nghiem, Richard P. Lifton, Jaehyuk Choi

**Affiliations:** ^1^ Department of Genetics, Yale School of Medicine, New Haven, CT, USA; ^2^ Howard Hughes Medical Institute, Yale School of Medicine, New Haven, CT, USA; ^3^ Department of Dermatology, Yale School of Medicine, New Haven, CT, USA; ^4^ Human Oncology and Pathogenesis Program, Memorial Sloan Kettering Cancer Center, New York, NY, USA; ^5^ Department of Dermatology, University of Washington, Seattle, WA, USA; ^6^ Department of Radiation Oncology, Memorial Sloan Kettering Cancer Center, New York, NY, USA; ^7^ Department of Pathology, University of Washington, Seattle, WA, USA; ^8^ Fred Hutchinson Cancer Center, Seattle, WA, USA; ^9^ Department of Dermatology, Veterans Affairs Healthcare, West Haven, CT, USA; ^10^ Current address: Department of Dermatology, Feinberg School of Medicine, Northwestern University, Chicago, IL, USA

**Keywords:** Merkel cell carcinoma, merkel cell polyomavirus, TP53, cancer genetics, tumor neoantigens

## Abstract

Merkel cell carcinoma (MCC) is a rare but highly aggressive cutaneous neuroendocrine carcinoma, associated with the Merkel cell polyomavirus (MCPyV) in 80% of cases. To define the genetic basis of MCCs, we performed exome sequencing of 49 MCCs. We show that MCPyV-negative MCCs have a high mutation burden (median of 1121 somatic single nucleotide variants (SSNVs) per-exome with frequent mutations in *RB1* and *TP53* and additional damaging mutations in genes in the chromatin modification (*ASXL1, MLL2*, and *MLL3*), JNK (*MAP3K1* and *TRAF7*), and DNA-damage pathways (*ATM, MSH2*, and *BRCA1*). In contrast, MCPyV-positive MCCs harbor few SSNVs (median of 12.5 SSNVs/tumor) with none in the genes listed above. In both subgroups, there are rare cancer-promoting mutations predicted to activate the PI3K pathway (*HRAS, KRAS, PIK3CA, PTEN*, and *TSC1*) and to inactivate the Notch pathway (*Notch1* and *Notch2*). *TP53* mutations appear to be clinically relevant in virus-negative MCCs as 37% of these tumors harbor potentially targetable gain-of-function mutations in *TP53* at p.R248 and p.P278. Moreover, *TP53* mutational status predicts death in early stage MCC (5-year survival in *TP53* mutant vs wild-type stage I and II MCCs is 20% vs. 92%, respectively; *P* = 0.0036). Lastly, we identified the tumor neoantigens in MCPyV-negative and MCPyV-positive MCCs. We found that virus-negative MCCs harbor more tumor neoantigens than melanomas or non-small cell lung cancers (median of 173, 65, and 111 neoantigens/sample, respectively), two cancers for which immune checkpoint blockade can produce durable clinical responses. Collectively, these data support the use of immunotherapies for virus-negative MCCs.

## INTRODUCTION

Merkel cell carcinoma (MCC) is an aggressive neuroendocrine carcinoma of the skin most commonly found on the sun-exposed skin of older Caucasian adults. Roughly one-third of patients with MCCs die of the disease, making MCCs the most lethal skin cancer on a case-by-case basis [1]. The Merkel cell polyomavirus (MCPyV) is clonally integrated in roughly 80% of MCCs (MCPyV-positive MCCs) [2]. MCPyV encodes two viral oncogenes, the small and large T antigens, which are critical for tumorigenesis. The identity of cancer promoting mutations in either MCPyV-negative or MCPyV-positive MCCs is still not completely clear.

In part because of its relative rarity, information is limited regarding the genetic basis of MCC. Genome-wide studies have had limited numbers of samples (*n* = 15 or fewer) [3]. Small cohorts prohibit the use of statistics to distinguish between cancer drivers and passenger mutations and are susceptible to both false positive and false negative findings [4]. Previous reports show that *RB1* is inactivated by large T antigen in MCPyV-positive MCCs and by inactivating mutations in MCPyV-negative MCCs [3]. In addition, there have been reports of rare, activating mutations in *PIK3CA* and *AKT1* in a small fraction of MCCs [5]. However, the incidence of disease promoting mutations in other genes such as *TP53* remains unclear [3, 6].

Strikingly, the incidence of MCCs is dramatically elevated in immunosuppressed patients [7]. These data suggested that MCCs are routinely subject to tumor immunosurveillance and led to the discovery of the cancer-promoting merkel cell polyomavirus (MCPyV). In virus-positive MCCs, the presumptive tumor antigens are non-self proteins encoded in the viral genome [8]. Although several studies have suggested that lymphocyte infiltration can occur and is highly protective in virus negative MCCs [9, 10], the basis for immune recognition of virus-negative MCCs remains unclear.

Herein we report the genomic landscape of MCCs from the study of 49 cases with the identification of putative cancer driver gene mutations and tumor antigens in both MCPyV-negative and MCPyV-positive MCCs.

## RESULTS AND DISCUSSION

To determine the genetic basis of Merkel cell carcinoma, we performed whole exome sequencing on 49 MCCs and matched normal peripheral blood mononuclear cells (Supplementary Table S1; Methods). Of note, some viral status data were available at the time of selecting cases for this exome sequencing study. These data were used to enrich the fraction of virus-negative MCCs (otherwise expected to be only about 20% of cases) in order to have good representation of these tumors and improve the ability to compare these two distinct subtypes. However, in the absence of a single, gold standard test for viral status, the exact number of MCPyV-positive and MCPyV-negative MCCs used in our study were not apparent at the start of the sequencing effort.

The tumors and matched normal cells were sequenced to a median coverage depth of 203 and 103 independent reads per targeted base, respectively (Supplementary Table S2). Somatic single nucleotide variants (SSNVs) and somatic copy number variants (SCNVs) were identified by comparing the read distributions between matched tumor and normal samples (Supplementary Table S3; Methods). Somatic mutations were only called if they were absent from the normal controls.

We examined our cohort for driver genes using the following analyses (see Materials and Methods).

**Figure 1:**
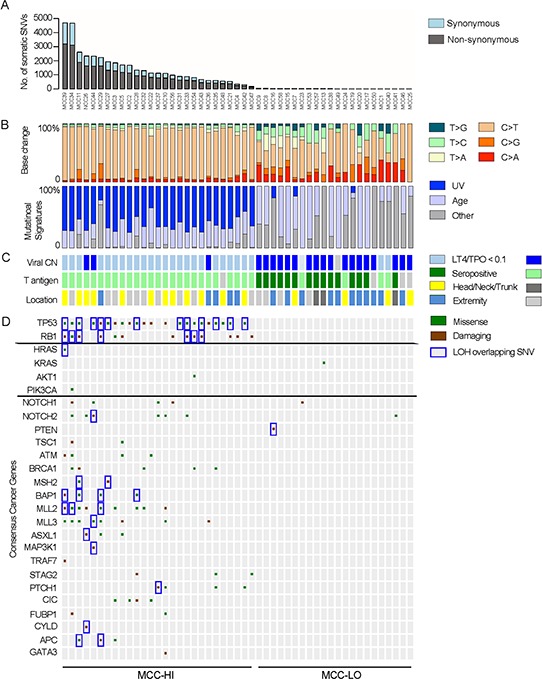
Landscape of somatic alterations in MCC **A.** Number of non-synonymous and synonymous somatic single nucleotide variants (SSNVs) per sample. **B.** Relative frequency of the SSNVs with the relative frequency of an ultraviolet light or age-induced mutational signature. **C.** Clinical parameters associated with each tumor that relate to viral status. For viral copy number (CN), light blue reflects LT4-TPO DNA-PCR ratios < 0.1. Dark blue reflects ratios > 0.1. For T antigen antibody serology, dark green indicates antibody titers< 1:150 (seropositive) and light green indicates antibody titers > 1:75 (seronegative). For viral CN and for T antigen serologies, light gray boxes indicate test not done for the sample. For location, light gray boxes indicate other location or primary site not known. **D.** Select significant somatic mutations identified by exome sequencing are shown. Genes were identified by significant mutation burden (TP53), significant burden of damaging mutations (TP53 and RB1), presence of hotspot mutations in canonical oncogenes (HRAS, KRAS, AKT1, PIK3CA), and presence of damaging mutations in canonical tumor suppressors. Brown square indicates damaging mutations, i.e. nonsense mutations, frameshift mutations, and splice-site mutations. Green indicates missense mutations.

**Figure 2:**
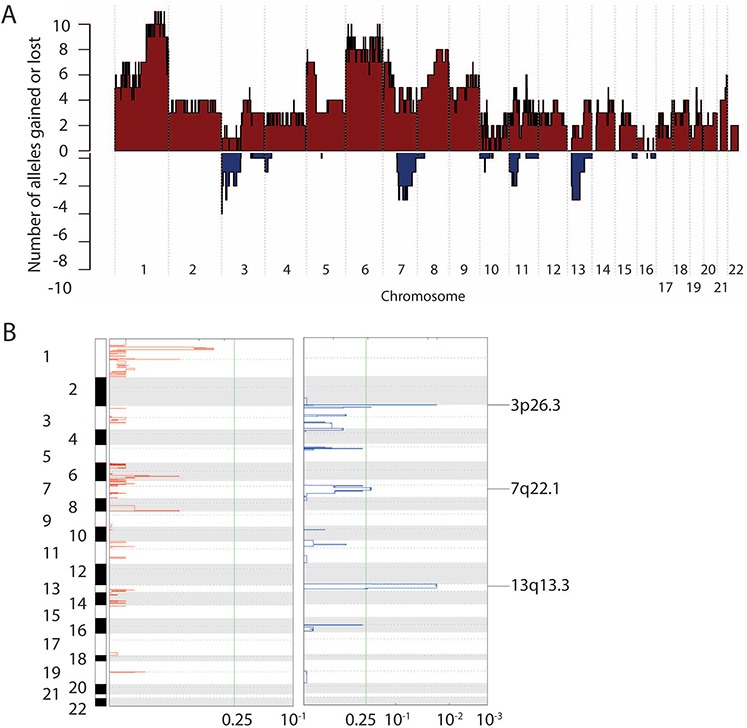
Somatic copy number variants in MCC **A.** Number of alleles deleted or amplified at each genomic position across the MCC cohort. These numbers reflect the product of the number of alleles gained or lost per each sample and the number of samples harboring SCNVs at that position. **B.** Significant focal SCNVs identified by GISTIC. *Q*-value threshold (indicated by green line) = 0.25.

1. We first identified genes that had a higher mutation burden than expected by chance (*Q* < 0.15). This analysis implicated only one gene, *TP53* (34 SSNVs in 22 MCCs; *Q* = 0.001) (Supplementary File S1; Figure 1).
2. We examined the cohort for putative tumor suppressors by looking for genes with a higher burden of loss-of-function mutations than expected by chance alone (*Q* < 0.15) (Supplementary File S1). These loss-of-function mutations included nonsense mutations, splice-site mutations, and frameshift mutations. Only two genes had more damaging SSNVs than expected by chance: *RB1* (13 damaging SSNVs in 11 MCCs; *Q* = 7E-14) and *TP53* (8 damaging SSNVs in 7 MCCs; *Q* = 1.1E-8).
3. We examined the cohort for other candidate tumor suppressors. We looked for damaging mutations in canonical tumor suppressors [11]. This analysis implicated an additional 20 putative tumor suppressors (Supplementary Table S4). These include tumor suppressors in the PI3K pathway (*PTEN, TSC1*), Notch pathway (*Notch1, Notch2*), the JNK pathway (*MAP3K1, TRAF7*), and in chromatin modifying genes (*MLL2, MLL3*, and *ASXL1*) (Supplementary Figure S1).
4. We examined the cohort for recurrent amino acid substitutions in MCCs that occurred more often than expected by chance. These recurrent alterations can suggest gain-of-function oncogenic mutations, such as *BRAF* p.V600E. This analysis implicated only two mutations. Both hotspot mutations occurred in *TP53*. p.R248 and p.P278 were mutated in 6 and 4 MCCs, respectively (*Q* < 0.15). p.R248 and p.P278 are sites of two previously characterized gain-of-function mutations in *TP53* [12].
5. We examined the cohort for functionally validated oncogenic mutations found in other cancers. We found single instances of oncogenic mutations that activate the PI-3-kinase pathway, each mutated in a single MCC sample. These included mutations in *KRAS* (p.G12D), *AKT1* (p.E17K), *HRAS* (p.G12S), and *PIK3CA* (p.M1004I) (Figure 1d).
6. We had high quality SCNV data for 16 of the 49 MCCs. We used GISTIC to identify focal somatic copy number variants (SCNVs) that recurred more often than expected by chance (*Q* < 0.25) (Figure 2a, 2b; Supplementary Table S5). This analysis identified three statistically significant recurrent, focal deletions: 3p26.3 (deleted in 6 of 16 MCCs), 13q12.12 (deleted in 5 of 16 MCCs), and 7q21.2 (deleted in 6 of 16 MCCs). Of these three deletions, only 13q12.12 harbored consensus tumor suppressors (*BRCA2* and *RB1*). There were no statistically significant recurrent amplifications.

### Bimodal distribution of number of SSNVs in MCCs

**Figure 3:**
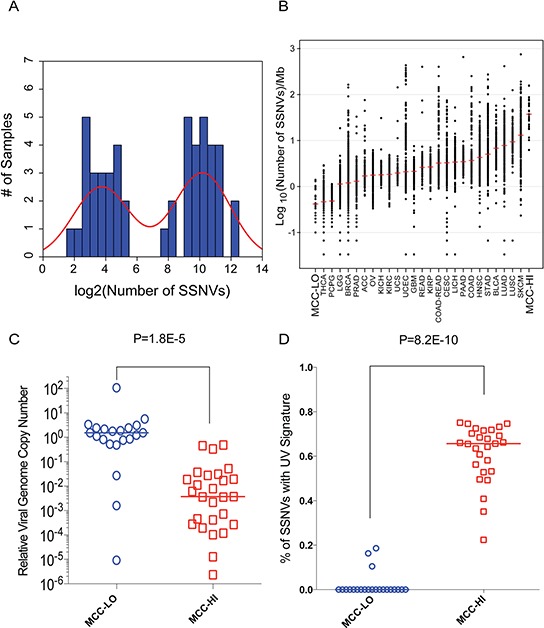
MCC-HI's are MCPyV-negative and harbor ultraviolet light-induced mutations **A.** Histogram of SSNVs among tumors demonstrates a striking bimodal distribution of mutational burden in MCCs. **B.** Relative number of SSNVs in MCCs compared to all solid tumors sequenced by The Cancer Genome Atlas. The red line reflects the median number of SSNVs in each group. Samples are indicated by standard TCGA terminology. **C.** Relative viral load in MCC-HIs and MCC-LOs. Relative number of viral genomes were assessed by qRT-PCR. TPO was used as a control for relative amounts of host genomic DNA. **D.** The proportion of mutations whose genomic context suggest they were caused by ultraviolet light. For panels C and D, the colored lines indicates the median value for each subgroup. Statistical significance was determined with a two-sided unpaired *t*-test.

We found that the number of SSNVs in MCC varied widely (range: 3–4707 per-exome) (Figure 1a, Supplementary Table S3). The distribution of SSNVs was bimodal with each subgroup of MCCs representing extreme cases in cancer biology (Figure 3a). One subgroup (MCC-LO) consisted of 22 MCCs and harbored a median of 12.5 SSNVs per-exome (range: 3–42 SSNVs per-exome). The second subgroup (MCC-HI) consisted of 27 MCCs and harbored a median of 1121 SSNVs per-exome (range: 187–4707 SSNVs per-exome). These median mutation rates for MCC-LO and MCC-HI were lower and higher respectively (Figure 3b) than any epithelial cancer sequenced by The Cancer Genome Atlas [13] (date of inquiry: March 15, 2015).

### MCC-LOs are MCPyV-positive

We hypothesized that the MCC-HIs and MCC-LOs represented MCPyV-negative and MCPyV-positive tumors, respectively. The biochemical functions of MCPyV oncoproteins may make most somatic mutations superfluous and thereby explain the low burden of mutations in MCPyV-positive MCCs. We thus assessed our cohort for viral status. Since there is currently no gold standard for the viral status of MCCs, we performed multiple parallel analyses.

First, we examined 48 of the 49 MCCs for the relative amounts of viral DNA using qRT-PCR. MCC-LOs harbored clonal levels of MCPyV (median viral copy number = 1.5) whereas MCC-HIs harbored very little MCPyV DNA (median viral copy number = 0.0037). This difference was highly significant (*P* = 1.8E-5 two-sided Mann-Whitney test) (Figure 3c).

Secondly, we examined the serum of 45 of the 49 patients in our cohort for antibodies against T antigens because this test is 100% specific (though only 64% sensitive) for MCPyV-positive MCCs [8]. 15 of the 19 MCC-LOs we tested and zero of the 26 MCC-HIs had detectable antibodies to T antigen. The presence of positive serologies exclusively in MCC-LOs was unlikely to occur by chance alone and supports a role for MCPyV in their pathogenesis (*P* = 3.03E-8; two-sided Fisher's exact test) (Figure 1c).

Lastly, we examined the anatomical distribution of MCC-LOs and MCC-HIs. Virus-positive MCCs have been found to occur more frequently on limbs than virus-negative MCCs [14]. Consistent with their viral etiology, MCC-LOs were significantly more likely to occur on the limbs than MCC-HIs (50% of MCC-LOs vs. 22% of MCC-HIs; *P* = 0.03; two-sided Fisher's exact test) (Figure 1c).

There is no single test that can distinguish viral status for MCCs. However, our data support the finding that MCC-LOs represent MCPyV-positive (MCPyV+) and MCC-HIs, MCPyV-negative (MCPyV-) MCCs. These results are consistent with a recently published report, which found a similar distribution of SSNVs in a comparison of a smaller cohort of virus-positive and virus-negative MCCs [3]. While these terms are not synonymous for the reasons detailed above, we will nevertheless forthwith call MCC-LOs MCPyV-positive MCCs and MCC-His MCPyV-negative MCCs.

Similar to MCCs, head and neck squamous cell carcinomas (HNSCCs) are thought to have a viral and non-viral etiologies. Analogous to MCCs, human papillomavirus (HPV) associated HNSCCs harbor two to four-fold fewer SSNVs than non-virally associated HNSCCs [15, 16] (compared to the two-log fold difference we observe in SSNVs in MCPyV-positive and MCPyV-negative MCCs).

### MCPyV-negative MCCs are mutagenized by UV light

We then investigated the mechanisms underlying the high mutation rate in MCPyV-negative MCCs. To do so, we first examined the cohort for the relative frequency of each class of SSNV mutations (Figure 1b). We found that MCC-HIs were significantly enriched for C > T transitions (median of 86% of SSNVs in MCC-HIs vs. 47% of SSNVs in MCC-LOs; *P* = 3.1E-7; two-sided Mann-Whitney test). Because of the presence of MCCs on the skin, we hypothesized that the enrichment of C > T were a result of ultraviolet light (UV); however, we acknowledged that C > T transitions can be caused by other mechanisms, e.g. such as aging and impaired mismatch repair [17].

To distinguish between these possibilities, we took advantage of a recently developed algorithm that extracts mutational signatures from somatic mutations [17].

We found that a median of 66% of SSNVs per MCPyV-negative MCC could be attributed to signature 7, typically thought to be due to UV exposure (Supplementary Figure S2). We hypothesized this number would be comparable for other UV-induced cancers. To show this, we performed a similar analysis on cutaneous melanoma [18] and found that 69% of SSNVs in melanoma can be explained by the UV signature. These values in virus-negative tumors were significantly higher than for MCPyV-positive MCCs, where the UV signature was not present at all (median of 0% of SSNVs per MCPyV-positive MCC, *P* = 8.2E-10; two-sided Mann-Whitney test) (Figure 3d).

These data suggest a primary role for UV light in the mutagenesis of MCPyV-negative MCCs but not in MCPyV-positive MCCs. It is possible that for MCPyV-positive MCCs, UV light promotes cancer not through its mutagenic effects but rather through its effects on the tumor microenvironment [19], e.g. its local immunosuppressive effects. Also, UV may promote the rare mutations required for MCPyV integration into the epidermal cell genome and truncation of the large T antigen that is clonal, required for tumorigenesis, and not compatible with continued propagation of the virus outside the host genome [20, 21].

### Driver genes in each subclass of MCCs

Virus-positive MCCs harbored very few SSNVs or SCNVs in putative cancer genes (mean of 0.18 disease-promoting SSNVs per-exome. There were no SSNVs in *TP53* and no SSNVs or SCNVs in *RB1*. There were also single instances of mutations in three candidate tumor suppressors and also a single instance of an oncogenic mutation in *KRAS* (p.G12D). These mutations are predicted to inactivate Notch (SSNVs in *Notch1* and *Notch2*) and activate the PI-3-kinase pathway (*KRAS* and a nonsense mutation in *PTEN*).

In contrast, MCPyV-negative MCCs harbored a mean of 4.9 SSNVs in putative disease-promoting cancer genes (Supplementary Table S4), the vast majority of which occur in putative tumor suppressors. These include frequent mutations in *TP53* (SSNVs in 76% of MCPyV- MCCs (22 of 29 samples)) and *RB1* (SSNVs in 45% of MCC-HIs (13 of 29 samples)). For the 16 MCCs with both SSNV and SCNV data, *RB1* is subject to deletion or SSNV in 67% of MCCs (6 of 9 MCPyV-negative MCCs; 0 of 7 MCPyV-positive MCCs) (Supplementary Table S6). 33% do not have a detectable SCNV or SSNV in *RB1*.

MCPyV-negative MCC harbors additional SSNVs (mean of 0.9 damaging SSNVs and 2.1 missense SSNVs) in other putative tumor suppressors (Supplementary Table S4, Supplementary Figure S1, Figure 1). These mutations are predicted to impair DNA damage repair pathways, impair JNK-mediated apoptosis, and alter chromatin, These pathways are not subject to somatic mutations in MCPyV-positive MCCs. These data suggest the importance of these pathways to MCPyV-negative MCC tumorigenesis *in vivo*. We did not find any damaging mutations in *PRUNE2*, which were reported in a previous cohort of 8 MCPyV-negative MCCs [3].

We had originally hypothesized that somatic mutations in MCPyV-negative MCCs may phenocopy the effects of viral oncoproteins, such as small T and large T antigens. For example, in MCPyV-negative MCCs, RB1 is inactivated by large T antigen whereas in MCPyV-negative MCCs, RB1 is inactivated by somatic mutations. Recent research has shown that MCPyV small and large T antigens inhibit apoptosis via upregulation of survivin [22], inhibit proteasomal degradation via inhibition of ubiquitin ligases [23], and augments cap-dependent translation of mRNA [24]. Surprisingly, we did not see mutations that occurred more often than expected by chance in these oncoprotein targets in MCPyV-negative MCCs. Alternatively, our data may suggest, however, an as yet undiscovered role for MCPyV oncoproteins in DNA damage repair, JNK signaing, or chromatin modification.

Furthermore, PP2A isoforms have been identified as a target of small T antigen in SV40 polyomavirus-induced cancers [25]. We therefore examined our MCC cohort for mutations in these genes (Supplementary Table S7). We found that there were fewer mutations in these genes than expected by chance alone. No individual PP2A isoform harbors a protein-altering mutation in more than 1 MCC, and the *Q*-value for statistical significance for each isoform is 1. Collectively, we had expected 20 non-synonymous mutations in PP2A isoforms and found only 12 non-synonymous SSNVs in our cohort. These data are consistent with previous reports that suggest that PP2A complex does not play a significant role in MCC tumorigenesis [24].

The Notch pathways and PI-3-K pathways are mutated in both virus-positive and virus-negative MCCs. As with MCPyV-negative MCCs, we found sporadic SSNVs affecting *Notch1* and *Notch2* as well as single instances of gain of function mutations in *AKT1* (p.E17K), *HRAS* (p.G12S), and *PIK3CA* (p.M1004I), which are all predicted to activate the PI-3-K pathway.

Small cell lung cancer (SCLC) has a similar spectrum of mutations with near universal mutations in *TP53* and *RB1*, sporadic inactivating mutations in the Notch pathway, and rare activating mutations in the PI-3-kinase pathways [26]. These data suggest that dysregulation of these genes and pathways are obligatory for the neuroendocrine differentiation of epithelial cells, a common feature of both MCCs and SCLCs.

### Role of TP53 in MCPyV-negative MCC

**Figure 4:**
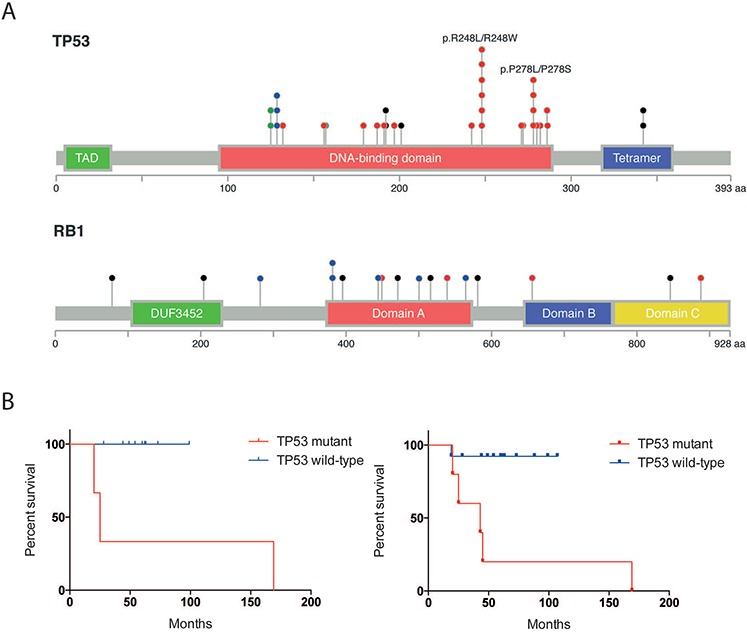
Statistically significant mutations in MCPyV-negative MCC **A.** Schematic of SSNVs in *RB1* and *TP53*. The domains were defined by Uniprot. Missense mutations are shown in Red. Damaging mutations are shown in black. **B.** Kaplan-Meier plot of overall survival as a function of *TP53* mutational status in patients with Stage I MCC (left) or a combined cohort of Stage I and II MCCs (right). *P*-values were assessed by log-rank (Mantel-Cox) test.

The mutations in *RB1* are typical of tumor suppressor mutations. There are highly prevalent loss-of-function mutations widely distributed across the gene (Figure 4a). In contrast, in *TP53*, there are frequent, recurrent amino acid substitutions at two amino acids, p.R248 (*n* = 6) and p.P278 (*n* = 4), which occur more frequently than predicted by chance alone (*P* = 1.2E-8; binomial distribution). There are two amino acid substitutions at each position (p.R248W/L, p.P278L/S). These amino acid substitutions may or may not have similar functional consequences.

Accordingly, both of these recurrent amino acid substitutions have been functionally validated by multiple groups to be oncogenic, gain-of-function (GOF) mutations (not loss-of-function mutations) that increase tumor aggressiveness in other cancer types [12]. Although the molecular mechanisms are not entirely clear, these mutations (p.R248W and p.P278S) have been shown in lung, pancreatic, colorectal, prostate, and osteosarcoma cell lines to increase tumor initiation, cell proliferation, tumor metastasis, and drug resistance [12].

Recent data suggest that mutant GOF P53 may be targetable. Stable expression of mutant GOF P53 requires HDAC6, which catalyzes the interaction of mutant P53 with the Hsp70/Hsp90 chaperone complex [27]. HDAC6 inhibitors and HSP90 inhibitors target mutant p53 for proteasomal degradation and have been shown to be effective in multiple cancer types including cutaneous squamous cell carcinomas [28], colorectal adenocarcinoma [28, 29], pancreatic adenocarcinoma [29], breast adenocarcinoma [29], and T cell lymphomas [27]. These agents may therefore also be a useful therapeutic strategy for p.R248W MCCs. The pan-HDAC inhibitor, SAHA, is FDA-approved, and HSP90 inhibitors [30] are currently in clinical trials.

*TP53* mutational status has been reported to portend a poor prognosis in several early stage cancers, such as node-negative breast cancer [31] and stage I non-small cell lung cancer [32]. To test the prognostic value of *TP53* mutations in early stage MCC, we performed a Kaplan-Meier analysis on the localized MCCs in our cohort (13 stage I cases and 5 Stage II cases). These included 5 *TP53* mutant MCPyV-negative MCCs and 13 *TP53* wild-type MCCs (2 MCPyV-negative MCCs and 11 MCPyV-positive MCCs) (Supplementary Table S8). *TP53* mutations in stage I MCCs were associated with a significantly poorer prognosis (*P* = 0.0044; log-rank Mantel-Cox test). There were no deaths in patients who had *TP53* wild-type stage I MCCs (median follow-up = 57 months). In contrast, all patients with stage I *TP53* mutant MCCs died of disease (median survival= 34 months). Similar patterns were seen when we compared the combined cohort of stage I and II MCCs (*P* = 0.032; log-rank Mantel-Cox test) (Figure 4b, Supplementary Table S8). Patient age was also predictive of overall survival in early stage MCCs. There was no significant association between prognosis, anatomic site, or viral status (Supplementary Table S9).

### MCPyV-negative MCCs harbor a high number of neoantigens

In our dataset, 18.5% (5 of the 27) virus-negative MCC-HIs occurred in patients who are considered chronically immunosuppressed. This high frequency of immune suppression suggest that these cancers, like the MCPyV-positive MCCs, are immunogenic and are similarly subject to tumor immunosurveillance. Because they do not express viral proteins, we hypothesized that their immunogenicity is a result of somatic mutations, which generate “non-self” peptides that can serve as tumor neoantigens. Across multiple cancer types, the number of somatic point mutations track with the number of neoantigens and predict responses to immunotherapies [33, 34]. The mutational burden in MCPyV-negative MCCs (median number of SSNVs = 1121) is roughly five-fold higher than that observed for non-small cell lung cancer (median number of SSNVs = 199), and melanoma (median number of SSNVs = 248), both of which are considered relatively responsive to immune checkpoint blockade [33, 34].

**Figure 5:**
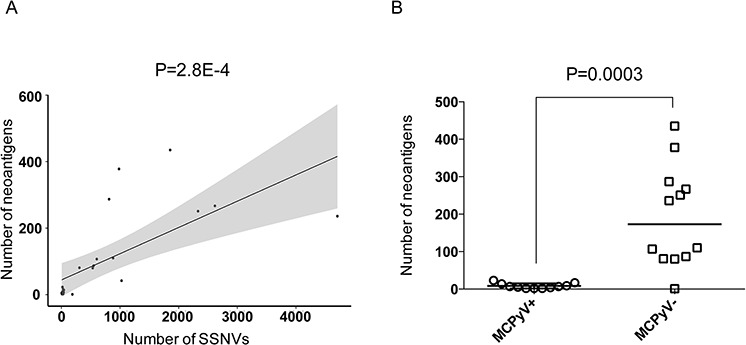
MCPyV- MCCs have a high burden of predicted neoantigens **A.** Plot of predicted neoantigens as a function of mutational burden. Mutant peptides that bind tightly to tumor cell's MHC class I molecules (Ka ≤ 500 nM) were identified for each tumor. These predicted neoantigens were plotted against the number of total somatic SNVs. Pearson linear regression analysis was performed. The gray shaded areas represent the 95% confidence interval. **B.** Plot of predicted neoantigens in MCPyV-negative (MCPyV-) MCCs vs. MCPyV-positive (MCPyV+) MCCs. Line indicates the median value for each subgroup. Statistical significance was determined using an unpaired *t*-test.

We hypothesized that because of the high incidence of point mutations, MCPyV-negative MCCs would harbor a significant number of tumor neoantigens. We therefore identified the MHC class I molecules expressed in each MCC from the exome data using a published algorithm [35]. We successfully identified MHC class I haplotypes for 23 MCCs (12 MCPyV-negative MCCs and 11 MCPyV-positive MCCs; median number of MHC class I HLA types identified per MCC= 3.2) (Supplementary Table S10). We then identified the number of mutant peptides that are predicted to bind tightly to the tumor MHC class I molecules (Ka ≤ 500 nM) (Supplementary Table S11). As expected, the number of predicted neoantigens correlated with the number of somatic point mutations (*R* = 0.68; *P* = 2.8E-4; Pearson's test of correlation) (Figure 5a). The median number of predicted neoantigens in MCPyV-negative MCCs was 173, which, as expected, was higher than for MCPyV-positive MCCs (median of 7 neoantigens in the MCPyV-positive MCCs, *P* = 0.0003; two-sided Mann-Whitney test) (Figure 5b). These numbers likely represent an underestimate as in the majority of cases, we failed to identify all 6 MHC class I haplotypes to which mutant peptides could bind (Supplementary Table S10). Strikingly, the burden of predicted neoantigens in MCPyV-negative MCCs was higher than for non-small cell lung cancer [34] (median number of neoantigens = 111) and melanoma [33] (median number of neoantigens = 65), which are two cancers for which immune checkpoint blockade can produce durable clinical responses.

Trials are currently underway to explore the use of immunotherapies for this disease [7]. To date, these immunotherapy studies have actively excluded immunosuppressed patients because of concerns that suboptimal immune systems may limit the effectiveness of these therapies. Their utility in this patient population will need to be determine empirically in future studies.

The rationale for MCPyV-positive MCCs is based on the concept that virus-associated cancers are intrinsically immunogenic because they express foreign, viral antigens that are recognized by the host lymphocytes [8, 36]. Our data supports the use of immunotherapies also for MCPyV-negative MCCs, which in fact have a higher mutation and neoantigen burden than melanoma [33] or non-small cell lung cancer [34]. Future clinical trials will determine if immunotherapies are effective for MCCs and if the number of neoantigens will predict responses to immunotherapy for MCPyV-positive or MCPyV-negative MCCs. The genomic landscape of Merkel cell carcinoma defined herein shape therapeutic opportunities and present challenges for future research.

## MATERIALS AND METHODS

### Sample preparation and sequencing

All studies were approved by the Institutional Review Board of the Fred Hutchinson Cancer Research Center and Yale University. The MCC tumor samples were deidentified formaldehyde fixed paraffin embedded (FFPE) archival specimens. Light microscopic evaluation was performed on a hematoxylin and eosin stained section of each FFPE tumor specimen for assessment of precent tumor nuclei and percent necrosis. Specimens with mixed differentiation, i.e. evidence of squamous and neuroendocrine differentiation, were excluded from the study. 2 mm cores were obtained that harbor > 80% tumor cells. Peripheral blood mononuclear cells were used as normal controls. Genomic DNA was prepared using standard procedures. Exome capture was performed using the 2.1M NimbleGen exome reagent (Roche NImblegen, Madison, WI). 75 base paired end sequencing on Illumina 2000 (Illumina, San Diego) as previously described [37]. Coverage depth is reported in Supplementary Table S2. Sequences were aligned to human genome build 37 with ELAND (Illumina, San Diego).

### Somatic mutation calling and identification of significantly mutated genes

Somatic mutations were called as previously described [37]. In brief, the significance of differences in read distribution between tumor and peripheral blood mononuclear cells was evaluated at all covered positions with the two-sided Fisher's exact test. *P*-value threshold of 1E-4 was used to yield a list of high-confidence somatic calls. Somatic mutations were filtered to remove variants present in public and Yale databases to remove likely miscalled germline variants.

We identified significant mutated genes using a standard pipeline that accounts for differences in background mutation rates at each gene according to gene length, gene expression, and DNA replication time [13]. Specifically, we used the publicly available MutSigCV v1.3 (https://www.broadinstitute.org/cancer/cga/mutsig).

### Identifying mutational signatures

We identified mutational processes underlying tumorigenesis in MCC by analyzing the somatic mutations in each MCC sample for mutational signatures. We utilized an unpublished R package deconstructSigs (Rosenthal et al, manuscript in preparation) to determine the combination of published mutational signatures [17] that most accurately reconstructed the mutational profile of each tumor sample. To compare with melanoma, we downloaded the dataset from the TCGA and ran the same analysis (https://tcga-data.nci.nih.gov; date of inquiry: August 7, 2015)

### Identifying putative tumor suppressors

Tumor suppressors harbor inactivating mutations, such as damaging nonsense mutations, frameshift mutations, and splice site mutations. To identify the genes with recurrent damaging mutations, we first determined the background damaging mutation rate for each gene correcting for gene length. We then used the binomial distribution to identify the genes whose burden of damaging mutations occurred more often than expected by chance alone. The Bonferroni correction was applied to correct for family-wise error rate. We used a *Q*-value cut-off of 0.15. We also sought individual instances of damaging mutations in from a list of canonical tumor suppressors [11, 38].

### Identifying putative oncogenes

We identified statistically significant hotspot mutations. We calculated the background mutation rate after correction for gene expression. We then used the binomial distribution to calculate the codons with a higher recurrent missense mutation rate than expected by chance. The Bonferroni correction was applied to correct for family-wise error rate (*Q*-value). We used a *Q*-value cut-off of 0.15. We also queried mutations that occurred in COSMIC in greater than 10 cases to identify hotspot mutations in other cancer types (http://cancer.sanger.ac.uk/cosmic). We filtered these mutations for mutations with supporting functional data.

To identify hotspot mutations in MCCs, we identified a background mutation rate after correction for transcription-coupled repair. We then identified the likelihood that more than one mutation would occur at the same codon using the binomial distribution. To assess the statistical significance of recurrent TP53 alterations at UV hotspots, we identified the number of putative UV hotspots in the gene (226 CC's in total on the template or non-template strand in *TP53*) and assessed the likelihood that 10 mutations would segregate at two positions.

### Somatic copy number variant calling and Identification of recurrent SCNVs

Somatic copy number variants from exome data were called using AdTex [39]. We filtered calls in areas of genomic segmental duplication and calls less than 1 Mb in length. SCNV calls from exome data can be variable. Therefore, to test the quality of the SCNV calls, we examined the areas that AdTex called as heterozygous deletions for the expected loss of heterozygosity (LOH) as previously described [37]. For the LOH analysis, we identified heterozygous SNPs in the normal controls (minor allele frequency = 0.4–0.6). We assessed the change in minor allele frequency in tumors with deletions spanning these SNPs. We found that for 16 of the 49 MCCs, 100% of the heterozygous deletions experienced loss-of-heterozygosity and were therefore suitable for downstream analysis. Collectively, because of tumor heterogeneity, we anticipated an expected ΔBAF of 0.304 in heterozygous deletions in these 16 samples. We observed a ΔBAF of 0.309 across the deletions in the 16 samples. To identify statistically significant, recurrent focal SCNVs, we used GISTIC2.0 [40] with the following settings: GeneGISTIC; 75% confidence interval; focal SCNV < 99% of length of a chromosomal arm.

### Quantification of viral copy number

Three to four 4 μm tissue curls were obtained from formaldehyde fixed paraffin embedded MCC tissue blocks. DNA was extracted from these tissue curls using Qiagen QIAamp DNA FFPE tissue kit (Qiagen). qRT PCR was performed on the tissue blocks with TaqMan Probes (Applied Biotechnologies). LT4 primers were used to amplify MCPyV viral genomes. Primers that target thyroid peroxidase (*TPO*) were used as a control because *TPO* is diploid in every Merkel cell carcinoma studied to date [41].

The LT4 primers are the following:

LT-4 F TTCCTCTGGGTATGGGTCCTT
LT-4 R GGTCCTCTGGACTGGGAGTCT
LT-4 Probe TCAGCGTCCCAGGCT

The TPO primers are the following:

TPO F TCCAGCTCAGCTTCTGTCTTTTT
TPO R TCTGCTGCTCGGGCAATC
TPO P CAAACTTCCTGAGCCAACAAGCGGAG

### T antigen antibody serologies

Serologies to the MCPyV T antigens were performed as previously described [8]. The titer of antibodies < 1:75 were considered negative.

### Neoantigen pipeline

All nonsynonymous point mutations identified were translated into strings of 17 amino acids with the mutant amino acid situated centrally using a bioinformatic tool called NAseek [33]. A sliding window method was used to identify the 9 amino acid substrings within the mutant 17mer that had a predicted MHC Class I binding affinity of ≤500nM to one (or more) of the patient-specific HLA alleles. Binding affinity for each nonamer were analyzed using stand-alone software package of NetMHCv3.4 software (http://www.cbs.dtu.dk/services/NetMHC/).

## SUPPLEMENTARY FIGURES AND TABLES






