# Supplementary material for: Mutational landscape of MCPyV-positive and MCPyV-negative Merkel cell carcinomas with implications for immunotherapy
Source: Oncotarget. 2015 Dec 7;7(3):3403–15. doi: 10.18632/oncotarget.6494 (PMC4823115; doi:10.18632/oncotarget.6494)
Supplement: Supplementary file 1 [file oncotarget-07-3403-s001.pdf]

SUPPLEMENTARY FIGURES AND TABLES

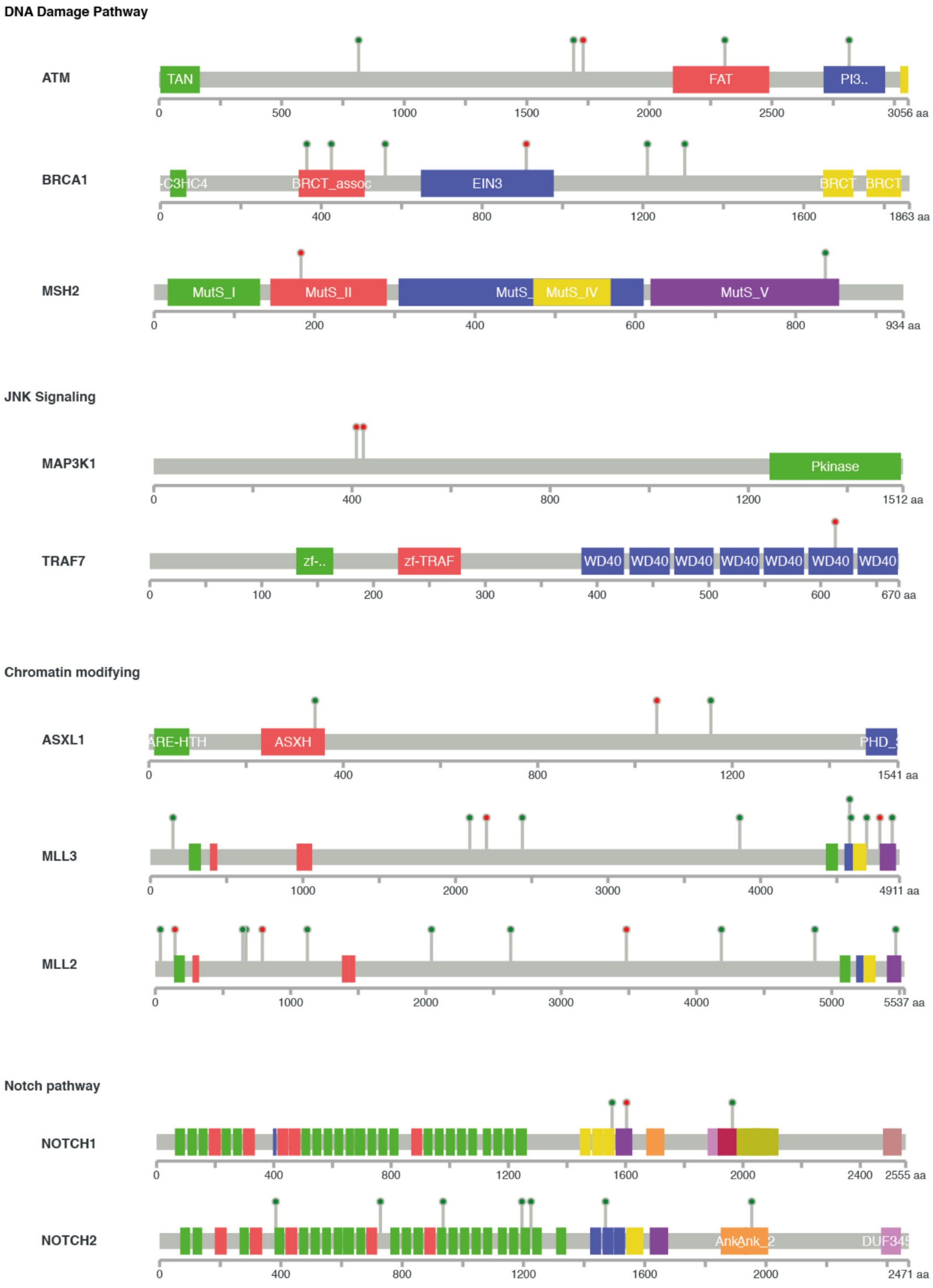

(*Continued*)

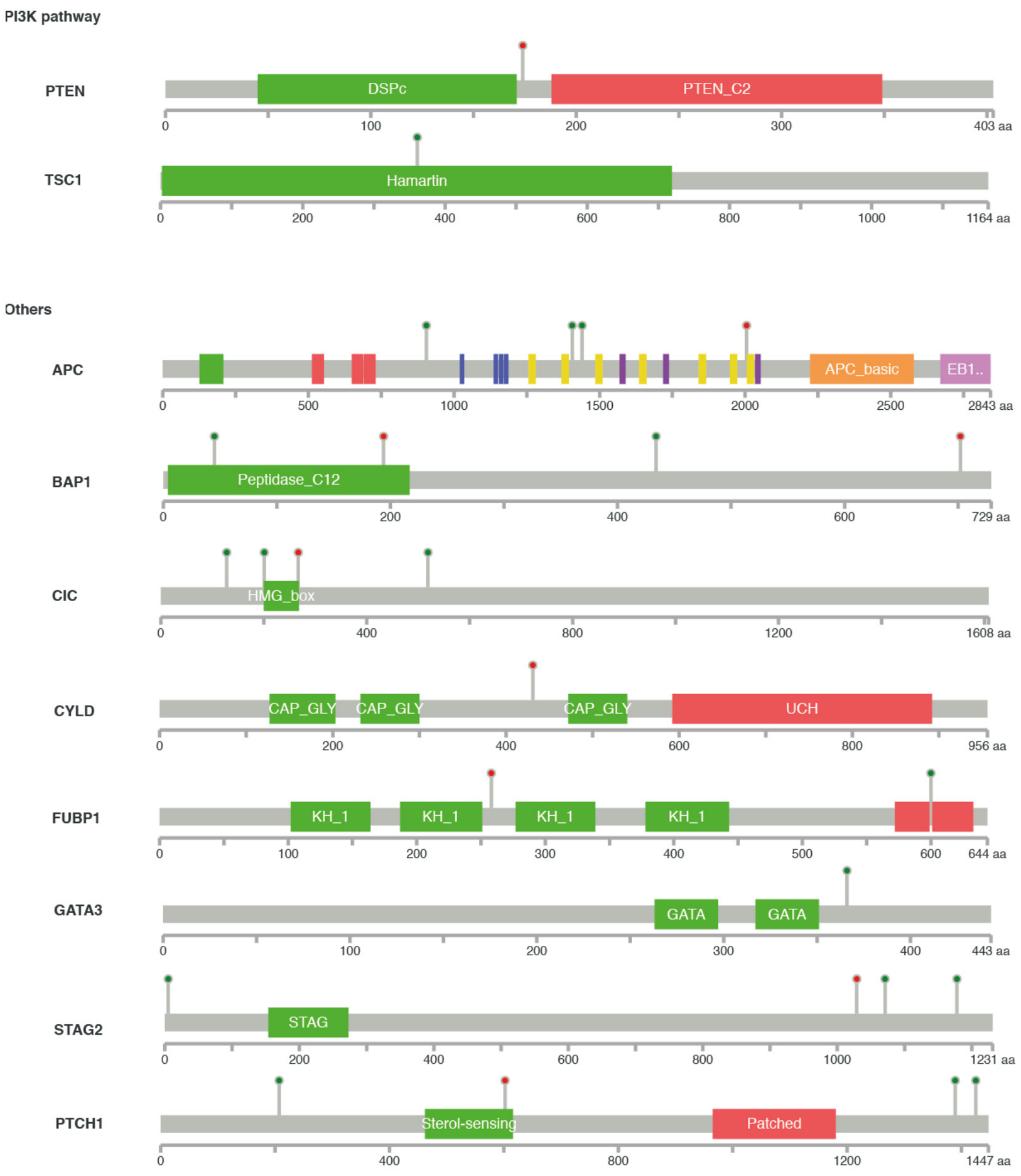

Supplementary Figure S1: Schematic of SSNVs in MCC in putative tumor suppressors. Genes listed are canonical tumor suppressors for which at least one MCC in our cohort harbors a one damaging mutation. All SSNVs in our cohort are detailed. Missense mutations are shown in green, damaging mutations, i.e. frameshift mutations, nonsense mutations, splice site mutations, are shown in red.

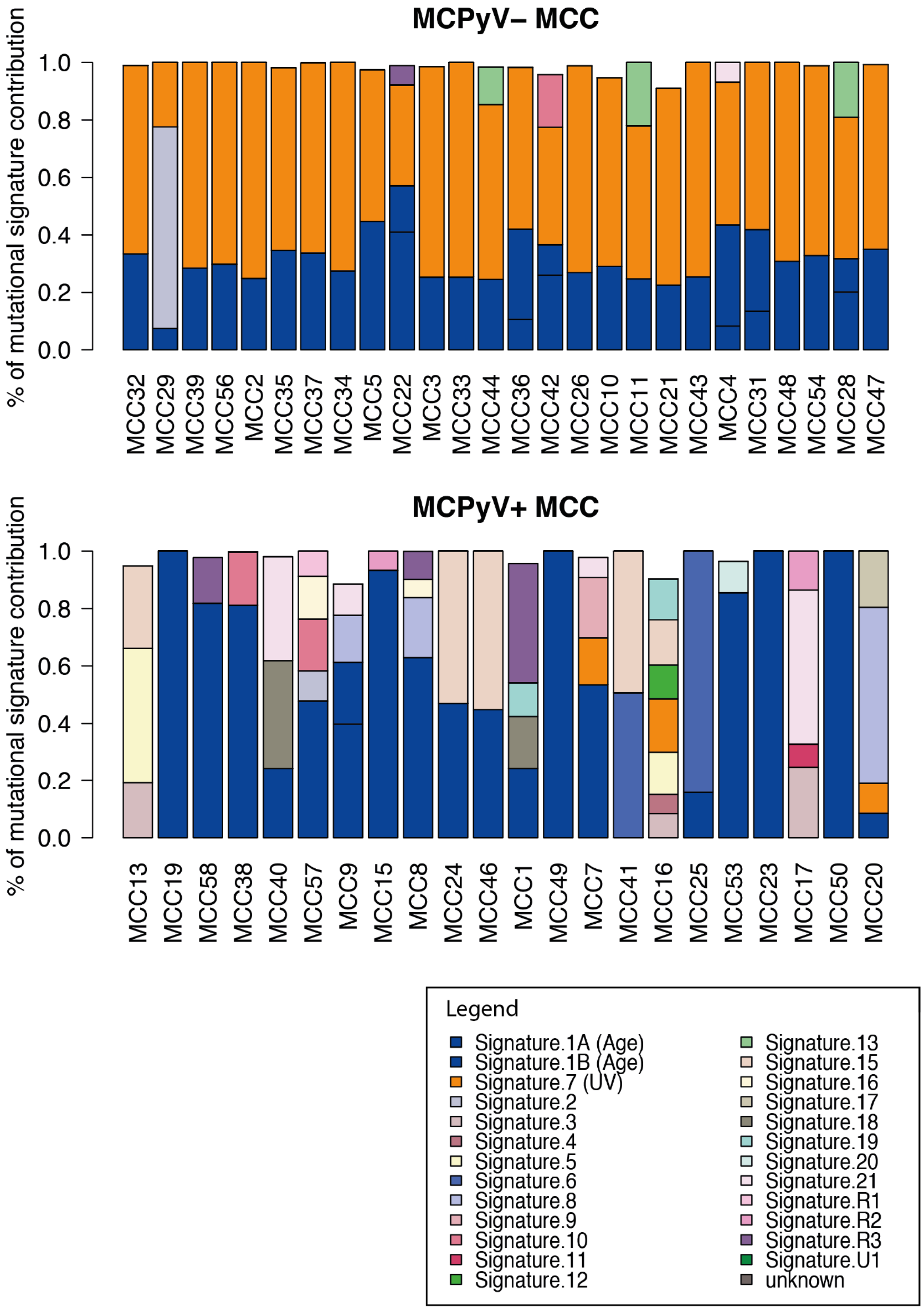

Supplementary Figure S2: Mutational signatures identified in MCCs. A mutational signature was assigned to each SSNV in the MCC cohort as described in the Methods. The relative frequency of SSNVs attributed to each mutational signature as shown in the y-axis; colors of the mutational signatures as detailed in the legend.

Supplementary Table S1: Clinical characteristics of MCCs in our cohort

Serology status reflects the antibody titers to MCPyV T antigen. Unknown reflects one patient whose antibodies were tested after her MCC was excised.

See Supplementary File 1.

Supplementary Table S2: Coverage depth per targeted base

Sample	Normal	Tumor
MCC1	111.665	298.441
MCC2	160.614	174.968
MCC3	116.092	197.255
MCC4	105.27	214.258
MCC5	115.149	207.397
MCC7	190.921	220.601
MCC8	113.499	173.461
MCC9	177.615	215.424
MCC10	110.04	179.794
MCC11	114.046	265.447
MCC13	96.951	157.442
MCC15	108.191	261.418
MCC16	98.182	225.765
MCC17	113.885	208.793
MCC19	110.544	227.47
MCC20	116.737	196.116
MCC21	116.37	188.232
MCC22	108.865	113.248
MCC23	103.417	186.858
MCC24	108.835	175.468
MCC25	82.432	189.248
MCC26	99.604	211.915
MCC27	85.755	145.174
MCC28	98.001	167.171
MCC29	80.321	194.614
MCC31	113.877	157.351
MCC32	99.112	174.702
MCC33	81.918	71.16
MCC34	118.979	159.037
MCC35	119.55	174.13
MCC36	98.29	203.3

(Continued)

Sample	Normal	Tumor
MCC37	84.897	184.17
MCC38	74.957	238.446
MCC39	86.122	274.531
MCC40	87.818	242.956
MCC41	99.968	198.987
MCC42	85.767	233.454
MCC43	68.51	244.711
MCC44	88.969	216.639
MCC46	88.525	240.134
MCC47	92.933	254.609
MCC48	80.514	265.326
MCC49	90.804	227.77
MCC50	89.944	192.161
MCC53	106.433	406.053
MCC54	96.441	211.597
MCC56	200.958	189.086
MCC57	173.251	184.232
MCC58	148.647	207.716

Supplementary Table S3: Table of number of SSNVs by tumor

Sample	Total # of SSNVs	# Synonymous SSNVs	# Non-synonymous SSNVs
MCC39	4707	1506	3201
MCC34	4677	1556	3121
MCC11	2618	731	1887
MCC26	2343	712	1631
MCC44	2330	713	1617
MCC29	2244	606	1638
MCC27	1945	618	1327
MCC3	1855	553	1302
MCC5	1700	527	1173
MCC2	1689	553	1136
MCC28	1340	395	945
MCC32	1308	383	925
MCC22	1126	303	823
MCC37	1121	337	784
MCC10	1104	326	778
MCC56	982	267	715
MCC31	892	269	623
MCC33	881	266	615
MCC54	813	220	593
MCC43	669	215	454
MCC36	604	190	414
MCC35	603	185	418
MCC48	549	167	382
MCC21	534	162	372
MCC4	348	101	247
MCC42	307	88	219
MCC47	187	56	131
MCC9	42	7	35
MCC8	39	7	32
MCC16	31	9	22
MCC58	29	11	18
MCC15	27	4	23
MCC7	26	3	23
MCC23	19	4	15
MCC53	18	5	13
MCC57	18	9	9

(Continued)

Sample	Total # of SSNVs	# Synonymous SSNVs	# Non-synonymous SSNVs
MCC13	16	5	11
MCC38	11	2	9
MCC49	13	2	11
MCC24	12	4	8
MCC20	10	2	8
MCC19	10	6	4
MCC17	7	0	7
MCC50	8	2	6
MCC1	8	4	4
MCC40	6	0	6
MCC41	6	0	6
MCC46	5	1	4
MCC25	3	1	2

Supplementary Table S4: Table of canonical tumor suppressors with at least one damaging mutation in our cohort

Gene	Nonsense	Splice-Site	Frameshift	Missense
NOTCH1	1	1	1	3
MAP3K1	2	0	0	0
PTEN	1	0	0	0
GATA3	1	0	0	0
MLL2	2	1	0	9
FUBP1	1	0	0	1
TRAF7	1	0	0	0
BAP1	1	0	0	3
CYLD	1	0	0	0
MSH2	1	0	0	1
TSC1	0	1	0	1
MLL3	1	1	0	9
STAG2	1	0	0	3
PTCH1	1	0	0	3
ASXL1	1	0	0	2
CIC	1	0	0	3
BRCA1	1	0	0	5
NOTCH2	0	1	0	7
APC	1	0	0	3
ATM	1	0	0	4

Only three mutations occurred in MCPyV+ MCC-LOs: 1 frameshift mutation in *NOTCH1*, 1 nonsense mutation in *PTEN*, and 1 missense mutation in *NOTCH2*.

Supplementary Table S5: Table of significant focal deletions in MCCs

Cytoband	Wide Peak Boundaries	Minimal Common Region	# of Genes	Residual Q Value	# Deletions		Candidate Genes
					MCC-HI	MCC-LO	
3p26.3	chr3:1-3112016	chr3:1-2787415	3	0.019	6	0	-
13q12.12	chr13:1-79894703	chr13:23777842-58207445	285	0.019	5	0	*RB1, BRCA2*
7q21.2	chr7:70255774-134576311	-	495	0.23	2	4	*POT1, ELN*

n = 17 MCCs. 9 MCPyV-negative MCC-HIs, 8 MCPyV-positive MCC-LOs. For 7q21.2, there are more than 1 minimal common regions. Candidate genes represent tumor suppressors from the COSMIC gene census residing on these respective intervals.

Supplementary Table S6: *RB1* mutational status in 9 MCPyV- MCCs with both SCNV and SSNV data

Sample	Viral Status	*RB1* SCNV	*RB1* SSNV
MCC10	MCPyV-	Deletion	
MCC2	MCPyV-	Deletion	
MCC21	MCPyV-		
MCC3	MCPyV-	Deletion	p.S888I
MCC31	MCPyV-		
MCC37	MCPyV-	Deletion	
MCC47	MCPyV-		SS*
MCC48	MCPyV-		
MCC5	MCPyV-	Deletion	

SS* reflects splice-site mutation.

Supplementary Table S7: Mutations in PP2A genes in MCCs

Sample	Chr	Position	Reference	Variant	Gene	Amino Acid Change
MCC29	8	30643801	G	A	*PPP2CB*	p.R294C
MCC22	19	52719792	CC	TT	*PPP2R1A*	p.S335F
MCC48	5	146030151	CC	TT	*PPP2R2B*	p.G195K
MCC39	4	6325311	C	T	*PPP2R2C*	p.M354I
MCC26	10	133761197	C	A	*PPP2R2D*	p.P100H
MCC22	3	135806736	A	T	*PPP2R3A*	p.R934W
MCC29	3	135721362	C	G	*PPP2R3A*	p.S341*
MCC39	3	135801134	G	A	*PPP2R3A*	p.D887N
MCC5	3	135721218	C	T	*PPP2R3A*	p.P293L
MCC44	14	35585844	T	A	*PPP2R3C*	p.Y53F
MCC29	1	212530613	G	C	*PPP2R5A*	p.E405Q
MCC44	14	102323040	C	T	*PPP2R5C*	p.Q38*
MCC26	6	42976923	C	T	*PPP2R5D*	p.L403L
MCC26	8	26227881	C	T	*PPP2R2A*	p.A432A
MCC11	5	146077582	G	A	*PPP2R2B*	p.L98L
MCC5	3	135789353	C	T	*PPP2R3A*	p.L835L

Supplementary Table S8: Survival data on Stage I and II MCCs in our cohort

Patient	# of SSNVs	Stage	Follow-up (months)	Survival	TP53 mutations
MCC11	2620	Stage IIA	43	Deceased	p.R156P
MCC22	1130	Stage IA	20	Deceased	p.R342X, SS*
MCC10	1104	Stage IA	25	Deceased	p.G187D, p.L201X
MCC48	550	Stage IB	169	Deceased	p.E286K, p.R280K
MCC42	307	Stage IIA	45	Deceased	p.K132N
MCC26	2343	Stage IA	60	Alive	wild-type
MCC33	882	Stage IB	54	Alive	wild-type
MCC16	31	Stage IB	73	Alive	wild-type
MCC15	27	Stage IIB	107	Alive	wild-type
MCC7	26	Stage IB	44	Alive	wild-type
MCC57	18	Stage IB	63	Alive	wild-type
MCC13	16	Stage IA	28	Alive	wild-type
MCC38	14	Stage IA	99	Alive	wild-type
MCC24	12	Stage IA	44	Alive	wild-type
MCC19	10	Stage IIA	49	Alive	wild-type
MCC20	10	Stage IA	62	Alive	wild-type
MCC17	8	Stage IIB	19	Deceased	wild-type
MCC41	6	Stage IA	88	Alive	wild-type

SS* Splice Site Mutations

Supplementary Table S9: Further analysis on survival data on MCCs in our cohort

<table>
<tr><th>Stage</th><th>Group</th><th>Number of patients</th><th>Median Survival (days)</th><th>Univariate p value</th><th>Multivariate p value</th></tr>
<tr><td rowspan="10">All stages</td><td>MCC-LO</td><td>22</td><td>NA</td><td rowspan="2">0.627</td><td rowspan="2">0.823</td></tr>
<tr><td>MCC-HI</td><td>27</td><td>1680</td></tr>
<tr><td>Unknown</td><td>14</td><td>2084</td><td rowspan="6">0.438</td><td rowspan="6">NA</td></tr>
<tr><td>Head and Neck</td><td>9</td><td>NA</td></tr>
<tr><td>Trunk</td><td>7</td><td>1322</td></tr>
<tr><td>Buttock</td><td>3</td><td>NA</td></tr>
<tr><td>Upper Limb</td><td>6</td><td>NA</td></tr>
<tr><td>Lower Limb</td><td>10</td><td>619</td></tr>
<tr><td>< 65 years old</td><td>26</td><td>NA</td><td rowspan="2">0.195</td><td rowspan="2">NA</td></tr>
<tr><td>> =5 years old</td><td>23</td><td>1620</td></tr>
<tr><td rowspan="9">StageI/II</td><td>MCC-LO</td><td>10</td><td>NA</td><td rowspan="2">0.135</td><td rowspan="2">0.941</td></tr>
<tr><td>MCC-HI</td><td>7</td><td>1365</td></tr>
<tr><td>Head and Neck</td><td>4</td><td>1643</td><td rowspan="5">0.438</td><td rowspan="5">NA</td></tr>
<tr><td>Trunk</td><td>5</td><td>1365</td></tr>
<tr><td>Buttock</td><td>2</td><td>849</td></tr>
<tr><td>Upper Limb</td><td>1</td><td>NA</td></tr>
<tr><td>Lower Limb</td><td>5</td><td>NA</td></tr>
<tr><td>< 65 years old</td><td>10</td><td>NA</td><td rowspan="2">0.032</td><td rowspan="2">0.748</td></tr>
<tr><td>> =65 years old</td><td>7</td><td>1365</td></tr>
</table>

NA, Median survival not reached.

Supplementary Table S10: Summary of MHC class I molecules identified in our MCC cohort

Sample	# of SSNV	# of Neoantigens	HLA haplotypes
MCC39	4707	236	B1801
MCC11	2618	267	A0301
MCC44	2330	251	A0301
MCC3	1855	435	A2402,A3301,C0602,C0702
MCC56	982	378	A0201,A2301,B4403,B5101,C0401,C0701
MCC33	881	110	A2402,B0702
MCC54	813	287	A0301,A2601,B0702,C0701,C0702
MCC35	603	107	A0101,A0201
MCC48	549	87	A0101,B0801,C0701
MCC21	534	80	A0201
MCC42	307	81	A0201,A0201,B3503,B4402,C0401,C0501
MCC47	187	1	A0101
MCC9	42	14	A0201,A2402,B1402,B4001,C0802
MCC8	39	7	A0201
MCC16	31	2	A0301
MCC58	29	7	A0201
MCC15	27	17	A0201,A0301,B1501,B1501
MCC23	19	23	A0301,A6801,B1402,B5101,C0802,C1402
MCC53	18	9	A1101,B5301,C0401,C0701
MCC57	18	2	A2601,A3002,B3801,C0701,C1203
MCC19	10	2	A0201,A2402,B0702,C0303,C0401
MCC20	10	4	A0201,A2301,B2705
MCC41	6	5	A0201,A0201,B1801,C0303,C0701

# of neoantigens reflect the number of mutated peptides with high affinity to one of the sample's MHC class I molecules. HLA haplotypes were identified by ATHLATES.

Supplementary Table S11: Table of predicted neoantigens identified in MCCs
Ka represents binding affinity of mutant nonamer to HLA allele.

See Supplementary File 2.
