# Supplementary material for: Mutational landscape of MCPyV-positive and MCPyV-negative Merkel cell carcinomas with implications for immunotherapy
Source: Oncotarget. 2015 Dec 7;7(3):3403–15. doi: 10.18632/oncotarget.6494 (PMC4823115; doi:10.18632/oncotarget.6494)
Supplement: Supplementary file 2 [file oncotarget-07-3403-s002.pdf]

| Gene | MCC_HI | | | | MCC_LO | | | | q-value |
|---|---|---|---|---|---|---|---|---|---|
| | Missense | Nonsense | Splice_site | Indels | Missense | Nonsense | Splice_site | Indels | |
| TP53 | 23 | 5 | 3 | 0 | 0 | 0 | 0 | 0 | 1.03E-03 |
| C5orf58 | 4 | 1 | 0 | 0 | 0 | 0 | 0 | 0 | 4.18E-01 |
| TTN | 175 | 13 | 2 | 0 | 1 | 0 | 0 | 0 | 1 |
| MUC16 | 104 | 6 | 0 | 0 | 1 | 0 | 0 | 0 | 1 |
| HYDIN | 52 | 3 | 1 | 0 | 0 | 1 | 0 | 0 | 1 |
| ABCA13 | 47 | 4 | 1 | 0 | 0 | 0 | 0 | 0 | 1 |
| RYR2 | 45 | 3 | 1 | 0 | 0 | 0 | 0 | 0 | 1 |
| CSMD3 | 36 | 4 | 2 | 0 | 0 | 0 | 0 | 0 | 1 |
| DNAH5 | 32 | 5 | 2 | 0 | 1 | 0 | 0 | 0 | 1 |
| PKHD1L1 | 37 | 2 | 0 | 0 | 0 | 0 | 0 | 0 | 1 |
| PCLO | 36 | 2 | 1 | 0 | 0 | 0 | 0 | 0 | 1 |
| LRP1B | 29 | 3 | 0 | 0 | 1 | 0 | 0 | 0 | 1 |
| GPR98 | 28 | 5 | 0 | 0 | 0 | 0 | 0 | 0 | 1 |
| SYNE1 | 28 | 1 | 2 | 0 | 2 | 0 | 0 | 0 | 1 |
| COL11A1 | 29 | 1 | 1 | 0 | 0 | 0 | 0 | 0 | 1 |
| CSMD1 | 25 | 5 | 0 | 0 | 0 | 0 | 0 | 0 | 1 |
| APOB | 25 | 3 | 1 | 0 | 0 | 0 | 0 | 0 | 1 |
| DNAH6 | 22 | 3 | 2 | 0 | 1 | 0 | 0 | 0 | 1 |
| RELN | 23 | 3 | 1 | 0 | 0 | 0 | 0 | 0 | 1 |
| PKHD1 | 22 | 5 | 0 | 0 | 0 | 0 | 0 | 0 | 1 |
| DNAH8 | 22 | 4 | 1 | 0 | 0 | 0 | 0 | 0 | 1 |
| USH2A | 25 | 2 | 0 | 0 | 0 | 0 | 0 | 0 | 1 |
| FAT3 | 24 | 1 | 0 | 0 | 0 | 0 | 0 | 0 | 1 |
| IGFN1 | 20 | 1 | 0 | 0 | 4 | 0 | 0 | 0 | 1 |
| RYR3 | 21 | 2 | 2 | 0 | 0 | 0 | 0 | 0 | 1 |
| DNAH14 | 23 | 1 | 0 | 0 | 0 | 0 | 0 | 0 | 1 |
| DNAH9 | 20 | 4 | 0 | 0 | 0 | 0 | 0 | 0 | 1 |
| THSD7A | 20 | 3 | 0 | 0 | 0 | 0 | 0 | 0 | 1 |
| DYNC2H1 | 19 | 1 | 1 | 0 | 1 | 0 | 0 | 0 | 1 |
| C1orf173 | 18 | 3 | 1 | 0 | 0 | 0 | 0 | 0 | 1 |
| EYS | 21 | 0 | 1 | 0 | 0 | 0 | 0 | 0 | 1 |
| LRP2 | 20 | 1 | 1 | 0 | 0 | 0 | 0 | 0 | 1 |
| SPTA1 | 18 | 1 | 1 | 0 | 1 | 0 | 0 | 0 | 1 |
| DNAH10 | 21 | 0 | 0 | 0 | 0 | 0 | 0 | 0 | 1 |
| SI | 18 | 1 | 0 | 0 | 0 | 1 | 0 | 0 | 1 |
| MYH8 | 20 | 0 | 0 | 0 | 0 | 0 | 0 | 0 | 1 |
| ZFHX4 | 19 | 1 | 0 | 0 | 0 | 0 | 0 | 0 | 1 |
| CUBN | 18 | 1 | 1 | 0 | 0 | 0 | 0 | 0 | 1 |
| BAI3 | 15 | 4 | 0 | 0 | 0 | 0 | 0 | 0 | 1 |
| CSMD2 | 15 | 3 | 1 | 0 | 0 | 0 | 0 | 0 | 1 |
| COL22A1 | 18 | 1 | 0 | 0 | 0 | 0 | 0 | 0 | 1 |
| ANK2 | 18 | 0 | 1 | 0 | 0 | 0 | 0 | 0 | 1 |
| SYNE2 | 17 | 0 | 1 | 1 | 0 | 0 | 0 | 0 | 1 |
| FAT4 | 18 | 0 | 0 | 0 | 1 | 0 | 0 | 0 | 1 |

| | | | | | | | | | |
|---|---|---|---|---|---|---|---|---|---|
| HMCN1 | 18 | 1 | 0 | 0 | 0 | 0 | 0 | 0 | 1 |
| NEB | 19 | 0 | 0 | 0 | 0 | 0 | 0 | 0 | 1 |
| LOXHD1 | 18 | 0 | 0 | 0 | 0 | 0 | 0 | 0 | 1 |
| RYR1 | 14 | 1 | 2 | 0 | 0 | 0 | 1 | 0 | 1 |
| DSCAM | 15 | 3 | 0 | 0 | 0 | 0 | 0 | 0 | 1 |
| DNAH11 | 16 | 2 | 0 | 0 | 0 | 0 | 0 | 0 | 1 |
| COL4A1 | 17 | 1 | 0 | 0 | 0 | 0 | 0 | 0 | 1 |
| RB1 | 4 | 7 | 6 | 0 | 0 | 0 | 0 | 0 | 1 |
| CD163 | 14 | 1 | 1 | 0 | 1 | 0 | 0 | 0 | 1 |
| FILIP1 | 16 | 1 | 0 | 0 | 0 | 0 | 0 | 0 | 1 |
| DPP10 | 17 | 0 | 0 | 0 | 0 | 0 | 0 | 0 | 1 |
| COL5A1 | 17 | 0 | 0 | 0 | 0 | 0 | 0 | 0 | 1 |
| COL6A5 | 16 | 1 | 0 | 0 | 0 | 0 | 0 | 0 | 1 |
| FRY | 14 | 3 | 0 | 0 | 0 | 0 | 0 | 0 | 1 |
| PAPPA2 | 17 | 0 | 0 | 0 | 0 | 0 | 0 | 0 | 1 |
| PKD1L1 | 16 | 0 | 1 | 0 | 0 | 0 | 0 | 0 | 1 |
| MYO18B | 16 | 1 | 0 | 0 | 0 | 0 | 0 | 0 | 1 |
| AHNAK | 17 | 0 | 0 | 0 | 0 | 0 | 0 | 0 | 1 |
| HEATR7B2 | 15 | 1 | 0 | 0 | 0 | 0 | 0 | 0 | 1 |
| ZNF804B | 16 | 0 | 0 | 0 | 0 | 0 | 0 | 0 | 1 |
| F5 | 13 | 1 | 1 | 0 | 1 | 0 | 0 | 0 | 1 |
| FLG2 | 12 | 4 | 0 | 0 | 0 | 0 | 0 | 0 | 1 |
| RP1 | 16 | 0 | 0 | 0 | 0 | 0 | 0 | 0 | 1 |
| PLCH1 | 14 | 2 | 0 | 0 | 0 | 0 | 0 | 0 | 1 |
| ODZ1 | 14 | 2 | 0 | 0 | 0 | 0 | 0 | 0 | 1 |
| COL24A1 | 15 | 0 | 1 | 0 | 0 | 0 | 0 | 0 | 1 |
| MYH2 | 15 | 0 | 1 | 0 | 0 | 0 | 0 | 0 | 1 |
| PEG3 | 13 | 3 | 0 | 0 | 0 | 0 | 0 | 0 | 1 |
| RIMS1 | 15 | 0 | 1 | 0 | 0 | 0 | 0 | 0 | 1 |
| COL1A2 | 16 | 0 | 0 | 0 | 0 | 0 | 0 | 0 | 1 |
| ASPM | 13 | 3 | 0 | 0 | 0 | 0 | 0 | 0 | 1 |
| SFMBT2 | 16 | 0 | 0 | 0 | 0 | 0 | 0 | 0 | 1 |
| XIRP2 | 14 | 2 | 0 | 0 | 0 | 0 | 0 | 0 | 1 |
| FAM135B | 15 | 0 | 1 | 0 | 0 | 0 | 0 | 0 | 1 |
| ITGA11 | 14 | 1 | 0 | 0 | 0 | 0 | 0 | 0 | 1 |
| SPHKAP | 14 | 1 | 0 | 0 | 0 | 0 | 0 | 0 | 1 |
| COL4A3 | 12 | 1 | 2 | 0 | 0 | 0 | 0 | 0 | 1 |
| TMEM132B | 15 | 0 | 0 | 0 | 0 | 0 | 0 | 0 | 1 |
| RFX6 | 15 | 0 | 0 | 0 | 0 | 0 | 0 | 0 | 1 |
| SVEP1 | 14 | 0 | 0 | 0 | 0 | 0 | 1 | 0 | 1 |
| CACNA1E | 13 | 2 | 0 | 0 | 0 | 0 | 0 | 0 | 1 |
| UNC13C | 14 | 1 | 0 | 0 | 0 | 0 | 0 | 0 | 1 |
| NAV3 | 14 | 0 | 1 | 0 | 0 | 0 | 0 | 0 | 1 |
| FBN3 | 14 | 1 | 0 | 0 | 0 | 0 | 0 | 0 | 1 |
| ACAN | 15 | 0 | 0 | 0 | 0 | 0 | 0 | 0 | 1 |
| DMD | 14 | 1 | 0 | 0 | 0 | 0 | 0 | 0 | 1 |

| | | | | | | | | | |
|---|---|---|---|---|---|---|---|---|---|
| SHANK2 | 12 | 3 | 0 | 0 | 0 | 0 | 0 | 0 | 1 |
| PPFIA2 | 14 | 1 | 0 | 0 | 0 | 0 | 0 | 0 | 1 |
| DCHS2 | 15 | 0 | 0 | 0 | 0 | 0 | 0 | 0 | 1 |
| VCAN | 15 | 0 | 0 | 0 | 0 | 0 | 0 | 0 | 1 |
| LAMA1 | 14 | 1 | 0 | 0 | 0 | 0 | 0 | 0 | 1 |
| ROS1 | 12 | 2 | 0 | 0 | 1 | 0 | 0 | 0 | 1 |
| CPAMD8 | 10 | 2 | 1 | 0 | 1 | 0 | 0 | 0 | 1 |
| TRPM6 | 11 | 2 | 1 | 0 | 0 | 0 | 0 | 0 | 1 |
| DOCK2 | 13 | 1 | 0 | 0 | 0 | 0 | 0 | 0 | 1 |
| DNAH2 | 12 | 2 | 0 | 0 | 0 | 0 | 0 | 0 | 1 |
| ABCA4 | 13 | 1 | 0 | 0 | 0 | 0 | 0 | 0 | 1 |
| DNAH7 | 14 | 0 | 0 | 0 | 0 | 0 | 0 | 0 | 1 |
| PREX2 | 14 | 0 | 0 | 0 | 0 | 0 | 0 | 0 | 1 |
| MYH4 | 13 | 0 | 1 | 0 | 0 | 0 | 0 | 0 | 1 |
| MYCBP2 | 11 | 2 | 1 | 0 | 0 | 0 | 0 | 0 | 1 |
| MACF1 | 14 | 0 | 0 | 0 | 0 | 0 | 0 | 0 | 1 |
| ANO2 | 10 | 2 | 1 | 0 | 0 | 0 | 0 | 0 | 1 |
| SCN9A | 10 | 2 | 1 | 0 | 0 | 0 | 0 | 0 | 1 |
| MYO16 | 11 | 1 | 1 | 0 | 0 | 0 | 0 | 0 | 1 |
| UNC80 | 9 | 1 | 2 | 0 | 0 | 1 | 0 | 0 | 1 |
| COL2A1 | 12 | 1 | 0 | 0 | 0 | 0 | 0 | 0 | 1 |
| DYSF | 12 | 0 | 0 | 0 | 1 | 0 | 0 | 0 | 1 |
| ABCC9 | 11 | 1 | 0 | 0 | 1 | 0 | 0 | 0 | 1 |
| TNR | 12 | 1 | 0 | 0 | 0 | 0 | 0 | 0 | 1 |
| DCC | 11 | 1 | 0 | 0 | 1 | 0 | 0 | 0 | 1 |
| PLCE1 | 13 | 0 | 0 | 0 | 0 | 0 | 0 | 0 | 1 |
| ITPR2 | 12 | 0 | 0 | 0 | 1 | 0 | 0 | 0 | 1 |
| COL4A4 | 13 | 0 | 0 | 0 | 0 | 0 | 0 | 0 | 1 |
| CTNNA2 | 11 | 1 | 0 | 0 | 1 | 0 | 0 | 0 | 1 |
| DST | 12 | 1 | 0 | 0 | 0 | 0 | 0 | 0 | 1 |
| FAT1 | 9 | 4 | 0 | 0 | 0 | 0 | 0 | 0 | 1 |
| C6 | 13 | 0 | 0 | 0 | 0 | 0 | 0 | 0 | 1 |
| COL14A1 | 11 | 2 | 0 | 0 | 0 | 0 | 0 | 0 | 1 |
| FRAS1 | 12 | 1 | 0 | 0 | 0 | 0 | 0 | 0 | 1 |
| CMYA5 | 12 | 1 | 0 | 0 | 0 | 0 | 0 | 0 | 1 |
| PDZD2 | 12 | 1 | 0 | 0 | 0 | 0 | 0 | 0 | 1 |
| NALCN | 12 | 1 | 0 | 0 | 0 | 0 | 0 | 0 | 1 |
| CLSTN2 | 12 | 0 | 0 | 0 | 0 | 0 | 0 | 0 | 1 |
| DCLK1 | 10 | 2 | 0 | 0 | 0 | 0 | 0 | 0 | 1 |
| CDH10 | 10 | 1 | 0 | 0 | 1 | 0 | 0 | 0 | 1 |
| MUC4 | 12 | 0 | 0 | 0 | 0 | 0 | 0 | 0 | 1 |
| MYH6 | 11 | 1 | 0 | 0 | 0 | 0 | 0 | 0 | 1 |
| TMPRSS15 | 12 | 0 | 0 | 0 | 0 | 0 | 0 | 0 | 1 |
| SPEF2 | 12 | 0 | 0 | 0 | 0 | 0 | 0 | 0 | 1 |
| PTPRQ | 10 | 2 | 0 | 0 | 0 | 0 | 0 | 0 | 1 |
| WDR7 | 12 | 0 | 0 | 0 | 0 | 0 | 0 | 0 | 1 |

| | | | | | | | | | |
|---|---|---|---|---|---|---|---|---|---|
| CNTNAP2 | 12 | 0 | 0 | 0 | 0 | 0 | 0 | 0 | 1 |
| ALPK2 | 12 | 0 | 0 | 0 | 0 | 0 | 0 | 0 | 1 |
| EPHA7 | 12 | 0 | 0 | 0 | 0 | 0 | 0 | 0 | 1 |
| DNAH12 | 11 | 0 | 0 | 0 | 1 | 0 | 0 | 0 | 1 |
| KALRN | 10 | 1 | 1 | 0 | 0 | 0 | 0 | 0 | 1 |
| MYH1 | 10 | 1 | 1 | 0 | 0 | 0 | 0 | 0 | 1 |
| WDFY4 | 10 | 2 | 0 | 0 | 0 | 0 | 0 | 0 | 1 |
| SDK1 | 10 | 2 | 0 | 0 | 0 | 0 | 0 | 0 | 1 |
| NBEA | 11 | 1 | 0 | 0 | 0 | 0 | 0 | 0 | 1 |
| MLL2 | 9 | 2 | 1 | 0 | 0 | 0 | 0 | 0 | 1 |
| COL12A1 | 11 | 1 | 0 | 0 | 0 | 0 | 0 | 0 | 1 |
| MLL | 10 | 2 | 0 | 0 | 0 | 0 | 0 | 0 | 1 |
| ATP10A | 12 | 0 | 0 | 0 | 0 | 0 | 0 | 0 | 1 |
| PCNX | 9 | 2 | 1 | 0 | 0 | 0 | 0 | 0 | 1 |
| LAMA2 | 11 | 0 | 0 | 0 | 1 | 0 | 0 | 0 | 1 |
| PRUNE2 | 12 | 0 | 0 | 0 | 0 | 0 | 0 | 0 | 1 |
| FAT2 | 11 | 1 | 0 | 0 | 0 | 0 | 0 | 0 | 1 |
| TG | 12 | 0 | 0 | 0 | 0 | 0 | 0 | 0 | 1 |
| MUC17 | 12 | 0 | 0 | 0 | 0 | 0 | 0 | 0 | 1 |
| PRKDC | 11 | 1 | 0 | 0 | 0 | 0 | 0 | 0 | 1 |
| PTPRD | 11 | 1 | 0 | 0 | 0 | 0 | 0 | 0 | 1 |
| VPS13D | 12 | 0 | 0 | 0 | 0 | 0 | 0 | 0 | 1 |
| FER1L6 | 12 | 0 | 0 | 0 | 0 | 0 | 0 | 0 | 1 |
| PCDH15 | 11 | 0 | 1 | 0 | 0 | 0 | 0 | 0 | 1 |
| SCN1A | 12 | 0 | 0 | 0 | 0 | 0 | 0 | 0 | 1 |
| HSPG2 | 11 | 0 | 1 | 0 | 0 | 0 | 0 | 0 | 1 |
| TRPA1 | 10 | 1 | 0 | 0 | 0 | 0 | 0 | 0 | 1 |
| ITGA8 | 9 | 2 | 0 | 0 | 0 | 0 | 0 | 0 | 1 |
| CACNA1F | 10 | 1 | 0 | 0 | 0 | 0 | 0 | 0 | 1 |
| GREB1L | 10 | 1 | 0 | 0 | 0 | 0 | 0 | 0 | 1 |
| DBC1 | 10 | 1 | 0 | 0 | 0 | 0 | 0 | 0 | 1 |
| MASP1 | 10 | 0 | 1 | 0 | 0 | 0 | 0 | 0 | 1 |
| COL9A1 | 11 | 0 | 0 | 0 | 0 | 0 | 0 | 0 | 1 |
| SCN7A | 10 | 1 | 0 | 0 | 0 | 0 | 0 | 0 | 1 |
| CNTNAP4 | 11 | 0 | 0 | 0 | 0 | 0 | 0 | 0 | 1 |
| SCN3A | 11 | 0 | 0 | 0 | 0 | 0 | 0 | 0 | 1 |
| LRRIQ1 | 10 | 1 | 0 | 0 | 0 | 0 | 0 | 0 | 1 |
| ASTN2 | 10 | 1 | 0 | 0 | 0 | 0 | 0 | 0 | 1 |
| CACNA1A | 9 | 1 | 1 | 0 | 0 | 0 | 0 | 0 | 1 |
| NRCAM | 10 | 1 | 0 | 0 | 0 | 0 | 0 | 0 | 1 |
| MME | 10 | 0 | 1 | 0 | 0 | 0 | 0 | 0 | 1 |
| NRXN3 | 9 | 1 | 0 | 0 | 1 | 0 | 0 | 0 | 1 |
| TEX15 | 11 | 0 | 0 | 0 | 0 | 0 | 0 | 0 | 1 |
| ASH1L | 11 | 0 | 0 | 0 | 0 | 0 | 0 | 0 | 1 |
| GRIN2A | 11 | 0 | 0 | 0 | 0 | 0 | 0 | 0 | 1 |
| ADAMTS20 | 7 | 3 | 1 | 0 | 0 | 0 | 0 | 0 | 1 |

| | | | | | | | | | |
|---|---|---|---|---|---|---|---|---|---|
| CACNA1G | 9 | 2 | 0 | 0 | 0 | 0 | 0 | 0 | 1 |
| TAF1L | 11 | 0 | 0 | 0 | 0 | 0 | 0 | 0 | 1 |
| OTOF | 11 | 0 | 0 | 0 | 0 | 0 | 0 | 0 | 1 |
| COL4A2 | 10 | 1 | 0 | 0 | 0 | 0 | 0 | 0 | 1 |
| STAB2 | 10 | 0 | 1 | 0 | 0 | 0 | 0 | 0 | 1 |
| CUX2 | 10 | 0 | 1 | 0 | 0 | 0 | 0 | 0 | 1 |
| MTUS2 | 11 | 0 | 0 | 0 | 0 | 0 | 0 | 0 | 1 |
| COL3A1 | 10 | 1 | 0 | 0 | 0 | 0 | 0 | 0 | 1 |
| STARD9 | 9 | 2 | 0 | 0 | 0 | 0 | 0 | 0 | 1 |
| SORL1 | 10 | 1 | 0 | 0 | 0 | 0 | 0 | 0 | 1 |
| MLL3 | 9 | 1 | 1 | 0 | 0 | 0 | 0 | 0 | 1 |
| CFH | 11 | 0 | 0 | 0 | 0 | 0 | 0 | 0 | 1 |
| VPS13B | 10 | 1 | 0 | 0 | 0 | 0 | 0 | 0 | 1 |
| KCNT2 | 10 | 0 | 1 | 0 | 0 | 0 | 0 | 0 | 1 |
| DOCK4 | 10 | 1 | 0 | 0 | 0 | 0 | 0 | 0 | 1 |
| SACS | 11 | 0 | 0 | 0 | 0 | 0 | 0 | 0 | 1 |
| DSCAML1 | 9 | 1 | 1 | 0 | 0 | 0 | 0 | 0 | 1 |
| LYST | 10 | 1 | 0 | 0 | 0 | 0 | 0 | 0 | 1 |
| OBSCN | 11 | 0 | 0 | 0 | 0 | 0 | 0 | 0 | 1 |
| DNAH3 | 11 | 0 | 0 | 0 | 0 | 0 | 0 | 0 | 1 |
| CNTNAP5 | 11 | 0 | 0 | 0 | 0 | 0 | 0 | 0 | 1 |
| GPR64 | 9 | 0 | 1 | 0 | 0 | 0 | 0 | 0 | 1 |
| PCDHA10 | 8 | 1 | 0 | 0 | 1 | 0 | 0 | 0 | 1 |
| FBXO40 | 9 | 1 | 0 | 0 | 0 | 0 | 0 | 0 | 1 |
| C1orf129 | 7 | 2 | 1 | 0 | 0 | 0 | 0 | 0 | 1 |
| EML6 | 8 | 2 | 0 | 0 | 0 | 0 | 0 | 0 | 1 |
| SLCO1B1 | 8 | 0 | 2 | 0 | 0 | 0 | 0 | 0 | 1 |
| ZBBX | 10 | 0 | 0 | 0 | 0 | 0 | 0 | 0 | 1 |
| FN1 | 9 | 0 | 1 | 0 | 0 | 0 | 0 | 0 | 1 |
| USP54 | 9 | 0 | 1 | 0 | 0 | 0 | 0 | 0 | 1 |
| SLIT2 | 10 | 0 | 0 | 0 | 0 | 0 | 0 | 0 | 1 |
| ZNF521 | 10 | 0 | 0 | 0 | 0 | 0 | 0 | 0 | 1 |
| NES | 10 | 0 | 0 | 0 | 0 | 0 | 0 | 0 | 1 |
| EPHA6 | 9 | 1 | 0 | 0 | 0 | 0 | 0 | 0 | 1 |
| CTNND2 | 10 | 0 | 0 | 0 | 0 | 0 | 0 | 0 | 1 |
| C2CD3 | 10 | 0 | 0 | 0 | 0 | 0 | 0 | 0 | 1 |
| MYH7 | 10 | 0 | 0 | 0 | 0 | 0 | 0 | 0 | 1 |
| PLCB4 | 9 | 1 | 0 | 0 | 0 | 0 | 0 | 0 | 1 |
| HECW1 | 10 | 0 | 0 | 0 | 0 | 0 | 0 | 0 | 1 |
| DLC1 | 9 | 1 | 0 | 0 | 0 | 0 | 0 | 0 | 1 |
| MUC2 | 9 | 1 | 0 | 0 | 0 | 0 | 0 | 0 | 1 |
| ASTN1 | 9 | 1 | 0 | 0 | 0 | 0 | 0 | 0 | 1 |
| TTC28 | 9 | 1 | 0 | 0 | 0 | 0 | 0 | 0 | 1 |
| LRRK2 | 9 | 1 | 0 | 0 | 0 | 0 | 0 | 0 | 1 |
| MAGEC1 | 10 | 0 | 0 | 0 | 0 | 0 | 0 | 0 | 1 |
| KNTC1 | 10 | 0 | 0 | 0 | 0 | 0 | 0 | 0 | 1 |

| | | | | | | | | | |
|---|---|---|---|---|---|---|---|---|---|
| PLXNA4 | 10 | 0 | 0 | 0 | 0 | 0 | 0 | 0 | 1 |
| DAPK1 | 9 | 0 | 1 | 0 | 0 | 0 | 0 | 0 | 1 |
| KIAA1107 | 10 | 0 | 0 | 0 | 0 | 0 | 0 | 0 | 1 |
| FBN1 | 9 | 1 | 0 | 0 | 0 | 0 | 0 | 0 | 1 |
| DOCK6 | 10 | 0 | 0 | 0 | 0 | 0 | 0 | 0 | 1 |
| MAGI2 | 10 | 0 | 0 | 0 | 0 | 0 | 0 | 0 | 1 |
| ALMS1 | 9 | 0 | 1 | 0 | 0 | 0 | 0 | 0 | 1 |
| C3 | 9 | 1 | 0 | 0 | 0 | 0 | 0 | 0 | 1 |
| COL15A1 | 10 | 0 | 0 | 0 | 0 | 0 | 0 | 0 | 1 |
| KIAA1109 | 8 | 1 | 1 | 0 | 0 | 0 | 0 | 0 | 1 |
| FMN2 | 10 | 0 | 0 | 0 | 0 | 0 | 0 | 0 | 1 |
| NRXN1 | 10 | 0 | 0 | 0 | 0 | 0 | 0 | 0 | 1 |
| EPHA5 | 10 | 0 | 0 | 0 | 0 | 0 | 0 | 0 | 1 |
| FREM1 | 9 | 1 | 0 | 0 | 0 | 0 | 0 | 0 | 1 |
| KIAA0947 | 9 | 1 | 0 | 0 | 0 | 0 | 0 | 0 | 1 |
| KIF13B | 9 | 1 | 0 | 0 | 0 | 0 | 0 | 0 | 1 |
| UNC5D | 9 | 0 | 1 | 0 | 0 | 0 | 0 | 0 | 1 |
| TMEM132D | 9 | 0 | 0 | 0 | 1 | 0 | 0 | 0 | 1 |
| UBR4 | 9 | 1 | 0 | 0 | 0 | 0 | 0 | 0 | 1 |
| FBN2 | 10 | 0 | 0 | 0 | 0 | 0 | 0 | 0 | 1 |
| CACNA1C | 10 | 0 | 0 | 0 | 0 | 0 | 0 | 0 | 1 |
| CNBD1 | 10 | 0 | 0 | 0 | 0 | 0 | 0 | 0 | 1 |
| CDH7 | 8 | 1 | 0 | 0 | 0 | 0 | 0 | 0 | 1 |
| ADAM18 | 8 | 1 | 0 | 0 | 0 | 0 | 0 | 0 | 1 |
| PEX5L | 7 | 2 | 0 | 0 | 0 | 0 | 0 | 0 | 1 |
| NCAM2 | 8 | 1 | 0 | 0 | 0 | 0 | 0 | 0 | 1 |
| USP29 | 8 | 1 | 0 | 0 | 0 | 0 | 0 | 0 | 1 |
| SLC12A5 | 8 | 0 | 1 | 0 | 0 | 0 | 0 | 0 | 1 |
| URB1 | 7 | 2 | 0 | 0 | 0 | 0 | 0 | 0 | 1 |
| FAM186A | 7 | 2 | 0 | 0 | 0 | 0 | 0 | 0 | 1 |
| ADAM22 | 9 | 0 | 0 | 0 | 0 | 0 | 0 | 0 | 1 |
| HEG1 | 9 | 0 | 0 | 0 | 0 | 0 | 0 | 0 | 1 |
| ADAMTSL3 | 7 | 1 | 1 | 0 | 0 | 0 | 0 | 0 | 1 |
| HCN1 | 9 | 0 | 0 | 0 | 0 | 0 | 0 | 0 | 1 |
| NFASC | 9 | 0 | 0 | 0 | 0 | 0 | 0 | 0 | 1 |
| OVCH1 | 9 | 0 | 0 | 0 | 0 | 0 | 0 | 0 | 1 |
| KIF21A | 5 | 4 | 0 | 0 | 0 | 0 | 0 | 0 | 1 |
| FRMPD4 | 9 | 0 | 0 | 0 | 0 | 0 | 0 | 0 | 1 |
| DGKB | 8 | 1 | 0 | 0 | 0 | 0 | 0 | 0 | 1 |
| SCAND3 | 8 | 1 | 0 | 0 | 0 | 0 | 0 | 0 | 1 |
| CACNA2D1 | 9 | 0 | 0 | 0 | 0 | 0 | 0 | 0 | 1 |
| ADAMTS9 | 7 | 2 | 0 | 0 | 0 | 0 | 0 | 0 | 1 |
| PDGFRA | 8 | 0 | 0 | 0 | 1 | 0 | 0 | 0 | 1 |
| PDE10A | 9 | 0 | 0 | 0 | 0 | 0 | 0 | 0 | 1 |
| NUP153 | 9 | 0 | 0 | 0 | 0 | 0 | 0 | 0 | 1 |
| ZNF347 | 8 | 1 | 0 | 0 | 0 | 0 | 0 | 0 | 1 |

| | | | | | | | | | |
|---|---|---|---|---|---|---|---|---|---|
| CLTCL1 | 7 | 2 | 0 | 0 | 0 | 0 | 0 | 0 | 1 |
| ABCB1 | 8 | 0 | 1 | 0 | 0 | 0 | 0 | 0 | 1 |
| CHL1 | 9 | 0 | 0 | 0 | 0 | 0 | 0 | 0 | 1 |
| SORBS2 | 9 | 0 | 0 | 0 | 0 | 0 | 0 | 0 | 1 |
| TLN2 | 8 | 0 | 1 | 0 | 0 | 0 | 0 | 0 | 1 |
| ALPK3 | 9 | 0 | 0 | 0 | 0 | 0 | 0 | 0 | 1 |
| ERBB4 | 7 | 1 | 0 | 0 | 0 | 1 | 0 | 0 | 1 |
| PPP1R3A | 9 | 0 | 0 | 0 | 0 | 0 | 0 | 0 | 1 |
| CNTN5 | 9 | 0 | 0 | 0 | 0 | 0 | 0 | 0 | 1 |
| COL19A1 | 8 | 0 | 1 | 0 | 0 | 0 | 0 | 0 | 1 |
| SAMD9 | 9 | 0 | 0 | 0 | 0 | 0 | 0 | 0 | 1 |
| MYO7B | 9 | 0 | 0 | 0 | 0 | 0 | 0 | 0 | 1 |
| WBSCR17 | 8 | 1 | 0 | 0 | 0 | 0 | 0 | 0 | 1 |
| STXBP5L | 9 | 0 | 0 | 0 | 0 | 0 | 0 | 0 | 1 |
| RIF1 | 5 | 1 | 2 | 1 | 0 | 0 | 0 | 0 | 1 |
| AFF2 | 8 | 1 | 0 | 0 | 0 | 0 | 0 | 0 | 1 |
| PTPRB | 8 | 1 | 0 | 0 | 0 | 0 | 0 | 0 | 1 |
| PTPRC | 6 | 2 | 1 | 0 | 0 | 0 | 0 | 0 | 1 |
| SPAG17 | 9 | 0 | 0 | 0 | 0 | 0 | 0 | 0 | 1 |
| ZAN | 9 | 0 | 0 | 0 | 0 | 0 | 0 | 0 | 1 |
| AFF3 | 9 | 0 | 0 | 0 | 0 | 0 | 0 | 0 | 1 |
| HEATR5B | 8 | 1 | 0 | 0 | 0 | 0 | 0 | 0 | 1 |
| GPR158 | 8 | 0 | 1 | 0 | 0 | 0 | 0 | 0 | 1 |
| SORCS1 | 9 | 0 | 0 | 0 | 0 | 0 | 0 | 0 | 1 |
| FAM47A | 8 | 1 | 0 | 0 | 0 | 0 | 0 | 0 | 1 |
| HIVEP1 | 9 | 0 | 0 | 0 | 0 | 0 | 0 | 0 | 1 |
| TACC2 | 9 | 0 | 0 | 0 | 0 | 0 | 0 | 0 | 1 |
| GPR179 | 8 | 1 | 0 | 0 | 0 | 0 | 0 | 0 | 1 |
| WDR87 | 9 | 0 | 0 | 0 | 0 | 0 | 0 | 0 | 1 |
| UTRN | 8 | 0 | 0 | 0 | 1 | 0 | 0 | 0 | 1 |
| MGAM | 9 | 0 | 0 | 0 | 0 | 0 | 0 | 0 | 1 |
| RGSL1 | 8 | 0 | 1 | 0 | 0 | 0 | 0 | 0 | 1 |
| COL6A3 | 9 | 0 | 0 | 0 | 0 | 0 | 0 | 0 | 1 |
| CDH23 | 8 | 0 | 0 | 0 | 1 | 0 | 0 | 0 | 1 |
| MAP2 | 9 | 0 | 0 | 0 | 0 | 0 | 0 | 0 | 1 |
| MYO15A | 8 | 1 | 0 | 0 | 0 | 0 | 0 | 0 | 1 |
| GPR112 | 9 | 0 | 0 | 0 | 0 | 0 | 0 | 0 | 1 |
| SDK2 | 9 | 0 | 0 | 0 | 0 | 0 | 0 | 0 | 1 |
| WDFY3 | 8 | 1 | 0 | 0 | 0 | 0 | 0 | 0 | 1 |
| MYH13 | 9 | 0 | 0 | 0 | 0 | 0 | 0 | 0 | 1 |
| AGL | 9 | 0 | 0 | 0 | 0 | 0 | 0 | 0 | 1 |
| MKI67 | 9 | 0 | 0 | 0 | 0 | 0 | 0 | 0 | 1 |
| SRCAP | 9 | 0 | 0 | 0 | 0 | 0 | 0 | 0 | 1 |
| FREM2 | 8 | 1 | 0 | 0 | 0 | 0 | 0 | 0 | 1 |
| MDN1 | 9 | 0 | 0 | 0 | 0 | 0 | 0 | 0 | 1 |
| CEP350 | 8 | 1 | 0 | 0 | 0 | 0 | 0 | 0 | 1 |

| | | | | | | | | | |
|---|---|---|---|---|---|---|---|---|---|
| TRIOBP | 9 | 0 | 0 | 0 | 0 | 0 | 0 | 0 | 1 |
| LPA | 8 | 1 | 0 | 0 | 0 | 0 | 0 | 0 | 1 |
| ECE2 | 5 | 1 | 2 | 0 | 0 | 0 | 0 | 0 | 1 |
| SPATA16 | 8 | 0 | 0 | 0 | 0 | 0 | 0 | 0 | 1 |
| DPF3 | 8 | 0 | 0 | 0 | 0 | 0 | 0 | 0 | 1 |
| BMP5 | 8 | 0 | 0 | 0 | 0 | 0 | 0 | 0 | 1 |
| KHDRBS2 | 8 | 0 | 0 | 0 | 0 | 0 | 0 | 0 | 1 |
| ASPH | 7 | 1 | 0 | 0 | 0 | 0 | 0 | 0 | 1 |
| MEF2C | 7 | 0 | 1 | 0 | 0 | 0 | 0 | 0 | 1 |
| SLC15A2 | 8 | 0 | 0 | 0 | 0 | 0 | 0 | 0 | 1 |
| EFCAB4B | 7 | 1 | 0 | 0 | 0 | 0 | 0 | 0 | 1 |
| POP1 | 8 | 0 | 0 | 0 | 0 | 0 | 0 | 0 | 1 |
| TRPV5 | 7 | 1 | 0 | 0 | 0 | 0 | 0 | 0 | 1 |
| FCRL5 | 8 | 0 | 0 | 0 | 0 | 0 | 0 | 0 | 1 |
| USP28 | 6 | 0 | 1 | 0 | 1 | 0 | 0 | 0 | 1 |
| ZNF804A | 7 | 1 | 0 | 0 | 0 | 0 | 0 | 0 | 1 |
| BICD1 | 8 | 0 | 0 | 0 | 0 | 0 | 0 | 0 | 1 |
| TLR4 | 8 | 0 | 0 | 0 | 0 | 0 | 0 | 0 | 1 |
| TYR | 8 | 0 | 0 | 0 | 0 | 0 | 0 | 0 | 1 |
| MTHFD1L | 8 | 0 | 0 | 0 | 0 | 0 | 0 | 0 | 1 |
| GRIA1 | 8 | 0 | 0 | 0 | 0 | 0 | 0 | 0 | 1 |
| ANKRD12 | 7 | 0 | 0 | 0 | 1 | 0 | 0 | 0 | 1 |
| WDR17 | 6 | 2 | 0 | 0 | 0 | 0 | 0 | 0 | 1 |
| EYA1 | 5 | 3 | 0 | 0 | 0 | 0 | 0 | 0 | 1 |
| ABI3BP | 8 | 0 | 0 | 0 | 0 | 0 | 0 | 0 | 1 |
| LAMA4 | 8 | 0 | 0 | 0 | 0 | 0 | 0 | 0 | 1 |
| PZP | 8 | 0 | 0 | 0 | 0 | 0 | 0 | 0 | 1 |
| VWCE | 8 | 0 | 0 | 0 | 0 | 0 | 0 | 0 | 1 |
| IMPG2 | 8 | 0 | 0 | 0 | 0 | 0 | 0 | 0 | 1 |
| BCAS3 | 8 | 0 | 0 | 0 | 0 | 0 | 0 | 0 | 1 |
| ANKRD31 | 7 | 1 | 0 | 0 | 0 | 0 | 0 | 0 | 1 |
| TDRD1 | 6 | 2 | 0 | 0 | 0 | 0 | 0 | 0 | 1 |
| SYTL2 | 8 | 0 | 0 | 0 | 0 | 0 | 0 | 0 | 1 |
| CRB1 | 8 | 0 | 0 | 0 | 0 | 0 | 0 | 0 | 1 |
| TP53BP1 | 7 | 1 | 0 | 0 | 0 | 0 | 0 | 0 | 1 |
| PPP1R9A | 6 | 1 | 1 | 0 | 0 | 0 | 0 | 0 | 1 |
| DOCK10 | 7 | 1 | 0 | 0 | 0 | 0 | 0 | 0 | 1 |
| BCORL1 | 7 | 1 | 0 | 0 | 0 | 0 | 0 | 0 | 1 |
| SCN8A | 8 | 0 | 0 | 0 | 0 | 0 | 0 | 0 | 1 |
| NRD1 | 7 | 1 | 0 | 0 | 0 | 0 | 0 | 0 | 1 |
| PLCL2 | 8 | 0 | 0 | 0 | 0 | 0 | 0 | 0 | 1 |
| COL17A1 | 7 | 1 | 0 | 0 | 0 | 0 | 0 | 0 | 1 |
| HDAC9 | 8 | 0 | 0 | 0 | 0 | 0 | 0 | 0 | 1 |
| DLGAP3 | 8 | 0 | 0 | 0 | 0 | 0 | 0 | 0 | 1 |
| A2M | 7 | 1 | 0 | 0 | 0 | 0 | 0 | 0 | 1 |
| CDH6 | 8 | 0 | 0 | 0 | 0 | 0 | 0 | 0 | 1 |

| | | | | | | | | | |
|---|---|---|---|---|---|---|---|---|---|
| KIF1B | 8 | 0 | 0 | 0 | 0 | 0 | 0 | 0 | 1 |
| DMBT1 | 8 | 0 | 0 | 0 | 0 | 0 | 0 | 0 | 1 |
| GRIA2 | 7 | 1 | 0 | 0 | 0 | 0 | 0 | 0 | 1 |
| NBAS | 8 | 0 | 0 | 0 | 0 | 0 | 0 | 0 | 1 |
| WDR64 | 8 | 0 | 0 | 0 | 0 | 0 | 0 | 0 | 1 |
| DIDO1 | 7 | 1 | 0 | 0 | 0 | 0 | 0 | 0 | 1 |
| ABCA12 | 7 | 1 | 0 | 0 | 0 | 0 | 0 | 0 | 1 |
| TEP1 | 7 | 1 | 0 | 0 | 0 | 0 | 0 | 0 | 1 |
| LRRC7 | 8 | 0 | 0 | 0 | 0 | 0 | 0 | 0 | 1 |
| TNIK | 6 | 1 | 1 | 0 | 0 | 0 | 0 | 0 | 1 |
| HIVEP2 | 7 | 1 | 0 | 0 | 0 | 0 | 0 | 0 | 1 |
| KIF5A | 7 | 1 | 0 | 0 | 0 | 0 | 0 | 0 | 1 |
| NOTCH2 | 6 | 0 | 1 | 0 | 1 | 0 | 0 | 0 | 1 |
| NCOR2 | 8 | 0 | 0 | 0 | 0 | 0 | 0 | 0 | 1 |
| MYO5B | 8 | 0 | 0 | 0 | 0 | 0 | 0 | 0 | 1 |
| CENPE | 7 | 1 | 0 | 0 | 0 | 0 | 0 | 0 | 1 |
| PKD1L2 | 8 | 0 | 0 | 0 | 0 | 0 | 0 | 0 | 1 |
| TJP1 | 8 | 0 | 0 | 0 | 0 | 0 | 0 | 0 | 1 |
| FAM5C | 6 | 2 | 0 | 0 | 0 | 0 | 0 | 0 | 1 |
| MDGA2 | 8 | 0 | 0 | 0 | 0 | 0 | 0 | 0 | 1 |
| C5orf42 | 8 | 0 | 0 | 0 | 0 | 0 | 0 | 0 | 1 |
| NLRP3 | 7 | 1 | 0 | 0 | 0 | 0 | 0 | 0 | 1 |
| CUL9 | 8 | 0 | 0 | 0 | 0 | 0 | 0 | 0 | 1 |
| MORC1 | 8 | 0 | 0 | 0 | 0 | 0 | 0 | 0 | 1 |
| C15orf2 | 8 | 0 | 0 | 0 | 0 | 0 | 0 | 0 | 1 |
| ABCA6 | 6 | 1 | 1 | 0 | 0 | 0 | 0 | 0 | 1 |
| COL28A1 | 7 | 0 | 0 | 0 | 1 | 0 | 0 | 0 | 1 |
| HERC1 | 7 | 1 | 0 | 0 | 0 | 0 | 0 | 0 | 1 |
| MYO3B | 7 | 1 | 0 | 0 | 0 | 0 | 0 | 0 | 1 |
| CACNA1S | 7 | 0 | 1 | 0 | 0 | 0 | 0 | 0 | 1 |
| ODZ4 | 7 | 1 | 0 | 0 | 0 | 0 | 0 | 0 | 1 |
| ANK3 | 8 | 0 | 0 | 0 | 0 | 0 | 0 | 0 | 1 |
| DNAH17 | 8 | 0 | 0 | 0 | 0 | 0 | 0 | 0 | 1 |
| KIAA0564 | 8 | 0 | 0 | 0 | 0 | 0 | 0 | 0 | 1 |
| BRCA2 | 8 | 0 | 0 | 0 | 0 | 0 | 0 | 0 | 1 |
| DOCK3 | 8 | 0 | 0 | 0 | 0 | 0 | 0 | 0 | 1 |
| KSR2 | 8 | 0 | 0 | 0 | 0 | 0 | 0 | 0 | 1 |
| SSPO | 7 | 1 | 0 | 0 | 0 | 0 | 0 | 0 | 1 |
| TRIO | 8 | 0 | 0 | 0 | 0 | 0 | 0 | 0 | 1 |
| NRXN2 | 8 | 0 | 0 | 0 | 0 | 0 | 0 | 0 | 1 |
| LRP1 | 7 | 1 | 0 | 0 | 0 | 0 | 0 | 0 | 1 |
| F13B | 5 | 2 | 0 | 0 | 1 | 0 | 0 | 0 | 1 |
| C12orf51 | 8 | 0 | 0 | 0 | 0 | 0 | 0 | 0 | 1 |
| GRIN2B | 8 | 0 | 0 | 0 | 0 | 0 | 0 | 0 | 1 |
| VPS13C | 8 | 0 | 0 | 0 | 0 | 0 | 0 | 0 | 1 |
| CD5L | 6 | 1 | 0 | 0 | 0 | 0 | 0 | 0 | 1 |

| | | | | | | | | | |
|---|---|---|---|---|---|---|---|---|---|
| AOAH | 3 | 2 | 2 | 0 | 0 | 0 | 0 | 0 | 1 |
| SLC6A1 | 6 | 1 | 0 | 0 | 0 | 0 | 0 | 0 | 1 |
| OR2G6 | 7 | 0 | 0 | 0 | 0 | 0 | 0 | 0 | 1 |
| OXR1 | 5 | 2 | 0 | 0 | 0 | 0 | 0 | 0 | 1 |
| TTLL6 | 6 | 1 | 0 | 0 | 0 | 0 | 0 | 0 | 1 |
| FCRL4 | 5 | 2 | 0 | 0 | 0 | 0 | 0 | 0 | 1 |
| CCDC102B | 5 | 2 | 0 | 0 | 0 | 0 | 0 | 0 | 1 |
| URB2 | 5 | 2 | 0 | 0 | 0 | 0 | 0 | 0 | 1 |
| MASP2 | 5 | 1 | 0 | 0 | 1 | 0 | 0 | 0 | 1 |
| KIF5C | 6 | 0 | 0 | 1 | 0 | 0 | 0 | 0 | 1 |
| NLRP10 | 7 | 0 | 0 | 0 | 0 | 0 | 0 | 0 | 1 |
| NCAM1 | 4 | 2 | 1 | 0 | 0 | 0 | 0 | 0 | 1 |
| KCNQ5 | 6 | 1 | 0 | 0 | 0 | 0 | 0 | 0 | 1 |
| CDHR3 | 7 | 0 | 0 | 0 | 0 | 0 | 0 | 0 | 1 |
| DPPA4 | 7 | 0 | 0 | 0 | 0 | 0 | 0 | 0 | 1 |
| MSH4 | 6 | 1 | 0 | 0 | 0 | 0 | 0 | 0 | 1 |
| SP110 | 6 | 1 | 0 | 0 | 0 | 0 | 0 | 0 | 1 |
| APBB1IP | 7 | 0 | 0 | 0 | 0 | 0 | 0 | 0 | 1 |
| ACTN3 | 5 | 2 | 0 | 0 | 0 | 0 | 0 | 0 | 1 |
| CR2 | 6 | 0 | 0 | 0 | 1 | 0 | 0 | 0 | 1 |
| CCDC73 | 7 | 0 | 0 | 0 | 0 | 0 | 0 | 0 | 1 |
| TTLL11 | 7 | 0 | 0 | 0 | 0 | 0 | 0 | 0 | 1 |
| HHIPL2 | 7 | 0 | 0 | 0 | 0 | 0 | 0 | 0 | 1 |
| GPR110 | 5 | 1 | 1 | 0 | 0 | 0 | 0 | 0 | 1 |
| CCDC132 | 6 | 1 | 0 | 0 | 0 | 0 | 0 | 0 | 1 |
| KIF4B | 5 | 2 | 0 | 0 | 0 | 0 | 0 | 0 | 1 |
| INPP4B | 6 | 1 | 0 | 0 | 0 | 0 | 0 | 0 | 1 |
| CD180 | 6 | 1 | 0 | 0 | 0 | 0 | 0 | 0 | 1 |
| ALDH1A2 | 7 | 0 | 0 | 0 | 0 | 0 | 0 | 0 | 1 |
| RGS7 | 7 | 0 | 0 | 0 | 0 | 0 | 0 | 0 | 1 |
| ALS2CR11 | 6 | 1 | 0 | 0 | 0 | 0 | 0 | 0 | 1 |
| SELP | 7 | 0 | 0 | 0 | 0 | 0 | 0 | 0 | 1 |
| ELTD1 | 7 | 0 | 0 | 0 | 0 | 0 | 0 | 0 | 1 |
| DGKG | 7 | 0 | 0 | 0 | 0 | 0 | 0 | 0 | 1 |
| ATP1A2 | 7 | 0 | 0 | 0 | 0 | 0 | 0 | 0 | 1 |
| ACTRT1 | 7 | 0 | 0 | 0 | 0 | 0 | 0 | 0 | 1 |
| C15orf55 | 6 | 1 | 0 | 0 | 0 | 0 | 0 | 0 | 1 |
| PC | 7 | 0 | 0 | 0 | 0 | 0 | 0 | 0 | 1 |
| PDE4D | 7 | 0 | 0 | 0 | 0 | 0 | 0 | 0 | 1 |
| PAPLN | 7 | 0 | 0 | 0 | 0 | 0 | 0 | 0 | 1 |
| SAMD4A | 7 | 0 | 0 | 0 | 0 | 0 | 0 | 0 | 1 |
| ITGBL1 | 7 | 0 | 0 | 0 | 0 | 0 | 0 | 0 | 1 |
| GRXCR1 | 7 | 0 | 0 | 0 | 0 | 0 | 0 | 0 | 1 |
| KANK4 | 7 | 0 | 0 | 0 | 0 | 0 | 0 | 0 | 1 |
| CGN | 6 | 0 | 0 | 0 | 1 | 0 | 0 | 0 | 1 |
| GRM8 | 7 | 0 | 0 | 0 | 0 | 0 | 0 | 0 | 1 |

| | | | | | | | | | |
|---|---|---|---|---|---|---|---|---|---|
| ATP1A3 | 7 | 0 | 0 | 0 | 0 | 0 | 0 | 0 | 1 |
| SGIP1 | 7 | 0 | 0 | 0 | 0 | 0 | 0 | 0 | 1 |
| DSG2 | 7 | 0 | 0 | 0 | 0 | 0 | 0 | 0 | 1 |
| KIF14 | 5 | 2 | 0 | 0 | 0 | 0 | 0 | 0 | 1 |
| PRKD1 | 7 | 0 | 0 | 0 | 0 | 0 | 0 | 0 | 1 |
| POM121L2 | 7 | 0 | 0 | 0 | 0 | 0 | 0 | 0 | 1 |
| MAP2K3 | 7 | 0 | 0 | 0 | 0 | 0 | 0 | 0 | 1 |
| TET1 | 6 | 1 | 0 | 0 | 0 | 0 | 0 | 0 | 1 |
| C2orf71 | 6 | 1 | 0 | 0 | 0 | 0 | 0 | 0 | 1 |
| C5 | 7 | 0 | 0 | 0 | 0 | 0 | 0 | 0 | 1 |
| KIAA1755 | 7 | 0 | 0 | 0 | 0 | 0 | 0 | 0 | 1 |
| PCDH20 | 6 | 1 | 0 | 0 | 0 | 0 | 0 | 0 | 1 |
| NLGN1 | 6 | 1 | 0 | 0 | 0 | 0 | 0 | 0 | 1 |
| ADAMTS6 | 7 | 0 | 0 | 0 | 0 | 0 | 0 | 0 | 1 |
| GYS2 | 7 | 0 | 0 | 0 | 0 | 0 | 0 | 0 | 1 |
| TRPM3 | 6 | 1 | 0 | 0 | 0 | 0 | 0 | 0 | 1 |
| MMP16 | 5 | 2 | 0 | 0 | 0 | 0 | 0 | 0 | 1 |
| NDST4 | 7 | 0 | 0 | 0 | 0 | 0 | 0 | 0 | 1 |
| ABCA10 | 7 | 0 | 0 | 0 | 0 | 0 | 0 | 0 | 1 |
| LMO7 | 7 | 0 | 0 | 0 | 0 | 0 | 0 | 0 | 1 |
| ATP1A4 | 7 | 0 | 0 | 0 | 0 | 0 | 0 | 0 | 1 |
| TARBP1 | 5 | 2 | 0 | 0 | 0 | 0 | 0 | 0 | 1 |
| LGR6 | 7 | 0 | 0 | 0 | 0 | 0 | 0 | 0 | 1 |
| ST18 | 6 | 1 | 0 | 0 | 0 | 0 | 0 | 0 | 1 |
| CDH4 | 7 | 0 | 0 | 0 | 0 | 0 | 0 | 0 | 1 |
| C20orf194 | 7 | 0 | 0 | 0 | 0 | 0 | 0 | 0 | 1 |
| ZNF831 | 6 | 1 | 0 | 0 | 0 | 0 | 0 | 0 | 1 |
| C7 | 6 | 1 | 0 | 0 | 0 | 0 | 0 | 0 | 1 |
| KIF13A | 5 | 2 | 0 | 0 | 0 | 0 | 0 | 0 | 1 |
| LRRK1 | 6 | 0 | 0 | 0 | 1 | 0 | 0 | 0 | 1 |
| LPHN2 | 7 | 0 | 0 | 0 | 0 | 0 | 0 | 0 | 1 |
| SCN10A | 6 | 1 | 0 | 0 | 0 | 0 | 0 | 0 | 1 |
| CNTN4 | 7 | 0 | 0 | 0 | 0 | 0 | 0 | 0 | 1 |
| PLD1 | 7 | 0 | 0 | 0 | 0 | 0 | 0 | 0 | 1 |
| MED13 | 5 | 1 | 0 | 0 | 1 | 0 | 0 | 0 | 1 |
| DLEC1 | 7 | 0 | 0 | 0 | 0 | 0 | 0 | 0 | 1 |
| RGAG1 | 6 | 1 | 0 | 0 | 0 | 0 | 0 | 0 | 1 |
| CCDC108 | 6 | 1 | 0 | 0 | 0 | 0 | 0 | 0 | 1 |
| ARHGEF17 | 7 | 0 | 0 | 0 | 0 | 0 | 0 | 0 | 1 |
| SEC16B | 7 | 0 | 0 | 0 | 0 | 0 | 0 | 0 | 1 |
| SLC12A1 | 6 | 0 | 1 | 0 | 0 | 0 | 0 | 0 | 1 |
| ARAP2 | 7 | 0 | 0 | 0 | 0 | 0 | 0 | 0 | 1 |
| VWDE | 7 | 0 | 0 | 0 | 0 | 0 | 0 | 0 | 1 |
| CADPS2 | 6 | 0 | 0 | 0 | 1 | 0 | 0 | 0 | 1 |
| EPHA3 | 7 | 0 | 0 | 0 | 0 | 0 | 0 | 0 | 1 |
| FLNC | 6 | 0 | 1 | 0 | 0 | 0 | 0 | 0 | 1 |

| | | | | | | | | | |
|---|---|---|---|---|---|---|---|---|---|
| GRID1 | 7 | 0 | 0 | 0 | 0 | 0 | 0 | 0 | 1 |
| WDR78 | 6 | 0 | 0 | 0 | 1 | 0 | 0 | 0 | 1 |
| ENPP2 | 7 | 0 | 0 | 0 | 0 | 0 | 0 | 0 | 1 |
| RIMS2 | 7 | 0 | 0 | 0 | 0 | 0 | 0 | 0 | 1 |
| SLITRK3 | 6 | 0 | 0 | 0 | 1 | 0 | 0 | 0 | 1 |
| TNC | 7 | 0 | 0 | 0 | 0 | 0 | 0 | 0 | 1 |
| KIAA1239 | 7 | 0 | 0 | 0 | 0 | 0 | 0 | 0 | 1 |
| ABCC1 | 7 | 0 | 0 | 0 | 0 | 0 | 0 | 0 | 1 |
| SCN2A | 7 | 0 | 0 | 0 | 0 | 0 | 0 | 0 | 1 |
| CNTN6 | 7 | 0 | 0 | 0 | 0 | 0 | 0 | 0 | 1 |
| ADAMTS18 | 6 | 0 | 0 | 0 | 1 | 0 | 0 | 0 | 1 |
| CDC42BPA | 6 | 1 | 0 | 0 | 0 | 0 | 0 | 0 | 1 |
| SCN5A | 7 | 0 | 0 | 0 | 0 | 0 | 0 | 0 | 1 |
| PLEKHG1 | 7 | 0 | 0 | 0 | 0 | 0 | 0 | 0 | 1 |
| RTTN | 7 | 0 | 0 | 0 | 0 | 0 | 0 | 0 | 1 |
| F8 | 7 | 0 | 0 | 0 | 0 | 0 | 0 | 0 | 1 |
| TGM6 | 7 | 0 | 0 | 0 | 0 | 0 | 0 | 0 | 1 |
| C20orf26 | 7 | 0 | 0 | 0 | 0 | 0 | 0 | 0 | 1 |
| MED13L | 7 | 0 | 0 | 0 | 0 | 0 | 0 | 0 | 1 |
| PIK3CG | 6 | 1 | 0 | 0 | 0 | 0 | 0 | 0 | 1 |
| PCNT | 7 | 0 | 0 | 0 | 0 | 0 | 0 | 0 | 1 |
| FNDC1 | 7 | 0 | 0 | 0 | 0 | 0 | 0 | 0 | 1 |
| COL5A2 | 6 | 1 | 0 | 0 | 0 | 0 | 0 | 0 | 1 |
| ROBO2 | 6 | 1 | 0 | 0 | 0 | 0 | 0 | 0 | 1 |
| COL27A1 | 7 | 0 | 0 | 0 | 0 | 0 | 0 | 0 | 1 |
| CP | 7 | 0 | 0 | 0 | 0 | 0 | 0 | 0 | 1 |
| LPHN3 | 7 | 0 | 0 | 0 | 0 | 0 | 0 | 0 | 1 |
| LCT | 7 | 0 | 0 | 0 | 0 | 0 | 0 | 0 | 1 |
| DMXL2 | 4 | 3 | 0 | 0 | 0 | 0 | 0 | 0 | 1 |
| ANKS1B | 7 | 0 | 0 | 0 | 0 | 0 | 0 | 0 | 1 |
| KIAA0368 | 6 | 1 | 0 | 0 | 0 | 0 | 0 | 0 | 1 |
| KIAA1199 | 6 | 1 | 0 | 0 | 0 | 0 | 0 | 0 | 1 |
| ARHGAP29 | 7 | 0 | 0 | 0 | 0 | 0 | 0 | 0 | 1 |
| MAGEL2 | 6 | 1 | 0 | 0 | 0 | 0 | 0 | 0 | 1 |
| PCM1 | 7 | 0 | 0 | 0 | 0 | 0 | 0 | 0 | 1 |
| DOPEY1 | 7 | 0 | 0 | 0 | 0 | 0 | 0 | 0 | 1 |
| GOLGB1 | 4 | 3 | 0 | 0 | 0 | 0 | 0 | 0 | 1 |
| THOC2 | 7 | 0 | 0 | 0 | 0 | 0 | 0 | 0 | 1 |
| ZNF226 | 7 | 0 | 0 | 0 | 0 | 0 | 0 | 0 | 1 |
| ZNF717 | 6 | 1 | 0 | 0 | 0 | 0 | 0 | 0 | 1 |
| CXorf22 | 7 | 0 | 0 | 0 | 0 | 0 | 0 | 0 | 1 |
| ATP13A5 | 7 | 0 | 0 | 0 | 0 | 0 | 0 | 0 | 1 |
| CDH19 | 6 | 1 | 0 | 0 | 0 | 0 | 0 | 0 | 1 |
| PTPRM | 7 | 0 | 0 | 0 | 0 | 0 | 0 | 0 | 1 |
| NCOR1 | 7 | 0 | 0 | 0 | 0 | 0 | 0 | 0 | 1 |
| MYO7A | 7 | 0 | 0 | 0 | 0 | 0 | 0 | 0 | 1 |

| | | | | | | | | | |
|---|---|---|---|---|---|---|---|---|---|
| MYPN | 7 | 0 | 0 | 0 | 0 | 0 | 0 | 0 | 1 |
| GTF3C2 | 6 | 1 | 0 | 0 | 0 | 0 | 0 | 0 | 1 |
| TRRAP | 6 | 1 | 0 | 0 | 0 | 0 | 0 | 0 | 1 |
| HTT | 7 | 0 | 0 | 0 | 0 | 0 | 0 | 0 | 1 |
| PTPRK | 6 | 1 | 0 | 0 | 0 | 0 | 0 | 0 | 1 |
| DOCK7 | 5 | 2 | 0 | 0 | 0 | 0 | 0 | 0 | 1 |
| PROX1 | 7 | 0 | 0 | 0 | 0 | 0 | 0 | 0 | 1 |
| ATG2A | 7 | 0 | 0 | 0 | 0 | 0 | 0 | 0 | 1 |
| UBR5 | 7 | 0 | 0 | 0 | 0 | 0 | 0 | 0 | 1 |
| ATP13A3 | 7 | 0 | 0 | 0 | 0 | 0 | 0 | 0 | 1 |
| C11orf41 | 5 | 1 | 0 | 0 | 1 | 0 | 0 | 0 | 1 |
| DSP | 6 | 1 | 0 | 0 | 0 | 0 | 0 | 0 | 1 |
| BRWD1 | 6 | 1 | 0 | 0 | 0 | 0 | 0 | 0 | 1 |
| CENPF | 5 | 2 | 0 | 0 | 0 | 0 | 0 | 0 | 1 |
| SCN4A | 7 | 0 | 0 | 0 | 0 | 0 | 0 | 0 | 1 |
| BDP1 | 7 | 0 | 0 | 0 | 0 | 0 | 0 | 0 | 1 |
| EP400 | 7 | 0 | 0 | 0 | 0 | 0 | 0 | 0 | 1 |
| RNF213 | 6 | 1 | 0 | 0 | 0 | 0 | 0 | 0 | 1 |
| TECTA | 6 | 1 | 0 | 0 | 0 | 0 | 0 | 0 | 1 |
| PTPN13 | 7 | 0 | 0 | 0 | 0 | 0 | 0 | 0 | 1 |
| ADAMTS16 | 7 | 0 | 0 | 0 | 0 | 0 | 0 | 0 | 1 |
| BSN | 6 | 1 | 0 | 0 | 0 | 0 | 0 | 0 | 1 |
| UBR2 | 6 | 0 | 0 | 0 | 1 | 0 | 0 | 0 | 1 |
| CHD7 | 7 | 0 | 0 | 0 | 0 | 0 | 0 | 0 | 1 |
| KIF26B | 7 | 0 | 0 | 0 | 0 | 0 | 0 | 0 | 1 |
| RGS22 | 6 | 0 | 1 | 0 | 0 | 0 | 0 | 0 | 1 |
| LAMB4 | 7 | 0 | 0 | 0 | 0 | 0 | 0 | 0 | 1 |
| ZFHX3 | 7 | 0 | 0 | 0 | 0 | 0 | 0 | 0 | 1 |
| FCGBP | 7 | 0 | 0 | 0 | 0 | 0 | 0 | 0 | 1 |
| CD163L1 | 6 | 1 | 0 | 0 | 0 | 0 | 0 | 0 | 1 |
| DOPEY2 | 7 | 0 | 0 | 0 | 0 | 0 | 0 | 0 | 1 |
| KIAA1211 | 7 | 0 | 0 | 0 | 0 | 0 | 0 | 0 | 1 |
| MUC5B | 7 | 0 | 0 | 0 | 0 | 0 | 0 | 0 | 1 |
| POLQ | 6 | 1 | 0 | 0 | 0 | 0 | 0 | 0 | 1 |
| NID1 | 7 | 0 | 0 | 0 | 0 | 0 | 0 | 0 | 1 |
| RASGRP2 | 7 | 0 | 0 | 0 | 0 | 0 | 0 | 0 | 1 |
| PAK7 | 6 | 1 | 0 | 0 | 0 | 0 | 0 | 0 | 1 |
| ABCA8 | 7 | 0 | 0 | 0 | 0 | 0 | 0 | 0 | 1 |
| RPTN | 6 | 0 | 0 | 0 | 1 | 0 | 0 | 0 | 1 |
| IPCEF1 | 5 | 1 | 0 | 0 | 0 | 0 | 0 | 0 | 1 |
| GSPT2 | 4 | 2 | 0 | 0 | 0 | 0 | 0 | 0 | 1 |
| CYBB | 4 | 0 | 2 | 0 | 0 | 0 | 0 | 0 | 1 |
| OR5H1 | 4 | 0 | 0 | 0 | 2 | 0 | 0 | 0 | 1 |
| UGT3A2 | 4 | 2 | 0 | 0 | 0 | 0 | 0 | 0 | 1 |
| UGT2A3 | 5 | 1 | 0 | 0 | 0 | 0 | 0 | 0 | 1 |
| THOC5 | 4 | 2 | 0 | 0 | 0 | 0 | 0 | 0 | 1 |

| | | | | | | | | | |
|---|---|---|---|---|---|---|---|---|---|
| CYP7A1 | 5 | 1 | 0 | 0 | 0 | 0 | 0 | 0 | 1 |
| GABRB2 | 5 | 1 | 0 | 0 | 0 | 0 | 0 | 0 | 1 |
| C20orf152 | 5 | 0 | 1 | 0 | 0 | 0 | 0 | 0 | 1 |
| CCDC67 | 5 | 0 | 1 | 0 | 0 | 0 | 0 | 0 | 1 |
| BCAT1 | 4 | 1 | 1 | 0 | 0 | 0 | 0 | 0 | 1 |
| CFHR5 | 5 | 0 | 1 | 0 | 0 | 0 | 0 | 0 | 1 |
| PHF10 | 6 | 0 | 0 | 0 | 0 | 0 | 0 | 0 | 1 |
| NECAB1 | 6 | 0 | 0 | 0 | 0 | 0 | 0 | 0 | 1 |
| SH3KBP1 | 6 | 0 | 0 | 0 | 0 | 0 | 0 | 0 | 1 |
| SLC14A2 | 6 | 0 | 0 | 0 | 0 | 0 | 0 | 0 | 1 |
| MCM10 | 4 | 1 | 0 | 0 | 1 | 0 | 0 | 0 | 1 |
| ZFYVE9 | 4 | 1 | 1 | 0 | 0 | 0 | 0 | 0 | 1 |
| SLC24A2 | 6 | 0 | 0 | 0 | 0 | 0 | 0 | 0 | 1 |
| PPP1R13B | 4 | 2 | 0 | 0 | 0 | 0 | 0 | 0 | 1 |
| ACE2 | 6 | 0 | 0 | 0 | 0 | 0 | 0 | 0 | 1 |
| PAMR1 | 5 | 0 | 0 | 0 | 1 | 0 | 0 | 0 | 1 |
| HRC | 6 | 0 | 0 | 0 | 0 | 0 | 0 | 0 | 1 |
| NPC1L1 | 4 | 2 | 0 | 0 | 0 | 0 | 0 | 0 | 1 |
| SLC13A2 | 6 | 0 | 0 | 0 | 0 | 0 | 0 | 0 | 1 |
| LCP2 | 6 | 0 | 0 | 0 | 0 | 0 | 0 | 0 | 1 |
| KIAA0226 | 5 | 0 | 0 | 0 | 0 | 0 | 1 | 0 | 1 |
| EVI5 | 5 | 0 | 1 | 0 | 0 | 0 | 0 | 0 | 1 |
| HAO1 | 6 | 0 | 0 | 0 | 0 | 0 | 0 | 0 | 1 |
| AMHR2 | 6 | 0 | 0 | 0 | 0 | 0 | 0 | 0 | 1 |
| SLC9A2 | 4 | 2 | 0 | 0 | 0 | 0 | 0 | 0 | 1 |
| TBX15 | 6 | 0 | 0 | 0 | 0 | 0 | 0 | 0 | 1 |
| NOBOX | 5 | 1 | 0 | 0 | 0 | 0 | 0 | 0 | 1 |
| SLC5A5 | 6 | 0 | 0 | 0 | 0 | 0 | 0 | 0 | 1 |
| ACSM4 | 6 | 0 | 0 | 0 | 0 | 0 | 0 | 0 | 1 |
| C10orf27 | 6 | 0 | 0 | 0 | 0 | 0 | 0 | 0 | 1 |
| VNN1 | 6 | 0 | 0 | 0 | 0 | 0 | 0 | 0 | 1 |
| C14orf38 | 6 | 0 | 0 | 0 | 0 | 0 | 0 | 0 | 1 |
| OR4B1 | 6 | 0 | 0 | 0 | 0 | 0 | 0 | 0 | 1 |
| C16orf62 | 6 | 0 | 0 | 0 | 0 | 0 | 0 | 0 | 1 |
| TCTN2 | 6 | 0 | 0 | 0 | 0 | 0 | 0 | 0 | 1 |
| PLG | 5 | 1 | 0 | 0 | 0 | 0 | 0 | 0 | 1 |
| OR1I1 | 6 | 0 | 0 | 0 | 0 | 0 | 0 | 0 | 1 |
| NUP155 | 4 | 2 | 0 | 0 | 0 | 0 | 0 | 0 | 1 |
| KCNB1 | 6 | 0 | 0 | 0 | 0 | 0 | 0 | 0 | 1 |
| ELMO1 | 6 | 0 | 0 | 0 | 0 | 0 | 0 | 0 | 1 |
| CCDC147 | 6 | 0 | 0 | 0 | 0 | 0 | 0 | 0 | 1 |
| LEKR1 | 6 | 0 | 0 | 0 | 0 | 0 | 0 | 0 | 1 |
| PLEKHA4 | 6 | 0 | 0 | 0 | 0 | 0 | 0 | 0 | 1 |
| ZNF534 | 5 | 1 | 0 | 0 | 0 | 0 | 0 | 0 | 1 |
| C9orf84 | 6 | 0 | 0 | 0 | 0 | 0 | 0 | 0 | 1 |
| LEPR | 6 | 0 | 0 | 0 | 0 | 0 | 0 | 0 | 1 |

| | | | | | | | | |
|---|---|---|---|---|---|---|---|---|
| ACLY | 6 | 0 | 0 | 0 | 0 | 0 | 0 | 0 | 1 |
| SPINK5 | 4 | 2 | 0 | 0 | 0 | 0 | 0 | 0 | 1 |
| TTLL2 | 6 | 0 | 0 | 0 | 0 | 0 | 0 | 0 | 1 |
| SSC5D | 4 | 2 | 0 | 0 | 0 | 0 | 0 | 0 | 1 |
| ALDH1L1 | 5 | 1 | 0 | 0 | 0 | 0 | 0 | 0 | 1 |
| MTBP | 6 | 0 | 0 | 0 | 0 | 0 | 0 | 0 | 1 |
| PDE1C | 5 | 0 | 0 | 0 | 1 | 0 | 0 | 0 | 1 |
| DPCR1 | 6 | 0 | 0 | 0 | 0 | 0 | 0 | 0 | 1 |
| PARD3 | 5 | 1 | 0 | 0 | 0 | 0 | 0 | 0 | 1 |
| ANO4 | 4 | 1 | 1 | 0 | 0 | 0 | 0 | 0 | 1 |
| MEGF10 | 5 | 1 | 0 | 0 | 0 | 0 | 0 | 0 | 1 |
| C1orf168 | 6 | 0 | 0 | 0 | 0 | 0 | 0 | 0 | 1 |
| TDRD5 | 6 | 0 | 0 | 0 | 0 | 0 | 0 | 0 | 1 |
| SLCO5A1 | 6 | 0 | 0 | 0 | 0 | 0 | 0 | 0 | 1 |
| ARFGEF2 | 5 | 1 | 0 | 0 | 0 | 0 | 0 | 0 | 1 |
| CPS1 | 5 | 0 | 1 | 0 | 0 | 0 | 0 | 0 | 1 |
| C20orf132 | 5 | 0 | 1 | 0 | 0 | 0 | 0 | 0 | 1 |
| ZCCHC6 | 5 | 1 | 0 | 0 | 0 | 0 | 0 | 0 | 1 |
| C7orf63 | 6 | 0 | 0 | 0 | 0 | 0 | 0 | 0 | 1 |
| MYEF2 | 6 | 0 | 0 | 0 | 0 | 0 | 0 | 0 | 1 |
| TTC18 | 6 | 0 | 0 | 0 | 0 | 0 | 0 | 0 | 1 |
| SNTG2 | 6 | 0 | 0 | 0 | 0 | 0 | 0 | 0 | 1 |
| CNTROB | 6 | 0 | 0 | 0 | 0 | 0 | 0 | 0 | 1 |
| PHKA2 | 6 | 0 | 0 | 0 | 0 | 0 | 0 | 0 | 1 |
| ANPEP | 6 | 0 | 0 | 0 | 0 | 0 | 0 | 0 | 1 |
| C6orf170 | 4 | 1 | 1 | 0 | 0 | 0 | 0 | 0 | 1 |
| ULK4 | 5 | 1 | 0 | 0 | 0 | 0 | 0 | 0 | 1 |
| CCDC88A | 5 | 1 | 0 | 0 | 0 | 0 | 0 | 0 | 1 |
| POLR1B | 5 | 1 | 0 | 0 | 0 | 0 | 0 | 0 | 1 |
| GALNTL6 | 5 | 0 | 0 | 0 | 1 | 0 | 0 | 0 | 1 |
| CPXM2 | 4 | 1 | 0 | 0 | 1 | 0 | 0 | 0 | 1 |
| CCDC39 | 5 | 1 | 0 | 0 | 0 | 0 | 0 | 0 | 1 |
| ZNF667 | 6 | 0 | 0 | 0 | 0 | 0 | 0 | 0 | 1 |
| IQGAP2 | 5 | 0 | 1 | 0 | 0 | 0 | 0 | 0 | 1 |
| KIAA0922 | 4 | 2 | 0 | 0 | 0 | 0 | 0 | 0 | 1 |
| RUNX1T1 | 6 | 0 | 0 | 0 | 0 | 0 | 0 | 0 | 1 |
| C8A | 6 | 0 | 0 | 0 | 0 | 0 | 0 | 0 | 1 |
| NAALAD2 | 6 | 0 | 0 | 0 | 0 | 0 | 0 | 0 | 1 |
| TEK | 6 | 0 | 0 | 0 | 0 | 0 | 0 | 0 | 1 |
| ITGA1 | 5 | 0 | 0 | 0 | 1 | 0 | 0 | 0 | 1 |
| AUTS2 | 4 | 1 | 1 | 0 | 0 | 0 | 0 | 0 | 1 |
| PCDHA12 | 6 | 0 | 0 | 0 | 0 | 0 | 0 | 0 | 1 |
| TXLNB | 6 | 0 | 0 | 0 | 0 | 0 | 0 | 0 | 1 |
| RAB3GAP2 | 6 | 0 | 0 | 0 | 0 | 0 | 0 | 0 | 1 |
| MAP9 | 6 | 0 | 0 | 0 | 0 | 0 | 0 | 0 | 1 |
| SRGAP1 | 6 | 0 | 0 | 0 | 0 | 0 | 0 | 0 | 1 |

| | | | | | | | | | |
|---|---|---|---|---|---|---|---|---|---|
| EFCAB5 | 5 | 1 | 0 | 0 | 0 | 0 | 0 | 0 | 1 |
| PCDHGA12 | 6 | 0 | 0 | 0 | 0 | 0 | 0 | 0 | 1 |
| PCDHB16 | 6 | 0 | 0 | 0 | 0 | 0 | 0 | 0 | 1 |
| TUBA3C | 6 | 0 | 0 | 0 | 0 | 0 | 0 | 0 | 1 |
| AMOTL1 | 6 | 0 | 0 | 0 | 0 | 0 | 0 | 0 | 1 |
| HEATR6 | 6 | 0 | 0 | 0 | 0 | 0 | 0 | 0 | 1 |
| ABCA3 | 6 | 0 | 0 | 0 | 0 | 0 | 0 | 0 | 1 |
| FYB | 5 | 1 | 0 | 0 | 0 | 0 | 0 | 0 | 1 |
| BAHCC1 | 4 | 1 | 1 | 0 | 0 | 0 | 0 | 0 | 1 |
| SLCO1C1 | 6 | 0 | 0 | 0 | 0 | 0 | 0 | 0 | 1 |
| OR2M7 | 6 | 0 | 0 | 0 | 0 | 0 | 0 | 0 | 1 |
| CHSY3 | 5 | 1 | 0 | 0 | 0 | 0 | 0 | 0 | 1 |
| PCDHGB1 | 6 | 0 | 0 | 0 | 0 | 0 | 0 | 0 | 1 |
| UNC13A | 5 | 0 | 1 | 0 | 0 | 0 | 0 | 0 | 1 |
| MMRN1 | 5 | 1 | 0 | 0 | 0 | 0 | 0 | 0 | 1 |
| CCDC33 | 6 | 0 | 0 | 0 | 0 | 0 | 0 | 0 | 1 |
| NLGN4X | 6 | 0 | 0 | 0 | 0 | 0 | 0 | 0 | 1 |
| SORCS3 | 5 | 1 | 0 | 0 | 0 | 0 | 0 | 0 | 1 |
| ANKRD27 | 6 | 0 | 0 | 0 | 0 | 0 | 0 | 0 | 1 |
| SHROOM3 | 6 | 0 | 0 | 0 | 0 | 0 | 0 | 0 | 1 |
| FILIP1L | 6 | 0 | 0 | 0 | 0 | 0 | 0 | 0 | 1 |
| FGA | 6 | 0 | 0 | 0 | 0 | 0 | 0 | 0 | 1 |
| FAM179A | 6 | 0 | 0 | 0 | 0 | 0 | 0 | 0 | 1 |
| ZNF423 | 6 | 0 | 0 | 0 | 0 | 0 | 0 | 0 | 1 |
| NCOA2 | 5 | 0 | 0 | 0 | 1 | 0 | 0 | 0 | 1 |
| ABCC12 | 6 | 0 | 0 | 0 | 0 | 0 | 0 | 0 | 1 |
| CYP2C8 | 6 | 0 | 0 | 0 | 0 | 0 | 0 | 0 | 1 |
| ITGA10 | 5 | 0 | 1 | 0 | 0 | 0 | 0 | 0 | 1 |
| CSPP1 | 5 | 1 | 0 | 0 | 0 | 0 | 0 | 0 | 1 |
| HCN4 | 6 | 0 | 0 | 0 | 0 | 0 | 0 | 0 | 1 |
| KIAA1210 | 6 | 0 | 0 | 0 | 0 | 0 | 0 | 0 | 1 |
| DHX57 | 5 | 1 | 0 | 0 | 0 | 0 | 0 | 0 | 1 |
| KDM5A | 4 | 1 | 1 | 0 | 0 | 0 | 0 | 0 | 1 |
| CAD | 6 | 0 | 0 | 0 | 0 | 0 | 0 | 0 | 1 |
| VWA3B | 6 | 0 | 0 | 0 | 0 | 0 | 0 | 0 | 1 |
| KCNQ3 | 6 | 0 | 0 | 0 | 0 | 0 | 0 | 0 | 1 |
| PDE3A | 6 | 0 | 0 | 0 | 0 | 0 | 0 | 0 | 1 |
| SMG7 | 4 | 2 | 0 | 0 | 0 | 0 | 0 | 0 | 1 |
| MET | 5 | 1 | 0 | 0 | 0 | 0 | 0 | 0 | 1 |
| AKAP12 | 6 | 0 | 0 | 0 | 0 | 0 | 0 | 0 | 1 |
| FLT3 | 6 | 0 | 0 | 0 | 0 | 0 | 0 | 0 | 1 |
| GUCY2C | 6 | 0 | 0 | 0 | 0 | 0 | 0 | 0 | 1 |
| RASGRF1 | 6 | 0 | 0 | 0 | 0 | 0 | 0 | 0 | 1 |
| C3orf77 | 6 | 0 | 0 | 0 | 0 | 0 | 0 | 0 | 1 |
| PTPRS | 5 | 1 | 0 | 0 | 0 | 0 | 0 | 0 | 1 |
| ZEB2 | 6 | 0 | 0 | 0 | 0 | 0 | 0 | 0 | 1 |

| | | | | | | | | |
|---|---|---|---|---|---|---|---|---|
| CRAMP1L | 6 | 0 | 0 | 0 | 0 | 0 | 0 | 0 | 1 |
| SIGLEC1 | 5 | 1 | 0 | 0 | 0 | 0 | 0 | 0 | 1 |
| MYLK | 5 | 1 | 0 | 0 | 0 | 0 | 0 | 0 | 1 |
| NLRP13 | 6 | 0 | 0 | 0 | 0 | 0 | 0 | 0 | 1 |
| ADAMTSL4 | 6 | 0 | 0 | 0 | 0 | 0 | 0 | 0 | 1 |
| RTL1 | 5 | 1 | 0 | 0 | 0 | 0 | 0 | 0 | 1 |
| ENPEP | 5 | 1 | 0 | 0 | 0 | 0 | 0 | 0 | 1 |
| ABCB4 | 6 | 0 | 0 | 0 | 0 | 0 | 0 | 0 | 1 |
| NBEAL1 | 6 | 0 | 0 | 0 | 0 | 0 | 0 | 0 | 1 |
| AKNA | 6 | 0 | 0 | 0 | 0 | 0 | 0 | 0 | 1 |
| KCNA4 | 6 | 0 | 0 | 0 | 0 | 0 | 0 | 0 | 1 |
| ITGA9 | 6 | 0 | 0 | 0 | 0 | 0 | 0 | 0 | 1 |
| FAM196B | 6 | 0 | 0 | 0 | 0 | 0 | 0 | 0 | 1 |
| IL16 | 5 | 1 | 0 | 0 | 0 | 0 | 0 | 0 | 1 |
| CNTN3 | 6 | 0 | 0 | 0 | 0 | 0 | 0 | 0 | 1 |
| GRIK2 | 6 | 0 | 0 | 0 | 0 | 0 | 0 | 0 | 1 |
| AKD1 | 6 | 0 | 0 | 0 | 0 | 0 | 0 | 0 | 1 |
| ATP8A2 | 6 | 0 | 0 | 0 | 0 | 0 | 0 | 0 | 1 |
| POLR2A | 6 | 0 | 0 | 0 | 0 | 0 | 0 | 0 | 1 |
| DNTT | 6 | 0 | 0 | 0 | 0 | 0 | 0 | 0 | 1 |
| E2F7 | 6 | 0 | 0 | 0 | 0 | 0 | 0 | 0 | 1 |
| CDH20 | 5 | 0 | 1 | 0 | 0 | 0 | 0 | 0 | 1 |
| COL21A1 | 6 | 0 | 0 | 0 | 0 | 0 | 0 | 0 | 1 |
| TRPM7 | 5 | 1 | 0 | 0 | 0 | 0 | 0 | 0 | 1 |
| TMEM131 | 6 | 0 | 0 | 0 | 0 | 0 | 0 | 0 | 1 |
| MYO3A | 6 | 0 | 0 | 0 | 0 | 0 | 0 | 0 | 1 |
| TDRD9 | 5 | 1 | 0 | 0 | 0 | 0 | 0 | 0 | 1 |
| ZNF608 | 5 | 1 | 0 | 0 | 0 | 0 | 0 | 0 | 1 |
| HECW2 | 6 | 0 | 0 | 0 | 0 | 0 | 0 | 0 | 1 |
| WDR52 | 5 | 1 | 0 | 0 | 0 | 0 | 0 | 0 | 1 |
| PXDNL | 5 | 0 | 1 | 0 | 0 | 0 | 0 | 0 | 1 |
| ZZEF1 | 5 | 0 | 1 | 0 | 0 | 0 | 0 | 0 | 1 |
| AHCTF1 | 5 | 0 | 1 | 0 | 0 | 0 | 0 | 0 | 1 |
| CDH26 | 4 | 0 | 2 | 0 | 0 | 0 | 0 | 0 | 1 |
| CUX1 | 6 | 0 | 0 | 0 | 0 | 0 | 0 | 0 | 1 |
| MED1 | 6 | 0 | 0 | 0 | 0 | 0 | 0 | 0 | 1 |
| GPR125 | 6 | 0 | 0 | 0 | 0 | 0 | 0 | 0 | 1 |
| MYH10 | 6 | 0 | 0 | 0 | 0 | 0 | 0 | 0 | 1 |
| TCHH | 6 | 0 | 0 | 0 | 0 | 0 | 0 | 0 | 1 |
| SEC31B | 4 | 2 | 0 | 0 | 0 | 0 | 0 | 0 | 1 |
| DZIP3 | 5 | 0 | 1 | 0 | 0 | 0 | 0 | 0 | 1 |
| NLRP5 | 6 | 0 | 0 | 0 | 0 | 0 | 0 | 0 | 1 |
| COL4A5 | 5 | 0 | 0 | 0 | 1 | 0 | 0 | 0 | 1 |
| WNK1 | 5 | 1 | 0 | 0 | 0 | 0 | 0 | 0 | 1 |
| PCDH18 | 6 | 0 | 0 | 0 | 0 | 0 | 0 | 0 | 1 |
| MON2 | 6 | 0 | 0 | 0 | 0 | 0 | 0 | 0 | 1 |

| | | | | | | | | | |
|---|---|---|---|---|---|---|---|---|---|
| RALGAPA2 | 6 | 0 | 0 | 0 | 0 | 0 | 0 | 0 | 1 |
| OR4C3 | 5 | 1 | 0 | 0 | 0 | 0 | 0 | 0 | 1 |
| PHF3 | 6 | 0 | 0 | 0 | 0 | 0 | 0 | 0 | 1 |
| CELSR2 | 6 | 0 | 0 | 0 | 0 | 0 | 0 | 0 | 1 |
| FLT1 | 6 | 0 | 0 | 0 | 0 | 0 | 0 | 0 | 1 |
| HIVEP3 | 6 | 0 | 0 | 0 | 0 | 0 | 0 | 0 | 1 |
| TOPBP1 | 4 | 2 | 0 | 0 | 0 | 0 | 0 | 0 | 1 |
| BRCA1 | 5 | 1 | 0 | 0 | 0 | 0 | 0 | 0 | 1 |
| HUWE1 | 4 | 1 | 0 | 0 | 1 | 0 | 0 | 0 | 1 |
| ZP2 | 6 | 0 | 0 | 0 | 0 | 0 | 0 | 0 | 1 |
| KIAA2018 | 6 | 0 | 0 | 0 | 0 | 0 | 0 | 0 | 1 |
| CDH17 | 6 | 0 | 0 | 0 | 0 | 0 | 0 | 0 | 1 |
| DNAJC13 | 6 | 0 | 0 | 0 | 0 | 0 | 0 | 0 | 1 |
| HPSE2 | 6 | 0 | 0 | 0 | 0 | 0 | 0 | 0 | 1 |
| COL5A3 | 6 | 0 | 0 | 0 | 0 | 0 | 0 | 0 | 1 |
| DYNC1H1 | 5 | 1 | 0 | 0 | 0 | 0 | 0 | 0 | 1 |
| MCF2 | 6 | 0 | 0 | 0 | 0 | 0 | 0 | 0 | 1 |
| USP9X | 6 | 0 | 0 | 0 | 0 | 0 | 0 | 0 | 1 |
| FSIP2 | 4 | 2 | 0 | 0 | 0 | 0 | 0 | 0 | 1 |
| POLA1 | 5 | 1 | 0 | 0 | 0 | 0 | 0 | 0 | 1 |
| PPP4R4 | 6 | 0 | 0 | 0 | 0 | 0 | 0 | 0 | 1 |
| NHS | 5 | 1 | 0 | 0 | 0 | 0 | 0 | 0 | 1 |
| L1CAM | 6 | 0 | 0 | 0 | 0 | 0 | 0 | 0 | 1 |
| NOTCH1 | 3 | 1 | 1 | 0 | 0 | 0 | 0 | 1 | 1 |
| ZMYM4 | 5 | 0 | 1 | 0 | 0 | 0 | 0 | 0 | 1 |
| SYCP1 | 5 | 1 | 0 | 0 | 0 | 0 | 0 | 0 | 1 |
| ASXL3 | 5 | 1 | 0 | 0 | 0 | 0 | 0 | 0 | 1 |
| DGKI | 5 | 1 | 0 | 0 | 0 | 0 | 0 | 0 | 1 |
| RNF216 | 6 | 0 | 0 | 0 | 0 | 0 | 0 | 0 | 1 |
| ALK | 6 | 0 | 0 | 0 | 0 | 0 | 0 | 0 | 1 |
| ADAMTS19 | 6 | 0 | 0 | 0 | 0 | 0 | 0 | 0 | 1 |
| STON1-GTF2A1L | 6 | 0 | 0 | 0 | 0 | 0 | 0 | 0 | 1 |
| SPEG | 6 | 0 | 0 | 0 | 0 | 0 | 0 | 0 | 1 |
| SLC44A5 | 6 | 0 | 0 | 0 | 0 | 0 | 0 | 0 | 1 |
| ADCY10 | 5 | 1 | 0 | 0 | 0 | 0 | 0 | 0 | 1 |
| GRM1 | 6 | 0 | 0 | 0 | 0 | 0 | 0 | 0 | 1 |
| AKAP3 | 5 | 1 | 0 | 0 | 0 | 0 | 0 | 0 | 1 |
| GTF3C1 | 5 | 1 | 0 | 0 | 0 | 0 | 0 | 0 | 1 |
| PRDM15 | 6 | 0 | 0 | 0 | 0 | 0 | 0 | 0 | 1 |
| ARID1B | 6 | 0 | 0 | 0 | 0 | 0 | 0 | 0 | 1 |
| THSD7B | 5 | 0 | 1 | 0 | 0 | 0 | 0 | 0 | 1 |
| SEMG1 | 5 | 0 | 0 | 0 | 1 | 0 | 0 | 0 | 1 |
| VWF | 6 | 0 | 0 | 0 | 0 | 0 | 0 | 0 | 1 |
| CHD8 | 6 | 0 | 0 | 0 | 0 | 0 | 0 | 0 | 1 |
| C11orf9 | 5 | 0 | 0 | 0 | 0 | 1 | 0 | 0 | 1 |
| RPRD2 | 6 | 0 | 0 | 0 | 0 | 0 | 0 | 0 | 1 |

| | | | | | | | | |
|---|---|---|---|---|---|---|---|---|
| NAV1 | 6 | 0 | 0 | 0 | 0 | 0 | 0 | 0 | 1 |
| RALGAPA1 | 6 | 0 | 0 | 0 | 0 | 0 | 0 | 0 | 1 |
| BIRC6 | 5 | 0 | 0 | 1 | 0 | 0 | 0 | 0 | 1 |
| ZFYVE26 | 6 | 0 | 0 | 0 | 0 | 0 | 0 | 0 | 1 |
| PLCB1 | 6 | 0 | 0 | 0 | 0 | 0 | 0 | 0 | 1 |
| LCE1F | 5 | 0 | 0 | 0 | 0 | 0 | 0 | 0 | 1 |
| GSDMC | 2 | 1 | 0 | 0 | 1 | 1 | 0 | 0 | 1 |
| ZNF182 | 4 | 1 | 0 | 0 | 0 | 0 | 0 | 0 | 1 |
| STEAP4 | 4 | 1 | 0 | 0 | 0 | 0 | 0 | 0 | 1 |
| APOA4 | 4 | 1 | 0 | 0 | 0 | 0 | 0 | 0 | 1 |
| MTF2 | 4 | 0 | 1 | 0 | 0 | 0 | 0 | 0 | 1 |
| CASQ2 | 4 | 1 | 0 | 0 | 0 | 0 | 0 | 0 | 1 |
| LRRC18 | 5 | 0 | 0 | 0 | 0 | 0 | 0 | 0 | 1 |
| UGT2B7 | 3 | 2 | 0 | 0 | 0 | 0 | 0 | 0 | 1 |
| PARL | 5 | 0 | 0 | 0 | 0 | 0 | 0 | 0 | 1 |
| GCM1 | 4 | 1 | 0 | 0 | 0 | 0 | 0 | 0 | 1 |
| CASQ1 | 4 | 1 | 0 | 0 | 0 | 0 | 0 | 0 | 1 |
| DTHD1 | 3 | 1 | 1 | 0 | 0 | 0 | 0 | 0 | 1 |
| GJA5 | 5 | 0 | 0 | 0 | 0 | 0 | 0 | 0 | 1 |
| ACTR10 | 5 | 0 | 0 | 0 | 0 | 0 | 0 | 0 | 1 |
| TMEM108 | 4 | 1 | 0 | 0 | 0 | 0 | 0 | 0 | 1 |
| HNRNPH1 | 5 | 0 | 0 | 0 | 0 | 0 | 0 | 0 | 1 |
| CPNE4 | 3 | 2 | 0 | 0 | 0 | 0 | 0 | 0 | 1 |
| GALR1 | 5 | 0 | 0 | 0 | 0 | 0 | 0 | 0 | 1 |
| ANGPTL1 | 4 | 1 | 0 | 0 | 0 | 0 | 0 | 0 | 1 |
| AMPD1 | 4 | 1 | 0 | 0 | 0 | 0 | 0 | 0 | 1 |
| NVL | 4 | 0 | 1 | 0 | 0 | 0 | 0 | 0 | 1 |
| APLP1 | 4 | 0 | 1 | 0 | 0 | 0 | 0 | 0 | 1 |
| RASGRP3 | 4 | 1 | 0 | 0 | 0 | 0 | 0 | 0 | 1 |
| CHIA | 4 | 1 | 0 | 0 | 0 | 0 | 0 | 0 | 1 |
| GDA | 4 | 0 | 1 | 0 | 0 | 0 | 0 | 0 | 1 |
| PGLYRP4 | 5 | 0 | 0 | 0 | 0 | 0 | 0 | 0 | 1 |
| SYT4 | 5 | 0 | 0 | 0 | 0 | 0 | 0 | 0 | 1 |
| AP1B1 | 4 | 1 | 0 | 0 | 0 | 0 | 0 | 0 | 1 |
| OR6N1 | 5 | 0 | 0 | 0 | 0 | 0 | 0 | 0 | 1 |
| OR4K1 | 5 | 0 | 0 | 0 | 0 | 0 | 0 | 0 | 1 |
| OSBPL9 | 4 | 1 | 0 | 0 | 0 | 0 | 0 | 0 | 1 |
| APBA3 | 3 | 1 | 1 | 0 | 0 | 0 | 0 | 0 | 1 |
| ST6GALNAC1 | 4 | 1 | 0 | 0 | 0 | 0 | 0 | 0 | 1 |
| PLCXD3 | 4 | 0 | 1 | 0 | 0 | 0 | 0 | 0 | 1 |
| PVRL3 | 4 | 0 | 0 | 0 | 0 | 1 | 0 | 0 | 1 |
| ATP1A1 | 4 | 1 | 0 | 0 | 0 | 0 | 0 | 0 | 1 |
| NEUROD6 | 4 | 1 | 0 | 0 | 0 | 0 | 0 | 0 | 1 |
| MCM6 | 4 | 1 | 0 | 0 | 0 | 0 | 0 | 0 | 1 |
| FAM194A | 5 | 0 | 0 | 0 | 0 | 0 | 0 | 0 | 1 |
| FAM154A | 5 | 0 | 0 | 0 | 0 | 0 | 0 | 0 | 1 |

| | | | | | | | | | |
|---|---|---|---|---|---|---|---|---|---|
| CUL2 | 5 | 0 | 0 | 0 | 0 | 0 | 0 | 0 | 1 |
| ZNF844 | 4 | 1 | 0 | 0 | 0 | 0 | 0 | 0 | 1 |
| EDIL3 | 4 | 1 | 0 | 0 | 0 | 0 | 0 | 0 | 1 |
| HEPACAM2 | 4 | 0 | 1 | 0 | 0 | 0 | 0 | 0 | 1 |
| TMC3 | 3 | 2 | 0 | 0 | 0 | 0 | 0 | 0 | 1 |
| FAM198B | 4 | 1 | 0 | 0 | 0 | 0 | 0 | 0 | 1 |
| LMX1A | 4 | 0 | 1 | 0 | 0 | 0 | 0 | 0 | 1 |
| AURKC | 5 | 0 | 0 | 0 | 0 | 0 | 0 | 0 | 1 |
| TMPRSS11A | 5 | 0 | 0 | 0 | 0 | 0 | 0 | 0 | 1 |
| COLEC12 | 4 | 1 | 0 | 0 | 0 | 0 | 0 | 0 | 1 |
| TECPR2 | 3 | 1 | 1 | 0 | 0 | 0 | 0 | 0 | 1 |
| GP6 | 4 | 1 | 0 | 0 | 0 | 0 | 0 | 0 | 1 |
| ING1 | 5 | 0 | 0 | 0 | 0 | 0 | 0 | 0 | 1 |
| C17orf47 | 5 | 0 | 0 | 0 | 0 | 0 | 0 | 0 | 1 |
| MMP3 | 5 | 0 | 0 | 0 | 0 | 0 | 0 | 0 | 1 |
| VN1R2 | 5 | 0 | 0 | 0 | 0 | 0 | 0 | 0 | 1 |
| EXOC2 | 4 | 1 | 0 | 0 | 0 | 0 | 0 | 0 | 1 |
| SLC5A7 | 4 | 0 | 0 | 0 | 1 | 0 | 0 | 0 | 1 |
| ZNF84 | 4 | 1 | 0 | 0 | 0 | 0 | 0 | 0 | 1 |
| MX2 | 5 | 0 | 0 | 0 | 0 | 0 | 0 | 0 | 1 |
| NAA25 | 4 | 0 | 1 | 0 | 0 | 0 | 0 | 0 | 1 |
| ZER1 | 5 | 0 | 0 | 0 | 0 | 0 | 0 | 0 | 1 |
| DPPA2 | 5 | 0 | 0 | 0 | 0 | 0 | 0 | 0 | 1 |
| KCNJ3 | 5 | 0 | 0 | 0 | 0 | 0 | 0 | 0 | 1 |
| ZNF595 | 4 | 0 | 0 | 0 | 1 | 0 | 0 | 0 | 1 |
| RNF8 | 5 | 0 | 0 | 0 | 0 | 0 | 0 | 0 | 1 |
| ZNF607 | 4 | 1 | 0 | 0 | 0 | 0 | 0 | 0 | 1 |
| ADAD1 | 5 | 0 | 0 | 0 | 0 | 0 | 0 | 0 | 1 |
| ZNF681 | 4 | 1 | 0 | 0 | 0 | 0 | 0 | 0 | 1 |
| OR5H2 | 5 | 0 | 0 | 0 | 0 | 0 | 0 | 0 | 1 |
| PABPC5 | 4 | 1 | 0 | 0 | 0 | 0 | 0 | 0 | 1 |
| FGB | 5 | 0 | 0 | 0 | 0 | 0 | 0 | 0 | 1 |
| LILRB5 | 5 | 0 | 0 | 0 | 0 | 0 | 0 | 0 | 1 |
| TXK | 5 | 0 | 0 | 0 | 0 | 0 | 0 | 0 | 1 |
| CXorf59 | 4 | 1 | 0 | 0 | 0 | 0 | 0 | 0 | 1 |
| CCNA1 | 5 | 0 | 0 | 0 | 0 | 0 | 0 | 0 | 1 |
| GPR162 | 5 | 0 | 0 | 0 | 0 | 0 | 0 | 0 | 1 |
| SLC2A2 | 5 | 0 | 0 | 0 | 0 | 0 | 0 | 0 | 1 |
| KCNA6 | 5 | 0 | 0 | 0 | 0 | 0 | 0 | 0 | 1 |
| IQGAP3 | 3 | 1 | 1 | 0 | 0 | 0 | 0 | 0 | 1 |
| GPR128 | 3 | 1 | 1 | 0 | 0 | 0 | 0 | 0 | 1 |
| TAB3 | 4 | 1 | 0 | 0 | 0 | 0 | 0 | 0 | 1 |
| SLC22A24 | 5 | 0 | 0 | 0 | 0 | 0 | 0 | 0 | 1 |
| CALN1 | 5 | 0 | 0 | 0 | 0 | 0 | 0 | 0 | 1 |
| CXorf23 | 5 | 0 | 0 | 0 | 0 | 0 | 0 | 0 | 1 |
| HPN | 5 | 0 | 0 | 0 | 0 | 0 | 0 | 0 | 1 |

| | | | | | | | | | |
|---|---|---|---|---|---|---|---|---|---|
| NOD1 | 4 | 1 | 0 | 0 | 0 | 0 | 0 | 0 | 1 |
| IL1RAPL1 | 5 | 0 | 0 | 0 | 0 | 0 | 0 | 0 | 1 |
| KLHL20 | 5 | 0 | 0 | 0 | 0 | 0 | 0 | 0 | 1 |
| TMPRSS2 | 5 | 0 | 0 | 0 | 0 | 0 | 0 | 0 | 1 |
| FIG4 | 3 | 2 | 0 | 0 | 0 | 0 | 0 | 0 | 1 |
| GK | 5 | 0 | 0 | 0 | 0 | 0 | 0 | 0 | 1 |
| OR5AN1 | 4 | 0 | 0 | 0 | 1 | 0 | 0 | 0 | 1 |
| PCDHGA4 | 4 | 1 | 0 | 0 | 0 | 0 | 0 | 0 | 1 |
| SLC6A16 | 5 | 0 | 0 | 0 | 0 | 0 | 0 | 0 | 1 |
| LRRTM4 | 4 | 1 | 0 | 0 | 0 | 0 | 0 | 0 | 1 |
| TPH2 | 5 | 0 | 0 | 0 | 0 | 0 | 0 | 0 | 1 |
| FMN1 | 3 | 2 | 0 | 0 | 0 | 0 | 0 | 0 | 1 |
| KIAA0664 | 5 | 0 | 0 | 0 | 0 | 0 | 0 | 0 | 1 |
| PLEKHG5 | 4 | 0 | 1 | 0 | 0 | 0 | 0 | 0 | 1 |
| ERG | 5 | 0 | 0 | 0 | 0 | 0 | 0 | 0 | 1 |
| PER2 | 3 | 1 | 1 | 0 | 0 | 0 | 0 | 0 | 1 |
| ADAM2 | 4 | 1 | 0 | 0 | 0 | 0 | 0 | 0 | 1 |
| ARHGAP20 | 4 | 0 | 1 | 0 | 0 | 0 | 0 | 0 | 1 |
| CDH15 | 4 | 1 | 0 | 0 | 0 | 0 | 0 | 0 | 1 |
| SALL4 | 4 | 1 | 0 | 0 | 0 | 0 | 0 | 0 | 1 |
| OR5T1 | 5 | 0 | 0 | 0 | 0 | 0 | 0 | 0 | 1 |
| RRP15 | 5 | 0 | 0 | 0 | 0 | 0 | 0 | 0 | 1 |
| MPO | 5 | 0 | 0 | 0 | 0 | 0 | 0 | 0 | 1 |
| SYCP2L | 4 | 0 | 0 | 0 | 1 | 0 | 0 | 0 | 1 |
| TXNDC3 | 5 | 0 | 0 | 0 | 0 | 0 | 0 | 0 | 1 |
| ETAA1 | 3 | 2 | 0 | 0 | 0 | 0 | 0 | 0 | 1 |
| VEGFC | 5 | 0 | 0 | 0 | 0 | 0 | 0 | 0 | 1 |
| FOXK2 | 5 | 0 | 0 | 0 | 0 | 0 | 0 | 0 | 1 |
| FRMD6 | 5 | 0 | 0 | 0 | 0 | 0 | 0 | 0 | 1 |
| PYHIN1 | 5 | 0 | 0 | 0 | 0 | 0 | 0 | 0 | 1 |
| WDR49 | 4 | 1 | 0 | 0 | 0 | 0 | 0 | 0 | 1 |
| ATP8B2 | 4 | 1 | 0 | 0 | 0 | 0 | 0 | 0 | 1 |
| AGGF1 | 5 | 0 | 0 | 0 | 0 | 0 | 0 | 0 | 1 |
| NPHP3 | 4 | 1 | 0 | 0 | 0 | 0 | 0 | 0 | 1 |
| GDPD4 | 5 | 0 | 0 | 0 | 0 | 0 | 0 | 0 | 1 |
| FSCB | 4 | 1 | 0 | 0 | 0 | 0 | 0 | 0 | 1 |
| HAL | 5 | 0 | 0 | 0 | 0 | 0 | 0 | 0 | 1 |
| OLFM4 | 5 | 0 | 0 | 0 | 0 | 0 | 0 | 0 | 1 |
| GCLC | 3 | 2 | 0 | 0 | 0 | 0 | 0 | 0 | 1 |
| DAB2 | 5 | 0 | 0 | 0 | 0 | 0 | 0 | 0 | 1 |
| TMPRSS9 | 5 | 0 | 0 | 0 | 0 | 0 | 0 | 0 | 1 |
| PFKM | 5 | 0 | 0 | 0 | 0 | 0 | 0 | 0 | 1 |
| OTUD7B | 5 | 0 | 0 | 0 | 0 | 0 | 0 | 0 | 1 |
| C10orf90 | 5 | 0 | 0 | 0 | 0 | 0 | 0 | 0 | 1 |
| ITK | 5 | 0 | 0 | 0 | 0 | 0 | 0 | 0 | 1 |
| LRCH3 | 4 | 0 | 0 | 0 | 1 | 0 | 0 | 0 | 1 |

| | | | | | | | | | |
|---|---|---|---|---|---|---|---|---|---|
| EPS8L3 | 5 | 0 | 0 | 0 | 0 | 0 | 0 | 0 | 1 |
| POU6F2 | 4 | 1 | 0 | 0 | 0 | 0 | 0 | 0 | 1 |
| CCDC158 | 5 | 0 | 0 | 0 | 0 | 0 | 0 | 0 | 1 |
| DHX34 | 4 | 1 | 0 | 0 | 0 | 0 | 0 | 0 | 1 |
| TAOK3 | 5 | 0 | 0 | 0 | 0 | 0 | 0 | 0 | 1 |
| CPB1 | 5 | 0 | 0 | 0 | 0 | 0 | 0 | 0 | 1 |
| OLFM3 | 5 | 0 | 0 | 0 | 0 | 0 | 0 | 0 | 1 |
| PRKCQ | 5 | 0 | 0 | 0 | 0 | 0 | 0 | 0 | 1 |
| NEK5 | 5 | 0 | 0 | 0 | 0 | 0 | 0 | 0 | 1 |
| AMPH | 5 | 0 | 0 | 0 | 0 | 0 | 0 | 0 | 1 |
| ZNF568 | 5 | 0 | 0 | 0 | 0 | 0 | 0 | 0 | 1 |
| SCUBE3 | 5 | 0 | 0 | 0 | 0 | 0 | 0 | 0 | 1 |
| PRKG2 | 5 | 0 | 0 | 0 | 0 | 0 | 0 | 0 | 1 |
| XIRP1 | 3 | 2 | 0 | 0 | 0 | 0 | 0 | 0 | 1 |
| PMFBP1 | 5 | 0 | 0 | 0 | 0 | 0 | 0 | 0 | 1 |
| KEL | 4 | 0 | 1 | 0 | 0 | 0 | 0 | 0 | 1 |
| CRISPLD1 | 5 | 0 | 0 | 0 | 0 | 0 | 0 | 0 | 1 |
| AK5 | 4 | 1 | 0 | 0 | 0 | 0 | 0 | 0 | 1 |
| SIGLEC7 | 5 | 0 | 0 | 0 | 0 | 0 | 0 | 0 | 1 |
| WDHD1 | 4 | 1 | 0 | 0 | 0 | 0 | 0 | 0 | 1 |
| PCDHB7 | 5 | 0 | 0 | 0 | 0 | 0 | 0 | 0 | 1 |
| MKL2 | 3 | 2 | 0 | 0 | 0 | 0 | 0 | 0 | 1 |
| DENND5A | 3 | 2 | 0 | 0 | 0 | 0 | 0 | 0 | 1 |
| COL25A1 | 5 | 0 | 0 | 0 | 0 | 0 | 0 | 0 | 1 |
| EPS8 | 5 | 0 | 0 | 0 | 0 | 0 | 0 | 0 | 1 |
| MYO1E | 5 | 0 | 0 | 0 | 0 | 0 | 0 | 0 | 1 |
| ITCH | 4 | 1 | 0 | 0 | 0 | 0 | 0 | 0 | 1 |
| PRG4 | 4 | 1 | 0 | 0 | 0 | 0 | 0 | 0 | 1 |
| L3MBTL3 | 4 | 1 | 0 | 0 | 0 | 0 | 0 | 0 | 1 |
| ROR2 | 4 | 1 | 0 | 0 | 0 | 0 | 0 | 0 | 1 |
| ZNF780A | 5 | 0 | 0 | 0 | 0 | 0 | 0 | 0 | 1 |
| PHKA1 | 4 | 1 | 0 | 0 | 0 | 0 | 0 | 0 | 1 |
| NUP210L | 4 | 1 | 0 | 0 | 0 | 0 | 0 | 0 | 1 |
| C17orf57 | 4 | 1 | 0 | 0 | 0 | 0 | 0 | 0 | 1 |
| ACSS2 | 5 | 0 | 0 | 0 | 0 | 0 | 0 | 0 | 1 |
| CAMTA2 | 5 | 0 | 0 | 0 | 0 | 0 | 0 | 0 | 1 |
| CASK | 5 | 0 | 0 | 0 | 0 | 0 | 0 | 0 | 1 |
| GPC5 | 5 | 0 | 0 | 0 | 0 | 0 | 0 | 0 | 1 |
| CYLC1 | 5 | 0 | 0 | 0 | 0 | 0 | 0 | 0 | 1 |
| IFT80 | 5 | 0 | 0 | 0 | 0 | 0 | 0 | 0 | 1 |
| ZSCAN20 | 4 | 1 | 0 | 0 | 0 | 0 | 0 | 0 | 1 |
| VPS8 | 3 | 1 | 1 | 0 | 0 | 0 | 0 | 0 | 1 |
| GAPVD1 | 4 | 0 | 0 | 0 | 1 | 0 | 0 | 0 | 1 |
| PDS5A | 4 | 1 | 0 | 0 | 0 | 0 | 0 | 0 | 1 |
| KIAA1468 | 4 | 0 | 1 | 0 | 0 | 0 | 0 | 0 | 1 |
| PNPLA7 | 4 | 1 | 0 | 0 | 0 | 0 | 0 | 0 | 1 |

| | | | | | | | | | |
|---|---|---|---|---|---|---|---|---|---|
| DEPDC1 | 5 | 0 | 0 | 0 | 0 | 0 | 0 | 0 | 1 |
| MED12L | 3 | 1 | 1 | 0 | 0 | 0 | 0 | 0 | 1 |
| FSHR | 5 | 0 | 0 | 0 | 0 | 0 | 0 | 0 | 1 |
| COPB2 | 4 | 1 | 0 | 0 | 0 | 0 | 0 | 0 | 1 |
| DPYD | 3 | 0 | 2 | 0 | 0 | 0 | 0 | 0 | 1 |
| CPNE8 | 5 | 0 | 0 | 0 | 0 | 0 | 0 | 0 | 1 |
| MAST3 | 4 | 1 | 0 | 0 | 0 | 0 | 0 | 0 | 1 |
| ITIH2 | 4 | 0 | 1 | 0 | 0 | 0 | 0 | 0 | 1 |
| IGSF9 | 5 | 0 | 0 | 0 | 0 | 0 | 0 | 0 | 1 |
| SEL1L2 | 5 | 0 | 0 | 0 | 0 | 0 | 0 | 0 | 1 |
| SNAP91 | 5 | 0 | 0 | 0 | 0 | 0 | 0 | 0 | 1 |
| PLXNC1 | 4 | 0 | 1 | 0 | 0 | 0 | 0 | 0 | 1 |
| CALCR | 5 | 0 | 0 | 0 | 0 | 0 | 0 | 0 | 1 |
| CLCN1 | 5 | 0 | 0 | 0 | 0 | 0 | 0 | 0 | 1 |
| SOS2 | 3 | 0 | 1 | 0 | 1 | 0 | 0 | 0 | 1 |
| NFX1 | 4 | 1 | 0 | 0 | 0 | 0 | 0 | 0 | 1 |
| SLC4A10 | 4 | 1 | 0 | 0 | 0 | 0 | 0 | 0 | 1 |
| KCNH3 | 5 | 0 | 0 | 0 | 0 | 0 | 0 | 0 | 1 |
| RPGRIP1 | 5 | 0 | 0 | 0 | 0 | 0 | 0 | 0 | 1 |
| RPS6KA2 | 5 | 0 | 0 | 0 | 0 | 0 | 0 | 0 | 1 |
| BMX | 5 | 0 | 0 | 0 | 0 | 0 | 0 | 0 | 1 |
| ZNF23 | 5 | 0 | 0 | 0 | 0 | 0 | 0 | 0 | 1 |
| TAS1R2 | 5 | 0 | 0 | 0 | 0 | 0 | 0 | 0 | 1 |
| PCDHGB5 | 4 | 1 | 0 | 0 | 0 | 0 | 0 | 0 | 1 |
| SNTG1 | 5 | 0 | 0 | 0 | 0 | 0 | 0 | 0 | 1 |
| OR2M3 | 4 | 1 | 0 | 0 | 0 | 0 | 0 | 0 | 1 |
| TIE1 | 5 | 0 | 0 | 0 | 0 | 0 | 0 | 0 | 1 |
| ZFYVE16 | 4 | 0 | 1 | 0 | 0 | 0 | 0 | 0 | 1 |
| PASK | 5 | 0 | 0 | 0 | 0 | 0 | 0 | 0 | 1 |
| RAD51AP2 | 4 | 1 | 0 | 0 | 0 | 0 | 0 | 0 | 1 |
| WDR11 | 5 | 0 | 0 | 0 | 0 | 0 | 0 | 0 | 1 |
| TCHHL1 | 5 | 0 | 0 | 0 | 0 | 0 | 0 | 0 | 1 |
| LRIG3 | 3 | 2 | 0 | 0 | 0 | 0 | 0 | 0 | 1 |
| ABLIM1 | 4 | 0 | 0 | 1 | 0 | 0 | 0 | 0 | 1 |
| TNKS | 4 | 1 | 0 | 0 | 0 | 0 | 0 | 0 | 1 |
| ENPP1 | 5 | 0 | 0 | 0 | 0 | 0 | 0 | 0 | 1 |
| CCDC80 | 5 | 0 | 0 | 0 | 0 | 0 | 0 | 0 | 1 |
| WDR16 | 5 | 0 | 0 | 0 | 0 | 0 | 0 | 0 | 1 |
| FAM65C | 4 | 0 | 1 | 0 | 0 | 0 | 0 | 0 | 1 |
| USP47 | 3 | 2 | 0 | 0 | 0 | 0 | 0 | 0 | 1 |
| PTPRH | 5 | 0 | 0 | 0 | 0 | 0 | 0 | 0 | 1 |
| C8orf80 | 5 | 0 | 0 | 0 | 0 | 0 | 0 | 0 | 1 |
| CCDC40 | 5 | 0 | 0 | 0 | 0 | 0 | 0 | 0 | 1 |
| HPS3 | 5 | 0 | 0 | 0 | 0 | 0 | 0 | 0 | 1 |
| PHF20L1 | 5 | 0 | 0 | 0 | 0 | 0 | 0 | 0 | 1 |
| TIAM2 | 4 | 1 | 0 | 0 | 0 | 0 | 0 | 0 | 1 |

| | | | | | | | | | |
|---|---|---|---|---|---|---|---|---|---|
| FGR | 4 | 1 | 0 | 0 | 0 | 0 | 0 | 0 | 1 |
| LIMCH1 | 5 | 0 | 0 | 0 | 0 | 0 | 0 | 0 | 1 |
| SEC16A | 3 | 2 | 0 | 0 | 0 | 0 | 0 | 0 | 1 |
| MOV10L1 | 4 | 1 | 0 | 0 | 0 | 0 | 0 | 0 | 1 |
| NHSL2 | 4 | 1 | 0 | 0 | 0 | 0 | 0 | 0 | 1 |
| C6orf132 | 5 | 0 | 0 | 0 | 0 | 0 | 0 | 0 | 1 |
| LRRC16A | 4 | 0 | 1 | 0 | 0 | 0 | 0 | 0 | 1 |
| IREB2 | 5 | 0 | 0 | 0 | 0 | 0 | 0 | 0 | 1 |
| RAD50 | 4 | 1 | 0 | 0 | 0 | 0 | 0 | 0 | 1 |
| LNX1 | 4 | 0 | 1 | 0 | 0 | 0 | 0 | 0 | 1 |
| KDM5B | 4 | 1 | 0 | 0 | 0 | 0 | 0 | 0 | 1 |
| OR10G9 | 3 | 0 | 0 | 0 | 2 | 0 | 0 | 0 | 1 |
| LRGUK | 5 | 0 | 0 | 0 | 0 | 0 | 0 | 0 | 1 |
| MERTK | 5 | 0 | 0 | 0 | 0 | 0 | 0 | 0 | 1 |
| NLRP11 | 5 | 0 | 0 | 0 | 0 | 0 | 0 | 0 | 1 |
| ABL1 | 5 | 0 | 0 | 0 | 0 | 0 | 0 | 0 | 1 |
| PTPRO | 5 | 0 | 0 | 0 | 0 | 0 | 0 | 0 | 1 |
| PLEKHH1 | 5 | 0 | 0 | 0 | 0 | 0 | 0 | 0 | 1 |
| C10orf12 | 3 | 2 | 0 | 0 | 0 | 0 | 0 | 0 | 1 |
| GREB1 | 4 | 1 | 0 | 0 | 0 | 0 | 0 | 0 | 1 |
| CCDC30 | 4 | 1 | 0 | 0 | 0 | 0 | 0 | 0 | 1 |
| CD109 | 5 | 0 | 0 | 0 | 0 | 0 | 0 | 0 | 1 |
| OR5W2 | 5 | 0 | 0 | 0 | 0 | 0 | 0 | 0 | 1 |
| GRIA3 | 5 | 0 | 0 | 0 | 0 | 0 | 0 | 0 | 1 |
| C12orf40 | 5 | 0 | 0 | 0 | 0 | 0 | 0 | 0 | 1 |
| STK31 | 5 | 0 | 0 | 0 | 0 | 0 | 0 | 0 | 1 |
| RLF | 5 | 0 | 0 | 0 | 0 | 0 | 0 | 0 | 1 |
| VWA3A | 5 | 0 | 0 | 0 | 0 | 0 | 0 | 0 | 1 |
| PCDHA1 | 5 | 0 | 0 | 0 | 0 | 0 | 0 | 0 | 1 |
| ERBB3 | 4 | 1 | 0 | 0 | 0 | 0 | 0 | 0 | 1 |
| UGGT2 | 4 | 1 | 0 | 0 | 0 | 0 | 0 | 0 | 1 |
| CDC42BPB | 5 | 0 | 0 | 0 | 0 | 0 | 0 | 0 | 1 |
| PAPPA | 3 | 2 | 0 | 0 | 0 | 0 | 0 | 0 | 1 |
| TTC3 | 5 | 0 | 0 | 0 | 0 | 0 | 0 | 0 | 1 |
| SBF2 | 5 | 0 | 0 | 0 | 0 | 0 | 0 | 0 | 1 |
| APLF | 5 | 0 | 0 | 0 | 0 | 0 | 0 | 0 | 1 |
| MED14 | 5 | 0 | 0 | 0 | 0 | 0 | 0 | 0 | 1 |
| PDCD11 | 4 | 1 | 0 | 0 | 0 | 0 | 0 | 0 | 1 |
| PIK3C2A | 5 | 0 | 0 | 0 | 0 | 0 | 0 | 0 | 1 |
| T | 4 | 1 | 0 | 0 | 0 | 0 | 0 | 0 | 1 |
| SYNJ1 | 3 | 1 | 1 | 0 | 0 | 0 | 0 | 0 | 1 |
| KCP | 4 | 1 | 0 | 0 | 0 | 0 | 0 | 0 | 1 |
| NFATC2 | 5 | 0 | 0 | 0 | 0 | 0 | 0 | 0 | 1 |
| DACH2 | 5 | 0 | 0 | 0 | 0 | 0 | 0 | 0 | 1 |
| ZSCAN5B | 5 | 0 | 0 | 0 | 0 | 0 | 0 | 0 | 1 |
| CC2D2A | 4 | 1 | 0 | 0 | 0 | 0 | 0 | 0 | 1 |

| | | | | | | | | |
|---|---|---|---|---|---|---|---|---|
| NLRP8 | 5 | 0 | 0 | 0 | 0 | 0 | 0 | 0 | 1 |
| CILP | 5 | 0 | 0 | 0 | 0 | 0 | 0 | 0 | 1 |
| CDH18 | 5 | 0 | 0 | 0 | 0 | 0 | 0 | 0 | 1 |
| C2orf16 | 5 | 0 | 0 | 0 | 0 | 0 | 0 | 0 | 1 |
| CCDC129 | 5 | 0 | 0 | 0 | 0 | 0 | 0 | 0 | 1 |
| SUPT6H | 5 | 0 | 0 | 0 | 0 | 0 | 0 | 0 | 1 |
| NRK | 4 | 1 | 0 | 0 | 0 | 0 | 0 | 0 | 1 |
| FCAMR | 4 | 1 | 0 | 0 | 0 | 0 | 0 | 0 | 1 |
| ADAMTSL1 | 4 | 1 | 0 | 0 | 0 | 0 | 0 | 0 | 1 |
| ANO5 | 4 | 1 | 0 | 0 | 0 | 0 | 0 | 0 | 1 |
| ITGAL | 5 | 0 | 0 | 0 | 0 | 0 | 0 | 0 | 1 |
| SEMA5B | 5 | 0 | 0 | 0 | 0 | 0 | 0 | 0 | 1 |
| CHD3 | 5 | 0 | 0 | 0 | 0 | 0 | 0 | 0 | 1 |
| ZNF334 | 5 | 0 | 0 | 0 | 0 | 0 | 0 | 0 | 1 |
| OSBPL6 | 3 | 1 | 1 | 0 | 0 | 0 | 0 | 0 | 1 |
| STK11IP | 5 | 0 | 0 | 0 | 0 | 0 | 0 | 0 | 1 |
| IGSF1 | 4 | 0 | 0 | 0 | 1 | 0 | 0 | 0 | 1 |
| SLC13A1 | 4 | 1 | 0 | 0 | 0 | 0 | 0 | 0 | 1 |
| ACVRL1 | 5 | 0 | 0 | 0 | 0 | 0 | 0 | 0 | 1 |
| PKD1L3 | 4 | 1 | 0 | 0 | 0 | 0 | 0 | 0 | 1 |
| KIAA2022 | 5 | 0 | 0 | 0 | 0 | 0 | 0 | 0 | 1 |
| ABCC8 | 3 | 1 | 1 | 0 | 0 | 0 | 0 | 0 | 1 |
| OR5H15 | 5 | 0 | 0 | 0 | 0 | 0 | 0 | 0 | 1 |
| DDX60L | 4 | 1 | 0 | 0 | 0 | 0 | 0 | 0 | 1 |
| ADAMTS3 | 4 | 1 | 0 | 0 | 0 | 0 | 0 | 0 | 1 |
| SETBP1 | 4 | 0 | 0 | 1 | 0 | 0 | 0 | 0 | 1 |
| NID2 | 5 | 0 | 0 | 0 | 0 | 0 | 0 | 0 | 1 |
| ATP10D | 5 | 0 | 0 | 0 | 0 | 0 | 0 | 0 | 1 |
| ABCC3 | 5 | 0 | 0 | 0 | 0 | 0 | 0 | 0 | 1 |
| SH2D3C | 5 | 0 | 0 | 0 | 0 | 0 | 0 | 0 | 1 |
| INPP5D | 5 | 0 | 0 | 0 | 0 | 0 | 0 | 0 | 1 |
| LRRC66 | 5 | 0 | 0 | 0 | 0 | 0 | 0 | 0 | 1 |
| PCDHA2 | 5 | 0 | 0 | 0 | 0 | 0 | 0 | 0 | 1 |
| NPR1 | 5 | 0 | 0 | 0 | 0 | 0 | 0 | 0 | 1 |
| RXFP1 | 5 | 0 | 0 | 0 | 0 | 0 | 0 | 0 | 1 |
| ADCY8 | 5 | 0 | 0 | 0 | 0 | 0 | 0 | 0 | 1 |
| ITSN1 | 5 | 0 | 0 | 0 | 0 | 0 | 0 | 0 | 1 |
| TSC2 | 5 | 0 | 0 | 0 | 0 | 0 | 0 | 0 | 1 |
| ABCB5 | 4 | 1 | 0 | 0 | 0 | 0 | 0 | 0 | 1 |
| MYH3 | 5 | 0 | 0 | 0 | 0 | 0 | 0 | 0 | 1 |
| KLHL1 | 5 | 0 | 0 | 0 | 0 | 0 | 0 | 0 | 1 |
| FLNA | 5 | 0 | 0 | 0 | 0 | 0 | 0 | 0 | 1 |
| RNF17 | 5 | 0 | 0 | 0 | 0 | 0 | 0 | 0 | 1 |
| SIPA1L1 | 5 | 0 | 0 | 0 | 0 | 0 | 0 | 0 | 1 |
| ZNF646 | 5 | 0 | 0 | 0 | 0 | 0 | 0 | 0 | 1 |
| KDR | 5 | 0 | 0 | 0 | 0 | 0 | 0 | 0 | 1 |

| | | | | | | | | | |
|---|---|---|---|---|---|---|---|---|---|
| FANCM | 5 | 0 | 0 | 0 | 0 | 0 | 0 | 0 | 1 |
| TBC1D8B | 5 | 0 | 0 | 0 | 0 | 0 | 0 | 0 | 1 |
| CDH2 | 4 | 1 | 0 | 0 | 0 | 0 | 0 | 0 | 1 |
| PDE1A | 5 | 0 | 0 | 0 | 0 | 0 | 0 | 0 | 1 |
| AQPEP | 5 | 0 | 0 | 0 | 0 | 0 | 0 | 0 | 1 |
| TMPRSS7 | 5 | 0 | 0 | 0 | 0 | 0 | 0 | 0 | 1 |
| NCKAP5L | 5 | 0 | 0 | 0 | 0 | 0 | 0 | 0 | 1 |
| BMPER | 5 | 0 | 0 | 0 | 0 | 0 | 0 | 0 | 1 |
| PARP15 | 5 | 0 | 0 | 0 | 0 | 0 | 0 | 0 | 1 |
| SLIT1 | 5 | 0 | 0 | 0 | 0 | 0 | 0 | 0 | 1 |
| HDLBP | 5 | 0 | 0 | 0 | 0 | 0 | 0 | 0 | 1 |
| DENND5B | 5 | 0 | 0 | 0 | 0 | 0 | 0 | 0 | 1 |
| CCNB3 | 5 | 0 | 0 | 0 | 0 | 0 | 0 | 0 | 1 |
| IGSF10 | 4 | 1 | 0 | 0 | 0 | 0 | 0 | 0 | 1 |
| CASZ1 | 4 | 1 | 0 | 0 | 0 | 0 | 0 | 0 | 1 |
| LIG4 | 5 | 0 | 0 | 0 | 0 | 0 | 0 | 0 | 1 |
| CASP8AP2 | 5 | 0 | 0 | 0 | 0 | 0 | 0 | 0 | 1 |
| MAN2A1 | 3 | 2 | 0 | 0 | 0 | 0 | 0 | 0 | 1 |
| TSGA10 | 5 | 0 | 0 | 0 | 0 | 0 | 0 | 0 | 1 |
| DICER1 | 5 | 0 | 0 | 0 | 0 | 0 | 0 | 0 | 1 |
| KIF18A | 5 | 0 | 0 | 0 | 0 | 0 | 0 | 0 | 1 |
| DOCK9 | 3 | 0 | 2 | 0 | 0 | 0 | 0 | 0 | 1 |
| DLGAP2 | 4 | 1 | 0 | 0 | 0 | 0 | 0 | 0 | 1 |
| SLC4A5 | 5 | 0 | 0 | 0 | 0 | 0 | 0 | 0 | 1 |
| QRICH2 | 5 | 0 | 0 | 0 | 0 | 0 | 0 | 0 | 1 |
| ADAMTS13 | 4 | 0 | 0 | 0 | 0 | 1 | 0 | 0 | 1 |
| FSD2 | 5 | 0 | 0 | 0 | 0 | 0 | 0 | 0 | 1 |
| KCNH7 | 5 | 0 | 0 | 0 | 0 | 0 | 0 | 0 | 1 |
| PKDREJ | 5 | 0 | 0 | 0 | 0 | 0 | 0 | 0 | 1 |
| ADAM19 | 5 | 0 | 0 | 0 | 0 | 0 | 0 | 0 | 1 |
| SULF2 | 3 | 0 | 2 | 0 | 0 | 0 | 0 | 0 | 1 |
| ABCC11 | 4 | 0 | 1 | 0 | 0 | 0 | 0 | 0 | 1 |
| CECR2 | 5 | 0 | 0 | 0 | 0 | 0 | 0 | 0 | 1 |
| ATR | 3 | 0 | 0 | 0 | 2 | 0 | 0 | 0 | 1 |
| HHLA1 | 5 | 0 | 0 | 0 | 0 | 0 | 0 | 0 | 1 |
| USP36 | 4 | 1 | 0 | 0 | 0 | 0 | 0 | 0 | 1 |
| ZNF318 | 5 | 0 | 0 | 0 | 0 | 0 | 0 | 0 | 1 |
| HELZ | 3 | 2 | 0 | 0 | 0 | 0 | 0 | 0 | 1 |
| MGA | 4 | 1 | 0 | 0 | 0 | 0 | 0 | 0 | 1 |
| CHD2 | 5 | 0 | 0 | 0 | 0 | 0 | 0 | 0 | 1 |
| DNMT1 | 5 | 0 | 0 | 0 | 0 | 0 | 0 | 0 | 1 |
| PCDH9 | 5 | 0 | 0 | 0 | 0 | 0 | 0 | 0 | 1 |
| SYT10 | 5 | 0 | 0 | 0 | 0 | 0 | 0 | 0 | 1 |
| KIAA1671 | 5 | 0 | 0 | 0 | 0 | 0 | 0 | 0 | 1 |
| A2ML1 | 5 | 0 | 0 | 0 | 0 | 0 | 0 | 0 | 1 |
| OCRL | 3 | 2 | 0 | 0 | 0 | 0 | 0 | 0 | 1 |

| | | | | | | | | | |
|---|---|---|---|---|---|---|---|---|---|
| SMCHD1 | 4 | 1 | 0 | 0 | 0 | 0 | 0 | 0 | 1 |
| SEC14L5 | 5 | 0 | 0 | 0 | 0 | 0 | 0 | 0 | 1 |
| GALNT13 | 4 | 0 | 1 | 0 | 0 | 0 | 0 | 0 | 1 |
| OR10K1 | 4 | 0 | 0 | 0 | 1 | 0 | 0 | 0 | 1 |
| KIAA0430 | 5 | 0 | 0 | 0 | 0 | 0 | 0 | 0 | 1 |
| C1orf9 | 5 | 0 | 0 | 0 | 0 | 0 | 0 | 0 | 1 |
| CACNA1D | 3 | 1 | 0 | 0 | 1 | 0 | 0 | 0 | 1 |
| MUC6 | 5 | 0 | 0 | 0 | 0 | 0 | 0 | 0 | 1 |
| ZBTB40 | 3 | 0 | 0 | 0 | 2 | 0 | 0 | 0 | 1 |
| CELSR3 | 4 | 0 | 1 | 0 | 0 | 0 | 0 | 0 | 1 |
| ZFPM2 | 4 | 0 | 1 | 0 | 0 | 0 | 0 | 0 | 1 |
| CDH11 | 5 | 0 | 0 | 0 | 0 | 0 | 0 | 0 | 1 |
| FAM83B | 5 | 0 | 0 | 0 | 0 | 0 | 0 | 0 | 1 |
| LBP | 5 | 0 | 0 | 0 | 0 | 0 | 0 | 0 | 1 |
| SEZ6L | 5 | 0 | 0 | 0 | 0 | 0 | 0 | 0 | 1 |
| SYNPO2 | 5 | 0 | 0 | 0 | 0 | 0 | 0 | 0 | 1 |
| CAND1 | 5 | 0 | 0 | 0 | 0 | 0 | 0 | 0 | 1 |
| TMC2 | 5 | 0 | 0 | 0 | 0 | 0 | 0 | 0 | 1 |
| NUP214 | 5 | 0 | 0 | 0 | 0 | 0 | 0 | 0 | 1 |
| KIAA0754 | 5 | 0 | 0 | 0 | 0 | 0 | 0 | 0 | 1 |
| CLVS1 | 4 | 1 | 0 | 0 | 0 | 0 | 0 | 0 | 1 |
| ZNF638 | 5 | 0 | 0 | 0 | 0 | 0 | 0 | 0 | 1 |
| UMODL1 | 5 | 0 | 0 | 0 | 0 | 0 | 0 | 0 | 1 |
| FLT4 | 5 | 0 | 0 | 0 | 0 | 0 | 0 | 0 | 1 |
| C14orf102 | 5 | 0 | 0 | 0 | 0 | 0 | 0 | 0 | 1 |
| NAALADL1 | 5 | 0 | 0 | 0 | 0 | 0 | 0 | 0 | 1 |
| ARHGAP32 | 5 | 0 | 0 | 0 | 0 | 0 | 0 | 0 | 1 |
| SLITRK5 | 5 | 0 | 0 | 0 | 0 | 0 | 0 | 0 | 1 |
| GIMAP8 | 5 | 0 | 0 | 0 | 0 | 0 | 0 | 0 | 1 |
| LAMA5 | 4 | 1 | 0 | 0 | 0 | 0 | 0 | 0 | 1 |
| CYP1A2 | 5 | 0 | 0 | 0 | 0 | 0 | 0 | 0 | 1 |
| FAM135A | 5 | 0 | 0 | 0 | 0 | 0 | 0 | 0 | 1 |
| AKAP6 | 5 | 0 | 0 | 0 | 0 | 0 | 0 | 0 | 1 |
| SLC6A15 | 5 | 0 | 0 | 0 | 0 | 0 | 0 | 0 | 1 |
| GPRC6A | 4 | 1 | 0 | 0 | 0 | 0 | 0 | 0 | 1 |
| SCEL | 3 | 0 | 2 | 0 | 0 | 0 | 0 | 0 | 1 |
| COL16A1 | 5 | 0 | 0 | 0 | 0 | 0 | 0 | 0 | 1 |
| PER1 | 5 | 0 | 0 | 0 | 0 | 0 | 0 | 0 | 1 |
| SORBS1 | 5 | 0 | 0 | 0 | 0 | 0 | 0 | 0 | 1 |
| HEATR5A | 5 | 0 | 0 | 0 | 0 | 0 | 0 | 0 | 1 |
| KCNB2 | 5 | 0 | 0 | 0 | 0 | 0 | 0 | 0 | 1 |
| MYO5C | 5 | 0 | 0 | 0 | 0 | 0 | 0 | 0 | 1 |
| KCNU1 | 5 | 0 | 0 | 0 | 0 | 0 | 0 | 0 | 1 |
| USP34 | 4 | 0 | 1 | 0 | 0 | 0 | 0 | 0 | 1 |
| FASN | 5 | 0 | 0 | 0 | 0 | 0 | 0 | 0 | 1 |
| SIGLEC9 | 5 | 0 | 0 | 0 | 0 | 0 | 0 | 0 | 1 |

| | | | | | | | | | |
|---|---|---|---|---|---|---|---|---|---|
| AKAP13 | 3 | 0 | 1 | 1 | 0 | 0 | 0 | 0 | 1 |
| SEMA5A | 5 | 0 | 0 | 0 | 0 | 0 | 0 | 0 | 1 |
| DCHS1 | 4 | 1 | 0 | 0 | 0 | 0 | 0 | 0 | 1 |
| REV3L | 4 | 1 | 0 | 0 | 0 | 0 | 0 | 0 | 1 |
| SMC1B | 5 | 0 | 0 | 0 | 0 | 0 | 0 | 0 | 1 |
| MAP1A | 5 | 0 | 0 | 0 | 0 | 0 | 0 | 0 | 1 |
| OR2L13 | 5 | 0 | 0 | 0 | 0 | 0 | 0 | 0 | 1 |
| DNAH1 | 4 | 1 | 0 | 0 | 0 | 0 | 0 | 0 | 1 |
| ODZ3 | 5 | 0 | 0 | 0 | 0 | 0 | 0 | 0 | 1 |
| CDC5L | 4 | 1 | 0 | 0 | 0 | 0 | 0 | 0 | 1 |
| COL6A6 | 5 | 0 | 0 | 0 | 0 | 0 | 0 | 0 | 1 |
| PTPRT | 4 | 1 | 0 | 0 | 0 | 0 | 0 | 0 | 1 |
| OR2L3 | 5 | 0 | 0 | 0 | 0 | 0 | 0 | 0 | 1 |
| SLCO1B3 | 5 | 0 | 0 | 0 | 0 | 0 | 0 | 0 | 1 |
| CDK5RAP2 | 4 | 0 | 1 | 0 | 0 | 0 | 0 | 0 | 1 |
| AKAP11 | 5 | 0 | 0 | 0 | 0 | 0 | 0 | 0 | 1 |
| DNHD1 | 5 | 0 | 0 | 0 | 0 | 0 | 0 | 0 | 1 |
| CAPRIN2 | 4 | 0 | 1 | 0 | 0 | 0 | 0 | 0 | 1 |
| LRRC4C | 4 | 1 | 0 | 0 | 0 | 0 | 0 | 0 | 1 |
| WDR72 | 5 | 0 | 0 | 0 | 0 | 0 | 0 | 0 | 1 |
| RP1L1 | 5 | 0 | 0 | 0 | 0 | 0 | 0 | 0 | 1 |
| ATM | 4 | 1 | 0 | 0 | 0 | 0 | 0 | 0 | 1 |
| ABCA7 | 5 | 0 | 0 | 0 | 0 | 0 | 0 | 0 | 1 |
| TRPM1 | 5 | 0 | 0 | 0 | 0 | 0 | 0 | 0 | 1 |
| OR2T4 | 5 | 0 | 0 | 0 | 0 | 0 | 0 | 0 | 1 |
| IGF2BP3 | 5 | 0 | 0 | 0 | 0 | 0 | 0 | 0 | 1 |
| CELSR1 | 5 | 0 | 0 | 0 | 0 | 0 | 0 | 0 | 1 |
| CLASP2 | 4 | 0 | 0 | 0 | 1 | 0 | 0 | 0 | 1 |
| DNMBP | 5 | 0 | 0 | 0 | 0 | 0 | 0 | 0 | 1 |
| CCDC88C | 4 | 0 | 1 | 0 | 0 | 0 | 0 | 0 | 1 |
| ABCA9 | 5 | 0 | 0 | 0 | 0 | 0 | 0 | 0 | 1 |
| VWA5B1 | 5 | 0 | 0 | 0 | 0 | 0 | 0 | 0 | 1 |
| CRIM1 | 5 | 0 | 0 | 0 | 0 | 0 | 0 | 0 | 1 |
| ARHGAP31 | 5 | 0 | 0 | 0 | 0 | 0 | 0 | 0 | 1 |
| ENAM | 5 | 0 | 0 | 0 | 0 | 0 | 0 | 0 | 1 |
| HS3ST4 | 4 | 1 | 0 | 0 | 0 | 0 | 0 | 0 | 1 |
| LRBA | 5 | 0 | 0 | 0 | 0 | 0 | 0 | 0 | 1 |
| LAMA3 | 5 | 0 | 0 | 0 | 0 | 0 | 0 | 0 | 1 |
| RPGR | 5 | 0 | 0 | 0 | 0 | 0 | 0 | 0 | 1 |
| DGKK | 5 | 0 | 0 | 0 | 0 | 0 | 0 | 0 | 1 |
| MTUS1 | 5 | 0 | 0 | 0 | 0 | 0 | 0 | 0 | 1 |
| AKAP9 | 4 | 1 | 0 | 0 | 0 | 0 | 0 | 0 | 1 |
| CHD6 | 4 | 0 | 0 | 0 | 1 | 0 | 0 | 0 | 1 |
| ATP10B | 4 | 1 | 0 | 0 | 0 | 0 | 0 | 0 | 1 |
| POLE | 5 | 0 | 0 | 0 | 0 | 0 | 0 | 0 | 1 |
| RBBP6 | 5 | 0 | 0 | 0 | 0 | 0 | 0 | 0 | 1 |

| | | | | | | | | |
|---|---|---|---|---|---|---|---|---|
| CTTNBP2 | 5 | 0 | 0 | 0 | 0 | 0 | 0 | 0 | 1 |
| SVIL | 5 | 0 | 0 | 0 | 0 | 0 | 0 | 0 | 1 |
| ADAMTS12 | 4 | 1 | 0 | 0 | 0 | 0 | 0 | 0 | 1 |
| FAM171A1 | 5 | 0 | 0 | 0 | 0 | 0 | 0 | 0 | 1 |
| MYOF | 5 | 0 | 0 | 0 | 0 | 0 | 0 | 0 | 1 |
| DLG2 | 4 | 0 | 0 | 0 | 1 | 0 | 0 | 0 | 1 |
| ADAM28 | 5 | 0 | 0 | 0 | 0 | 0 | 0 | 0 | 1 |
| GCLM | 3 | 0 | 1 | 0 | 0 | 0 | 0 | 0 | 1 |
| ETHE1 | 3 | 0 | 1 | 0 | 0 | 0 | 0 | 0 | 1 |
| DKK2 | 2 | 2 | 0 | 0 | 0 | 0 | 0 | 0 | 1 |
| ATG4C | 2 | 2 | 0 | 0 | 0 | 0 | 0 | 0 | 1 |
| TAS2R7 | 3 | 0 | 0 | 0 | 0 | 1 | 0 | 0 | 1 |
| OR4C45 | 3 | 1 | 0 | 0 | 0 | 0 | 0 | 0 | 1 |
| RNF148 | 3 | 1 | 0 | 0 | 0 | 0 | 0 | 0 | 1 |
| SULT2A1 | 3 | 1 | 0 | 0 | 0 | 0 | 0 | 0 | 1 |
| OIT3 | 2 | 1 | 1 | 0 | 0 | 0 | 0 | 0 | 1 |
| AKR1D1 | 3 | 1 | 0 | 0 | 0 | 0 | 0 | 0 | 1 |
| EFCAB3 | 3 | 0 | 1 | 0 | 0 | 0 | 0 | 0 | 1 |
| DPPA3 | 4 | 0 | 0 | 0 | 0 | 0 | 0 | 0 | 1 |
| TULP3 | 3 | 1 | 0 | 0 | 0 | 0 | 0 | 0 | 1 |
| HPS5 | 2 | 1 | 1 | 0 | 0 | 0 | 0 | 0 | 1 |
| SNAP25 | 4 | 0 | 0 | 0 | 0 | 0 | 0 | 0 | 1 |
| CYP4X1 | 3 | 1 | 0 | 0 | 0 | 0 | 0 | 0 | 1 |
| TCEB3B | 2 | 2 | 0 | 0 | 0 | 0 | 0 | 0 | 1 |
| OR4D11 | 3 | 0 | 0 | 0 | 1 | 0 | 0 | 0 | 1 |
| FAM91A1 | 2 | 2 | 0 | 0 | 0 | 0 | 0 | 0 | 1 |
| DIRAS3 | 4 | 0 | 0 | 0 | 0 | 0 | 0 | 0 | 1 |
| ZNF883 | 4 | 0 | 0 | 0 | 0 | 0 | 0 | 0 | 1 |
| HHIP | 2 | 2 | 0 | 0 | 0 | 0 | 0 | 0 | 1 |
| CCDC19 | 3 | 1 | 0 | 0 | 0 | 0 | 0 | 0 | 1 |
| NTM | 3 | 0 | 1 | 0 | 0 | 0 | 0 | 0 | 1 |
| SUPT3H | 3 | 1 | 0 | 0 | 0 | 0 | 0 | 0 | 1 |
| CCDC28A | 4 | 0 | 0 | 0 | 0 | 0 | 0 | 0 | 1 |
| B4GALT6 | 3 | 0 | 0 | 0 | 1 | 0 | 0 | 0 | 1 |
| NDC80 | 3 | 1 | 0 | 0 | 0 | 0 | 0 | 0 | 1 |
| TNFSF10 | 4 | 0 | 0 | 0 | 0 | 0 | 0 | 0 | 1 |
| SLC26A3 | 2 | 0 | 2 | 0 | 0 | 0 | 0 | 0 | 1 |
| MAEL | 3 | 1 | 0 | 0 | 0 | 0 | 0 | 0 | 1 |
| DAP3 | 4 | 0 | 0 | 0 | 0 | 0 | 0 | 0 | 1 |
| OVCH2 | 3 | 0 | 1 | 0 | 0 | 0 | 0 | 0 | 1 |
| EGFL6 | 4 | 0 | 0 | 0 | 0 | 0 | 0 | 0 | 1 |
| KPNA7 | 3 | 1 | 0 | 0 | 0 | 0 | 0 | 0 | 1 |
| PALMD | 3 | 1 | 0 | 0 | 0 | 0 | 0 | 0 | 1 |
| THEMIS | 2 | 2 | 0 | 0 | 0 | 0 | 0 | 0 | 1 |
| CD27 | 4 | 0 | 0 | 0 | 0 | 0 | 0 | 0 | 1 |
| EPHX4 | 4 | 0 | 0 | 0 | 0 | 0 | 0 | 0 | 1 |

| | | | | | | | | | |
|---|---|---|---|---|---|---|---|---|---|
| ZBTB26 | 4 | 0 | 0 | 0 | 0 | 0 | 0 | 0 | 1 |
| CXorf30 | 3 | 1 | 0 | 0 | 0 | 0 | 0 | 0 | 1 |
| MS4A1 | 4 | 0 | 0 | 0 | 0 | 0 | 0 | 0 | 1 |
| TMEM202 | 4 | 0 | 0 | 0 | 0 | 0 | 0 | 0 | 1 |
| SDSL | 4 | 0 | 0 | 0 | 0 | 0 | 0 | 0 | 1 |
| UBE2Z | 3 | 1 | 0 | 0 | 0 | 0 | 0 | 0 | 1 |
| ALLC | 3 | 1 | 0 | 0 | 0 | 0 | 0 | 0 | 1 |
| SAMHD1 | 3 | 1 | 0 | 0 | 0 | 0 | 0 | 0 | 1 |
| ATP6V1B2 | 3 | 1 | 0 | 0 | 0 | 0 | 0 | 0 | 1 |
| FCER1A | 3 | 1 | 0 | 0 | 0 | 0 | 0 | 0 | 1 |
| MATK | 3 | 0 | 0 | 0 | 1 | 0 | 0 | 0 | 1 |
| CD1E | 3 | 1 | 0 | 0 | 0 | 0 | 0 | 0 | 1 |
| SUPT7L | 4 | 0 | 0 | 0 | 0 | 0 | 0 | 0 | 1 |
| EIF5 | 3 | 0 | 0 | 0 | 1 | 0 | 0 | 0 | 1 |
| ARHGEF3 | 3 | 1 | 0 | 0 | 0 | 0 | 0 | 0 | 1 |
| CCR6 | 4 | 0 | 0 | 0 | 0 | 0 | 0 | 0 | 1 |
| MRPL46 | 2 | 1 | 1 | 0 | 0 | 0 | 0 | 0 | 1 |
| ZCCHC5 | 3 | 1 | 0 | 0 | 0 | 0 | 0 | 0 | 1 |
| PLA2G4C | 3 | 0 | 1 | 0 | 0 | 0 | 0 | 0 | 1 |
| CD1C | 1 | 2 | 1 | 0 | 0 | 0 | 0 | 0 | 1 |
| SYT16 | 3 | 1 | 0 | 0 | 0 | 0 | 0 | 0 | 1 |
| MMP8 | 4 | 0 | 0 | 0 | 0 | 0 | 0 | 0 | 1 |
| TEKT3 | 3 | 0 | 1 | 0 | 0 | 0 | 0 | 0 | 1 |
| RNPS1 | 4 | 0 | 0 | 0 | 0 | 0 | 0 | 0 | 1 |
| CERKL | 3 | 1 | 0 | 0 | 0 | 0 | 0 | 0 | 1 |
| GDF3 | 4 | 0 | 0 | 0 | 0 | 0 | 0 | 0 | 1 |
| AGPS | 3 | 1 | 0 | 0 | 0 | 0 | 0 | 0 | 1 |
| OR6C75 | 4 | 0 | 0 | 0 | 0 | 0 | 0 | 0 | 1 |
| ARSJ | 3 | 1 | 0 | 0 | 0 | 0 | 0 | 0 | 1 |
| PTGIS | 4 | 0 | 0 | 0 | 0 | 0 | 0 | 0 | 1 |
| GMIP | 2 | 1 | 1 | 0 | 0 | 0 | 0 | 0 | 1 |
| GLP1R | 3 | 1 | 0 | 0 | 0 | 0 | 0 | 0 | 1 |
| SUV420H1 | 3 | 0 | 1 | 0 | 0 | 0 | 0 | 0 | 1 |
| ALDH9A1 | 4 | 0 | 0 | 0 | 0 | 0 | 0 | 0 | 1 |
| BMP15 | 3 | 0 | 0 | 0 | 1 | 0 | 0 | 0 | 1 |
| UGT2A1 | 3 | 1 | 0 | 0 | 0 | 0 | 0 | 0 | 1 |
| WLS | 3 | 1 | 0 | 0 | 0 | 0 | 0 | 0 | 1 |
| PRKCG | 2 | 1 | 1 | 0 | 0 | 0 | 0 | 0 | 1 |
| TAB2 | 3 | 1 | 0 | 0 | 0 | 0 | 0 | 0 | 1 |
| ASB17 | 4 | 0 | 0 | 0 | 0 | 0 | 0 | 0 | 1 |
| ARNT | 3 | 0 | 1 | 0 | 0 | 0 | 0 | 0 | 1 |
| LRRC46 | 4 | 0 | 0 | 0 | 0 | 0 | 0 | 0 | 1 |
| ALDH8A1 | 4 | 0 | 0 | 0 | 0 | 0 | 0 | 0 | 1 |
| ANGEL1 | 3 | 1 | 0 | 0 | 0 | 0 | 0 | 0 | 1 |
| SLC24A5 | 3 | 1 | 0 | 0 | 0 | 0 | 0 | 0 | 1 |
| BAP1 | 3 | 1 | 0 | 0 | 0 | 0 | 0 | 0 | 1 |

| | | | | | | | | | |
|---|---|---|---|---|---|---|---|---|---|
| ZNF132 | 3 | 1 | 0 | 0 | 0 | 0 | 0 | 0 | 1 |
| HEXDC | 4 | 0 | 0 | 0 | 0 | 0 | 0 | 0 | 1 |
| LPPR5 | 4 | 0 | 0 | 0 | 0 | 0 | 0 | 0 | 1 |
| RXRG | 4 | 0 | 0 | 0 | 0 | 0 | 0 | 0 | 1 |
| LPO | 3 | 1 | 0 | 0 | 0 | 0 | 0 | 0 | 1 |
| SERPINA7 | 4 | 0 | 0 | 0 | 0 | 0 | 0 | 0 | 1 |
| GTPBP10 | 4 | 0 | 0 | 0 | 0 | 0 | 0 | 0 | 1 |
| SPATA17 | 4 | 0 | 0 | 0 | 0 | 0 | 0 | 0 | 1 |
| GABPB2 | 4 | 0 | 0 | 0 | 0 | 0 | 0 | 0 | 1 |
| SORT1 | 3 | 1 | 0 | 0 | 0 | 0 | 0 | 0 | 1 |
| IL1RL2 | 3 | 0 | 1 | 0 | 0 | 0 | 0 | 0 | 1 |
| CAPN8 | 3 | 1 | 0 | 0 | 0 | 0 | 0 | 0 | 1 |
| RASA1 | 2 | 2 | 0 | 0 | 0 | 0 | 0 | 0 | 1 |
| SUN3 | 4 | 0 | 0 | 0 | 0 | 0 | 0 | 0 | 1 |
| KHDRBS3 | 3 | 1 | 0 | 0 | 0 | 0 | 0 | 0 | 1 |
| COG3 | 3 | 1 | 0 | 0 | 0 | 0 | 0 | 0 | 1 |
| OR6M1 | 4 | 0 | 0 | 0 | 0 | 0 | 0 | 0 | 1 |
| OR6C1 | 4 | 0 | 0 | 0 | 0 | 0 | 0 | 0 | 1 |
| USHBP1 | 3 | 1 | 0 | 0 | 0 | 0 | 0 | 0 | 1 |
| RALYL | 4 | 0 | 0 | 0 | 0 | 0 | 0 | 0 | 1 |
| FUT10 | 4 | 0 | 0 | 0 | 0 | 0 | 0 | 0 | 1 |
| FPR1 | 4 | 0 | 0 | 0 | 0 | 0 | 0 | 0 | 1 |
| CPEB3 | 3 | 0 | 1 | 0 | 0 | 0 | 0 | 0 | 1 |
| SLC4A9 | 3 | 0 | 1 | 0 | 0 | 0 | 0 | 0 | 1 |
| MMP19 | 4 | 0 | 0 | 0 | 0 | 0 | 0 | 0 | 1 |
| TRIT1 | 4 | 0 | 0 | 0 | 0 | 0 | 0 | 0 | 1 |
| KIAA1257 | 4 | 0 | 0 | 0 | 0 | 0 | 0 | 0 | 1 |
| VCP | 3 | 0 | 0 | 1 | 0 | 0 | 0 | 0 | 1 |
| AGXT2L1 | 4 | 0 | 0 | 0 | 0 | 0 | 0 | 0 | 1 |
| TXNDC2 | 3 | 1 | 0 | 0 | 0 | 0 | 0 | 0 | 1 |
| TBCEL | 4 | 0 | 0 | 0 | 0 | 0 | 0 | 0 | 1 |
| RFX8 | 3 | 1 | 0 | 0 | 0 | 0 | 0 | 0 | 1 |
| PDILT | 4 | 0 | 0 | 0 | 0 | 0 | 0 | 0 | 1 |
| IDO2 | 4 | 0 | 0 | 0 | 0 | 0 | 0 | 0 | 1 |
| VPS4A | 4 | 0 | 0 | 0 | 0 | 0 | 0 | 0 | 1 |
| OR4X1 | 4 | 0 | 0 | 0 | 0 | 0 | 0 | 0 | 1 |
| ITGB7 | 3 | 1 | 0 | 0 | 0 | 0 | 0 | 0 | 1 |
| SERPINA6 | 4 | 0 | 0 | 0 | 0 | 0 | 0 | 0 | 1 |
| IRF8 | 4 | 0 | 0 | 0 | 0 | 0 | 0 | 0 | 1 |
| WIPI1 | 4 | 0 | 0 | 0 | 0 | 0 | 0 | 0 | 1 |
| PLAGL1 | 4 | 0 | 0 | 0 | 0 | 0 | 0 | 0 | 1 |
| ZNF619 | 4 | 0 | 0 | 0 | 0 | 0 | 0 | 0 | 1 |
| C15orf33 | 4 | 0 | 0 | 0 | 0 | 0 | 0 | 0 | 1 |
| SLC7A13 | 4 | 0 | 0 | 0 | 0 | 0 | 0 | 0 | 1 |
| SLC39A6 | 4 | 0 | 0 | 0 | 0 | 0 | 0 | 0 | 1 |
| PSEN1 | 3 | 0 | 0 | 0 | 1 | 0 | 0 | 0 | 1 |

| | | | | | | | | | |
|---|---|---|---|---|---|---|---|---|---|
| CPVL | 4 | 0 | 0 | 0 | 0 | 0 | 0 | 0 | 1 |
| SLC41A2 | 4 | 0 | 0 | 0 | 0 | 0 | 0 | 0 | 1 |
| CATSPERB | 3 | 1 | 0 | 0 | 0 | 0 | 0 | 0 | 1 |
| AACS | 4 | 0 | 0 | 0 | 0 | 0 | 0 | 0 | 1 |
| PAX2 | 4 | 0 | 0 | 0 | 0 | 0 | 0 | 0 | 1 |
| CPT1A | 4 | 0 | 0 | 0 | 0 | 0 | 0 | 0 | 1 |
| CDR1 | 4 | 0 | 0 | 0 | 0 | 0 | 0 | 0 | 1 |
| FRK | 4 | 0 | 0 | 0 | 0 | 0 | 0 | 0 | 1 |
| BTNL8 | 4 | 0 | 0 | 0 | 0 | 0 | 0 | 0 | 1 |
| NOX5 | 3 | 1 | 0 | 0 | 0 | 0 | 0 | 0 | 1 |
| SAMD3 | 3 | 1 | 0 | 0 | 0 | 0 | 0 | 0 | 1 |
| VCL | 3 | 1 | 0 | 0 | 0 | 0 | 0 | 0 | 1 |
| PIK3R1 | 4 | 0 | 0 | 0 | 0 | 0 | 0 | 0 | 1 |
| LRRC3B | 4 | 0 | 0 | 0 | 0 | 0 | 0 | 0 | 1 |
| HTR3D | 4 | 0 | 0 | 0 | 0 | 0 | 0 | 0 | 1 |
| NUMB | 4 | 0 | 0 | 0 | 0 | 0 | 0 | 0 | 1 |
| SDPR | 4 | 0 | 0 | 0 | 0 | 0 | 0 | 0 | 1 |
| SLC17A3 | 4 | 0 | 0 | 0 | 0 | 0 | 0 | 0 | 1 |
| EXOC4 | 4 | 0 | 0 | 0 | 0 | 0 | 0 | 0 | 1 |
| RAP1GAP | 2 | 1 | 1 | 0 | 0 | 0 | 0 | 0 | 1 |
| EHHADH | 3 | 1 | 0 | 0 | 0 | 0 | 0 | 0 | 1 |
| TFEC | 4 | 0 | 0 | 0 | 0 | 0 | 0 | 0 | 1 |
| TMEM200A | 4 | 0 | 0 | 0 | 0 | 0 | 0 | 0 | 1 |
| UGT3A1 | 4 | 0 | 0 | 0 | 0 | 0 | 0 | 0 | 1 |
| PPIL2 | 4 | 0 | 0 | 0 | 0 | 0 | 0 | 0 | 1 |
| MAPK14 | 4 | 0 | 0 | 0 | 0 | 0 | 0 | 0 | 1 |
| SLCO6A1 | 3 | 0 | 1 | 0 | 0 | 0 | 0 | 0 | 1 |
| ZBTB2 | 4 | 0 | 0 | 0 | 0 | 0 | 0 | 0 | 1 |
| SPAG1 | 3 | 1 | 0 | 0 | 0 | 0 | 0 | 0 | 1 |
| SSX2IP | 4 | 0 | 0 | 0 | 0 | 0 | 0 | 0 | 1 |
| ENOX1 | 4 | 0 | 0 | 0 | 0 | 0 | 0 | 0 | 1 |
| PPM1F | 4 | 0 | 0 | 0 | 0 | 0 | 0 | 0 | 1 |
| C16orf89 | 3 | 0 | 1 | 0 | 0 | 0 | 0 | 0 | 1 |
| FGF10 | 4 | 0 | 0 | 0 | 0 | 0 | 0 | 0 | 1 |
| BTRC | 4 | 0 | 0 | 0 | 0 | 0 | 0 | 0 | 1 |
| CTNNAL1 | 4 | 0 | 0 | 0 | 0 | 0 | 0 | 0 | 1 |
| PAPOLB | 4 | 0 | 0 | 0 | 0 | 0 | 0 | 0 | 1 |
| MYOZ3 | 4 | 0 | 0 | 0 | 0 | 0 | 0 | 0 | 1 |
| STK32A | 4 | 0 | 0 | 0 | 0 | 0 | 0 | 0 | 1 |
| CHRNA1 | 4 | 0 | 0 | 0 | 0 | 0 | 0 | 0 | 1 |
| FMO3 | 3 | 1 | 0 | 0 | 0 | 0 | 0 | 0 | 1 |
| OR11H6 | 4 | 0 | 0 | 0 | 0 | 0 | 0 | 0 | 1 |
| VPS4B | 3 | 0 | 1 | 0 | 0 | 0 | 0 | 0 | 1 |
| PRKCE | 4 | 0 | 0 | 0 | 0 | 0 | 0 | 0 | 1 |
| ST6GAL1 | 4 | 0 | 0 | 0 | 0 | 0 | 0 | 0 | 1 |
| ITGAV | 3 | 1 | 0 | 0 | 0 | 0 | 0 | 0 | 1 |

| | | | | | | | | | |
|---|---|---|---|---|---|---|---|---|---|
| SAMD7 | 4 | 0 | 0 | 0 | 0 | 0 | 0 | 0 | 1 |
| EPB41L1 | 3 | 1 | 0 | 0 | 0 | 0 | 0 | 0 | 1 |
| JAKMIP3 | 3 | 1 | 0 | 0 | 0 | 0 | 0 | 0 | 1 |
| PIBF1 | 4 | 0 | 0 | 0 | 0 | 0 | 0 | 0 | 1 |
| TBCK | 2 | 1 | 0 | 0 | 1 | 0 | 0 | 0 | 1 |
| CSRP2BP | 4 | 0 | 0 | 0 | 0 | 0 | 0 | 0 | 1 |
| PTPN14 | 2 | 1 | 1 | 0 | 0 | 0 | 0 | 0 | 1 |
| SASS6 | 4 | 0 | 0 | 0 | 0 | 0 | 0 | 0 | 1 |
| GLP2R | 3 | 0 | 0 | 0 | 1 | 0 | 0 | 0 | 1 |
| EPB41L4B | 3 | 0 | 1 | 0 | 0 | 0 | 0 | 0 | 1 |
| THBS3 | 3 | 1 | 0 | 0 | 0 | 0 | 0 | 0 | 1 |
| OR10A7 | 3 | 0 | 0 | 0 | 1 | 0 | 0 | 0 | 1 |
| LEPREL1 | 3 | 0 | 1 | 0 | 0 | 0 | 0 | 0 | 1 |
| APOOL | 4 | 0 | 0 | 0 | 0 | 0 | 0 | 0 | 1 |
| KCTD16 | 4 | 0 | 0 | 0 | 0 | 0 | 0 | 0 | 1 |
| CPEB1 | 4 | 0 | 0 | 0 | 0 | 0 | 0 | 0 | 1 |
| SLC2A7 | 4 | 0 | 0 | 0 | 0 | 0 | 0 | 0 | 1 |
| SEC63 | 4 | 0 | 0 | 0 | 0 | 0 | 0 | 0 | 1 |
| EHD3 | 4 | 0 | 0 | 0 | 0 | 0 | 0 | 0 | 1 |
| GAS2L2 | 3 | 1 | 0 | 0 | 0 | 0 | 0 | 0 | 1 |
| CCDC151 | 4 | 0 | 0 | 0 | 0 | 0 | 0 | 0 | 1 |
| FAM161A | 4 | 0 | 0 | 0 | 0 | 0 | 0 | 0 | 1 |
| ACO2 | 3 | 1 | 0 | 0 | 0 | 0 | 0 | 0 | 1 |
| TTC25 | 4 | 0 | 0 | 0 | 0 | 0 | 0 | 0 | 1 |
| KRTAP24-1 | 4 | 0 | 0 | 0 | 0 | 0 | 0 | 0 | 1 |
| RASGRF2 | 3 | 0 | 1 | 0 | 0 | 0 | 0 | 0 | 1 |
| CD248 | 4 | 0 | 0 | 0 | 0 | 0 | 0 | 0 | 1 |
| SLC17A8 | 4 | 0 | 0 | 0 | 0 | 0 | 0 | 0 | 1 |
| ZMYND8 | 3 | 0 | 0 | 0 | 0 | 1 | 0 | 0 | 1 |
| WHSC1 | 3 | 0 | 1 | 0 | 0 | 0 | 0 | 0 | 1 |
| AMBN | 4 | 0 | 0 | 0 | 0 | 0 | 0 | 0 | 1 |
| DDX43 | 3 | 1 | 0 | 0 | 0 | 0 | 0 | 0 | 1 |
| LRRC31 | 4 | 0 | 0 | 0 | 0 | 0 | 0 | 0 | 1 |
| OR1D2 | 4 | 0 | 0 | 0 | 0 | 0 | 0 | 0 | 1 |
| MAP4K1 | 3 | 0 | 1 | 0 | 0 | 0 | 0 | 0 | 1 |
| CENPC1 | 2 | 2 | 0 | 0 | 0 | 0 | 0 | 0 | 1 |
| SLC1A6 | 4 | 0 | 0 | 0 | 0 | 0 | 0 | 0 | 1 |
| OR2F1 | 4 | 0 | 0 | 0 | 0 | 0 | 0 | 0 | 1 |
| LATS2 | 3 | 1 | 0 | 0 | 0 | 0 | 0 | 0 | 1 |
| MDH1B | 4 | 0 | 0 | 0 | 0 | 0 | 0 | 0 | 1 |
| NCKAP1L | 3 | 0 | 1 | 0 | 0 | 0 | 0 | 0 | 1 |
| LIPI | 4 | 0 | 0 | 0 | 0 | 0 | 0 | 0 | 1 |
| ANKRD55 | 4 | 0 | 0 | 0 | 0 | 0 | 0 | 0 | 1 |
| TLR7 | 3 | 1 | 0 | 0 | 0 | 0 | 0 | 0 | 1 |
| PKP2 | 3 | 1 | 0 | 0 | 0 | 0 | 0 | 0 | 1 |
| ANXA6 | 4 | 0 | 0 | 0 | 0 | 0 | 0 | 0 | 1 |

| | | | | | | | | | |
|---|---|---|---|---|---|---|---|---|---|
| NNT | 3 | 0 | 1 | 0 | 0 | 0 | 0 | 0 | 1 |
| COG2 | 4 | 0 | 0 | 0 | 0 | 0 | 0 | 0 | 1 |
| FER1L5 | 2 | 0 | 2 | 0 | 0 | 0 | 0 | 0 | 1 |
| ATP9A | 3 | 1 | 0 | 0 | 0 | 0 | 0 | 0 | 1 |
| CNKSR1 | 4 | 0 | 0 | 0 | 0 | 0 | 0 | 0 | 1 |
| MMP27 | 4 | 0 | 0 | 0 | 0 | 0 | 0 | 0 | 1 |
| KRT76 | 4 | 0 | 0 | 0 | 0 | 0 | 0 | 0 | 1 |
| ATP8B4 | 3 | 1 | 0 | 0 | 0 | 0 | 0 | 0 | 1 |
| OR6C76 | 4 | 0 | 0 | 0 | 0 | 0 | 0 | 0 | 1 |
| DDR1 | 4 | 0 | 0 | 0 | 0 | 0 | 0 | 0 | 1 |
| SYT1 | 3 | 0 | 1 | 0 | 0 | 0 | 0 | 0 | 1 |
| GRID2 | 2 | 2 | 0 | 0 | 0 | 0 | 0 | 0 | 1 |
| OR9G9 | 4 | 0 | 0 | 0 | 0 | 0 | 0 | 0 | 1 |
| PNLIP | 4 | 0 | 0 | 0 | 0 | 0 | 0 | 0 | 1 |
| CD244 | 3 | 1 | 0 | 0 | 0 | 0 | 0 | 0 | 1 |
| MAMDC2 | 3 | 0 | 1 | 0 | 0 | 0 | 0 | 0 | 1 |
| LNPEP | 3 | 1 | 0 | 0 | 0 | 0 | 0 | 0 | 1 |
| DNA2 | 3 | 0 | 0 | 0 | 1 | 0 | 0 | 0 | 1 |
| ZEB1 | 3 | 1 | 0 | 0 | 0 | 0 | 0 | 0 | 1 |
| ZNF439 | 4 | 0 | 0 | 0 | 0 | 0 | 0 | 0 | 1 |
| PCSK6 | 3 | 0 | 0 | 0 | 1 | 0 | 0 | 0 | 1 |
| EFTUD2 | 3 | 1 | 0 | 0 | 0 | 0 | 0 | 0 | 1 |
| FAM186B | 4 | 0 | 0 | 0 | 0 | 0 | 0 | 0 | 1 |
| KLHL25 | 4 | 0 | 0 | 0 | 0 | 0 | 0 | 0 | 1 |
| C2CD2L | 4 | 0 | 0 | 0 | 0 | 0 | 0 | 0 | 1 |
| SP140L | 4 | 0 | 0 | 0 | 0 | 0 | 0 | 0 | 1 |
| C1orf127 | 4 | 0 | 0 | 0 | 0 | 0 | 0 | 0 | 1 |
| GPR111 | 4 | 0 | 0 | 0 | 0 | 0 | 0 | 0 | 1 |
| FANCB | 4 | 0 | 0 | 0 | 0 | 0 | 0 | 0 | 1 |
| MED17 | 4 | 0 | 0 | 0 | 0 | 0 | 0 | 0 | 1 |
| GABRA6 | 4 | 0 | 0 | 0 | 0 | 0 | 0 | 0 | 1 |
| EPHB1 | 3 | 0 | 1 | 0 | 0 | 0 | 0 | 0 | 1 |
| FBXL7 | 4 | 0 | 0 | 0 | 0 | 0 | 0 | 0 | 1 |
| ELAVL4 | 4 | 0 | 0 | 0 | 0 | 0 | 0 | 0 | 1 |
| ST7 | 4 | 0 | 0 | 0 | 0 | 0 | 0 | 0 | 1 |
| OTOA | 3 | 1 | 0 | 0 | 0 | 0 | 0 | 0 | 1 |
| SLCO2B1 | 4 | 0 | 0 | 0 | 0 | 0 | 0 | 0 | 1 |
| GBA3 | 4 | 0 | 0 | 0 | 0 | 0 | 0 | 0 | 1 |
| MAP3K7 | 4 | 0 | 0 | 0 | 0 | 0 | 0 | 0 | 1 |
| ZSWIM3 | 4 | 0 | 0 | 0 | 0 | 0 | 0 | 0 | 1 |
| FLJ43860 | 3 | 0 | 1 | 0 | 0 | 0 | 0 | 0 | 1 |
| EPHA8 | 3 | 1 | 0 | 0 | 0 | 0 | 0 | 0 | 1 |
| TMTC2 | 3 | 1 | 0 | 0 | 0 | 0 | 0 | 0 | 1 |
| GCNT2 | 4 | 0 | 0 | 0 | 0 | 0 | 0 | 0 | 1 |
| FLI1 | 4 | 0 | 0 | 0 | 0 | 0 | 0 | 0 | 1 |
| NAA35 | 4 | 0 | 0 | 0 | 0 | 0 | 0 | 0 | 1 |

| | | | | | | | | | |
|---|---|---|---|---|---|---|---|---|---|
| SLC26A9 | 4 | 0 | 0 | 0 | 0 | 0 | 0 | 0 | 1 |
| FBXO18 | 4 | 0 | 0 | 0 | 0 | 0 | 0 | 0 | 1 |
| ARHGAP17 | 4 | 0 | 0 | 0 | 0 | 0 | 0 | 0 | 1 |
| HRG | 4 | 0 | 0 | 0 | 0 | 0 | 0 | 0 | 1 |
| ILDR2 | 4 | 0 | 0 | 0 | 0 | 0 | 0 | 0 | 1 |
| NWD1 | 3 | 1 | 0 | 0 | 0 | 0 | 0 | 0 | 1 |
| SPAG16 | 4 | 0 | 0 | 0 | 0 | 0 | 0 | 0 | 1 |
| ANGPT1 | 4 | 0 | 0 | 0 | 0 | 0 | 0 | 0 | 1 |
| OR52L1 | 4 | 0 | 0 | 0 | 0 | 0 | 0 | 0 | 1 |
| SLCO2A1 | 4 | 0 | 0 | 0 | 0 | 0 | 0 | 0 | 1 |
| A1CF | 4 | 0 | 0 | 0 | 0 | 0 | 0 | 0 | 1 |
| TLR10 | 4 | 0 | 0 | 0 | 0 | 0 | 0 | 0 | 1 |
| ZNF350 | 4 | 0 | 0 | 0 | 0 | 0 | 0 | 0 | 1 |
| PCK1 | 4 | 0 | 0 | 0 | 0 | 0 | 0 | 0 | 1 |
| MATN2 | 4 | 0 | 0 | 0 | 0 | 0 | 0 | 0 | 1 |
| PDE6B | 4 | 0 | 0 | 0 | 0 | 0 | 0 | 0 | 1 |
| SH3D19 | 4 | 0 | 0 | 0 | 0 | 0 | 0 | 0 | 1 |
| C3orf15 | 4 | 0 | 0 | 0 | 0 | 0 | 0 | 0 | 1 |
| SLC14A1 | 4 | 0 | 0 | 0 | 0 | 0 | 0 | 0 | 1 |
| EPN3 | 4 | 0 | 0 | 0 | 0 | 0 | 0 | 0 | 1 |
| CHRD | 4 | 0 | 0 | 0 | 0 | 0 | 0 | 0 | 1 |
| PRB3 | 4 | 0 | 0 | 0 | 0 | 0 | 0 | 0 | 1 |
| HIST1H1C | 4 | 0 | 0 | 0 | 0 | 0 | 0 | 0 | 1 |
| ZCCHC2 | 3 | 1 | 0 | 0 | 0 | 0 | 0 | 0 | 1 |
| TARS2 | 4 | 0 | 0 | 0 | 0 | 0 | 0 | 0 | 1 |
| KIAA1407 | 4 | 0 | 0 | 0 | 0 | 0 | 0 | 0 | 1 |
| YSK4 | 3 | 1 | 0 | 0 | 0 | 0 | 0 | 0 | 1 |
| ZNF491 | 4 | 0 | 0 | 0 | 0 | 0 | 0 | 0 | 1 |
| CD97 | 4 | 0 | 0 | 0 | 0 | 0 | 0 | 0 | 1 |
| C11orf30 | 3 | 0 | 1 | 0 | 0 | 0 | 0 | 0 | 1 |
| GAD1 | 4 | 0 | 0 | 0 | 0 | 0 | 0 | 0 | 1 |
| TMPRSS3 | 4 | 0 | 0 | 0 | 0 | 0 | 0 | 0 | 1 |
| STAC | 4 | 0 | 0 | 0 | 0 | 0 | 0 | 0 | 1 |
| GAS2L3 | 3 | 0 | 1 | 0 | 0 | 0 | 0 | 0 | 1 |
| GALNT6 | 4 | 0 | 0 | 0 | 0 | 0 | 0 | 0 | 1 |
| USP25 | 4 | 0 | 0 | 0 | 0 | 0 | 0 | 0 | 1 |
| GARS | 4 | 0 | 0 | 0 | 0 | 0 | 0 | 0 | 1 |
| RNF31 | 4 | 0 | 0 | 0 | 0 | 0 | 0 | 0 | 1 |
| CAST | 3 | 1 | 0 | 0 | 0 | 0 | 0 | 0 | 1 |
| DDX46 | 4 | 0 | 0 | 0 | 0 | 0 | 0 | 0 | 1 |
| SLC1A3 | 4 | 0 | 0 | 0 | 0 | 0 | 0 | 0 | 1 |
| SEMA3D | 3 | 1 | 0 | 0 | 0 | 0 | 0 | 0 | 1 |
| LAMB3 | 3 | 1 | 0 | 0 | 0 | 0 | 0 | 0 | 1 |
| BACH2 | 3 | 1 | 0 | 0 | 0 | 0 | 0 | 0 | 1 |
| XRCC5 | 4 | 0 | 0 | 0 | 0 | 0 | 0 | 0 | 1 |
| RBM12 | 4 | 0 | 0 | 0 | 0 | 0 | 0 | 0 | 1 |

| | | | | | | | | | |
|---|---|---|---|---|---|---|---|---|---|
| CACNA2D4 | 3 | 1 | 0 | 0 | 0 | 0 | 0 | 0 | 1 |
| EPS15L1 | 4 | 0 | 0 | 0 | 0 | 0 | 0 | 0 | 1 |
| SV2C | 4 | 0 | 0 | 0 | 0 | 0 | 0 | 0 | 1 |
| C11orf63 | 4 | 0 | 0 | 0 | 0 | 0 | 0 | 0 | 1 |
| TLR2 | 4 | 0 | 0 | 0 | 0 | 0 | 0 | 0 | 1 |
| SEMA4D | 4 | 0 | 0 | 0 | 0 | 0 | 0 | 0 | 1 |
| FRMD7 | 4 | 0 | 0 | 0 | 0 | 0 | 0 | 0 | 1 |
| AMICA1 | 4 | 0 | 0 | 0 | 0 | 0 | 0 | 0 | 1 |
| GDAP1 | 4 | 0 | 0 | 0 | 0 | 0 | 0 | 0 | 1 |
| SCIN | 4 | 0 | 0 | 0 | 0 | 0 | 0 | 0 | 1 |
| MACC1 | 4 | 0 | 0 | 0 | 0 | 0 | 0 | 0 | 1 |
| SARM1 | 4 | 0 | 0 | 0 | 0 | 0 | 0 | 0 | 1 |
| ZNF222 | 3 | 1 | 0 | 0 | 0 | 0 | 0 | 0 | 1 |
| FAM194B | 4 | 0 | 0 | 0 | 0 | 0 | 0 | 0 | 1 |
| CCDC136 | 4 | 0 | 0 | 0 | 0 | 0 | 0 | 0 | 1 |
| MARCO | 4 | 0 | 0 | 0 | 0 | 0 | 0 | 0 | 1 |
| ARHGEF9 | 4 | 0 | 0 | 0 | 0 | 0 | 0 | 0 | 1 |
| RERGL | 4 | 0 | 0 | 0 | 0 | 0 | 0 | 0 | 1 |
| KCNH1 | 3 | 1 | 0 | 0 | 0 | 0 | 0 | 0 | 1 |
| PTK2B | 3 | 0 | 1 | 0 | 0 | 0 | 0 | 0 | 1 |
| CSF3R | 4 | 0 | 0 | 0 | 0 | 0 | 0 | 0 | 1 |
| TMEM132C | 3 | 1 | 0 | 0 | 0 | 0 | 0 | 0 | 1 |
| CDH5 | 4 | 0 | 0 | 0 | 0 | 0 | 0 | 0 | 1 |
| NINL | 2 | 1 | 1 | 0 | 0 | 0 | 0 | 0 | 1 |
| YTHDC2 | 2 | 2 | 0 | 0 | 0 | 0 | 0 | 0 | 1 |
| FAM59A | 4 | 0 | 0 | 0 | 0 | 0 | 0 | 0 | 1 |
| LAMC2 | 3 | 1 | 0 | 0 | 0 | 0 | 0 | 0 | 1 |
| DAGLA | 4 | 0 | 0 | 0 | 0 | 0 | 0 | 0 | 1 |
| NOL4 | 4 | 0 | 0 | 0 | 0 | 0 | 0 | 0 | 1 |
| CAPN9 | 4 | 0 | 0 | 0 | 0 | 0 | 0 | 0 | 1 |
| GABRA1 | 4 | 0 | 0 | 0 | 0 | 0 | 0 | 0 | 1 |
| LIFR | 4 | 0 | 0 | 0 | 0 | 0 | 0 | 0 | 1 |
| CEP152 | 3 | 1 | 0 | 0 | 0 | 0 | 0 | 0 | 1 |
| PTCH2 | 4 | 0 | 0 | 0 | 0 | 0 | 0 | 0 | 1 |
| ZNF442 | 4 | 0 | 0 | 0 | 0 | 0 | 0 | 0 | 1 |
| KIAA0195 | 4 | 0 | 0 | 0 | 0 | 0 | 0 | 0 | 1 |
| GABRA5 | 4 | 0 | 0 | 0 | 0 | 0 | 0 | 0 | 1 |
| NR3C2 | 3 | 1 | 0 | 0 | 0 | 0 | 0 | 0 | 1 |
| ABCG1 | 4 | 0 | 0 | 0 | 0 | 0 | 0 | 0 | 1 |
| ZNF483 | 4 | 0 | 0 | 0 | 0 | 0 | 0 | 0 | 1 |
| ZNF433 | 4 | 0 | 0 | 0 | 0 | 0 | 0 | 0 | 1 |
| BRSK1 | 4 | 0 | 0 | 0 | 0 | 0 | 0 | 0 | 1 |
| L3MBTL4 | 3 | 0 | 0 | 0 | 1 | 0 | 0 | 0 | 1 |
| TRIM37 | 3 | 1 | 0 | 0 | 0 | 0 | 0 | 0 | 1 |
| SLC26A8 | 3 | 0 | 1 | 0 | 0 | 0 | 0 | 0 | 1 |
| FAM65B | 4 | 0 | 0 | 0 | 0 | 0 | 0 | 0 | 1 |

| | | | | | | | | | |
|---|---|---|---|---|---|---|---|---|---|
| TJP3 | 2 | 1 | 1 | 0 | 0 | 0 | 0 | 0 | 1 |
| ANKRD24 | 4 | 0 | 0 | 0 | 0 | 0 | 0 | 0 | 1 |
| LMOD2 | 4 | 0 | 0 | 0 | 0 | 0 | 0 | 0 | 1 |
| DAB1 | 4 | 0 | 0 | 0 | 0 | 0 | 0 | 0 | 1 |
| KCNH4 | 3 | 1 | 0 | 0 | 0 | 0 | 0 | 0 | 1 |
| SLC6A12 | 4 | 0 | 0 | 0 | 0 | 0 | 0 | 0 | 1 |
| NOC3L | 4 | 0 | 0 | 0 | 0 | 0 | 0 | 0 | 1 |
| ADAM32 | 4 | 0 | 0 | 0 | 0 | 0 | 0 | 0 | 1 |
| TOM1L2 | 4 | 0 | 0 | 0 | 0 | 0 | 0 | 0 | 1 |
| SESN2 | 4 | 0 | 0 | 0 | 0 | 0 | 0 | 0 | 1 |
| KIRREL2 | 4 | 0 | 0 | 0 | 0 | 0 | 0 | 0 | 1 |
| GAB4 | 4 | 0 | 0 | 0 | 0 | 0 | 0 | 0 | 1 |
| PRDM14 | 4 | 0 | 0 | 0 | 0 | 0 | 0 | 0 | 1 |
| PCDHGA8 | 4 | 0 | 0 | 0 | 0 | 0 | 0 | 0 | 1 |
| SMC5 | 4 | 0 | 0 | 0 | 0 | 0 | 0 | 0 | 1 |
| PRKCA | 4 | 0 | 0 | 0 | 0 | 0 | 0 | 0 | 1 |
| CNTNAP1 | 4 | 0 | 0 | 0 | 0 | 0 | 0 | 0 | 1 |
| MTMR8 | 4 | 0 | 0 | 0 | 0 | 0 | 0 | 0 | 1 |
| INCENP | 4 | 0 | 0 | 0 | 0 | 0 | 0 | 0 | 1 |
| LTBP2 | 3 | 1 | 0 | 0 | 0 | 0 | 0 | 0 | 1 |
| USP48 | 4 | 0 | 0 | 0 | 0 | 0 | 0 | 0 | 1 |
| SLC3A1 | 4 | 0 | 0 | 0 | 0 | 0 | 0 | 0 | 1 |
| BAZ2A | 3 | 0 | 1 | 0 | 0 | 0 | 0 | 0 | 1 |
| ZNF354C | 4 | 0 | 0 | 0 | 0 | 0 | 0 | 0 | 1 |
| ALDH1L2 | 4 | 0 | 0 | 0 | 0 | 0 | 0 | 0 | 1 |
| ALOX5 | 4 | 0 | 0 | 0 | 0 | 0 | 0 | 0 | 1 |
| KIAA1524 | 4 | 0 | 0 | 0 | 0 | 0 | 0 | 0 | 1 |
| PARP8 | 3 | 0 | 0 | 0 | 1 | 0 | 0 | 0 | 1 |
| LRRC43 | 4 | 0 | 0 | 0 | 0 | 0 | 0 | 0 | 1 |
| SLC27A1 | 3 | 0 | 1 | 0 | 0 | 0 | 0 | 0 | 1 |
| SLC4A7 | 3 | 1 | 0 | 0 | 0 | 0 | 0 | 0 | 1 |
| ANKRD5 | 4 | 0 | 0 | 0 | 0 | 0 | 0 | 0 | 1 |
| ATXN2L | 4 | 0 | 0 | 0 | 0 | 0 | 0 | 0 | 1 |
| PCDHB6 | 4 | 0 | 0 | 0 | 0 | 0 | 0 | 0 | 1 |
| TNFRSF8 | 4 | 0 | 0 | 0 | 0 | 0 | 0 | 0 | 1 |
| AP4E1 | 4 | 0 | 0 | 0 | 0 | 0 | 0 | 0 | 1 |
| COPA | 4 | 0 | 0 | 0 | 0 | 0 | 0 | 0 | 1 |
| MCM4 | 4 | 0 | 0 | 0 | 0 | 0 | 0 | 0 | 1 |
| DDX4 | 4 | 0 | 0 | 0 | 0 | 0 | 0 | 0 | 1 |
| NETO1 | 4 | 0 | 0 | 0 | 0 | 0 | 0 | 0 | 1 |
| DENND1B | 4 | 0 | 0 | 0 | 0 | 0 | 0 | 0 | 1 |
| ZNF366 | 4 | 0 | 0 | 0 | 0 | 0 | 0 | 0 | 1 |
| ADAM20 | 4 | 0 | 0 | 0 | 0 | 0 | 0 | 0 | 1 |
| PCDHAC2 | 4 | 0 | 0 | 0 | 0 | 0 | 0 | 0 | 1 |
| WDR19 | 4 | 0 | 0 | 0 | 0 | 0 | 0 | 0 | 1 |
| GALNT9 | 4 | 0 | 0 | 0 | 0 | 0 | 0 | 0 | 1 |

| | | | | | | | | | |
|---|---|---|---|---|---|---|---|---|---|
| ROR1 | 4 | 0 | 0 | 0 | 0 | 0 | 0 | 0 | 1 |
| IKBKAP | 3 | 1 | 0 | 0 | 0 | 0 | 0 | 0 | 1 |
| BRIP1 | 4 | 0 | 0 | 0 | 0 | 0 | 0 | 0 | 1 |
| PSD | 3 | 0 | 0 | 0 | 1 | 0 | 0 | 0 | 1 |
| KIF18B | 4 | 0 | 0 | 0 | 0 | 0 | 0 | 0 | 1 |
| FLRT2 | 4 | 0 | 0 | 0 | 0 | 0 | 0 | 0 | 1 |
| EBF2 | 4 | 0 | 0 | 0 | 0 | 0 | 0 | 0 | 1 |
| JAKMIP2 | 4 | 0 | 0 | 0 | 0 | 0 | 0 | 0 | 1 |
| ATAD2 | 3 | 1 | 0 | 0 | 0 | 0 | 0 | 0 | 1 |
| MTMR3 | 4 | 0 | 0 | 0 | 0 | 0 | 0 | 0 | 1 |
| PEAR1 | 4 | 0 | 0 | 0 | 0 | 0 | 0 | 0 | 1 |
| DIS3L | 4 | 0 | 0 | 0 | 0 | 0 | 0 | 0 | 1 |
| CLVS2 | 4 | 0 | 0 | 0 | 0 | 0 | 0 | 0 | 1 |
| KDM3B | 3 | 0 | 1 | 0 | 0 | 0 | 0 | 0 | 1 |
| TBX18 | 4 | 0 | 0 | 0 | 0 | 0 | 0 | 0 | 1 |
| FAM190A | 4 | 0 | 0 | 0 | 0 | 0 | 0 | 0 | 1 |
| GRM7 | 4 | 0 | 0 | 0 | 0 | 0 | 0 | 0 | 1 |
| SEZ6L2 | 3 | 0 | 1 | 0 | 0 | 0 | 0 | 0 | 1 |
| ZNF780B | 3 | 1 | 0 | 0 | 0 | 0 | 0 | 0 | 1 |
| DDC | 4 | 0 | 0 | 0 | 0 | 0 | 0 | 0 | 1 |
| ENPP3 | 4 | 0 | 0 | 0 | 0 | 0 | 0 | 0 | 1 |
| BAIAP3 | 3 | 0 | 1 | 0 | 0 | 0 | 0 | 0 | 1 |
| C10orf68 | 4 | 0 | 0 | 0 | 0 | 0 | 0 | 0 | 1 |
| CACHD1 | 4 | 0 | 0 | 0 | 0 | 0 | 0 | 0 | 1 |
| FRMPD2 | 4 | 0 | 0 | 0 | 0 | 0 | 0 | 0 | 1 |
| HGF | 4 | 0 | 0 | 0 | 0 | 0 | 0 | 0 | 1 |
| C6orf118 | 4 | 0 | 0 | 0 | 0 | 0 | 0 | 0 | 1 |
| PHF14 | 4 | 0 | 0 | 0 | 0 | 0 | 0 | 0 | 1 |
| ZNF560 | 4 | 0 | 0 | 0 | 0 | 0 | 0 | 0 | 1 |
| ATP2B3 | 4 | 0 | 0 | 0 | 0 | 0 | 0 | 0 | 1 |
| PCDHGB6 | 4 | 0 | 0 | 0 | 0 | 0 | 0 | 0 | 1 |
| DMGDH | 4 | 0 | 0 | 0 | 0 | 0 | 0 | 0 | 1 |
| DCAF4L2 | 3 | 1 | 0 | 0 | 0 | 0 | 0 | 0 | 1 |
| C18orf34 | 3 | 1 | 0 | 0 | 0 | 0 | 0 | 0 | 1 |
| DSG4 | 4 | 0 | 0 | 0 | 0 | 0 | 0 | 0 | 1 |
| PPP2R3A | 3 | 1 | 0 | 0 | 0 | 0 | 0 | 0 | 1 |
| SLC4A8 | 4 | 0 | 0 | 0 | 0 | 0 | 0 | 0 | 1 |
| TBX5 | 4 | 0 | 0 | 0 | 0 | 0 | 0 | 0 | 1 |
| MYO1A | 4 | 0 | 0 | 0 | 0 | 0 | 0 | 0 | 1 |
| GPR126 | 4 | 0 | 0 | 0 | 0 | 0 | 0 | 0 | 1 |
| RC3H1 | 4 | 0 | 0 | 0 | 0 | 0 | 0 | 0 | 1 |
| FSTL4 | 4 | 0 | 0 | 0 | 0 | 0 | 0 | 0 | 1 |
| TRIP12 | 2 | 0 | 1 | 0 | 1 | 0 | 0 | 0 | 1 |
| HFM1 | 4 | 0 | 0 | 0 | 0 | 0 | 0 | 0 | 1 |
| ALPK1 | 4 | 0 | 0 | 0 | 0 | 0 | 0 | 0 | 1 |
| CYFIP2 | 4 | 0 | 0 | 0 | 0 | 0 | 0 | 0 | 1 |

| | | | | | | | | | |
|---|---|---|---|---|---|---|---|---|---|
| PCDHAC1 | 4 | 0 | 0 | 0 | 0 | 0 | 0 | 0 | 1 |
| AGAP2 | 4 | 0 | 0 | 0 | 0 | 0 | 0 | 0 | 1 |
| WNK3 | 3 | 1 | 0 | 0 | 0 | 0 | 0 | 0 | 1 |
| KIAA0146 | 4 | 0 | 0 | 0 | 0 | 0 | 0 | 0 | 1 |
| NMUR2 | 4 | 0 | 0 | 0 | 0 | 0 | 0 | 0 | 1 |
| CCDC141 | 4 | 0 | 0 | 0 | 0 | 0 | 0 | 0 | 1 |
| UGGT1 | 3 | 1 | 0 | 0 | 0 | 0 | 0 | 0 | 1 |
| EFCAB6 | 3 | 1 | 0 | 0 | 0 | 0 | 0 | 0 | 1 |
| CAMTA1 | 4 | 0 | 0 | 0 | 0 | 0 | 0 | 0 | 1 |
| BNC1 | 4 | 0 | 0 | 0 | 0 | 0 | 0 | 0 | 1 |
| KIF6 | 4 | 0 | 0 | 0 | 0 | 0 | 0 | 0 | 1 |
| UBQLNL | 4 | 0 | 0 | 0 | 0 | 0 | 0 | 0 | 1 |
| RANBP17 | 4 | 0 | 0 | 0 | 0 | 0 | 0 | 0 | 1 |
| PCDHA11 | 4 | 0 | 0 | 0 | 0 | 0 | 0 | 0 | 1 |
| SPHK2 | 3 | 1 | 0 | 0 | 0 | 0 | 0 | 0 | 1 |
| PRPF8 | 3 | 1 | 0 | 0 | 0 | 0 | 0 | 0 | 1 |
| TPP2 | 3 | 0 | 1 | 0 | 0 | 0 | 0 | 0 | 1 |
| PIK3R5 | 4 | 0 | 0 | 0 | 0 | 0 | 0 | 0 | 1 |
| PTPN3 | 4 | 0 | 0 | 0 | 0 | 0 | 0 | 0 | 1 |
| IFT172 | 3 | 0 | 0 | 0 | 1 | 0 | 0 | 0 | 1 |
| HDAC4 | 4 | 0 | 0 | 0 | 0 | 0 | 0 | 0 | 1 |
| ZNF267 | 4 | 0 | 0 | 0 | 0 | 0 | 0 | 0 | 1 |
| ENG | 3 | 1 | 0 | 0 | 0 | 0 | 0 | 0 | 1 |
| ARHGAP26 | 4 | 0 | 0 | 0 | 0 | 0 | 0 | 0 | 1 |
| C14orf37 | 4 | 0 | 0 | 0 | 0 | 0 | 0 | 0 | 1 |
| TEX14 | 4 | 0 | 0 | 0 | 0 | 0 | 0 | 0 | 1 |
| STAG2 | 3 | 1 | 0 | 0 | 0 | 0 | 0 | 0 | 1 |
| WIPF1 | 4 | 0 | 0 | 0 | 0 | 0 | 0 | 0 | 1 |
| HIPK2 | 4 | 0 | 0 | 0 | 0 | 0 | 0 | 0 | 1 |
| ETV1 | 3 | 1 | 0 | 0 | 0 | 0 | 0 | 0 | 1 |
| ITGA7 | 4 | 0 | 0 | 0 | 0 | 0 | 0 | 0 | 1 |
| EPB41L3 | 4 | 0 | 0 | 0 | 0 | 0 | 0 | 0 | 1 |
| UBE4A | 3 | 1 | 0 | 0 | 0 | 0 | 0 | 0 | 1 |
| SLITRK4 | 4 | 0 | 0 | 0 | 0 | 0 | 0 | 0 | 1 |
| SREBF2 | 4 | 0 | 0 | 0 | 0 | 0 | 0 | 0 | 1 |
| PLEKHA5 | 4 | 0 | 0 | 0 | 0 | 0 | 0 | 0 | 1 |
| WHSC1L1 | 4 | 0 | 0 | 0 | 0 | 0 | 0 | 0 | 1 |
| ATP11C | 4 | 0 | 0 | 0 | 0 | 0 | 0 | 0 | 1 |
| ARHGEF10 | 3 | 1 | 0 | 0 | 0 | 0 | 0 | 0 | 1 |
| PHLDB2 | 4 | 0 | 0 | 0 | 0 | 0 | 0 | 0 | 1 |
| UHRF1BP1 | 4 | 0 | 0 | 0 | 0 | 0 | 0 | 0 | 1 |
| ACTN2 | 4 | 0 | 0 | 0 | 0 | 0 | 0 | 0 | 1 |
| FBXO38 | 4 | 0 | 0 | 0 | 0 | 0 | 0 | 0 | 1 |
| DGKD | 4 | 0 | 0 | 0 | 0 | 0 | 0 | 0 | 1 |
| SP140 | 4 | 0 | 0 | 0 | 0 | 0 | 0 | 0 | 1 |
| CDHR2 | 4 | 0 | 0 | 0 | 0 | 0 | 0 | 0 | 1 |

| | | | | | | | | | |
|---|---|---|---|---|---|---|---|---|---|
| FBXW7 | 4 | 0 | 0 | 0 | 0 | 0 | 0 | 0 | 1 |
| GIF | 4 | 0 | 0 | 0 | 0 | 0 | 0 | 0 | 1 |
| SLC15A5 | 3 | 1 | 0 | 0 | 0 | 0 | 0 | 0 | 1 |
| HCFC1 | 4 | 0 | 0 | 0 | 0 | 0 | 0 | 0 | 1 |
| ADCY5 | 4 | 0 | 0 | 0 | 0 | 0 | 0 | 0 | 1 |
| UPF2 | 4 | 0 | 0 | 0 | 0 | 0 | 0 | 0 | 1 |
| BAI1 | 3 | 0 | 1 | 0 | 0 | 0 | 0 | 0 | 1 |
| PDZRN4 | 3 | 1 | 0 | 0 | 0 | 0 | 0 | 0 | 1 |
| TYK2 | 4 | 0 | 0 | 0 | 0 | 0 | 0 | 0 | 1 |
| MAGEC3 | 4 | 0 | 0 | 0 | 0 | 0 | 0 | 0 | 1 |
| GLIS3 | 4 | 0 | 0 | 0 | 0 | 0 | 0 | 0 | 1 |
| TAF2 | 4 | 0 | 0 | 0 | 0 | 0 | 0 | 0 | 1 |
| PCDHGA1 | 4 | 0 | 0 | 0 | 0 | 0 | 0 | 0 | 1 |
| IGF2R | 3 | 0 | 1 | 0 | 0 | 0 | 0 | 0 | 1 |
| C11orf82 | 4 | 0 | 0 | 0 | 0 | 0 | 0 | 0 | 1 |
| IFT140 | 4 | 0 | 0 | 0 | 0 | 0 | 0 | 0 | 1 |
| KCNK18 | 4 | 0 | 0 | 0 | 0 | 0 | 0 | 0 | 1 |
| INTS1 | 4 | 0 | 0 | 0 | 0 | 0 | 0 | 0 | 1 |
| ADAR | 4 | 0 | 0 | 0 | 0 | 0 | 0 | 0 | 1 |
| ZNF496 | 4 | 0 | 0 | 0 | 0 | 0 | 0 | 0 | 1 |
| ZFR2 | 4 | 0 | 0 | 0 | 0 | 0 | 0 | 0 | 1 |
| SPTB | 3 | 1 | 0 | 0 | 0 | 0 | 0 | 0 | 1 |
| ANLN | 4 | 0 | 0 | 0 | 0 | 0 | 0 | 0 | 1 |
| GPR156 | 4 | 0 | 0 | 0 | 0 | 0 | 0 | 0 | 1 |
| PDZD8 | 4 | 0 | 0 | 0 | 0 | 0 | 0 | 0 | 1 |
| NCOA6 | 2 | 2 | 0 | 0 | 0 | 0 | 0 | 0 | 1 |
| LAMC1 | 4 | 0 | 0 | 0 | 0 | 0 | 0 | 0 | 1 |
| IFT122 | 4 | 0 | 0 | 0 | 0 | 0 | 0 | 0 | 1 |
| RGS3 | 4 | 0 | 0 | 0 | 0 | 0 | 0 | 0 | 1 |
| WWC2 | 4 | 0 | 0 | 0 | 0 | 0 | 0 | 0 | 1 |
| PLEKHM2 | 4 | 0 | 0 | 0 | 0 | 0 | 0 | 0 | 1 |
| ZBTB41 | 3 | 1 | 0 | 0 | 0 | 0 | 0 | 0 | 1 |
| RREB1 | 4 | 0 | 0 | 0 | 0 | 0 | 0 | 0 | 1 |
| PLXNB1 | 4 | 0 | 0 | 0 | 0 | 0 | 0 | 0 | 1 |
| SMC3 | 3 | 1 | 0 | 0 | 0 | 0 | 0 | 0 | 1 |
| VPS13A | 2 | 0 | 2 | 0 | 0 | 0 | 0 | 0 | 1 |
| TRPS1 | 3 | 0 | 0 | 0 | 1 | 0 | 0 | 0 | 1 |
| SGSM1 | 4 | 0 | 0 | 0 | 0 | 0 | 0 | 0 | 1 |
| KIAA0240 | 4 | 0 | 0 | 0 | 0 | 0 | 0 | 0 | 1 |
| ARAP3 | 4 | 0 | 0 | 0 | 0 | 0 | 0 | 0 | 1 |
| IDO1 | 4 | 0 | 0 | 0 | 0 | 0 | 0 | 0 | 1 |
| OR2L2 | 4 | 0 | 0 | 0 | 0 | 0 | 0 | 0 | 1 |
| PANK4 | 4 | 0 | 0 | 0 | 0 | 0 | 0 | 0 | 1 |
| NAALADL2 | 3 | 1 | 0 | 0 | 0 | 0 | 0 | 0 | 1 |
| FCGR2A | 3 | 1 | 0 | 0 | 0 | 0 | 0 | 0 | 1 |
| ATG2B | 3 | 1 | 0 | 0 | 0 | 0 | 0 | 0 | 1 |

| | | | | | | | | | |
|---|---|---|---|---|---|---|---|---|---|
| ADAMTS15 | 3 | 1 | 0 | 0 | 0 | 0 | 0 | 0 | 1 |
| GUCY2F | 4 | 0 | 0 | 0 | 0 | 0 | 0 | 0 | 1 |
| ITGB4 | 4 | 0 | 0 | 0 | 0 | 0 | 0 | 0 | 1 |
| LPPR4 | 4 | 0 | 0 | 0 | 0 | 0 | 0 | 0 | 1 |
| KCNMA1 | 4 | 0 | 0 | 0 | 0 | 0 | 0 | 0 | 1 |
| AP3B2 | 4 | 0 | 0 | 0 | 0 | 0 | 0 | 0 | 1 |
| TBC1D5 | 4 | 0 | 0 | 0 | 0 | 0 | 0 | 0 | 1 |
| PCDH10 | 4 | 0 | 0 | 0 | 0 | 0 | 0 | 0 | 1 |
| DOCK11 | 4 | 0 | 0 | 0 | 0 | 0 | 0 | 0 | 1 |
| ABCC2 | 4 | 0 | 0 | 0 | 0 | 0 | 0 | 0 | 1 |
| ZNF214 | 4 | 0 | 0 | 0 | 0 | 0 | 0 | 0 | 1 |
| DEPDC5 | 4 | 0 | 0 | 0 | 0 | 0 | 0 | 0 | 1 |
| ECT2 | 4 | 0 | 0 | 0 | 0 | 0 | 0 | 0 | 1 |
| CIC | 3 | 1 | 0 | 0 | 0 | 0 | 0 | 0 | 1 |
| NOVA1 | 3 | 1 | 0 | 0 | 0 | 0 | 0 | 0 | 1 |
| SLC5A4 | 4 | 0 | 0 | 0 | 0 | 0 | 0 | 0 | 1 |
| NEO1 | 4 | 0 | 0 | 0 | 0 | 0 | 0 | 0 | 1 |
| ABCA5 | 3 | 1 | 0 | 0 | 0 | 0 | 0 | 0 | 1 |
| COL20A1 | 4 | 0 | 0 | 0 | 0 | 0 | 0 | 0 | 1 |
| ZNF541 | 4 | 0 | 0 | 0 | 0 | 0 | 0 | 0 | 1 |
| C10orf71 | 4 | 0 | 0 | 0 | 0 | 0 | 0 | 0 | 1 |
| DISC1 | 4 | 0 | 0 | 0 | 0 | 0 | 0 | 0 | 1 |
| C9orf71 | 4 | 0 | 0 | 0 | 0 | 0 | 0 | 0 | 1 |
| RPS6KA6 | 4 | 0 | 0 | 0 | 0 | 0 | 0 | 0 | 1 |
| DTNA | 4 | 0 | 0 | 0 | 0 | 0 | 0 | 0 | 1 |
| GARNL3 | 4 | 0 | 0 | 0 | 0 | 0 | 0 | 0 | 1 |
| MYO9A | 3 | 1 | 0 | 0 | 0 | 0 | 0 | 0 | 1 |
| PCF11 | 3 | 1 | 0 | 0 | 0 | 0 | 0 | 0 | 1 |
| ACE | 4 | 0 | 0 | 0 | 0 | 0 | 0 | 0 | 1 |
| GRB10 | 4 | 0 | 0 | 0 | 0 | 0 | 0 | 0 | 1 |
| BEND2 | 4 | 0 | 0 | 0 | 0 | 0 | 0 | 0 | 1 |
| DACT1 | 4 | 0 | 0 | 0 | 0 | 0 | 0 | 0 | 1 |
| PPIP5K2 | 4 | 0 | 0 | 0 | 0 | 0 | 0 | 0 | 1 |
| SIN3A | 4 | 0 | 0 | 0 | 0 | 0 | 0 | 0 | 1 |
| POLR1A | 4 | 0 | 0 | 0 | 0 | 0 | 0 | 0 | 1 |
| MYOM3 | 4 | 0 | 0 | 0 | 0 | 0 | 0 | 0 | 1 |
| SLC35F1 | 4 | 0 | 0 | 0 | 0 | 0 | 0 | 0 | 1 |
| ITGB8 | 4 | 0 | 0 | 0 | 0 | 0 | 0 | 0 | 1 |
| COL1A1 | 4 | 0 | 0 | 0 | 0 | 0 | 0 | 0 | 1 |
| PRKCZ | 3 | 0 | 1 | 0 | 0 | 0 | 0 | 0 | 1 |
| TRPC4 | 4 | 0 | 0 | 0 | 0 | 0 | 0 | 0 | 1 |
| EIF4G1 | 4 | 0 | 0 | 0 | 0 | 0 | 0 | 0 | 1 |
| KIF16B | 4 | 0 | 0 | 0 | 0 | 0 | 0 | 0 | 1 |
| UBR3 | 4 | 0 | 0 | 0 | 0 | 0 | 0 | 0 | 1 |
| MYCBPAP | 4 | 0 | 0 | 0 | 0 | 0 | 0 | 0 | 1 |
| BCOR | 4 | 0 | 0 | 0 | 0 | 0 | 0 | 0 | 1 |

| | | | | | | | | | |
|---|---|---|---|---|---|---|---|---|---|
| GGT5 | 4 | 0 | 0 | 0 | 0 | 0 | 0 | 0 | 1 |
| GBF1 | 4 | 0 | 0 | 0 | 0 | 0 | 0 | 0 | 1 |
| TAF1 | 4 | 0 | 0 | 0 | 0 | 0 | 0 | 0 | 1 |
| GLI3 | 3 | 1 | 0 | 0 | 0 | 0 | 0 | 0 | 1 |
| RERE | 4 | 0 | 0 | 0 | 0 | 0 | 0 | 0 | 1 |
| CASR | 4 | 0 | 0 | 0 | 0 | 0 | 0 | 0 | 1 |
| MBD5 | 4 | 0 | 0 | 0 | 0 | 0 | 0 | 0 | 1 |
| DDR2 | 3 | 1 | 0 | 0 | 0 | 0 | 0 | 0 | 1 |
| MYT1 | 4 | 0 | 0 | 0 | 0 | 0 | 0 | 0 | 1 |
| SRRM2 | 3 | 1 | 0 | 0 | 0 | 0 | 0 | 0 | 1 |
| POLD1 | 4 | 0 | 0 | 0 | 0 | 0 | 0 | 0 | 1 |
| RAPGEF6 | 4 | 0 | 0 | 0 | 0 | 0 | 0 | 0 | 1 |
| MAST2 | 4 | 0 | 0 | 0 | 0 | 0 | 0 | 0 | 1 |
| MICAL3 | 4 | 0 | 0 | 0 | 0 | 0 | 0 | 0 | 1 |
| NIPBL | 3 | 1 | 0 | 0 | 0 | 0 | 0 | 0 | 1 |
| NEK4 | 4 | 0 | 0 | 0 | 0 | 0 | 0 | 0 | 1 |
| SNED1 | 4 | 0 | 0 | 0 | 0 | 0 | 0 | 0 | 1 |
| OPRM1 | 3 | 1 | 0 | 0 | 0 | 0 | 0 | 0 | 1 |
| PCDH11X | 4 | 0 | 0 | 0 | 0 | 0 | 0 | 0 | 1 |
| CD207 | 4 | 0 | 0 | 0 | 0 | 0 | 0 | 0 | 1 |
| OR5AS1 | 4 | 0 | 0 | 0 | 0 | 0 | 0 | 0 | 1 |
| LRP6 | 4 | 0 | 0 | 0 | 0 | 0 | 0 | 0 | 1 |
| CHD5 | 4 | 0 | 0 | 0 | 0 | 0 | 0 | 0 | 1 |
| ROCK2 | 3 | 1 | 0 | 0 | 0 | 0 | 0 | 0 | 1 |
| DLG5 | 3 | 0 | 0 | 0 | 1 | 0 | 0 | 0 | 1 |
| PRDM5 | 4 | 0 | 0 | 0 | 0 | 0 | 0 | 0 | 1 |
| EPRS | 4 | 0 | 0 | 0 | 0 | 0 | 0 | 0 | 1 |
| DOCK8 | 4 | 0 | 0 | 0 | 0 | 0 | 0 | 0 | 1 |
| FCRL3 | 4 | 0 | 0 | 0 | 0 | 0 | 0 | 0 | 1 |
| EXPH5 | 4 | 0 | 0 | 0 | 0 | 0 | 0 | 0 | 1 |
| CDH1 | 4 | 0 | 0 | 0 | 0 | 0 | 0 | 0 | 1 |
| ZDBF2 | 4 | 0 | 0 | 0 | 0 | 0 | 0 | 0 | 1 |
| OR8H1 | 4 | 0 | 0 | 0 | 0 | 0 | 0 | 0 | 1 |
| ABCA1 | 3 | 0 | 0 | 0 | 1 | 0 | 0 | 0 | 1 |
| SPTBN1 | 3 | 0 | 0 | 0 | 1 | 0 | 0 | 0 | 1 |
| MYOCD | 4 | 0 | 0 | 0 | 0 | 0 | 0 | 0 | 1 |
| ZFP106 | 4 | 0 | 0 | 0 | 0 | 0 | 0 | 0 | 1 |
| LY9 | 4 | 0 | 0 | 0 | 0 | 0 | 0 | 0 | 1 |
| F13A1 | 4 | 0 | 0 | 0 | 0 | 0 | 0 | 0 | 1 |
| DPP6 | 4 | 0 | 0 | 0 | 0 | 0 | 0 | 0 | 1 |
| EML5 | 4 | 0 | 0 | 0 | 0 | 0 | 0 | 0 | 1 |
| NRAP | 3 | 1 | 0 | 0 | 0 | 0 | 0 | 0 | 1 |
| PTPRG | 4 | 0 | 0 | 0 | 0 | 0 | 0 | 0 | 1 |
| MAP1B | 4 | 0 | 0 | 0 | 0 | 0 | 0 | 0 | 1 |
| NDST1 | 4 | 0 | 0 | 0 | 0 | 0 | 0 | 0 | 1 |
| CR1 | 3 | 1 | 0 | 0 | 0 | 0 | 0 | 0 | 1 |

| | | | | | | | | |
|---|---|---|---|---|---|---|---|---|
| FRMPD1 | 3 | 1 | 0 | 0 | 0 | 0 | 0 | 0 | 1 |
| RUSC2 | 4 | 0 | 0 | 0 | 0 | 0 | 0 | 0 | 1 |
| MYH15 | 3 | 1 | 0 | 0 | 0 | 0 | 0 | 0 | 1 |
| KIAA0284 | 4 | 0 | 0 | 0 | 0 | 0 | 0 | 0 | 1 |
| LRRC16B | 4 | 0 | 0 | 0 | 0 | 0 | 0 | 0 | 1 |
| ROBO1 | 4 | 0 | 0 | 0 | 0 | 0 | 0 | 0 | 1 |
| KIAA1614 | 4 | 0 | 0 | 0 | 0 | 0 | 0 | 0 | 1 |
| DENND4B | 3 | 0 | 0 | 0 | 1 | 0 | 0 | 0 | 1 |
| HACE1 | 4 | 0 | 0 | 0 | 0 | 0 | 0 | 0 | 1 |
| SON | 4 | 0 | 0 | 0 | 0 | 0 | 0 | 0 | 1 |
| CKAP5 | 4 | 0 | 0 | 0 | 0 | 0 | 0 | 0 | 1 |
| SORCS2 | 4 | 0 | 0 | 0 | 0 | 0 | 0 | 0 | 1 |
| CEP250 | 4 | 0 | 0 | 0 | 0 | 0 | 0 | 0 | 1 |
| GLI1 | 4 | 0 | 0 | 0 | 0 | 0 | 0 | 0 | 1 |
| MYT1L | 4 | 0 | 0 | 0 | 0 | 0 | 0 | 0 | 1 |
| EMR1 | 4 | 0 | 0 | 0 | 0 | 0 | 0 | 0 | 1 |
| C4orf21 | 3 | 0 | 1 | 0 | 0 | 0 | 0 | 0 | 1 |
| NAV2 | 3 | 0 | 0 | 0 | 1 | 0 | 0 | 0 | 1 |
| BCL9 | 4 | 0 | 0 | 0 | 0 | 0 | 0 | 0 | 1 |
| LTBP1 | 4 | 0 | 0 | 0 | 0 | 0 | 0 | 0 | 1 |
| COL18A1 | 3 | 0 | 0 | 0 | 1 | 0 | 0 | 0 | 1 |
| PDE4DIP | 2 | 0 | 2 | 0 | 0 | 0 | 0 | 0 | 1 |
| ZNF462 | 4 | 0 | 0 | 0 | 0 | 0 | 0 | 0 | 1 |
| SPEN | 4 | 0 | 0 | 0 | 0 | 0 | 0 | 0 | 1 |
| AGBL1 | 4 | 0 | 0 | 0 | 0 | 0 | 0 | 0 | 1 |
| SLC5A8 | 4 | 0 | 0 | 0 | 0 | 0 | 0 | 0 | 1 |
| OR5T2 | 4 | 0 | 0 | 0 | 0 | 0 | 0 | 0 | 1 |
| RIMBP2 | 4 | 0 | 0 | 0 | 0 | 0 | 0 | 0 | 1 |
| ARNT2 | 4 | 0 | 0 | 0 | 0 | 0 | 0 | 0 | 1 |
| SLC15A1 | 3 | 0 | 1 | 0 | 0 | 0 | 0 | 0 | 1 |
| CENPJ | 4 | 0 | 0 | 0 | 0 | 0 | 0 | 0 | 1 |
| PCNXL3 | 4 | 0 | 0 | 0 | 0 | 0 | 0 | 0 | 1 |
| ADCY2 | 4 | 0 | 0 | 0 | 0 | 0 | 0 | 0 | 1 |
| CLNK | 4 | 0 | 0 | 0 | 0 | 0 | 0 | 0 | 1 |
| TDRD6 | 4 | 0 | 0 | 0 | 0 | 0 | 0 | 0 | 1 |
| PER3 | 3 | 1 | 0 | 0 | 0 | 0 | 0 | 0 | 1 |
| ABCA2 | 4 | 0 | 0 | 0 | 0 | 0 | 0 | 0 | 1 |
| CA10 | 4 | 0 | 0 | 0 | 0 | 0 | 0 | 0 | 1 |
| KCNH5 | 4 | 0 | 0 | 0 | 0 | 0 | 0 | 0 | 1 |
| KIAA2026 | 4 | 0 | 0 | 0 | 0 | 0 | 0 | 0 | 1 |
| THBS2 | 4 | 0 | 0 | 0 | 0 | 0 | 0 | 0 | 1 |
| MUC12 | 4 | 0 | 0 | 0 | 0 | 0 | 0 | 0 | 1 |
| KIF24 | 4 | 0 | 0 | 0 | 0 | 0 | 0 | 0 | 1 |
| GRAMD1B | 4 | 0 | 0 | 0 | 0 | 0 | 0 | 0 | 1 |
| DUOX2 | 2 | 1 | 0 | 0 | 0 | 1 | 0 | 0 | 1 |
| IGSF22 | 4 | 0 | 0 | 0 | 0 | 0 | 0 | 0 | 1 |

| | | | | | | | | | |
|---|---|---|---|---|---|---|---|---|---|
| OR2M2 | 4 | 0 | 0 | 0 | 0 | 0 | 0 | 0 | 1 |
| CHRM3 | 3 | 1 | 0 | 0 | 0 | 0 | 0 | 0 | 1 |
| GPRIN1 | 4 | 0 | 0 | 0 | 0 | 0 | 0 | 0 | 1 |
| MAML2 | 3 | 0 | 1 | 0 | 0 | 0 | 0 | 0 | 1 |
| MSR1 | 4 | 0 | 0 | 0 | 0 | 0 | 0 | 0 | 1 |
| MYBPC2 | 4 | 0 | 0 | 0 | 0 | 0 | 0 | 0 | 1 |
| SETD2 | 4 | 0 | 0 | 0 | 0 | 0 | 0 | 0 | 1 |
| TNRC6A | 4 | 0 | 0 | 0 | 0 | 0 | 0 | 0 | 1 |
| ANKHD1 | 4 | 0 | 0 | 0 | 0 | 0 | 0 | 0 | 1 |
| PCSK5 | 4 | 0 | 0 | 0 | 0 | 0 | 0 | 0 | 1 |
| USP24 | 3 | 0 | 1 | 0 | 0 | 0 | 0 | 0 | 1 |
| MAP3K13 | 4 | 0 | 0 | 0 | 0 | 0 | 0 | 0 | 1 |
| SENP7 | 2 | 1 | 0 | 0 | 1 | 0 | 0 | 0 | 1 |
| SLC6A13 | 4 | 0 | 0 | 0 | 0 | 0 | 0 | 0 | 1 |
| SMARCA4 | 4 | 0 | 0 | 0 | 0 | 0 | 0 | 0 | 1 |
| BRWD3 | 4 | 0 | 0 | 0 | 0 | 0 | 0 | 0 | 1 |
| IGSF9B | 4 | 0 | 0 | 0 | 0 | 0 | 0 | 0 | 1 |
| SOBP | 4 | 0 | 0 | 0 | 0 | 0 | 0 | 0 | 1 |
| CABIN1 | 4 | 0 | 0 | 0 | 0 | 0 | 0 | 0 | 1 |
| NTRK3 | 3 | 0 | 0 | 0 | 1 | 0 | 0 | 0 | 1 |
| SMG1 | 3 | 1 | 0 | 0 | 0 | 0 | 0 | 0 | 1 |
| TRHDE | 4 | 0 | 0 | 0 | 0 | 0 | 0 | 0 | 1 |
| DMXL1 | 4 | 0 | 0 | 0 | 0 | 0 | 0 | 0 | 1 |
| PGAP1 | 4 | 0 | 0 | 0 | 0 | 0 | 0 | 0 | 1 |
| TET3 | 4 | 0 | 0 | 0 | 0 | 0 | 0 | 0 | 1 |
| PTCH1 | 3 | 1 | 0 | 0 | 0 | 0 | 0 | 0 | 1 |
| ZNF292 | 4 | 0 | 0 | 0 | 0 | 0 | 0 | 0 | 1 |
| ARHGEF12 | 3 | 1 | 0 | 0 | 0 | 0 | 0 | 0 | 1 |
| PIKFYVE | 3 | 0 | 1 | 0 | 0 | 0 | 0 | 0 | 1 |
| SEL1L3 | 4 | 0 | 0 | 0 | 0 | 0 | 0 | 0 | 1 |
| ZFC3H1 | 4 | 0 | 0 | 0 | 0 | 0 | 0 | 0 | 1 |
| APC | 3 | 1 | 0 | 0 | 0 | 0 | 0 | 0 | 1 |
| NEDD4 | 4 | 0 | 0 | 0 | 0 | 0 | 0 | 0 | 1 |
| ANXA3 | 4 | 0 | 0 | 0 | 0 | 0 | 0 | 0 | 1 |
| NCOA3 | 3 | 1 | 0 | 0 | 0 | 0 | 0 | 0 | 1 |
| JMJD1C | 4 | 0 | 0 | 0 | 0 | 0 | 0 | 0 | 1 |
| FUT9 | 4 | 0 | 0 | 0 | 0 | 0 | 0 | 0 | 1 |
| NRG3 | 4 | 0 | 0 | 0 | 0 | 0 | 0 | 0 | 1 |
| KIAA1009 | 3 | 0 | 0 | 0 | 1 | 0 | 0 | 0 | 1 |
| ACACB | 4 | 0 | 0 | 0 | 0 | 0 | 0 | 0 | 1 |
| SEMA3E | 3 | 1 | 0 | 0 | 0 | 0 | 0 | 0 | 1 |
| TNXB | 4 | 0 | 0 | 0 | 0 | 0 | 0 | 0 | 1 |
| DOCK1 | 3 | 0 | 0 | 0 | 0 | 1 | 0 | 0 | 1 |
| OR5F1 | 3 | 0 | 0 | 1 | 0 | 0 | 0 | 0 | 1 |
| EXOC7 | 2 | 0 | 1 | 0 | 1 | 0 | 0 | 0 | 1 |
| TPR | 4 | 0 | 0 | 0 | 0 | 0 | 0 | 0 | 1 |

| | | | | | | | | | |
|---|---|---|---|---|---|---|---|---|---|
| WDR67 | 3 | 1 | 0 | 0 | 0 | 0 | 0 | 0 | 1 |
| WNK2 | 4 | 0 | 0 | 0 | 0 | 0 | 0 | 0 | 1 |
| SULF1 | 3 | 0 | 1 | 0 | 0 | 0 | 0 | 0 | 1 |
| COL13A1 | 4 | 0 | 0 | 0 | 0 | 0 | 0 | 0 | 1 |
| PIWIL1 | 4 | 0 | 0 | 0 | 0 | 0 | 0 | 0 | 1 |
| PLD5 | 4 | 0 | 0 | 0 | 0 | 0 | 0 | 0 | 1 |
| ACPL2 | 4 | 0 | 0 | 0 | 0 | 0 | 0 | 0 | 1 |
| CACNA1B | 4 | 0 | 0 | 0 | 0 | 0 | 0 | 0 | 1 |
| LILRB4 | 4 | 0 | 0 | 0 | 0 | 0 | 0 | 0 | 1 |
| KCNH8 | 4 | 0 | 0 | 0 | 0 | 0 | 0 | 0 | 1 |
| APOC2 | 3 | 0 | 0 | 0 | 0 | 0 | 0 | 0 | 1 |
| C17orf63 | 3 | 0 | 0 | 0 | 0 | 0 | 0 | 0 | 1 |
| ZNHIT1 | 2 | 1 | 0 | 0 | 0 | 0 | 0 | 0 | 1 |
| UTS2D | 3 | 0 | 0 | 0 | 0 | 0 | 0 | 0 | 1 |
| TSPAN19 | 1 | 1 | 1 | 0 | 0 | 0 | 0 | 0 | 1 |
| PTHLH | 2 | 1 | 0 | 0 | 0 | 0 | 0 | 0 | 1 |
| CD1D | 1 | 2 | 0 | 0 | 0 | 0 | 0 | 0 | 1 |
| TMX1 | 2 | 1 | 0 | 0 | 0 | 0 | 0 | 0 | 1 |
| RHO | 2 | 1 | 0 | 0 | 0 | 0 | 0 | 0 | 1 |
| C10orf122 | 2 | 1 | 0 | 0 | 0 | 0 | 0 | 0 | 1 |
| PDCL2 | 2 | 1 | 0 | 0 | 0 | 0 | 0 | 0 | 1 |
| OSCP1 | 2 | 0 | 1 | 0 | 0 | 0 | 0 | 0 | 1 |
| ETFA | 2 | 0 | 1 | 0 | 0 | 0 | 0 | 0 | 1 |
| DDX19A | 1 | 1 | 0 | 0 | 1 | 0 | 0 | 0 | 1 |
| SSR3 | 2 | 0 | 0 | 0 | 1 | 0 | 0 | 0 | 1 |
| PTN | 2 | 1 | 0 | 0 | 0 | 0 | 0 | 0 | 1 |
| ERI3 | 2 | 1 | 0 | 0 | 0 | 0 | 0 | 0 | 1 |
| KRTAP25-1 | 3 | 0 | 0 | 0 | 0 | 0 | 0 | 0 | 1 |
| REG1A | 2 | 1 | 0 | 0 | 0 | 0 | 0 | 0 | 1 |
| KCNIP4 | 2 | 0 | 1 | 0 | 0 | 0 | 0 | 0 | 1 |
| PLAC8 | 3 | 0 | 0 | 0 | 0 | 0 | 0 | 0 | 1 |
| PPME1 | 2 | 0 | 1 | 0 | 0 | 0 | 0 | 0 | 1 |
| FAM53C | 2 | 1 | 0 | 0 | 0 | 0 | 0 | 0 | 1 |
| C6orf201 | 3 | 0 | 0 | 0 | 0 | 0 | 0 | 0 | 1 |
| DYRK3 | 1 | 2 | 0 | 0 | 0 | 0 | 0 | 0 | 1 |
| TPK1 | 2 | 1 | 0 | 0 | 0 | 0 | 0 | 0 | 1 |
| C1orf198 | 1 | 1 | 0 | 0 | 1 | 0 | 0 | 0 | 1 |
| LGSN | 2 | 1 | 0 | 0 | 0 | 0 | 0 | 0 | 1 |
| MPP6 | 1 | 1 | 1 | 0 | 0 | 0 | 0 | 0 | 1 |
| SERPINB11 | 2 | 1 | 0 | 0 | 0 | 0 | 0 | 0 | 1 |
| OR2AT4 | 2 | 1 | 0 | 0 | 0 | 0 | 0 | 0 | 1 |
| FOXI1 | 3 | 0 | 0 | 0 | 0 | 0 | 0 | 0 | 1 |
| CLEC4D | 3 | 0 | 0 | 0 | 0 | 0 | 0 | 0 | 1 |
| SLC36A3 | 2 | 0 | 1 | 0 | 0 | 0 | 0 | 0 | 1 |
| MYL1 | 3 | 0 | 0 | 0 | 0 | 0 | 0 | 0 | 1 |
| GTSF1 | 3 | 0 | 0 | 0 | 0 | 0 | 0 | 0 | 1 |

| | | | | | | | | | |
|---|---|---|---|---|---|---|---|---|---|
| F3 | 2 | 1 | 0 | 0 | 0 | 0 | 0 | 0 | 1 |
| RASSF8 | 2 | 1 | 0 | 0 | 0 | 0 | 0 | 0 | 1 |
| BSX | 3 | 0 | 0 | 0 | 0 | 0 | 0 | 0 | 1 |
| HBEGF | 3 | 0 | 0 | 0 | 0 | 0 | 0 | 0 | 1 |
| KAT5 | 1 | 1 | 0 | 0 | 1 | 0 | 0 | 0 | 1 |
| ZC3H8 | 3 | 0 | 0 | 0 | 0 | 0 | 0 | 0 | 1 |
| TAS2R42 | 2 | 1 | 0 | 0 | 0 | 0 | 0 | 0 | 1 |
| ASS1 | 2 | 1 | 0 | 0 | 0 | 0 | 0 | 0 | 1 |
| ANAPC10 | 3 | 0 | 0 | 0 | 0 | 0 | 0 | 0 | 1 |
| ASB2 | 2 | 1 | 0 | 0 | 0 | 0 | 0 | 0 | 1 |
| APBB2 | 1 | 2 | 0 | 0 | 0 | 0 | 0 | 0 | 1 |
| TMEM182 | 2 | 0 | 0 | 0 | 1 | 0 | 0 | 0 | 1 |
| ALDH2 | 2 | 1 | 0 | 0 | 0 | 0 | 0 | 0 | 1 |
| MSI2 | 2 | 0 | 1 | 0 | 0 | 0 | 0 | 0 | 1 |
| TIMM50 | 2 | 1 | 0 | 0 | 0 | 0 | 0 | 0 | 1 |
| PLA2G2F | 3 | 0 | 0 | 0 | 0 | 0 | 0 | 0 | 1 |
| FN3KRP | 3 | 0 | 0 | 0 | 0 | 0 | 0 | 0 | 1 |
| GNAQ | 2 | 1 | 0 | 0 | 0 | 0 | 0 | 0 | 1 |
| RAB39B | 3 | 0 | 0 | 0 | 0 | 0 | 0 | 0 | 1 |
| FBXO28 | 2 | 0 | 0 | 0 | 1 | 0 | 0 | 0 | 1 |
| LPXN | 2 | 1 | 0 | 0 | 0 | 0 | 0 | 0 | 1 |
| BMP3 | 2 | 1 | 0 | 0 | 0 | 0 | 0 | 0 | 1 |
| RGS14 | 3 | 0 | 0 | 0 | 0 | 0 | 0 | 0 | 1 |
| RERG | 3 | 0 | 0 | 0 | 0 | 0 | 0 | 0 | 1 |
| PCDP1 | 2 | 0 | 1 | 0 | 0 | 0 | 0 | 0 | 1 |
| IBSP | 3 | 0 | 0 | 0 | 0 | 0 | 0 | 0 | 1 |
| CLEC7A | 3 | 0 | 0 | 0 | 0 | 0 | 0 | 0 | 1 |
| ASGR2 | 2 | 0 | 1 | 0 | 0 | 0 | 0 | 0 | 1 |
| EDN3 | 3 | 0 | 0 | 0 | 0 | 0 | 0 | 0 | 1 |
| NUPL2 | 2 | 1 | 0 | 0 | 0 | 0 | 0 | 0 | 1 |
| PDE1B | 2 | 0 | 1 | 0 | 0 | 0 | 0 | 0 | 1 |
| IFNG | 3 | 0 | 0 | 0 | 0 | 0 | 0 | 0 | 1 |
| PARVG | 3 | 0 | 0 | 0 | 0 | 0 | 0 | 0 | 1 |
| HTR3E | 2 | 0 | 1 | 0 | 0 | 0 | 0 | 0 | 1 |
| GPX6 | 3 | 0 | 0 | 0 | 0 | 0 | 0 | 0 | 1 |
| TMED9 | 3 | 0 | 0 | 0 | 0 | 0 | 0 | 0 | 1 |
| BTG4 | 3 | 0 | 0 | 0 | 0 | 0 | 0 | 0 | 1 |
| GABRB1 | 2 | 1 | 0 | 0 | 0 | 0 | 0 | 0 | 1 |
| ANKRD29 | 3 | 0 | 0 | 0 | 0 | 0 | 0 | 0 | 1 |
| CCDC125 | 2 | 0 | 1 | 0 | 0 | 0 | 0 | 0 | 1 |
| EBLN2 | 3 | 0 | 0 | 0 | 0 | 0 | 0 | 0 | 1 |
| SEBOX | 3 | 0 | 0 | 0 | 0 | 0 | 0 | 0 | 1 |
| FEM1C | 2 | 1 | 0 | 0 | 0 | 0 | 0 | 0 | 1 |
| GIPC3 | 3 | 0 | 0 | 0 | 0 | 0 | 0 | 0 | 1 |
| VSTM1 | 3 | 0 | 0 | 0 | 0 | 0 | 0 | 0 | 1 |
| OR51I2 | 3 | 0 | 0 | 0 | 0 | 0 | 0 | 0 | 1 |

| | | | | | | | | | |
|---|---|---|---|---|---|---|---|---|---|
| ATXN1 | 2 | 1 | 0 | 0 | 0 | 0 | 0 | 0 | 1 |
| C16orf45 | 3 | 0 | 0 | 0 | 0 | 0 | 0 | 0 | 1 |
| FHL5 | 3 | 0 | 0 | 0 | 0 | 0 | 0 | 0 | 1 |
| LOC100287718 | 3 | 0 | 0 | 0 | 0 | 0 | 0 | 0 | 1 |
| ING4 | 3 | 0 | 0 | 0 | 0 | 0 | 0 | 0 | 1 |
| PEX11B | 3 | 0 | 0 | 0 | 0 | 0 | 0 | 0 | 1 |
| POLI | 2 | 1 | 0 | 0 | 0 | 0 | 0 | 0 | 1 |
| FAIM2 | 3 | 0 | 0 | 0 | 0 | 0 | 0 | 0 | 1 |
| ACSL4 | 2 | 1 | 0 | 0 | 0 | 0 | 0 | 0 | 1 |
| UBXN10 | 3 | 0 | 0 | 0 | 0 | 0 | 0 | 0 | 1 |
| ZNF750 | 2 | 1 | 0 | 0 | 0 | 0 | 0 | 0 | 1 |
| MORN3 | 3 | 0 | 0 | 0 | 0 | 0 | 0 | 0 | 1 |
| BTN2A1 | 2 | 1 | 0 | 0 | 0 | 0 | 0 | 0 | 1 |
| DLST | 3 | 0 | 0 | 0 | 0 | 0 | 0 | 0 | 1 |
| RGS7BP | 1 | 2 | 0 | 0 | 0 | 0 | 0 | 0 | 1 |
| CHIC1 | 3 | 0 | 0 | 0 | 0 | 0 | 0 | 0 | 1 |
| BEND4 | 2 | 1 | 0 | 0 | 0 | 0 | 0 | 0 | 1 |
| ZNF683 | 2 | 1 | 0 | 0 | 0 | 0 | 0 | 0 | 1 |
| GSG1L | 3 | 0 | 0 | 0 | 0 | 0 | 0 | 0 | 1 |
| ZNF641 | 2 | 1 | 0 | 0 | 0 | 0 | 0 | 0 | 1 |
| FBXL21 | 3 | 0 | 0 | 0 | 0 | 0 | 0 | 0 | 1 |
| FBXL20 | 2 | 1 | 0 | 0 | 0 | 0 | 0 | 0 | 1 |
| GPR119 | 3 | 0 | 0 | 0 | 0 | 0 | 0 | 0 | 1 |
| CAB39L | 3 | 0 | 0 | 0 | 0 | 0 | 0 | 0 | 1 |
| ALDOB | 2 | 0 | 1 | 0 | 0 | 0 | 0 | 0 | 1 |
| CREB1 | 3 | 0 | 0 | 0 | 0 | 0 | 0 | 0 | 1 |
| SEC23B | 1 | 1 | 1 | 0 | 0 | 0 | 0 | 0 | 1 |
| ZNF518A | 1 | 2 | 0 | 0 | 0 | 0 | 0 | 0 | 1 |
| ALOX15B | 2 | 1 | 0 | 0 | 0 | 0 | 0 | 0 | 1 |
| KPNA5 | 2 | 0 | 0 | 0 | 1 | 0 | 0 | 0 | 1 |
| TLR3 | 2 | 1 | 0 | 0 | 0 | 0 | 0 | 0 | 1 |
| TTLL9 | 2 | 0 | 1 | 0 | 0 | 0 | 0 | 0 | 1 |
| TGM7 | 2 | 1 | 0 | 0 | 0 | 0 | 0 | 0 | 1 |
| OR51B5 | 3 | 0 | 0 | 0 | 0 | 0 | 0 | 0 | 1 |
| PRL | 2 | 1 | 0 | 0 | 0 | 0 | 0 | 0 | 1 |
| RSPO1 | 3 | 0 | 0 | 0 | 0 | 0 | 0 | 0 | 1 |
| TNIP3 | 1 | 2 | 0 | 0 | 0 | 0 | 0 | 0 | 1 |
| ADCYAP1R1 | 2 | 1 | 0 | 0 | 0 | 0 | 0 | 0 | 1 |
| CRTC1 | 2 | 0 | 0 | 1 | 0 | 0 | 0 | 0 | 1 |
| GK2 | 2 | 1 | 0 | 0 | 0 | 0 | 0 | 0 | 1 |
| OR52D1 | 3 | 0 | 0 | 0 | 0 | 0 | 0 | 0 | 1 |
| STARD3NL | 2 | 1 | 0 | 0 | 0 | 0 | 0 | 0 | 1 |
| ZNF282 | 2 | 1 | 0 | 0 | 0 | 0 | 0 | 0 | 1 |
| ALG12 | 2 | 1 | 0 | 0 | 0 | 0 | 0 | 0 | 1 |
| TMEM196 | 2 | 0 | 1 | 0 | 0 | 0 | 0 | 0 | 1 |
| PTK6 | 2 | 1 | 0 | 0 | 0 | 0 | 0 | 0 | 1 |

| | | | | | | | | | |
|---|---|---|---|---|---|---|---|---|---|
| MC2R | 3 | 0 | 0 | 0 | 0 | 0 | 0 | 0 | 1 |
| SPDYA | 3 | 0 | 0 | 0 | 0 | 0 | 0 | 0 | 1 |
| ZBTB11 | 2 | 1 | 0 | 0 | 0 | 0 | 0 | 0 | 1 |
| DSN1 | 3 | 0 | 0 | 0 | 0 | 0 | 0 | 0 | 1 |
| OR5R1 | 3 | 0 | 0 | 0 | 0 | 0 | 0 | 0 | 1 |
| TFCP2 | 2 | 0 | 1 | 0 | 0 | 0 | 0 | 0 | 1 |
| MCOLN3 | 2 | 1 | 0 | 0 | 0 | 0 | 0 | 0 | 1 |
| KRT5 | 2 | 0 | 1 | 0 | 0 | 0 | 0 | 0 | 1 |
| C1orf158 | 3 | 0 | 0 | 0 | 0 | 0 | 0 | 0 | 1 |
| FGFR4 | 2 | 1 | 0 | 0 | 0 | 0 | 0 | 0 | 1 |
| ITGB2 | 2 | 1 | 0 | 0 | 0 | 0 | 0 | 0 | 1 |
| LARP7 | 2 | 1 | 0 | 0 | 0 | 0 | 0 | 0 | 1 |
| USP2 | 2 | 1 | 0 | 0 | 0 | 0 | 0 | 0 | 1 |
| DEGS2 | 3 | 0 | 0 | 0 | 0 | 0 | 0 | 0 | 1 |
| RPH3A | 1 | 2 | 0 | 0 | 0 | 0 | 0 | 0 | 1 |
| IQCH | 1 | 2 | 0 | 0 | 0 | 0 | 0 | 0 | 1 |
| ECHS1 | 3 | 0 | 0 | 0 | 0 | 0 | 0 | 0 | 1 |
| OR51B2 | 3 | 0 | 0 | 0 | 0 | 0 | 0 | 0 | 1 |
| C9orf96 | 2 | 0 | 1 | 0 | 0 | 0 | 0 | 0 | 1 |
| OSM | 3 | 0 | 0 | 0 | 0 | 0 | 0 | 0 | 1 |
| IRF3 | 3 | 0 | 0 | 0 | 0 | 0 | 0 | 0 | 1 |
| ASZ1 | 3 | 0 | 0 | 0 | 0 | 0 | 0 | 0 | 1 |
| PLOD1 | 2 | 1 | 0 | 0 | 0 | 0 | 0 | 0 | 1 |
| UNC45A | 1 | 1 | 0 | 1 | 0 | 0 | 0 | 0 | 1 |
| CPA2 | 3 | 0 | 0 | 0 | 0 | 0 | 0 | 0 | 1 |
| CSNK1G3 | 3 | 0 | 0 | 0 | 0 | 0 | 0 | 0 | 1 |
| GLRA3 | 2 | 1 | 0 | 0 | 0 | 0 | 0 | 0 | 1 |
| EIF3D | 2 | 1 | 0 | 0 | 0 | 0 | 0 | 0 | 1 |
| CAGE1 | 2 | 1 | 0 | 0 | 0 | 0 | 0 | 0 | 1 |
| PDIA5 | 2 | 1 | 0 | 0 | 0 | 0 | 0 | 0 | 1 |
| IPO13 | 2 | 0 | 1 | 0 | 0 | 0 | 0 | 0 | 1 |
| ZNF528 | 2 | 1 | 0 | 0 | 0 | 0 | 0 | 0 | 1 |
| ZNF385D | 2 | 1 | 0 | 0 | 0 | 0 | 0 | 0 | 1 |
| XKR9 | 3 | 0 | 0 | 0 | 0 | 0 | 0 | 0 | 1 |
| OR1L3 | 3 | 0 | 0 | 0 | 0 | 0 | 0 | 0 | 1 |
| AAMP | 3 | 0 | 0 | 0 | 0 | 0 | 0 | 0 | 1 |
| C17orf77 | 3 | 0 | 0 | 0 | 0 | 0 | 0 | 0 | 1 |
| EEF1G | 3 | 0 | 0 | 0 | 0 | 0 | 0 | 0 | 1 |
| CEACAM18 | 3 | 0 | 0 | 0 | 0 | 0 | 0 | 0 | 1 |
| ZFP1 | 3 | 0 | 0 | 0 | 0 | 0 | 0 | 0 | 1 |
| VPS33A | 3 | 0 | 0 | 0 | 0 | 0 | 0 | 0 | 1 |
| PMEPA1 | 3 | 0 | 0 | 0 | 0 | 0 | 0 | 0 | 1 |
| PTRH1 | 3 | 0 | 0 | 0 | 0 | 0 | 0 | 0 | 1 |
| FBP1 | 3 | 0 | 0 | 0 | 0 | 0 | 0 | 0 | 1 |
| RNF133 | 3 | 0 | 0 | 0 | 0 | 0 | 0 | 0 | 1 |
| SCLY | 3 | 0 | 0 | 0 | 0 | 0 | 0 | 0 | 1 |

| | | | | | | | | | |
|---|---|---|---|---|---|---|---|---|---|
| SNIP1 | 3 | 0 | 0 | 0 | 0 | 0 | 0 | 0 | 1 |
| IQCE | 2 | 1 | 0 | 0 | 0 | 0 | 0 | 0 | 1 |
| OR1N2 | 3 | 0 | 0 | 0 | 0 | 0 | 0 | 0 | 1 |
| TUBA8 | 3 | 0 | 0 | 0 | 0 | 0 | 0 | 0 | 1 |
| TNFSF13B | 3 | 0 | 0 | 0 | 0 | 0 | 0 | 0 | 1 |
| PRPF40B | 2 | 0 | 1 | 0 | 0 | 0 | 0 | 0 | 1 |
| YIPF7 | 3 | 0 | 0 | 0 | 0 | 0 | 0 | 0 | 1 |
| FADS6 | 3 | 0 | 0 | 0 | 0 | 0 | 0 | 0 | 1 |
| KCNK16 | 3 | 0 | 0 | 0 | 0 | 0 | 0 | 0 | 1 |
| PRMT3 | 2 | 0 | 0 | 0 | 1 | 0 | 0 | 0 | 1 |
| FAM58A | 3 | 0 | 0 | 0 | 0 | 0 | 0 | 0 | 1 |
| SLC13A3 | 2 | 1 | 0 | 0 | 0 | 0 | 0 | 0 | 1 |
| ABHD2 | 3 | 0 | 0 | 0 | 0 | 0 | 0 | 0 | 1 |
| SRL | 3 | 0 | 0 | 0 | 0 | 0 | 0 | 0 | 1 |
| RIOK3 | 3 | 0 | 0 | 0 | 0 | 0 | 0 | 0 | 1 |
| EPDR1 | 3 | 0 | 0 | 0 | 0 | 0 | 0 | 0 | 1 |
| CDKL4 | 3 | 0 | 0 | 0 | 0 | 0 | 0 | 0 | 1 |
| CUEDC2 | 3 | 0 | 0 | 0 | 0 | 0 | 0 | 0 | 1 |
| ATP6V1H | 3 | 0 | 0 | 0 | 0 | 0 | 0 | 0 | 1 |
| ANXA1 | 3 | 0 | 0 | 0 | 0 | 0 | 0 | 0 | 1 |
| SGPP1 | 3 | 0 | 0 | 0 | 0 | 0 | 0 | 0 | 1 |
| PNLDC1 | 3 | 0 | 0 | 0 | 0 | 0 | 0 | 0 | 1 |
| TBXAS1 | 2 | 1 | 0 | 0 | 0 | 0 | 0 | 0 | 1 |
| CACNG7 | 3 | 0 | 0 | 0 | 0 | 0 | 0 | 0 | 1 |
| CD300LF | 3 | 0 | 0 | 0 | 0 | 0 | 0 | 0 | 1 |
| KHSRP | 1 | 2 | 0 | 0 | 0 | 0 | 0 | 0 | 1 |
| ZNF645 | 3 | 0 | 0 | 0 | 0 | 0 | 0 | 0 | 1 |
| ZNF684 | 3 | 0 | 0 | 0 | 0 | 0 | 0 | 0 | 1 |
| SETD3 | 3 | 0 | 0 | 0 | 0 | 0 | 0 | 0 | 1 |
| VPS37C | 3 | 0 | 0 | 0 | 0 | 0 | 0 | 0 | 1 |
| OR5B21 | 3 | 0 | 0 | 0 | 0 | 0 | 0 | 0 | 1 |
| RNF113B | 3 | 0 | 0 | 0 | 0 | 0 | 0 | 0 | 1 |
| CLEC4E | 3 | 0 | 0 | 0 | 0 | 0 | 0 | 0 | 1 |
| LIN54 | 2 | 1 | 0 | 0 | 0 | 0 | 0 | 0 | 1 |
| FGGY | 2 | 0 | 1 | 0 | 0 | 0 | 0 | 0 | 1 |
| MMP13 | 3 | 0 | 0 | 0 | 0 | 0 | 0 | 0 | 1 |
| OR9Q1 | 3 | 0 | 0 | 0 | 0 | 0 | 0 | 0 | 1 |
| TARM1 | 3 | 0 | 0 | 0 | 0 | 0 | 0 | 0 | 1 |
| CCR3 | 3 | 0 | 0 | 0 | 0 | 0 | 0 | 0 | 1 |
| EZR | 2 | 1 | 0 | 0 | 0 | 0 | 0 | 0 | 1 |
| OR1Q1 | 3 | 0 | 0 | 0 | 0 | 0 | 0 | 0 | 1 |
| TIMD4 | 3 | 0 | 0 | 0 | 0 | 0 | 0 | 0 | 1 |
| LMOD3 | 2 | 0 | 0 | 1 | 0 | 0 | 0 | 0 | 1 |
| PGLYRP2 | 2 | 1 | 0 | 0 | 0 | 0 | 0 | 0 | 1 |
| PPM1N | 3 | 0 | 0 | 0 | 0 | 0 | 0 | 0 | 1 |
| TDRD12 | 3 | 0 | 0 | 0 | 0 | 0 | 0 | 0 | 1 |

| | | | | | | | | | |
|---|---|---|---|---|---|---|---|---|---|
| UVRAG | 2 | 1 | 0 | 0 | 0 | 0 | 0 | 0 | 1 |
| LIMS2 | 3 | 0 | 0 | 0 | 0 | 0 | 0 | 0 | 1 |
| SFRP4 | 2 | 1 | 0 | 0 | 0 | 0 | 0 | 0 | 1 |
| OR5B12 | 3 | 0 | 0 | 0 | 0 | 0 | 0 | 0 | 1 |
| ANO9 | 1 | 1 | 0 | 0 | 1 | 0 | 0 | 0 | 1 |
| OR51F2 | 3 | 0 | 0 | 0 | 0 | 0 | 0 | 0 | 1 |
| OR6C6 | 2 | 1 | 0 | 0 | 0 | 0 | 0 | 0 | 1 |
| DDI1 | 3 | 0 | 0 | 0 | 0 | 0 | 0 | 0 | 1 |
| AKAP8L | 2 | 0 | 1 | 0 | 0 | 0 | 0 | 0 | 1 |
| VEGFA | 3 | 0 | 0 | 0 | 0 | 0 | 0 | 0 | 1 |
| OR4K2 | 3 | 0 | 0 | 0 | 0 | 0 | 0 | 0 | 1 |
| PM20D2 | 3 | 0 | 0 | 0 | 0 | 0 | 0 | 0 | 1 |
| BZW2 | 3 | 0 | 0 | 0 | 0 | 0 | 0 | 0 | 1 |
| ETV4 | 2 | 1 | 0 | 0 | 0 | 0 | 0 | 0 | 1 |
| HTR5A | 3 | 0 | 0 | 0 | 0 | 0 | 0 | 0 | 1 |
| ADAMTSL2 | 2 | 1 | 0 | 0 | 0 | 0 | 0 | 0 | 1 |
| SNX2 | 3 | 0 | 0 | 0 | 0 | 0 | 0 | 0 | 1 |
| ZNF660 | 3 | 0 | 0 | 0 | 0 | 0 | 0 | 0 | 1 |
| SDR16C5 | 3 | 0 | 0 | 0 | 0 | 0 | 0 | 0 | 1 |
| YARS2 | 3 | 0 | 0 | 0 | 0 | 0 | 0 | 0 | 1 |
| C16orf42 | 3 | 0 | 0 | 0 | 0 | 0 | 0 | 0 | 1 |
| PDGFRL | 3 | 0 | 0 | 0 | 0 | 0 | 0 | 0 | 1 |
| NMT1 | 3 | 0 | 0 | 0 | 0 | 0 | 0 | 0 | 1 |
| HOMER1 | 3 | 0 | 0 | 0 | 0 | 0 | 0 | 0 | 1 |
| SH3TC2 | 1 | 2 | 0 | 0 | 0 | 0 | 0 | 0 | 1 |
| PAX6 | 2 | 1 | 0 | 0 | 0 | 0 | 0 | 0 | 1 |
| CHGB | 2 | 1 | 0 | 0 | 0 | 0 | 0 | 0 | 1 |
| SLC11A1 | 3 | 0 | 0 | 0 | 0 | 0 | 0 | 0 | 1 |
| GCNT1 | 3 | 0 | 0 | 0 | 0 | 0 | 0 | 0 | 1 |
| CCDC57 | 2 | 0 | 1 | 0 | 0 | 0 | 0 | 0 | 1 |
| KCNA1 | 2 | 0 | 0 | 0 | 1 | 0 | 0 | 0 | 1 |
| OR6C65 | 3 | 0 | 0 | 0 | 0 | 0 | 0 | 0 | 1 |
| MS4A4A | 3 | 0 | 0 | 0 | 0 | 0 | 0 | 0 | 1 |
| CPO | 3 | 0 | 0 | 0 | 0 | 0 | 0 | 0 | 1 |
| DIAPH3 | 1 | 1 | 1 | 0 | 0 | 0 | 0 | 0 | 1 |
| PMS1 | 2 | 1 | 0 | 0 | 0 | 0 | 0 | 0 | 1 |
| PI4KB | 2 | 1 | 0 | 0 | 0 | 0 | 0 | 0 | 1 |
| CD34 | 3 | 0 | 0 | 0 | 0 | 0 | 0 | 0 | 1 |
| SPOCK1 | 3 | 0 | 0 | 0 | 0 | 0 | 0 | 0 | 1 |
| FASTKD2 | 2 | 0 | 1 | 0 | 0 | 0 | 0 | 0 | 1 |
| PDE7B | 3 | 0 | 0 | 0 | 0 | 0 | 0 | 0 | 1 |
| TH1L | 2 | 1 | 0 | 0 | 0 | 0 | 0 | 0 | 1 |
| GPM6B | 3 | 0 | 0 | 0 | 0 | 0 | 0 | 0 | 1 |
| IMPDH1 | 2 | 0 | 0 | 0 | 1 | 0 | 0 | 0 | 1 |
| OR4K5 | 3 | 0 | 0 | 0 | 0 | 0 | 0 | 0 | 1 |
| KCNAB3 | 3 | 0 | 0 | 0 | 0 | 0 | 0 | 0 | 1 |

| | | | | | | | | | |
|---|---|---|---|---|---|---|---|---|---|
| PES1 | 2 | 1 | 0 | 0 | 0 | 0 | 0 | 0 | 1 |
| FAM46D | 2 | 1 | 0 | 0 | 0 | 0 | 0 | 0 | 1 |
| DAAM1 | 1 | 2 | 0 | 0 | 0 | 0 | 0 | 0 | 1 |
| CD46 | 3 | 0 | 0 | 0 | 0 | 0 | 0 | 0 | 1 |
| CCDC135 | 2 | 1 | 0 | 0 | 0 | 0 | 0 | 0 | 1 |
| WDR1 | 2 | 1 | 0 | 0 | 0 | 0 | 0 | 0 | 1 |
| NUPL1 | 3 | 0 | 0 | 0 | 0 | 0 | 0 | 0 | 1 |
| TRIM22 | 2 | 0 | 0 | 0 | 1 | 0 | 0 | 0 | 1 |
| DERA | 2 | 1 | 0 | 0 | 0 | 0 | 0 | 0 | 1 |
| GRK7 | 3 | 0 | 0 | 0 | 0 | 0 | 0 | 0 | 1 |
| PBX4 | 3 | 0 | 0 | 0 | 0 | 0 | 0 | 0 | 1 |
| LONRF2 | 2 | 1 | 0 | 0 | 0 | 0 | 0 | 0 | 1 |
| TM9SF1 | 3 | 0 | 0 | 0 | 0 | 0 | 0 | 0 | 1 |
| TH | 3 | 0 | 0 | 0 | 0 | 0 | 0 | 0 | 1 |
| OR6V1 | 3 | 0 | 0 | 0 | 0 | 0 | 0 | 0 | 1 |
| ST7L | 3 | 0 | 0 | 0 | 0 | 0 | 0 | 0 | 1 |
| SLC29A3 | 3 | 0 | 0 | 0 | 0 | 0 | 0 | 0 | 1 |
| ZNF391 | 3 | 0 | 0 | 0 | 0 | 0 | 0 | 0 | 1 |
| OR2AK2 | 3 | 0 | 0 | 0 | 0 | 0 | 0 | 0 | 1 |
| ENTPD7 | 3 | 0 | 0 | 0 | 0 | 0 | 0 | 0 | 1 |
| C2orf89 | 3 | 0 | 0 | 0 | 0 | 0 | 0 | 0 | 1 |
| PLTP | 3 | 0 | 0 | 0 | 0 | 0 | 0 | 0 | 1 |
| SYT7 | 3 | 0 | 0 | 0 | 0 | 0 | 0 | 0 | 1 |
| MSL3 | 2 | 0 | 0 | 0 | 1 | 0 | 0 | 0 | 1 |
| ATAD1 | 3 | 0 | 0 | 0 | 0 | 0 | 0 | 0 | 1 |
| C1QA | 3 | 0 | 0 | 0 | 0 | 0 | 0 | 0 | 1 |
| EYA4 | 2 | 1 | 0 | 0 | 0 | 0 | 0 | 0 | 1 |
| UBA3 | 2 | 1 | 0 | 0 | 0 | 0 | 0 | 0 | 1 |
| MEFV | 2 | 1 | 0 | 0 | 0 | 0 | 0 | 0 | 1 |
| TSHR | 2 | 1 | 0 | 0 | 0 | 0 | 0 | 0 | 1 |
| OR5D16 | 3 | 0 | 0 | 0 | 0 | 0 | 0 | 0 | 1 |
| LGI1 | 3 | 0 | 0 | 0 | 0 | 0 | 0 | 0 | 1 |
| EPN1 | 2 | 1 | 0 | 0 | 0 | 0 | 0 | 0 | 1 |
| IFI44L | 3 | 0 | 0 | 0 | 0 | 0 | 0 | 0 | 1 |
| AGT | 3 | 0 | 0 | 0 | 0 | 0 | 0 | 0 | 1 |
| CD300LG | 3 | 0 | 0 | 0 | 0 | 0 | 0 | 0 | 1 |
| MYBPH | 3 | 0 | 0 | 0 | 0 | 0 | 0 | 0 | 1 |
| EWSR1 | 3 | 0 | 0 | 0 | 0 | 0 | 0 | 0 | 1 |
| SCCPDH | 2 | 1 | 0 | 0 | 0 | 0 | 0 | 0 | 1 |
| CCDC63 | 3 | 0 | 0 | 0 | 0 | 0 | 0 | 0 | 1 |
| RGL3 | 2 | 1 | 0 | 0 | 0 | 0 | 0 | 0 | 1 |
| RBMS2 | 3 | 0 | 0 | 0 | 0 | 0 | 0 | 0 | 1 |
| HOXD10 | 3 | 0 | 0 | 0 | 0 | 0 | 0 | 0 | 1 |
| USP3 | 3 | 0 | 0 | 0 | 0 | 0 | 0 | 0 | 1 |
| GSG1 | 3 | 0 | 0 | 0 | 0 | 0 | 0 | 0 | 1 |
| SASH3 | 3 | 0 | 0 | 0 | 0 | 0 | 0 | 0 | 1 |

| | | | | | | | | | |
|---|---|---|---|---|---|---|---|---|---|
| DOK2 | 2 | 0 | 0 | 0 | 1 | 0 | 0 | 0 | 1 |
| MLF1IP | 3 | 0 | 0 | 0 | 0 | 0 | 0 | 0 | 1 |
| FAM200A | 3 | 0 | 0 | 0 | 0 | 0 | 0 | 0 | 1 |
| CYP26A1 | 3 | 0 | 0 | 0 | 0 | 0 | 0 | 0 | 1 |
| MAGEB10 | 3 | 0 | 0 | 0 | 0 | 0 | 0 | 0 | 1 |
| ISPD | 2 | 1 | 0 | 0 | 0 | 0 | 0 | 0 | 1 |
| COPS2 | 3 | 0 | 0 | 0 | 0 | 0 | 0 | 0 | 1 |
| CCNA2 | 3 | 0 | 0 | 0 | 0 | 0 | 0 | 0 | 1 |
| RNF123 | 2 | 1 | 0 | 0 | 0 | 0 | 0 | 0 | 1 |
| HDGFRP2 | 2 | 0 | 0 | 0 | 1 | 0 | 0 | 0 | 1 |
| PROX2 | 3 | 0 | 0 | 0 | 0 | 0 | 0 | 0 | 1 |
| ZNF114 | 3 | 0 | 0 | 0 | 0 | 0 | 0 | 0 | 1 |
| SLC1A2 | 3 | 0 | 0 | 0 | 0 | 0 | 0 | 0 | 1 |
| TEAD4 | 3 | 0 | 0 | 0 | 0 | 0 | 0 | 0 | 1 |
| COG4 | 3 | 0 | 0 | 0 | 0 | 0 | 0 | 0 | 1 |
| PPIG | 2 | 1 | 0 | 0 | 0 | 0 | 0 | 0 | 1 |
| RSL1D1 | 3 | 0 | 0 | 0 | 0 | 0 | 0 | 0 | 1 |
| GLYR1 | 3 | 0 | 0 | 0 | 0 | 0 | 0 | 0 | 1 |
| CEP63 | 2 | 1 | 0 | 0 | 0 | 0 | 0 | 0 | 1 |
| CEP72 | 2 | 1 | 0 | 0 | 0 | 0 | 0 | 0 | 1 |
| OR10H3 | 3 | 0 | 0 | 0 | 0 | 0 | 0 | 0 | 1 |
| CD84 | 3 | 0 | 0 | 0 | 0 | 0 | 0 | 0 | 1 |
| ZNF230 | 2 | 1 | 0 | 0 | 0 | 0 | 0 | 0 | 1 |
| GSTA4 | 2 | 1 | 0 | 0 | 0 | 0 | 0 | 0 | 1 |
| DENND2C | 2 | 1 | 0 | 0 | 0 | 0 | 0 | 0 | 1 |
| DENND1C | 3 | 0 | 0 | 0 | 0 | 0 | 0 | 0 | 1 |
| NLRC4 | 2 | 1 | 0 | 0 | 0 | 0 | 0 | 0 | 1 |
| RBM11 | 3 | 0 | 0 | 0 | 0 | 0 | 0 | 0 | 1 |
| OR5AC2 | 3 | 0 | 0 | 0 | 0 | 0 | 0 | 0 | 1 |
| LEF1 | 3 | 0 | 0 | 0 | 0 | 0 | 0 | 0 | 1 |
| ZNF557 | 3 | 0 | 0 | 0 | 0 | 0 | 0 | 0 | 1 |
| ATF6B | 2 | 1 | 0 | 0 | 0 | 0 | 0 | 0 | 1 |
| TEX101 | 3 | 0 | 0 | 0 | 0 | 0 | 0 | 0 | 1 |
| LGALS12 | 3 | 0 | 0 | 0 | 0 | 0 | 0 | 0 | 1 |
| NPR3 | 3 | 0 | 0 | 0 | 0 | 0 | 0 | 0 | 1 |
| MCTP1 | 2 | 1 | 0 | 0 | 0 | 0 | 0 | 0 | 1 |
| ZNF167 | 2 | 1 | 0 | 0 | 0 | 0 | 0 | 0 | 1 |
| BCAR3 | 3 | 0 | 0 | 0 | 0 | 0 | 0 | 0 | 1 |
| CCDC14 | 1 | 2 | 0 | 0 | 0 | 0 | 0 | 0 | 1 |
| KRT78 | 3 | 0 | 0 | 0 | 0 | 0 | 0 | 0 | 1 |
| EPHA1 | 2 | 1 | 0 | 0 | 0 | 0 | 0 | 0 | 1 |
| BCL6 | 3 | 0 | 0 | 0 | 0 | 0 | 0 | 0 | 1 |
| TARS | 2 | 0 | 0 | 0 | 0 | 0 | 1 | 0 | 1 |
| RTN2 | 3 | 0 | 0 | 0 | 0 | 0 | 0 | 0 | 1 |
| VPS54 | 2 | 0 | 1 | 0 | 0 | 0 | 0 | 0 | 1 |
| ETS1 | 3 | 0 | 0 | 0 | 0 | 0 | 0 | 0 | 1 |

| | | | | | | | | | |
|---|---|---|---|---|---|---|---|---|---|
| ETV3 | 2 | 1 | 0 | 0 | 0 | 0 | 0 | 0 | 1 |
| CDX4 | 3 | 0 | 0 | 0 | 0 | 0 | 0 | 0 | 1 |
| SEPP1 | 3 | 0 | 0 | 0 | 0 | 0 | 0 | 0 | 1 |
| NTN4 | 3 | 0 | 0 | 0 | 0 | 0 | 0 | 0 | 1 |
| HSD3B2 | 3 | 0 | 0 | 0 | 0 | 0 | 0 | 0 | 1 |
| MS4A8B | 3 | 0 | 0 | 0 | 0 | 0 | 0 | 0 | 1 |
| RALGPS2 | 3 | 0 | 0 | 0 | 0 | 0 | 0 | 0 | 1 |
| CD200R1L | 3 | 0 | 0 | 0 | 0 | 0 | 0 | 0 | 1 |
| PTGFR | 3 | 0 | 0 | 0 | 0 | 0 | 0 | 0 | 1 |
| GLT25D1 | 3 | 0 | 0 | 0 | 0 | 0 | 0 | 0 | 1 |
| ZNF610 | 3 | 0 | 0 | 0 | 0 | 0 | 0 | 0 | 1 |
| PLCB3 | 2 | 1 | 0 | 0 | 0 | 0 | 0 | 0 | 1 |
| NUSAP1 | 3 | 0 | 0 | 0 | 0 | 0 | 0 | 0 | 1 |
| PDIA2 | 3 | 0 | 0 | 0 | 0 | 0 | 0 | 0 | 1 |
| THPO | 3 | 0 | 0 | 0 | 0 | 0 | 0 | 0 | 1 |
| SCP2 | 3 | 0 | 0 | 0 | 0 | 0 | 0 | 0 | 1 |
| SQRDL | 3 | 0 | 0 | 0 | 0 | 0 | 0 | 0 | 1 |
| CACNG3 | 3 | 0 | 0 | 0 | 0 | 0 | 0 | 0 | 1 |
| EFR3A | 2 | 1 | 0 | 0 | 0 | 0 | 0 | 0 | 1 |
| SLC30A3 | 3 | 0 | 0 | 0 | 0 | 0 | 0 | 0 | 1 |
| ZNF484 | 3 | 0 | 0 | 0 | 0 | 0 | 0 | 0 | 1 |
| ODAM | 3 | 0 | 0 | 0 | 0 | 0 | 0 | 0 | 1 |
| ZNF627 | 3 | 0 | 0 | 0 | 0 | 0 | 0 | 0 | 1 |
| PRKCI | 2 | 0 | 0 | 0 | 1 | 0 | 0 | 0 | 1 |
| CAPN2 | 2 | 1 | 0 | 0 | 0 | 0 | 0 | 0 | 1 |
| B4GALNT1 | 3 | 0 | 0 | 0 | 0 | 0 | 0 | 0 | 1 |
| SOX10 | 3 | 0 | 0 | 0 | 0 | 0 | 0 | 0 | 1 |
| TBX19 | 2 | 1 | 0 | 0 | 0 | 0 | 0 | 0 | 1 |
| SGK1 | 2 | 1 | 0 | 0 | 0 | 0 | 0 | 0 | 1 |
| DYNC1I1 | 2 | 1 | 0 | 0 | 0 | 0 | 0 | 0 | 1 |
| PTPN6 | 3 | 0 | 0 | 0 | 0 | 0 | 0 | 0 | 1 |
| HNF1A | 3 | 0 | 0 | 0 | 0 | 0 | 0 | 0 | 1 |
| ZNF277 | 3 | 0 | 0 | 0 | 0 | 0 | 0 | 0 | 1 |
| KPNA1 | 3 | 0 | 0 | 0 | 0 | 0 | 0 | 0 | 1 |
| MCTP2 | 1 | 1 | 1 | 0 | 0 | 0 | 0 | 0 | 1 |
| EDEM2 | 3 | 0 | 0 | 0 | 0 | 0 | 0 | 0 | 1 |
| SLC5A12 | 2 | 1 | 0 | 0 | 0 | 0 | 0 | 0 | 1 |
| ZNF689 | 3 | 0 | 0 | 0 | 0 | 0 | 0 | 0 | 1 |
| SLC17A1 | 3 | 0 | 0 | 0 | 0 | 0 | 0 | 0 | 1 |
| UBC | 3 | 0 | 0 | 0 | 0 | 0 | 0 | 0 | 1 |
| GJA8 | 3 | 0 | 0 | 0 | 0 | 0 | 0 | 0 | 1 |
| TRIM67 | 2 | 0 | 1 | 0 | 0 | 0 | 0 | 0 | 1 |
| KCNA5 | 3 | 0 | 0 | 0 | 0 | 0 | 0 | 0 | 1 |
| HTR2C | 3 | 0 | 0 | 0 | 0 | 0 | 0 | 0 | 1 |
| OAS3 | 1 | 1 | 0 | 0 | 1 | 0 | 0 | 0 | 1 |
| BRE | 3 | 0 | 0 | 0 | 0 | 0 | 0 | 0 | 1 |

| | | | | | | | | | |
|---|---|---|---|---|---|---|---|---|---|
| KRT37 | 3 | 0 | 0 | 0 | 0 | 0 | 0 | 0 | 1 |
| CACNB4 | 3 | 0 | 0 | 0 | 0 | 0 | 0 | 0 | 1 |
| AMBP | 3 | 0 | 0 | 0 | 0 | 0 | 0 | 0 | 1 |
| ADD1 | 3 | 0 | 0 | 0 | 0 | 0 | 0 | 0 | 1 |
| OR7D2 | 3 | 0 | 0 | 0 | 0 | 0 | 0 | 0 | 1 |
| ALG11 | 3 | 0 | 0 | 0 | 0 | 0 | 0 | 0 | 1 |
| GCM2 | 3 | 0 | 0 | 0 | 0 | 0 | 0 | 0 | 1 |
| AKR1C4 | 3 | 0 | 0 | 0 | 0 | 0 | 0 | 0 | 1 |
| TOR1AIP1 | 3 | 0 | 0 | 0 | 0 | 0 | 0 | 0 | 1 |
| RANBP3L | 3 | 0 | 0 | 0 | 0 | 0 | 0 | 0 | 1 |
| FRMD1 | 2 | 1 | 0 | 0 | 0 | 0 | 0 | 0 | 1 |
| PDHA1 | 3 | 0 | 0 | 0 | 0 | 0 | 0 | 0 | 1 |
| H1FOO | 3 | 0 | 0 | 0 | 0 | 0 | 0 | 0 | 1 |
| ICAM1 | 3 | 0 | 0 | 0 | 0 | 0 | 0 | 0 | 1 |
| KIAA1377 | 2 | 1 | 0 | 0 | 0 | 0 | 0 | 0 | 1 |
| SLC2A12 | 3 | 0 | 0 | 0 | 0 | 0 | 0 | 0 | 1 |
| UNC93A | 3 | 0 | 0 | 0 | 0 | 0 | 0 | 0 | 1 |
| TEKT5 | 3 | 0 | 0 | 0 | 0 | 0 | 0 | 0 | 1 |
| STAMBP | 3 | 0 | 0 | 0 | 0 | 0 | 0 | 0 | 1 |
| DCDC2 | 2 | 1 | 0 | 0 | 0 | 0 | 0 | 0 | 1 |
| RRP12 | 2 | 1 | 0 | 0 | 0 | 0 | 0 | 0 | 1 |
| ZNF480 | 3 | 0 | 0 | 0 | 0 | 0 | 0 | 0 | 1 |
| CCDC148 | 3 | 0 | 0 | 0 | 0 | 0 | 0 | 0 | 1 |
| CASP4 | 3 | 0 | 0 | 0 | 0 | 0 | 0 | 0 | 1 |
| OR6K2 | 3 | 0 | 0 | 0 | 0 | 0 | 0 | 0 | 1 |
| CCDC157 | 2 | 0 | 0 | 0 | 1 | 0 | 0 | 0 | 1 |
| RAD21L1 | 3 | 0 | 0 | 0 | 0 | 0 | 0 | 0 | 1 |
| MTNR1A | 3 | 0 | 0 | 0 | 0 | 0 | 0 | 0 | 1 |
| SLC22A10 | 3 | 0 | 0 | 0 | 0 | 0 | 0 | 0 | 1 |
| MYOC | 3 | 0 | 0 | 0 | 0 | 0 | 0 | 0 | 1 |
| RCSD1 | 3 | 0 | 0 | 0 | 0 | 0 | 0 | 0 | 1 |
| GABRQ | 3 | 0 | 0 | 0 | 0 | 0 | 0 | 0 | 1 |
| HP1BP3 | 3 | 0 | 0 | 0 | 0 | 0 | 0 | 0 | 1 |
| SUN5 | 3 | 0 | 0 | 0 | 0 | 0 | 0 | 0 | 1 |
| CLIC6 | 2 | 0 | 0 | 0 | 1 | 0 | 0 | 0 | 1 |
| EPS15 | 3 | 0 | 0 | 0 | 0 | 0 | 0 | 0 | 1 |
| SEMA4F | 2 | 1 | 0 | 0 | 0 | 0 | 0 | 0 | 1 |
| OR6C70 | 3 | 0 | 0 | 0 | 0 | 0 | 0 | 0 | 1 |
| ADHFE1 | 3 | 0 | 0 | 0 | 0 | 0 | 0 | 0 | 1 |
| SLC24A1 | 3 | 0 | 0 | 0 | 0 | 0 | 0 | 0 | 1 |
| OR9A4 | 3 | 0 | 0 | 0 | 0 | 0 | 0 | 0 | 1 |
| SMOX | 3 | 0 | 0 | 0 | 0 | 0 | 0 | 0 | 1 |
| CD19 | 3 | 0 | 0 | 0 | 0 | 0 | 0 | 0 | 1 |
| TRIML2 | 3 | 0 | 0 | 0 | 0 | 0 | 0 | 0 | 1 |
| SUPT5H | 2 | 1 | 0 | 0 | 0 | 0 | 0 | 0 | 1 |
| PFAS | 1 | 2 | 0 | 0 | 0 | 0 | 0 | 0 | 1 |

| | | | | | | | | | |
|---|---|---|---|---|---|---|---|---|---|
| OR4L1 | 3 | 0 | 0 | 0 | 0 | 0 | 0 | 0 | 1 |
| C3orf30 | 3 | 0 | 0 | 0 | 0 | 0 | 0 | 0 | 1 |
| OR6Y1 | 3 | 0 | 0 | 0 | 0 | 0 | 0 | 0 | 1 |
| SRP72 | 3 | 0 | 0 | 0 | 0 | 0 | 0 | 0 | 1 |
| MLLT3 | 3 | 0 | 0 | 0 | 0 | 0 | 0 | 0 | 1 |
| CADM3 | 3 | 0 | 0 | 0 | 0 | 0 | 0 | 0 | 1 |
| HSPA4 | 3 | 0 | 0 | 0 | 0 | 0 | 0 | 0 | 1 |
| ABCG4 | 3 | 0 | 0 | 0 | 0 | 0 | 0 | 0 | 1 |
| PRMT8 | 3 | 0 | 0 | 0 | 0 | 0 | 0 | 0 | 1 |
| SCLT1 | 2 | 1 | 0 | 0 | 0 | 0 | 0 | 0 | 1 |
| GGA3 | 3 | 0 | 0 | 0 | 0 | 0 | 0 | 0 | 1 |
| CAPN11 | 2 | 0 | 0 | 0 | 1 | 0 | 0 | 0 | 1 |
| STOX1 | 3 | 0 | 0 | 0 | 0 | 0 | 0 | 0 | 1 |
| ZNF280C | 3 | 0 | 0 | 0 | 0 | 0 | 0 | 0 | 1 |
| CPSF6 | 3 | 0 | 0 | 0 | 0 | 0 | 0 | 0 | 1 |
| DES | 3 | 0 | 0 | 0 | 0 | 0 | 0 | 0 | 1 |
| ZNF333 | 2 | 1 | 0 | 0 | 0 | 0 | 0 | 0 | 1 |
| DYM | 3 | 0 | 0 | 0 | 0 | 0 | 0 | 0 | 1 |
| SRBD1 | 2 | 0 | 0 | 0 | 1 | 0 | 0 | 0 | 1 |
| NONO | 3 | 0 | 0 | 0 | 0 | 0 | 0 | 0 | 1 |
| AKNAD1 | 3 | 0 | 0 | 0 | 0 | 0 | 0 | 0 | 1 |
| HSPA5 | 3 | 0 | 0 | 0 | 0 | 0 | 0 | 0 | 1 |
| KRT72 | 3 | 0 | 0 | 0 | 0 | 0 | 0 | 0 | 1 |
| KBTBD8 | 2 | 0 | 0 | 0 | 1 | 0 | 0 | 0 | 1 |
| SLC18A1 | 3 | 0 | 0 | 0 | 0 | 0 | 0 | 0 | 1 |
| FBXL4 | 3 | 0 | 0 | 0 | 0 | 0 | 0 | 0 | 1 |
| RHOT1 | 3 | 0 | 0 | 0 | 0 | 0 | 0 | 0 | 1 |
| PRIM2 | 3 | 0 | 0 | 0 | 0 | 0 | 0 | 0 | 1 |
| FAAH | 3 | 0 | 0 | 0 | 0 | 0 | 0 | 0 | 1 |
| ZNF823 | 3 | 0 | 0 | 0 | 0 | 0 | 0 | 0 | 1 |
| WWP1 | 2 | 1 | 0 | 0 | 0 | 0 | 0 | 0 | 1 |
| CCDC37 | 3 | 0 | 0 | 0 | 0 | 0 | 0 | 0 | 1 |
| TCTN1 | 3 | 0 | 0 | 0 | 0 | 0 | 0 | 0 | 1 |
| ATP2A1 | 3 | 0 | 0 | 0 | 0 | 0 | 0 | 0 | 1 |
| FBXO41 | 3 | 0 | 0 | 0 | 0 | 0 | 0 | 0 | 1 |
| SLC27A4 | 3 | 0 | 0 | 0 | 0 | 0 | 0 | 0 | 1 |
| ANGPT2 | 3 | 0 | 0 | 0 | 0 | 0 | 0 | 0 | 1 |
| SIAE | 2 | 0 | 0 | 0 | 1 | 0 | 0 | 0 | 1 |
| SLC16A9 | 3 | 0 | 0 | 0 | 0 | 0 | 0 | 0 | 1 |
| ODF2 | 3 | 0 | 0 | 0 | 0 | 0 | 0 | 0 | 1 |
| ZNF695 | 3 | 0 | 0 | 0 | 0 | 0 | 0 | 0 | 1 |
| TTC24 | 3 | 0 | 0 | 0 | 0 | 0 | 0 | 0 | 1 |
| ZNF425 | 2 | 1 | 0 | 0 | 0 | 0 | 0 | 0 | 1 |
| CRAT | 3 | 0 | 0 | 0 | 0 | 0 | 0 | 0 | 1 |
| ZDHHC17 | 3 | 0 | 0 | 0 | 0 | 0 | 0 | 0 | 1 |
| MS4A14 | 2 | 1 | 0 | 0 | 0 | 0 | 0 | 0 | 1 |

| | | | | | | | | | |
|---|---|---|---|---|---|---|---|---|---|
| SIGLEC6 | 2 | 1 | 0 | 0 | 0 | 0 | 0 | 0 | 1 |
| ZFYVE20 | 3 | 0 | 0 | 0 | 0 | 0 | 0 | 0 | 1 |
| ZNF34 | 3 | 0 | 0 | 0 | 0 | 0 | 0 | 0 | 1 |
| DAXX | 3 | 0 | 0 | 0 | 0 | 0 | 0 | 0 | 1 |
| SNTB1 | 3 | 0 | 0 | 0 | 0 | 0 | 0 | 0 | 1 |
| PTPN21 | 2 | 1 | 0 | 0 | 0 | 0 | 0 | 0 | 1 |
| RASGRP4 | 3 | 0 | 0 | 0 | 0 | 0 | 0 | 0 | 1 |
| LCA5 | 3 | 0 | 0 | 0 | 0 | 0 | 0 | 0 | 1 |
| CD53 | 2 | 1 | 0 | 0 | 0 | 0 | 0 | 0 | 1 |
| DNPEP | 3 | 0 | 0 | 0 | 0 | 0 | 0 | 0 | 1 |
| ACSBG2 | 3 | 0 | 0 | 0 | 0 | 0 | 0 | 0 | 1 |
| ANKRD34C | 3 | 0 | 0 | 0 | 0 | 0 | 0 | 0 | 1 |
| DEAF1 | 3 | 0 | 0 | 0 | 0 | 0 | 0 | 0 | 1 |
| FERMT1 | 3 | 0 | 0 | 0 | 0 | 0 | 0 | 0 | 1 |
| PPP1R13L | 2 | 0 | 0 | 0 | 1 | 0 | 0 | 0 | 1 |
| TPRX1 | 3 | 0 | 0 | 0 | 0 | 0 | 0 | 0 | 1 |
| IL31RA | 2 | 0 | 0 | 0 | 1 | 0 | 0 | 0 | 1 |
| HEPH | 1 | 2 | 0 | 0 | 0 | 0 | 0 | 0 | 1 |
| B3GALT5 | 3 | 0 | 0 | 0 | 0 | 0 | 0 | 0 | 1 |
| SYNCRIP | 3 | 0 | 0 | 0 | 0 | 0 | 0 | 0 | 1 |
| CCDC61 | 3 | 0 | 0 | 0 | 0 | 0 | 0 | 0 | 1 |
| TMEM132E | 2 | 1 | 0 | 0 | 0 | 0 | 0 | 0 | 1 |
| GDF2 | 3 | 0 | 0 | 0 | 0 | 0 | 0 | 0 | 1 |
| BRAF | 3 | 0 | 0 | 0 | 0 | 0 | 0 | 0 | 1 |
| SYTL5 | 3 | 0 | 0 | 0 | 0 | 0 | 0 | 0 | 1 |
| RNF157 | 3 | 0 | 0 | 0 | 0 | 0 | 0 | 0 | 1 |
| CREB3L2 | 3 | 0 | 0 | 0 | 0 | 0 | 0 | 0 | 1 |
| MGAT4C | 3 | 0 | 0 | 0 | 0 | 0 | 0 | 0 | 1 |
| AGPAT5 | 3 | 0 | 0 | 0 | 0 | 0 | 0 | 0 | 1 |
| SLC6A19 | 3 | 0 | 0 | 0 | 0 | 0 | 0 | 0 | 1 |
| SPP1 | 3 | 0 | 0 | 0 | 0 | 0 | 0 | 0 | 1 |
| CNOT2 | 3 | 0 | 0 | 0 | 0 | 0 | 0 | 0 | 1 |
| PABPC1L | 3 | 0 | 0 | 0 | 0 | 0 | 0 | 0 | 1 |
| BFSP1 | 3 | 0 | 0 | 0 | 0 | 0 | 0 | 0 | 1 |
| TFRC | 3 | 0 | 0 | 0 | 0 | 0 | 0 | 0 | 1 |
| DMPK | 3 | 0 | 0 | 0 | 0 | 0 | 0 | 0 | 1 |
| USP5 | 3 | 0 | 0 | 0 | 0 | 0 | 0 | 0 | 1 |
| DOK6 | 3 | 0 | 0 | 0 | 0 | 0 | 0 | 0 | 1 |
| TESK2 | 3 | 0 | 0 | 0 | 0 | 0 | 0 | 0 | 1 |
| HFE2 | 3 | 0 | 0 | 0 | 0 | 0 | 0 | 0 | 1 |
| FAM63A | 3 | 0 | 0 | 0 | 0 | 0 | 0 | 0 | 1 |
| DYRK1A | 3 | 0 | 0 | 0 | 0 | 0 | 0 | 0 | 1 |
| TAX1BP1 | 2 | 1 | 0 | 0 | 0 | 0 | 0 | 0 | 1 |
| MMP10 | 3 | 0 | 0 | 0 | 0 | 0 | 0 | 0 | 1 |
| ZNF148 | 3 | 0 | 0 | 0 | 0 | 0 | 0 | 0 | 1 |
| PPEF2 | 3 | 0 | 0 | 0 | 0 | 0 | 0 | 0 | 1 |

| | | | | | | | | | |
|---|---|---|---|---|---|---|---|---|---|
| HKDC1 | 3 | 0 | 0 | 0 | 0 | 0 | 0 | 0 | 1 |
| OR10J1 | 3 | 0 | 0 | 0 | 0 | 0 | 0 | 0 | 1 |
| GABRA4 | 3 | 0 | 0 | 0 | 0 | 0 | 0 | 0 | 1 |
| SLC6A11 | 3 | 0 | 0 | 0 | 0 | 0 | 0 | 0 | 1 |
| KRT3 | 3 | 0 | 0 | 0 | 0 | 0 | 0 | 0 | 1 |
| SLC5A11 | 3 | 0 | 0 | 0 | 0 | 0 | 0 | 0 | 1 |
| OR56A4 | 3 | 0 | 0 | 0 | 0 | 0 | 0 | 0 | 1 |
| THAP5 | 3 | 0 | 0 | 0 | 0 | 0 | 0 | 0 | 1 |
| MS4A2 | 3 | 0 | 0 | 0 | 0 | 0 | 0 | 0 | 1 |
| UNCX | 3 | 0 | 0 | 0 | 0 | 0 | 0 | 0 | 1 |
| C16orf58 | 3 | 0 | 0 | 0 | 0 | 0 | 0 | 0 | 1 |
| ZNF721 | 3 | 0 | 0 | 0 | 0 | 0 | 0 | 0 | 1 |
| PBXIP1 | 2 | 1 | 0 | 0 | 0 | 0 | 0 | 0 | 1 |
| NBN | 3 | 0 | 0 | 0 | 0 | 0 | 0 | 0 | 1 |
| LRRC49 | 3 | 0 | 0 | 0 | 0 | 0 | 0 | 0 | 1 |
| AFAP1 | 3 | 0 | 0 | 0 | 0 | 0 | 0 | 0 | 1 |
| PLSCR2 | 3 | 0 | 0 | 0 | 0 | 0 | 0 | 0 | 1 |
| TGFBR3 | 3 | 0 | 0 | 0 | 0 | 0 | 0 | 0 | 1 |
| PLA2G4D | 3 | 0 | 0 | 0 | 0 | 0 | 0 | 0 | 1 |
| SLC7A2 | 3 | 0 | 0 | 0 | 0 | 0 | 0 | 0 | 1 |
| CNDP2 | 3 | 0 | 0 | 0 | 0 | 0 | 0 | 0 | 1 |
| PRPF39 | 3 | 0 | 0 | 0 | 0 | 0 | 0 | 0 | 1 |
| PHC3 | 2 | 0 | 0 | 0 | 1 | 0 | 0 | 0 | 1 |
| TREH | 3 | 0 | 0 | 0 | 0 | 0 | 0 | 0 | 1 |
| CYP4F8 | 3 | 0 | 0 | 0 | 0 | 0 | 0 | 0 | 1 |
| MAD1L1 | 3 | 0 | 0 | 0 | 0 | 0 | 0 | 0 | 1 |
| ABLIM3 | 3 | 0 | 0 | 0 | 0 | 0 | 0 | 0 | 1 |
| EBF1 | 3 | 0 | 0 | 0 | 0 | 0 | 0 | 0 | 1 |
| LOC81691 | 3 | 0 | 0 | 0 | 0 | 0 | 0 | 0 | 1 |
| SIN3B | 2 | 1 | 0 | 0 | 0 | 0 | 0 | 0 | 1 |
| OR2C3 | 3 | 0 | 0 | 0 | 0 | 0 | 0 | 0 | 1 |
| OPCML | 3 | 0 | 0 | 0 | 0 | 0 | 0 | 0 | 1 |
| PPHLN1 | 3 | 0 | 0 | 0 | 0 | 0 | 0 | 0 | 1 |
| MKRN3 | 3 | 0 | 0 | 0 | 0 | 0 | 0 | 0 | 1 |
| USP44 | 3 | 0 | 0 | 0 | 0 | 0 | 0 | 0 | 1 |
| REG1B | 3 | 0 | 0 | 0 | 0 | 0 | 0 | 0 | 1 |
| DDOST | 3 | 0 | 0 | 0 | 0 | 0 | 0 | 0 | 1 |
| MUC7 | 3 | 0 | 0 | 0 | 0 | 0 | 0 | 0 | 1 |
| CSN2 | 3 | 0 | 0 | 0 | 0 | 0 | 0 | 0 | 1 |
| ZNF490 | 3 | 0 | 0 | 0 | 0 | 0 | 0 | 0 | 1 |
| DMRTC2 | 3 | 0 | 0 | 0 | 0 | 0 | 0 | 0 | 1 |
| SMAD9 | 3 | 0 | 0 | 0 | 0 | 0 | 0 | 0 | 1 |
| CAPN14 | 3 | 0 | 0 | 0 | 0 | 0 | 0 | 0 | 1 |
| SMARCC2 | 2 | 1 | 0 | 0 | 0 | 0 | 0 | 0 | 1 |
| REXO1 | 2 | 1 | 0 | 0 | 0 | 0 | 0 | 0 | 1 |
| ESAM | 3 | 0 | 0 | 0 | 0 | 0 | 0 | 0 | 1 |

| | | | | | | | | | |
|---|---|---|---|---|---|---|---|---|---|
| FBXO30 | 3 | 0 | 0 | 0 | 0 | 0 | 0 | 0 | 1 |
| ZNF227 | 2 | 1 | 0 | 0 | 0 | 0 | 0 | 0 | 1 |
| CAPRIN1 | 3 | 0 | 0 | 0 | 0 | 0 | 0 | 0 | 1 |
| ANKRD13B | 3 | 0 | 0 | 0 | 0 | 0 | 0 | 0 | 1 |
| PAM | 2 | 0 | 1 | 0 | 0 | 0 | 0 | 0 | 1 |
| OR4C6 | 3 | 0 | 0 | 0 | 0 | 0 | 0 | 0 | 1 |
| OR10X1 | 3 | 0 | 0 | 0 | 0 | 0 | 0 | 0 | 1 |
| ZSCAN4 | 3 | 0 | 0 | 0 | 0 | 0 | 0 | 0 | 1 |
| SP2 | 3 | 0 | 0 | 0 | 0 | 0 | 0 | 0 | 1 |
| KCNIP1 | 3 | 0 | 0 | 0 | 0 | 0 | 0 | 0 | 1 |
| RASAL1 | 2 | 1 | 0 | 0 | 0 | 0 | 0 | 0 | 1 |
| SCG2 | 3 | 0 | 0 | 0 | 0 | 0 | 0 | 0 | 1 |
| ZNF529 | 3 | 0 | 0 | 0 | 0 | 0 | 0 | 0 | 1 |
| C17orf70 | 3 | 0 | 0 | 0 | 0 | 0 | 0 | 0 | 1 |
| SLC22A25 | 3 | 0 | 0 | 0 | 0 | 0 | 0 | 0 | 1 |
| MAGEA8 | 2 | 1 | 0 | 0 | 0 | 0 | 0 | 0 | 1 |
| SLC27A3 | 3 | 0 | 0 | 0 | 0 | 0 | 0 | 0 | 1 |
| ACSL6 | 3 | 0 | 0 | 0 | 0 | 0 | 0 | 0 | 1 |
| ZNF879 | 3 | 0 | 0 | 0 | 0 | 0 | 0 | 0 | 1 |
| ZNF605 | 3 | 0 | 0 | 0 | 0 | 0 | 0 | 0 | 1 |
| ENTHD1 | 3 | 0 | 0 | 0 | 0 | 0 | 0 | 0 | 1 |
| STRA6 | 3 | 0 | 0 | 0 | 0 | 0 | 0 | 0 | 1 |
| TRIM9 | 2 | 1 | 0 | 0 | 0 | 0 | 0 | 0 | 1 |
| KLB | 2 | 1 | 0 | 0 | 0 | 0 | 0 | 0 | 1 |
| TTC12 | 3 | 0 | 0 | 0 | 0 | 0 | 0 | 0 | 1 |
| ATXN7L2 | 2 | 0 | 0 | 0 | 1 | 0 | 0 | 0 | 1 |
| AKAP10 | 3 | 0 | 0 | 0 | 0 | 0 | 0 | 0 | 1 |
| MSH3 | 2 | 1 | 0 | 0 | 0 | 0 | 0 | 0 | 1 |
| GAD2 | 3 | 0 | 0 | 0 | 0 | 0 | 0 | 0 | 1 |
| CYP2C18 | 3 | 0 | 0 | 0 | 0 | 0 | 0 | 0 | 1 |
| PHTF1 | 3 | 0 | 0 | 0 | 0 | 0 | 0 | 0 | 1 |
| PADI6 | 2 | 1 | 0 | 0 | 0 | 0 | 0 | 0 | 1 |
| PHTF2 | 3 | 0 | 0 | 0 | 0 | 0 | 0 | 0 | 1 |
| TBC1D2 | 3 | 0 | 0 | 0 | 0 | 0 | 0 | 0 | 1 |
| LPP | 3 | 0 | 0 | 0 | 0 | 0 | 0 | 0 | 1 |
| MMP15 | 3 | 0 | 0 | 0 | 0 | 0 | 0 | 0 | 1 |
| LRRC8C | 3 | 0 | 0 | 0 | 0 | 0 | 0 | 0 | 1 |
| SCFD2 | 3 | 0 | 0 | 0 | 0 | 0 | 0 | 0 | 1 |
| PHACTR3 | 3 | 0 | 0 | 0 | 0 | 0 | 0 | 0 | 1 |
| IMPG1 | 2 | 0 | 0 | 0 | 1 | 0 | 0 | 0 | 1 |
| XPO6 | 3 | 0 | 0 | 0 | 0 | 0 | 0 | 0 | 1 |
| SOS1 | 2 | 1 | 0 | 0 | 0 | 0 | 0 | 0 | 1 |
| MUM1L1 | 3 | 0 | 0 | 0 | 0 | 0 | 0 | 0 | 1 |
| C1orf38 | 3 | 0 | 0 | 0 | 0 | 0 | 0 | 0 | 1 |
| MMP12 | 3 | 0 | 0 | 0 | 0 | 0 | 0 | 0 | 1 |
| MARS | 3 | 0 | 0 | 0 | 0 | 0 | 0 | 0 | 1 |

| | | | | | | | | | |
|---|---|---|---|---|---|---|---|---|---|
| FOXN1 | 3 | 0 | 0 | 0 | 0 | 0 | 0 | 0 | 1 |
| OR1J2 | 3 | 0 | 0 | 0 | 0 | 0 | 0 | 0 | 1 |
| ADAM8 | 3 | 0 | 0 | 0 | 0 | 0 | 0 | 0 | 1 |
| GPA33 | 3 | 0 | 0 | 0 | 0 | 0 | 0 | 0 | 1 |
| MEP1B | 3 | 0 | 0 | 0 | 0 | 0 | 0 | 0 | 1 |
| NAA15 | 3 | 0 | 0 | 0 | 0 | 0 | 0 | 0 | 1 |
| OR51B4 | 3 | 0 | 0 | 0 | 0 | 0 | 0 | 0 | 1 |
| ARHGAP25 | 3 | 0 | 0 | 0 | 0 | 0 | 0 | 0 | 1 |
| ESRRG | 3 | 0 | 0 | 0 | 0 | 0 | 0 | 0 | 1 |
| LRFN2 | 3 | 0 | 0 | 0 | 0 | 0 | 0 | 0 | 1 |
| ALB | 3 | 0 | 0 | 0 | 0 | 0 | 0 | 0 | 1 |
| IL27RA | 2 | 1 | 0 | 0 | 0 | 0 | 0 | 0 | 1 |
| RBM26 | 2 | 1 | 0 | 0 | 0 | 0 | 0 | 0 | 1 |
| PION | 3 | 0 | 0 | 0 | 0 | 0 | 0 | 0 | 1 |
| PFKP | 3 | 0 | 0 | 0 | 0 | 0 | 0 | 0 | 1 |
| HELQ | 3 | 0 | 0 | 0 | 0 | 0 | 0 | 0 | 1 |
| POLG | 3 | 0 | 0 | 0 | 0 | 0 | 0 | 0 | 1 |
| PLOD3 | 3 | 0 | 0 | 0 | 0 | 0 | 0 | 0 | 1 |
| CNGB3 | 3 | 0 | 0 | 0 | 0 | 0 | 0 | 0 | 1 |
| UBTF | 3 | 0 | 0 | 0 | 0 | 0 | 0 | 0 | 1 |
| CAPN13 | 2 | 1 | 0 | 0 | 0 | 0 | 0 | 0 | 1 |
| ZNF547 | 3 | 0 | 0 | 0 | 0 | 0 | 0 | 0 | 1 |
| FEZF2 | 3 | 0 | 0 | 0 | 0 | 0 | 0 | 0 | 1 |
| SLC2A3 | 3 | 0 | 0 | 0 | 0 | 0 | 0 | 0 | 1 |
| OR6C74 | 3 | 0 | 0 | 0 | 0 | 0 | 0 | 0 | 1 |
| ZNF569 | 3 | 0 | 0 | 0 | 0 | 0 | 0 | 0 | 1 |
| MAPK8IP2 | 2 | 0 | 1 | 0 | 0 | 0 | 0 | 0 | 1 |
| SEMA7A | 3 | 0 | 0 | 0 | 0 | 0 | 0 | 0 | 1 |
| SLC6A17 | 3 | 0 | 0 | 0 | 0 | 0 | 0 | 0 | 1 |
| TTLL3 | 3 | 0 | 0 | 0 | 0 | 0 | 0 | 0 | 1 |
| LMOD1 | 3 | 0 | 0 | 0 | 0 | 0 | 0 | 0 | 1 |
| NOS3 | 2 | 1 | 0 | 0 | 0 | 0 | 0 | 0 | 1 |
| PTPRN | 3 | 0 | 0 | 0 | 0 | 0 | 0 | 0 | 1 |
| UHRF1BP1L | 2 | 1 | 0 | 0 | 0 | 0 | 0 | 0 | 1 |
| PTPRR | 3 | 0 | 0 | 0 | 0 | 0 | 0 | 0 | 1 |
| HERC6 | 3 | 0 | 0 | 0 | 0 | 0 | 0 | 0 | 1 |
| PEX5 | 3 | 0 | 0 | 0 | 0 | 0 | 0 | 0 | 1 |
| ANKFN1 | 3 | 0 | 0 | 0 | 0 | 0 | 0 | 0 | 1 |
| C3orf20 | 3 | 0 | 0 | 0 | 0 | 0 | 0 | 0 | 1 |
| BTBD18 | 2 | 1 | 0 | 0 | 0 | 0 | 0 | 0 | 1 |
| ZKSCAN5 | 3 | 0 | 0 | 0 | 0 | 0 | 0 | 0 | 1 |
| JAK1 | 2 | 1 | 0 | 0 | 0 | 0 | 0 | 0 | 1 |
| ZNF91 | 2 | 1 | 0 | 0 | 0 | 0 | 0 | 0 | 1 |
| IFI16 | 3 | 0 | 0 | 0 | 0 | 0 | 0 | 0 | 1 |
| SETDB1 | 2 | 1 | 0 | 0 | 0 | 0 | 0 | 0 | 1 |
| SEC14L1 | 3 | 0 | 0 | 0 | 0 | 0 | 0 | 0 | 1 |

| | | | | | | | | | |
|---|---|---|---|---|---|---|---|---|---|
| LDB3 | 2 | 0 | 1 | 0 | 0 | 0 | 0 | 0 | 1 |
| CEACAM5 | 3 | 0 | 0 | 0 | 0 | 0 | 0 | 0 | 1 |
| POF1B | 3 | 0 | 0 | 0 | 0 | 0 | 0 | 0 | 1 |
| IGSF21 | 3 | 0 | 0 | 0 | 0 | 0 | 0 | 0 | 1 |
| OR10R2 | 3 | 0 | 0 | 0 | 0 | 0 | 0 | 0 | 1 |
| MCM2 | 3 | 0 | 0 | 0 | 0 | 0 | 0 | 0 | 1 |
| CBL | 3 | 0 | 0 | 0 | 0 | 0 | 0 | 0 | 1 |
| DPP4 | 3 | 0 | 0 | 0 | 0 | 0 | 0 | 0 | 1 |
| SEC24B | 2 | 0 | 1 | 0 | 0 | 0 | 0 | 0 | 1 |
| DSG1 | 3 | 0 | 0 | 0 | 0 | 0 | 0 | 0 | 1 |
| CSDE1 | 3 | 0 | 0 | 0 | 0 | 0 | 0 | 0 | 1 |
| ZNF878 | 3 | 0 | 0 | 0 | 0 | 0 | 0 | 0 | 1 |
| CDKN2AIP | 3 | 0 | 0 | 0 | 0 | 0 | 0 | 0 | 1 |
| SPG11 | 1 | 2 | 0 | 0 | 0 | 0 | 0 | 0 | 1 |
| ABCB6 | 3 | 0 | 0 | 0 | 0 | 0 | 0 | 0 | 1 |
| ALDH1B1 | 3 | 0 | 0 | 0 | 0 | 0 | 0 | 0 | 1 |
| ZNF253 | 3 | 0 | 0 | 0 | 0 | 0 | 0 | 0 | 1 |
| GPR116 | 2 | 1 | 0 | 0 | 0 | 0 | 0 | 0 | 1 |
| CD6 | 3 | 0 | 0 | 0 | 0 | 0 | 0 | 0 | 1 |
| ITPKC | 3 | 0 | 0 | 0 | 0 | 0 | 0 | 0 | 1 |
| CPXM1 | 3 | 0 | 0 | 0 | 0 | 0 | 0 | 0 | 1 |
| PCDHB15 | 3 | 0 | 0 | 0 | 0 | 0 | 0 | 0 | 1 |
| SCML2 | 3 | 0 | 0 | 0 | 0 | 0 | 0 | 0 | 1 |
| ST8SIA5 | 2 | 1 | 0 | 0 | 0 | 0 | 0 | 0 | 1 |
| DCAF13 | 3 | 0 | 0 | 0 | 0 | 0 | 0 | 0 | 1 |
| ZC3H11A | 3 | 0 | 0 | 0 | 0 | 0 | 0 | 0 | 1 |
| PLCL1 | 3 | 0 | 0 | 0 | 0 | 0 | 0 | 0 | 1 |
| NR5A1 | 3 | 0 | 0 | 0 | 0 | 0 | 0 | 0 | 1 |
| AXIN2 | 3 | 0 | 0 | 0 | 0 | 0 | 0 | 0 | 1 |
| DHX32 | 3 | 0 | 0 | 0 | 0 | 0 | 0 | 0 | 1 |
| KLHL13 | 2 | 0 | 0 | 0 | 1 | 0 | 0 | 0 | 1 |
| CLSTN1 | 3 | 0 | 0 | 0 | 0 | 0 | 0 | 0 | 1 |
| PKN3 | 3 | 0 | 0 | 0 | 0 | 0 | 0 | 0 | 1 |
| PUS7L | 3 | 0 | 0 | 0 | 0 | 0 | 0 | 0 | 1 |
| PPARGC1B | 3 | 0 | 0 | 0 | 0 | 0 | 0 | 0 | 1 |
| EMILIN2 | 3 | 0 | 0 | 0 | 0 | 0 | 0 | 0 | 1 |
| TTF1 | 3 | 0 | 0 | 0 | 0 | 0 | 0 | 0 | 1 |
| CROT | 3 | 0 | 0 | 0 | 0 | 0 | 0 | 0 | 1 |
| MUSK | 3 | 0 | 0 | 0 | 0 | 0 | 0 | 0 | 1 |
| HJURP | 3 | 0 | 0 | 0 | 0 | 0 | 0 | 0 | 1 |
| ZNF382 | 3 | 0 | 0 | 0 | 0 | 0 | 0 | 0 | 1 |
| TRDMT1 | 3 | 0 | 0 | 0 | 0 | 0 | 0 | 0 | 1 |
| GPR115 | 3 | 0 | 0 | 0 | 0 | 0 | 0 | 0 | 1 |
| ZNF599 | 3 | 0 | 0 | 0 | 0 | 0 | 0 | 0 | 1 |
| ZNF665 | 3 | 0 | 0 | 0 | 0 | 0 | 0 | 0 | 1 |
| ADAM7 | 3 | 0 | 0 | 0 | 0 | 0 | 0 | 0 | 1 |

| | | | | | | | | | |
|---|---|---|---|---|---|---|---|---|---|
| ZCCHC11 | 2 | 0 | 1 | 0 | 0 | 0 | 0 | 0 | 1 |
| BBS7 | 3 | 0 | 0 | 0 | 0 | 0 | 0 | 0 | 1 |
| SLC22A9 | 3 | 0 | 0 | 0 | 0 | 0 | 0 | 0 | 1 |
| PDZD3 | 2 | 1 | 0 | 0 | 0 | 0 | 0 | 0 | 1 |
| RNF220 | 3 | 0 | 0 | 0 | 0 | 0 | 0 | 0 | 1 |
| NISCH | 3 | 0 | 0 | 0 | 0 | 0 | 0 | 0 | 1 |
| ATP6V0A2 | 3 | 0 | 0 | 0 | 0 | 0 | 0 | 0 | 1 |
| AKT3 | 3 | 0 | 0 | 0 | 0 | 0 | 0 | 0 | 1 |
| CLEC16A | 3 | 0 | 0 | 0 | 0 | 0 | 0 | 0 | 1 |
| MGAT4A | 3 | 0 | 0 | 0 | 0 | 0 | 0 | 0 | 1 |
| ARHGEF15 | 2 | 0 | 0 | 0 | 1 | 0 | 0 | 0 | 1 |
| NEBL | 1 | 0 | 2 | 0 | 0 | 0 | 0 | 0 | 1 |
| CES3 | 3 | 0 | 0 | 0 | 0 | 0 | 0 | 0 | 1 |
| LCP1 | 2 | 0 | 1 | 0 | 0 | 0 | 0 | 0 | 1 |
| PSMD1 | 3 | 0 | 0 | 0 | 0 | 0 | 0 | 0 | 1 |
| UBA6 | 3 | 0 | 0 | 0 | 0 | 0 | 0 | 0 | 1 |
| OR2G3 | 3 | 0 | 0 | 0 | 0 | 0 | 0 | 0 | 1 |
| PPEF1 | 3 | 0 | 0 | 0 | 0 | 0 | 0 | 0 | 1 |
| ITM2C | 3 | 0 | 0 | 0 | 0 | 0 | 0 | 0 | 1 |
| F11 | 3 | 0 | 0 | 0 | 0 | 0 | 0 | 0 | 1 |
| SH2D5 | 3 | 0 | 0 | 0 | 0 | 0 | 0 | 0 | 1 |
| MAN2B1 | 2 | 0 | 0 | 0 | 1 | 0 | 0 | 0 | 1 |
| DAB2IP | 3 | 0 | 0 | 0 | 0 | 0 | 0 | 0 | 1 |
| NCOA5 | 3 | 0 | 0 | 0 | 0 | 0 | 0 | 0 | 1 |
| TULP2 | 3 | 0 | 0 | 0 | 0 | 0 | 0 | 0 | 1 |
| PRPF4B | 2 | 1 | 0 | 0 | 0 | 0 | 0 | 0 | 1 |
| PDE6A | 3 | 0 | 0 | 0 | 0 | 0 | 0 | 0 | 1 |
| RPTOR | 3 | 0 | 0 | 0 | 0 | 0 | 0 | 0 | 1 |
| ATP2C2 | 3 | 0 | 0 | 0 | 0 | 0 | 0 | 0 | 1 |
| OLFML2B | 3 | 0 | 0 | 0 | 0 | 0 | 0 | 0 | 1 |
| FKBP15 | 2 | 1 | 0 | 0 | 0 | 0 | 0 | 0 | 1 |
| NEUROD4 | 3 | 0 | 0 | 0 | 0 | 0 | 0 | 0 | 1 |
| RRBP1 | 3 | 0 | 0 | 0 | 0 | 0 | 0 | 0 | 1 |
| AHI1 | 3 | 0 | 0 | 0 | 0 | 0 | 0 | 0 | 1 |
| ZBTB38 | 3 | 0 | 0 | 0 | 0 | 0 | 0 | 0 | 1 |
| ZP4 | 3 | 0 | 0 | 0 | 0 | 0 | 0 | 0 | 1 |
| ALDH16A1 | 3 | 0 | 0 | 0 | 0 | 0 | 0 | 0 | 1 |
| LRRIQ3 | 3 | 0 | 0 | 0 | 0 | 0 | 0 | 0 | 1 |
| CARD14 | 3 | 0 | 0 | 0 | 0 | 0 | 0 | 0 | 1 |
| C4orf37 | 3 | 0 | 0 | 0 | 0 | 0 | 0 | 0 | 1 |
| ADAM11 | 3 | 0 | 0 | 0 | 0 | 0 | 0 | 0 | 1 |
| DLG3 | 3 | 0 | 0 | 0 | 0 | 0 | 0 | 0 | 1 |
| SLC17A4 | 3 | 0 | 0 | 0 | 0 | 0 | 0 | 0 | 1 |
| ACSM1 | 3 | 0 | 0 | 0 | 0 | 0 | 0 | 0 | 1 |
| ZNF251 | 3 | 0 | 0 | 0 | 0 | 0 | 0 | 0 | 1 |
| FGFR1 | 3 | 0 | 0 | 0 | 0 | 0 | 0 | 0 | 1 |

| | | | | | | | | | |
|---|---|---|---|---|---|---|---|---|---|
| PADI2 | 3 | 0 | 0 | 0 | 0 | 0 | 0 | 0 | 1 |
| TEX2 | 3 | 0 | 0 | 0 | 0 | 0 | 0 | 0 | 1 |
| FBXL13 | 3 | 0 | 0 | 0 | 0 | 0 | 0 | 0 | 1 |
| ZMYM6 | 3 | 0 | 0 | 0 | 0 | 0 | 0 | 0 | 1 |
| RC3H2 | 3 | 0 | 0 | 0 | 0 | 0 | 0 | 0 | 1 |
| CASP14 | 3 | 0 | 0 | 0 | 0 | 0 | 0 | 0 | 1 |
| ZNF827 | 3 | 0 | 0 | 0 | 0 | 0 | 0 | 0 | 1 |
| IGDCC4 | 3 | 0 | 0 | 0 | 0 | 0 | 0 | 0 | 1 |
| XAB2 | 3 | 0 | 0 | 0 | 0 | 0 | 0 | 0 | 1 |
| CSF1R | 2 | 0 | 1 | 0 | 0 | 0 | 0 | 0 | 1 |
| HCFC2 | 3 | 0 | 0 | 0 | 0 | 0 | 0 | 0 | 1 |
| LIMA1 | 3 | 0 | 0 | 0 | 0 | 0 | 0 | 0 | 1 |
| IPO4 | 3 | 0 | 0 | 0 | 0 | 0 | 0 | 0 | 1 |
| MPP4 | 3 | 0 | 0 | 0 | 0 | 0 | 0 | 0 | 1 |
| KIAA1967 | 3 | 0 | 0 | 0 | 0 | 0 | 0 | 0 | 1 |
| USPL1 | 3 | 0 | 0 | 0 | 0 | 0 | 0 | 0 | 1 |
| KCND2 | 3 | 0 | 0 | 0 | 0 | 0 | 0 | 0 | 1 |
| NEDD4L | 2 | 1 | 0 | 0 | 0 | 0 | 0 | 0 | 1 |
| IQGAP1 | 2 | 1 | 0 | 0 | 0 | 0 | 0 | 0 | 1 |
| CHST9 | 3 | 0 | 0 | 0 | 0 | 0 | 0 | 0 | 1 |
| LTK | 3 | 0 | 0 | 0 | 0 | 0 | 0 | 0 | 1 |
| KCNK10 | 3 | 0 | 0 | 0 | 0 | 0 | 0 | 0 | 1 |
| JAK3 | 3 | 0 | 0 | 0 | 0 | 0 | 0 | 0 | 1 |
| CACNB2 | 3 | 0 | 0 | 0 | 0 | 0 | 0 | 0 | 1 |
| ZNF841 | 3 | 0 | 0 | 0 | 0 | 0 | 0 | 0 | 1 |
| AP2A1 | 3 | 0 | 0 | 0 | 0 | 0 | 0 | 0 | 1 |
| PCDHGA5 | 3 | 0 | 0 | 0 | 0 | 0 | 0 | 0 | 1 |
| NPHS1 | 1 | 2 | 0 | 0 | 0 | 0 | 0 | 0 | 1 |
| NOL6 | 3 | 0 | 0 | 0 | 0 | 0 | 0 | 0 | 1 |
| CDON | 2 | 0 | 1 | 0 | 0 | 0 | 0 | 0 | 1 |
| DHX33 | 3 | 0 | 0 | 0 | 0 | 0 | 0 | 0 | 1 |
| IRAK3 | 3 | 0 | 0 | 0 | 0 | 0 | 0 | 0 | 1 |
| DCLK2 | 3 | 0 | 0 | 0 | 0 | 0 | 0 | 0 | 1 |
| TRIM25 | 3 | 0 | 0 | 0 | 0 | 0 | 0 | 0 | 1 |
| COL9A2 | 3 | 0 | 0 | 0 | 0 | 0 | 0 | 0 | 1 |
| ZNF160 | 3 | 0 | 0 | 0 | 0 | 0 | 0 | 0 | 1 |
| ARSH | 3 | 0 | 0 | 0 | 0 | 0 | 0 | 0 | 1 |
| LRRN2 | 2 | 1 | 0 | 0 | 0 | 0 | 0 | 0 | 1 |
| PCDHGA7 | 3 | 0 | 0 | 0 | 0 | 0 | 0 | 0 | 1 |
| KIAA1751 | 3 | 0 | 0 | 0 | 0 | 0 | 0 | 0 | 1 |
| RBL1 | 3 | 0 | 0 | 0 | 0 | 0 | 0 | 0 | 1 |
| TTC29 | 3 | 0 | 0 | 0 | 0 | 0 | 0 | 0 | 1 |
| DAAM2 | 1 | 1 | 1 | 0 | 0 | 0 | 0 | 0 | 1 |
| PIWIL2 | 3 | 0 | 0 | 0 | 0 | 0 | 0 | 0 | 1 |
| TJAP1 | 3 | 0 | 0 | 0 | 0 | 0 | 0 | 0 | 1 |
| ZNF329 | 3 | 0 | 0 | 0 | 0 | 0 | 0 | 0 | 1 |

| | | | | | | | | | |
|---|---|---|---|---|---|---|---|---|---|
| POLN | 3 | 0 | 0 | 0 | 0 | 0 | 0 | 0 | 1 |
| ZNF77 | 2 | 1 | 0 | 0 | 0 | 0 | 0 | 0 | 1 |
| FCHO2 | 3 | 0 | 0 | 0 | 0 | 0 | 0 | 0 | 1 |
| FAM71E2 | 3 | 0 | 0 | 0 | 0 | 0 | 0 | 0 | 1 |
| INSR | 2 | 1 | 0 | 0 | 0 | 0 | 0 | 0 | 1 |
| ALOXE3 | 3 | 0 | 0 | 0 | 0 | 0 | 0 | 0 | 1 |
| PCDHGB2 | 3 | 0 | 0 | 0 | 0 | 0 | 0 | 0 | 1 |
| SLC5A1 | 3 | 0 | 0 | 0 | 0 | 0 | 0 | 0 | 1 |
| MLXIP | 3 | 0 | 0 | 0 | 0 | 0 | 0 | 0 | 1 |
| SLITRK6 | 3 | 0 | 0 | 0 | 0 | 0 | 0 | 0 | 1 |
| WFS1 | 3 | 0 | 0 | 0 | 0 | 0 | 0 | 0 | 1 |
| MAP7 | 3 | 0 | 0 | 0 | 0 | 0 | 0 | 0 | 1 |
| WDR33 | 2 | 1 | 0 | 0 | 0 | 0 | 0 | 0 | 1 |
| OR2G2 | 3 | 0 | 0 | 0 | 0 | 0 | 0 | 0 | 1 |
| PCDHA13 | 3 | 0 | 0 | 0 | 0 | 0 | 0 | 0 | 1 |
| LARP1 | 3 | 0 | 0 | 0 | 0 | 0 | 0 | 0 | 1 |
| ZNF592 | 3 | 0 | 0 | 0 | 0 | 0 | 0 | 0 | 1 |
| PCSK2 | 3 | 0 | 0 | 0 | 0 | 0 | 0 | 0 | 1 |
| PSD4 | 3 | 0 | 0 | 0 | 0 | 0 | 0 | 0 | 1 |
| MTSS1 | 3 | 0 | 0 | 0 | 0 | 0 | 0 | 0 | 1 |
| GLG1 | 2 | 1 | 0 | 0 | 0 | 0 | 0 | 0 | 1 |
| PLEKHG4 | 3 | 0 | 0 | 0 | 0 | 0 | 0 | 0 | 1 |
| C14orf39 | 3 | 0 | 0 | 0 | 0 | 0 | 0 | 0 | 1 |
| FCRL1 | 3 | 0 | 0 | 0 | 0 | 0 | 0 | 0 | 1 |
| CEACAM8 | 3 | 0 | 0 | 0 | 0 | 0 | 0 | 0 | 1 |
| DLG1 | 3 | 0 | 0 | 0 | 0 | 0 | 0 | 0 | 1 |
| PCDHGA2 | 3 | 0 | 0 | 0 | 0 | 0 | 0 | 0 | 1 |
| ZNF573 | 3 | 0 | 0 | 0 | 0 | 0 | 0 | 0 | 1 |
| DHX36 | 1 | 1 | 1 | 0 | 0 | 0 | 0 | 0 | 1 |
| GPAM | 3 | 0 | 0 | 0 | 0 | 0 | 0 | 0 | 1 |
| CLCN6 | 3 | 0 | 0 | 0 | 0 | 0 | 0 | 0 | 1 |
| AGBL3 | 3 | 0 | 0 | 0 | 0 | 0 | 0 | 0 | 1 |
| TLE4 | 3 | 0 | 0 | 0 | 0 | 0 | 0 | 0 | 1 |
| OSTN | 3 | 0 | 0 | 0 | 0 | 0 | 0 | 0 | 1 |
| EIF2AK3 | 3 | 0 | 0 | 0 | 0 | 0 | 0 | 0 | 1 |
| SLAMF1 | 3 | 0 | 0 | 0 | 0 | 0 | 0 | 0 | 1 |
| ZNF629 | 3 | 0 | 0 | 0 | 0 | 0 | 0 | 0 | 1 |
| CYP19A1 | 3 | 0 | 0 | 0 | 0 | 0 | 0 | 0 | 1 |
| FGD3 | 3 | 0 | 0 | 0 | 0 | 0 | 0 | 0 | 1 |
| CNTN1 | 2 | 1 | 0 | 0 | 0 | 0 | 0 | 0 | 1 |
| SLC26A5 | 2 | 1 | 0 | 0 | 0 | 0 | 0 | 0 | 1 |
| NLRP7 | 3 | 0 | 0 | 0 | 0 | 0 | 0 | 0 | 1 |
| EGFLAM | 2 | 0 | 0 | 0 | 1 | 0 | 0 | 0 | 1 |
| PAPSS2 | 3 | 0 | 0 | 0 | 0 | 0 | 0 | 0 | 1 |
| HMGCLL1 | 3 | 0 | 0 | 0 | 0 | 0 | 0 | 0 | 1 |
| RSPO2 | 3 | 0 | 0 | 0 | 0 | 0 | 0 | 0 | 1 |

| | | | | | | | | | |
|---|---|---|---|---|---|---|---|---|---|
| IL4R | 3 | 0 | 0 | 0 | 0 | 0 | 0 | 0 | 1 |
| MEGF11 | 2 | 1 | 0 | 0 | 0 | 0 | 0 | 0 | 1 |
| NARG2 | 3 | 0 | 0 | 0 | 0 | 0 | 0 | 0 | 1 |
| ZNF836 | 3 | 0 | 0 | 0 | 0 | 0 | 0 | 0 | 1 |
| BCAS1 | 3 | 0 | 0 | 0 | 0 | 0 | 0 | 0 | 1 |
| C17orf104 | 3 | 0 | 0 | 0 | 0 | 0 | 0 | 0 | 1 |
| GPR144 | 3 | 0 | 0 | 0 | 0 | 0 | 0 | 0 | 1 |
| TMC1 | 3 | 0 | 0 | 0 | 0 | 0 | 0 | 0 | 1 |
| FAM129A | 3 | 0 | 0 | 0 | 0 | 0 | 0 | 0 | 1 |
| STOX2 | 3 | 0 | 0 | 0 | 0 | 0 | 0 | 0 | 1 |
| OR5AU1 | 2 | 1 | 0 | 0 | 0 | 0 | 0 | 0 | 1 |
| SEC23IP | 3 | 0 | 0 | 0 | 0 | 0 | 0 | 0 | 1 |
| DDX60 | 2 | 1 | 0 | 0 | 0 | 0 | 0 | 0 | 1 |
| INTU | 3 | 0 | 0 | 0 | 0 | 0 | 0 | 0 | 1 |
| SCUBE1 | 3 | 0 | 0 | 0 | 0 | 0 | 0 | 0 | 1 |
| UBE2O | 3 | 0 | 0 | 0 | 0 | 0 | 0 | 0 | 1 |
| REV1 | 3 | 0 | 0 | 0 | 0 | 0 | 0 | 0 | 1 |
| LRRTM1 | 3 | 0 | 0 | 0 | 0 | 0 | 0 | 0 | 1 |
| ZNF845 | 3 | 0 | 0 | 0 | 0 | 0 | 0 | 0 | 1 |
| BRD4 | 3 | 0 | 0 | 0 | 0 | 0 | 0 | 0 | 1 |
| OR6B1 | 2 | 1 | 0 | 0 | 0 | 0 | 0 | 0 | 1 |
| USP19 | 3 | 0 | 0 | 0 | 0 | 0 | 0 | 0 | 1 |
| TBX3 | 3 | 0 | 0 | 0 | 0 | 0 | 0 | 0 | 1 |
| PELP1 | 3 | 0 | 0 | 0 | 0 | 0 | 0 | 0 | 1 |
| OR5L2 | 3 | 0 | 0 | 0 | 0 | 0 | 0 | 0 | 1 |
| PDE6C | 3 | 0 | 0 | 0 | 0 | 0 | 0 | 0 | 1 |
| GRPR | 3 | 0 | 0 | 0 | 0 | 0 | 0 | 0 | 1 |
| GRIK1 | 2 | 0 | 1 | 0 | 0 | 0 | 0 | 0 | 1 |
| RTN1 | 3 | 0 | 0 | 0 | 0 | 0 | 0 | 0 | 1 |
| SART3 | 2 | 1 | 0 | 0 | 0 | 0 | 0 | 0 | 1 |
| LARS | 3 | 0 | 0 | 0 | 0 | 0 | 0 | 0 | 1 |
| CHAT | 3 | 0 | 0 | 0 | 0 | 0 | 0 | 0 | 1 |
| MICAL2 | 3 | 0 | 0 | 0 | 0 | 0 | 0 | 0 | 1 |
| HTR1B | 3 | 0 | 0 | 0 | 0 | 0 | 0 | 0 | 1 |
| PPFIA1 | 3 | 0 | 0 | 0 | 0 | 0 | 0 | 0 | 1 |
| EPHB2 | 3 | 0 | 0 | 0 | 0 | 0 | 0 | 0 | 1 |
| ABCC10 | 3 | 0 | 0 | 0 | 0 | 0 | 0 | 0 | 1 |
| SLC4A1 | 2 | 0 | 1 | 0 | 0 | 0 | 0 | 0 | 1 |
| MAPT | 2 | 1 | 0 | 0 | 0 | 0 | 0 | 0 | 1 |
| UBE4B | 3 | 0 | 0 | 0 | 0 | 0 | 0 | 0 | 1 |
| AP3D1 | 3 | 0 | 0 | 0 | 0 | 0 | 0 | 0 | 1 |
| OPTC | 3 | 0 | 0 | 0 | 0 | 0 | 0 | 0 | 1 |
| OSBPL7 | 3 | 0 | 0 | 0 | 0 | 0 | 0 | 0 | 1 |
| LRFN5 | 3 | 0 | 0 | 0 | 0 | 0 | 0 | 0 | 1 |
| CLMN | 3 | 0 | 0 | 0 | 0 | 0 | 0 | 0 | 1 |
| CPNE6 | 3 | 0 | 0 | 0 | 0 | 0 | 0 | 0 | 1 |

| | | | | | | | | | |
|---|---|---|---|---|---|---|---|---|---|
| ZNF224 | 2 | 1 | 0 | 0 | 0 | 0 | 0 | 0 | 1 |
| KIAA0319 | 3 | 0 | 0 | 0 | 0 | 0 | 0 | 0 | 1 |
| OR5L1 | 3 | 0 | 0 | 0 | 0 | 0 | 0 | 0 | 1 |
| KCNT1 | 3 | 0 | 0 | 0 | 0 | 0 | 0 | 0 | 1 |
| RPL10L | 3 | 0 | 0 | 0 | 0 | 0 | 0 | 0 | 1 |
| MYO1H | 3 | 0 | 0 | 0 | 0 | 0 | 0 | 0 | 1 |
| VPS39 | 3 | 0 | 0 | 0 | 0 | 0 | 0 | 0 | 1 |
| TMEM48 | 3 | 0 | 0 | 0 | 0 | 0 | 0 | 0 | 1 |
| SIK2 | 3 | 0 | 0 | 0 | 0 | 0 | 0 | 0 | 1 |
| MAP3K9 | 2 | 0 | 0 | 0 | 1 | 0 | 0 | 0 | 1 |
| ADAMTS1 | 3 | 0 | 0 | 0 | 0 | 0 | 0 | 0 | 1 |
| EHBP1 | 3 | 0 | 0 | 0 | 0 | 0 | 0 | 0 | 1 |
| MAML1 | 2 | 0 | 0 | 0 | 1 | 0 | 0 | 0 | 1 |
| EIF3A | 3 | 0 | 0 | 0 | 0 | 0 | 0 | 0 | 1 |
| ILF3 | 3 | 0 | 0 | 0 | 0 | 0 | 0 | 0 | 1 |
| SOX6 | 3 | 0 | 0 | 0 | 0 | 0 | 0 | 0 | 1 |
| PIK3R6 | 3 | 0 | 0 | 0 | 0 | 0 | 0 | 0 | 1 |
| RECQL5 | 3 | 0 | 0 | 0 | 0 | 0 | 0 | 0 | 1 |
| RFX7 | 2 | 1 | 0 | 0 | 0 | 0 | 0 | 0 | 1 |
| RBMXL3 | 3 | 0 | 0 | 0 | 0 | 0 | 0 | 0 | 1 |
| CLCA1 | 3 | 0 | 0 | 0 | 0 | 0 | 0 | 0 | 1 |
| ATCAY | 3 | 0 | 0 | 0 | 0 | 0 | 0 | 0 | 1 |
| RBM20 | 3 | 0 | 0 | 0 | 0 | 0 | 0 | 0 | 1 |
| ZXDC | 2 | 1 | 0 | 0 | 0 | 0 | 0 | 0 | 1 |
| PCSK1 | 3 | 0 | 0 | 0 | 0 | 0 | 0 | 0 | 1 |
| TRIM36 | 3 | 0 | 0 | 0 | 0 | 0 | 0 | 0 | 1 |
| TAF4 | 3 | 0 | 0 | 0 | 0 | 0 | 0 | 0 | 1 |
| CNTN2 | 3 | 0 | 0 | 0 | 0 | 0 | 0 | 0 | 1 |
| C1orf112 | 2 | 0 | 0 | 0 | 1 | 0 | 0 | 0 | 1 |
| CDHR5 | 3 | 0 | 0 | 0 | 0 | 0 | 0 | 0 | 1 |
| PCDHGA3 | 3 | 0 | 0 | 0 | 0 | 0 | 0 | 0 | 1 |
| NCAPD2 | 3 | 0 | 0 | 0 | 0 | 0 | 0 | 0 | 1 |
| XRN1 | 1 | 1 | 1 | 0 | 0 | 0 | 0 | 0 | 1 |
| ADNP | 3 | 0 | 0 | 0 | 0 | 0 | 0 | 0 | 1 |
| RICTOR | 3 | 0 | 0 | 0 | 0 | 0 | 0 | 0 | 1 |
| CDH8 | 2 | 1 | 0 | 0 | 0 | 0 | 0 | 0 | 1 |
| ATP8B3 | 3 | 0 | 0 | 0 | 0 | 0 | 0 | 0 | 1 |
| PALB2 | 3 | 0 | 0 | 0 | 0 | 0 | 0 | 0 | 1 |
| CPT1C | 3 | 0 | 0 | 0 | 0 | 0 | 0 | 0 | 1 |
| ERBB2IP | 3 | 0 | 0 | 0 | 0 | 0 | 0 | 0 | 1 |
| ZNF583 | 3 | 0 | 0 | 0 | 0 | 0 | 0 | 0 | 1 |
| SHC4 | 3 | 0 | 0 | 0 | 0 | 0 | 0 | 0 | 1 |
| ERMP1 | 2 | 0 | 0 | 0 | 1 | 0 | 0 | 0 | 1 |
| SHROOM4 | 3 | 0 | 0 | 0 | 0 | 0 | 0 | 0 | 1 |
| ERN1 | 3 | 0 | 0 | 0 | 0 | 0 | 0 | 0 | 1 |
| RNF20 | 2 | 1 | 0 | 0 | 0 | 0 | 0 | 0 | 1 |

| | | | | | | | | | |
|---|---|---|---|---|---|---|---|---|---|
| ESPNL | 3 | 0 | 0 | 0 | 0 | 0 | 0 | 0 | 1 |
| FAM184A | 2 | 1 | 0 | 0 | 0 | 0 | 0 | 0 | 1 |
| GORAB | 3 | 0 | 0 | 0 | 0 | 0 | 0 | 0 | 1 |
| LHCGR | 3 | 0 | 0 | 0 | 0 | 0 | 0 | 0 | 1 |
| ZNF407 | 2 | 1 | 0 | 0 | 0 | 0 | 0 | 0 | 1 |
| PREX1 | 3 | 0 | 0 | 0 | 0 | 0 | 0 | 0 | 1 |
| GIGYF1 | 3 | 0 | 0 | 0 | 0 | 0 | 0 | 0 | 1 |
| KNDC1 | 2 | 1 | 0 | 0 | 0 | 0 | 0 | 0 | 1 |
| OR5I1 | 3 | 0 | 0 | 0 | 0 | 0 | 0 | 0 | 1 |
| VSIG4 | 3 | 0 | 0 | 0 | 0 | 0 | 0 | 0 | 1 |
| PITPNM2 | 3 | 0 | 0 | 0 | 0 | 0 | 0 | 0 | 1 |
| ANKRD50 | 1 | 2 | 0 | 0 | 0 | 0 | 0 | 0 | 1 |
| CDH13 | 3 | 0 | 0 | 0 | 0 | 0 | 0 | 0 | 1 |
| CALD1 | 3 | 0 | 0 | 0 | 0 | 0 | 0 | 0 | 1 |
| GALNT5 | 3 | 0 | 0 | 0 | 0 | 0 | 0 | 0 | 1 |
| SMG5 | 1 | 2 | 0 | 0 | 0 | 0 | 0 | 0 | 1 |
| SLC12A2 | 2 | 1 | 0 | 0 | 0 | 0 | 0 | 0 | 1 |
| MYBPC1 | 3 | 0 | 0 | 0 | 0 | 0 | 0 | 0 | 1 |
| ITGA4 | 3 | 0 | 0 | 0 | 0 | 0 | 0 | 0 | 1 |
| KRT9 | 3 | 0 | 0 | 0 | 0 | 0 | 0 | 0 | 1 |
| CDC25B | 2 | 1 | 0 | 0 | 0 | 0 | 0 | 0 | 1 |
| PLIN4 | 3 | 0 | 0 | 0 | 0 | 0 | 0 | 0 | 1 |
| OR2L8 | 3 | 0 | 0 | 0 | 0 | 0 | 0 | 0 | 1 |
| SOX5 | 3 | 0 | 0 | 0 | 0 | 0 | 0 | 0 | 1 |
| ANO6 | 3 | 0 | 0 | 0 | 0 | 0 | 0 | 0 | 1 |
| TRPM4 | 3 | 0 | 0 | 0 | 0 | 0 | 0 | 0 | 1 |
| ITGAM | 3 | 0 | 0 | 0 | 0 | 0 | 0 | 0 | 1 |
| INPP5B | 3 | 0 | 0 | 0 | 0 | 0 | 0 | 0 | 1 |
| WNK4 | 3 | 0 | 0 | 0 | 0 | 0 | 0 | 0 | 1 |
| CCDC88B | 3 | 0 | 0 | 0 | 0 | 0 | 0 | 0 | 1 |
| NOS2 | 3 | 0 | 0 | 0 | 0 | 0 | 0 | 0 | 1 |
| TULP4 | 3 | 0 | 0 | 0 | 0 | 0 | 0 | 0 | 1 |
| ZNF235 | 3 | 0 | 0 | 0 | 0 | 0 | 0 | 0 | 1 |
| UROC1 | 2 | 1 | 0 | 0 | 0 | 0 | 0 | 0 | 1 |
| ADAMTS10 | 3 | 0 | 0 | 0 | 0 | 0 | 0 | 0 | 1 |
| ZNF644 | 3 | 0 | 0 | 0 | 0 | 0 | 0 | 0 | 1 |
| PROL1 | 3 | 0 | 0 | 0 | 0 | 0 | 0 | 0 | 1 |
| HNRNPUL1 | 3 | 0 | 0 | 0 | 0 | 0 | 0 | 0 | 1 |
| NFATC4 | 3 | 0 | 0 | 0 | 0 | 0 | 0 | 0 | 1 |
| KIAA0586 | 3 | 0 | 0 | 0 | 0 | 0 | 0 | 0 | 1 |
| WWC3 | 3 | 0 | 0 | 0 | 0 | 0 | 0 | 0 | 1 |
| SMARCA2 | 3 | 0 | 0 | 0 | 0 | 0 | 0 | 0 | 1 |
| BCR | 2 | 0 | 1 | 0 | 0 | 0 | 0 | 0 | 1 |
| UBR1 | 3 | 0 | 0 | 0 | 0 | 0 | 0 | 0 | 1 |
| CD22 | 3 | 0 | 0 | 0 | 0 | 0 | 0 | 0 | 1 |
| CLCA2 | 3 | 0 | 0 | 0 | 0 | 0 | 0 | 0 | 1 |

| | | | | | | | | | |
|---|---|---|---|---|---|---|---|---|---|
| FNDC3B | 3 | 0 | 0 | 0 | 0 | 0 | 0 | 0 | 1 |
| BICC1 | 3 | 0 | 0 | 0 | 0 | 0 | 0 | 0 | 1 |
| ZFR | 2 | 0 | 1 | 0 | 0 | 0 | 0 | 0 | 1 |
| LRP3 | 3 | 0 | 0 | 0 | 0 | 0 | 0 | 0 | 1 |
| SPON1 | 3 | 0 | 0 | 0 | 0 | 0 | 0 | 0 | 1 |
| PCDHA4 | 3 | 0 | 0 | 0 | 0 | 0 | 0 | 0 | 1 |
| PCDHA3 | 3 | 0 | 0 | 0 | 0 | 0 | 0 | 0 | 1 |
| TTBK1 | 3 | 0 | 0 | 0 | 0 | 0 | 0 | 0 | 1 |
| CDH9 | 3 | 0 | 0 | 0 | 0 | 0 | 0 | 0 | 1 |
| CASC5 | 3 | 0 | 0 | 0 | 0 | 0 | 0 | 0 | 1 |
| XYLT1 | 3 | 0 | 0 | 0 | 0 | 0 | 0 | 0 | 1 |
| STXBP5 | 3 | 0 | 0 | 0 | 0 | 0 | 0 | 0 | 1 |
| ZNF85 | 3 | 0 | 0 | 0 | 0 | 0 | 0 | 0 | 1 |
| SLC4A3 | 2 | 0 | 1 | 0 | 0 | 0 | 0 | 0 | 1 |
| ZNF335 | 2 | 0 | 0 | 1 | 0 | 0 | 0 | 0 | 1 |
| KIF20B | 3 | 0 | 0 | 0 | 0 | 0 | 0 | 0 | 1 |
| PDZRN3 | 3 | 0 | 0 | 0 | 0 | 0 | 0 | 0 | 1 |
| EIF2AK4 | 3 | 0 | 0 | 0 | 0 | 0 | 0 | 0 | 1 |
| LMTK2 | 3 | 0 | 0 | 0 | 0 | 0 | 0 | 0 | 1 |
| USP42 | 3 | 0 | 0 | 0 | 0 | 0 | 0 | 0 | 1 |
| SBF1 | 3 | 0 | 0 | 0 | 0 | 0 | 0 | 0 | 1 |
| ASXL2 | 3 | 0 | 0 | 0 | 0 | 0 | 0 | 0 | 1 |
| PEX1 | 3 | 0 | 0 | 0 | 0 | 0 | 0 | 0 | 1 |
| ZNF14 | 1 | 2 | 0 | 0 | 0 | 0 | 0 | 0 | 1 |
| XDH | 3 | 0 | 0 | 0 | 0 | 0 | 0 | 0 | 1 |
| DENND3 | 3 | 0 | 0 | 0 | 0 | 0 | 0 | 0 | 1 |
| NUP188 | 3 | 0 | 0 | 0 | 0 | 0 | 0 | 0 | 1 |
| ZIM2 | 3 | 0 | 0 | 0 | 0 | 0 | 0 | 0 | 1 |
| CARNS1 | 3 | 0 | 0 | 0 | 0 | 0 | 0 | 0 | 1 |
| OR8K5 | 2 | 1 | 0 | 0 | 0 | 0 | 0 | 0 | 1 |
| RNASEL | 3 | 0 | 0 | 0 | 0 | 0 | 0 | 0 | 1 |
| SLCO1A2 | 3 | 0 | 0 | 0 | 0 | 0 | 0 | 0 | 1 |
| PLEKHG2 | 3 | 0 | 0 | 0 | 0 | 0 | 0 | 0 | 1 |
| HGD | 3 | 0 | 0 | 0 | 0 | 0 | 0 | 0 | 1 |
| SLC28A2 | 2 | 0 | 0 | 0 | 1 | 0 | 0 | 0 | 1 |
| TRAPPC9 | 3 | 0 | 0 | 0 | 0 | 0 | 0 | 0 | 1 |
| DHX38 | 3 | 0 | 0 | 0 | 0 | 0 | 0 | 0 | 1 |
| MYH7B | 3 | 0 | 0 | 0 | 0 | 0 | 0 | 0 | 1 |
| SPOCD1 | 3 | 0 | 0 | 0 | 0 | 0 | 0 | 0 | 1 |
| SRRM1 | 2 | 0 | 1 | 0 | 0 | 0 | 0 | 0 | 1 |
| CGNL1 | 2 | 1 | 0 | 0 | 0 | 0 | 0 | 0 | 1 |
| CFTR | 3 | 0 | 0 | 0 | 0 | 0 | 0 | 0 | 1 |
| RASAL2 | 2 | 0 | 1 | 0 | 0 | 0 | 0 | 0 | 1 |
| SEMA6D | 3 | 0 | 0 | 0 | 0 | 0 | 0 | 0 | 1 |
| LRRN4 | 3 | 0 | 0 | 0 | 0 | 0 | 0 | 0 | 1 |
| UBN2 | 2 | 1 | 0 | 0 | 0 | 0 | 0 | 0 | 1 |

| | | | | | | | | | |
|---|---|---|---|---|---|---|---|---|---|
| DBN1 | 3 | 0 | 0 | 0 | 0 | 0 | 0 | 0 | 1 |
| SLC4A1AP | 3 | 0 | 0 | 0 | 0 | 0 | 0 | 0 | 1 |
| AMOTL2 | 3 | 0 | 0 | 0 | 0 | 0 | 0 | 0 | 1 |
| TRPC6 | 3 | 0 | 0 | 0 | 0 | 0 | 0 | 0 | 1 |
| SLCO4C1 | 3 | 0 | 0 | 0 | 0 | 0 | 0 | 0 | 1 |
| KIAA1244 | 3 | 0 | 0 | 0 | 0 | 0 | 0 | 0 | 1 |
| CYP4F11 | 3 | 0 | 0 | 0 | 0 | 0 | 0 | 0 | 1 |
| ITGAD | 3 | 0 | 0 | 0 | 0 | 0 | 0 | 0 | 1 |
| PRTG | 1 | 2 | 0 | 0 | 0 | 0 | 0 | 0 | 1 |
| FAM5B | 3 | 0 | 0 | 0 | 0 | 0 | 0 | 0 | 1 |
| ATP7B | 3 | 0 | 0 | 0 | 0 | 0 | 0 | 0 | 1 |
| MYO5A | 3 | 0 | 0 | 0 | 0 | 0 | 0 | 0 | 1 |
| LRP4 | 3 | 0 | 0 | 0 | 0 | 0 | 0 | 0 | 1 |
| BCMO1 | 3 | 0 | 0 | 0 | 0 | 0 | 0 | 0 | 1 |
| PTPRN2 | 3 | 0 | 0 | 0 | 0 | 0 | 0 | 0 | 1 |
| ST5 | 3 | 0 | 0 | 0 | 0 | 0 | 0 | 0 | 1 |
| CETN1 | 3 | 0 | 0 | 0 | 0 | 0 | 0 | 0 | 1 |
| TRIM58 | 3 | 0 | 0 | 0 | 0 | 0 | 0 | 0 | 1 |
| PARP14 | 3 | 0 | 0 | 0 | 0 | 0 | 0 | 0 | 1 |
| MED23 | 3 | 0 | 0 | 0 | 0 | 0 | 0 | 0 | 1 |
| SETD1A | 3 | 0 | 0 | 0 | 0 | 0 | 0 | 0 | 1 |
| KIRREL3 | 3 | 0 | 0 | 0 | 0 | 0 | 0 | 0 | 1 |
| PCDHGB7 | 3 | 0 | 0 | 0 | 0 | 0 | 0 | 0 | 1 |
| ARID4B | 2 | 0 | 0 | 0 | 1 | 0 | 0 | 0 | 1 |
| GPHN | 3 | 0 | 0 | 0 | 0 | 0 | 0 | 0 | 1 |
| AGTPBP1 | 2 | 1 | 0 | 0 | 0 | 0 | 0 | 0 | 1 |
| GRIK3 | 1 | 2 | 0 | 0 | 0 | 0 | 0 | 0 | 1 |
| SLITRK2 | 3 | 0 | 0 | 0 | 0 | 0 | 0 | 0 | 1 |
| MOCOS | 3 | 0 | 0 | 0 | 0 | 0 | 0 | 0 | 1 |
| GRM3 | 2 | 1 | 0 | 0 | 0 | 0 | 0 | 0 | 1 |
| OR7C1 | 3 | 0 | 0 | 0 | 0 | 0 | 0 | 0 | 1 |
| BCL9L | 3 | 0 | 0 | 0 | 0 | 0 | 0 | 0 | 1 |
| SPARCL1 | 3 | 0 | 0 | 0 | 0 | 0 | 0 | 0 | 1 |
| TNS1 | 3 | 0 | 0 | 0 | 0 | 0 | 0 | 0 | 1 |
| MYH9 | 3 | 0 | 0 | 0 | 0 | 0 | 0 | 0 | 1 |
| CEACAM7 | 3 | 0 | 0 | 0 | 0 | 0 | 0 | 0 | 1 |
| CNR1 | 2 | 1 | 0 | 0 | 0 | 0 | 0 | 0 | 1 |
| KLHL4 | 3 | 0 | 0 | 0 | 0 | 0 | 0 | 0 | 1 |
| NEK9 | 1 | 1 | 1 | 0 | 0 | 0 | 0 | 0 | 1 |
| RSF1 | 3 | 0 | 0 | 0 | 0 | 0 | 0 | 0 | 1 |
| PEG10 | 2 | 1 | 0 | 0 | 0 | 0 | 0 | 0 | 1 |
| KIF7 | 3 | 0 | 0 | 0 | 0 | 0 | 0 | 0 | 1 |
| CUL7 | 3 | 0 | 0 | 0 | 0 | 0 | 0 | 0 | 1 |
| PALM2-AKAP2 | 3 | 0 | 0 | 0 | 0 | 0 | 0 | 0 | 1 |
| LAMB1 | 3 | 0 | 0 | 0 | 0 | 0 | 0 | 0 | 1 |
| AARS2 | 3 | 0 | 0 | 0 | 0 | 0 | 0 | 0 | 1 |

| | | | | | | | | | |
|---|---|---|---|---|---|---|---|---|---|
| TPO | 3 | 0 | 0 | 0 | 0 | 0 | 0 | 0 | 1 |
| EIF4ENIF1 | 3 | 0 | 0 | 0 | 0 | 0 | 0 | 0 | 1 |
| TNN | 3 | 0 | 0 | 0 | 0 | 0 | 0 | 0 | 1 |
| MYOM1 | 3 | 0 | 0 | 0 | 0 | 0 | 0 | 0 | 1 |
| ARHGEF11 | 3 | 0 | 0 | 0 | 0 | 0 | 0 | 0 | 1 |
| PCDH1 | 3 | 0 | 0 | 0 | 0 | 0 | 0 | 0 | 1 |
| TRIM55 | 3 | 0 | 0 | 0 | 0 | 0 | 0 | 0 | 1 |
| BEST3 | 3 | 0 | 0 | 0 | 0 | 0 | 0 | 0 | 1 |
| RBBP8 | 3 | 0 | 0 | 0 | 0 | 0 | 0 | 0 | 1 |
| POSTN | 3 | 0 | 0 | 0 | 0 | 0 | 0 | 0 | 1 |
| GCN1L1 | 2 | 1 | 0 | 0 | 0 | 0 | 0 | 0 | 1 |
| NFRKB | 3 | 0 | 0 | 0 | 0 | 0 | 0 | 0 | 1 |
| USH1C | 3 | 0 | 0 | 0 | 0 | 0 | 0 | 0 | 1 |
| MLLT4 | 3 | 0 | 0 | 0 | 0 | 0 | 0 | 0 | 1 |
| PIK3AP1 | 3 | 0 | 0 | 0 | 0 | 0 | 0 | 0 | 1 |
| PLCZ1 | 3 | 0 | 0 | 0 | 0 | 0 | 0 | 0 | 1 |
| OR14C36 | 3 | 0 | 0 | 0 | 0 | 0 | 0 | 0 | 1 |
| GRIP2 | 3 | 0 | 0 | 0 | 0 | 0 | 0 | 0 | 1 |
| DENND1A | 3 | 0 | 0 | 0 | 0 | 0 | 0 | 0 | 1 |
| GHR | 3 | 0 | 0 | 0 | 0 | 0 | 0 | 0 | 1 |
| FAM13B | 3 | 0 | 0 | 0 | 0 | 0 | 0 | 0 | 1 |
| NUP205 | 3 | 0 | 0 | 0 | 0 | 0 | 0 | 0 | 1 |
| CACNA1H | 3 | 0 | 0 | 0 | 0 | 0 | 0 | 0 | 1 |
| SMOC2 | 2 | 1 | 0 | 0 | 0 | 0 | 0 | 0 | 1 |
| RAB11FIP1 | 3 | 0 | 0 | 0 | 0 | 0 | 0 | 0 | 1 |
| SLC5A10 | 3 | 0 | 0 | 0 | 0 | 0 | 0 | 0 | 1 |
| GPR97 | 3 | 0 | 0 | 0 | 0 | 0 | 0 | 0 | 1 |
| MAPK8IP3 | 3 | 0 | 0 | 0 | 0 | 0 | 0 | 0 | 1 |
| LRRC33 | 3 | 0 | 0 | 0 | 0 | 0 | 0 | 0 | 1 |
| MYOM2 | 2 | 0 | 0 | 0 | 1 | 0 | 0 | 0 | 1 |
| WDR81 | 3 | 0 | 0 | 0 | 0 | 0 | 0 | 0 | 1 |
| C12orf42 | 3 | 0 | 0 | 0 | 0 | 0 | 0 | 0 | 1 |
| PARD3B | 3 | 0 | 0 | 0 | 0 | 0 | 0 | 0 | 1 |
| ECT2L | 3 | 0 | 0 | 0 | 0 | 0 | 0 | 0 | 1 |
| EIF4G3 | 3 | 0 | 0 | 0 | 0 | 0 | 0 | 0 | 1 |
| DET1 | 3 | 0 | 0 | 0 | 0 | 0 | 0 | 0 | 1 |
| PPL | 3 | 0 | 0 | 0 | 0 | 0 | 0 | 0 | 1 |
| C2orf55 | 3 | 0 | 0 | 0 | 0 | 0 | 0 | 0 | 1 |
| SULT1C3 | 3 | 0 | 0 | 0 | 0 | 0 | 0 | 0 | 1 |
| ASXL1 | 2 | 1 | 0 | 0 | 0 | 0 | 0 | 0 | 1 |
| COG5 | 2 | 1 | 0 | 0 | 0 | 0 | 0 | 0 | 1 |
| PHLPP2 | 3 | 0 | 0 | 0 | 0 | 0 | 0 | 0 | 1 |
| TMTC1 | 3 | 0 | 0 | 0 | 0 | 0 | 0 | 0 | 1 |
| ZFP64 | 3 | 0 | 0 | 0 | 0 | 0 | 0 | 0 | 1 |
| FHAD1 | 3 | 0 | 0 | 0 | 0 | 0 | 0 | 0 | 1 |
| VIT | 3 | 0 | 0 | 0 | 0 | 0 | 0 | 0 | 1 |

| | | | | | | | | | |
|---|---|---|---|---|---|---|---|---|---|
| FHOD3 | 3 | 0 | 0 | 0 | 0 | 0 | 0 | 0 | 1 |
| PIK3C2G | 3 | 0 | 0 | 0 | 0 | 0 | 0 | 0 | 1 |
| COBL | 2 | 1 | 0 | 0 | 0 | 0 | 0 | 0 | 1 |
| SART1 | 3 | 0 | 0 | 0 | 0 | 0 | 0 | 0 | 1 |
| NLRP2 | 3 | 0 | 0 | 0 | 0 | 0 | 0 | 0 | 1 |
| TLR5 | 3 | 0 | 0 | 0 | 0 | 0 | 0 | 0 | 1 |
| ZFP28 | 3 | 0 | 0 | 0 | 0 | 0 | 0 | 0 | 1 |
| SHANK1 | 3 | 0 | 0 | 0 | 0 | 0 | 0 | 0 | 1 |
| PHLDB1 | 2 | 1 | 0 | 0 | 0 | 0 | 0 | 0 | 1 |
| SPTBN4 | 3 | 0 | 0 | 0 | 0 | 0 | 0 | 0 | 1 |
| PUM1 | 3 | 0 | 0 | 0 | 0 | 0 | 0 | 0 | 1 |
| ITIH3 | 2 | 0 | 1 | 0 | 0 | 0 | 0 | 0 | 1 |
| PRDM2 | 3 | 0 | 0 | 0 | 0 | 0 | 0 | 0 | 1 |
| PPFIBP1 | 3 | 0 | 0 | 0 | 0 | 0 | 0 | 0 | 1 |
| DOCK5 | 3 | 0 | 0 | 0 | 0 | 0 | 0 | 0 | 1 |
| SYCP2 | 3 | 0 | 0 | 0 | 0 | 0 | 0 | 0 | 1 |
| RADIL | 3 | 0 | 0 | 0 | 0 | 0 | 0 | 0 | 1 |
| ISM2 | 2 | 1 | 0 | 0 | 0 | 0 | 0 | 0 | 1 |
| NSMCE4A | 3 | 0 | 0 | 0 | 0 | 0 | 0 | 0 | 1 |
| ATP12A | 2 | 1 | 0 | 0 | 0 | 0 | 0 | 0 | 1 |
| SLIT3 | 3 | 0 | 0 | 0 | 0 | 0 | 0 | 0 | 1 |
| SSH3 | 3 | 0 | 0 | 0 | 0 | 0 | 0 | 0 | 1 |
| SFMBT1 | 3 | 0 | 0 | 0 | 0 | 0 | 0 | 0 | 1 |
| KIF1A | 3 | 0 | 0 | 0 | 0 | 0 | 0 | 0 | 1 |
| RORC | 3 | 0 | 0 | 0 | 0 | 0 | 0 | 0 | 1 |
| ARHGAP39 | 3 | 0 | 0 | 0 | 0 | 0 | 0 | 0 | 1 |
| ANO3 | 3 | 0 | 0 | 0 | 0 | 0 | 0 | 0 | 1 |
| PTPRF | 3 | 0 | 0 | 0 | 0 | 0 | 0 | 0 | 1 |
| LAMC3 | 3 | 0 | 0 | 0 | 0 | 0 | 0 | 0 | 1 |
| BAZ2B | 1 | 1 | 0 | 0 | 1 | 0 | 0 | 0 | 1 |
| ETV5 | 3 | 0 | 0 | 0 | 0 | 0 | 0 | 0 | 1 |
| FAM155A | 2 | 1 | 0 | 0 | 0 | 0 | 0 | 0 | 1 |
| OR52A1 | 3 | 0 | 0 | 0 | 0 | 0 | 0 | 0 | 1 |
| PLXNA2 | 3 | 0 | 0 | 0 | 0 | 0 | 0 | 0 | 1 |
| INPP4A | 2 | 1 | 0 | 0 | 0 | 0 | 0 | 0 | 1 |
| GPR15 | 3 | 0 | 0 | 0 | 0 | 0 | 0 | 0 | 1 |
| XKR3 | 3 | 0 | 0 | 0 | 0 | 0 | 0 | 0 | 1 |
| MAGI1 | 3 | 0 | 0 | 0 | 0 | 0 | 0 | 0 | 1 |
| ACSL5 | 3 | 0 | 0 | 0 | 0 | 0 | 0 | 0 | 1 |
| PXDN | 3 | 0 | 0 | 0 | 0 | 0 | 0 | 0 | 1 |
| LRPPRC | 3 | 0 | 0 | 0 | 0 | 0 | 0 | 0 | 1 |
| HMGCR | 3 | 0 | 0 | 0 | 0 | 0 | 0 | 0 | 1 |
| USP32 | 2 | 1 | 0 | 0 | 0 | 0 | 0 | 0 | 1 |
| SNW1 | 3 | 0 | 0 | 0 | 0 | 0 | 0 | 0 | 1 |
| ACIN1 | 3 | 0 | 0 | 0 | 0 | 0 | 0 | 0 | 1 |
| ZNF862 | 3 | 0 | 0 | 0 | 0 | 0 | 0 | 0 | 1 |

| | | | | | | | | | |
|---|---|---|---|---|---|---|---|---|---|
| ERCC6 | 3 | 0 | 0 | 0 | 0 | 0 | 0 | 0 | 1 |
| PHIP | 3 | 0 | 0 | 0 | 0 | 0 | 0 | 0 | 1 |
| TRPM2 | 3 | 0 | 0 | 0 | 0 | 0 | 0 | 0 | 1 |
| MTR | 3 | 0 | 0 | 0 | 0 | 0 | 0 | 0 | 1 |
| SPAM1 | 3 | 0 | 0 | 0 | 0 | 0 | 0 | 0 | 1 |
| UNC5C | 3 | 0 | 0 | 0 | 0 | 0 | 0 | 0 | 1 |
| SHPRH | 3 | 0 | 0 | 0 | 0 | 0 | 0 | 0 | 1 |
| MAST4 | 3 | 0 | 0 | 0 | 0 | 0 | 0 | 0 | 1 |
| MEGF8 | 2 | 1 | 0 | 0 | 0 | 0 | 0 | 0 | 1 |
| HNF1B | 3 | 0 | 0 | 0 | 0 | 0 | 0 | 0 | 1 |
| SAGE1 | 3 | 0 | 0 | 0 | 0 | 0 | 0 | 0 | 1 |
| IPO8 | 3 | 0 | 0 | 0 | 0 | 0 | 0 | 0 | 1 |
| IARS2 | 3 | 0 | 0 | 0 | 0 | 0 | 0 | 0 | 1 |
| SLAMF7 | 3 | 0 | 0 | 0 | 0 | 0 | 0 | 0 | 1 |
| PIK3CA | 3 | 0 | 0 | 0 | 0 | 0 | 0 | 0 | 1 |
| BAZ1A | 3 | 0 | 0 | 0 | 0 | 0 | 0 | 0 | 1 |
| NRIP1 | 3 | 0 | 0 | 0 | 0 | 0 | 0 | 0 | 1 |
| VWA2 | 3 | 0 | 0 | 0 | 0 | 0 | 0 | 0 | 1 |
| PIK3R4 | 3 | 0 | 0 | 0 | 0 | 0 | 0 | 0 | 1 |
| PRDM16 | 3 | 0 | 0 | 0 | 0 | 0 | 0 | 0 | 1 |
| KIAA1024 | 3 | 0 | 0 | 0 | 0 | 0 | 0 | 0 | 1 |
| MECOM | 3 | 0 | 0 | 0 | 0 | 0 | 0 | 0 | 1 |
| SLC1A1 | 2 | 1 | 0 | 0 | 0 | 0 | 0 | 0 | 1 |
| NLRP12 | 3 | 0 | 0 | 0 | 0 | 0 | 0 | 0 | 1 |
| PLEKHG3 | 3 | 0 | 0 | 0 | 0 | 0 | 0 | 0 | 1 |
| ACACA | 3 | 0 | 0 | 0 | 0 | 0 | 0 | 0 | 1 |
| BZRAP1 | 3 | 0 | 0 | 0 | 0 | 0 | 0 | 0 | 1 |
| RGNEF | 3 | 0 | 0 | 0 | 0 | 0 | 0 | 0 | 1 |
| MPDZ | 3 | 0 | 0 | 0 | 0 | 0 | 0 | 0 | 1 |
| MAP7D3 | 3 | 0 | 0 | 0 | 0 | 0 | 0 | 0 | 1 |
| OR6F1 | 2 | 0 | 0 | 0 | 1 | 0 | 0 | 0 | 1 |
| SNRNP200 | 2 | 1 | 0 | 0 | 0 | 0 | 0 | 0 | 1 |
| ASCC3 | 3 | 0 | 0 | 0 | 0 | 0 | 0 | 0 | 1 |
| SAMD9L | 3 | 0 | 0 | 0 | 0 | 0 | 0 | 0 | 1 |
| QSER1 | 3 | 0 | 0 | 0 | 0 | 0 | 0 | 0 | 1 |
| SYNRG | 3 | 0 | 0 | 0 | 0 | 0 | 0 | 0 | 1 |
| ITGB3 | 2 | 0 | 0 | 0 | 1 | 0 | 0 | 0 | 1 |
| OGDHL | 3 | 0 | 0 | 0 | 0 | 0 | 0 | 0 | 1 |
| TUBGCP5 | 3 | 0 | 0 | 0 | 0 | 0 | 0 | 0 | 1 |
| WDR62 | 3 | 0 | 0 | 0 | 0 | 0 | 0 | 0 | 1 |
| OR5T3 | 3 | 0 | 0 | 0 | 0 | 0 | 0 | 0 | 1 |
| NEURL4 | 2 | 0 | 1 | 0 | 0 | 0 | 0 | 0 | 1 |
| CYP2C19 | 3 | 0 | 0 | 0 | 0 | 0 | 0 | 0 | 1 |
| BLM | 1 | 0 | 1 | 0 | 0 | 1 | 0 | 0 | 1 |
| COL4A6 | 3 | 0 | 0 | 0 | 0 | 0 | 0 | 0 | 1 |
| SETD5 | 2 | 1 | 0 | 0 | 0 | 0 | 0 | 0 | 1 |

| | | | | | | | | | |
|---|---|---|---|---|---|---|---|---|---|
| TRDN | 3 | 0 | 0 | 0 | 0 | 0 | 0 | 0 | 1 |
| OR2T6 | 3 | 0 | 0 | 0 | 0 | 0 | 0 | 0 | 1 |
| PLXNB3 | 3 | 0 | 0 | 0 | 0 | 0 | 0 | 0 | 1 |
| HECTD1 | 3 | 0 | 0 | 0 | 0 | 0 | 0 | 0 | 1 |
| ARID4A | 3 | 0 | 0 | 0 | 0 | 0 | 0 | 0 | 1 |
| NCKAP5 | 3 | 0 | 0 | 0 | 0 | 0 | 0 | 0 | 1 |
| SEMA3A | 3 | 0 | 0 | 0 | 0 | 0 | 0 | 0 | 1 |
| EHMT1 | 3 | 0 | 0 | 0 | 0 | 0 | 0 | 0 | 1 |
| NTRK1 | 3 | 0 | 0 | 0 | 0 | 0 | 0 | 0 | 1 |
| ZBP1 | 3 | 0 | 0 | 0 | 0 | 0 | 0 | 0 | 1 |
| PCDH7 | 2 | 1 | 0 | 0 | 0 | 0 | 0 | 0 | 1 |
| DIP2C | 3 | 0 | 0 | 0 | 0 | 0 | 0 | 0 | 1 |
| HEATR1 | 2 | 0 | 0 | 0 | 1 | 0 | 0 | 0 | 1 |
| SETD1B | 3 | 0 | 0 | 0 | 0 | 0 | 0 | 0 | 1 |
| TRA2A | 1 | 2 | 0 | 0 | 0 | 0 | 0 | 0 | 1 |
| CNTLN | 3 | 0 | 0 | 0 | 0 | 0 | 0 | 0 | 1 |
| MED12 | 3 | 0 | 0 | 0 | 0 | 0 | 0 | 0 | 1 |
| FAM123B | 3 | 0 | 0 | 0 | 0 | 0 | 0 | 0 | 1 |
| MIB1 | 3 | 0 | 0 | 0 | 0 | 0 | 0 | 0 | 1 |
| CATSPER1 | 3 | 0 | 0 | 0 | 0 | 0 | 0 | 0 | 1 |
| OR8J3 | 3 | 0 | 0 | 0 | 0 | 0 | 0 | 0 | 1 |
| TTF2 | 3 | 0 | 0 | 0 | 0 | 0 | 0 | 0 | 1 |
| EPPK1 | 3 | 0 | 0 | 0 | 0 | 0 | 0 | 0 | 1 |
| C15orf53 | 3 | 0 | 0 | 0 | 0 | 0 | 0 | 0 | 1 |
| ARFGEF1 | 2 | 1 | 0 | 0 | 0 | 0 | 0 | 0 | 1 |
| GABBR2 | 3 | 0 | 0 | 0 | 0 | 0 | 0 | 0 | 1 |
| CNOT1 | 3 | 0 | 0 | 0 | 0 | 0 | 0 | 0 | 1 |
| NMNAT2 | 2 | 1 | 0 | 0 | 0 | 0 | 0 | 0 | 1 |
| FAM179B | 3 | 0 | 0 | 0 | 0 | 0 | 0 | 0 | 1 |
| PI4KA | 3 | 0 | 0 | 0 | 0 | 0 | 0 | 0 | 1 |
| LDB2 | 3 | 0 | 0 | 0 | 0 | 0 | 0 | 0 | 1 |
| CABYR | 3 | 0 | 0 | 0 | 0 | 0 | 0 | 0 | 1 |
| FLG | 3 | 0 | 0 | 0 | 0 | 0 | 0 | 0 | 1 |
| DISP1 | 3 | 0 | 0 | 0 | 0 | 0 | 0 | 0 | 1 |
| ATP8A1 | 1 | 2 | 0 | 0 | 0 | 0 | 0 | 0 | 1 |
| LPHN1 | 3 | 0 | 0 | 0 | 0 | 0 | 0 | 0 | 1 |
| PIWIL3 | 2 | 1 | 0 | 0 | 0 | 0 | 0 | 0 | 1 |
| MAP3K15 | 3 | 0 | 0 | 0 | 0 | 0 | 0 | 0 | 1 |
| PADI4 | 3 | 0 | 0 | 0 | 0 | 0 | 0 | 0 | 1 |
| NCAPD3 | 3 | 0 | 0 | 0 | 0 | 0 | 0 | 0 | 1 |
| PDE8B | 2 | 0 | 1 | 0 | 0 | 0 | 0 | 0 | 1 |
| MTOR | 3 | 0 | 0 | 0 | 0 | 0 | 0 | 0 | 1 |
| MAGI3 | 3 | 0 | 0 | 0 | 0 | 0 | 0 | 0 | 1 |
| KDM2B | 3 | 0 | 0 | 0 | 0 | 0 | 0 | 0 | 1 |
| DHDH | 3 | 0 | 0 | 0 | 0 | 0 | 0 | 0 | 1 |
| XRN2 | 2 | 1 | 0 | 0 | 0 | 0 | 0 | 0 | 1 |

| | | | | | | | | | |
|---|---|---|---|---|---|---|---|---|---|
| DYRK1B | 3 | 0 | 0 | 0 | 0 | 0 | 0 | 0 | 1 |
| LAD1 | 3 | 0 | 0 | 0 | 0 | 0 | 0 | 0 | 1 |
| FAM171B | 3 | 0 | 0 | 0 | 0 | 0 | 0 | 0 | 1 |
| FCRL2 | 3 | 0 | 0 | 0 | 0 | 0 | 0 | 0 | 1 |
| ODZ2 | 3 | 0 | 0 | 0 | 0 | 0 | 0 | 0 | 1 |
| ANKRD17 | 3 | 0 | 0 | 0 | 0 | 0 | 0 | 0 | 1 |
| GART | 3 | 0 | 0 | 0 | 0 | 0 | 0 | 0 | 1 |
| GAK | 3 | 0 | 0 | 0 | 0 | 0 | 0 | 0 | 1 |
| LTBP4 | 3 | 0 | 0 | 0 | 0 | 0 | 0 | 0 | 1 |
| C1RL | 3 | 0 | 0 | 0 | 0 | 0 | 0 | 0 | 1 |
| MRC2 | 2 | 0 | 0 | 0 | 1 | 0 | 0 | 0 | 1 |
| SETX | 3 | 0 | 0 | 0 | 0 | 0 | 0 | 0 | 1 |
| CYP2C9 | 3 | 0 | 0 | 0 | 0 | 0 | 0 | 0 | 1 |
| SECISBP2 | 3 | 0 | 0 | 0 | 0 | 0 | 0 | 0 | 1 |
| AKAP4 | 3 | 0 | 0 | 0 | 0 | 0 | 0 | 0 | 1 |
| IL13RA1 | 2 | 1 | 0 | 0 | 0 | 0 | 0 | 0 | 1 |
| PDS5B | 2 | 0 | 0 | 0 | 1 | 0 | 0 | 0 | 1 |
| NUP98 | 3 | 0 | 0 | 0 | 0 | 0 | 0 | 0 | 1 |
| KIAA0556 | 3 | 0 | 0 | 0 | 0 | 0 | 0 | 0 | 1 |
| UNC13B | 3 | 0 | 0 | 0 | 0 | 0 | 0 | 0 | 1 |
| THADA | 1 | 0 | 0 | 0 | 1 | 1 | 0 | 0 | 1 |
| ITPR1 | 1 | 2 | 0 | 0 | 0 | 0 | 0 | 0 | 1 |
| DIP2B | 3 | 0 | 0 | 0 | 0 | 0 | 0 | 0 | 1 |
| ZNF536 | 3 | 0 | 0 | 0 | 0 | 0 | 0 | 0 | 1 |
| FMO1 | 3 | 0 | 0 | 0 | 0 | 0 | 0 | 0 | 1 |
| CPD | 3 | 0 | 0 | 0 | 0 | 0 | 0 | 0 | 1 |
| MYO10 | 3 | 0 | 0 | 0 | 0 | 0 | 0 | 0 | 1 |
| CSNK1A1L | 3 | 0 | 0 | 0 | 0 | 0 | 0 | 0 | 1 |
| GRIA4 | 3 | 0 | 0 | 0 | 0 | 0 | 0 | 0 | 1 |
| DNAJC6 | 2 | 1 | 0 | 0 | 0 | 0 | 0 | 0 | 1 |
| AQR | 2 | 1 | 0 | 0 | 0 | 0 | 0 | 0 | 1 |
| NPSR1 | 3 | 0 | 0 | 0 | 0 | 0 | 0 | 0 | 1 |
| RCAN2 | 3 | 0 | 0 | 0 | 0 | 0 | 0 | 0 | 1 |
| TSHZ3 | 3 | 0 | 0 | 0 | 0 | 0 | 0 | 0 | 1 |
| IGF1R | 2 | 0 | 1 | 0 | 0 | 0 | 0 | 0 | 1 |
| HERC2 | 3 | 0 | 0 | 0 | 0 | 0 | 0 | 0 | 1 |
| SLC7A14 | 3 | 0 | 0 | 0 | 0 | 0 | 0 | 0 | 1 |
| SLC24A3 | 3 | 0 | 0 | 0 | 0 | 0 | 0 | 0 | 1 |
| SUPT16H | 2 | 0 | 0 | 0 | 1 | 0 | 0 | 0 | 1 |
| HNF4G | 3 | 0 | 0 | 0 | 0 | 0 | 0 | 0 | 1 |
| PAH | 3 | 0 | 0 | 0 | 0 | 0 | 0 | 0 | 1 |
| LEMD1 | 1 | 0 | 1 | 0 | 0 | 0 | 0 | 0 | 1 |
| PRDX1 | 0 | 2 | 0 | 0 | 0 | 0 | 0 | 0 | 1 |
| SELS | 1 | 0 | 1 | 0 | 0 | 0 | 0 | 0 | 1 |
| NINJ1 | 1 | 1 | 0 | 0 | 0 | 0 | 0 | 0 | 1 |
| ATPIF1 | 1 | 1 | 0 | 0 | 0 | 0 | 0 | 0 | 1 |

| | | | | | | | | | |
|---|---|---|---|---|---|---|---|---|---|
| RAB6B | 0 | 1 | 1 | 0 | 0 | 0 | 0 | 0 | 1 |
| CBX5 | 1 | 1 | 0 | 0 | 0 | 0 | 0 | 0 | 1 |
| SNRNP27 | 1 | 1 | 0 | 0 | 0 | 0 | 0 | 0 | 1 |
| MRPS18A | 1 | 1 | 0 | 0 | 0 | 0 | 0 | 0 | 1 |
| LCN6 | 1 | 1 | 0 | 0 | 0 | 0 | 0 | 0 | 1 |
| MED22 | 1 | 1 | 0 | 0 | 0 | 0 | 0 | 0 | 1 |
| C19orf10 | 1 | 0 | 0 | 0 | 0 | 0 | 1 | 0 | 1 |
| XAF1 | 0 | 2 | 0 | 0 | 0 | 0 | 0 | 0 | 1 |
| GML | 1 | 1 | 0 | 0 | 0 | 0 | 0 | 0 | 1 |
| CCDC152 | 0 | 1 | 1 | 0 | 0 | 0 | 0 | 0 | 1 |
| IL17F | 1 | 1 | 0 | 0 | 0 | 0 | 0 | 0 | 1 |
| CTDSP1 | 1 | 1 | 0 | 0 | 0 | 0 | 0 | 0 | 1 |
| EPO | 1 | 1 | 0 | 0 | 0 | 0 | 0 | 0 | 1 |
| TCL1B | 1 | 1 | 0 | 0 | 0 | 0 | 0 | 0 | 1 |
| TCF23 | 0 | 1 | 0 | 0 | 1 | 0 | 0 | 0 | 1 |
| SPINK4 | 2 | 0 | 0 | 0 | 0 | 0 | 0 | 0 | 1 |
| C10orf25 | 1 | 0 | 0 | 0 | 1 | 0 | 0 | 0 | 1 |
| CCDC53 | 1 | 1 | 0 | 0 | 0 | 0 | 0 | 0 | 1 |
| UBE2I | 1 | 1 | 0 | 0 | 0 | 0 | 0 | 0 | 1 |
| MED28 | 1 | 1 | 0 | 0 | 0 | 0 | 0 | 0 | 1 |
| CRISP2 | 1 | 0 | 1 | 0 | 0 | 0 | 0 | 0 | 1 |
| PCGF5 | 1 | 1 | 0 | 0 | 0 | 0 | 0 | 0 | 1 |
| ACER2 | 1 | 1 | 0 | 0 | 0 | 0 | 0 | 0 | 1 |
| PRSS27 | 1 | 0 | 1 | 0 | 0 | 0 | 0 | 0 | 1 |
| CSTB | 2 | 0 | 0 | 0 | 0 | 0 | 0 | 0 | 1 |
| C12orf60 | 1 | 0 | 0 | 1 | 0 | 0 | 0 | 0 | 1 |
| TRMT112 | 2 | 0 | 0 | 0 | 0 | 0 | 0 | 0 | 1 |
| IAH1 | 1 | 1 | 0 | 0 | 0 | 0 | 0 | 0 | 1 |
| CDV3 | 1 | 0 | 1 | 0 | 0 | 0 | 0 | 0 | 1 |
| MED10 | 1 | 1 | 0 | 0 | 0 | 0 | 0 | 0 | 1 |
| SLC25A10 | 1 | 1 | 0 | 0 | 0 | 0 | 0 | 0 | 1 |
| KCNMB4 | 1 | 1 | 0 | 0 | 0 | 0 | 0 | 0 | 1 |
| C6orf225 | 2 | 0 | 0 | 0 | 0 | 0 | 0 | 0 | 1 |
| RPS21 | 2 | 0 | 0 | 0 | 0 | 0 | 0 | 0 | 1 |
| C6orf223 | 1 | 1 | 0 | 0 | 0 | 0 | 0 | 0 | 1 |
| HIST1H2BD | 2 | 0 | 0 | 0 | 0 | 0 | 0 | 0 | 1 |
| LYPD5 | 1 | 1 | 0 | 0 | 0 | 0 | 0 | 0 | 1 |
| DEFB125 | 1 | 0 | 1 | 0 | 0 | 0 | 0 | 0 | 1 |
| GNLY | 2 | 0 | 0 | 0 | 0 | 0 | 0 | 0 | 1 |
| ARFIP1 | 1 | 1 | 0 | 0 | 0 | 0 | 0 | 0 | 1 |
| IL17B | 1 | 1 | 0 | 0 | 0 | 0 | 0 | 0 | 1 |
| VDAC3 | 1 | 1 | 0 | 0 | 0 | 0 | 0 | 0 | 1 |
| S100A3 | 2 | 0 | 0 | 0 | 0 | 0 | 0 | 0 | 1 |
| PPM1D | 0 | 2 | 0 | 0 | 0 | 0 | 0 | 0 | 1 |
| COQ10B | 1 | 1 | 0 | 0 | 0 | 0 | 0 | 0 | 1 |
| RPE65 | 0 | 1 | 1 | 0 | 0 | 0 | 0 | 0 | 1 |

| | | | | | | | | | |
|---|---|---|---|---|---|---|---|---|---|
| VPS28 | 1 | 1 | 0 | 0 | 0 | 0 | 0 | 0 | 1 |
| LILRA5 | 1 | 1 | 0 | 0 | 0 | 0 | 0 | 0 | 1 |
| RPL35A | 2 | 0 | 0 | 0 | 0 | 0 | 0 | 0 | 1 |
| CTSK | 1 | 1 | 0 | 0 | 0 | 0 | 0 | 0 | 1 |
| C19orf42 | 2 | 0 | 0 | 0 | 0 | 0 | 0 | 0 | 1 |
| ALG3 | 1 | 1 | 0 | 0 | 0 | 0 | 0 | 0 | 1 |
| GPHA2 | 2 | 0 | 0 | 0 | 0 | 0 | 0 | 0 | 1 |
| FLJ25363 | 2 | 0 | 0 | 0 | 0 | 0 | 0 | 0 | 1 |
| CLEC12A | 2 | 0 | 0 | 0 | 0 | 0 | 0 | 0 | 1 |
| HMGA2 | 2 | 0 | 0 | 0 | 0 | 0 | 0 | 0 | 1 |
| CD300LD | 1 | 1 | 0 | 0 | 0 | 0 | 0 | 0 | 1 |
| PKIB | 2 | 0 | 0 | 0 | 0 | 0 | 0 | 0 | 1 |
| TOR1A | 1 | 1 | 0 | 0 | 0 | 0 | 0 | 0 | 1 |
| ISCU | 2 | 0 | 0 | 0 | 0 | 0 | 0 | 0 | 1 |
| SAMD13 | 1 | 1 | 0 | 0 | 0 | 0 | 0 | 0 | 1 |
| MSMB | 2 | 0 | 0 | 0 | 0 | 0 | 0 | 0 | 1 |
| TAAR8 | 1 | 1 | 0 | 0 | 0 | 0 | 0 | 0 | 1 |
| KCNIP3 | 1 | 0 | 1 | 0 | 0 | 0 | 0 | 0 | 1 |
| OSR2 | 1 | 1 | 0 | 0 | 0 | 0 | 0 | 0 | 1 |
| PHF11 | 1 | 0 | 1 | 0 | 0 | 0 | 0 | 0 | 1 |
| RNF144B | 0 | 1 | 0 | 0 | 1 | 0 | 0 | 0 | 1 |
| CIDEB | 1 | 0 | 0 | 0 | 1 | 0 | 0 | 0 | 1 |
| REXO2 | 1 | 1 | 0 | 0 | 0 | 0 | 0 | 0 | 1 |
| SST | 2 | 0 | 0 | 0 | 0 | 0 | 0 | 0 | 1 |
| HIST1H2AH | 2 | 0 | 0 | 0 | 0 | 0 | 0 | 0 | 1 |
| PRDX5 | 2 | 0 | 0 | 0 | 0 | 0 | 0 | 0 | 1 |
| BCL2L15 | 2 | 0 | 0 | 0 | 0 | 0 | 0 | 0 | 1 |
| PTP4A2 | 2 | 0 | 0 | 0 | 0 | 0 | 0 | 0 | 1 |
| P4HB | 0 | 0 | 1 | 0 | 1 | 0 | 0 | 0 | 1 |
| CLEC1A | 1 | 1 | 0 | 0 | 0 | 0 | 0 | 0 | 1 |
| GPR141 | 1 | 1 | 0 | 0 | 0 | 0 | 0 | 0 | 1 |
| OR5AP2 | 1 | 1 | 0 | 0 | 0 | 0 | 0 | 0 | 1 |
| PRPF19 | 1 | 1 | 0 | 0 | 0 | 0 | 0 | 0 | 1 |
| SEC14L4 | 1 | 0 | 1 | 0 | 0 | 0 | 0 | 0 | 1 |
| PYDC2 | 2 | 0 | 0 | 0 | 0 | 0 | 0 | 0 | 1 |
| ODF4 | 1 | 1 | 0 | 0 | 0 | 0 | 0 | 0 | 1 |
| LAT2 | 1 | 1 | 0 | 0 | 0 | 0 | 0 | 0 | 1 |
| FNTA | 1 | 1 | 0 | 0 | 0 | 0 | 0 | 0 | 1 |
| HRH4 | 1 | 1 | 0 | 0 | 0 | 0 | 0 | 0 | 1 |
| PPIB | 2 | 0 | 0 | 0 | 0 | 0 | 0 | 0 | 1 |
| TAS2R10 | 1 | 1 | 0 | 0 | 0 | 0 | 0 | 0 | 1 |
| PPPDE2 | 2 | 0 | 0 | 0 | 0 | 0 | 0 | 0 | 1 |
| MAF1 | 2 | 0 | 0 | 0 | 0 | 0 | 0 | 0 | 1 |
| C3orf55 | 1 | 0 | 1 | 0 | 0 | 0 | 0 | 0 | 1 |
| TAF7L | 1 | 1 | 0 | 0 | 0 | 0 | 0 | 0 | 1 |
| TAS2R9 | 1 | 1 | 0 | 0 | 0 | 0 | 0 | 0 | 1 |

| | | | | | | | | | |
|---|---|---|---|---|---|---|---|---|---|
| PRPS1L1 | 1 | 1 | 0 | 0 | 0 | 0 | 0 | 0 | 1 |
| AMMECR1 | 1 | 1 | 0 | 0 | 0 | 0 | 0 | 0 | 1 |
| IGFBPL1 | 1 | 1 | 0 | 0 | 0 | 0 | 0 | 0 | 1 |
| GCNT7 | 1 | 1 | 0 | 0 | 0 | 0 | 0 | 0 | 1 |
| TIGD7 | 1 | 1 | 0 | 0 | 0 | 0 | 0 | 0 | 1 |
| PSMD9 | 2 | 0 | 0 | 0 | 0 | 0 | 0 | 0 | 1 |
| BATF3 | 2 | 0 | 0 | 0 | 0 | 0 | 0 | 0 | 1 |
| OTOS | 2 | 0 | 0 | 0 | 0 | 0 | 0 | 0 | 1 |
| RHOD | 2 | 0 | 0 | 0 | 0 | 0 | 0 | 0 | 1 |
| GM2A | 2 | 0 | 0 | 0 | 0 | 0 | 0 | 0 | 1 |
| LMO4 | 2 | 0 | 0 | 0 | 0 | 0 | 0 | 0 | 1 |
| TEC | 0 | 2 | 0 | 0 | 0 | 0 | 0 | 0 | 1 |
| TROVE2 | 1 | 1 | 0 | 0 | 0 | 0 | 0 | 0 | 1 |
| NAA10 | 2 | 0 | 0 | 0 | 0 | 0 | 0 | 0 | 1 |
| SDHAF2 | 2 | 0 | 0 | 0 | 0 | 0 | 0 | 0 | 1 |
| AIF1 | 2 | 0 | 0 | 0 | 0 | 0 | 0 | 0 | 1 |
| GJB7 | 1 | 1 | 0 | 0 | 0 | 0 | 0 | 0 | 1 |
| GZMK | 1 | 1 | 0 | 0 | 0 | 0 | 0 | 0 | 1 |
| KLRC1 | 2 | 0 | 0 | 0 | 0 | 0 | 0 | 0 | 1 |
| PDLIM1 | 1 | 1 | 0 | 0 | 0 | 0 | 0 | 0 | 1 |
| PNOC | 2 | 0 | 0 | 0 | 0 | 0 | 0 | 0 | 1 |
| HIST1H2BB | 2 | 0 | 0 | 0 | 0 | 0 | 0 | 0 | 1 |
| CABP7 | 2 | 0 | 0 | 0 | 0 | 0 | 0 | 0 | 1 |
| C1orf51 | 1 | 1 | 0 | 0 | 0 | 0 | 0 | 0 | 1 |
| FCN1 | 1 | 1 | 0 | 0 | 0 | 0 | 0 | 0 | 1 |
| KRAS | 1 | 0 | 0 | 0 | 1 | 0 | 0 | 0 | 1 |
| TMEM100 | 2 | 0 | 0 | 0 | 0 | 0 | 0 | 0 | 1 |
| TRIM44 | 1 | 1 | 0 | 0 | 0 | 0 | 0 | 0 | 1 |
| SCAMP3 | 1 | 1 | 0 | 0 | 0 | 0 | 0 | 0 | 1 |
| C7orf65 | 2 | 0 | 0 | 0 | 0 | 0 | 0 | 0 | 1 |
| MYOT | 1 | 1 | 0 | 0 | 0 | 0 | 0 | 0 | 1 |
| FAM158A | 2 | 0 | 0 | 0 | 0 | 0 | 0 | 0 | 1 |
| KRTAP19-1 | 2 | 0 | 0 | 0 | 0 | 0 | 0 | 0 | 1 |
| ARL14 | 2 | 0 | 0 | 0 | 0 | 0 | 0 | 0 | 1 |
| SLC22A15 | 1 | 1 | 0 | 0 | 0 | 0 | 0 | 0 | 1 |
| CYP1A1 | 1 | 1 | 0 | 0 | 0 | 0 | 0 | 0 | 1 |
| TMEM211 | 2 | 0 | 0 | 0 | 0 | 0 | 0 | 0 | 1 |
| SHISA3 | 2 | 0 | 0 | 0 | 0 | 0 | 0 | 0 | 1 |
| LITAF | 2 | 0 | 0 | 0 | 0 | 0 | 0 | 0 | 1 |
| CLEC2A | 2 | 0 | 0 | 0 | 0 | 0 | 0 | 0 | 1 |
| IGFBP7 | 1 | 0 | 1 | 0 | 0 | 0 | 0 | 0 | 1 |
| RNPC3 | 2 | 0 | 0 | 0 | 0 | 0 | 0 | 0 | 1 |
| FIP1L1 | 1 | 0 | 1 | 0 | 0 | 0 | 0 | 0 | 1 |
| C11orf70 | 2 | 0 | 0 | 0 | 0 | 0 | 0 | 0 | 1 |
| RPS9 | 2 | 0 | 0 | 0 | 0 | 0 | 0 | 0 | 1 |
| OR6K6 | 1 | 1 | 0 | 0 | 0 | 0 | 0 | 0 | 1 |

| | | | | | | | | | |
|---|---|---|---|---|---|---|---|---|---|
| PMP22 | 2 | 0 | 0 | 0 | 0 | 0 | 0 | 0 | 1 |
| FAM3B | 2 | 0 | 0 | 0 | 0 | 0 | 0 | 0 | 1 |
| TK1 | 2 | 0 | 0 | 0 | 0 | 0 | 0 | 0 | 1 |
| ATP6V1E1 | 2 | 0 | 0 | 0 | 0 | 0 | 0 | 0 | 1 |
| OR2Z1 | 1 | 1 | 0 | 0 | 0 | 0 | 0 | 0 | 1 |
| IL19 | 2 | 0 | 0 | 0 | 0 | 0 | 0 | 0 | 1 |
| OR2A14 | 1 | 1 | 0 | 0 | 0 | 0 | 0 | 0 | 1 |
| TMEM207 | 2 | 0 | 0 | 0 | 0 | 0 | 0 | 0 | 1 |
| FAM19A1 | 2 | 0 | 0 | 0 | 0 | 0 | 0 | 0 | 1 |
| CCDC134 | 2 | 0 | 0 | 0 | 0 | 0 | 0 | 0 | 1 |
| ERGIC1 | 2 | 0 | 0 | 0 | 0 | 0 | 0 | 0 | 1 |
| C4orf26 | 2 | 0 | 0 | 0 | 0 | 0 | 0 | 0 | 1 |
| RDBP | 1 | 1 | 0 | 0 | 0 | 0 | 0 | 0 | 1 |
| ICAM4 | 1 | 1 | 0 | 0 | 0 | 0 | 0 | 0 | 1 |
| C1orf64 | 2 | 0 | 0 | 0 | 0 | 0 | 0 | 0 | 1 |
| DHRS7C | 1 | 1 | 0 | 0 | 0 | 0 | 0 | 0 | 1 |
| SULT2B1 | 1 | 0 | 1 | 0 | 0 | 0 | 0 | 0 | 1 |
| CCL16 | 2 | 0 | 0 | 0 | 0 | 0 | 0 | 0 | 1 |
| TRAM1 | 1 | 1 | 0 | 0 | 0 | 0 | 0 | 0 | 1 |
| SPI1 | 2 | 0 | 0 | 0 | 0 | 0 | 0 | 0 | 1 |
| FGFBP2 | 2 | 0 | 0 | 0 | 0 | 0 | 0 | 0 | 1 |
| ACTR2 | 1 | 0 | 0 | 0 | 1 | 0 | 0 | 0 | 1 |
| SEC14L3 | 1 | 1 | 0 | 0 | 0 | 0 | 0 | 0 | 1 |
| ACAA2 | 1 | 0 | 1 | 0 | 0 | 0 | 0 | 0 | 1 |
| CCDC65 | 1 | 0 | 1 | 0 | 0 | 0 | 0 | 0 | 1 |
| ASTL | 1 | 1 | 0 | 0 | 0 | 0 | 0 | 0 | 1 |
| NDUFV1 | 1 | 1 | 0 | 0 | 0 | 0 | 0 | 0 | 1 |
| MMP26 | 1 | 1 | 0 | 0 | 0 | 0 | 0 | 0 | 1 |
| GALNTL5 | 1 | 1 | 0 | 0 | 0 | 0 | 0 | 0 | 1 |
| TFPI | 1 | 1 | 0 | 0 | 0 | 0 | 0 | 0 | 1 |
| GUCA1B | 2 | 0 | 0 | 0 | 0 | 0 | 0 | 0 | 1 |
| DFNA5 | 1 | 1 | 0 | 0 | 0 | 0 | 0 | 0 | 1 |
| PRRX1 | 1 | 1 | 0 | 0 | 0 | 0 | 0 | 0 | 1 |
| TCP10L | 1 | 0 | 0 | 0 | 1 | 0 | 0 | 0 | 1 |
| STMN4 | 2 | 0 | 0 | 0 | 0 | 0 | 0 | 0 | 1 |
| LGALS14 | 2 | 0 | 0 | 0 | 0 | 0 | 0 | 0 | 1 |
| TNFRSF1B | 1 | 1 | 0 | 0 | 0 | 0 | 0 | 0 | 1 |
| LIPM | 1 | 1 | 0 | 0 | 0 | 0 | 0 | 0 | 1 |
| NEIL3 | 0 | 2 | 0 | 0 | 0 | 0 | 0 | 0 | 1 |
| ERCC1 | 2 | 0 | 0 | 0 | 0 | 0 | 0 | 0 | 1 |
| CARTPT | 2 | 0 | 0 | 0 | 0 | 0 | 0 | 0 | 1 |
| DBR1 | 1 | 0 | 1 | 0 | 0 | 0 | 0 | 0 | 1 |
| GJA9 | 1 | 1 | 0 | 0 | 0 | 0 | 0 | 0 | 1 |
| SSR1 | 2 | 0 | 0 | 0 | 0 | 0 | 0 | 0 | 1 |
| ANKRD13C | 1 | 1 | 0 | 0 | 0 | 0 | 0 | 0 | 1 |
| TRAF1 | 2 | 0 | 0 | 0 | 0 | 0 | 0 | 0 | 1 |

| | | | | | | | | | |
|---|---|---|---|---|---|---|---|---|---|
| AADACL2 | 1 | 1 | 0 | 0 | 0 | 0 | 0 | 0 | 1 |
| SIGLEC12 | 1 | 1 | 0 | 0 | 0 | 0 | 0 | 0 | 1 |
| KRTAP8-1 | 2 | 0 | 0 | 0 | 0 | 0 | 0 | 0 | 1 |
| FAM3D | 2 | 0 | 0 | 0 | 0 | 0 | 0 | 0 | 1 |
| LIPH | 1 | 1 | 0 | 0 | 0 | 0 | 0 | 0 | 1 |
| ODF3 | 2 | 0 | 0 | 0 | 0 | 0 | 0 | 0 | 1 |
| PPP1R3B | 2 | 0 | 0 | 0 | 0 | 0 | 0 | 0 | 1 |
| HOXC9 | 2 | 0 | 0 | 0 | 0 | 0 | 0 | 0 | 1 |
| VEZF1 | 1 | 1 | 0 | 0 | 0 | 0 | 0 | 0 | 1 |
| LARP4 | 1 | 1 | 0 | 0 | 0 | 0 | 0 | 0 | 1 |
| NPLOC4 | 1 | 1 | 0 | 0 | 0 | 0 | 0 | 0 | 1 |
| OR4P4 | 1 | 1 | 0 | 0 | 0 | 0 | 0 | 0 | 1 |
| PRG3 | 2 | 0 | 0 | 0 | 0 | 0 | 0 | 0 | 1 |
| RGS4 | 1 | 1 | 0 | 0 | 0 | 0 | 0 | 0 | 1 |
| OR8U8 | 2 | 0 | 0 | 0 | 0 | 0 | 0 | 0 | 1 |
| LCE2C | 2 | 0 | 0 | 0 | 0 | 0 | 0 | 0 | 1 |
| NETO2 | 1 | 1 | 0 | 0 | 0 | 0 | 0 | 0 | 1 |
| NMS | 2 | 0 | 0 | 0 | 0 | 0 | 0 | 0 | 1 |
| CNTFR | 1 | 1 | 0 | 0 | 0 | 0 | 0 | 0 | 1 |
| CD83 | 2 | 0 | 0 | 0 | 0 | 0 | 0 | 0 | 1 |
| TMCO6 | 1 | 1 | 0 | 0 | 0 | 0 | 0 | 0 | 1 |
| MRPS2 | 2 | 0 | 0 | 0 | 0 | 0 | 0 | 0 | 1 |
| IL22RA2 | 2 | 0 | 0 | 0 | 0 | 0 | 0 | 0 | 1 |
| KLHL38 | 1 | 0 | 1 | 0 | 0 | 0 | 0 | 0 | 1 |
| CCDC70 | 2 | 0 | 0 | 0 | 0 | 0 | 0 | 0 | 1 |
| NUDT6 | 2 | 0 | 0 | 0 | 0 | 0 | 0 | 0 | 1 |
| DNAJB12 | 2 | 0 | 0 | 0 | 0 | 0 | 0 | 0 | 1 |
| GGA1 | 0 | 1 | 1 | 0 | 0 | 0 | 0 | 0 | 1 |
| CCDC68 | 2 | 0 | 0 | 0 | 0 | 0 | 0 | 0 | 1 |
| CPB2 | 1 | 1 | 0 | 0 | 0 | 0 | 0 | 0 | 1 |
| TMEM5 | 1 | 1 | 0 | 0 | 0 | 0 | 0 | 0 | 1 |
| UBXN2A | 2 | 0 | 0 | 0 | 0 | 0 | 0 | 0 | 1 |
| PCDHB4 | 0 | 2 | 0 | 0 | 0 | 0 | 0 | 0 | 1 |
| TSTD2 | 1 | 1 | 0 | 0 | 0 | 0 | 0 | 0 | 1 |
| C2orf73 | 2 | 0 | 0 | 0 | 0 | 0 | 0 | 0 | 1 |
| TOMM40L | 2 | 0 | 0 | 0 | 0 | 0 | 0 | 0 | 1 |
| PIGT | 1 | 0 | 1 | 0 | 0 | 0 | 0 | 0 | 1 |
| WAS | 1 | 0 | 1 | 0 | 0 | 0 | 0 | 0 | 1 |
| OR6Q1 | 2 | 0 | 0 | 0 | 0 | 0 | 0 | 0 | 1 |
| SETD8 | 2 | 0 | 0 | 0 | 0 | 0 | 0 | 0 | 1 |
| U2AF1L4 | 2 | 0 | 0 | 0 | 0 | 0 | 0 | 0 | 1 |
| IVD | 2 | 0 | 0 | 0 | 0 | 0 | 0 | 0 | 1 |
| GEM | 2 | 0 | 0 | 0 | 0 | 0 | 0 | 0 | 1 |
| PDSS1 | 2 | 0 | 0 | 0 | 0 | 0 | 0 | 0 | 1 |
| FAM19A3 | 2 | 0 | 0 | 0 | 0 | 0 | 0 | 0 | 1 |
| MYL10 | 2 | 0 | 0 | 0 | 0 | 0 | 0 | 0 | 1 |

| | | | | | | | | | |
|---|---|---|---|---|---|---|---|---|---|
| PPP1CB | 2 | 0 | 0 | 0 | 0 | 0 | 0 | 0 | 1 |
| TAS2R46 | 1 | 0 | 0 | 0 | 1 | 0 | 0 | 0 | 1 |
| FAM149A | 1 | 1 | 0 | 0 | 0 | 0 | 0 | 0 | 1 |
| FABP6 | 2 | 0 | 0 | 0 | 0 | 0 | 0 | 0 | 1 |
| RNASE9 | 2 | 0 | 0 | 0 | 0 | 0 | 0 | 0 | 1 |
| C8orf45 | 1 | 1 | 0 | 0 | 0 | 0 | 0 | 0 | 1 |
| SUCNR1 | 2 | 0 | 0 | 0 | 0 | 0 | 0 | 0 | 1 |
| NT5C3 | 2 | 0 | 0 | 0 | 0 | 0 | 0 | 0 | 1 |
| BHMT | 1 | 0 | 1 | 0 | 0 | 0 | 0 | 0 | 1 |
| SYCP3 | 2 | 0 | 0 | 0 | 0 | 0 | 0 | 0 | 1 |
| FBLN7 | 1 | 0 | 1 | 0 | 0 | 0 | 0 | 0 | 1 |
| PDGFB | 2 | 0 | 0 | 0 | 0 | 0 | 0 | 0 | 1 |
| PCGF1 | 2 | 0 | 0 | 0 | 0 | 0 | 0 | 0 | 1 |
| EXD2 | 1 | 1 | 0 | 0 | 0 | 0 | 0 | 0 | 1 |
| POU1F1 | 1 | 1 | 0 | 0 | 0 | 0 | 0 | 0 | 1 |
| LAIR1 | 2 | 0 | 0 | 0 | 0 | 0 | 0 | 0 | 1 |
| ZMYM5 | 1 | 1 | 0 | 0 | 0 | 0 | 0 | 0 | 1 |
| C6orf221 | 2 | 0 | 0 | 0 | 0 | 0 | 0 | 0 | 1 |
| PLEKHA1 | 1 | 1 | 0 | 0 | 0 | 0 | 0 | 0 | 1 |
| DDX53 | 1 | 1 | 0 | 0 | 0 | 0 | 0 | 0 | 1 |
| CLEC4C | 2 | 0 | 0 | 0 | 0 | 0 | 0 | 0 | 1 |
| C1orf88 | 2 | 0 | 0 | 0 | 0 | 0 | 0 | 0 | 1 |
| KLF9 | 2 | 0 | 0 | 0 | 0 | 0 | 0 | 0 | 1 |
| CRYGA | 2 | 0 | 0 | 0 | 0 | 0 | 0 | 0 | 1 |
| SH3BGR | 2 | 0 | 0 | 0 | 0 | 0 | 0 | 0 | 1 |
| CMA1 | 2 | 0 | 0 | 0 | 0 | 0 | 0 | 0 | 1 |
| THAP6 | 2 | 0 | 0 | 0 | 0 | 0 | 0 | 0 | 1 |
| LYG2 | 2 | 0 | 0 | 0 | 0 | 0 | 0 | 0 | 1 |
| PARP16 | 2 | 0 | 0 | 0 | 0 | 0 | 0 | 0 | 1 |
| UGCG | 2 | 0 | 0 | 0 | 0 | 0 | 0 | 0 | 1 |
| LETMD1 | 2 | 0 | 0 | 0 | 0 | 0 | 0 | 0 | 1 |
| GJD2 | 2 | 0 | 0 | 0 | 0 | 0 | 0 | 0 | 1 |
| CHMP4C | 2 | 0 | 0 | 0 | 0 | 0 | 0 | 0 | 1 |
| TATDN1 | 2 | 0 | 0 | 0 | 0 | 0 | 0 | 0 | 1 |
| KRT28 | 1 | 1 | 0 | 0 | 0 | 0 | 0 | 0 | 1 |
| C15orf23 | 2 | 0 | 0 | 0 | 0 | 0 | 0 | 0 | 1 |
| KLF7 | 2 | 0 | 0 | 0 | 0 | 0 | 0 | 0 | 1 |
| HEY2 | 2 | 0 | 0 | 0 | 0 | 0 | 0 | 0 | 1 |
| SARS2 | 1 | 1 | 0 | 0 | 0 | 0 | 0 | 0 | 1 |
| CCBL1 | 2 | 0 | 0 | 0 | 0 | 0 | 0 | 0 | 1 |
| BCL7A | 2 | 0 | 0 | 0 | 0 | 0 | 0 | 0 | 1 |
| ASPDH | 2 | 0 | 0 | 0 | 0 | 0 | 0 | 0 | 1 |
| TDP2 | 2 | 0 | 0 | 0 | 0 | 0 | 0 | 0 | 1 |
| MUS81 | 1 | 0 | 1 | 0 | 0 | 0 | 0 | 0 | 1 |
| FBXL18 | 1 | 0 | 0 | 1 | 0 | 0 | 0 | 0 | 1 |
| SCD | 2 | 0 | 0 | 0 | 0 | 0 | 0 | 0 | 1 |

| | | | | | | | | | |
|---|---|---|---|---|---|---|---|---|---|
| FKBP8 | 2 | 0 | 0 | 0 | 0 | 0 | 0 | 0 | 1 |
| TREX1 | 2 | 0 | 0 | 0 | 0 | 0 | 0 | 0 | 1 |
| SLC17A5 | 1 | 1 | 0 | 0 | 0 | 0 | 0 | 0 | 1 |
| RPP30 | 2 | 0 | 0 | 0 | 0 | 0 | 0 | 0 | 1 |
| NR1I2 | 1 | 1 | 0 | 0 | 0 | 0 | 0 | 0 | 1 |
| DOK4 | 2 | 0 | 0 | 0 | 0 | 0 | 0 | 0 | 1 |
| PSMA8 | 2 | 0 | 0 | 0 | 0 | 0 | 0 | 0 | 1 |
| OLA1 | 2 | 0 | 0 | 0 | 0 | 0 | 0 | 0 | 1 |
| APOBEC1 | 2 | 0 | 0 | 0 | 0 | 0 | 0 | 0 | 1 |
| LSR | 0 | 2 | 0 | 0 | 0 | 0 | 0 | 0 | 1 |
| OR6X1 | 2 | 0 | 0 | 0 | 0 | 0 | 0 | 0 | 1 |
| GPR77 | 2 | 0 | 0 | 0 | 0 | 0 | 0 | 0 | 1 |
| TAS2R39 | 2 | 0 | 0 | 0 | 0 | 0 | 0 | 0 | 1 |
| SNRNP35 | 2 | 0 | 0 | 0 | 0 | 0 | 0 | 0 | 1 |
| ZNF436 | 1 | 1 | 0 | 0 | 0 | 0 | 0 | 0 | 1 |
| ZDHHC15 | 1 | 1 | 0 | 0 | 0 | 0 | 0 | 0 | 1 |
| SLC25A11 | 2 | 0 | 0 | 0 | 0 | 0 | 0 | 0 | 1 |
| TINF2 | 2 | 0 | 0 | 0 | 0 | 0 | 0 | 0 | 1 |
| DNASE1L3 | 2 | 0 | 0 | 0 | 0 | 0 | 0 | 0 | 1 |
| PRICKLE4 | 2 | 0 | 0 | 0 | 0 | 0 | 0 | 0 | 1 |
| HDAC8 | 2 | 0 | 0 | 0 | 0 | 0 | 0 | 0 | 1 |
| NMNAT1 | 2 | 0 | 0 | 0 | 0 | 0 | 0 | 0 | 1 |
| TSGA10IP | 1 | 1 | 0 | 0 | 0 | 0 | 0 | 0 | 1 |
| C1orf49 | 2 | 0 | 0 | 0 | 0 | 0 | 0 | 0 | 1 |
| GMCL1 | 2 | 0 | 0 | 0 | 0 | 0 | 0 | 0 | 1 |
| NT5C2 | 1 | 0 | 1 | 0 | 0 | 0 | 0 | 0 | 1 |
| POLR3H | 2 | 0 | 0 | 0 | 0 | 0 | 0 | 0 | 1 |
| CEACAM4 | 2 | 0 | 0 | 0 | 0 | 0 | 0 | 0 | 1 |
| IDS | 1 | 1 | 0 | 0 | 0 | 0 | 0 | 0 | 1 |
| NXF1 | 1 | 0 | 1 | 0 | 0 | 0 | 0 | 0 | 1 |
| SLC5A6 | 1 | 1 | 0 | 0 | 0 | 0 | 0 | 0 | 1 |
| MCM3 | 1 | 1 | 0 | 0 | 0 | 0 | 0 | 0 | 1 |
| DNTTIP1 | 2 | 0 | 0 | 0 | 0 | 0 | 0 | 0 | 1 |
| C10orf11 | 2 | 0 | 0 | 0 | 0 | 0 | 0 | 0 | 1 |
| MECR | 2 | 0 | 0 | 0 | 0 | 0 | 0 | 0 | 1 |
| ZMPSTE24 | 2 | 0 | 0 | 0 | 0 | 0 | 0 | 0 | 1 |
| GAL3ST1 | 2 | 0 | 0 | 0 | 0 | 0 | 0 | 0 | 1 |
| OR10H1 | 2 | 0 | 0 | 0 | 0 | 0 | 0 | 0 | 1 |
| UNC50 | 2 | 0 | 0 | 0 | 0 | 0 | 0 | 0 | 1 |
| C7orf42 | 2 | 0 | 0 | 0 | 0 | 0 | 0 | 0 | 1 |
| EIF3H | 2 | 0 | 0 | 0 | 0 | 0 | 0 | 0 | 1 |
| F11R | 2 | 0 | 0 | 0 | 0 | 0 | 0 | 0 | 1 |
| MRO | 2 | 0 | 0 | 0 | 0 | 0 | 0 | 0 | 1 |
| GLT8D2 | 2 | 0 | 0 | 0 | 0 | 0 | 0 | 0 | 1 |
| DQX1 | 1 | 0 | 1 | 0 | 0 | 0 | 0 | 0 | 1 |
| FUT1 | 2 | 0 | 0 | 0 | 0 | 0 | 0 | 0 | 1 |

| | | | | | | | | | |
|---|---|---|---|---|---|---|---|---|---|
| BNIPL | 2 | 0 | 0 | 0 | 0 | 0 | 0 | 0 | 1 |
| DERL3 | 2 | 0 | 0 | 0 | 0 | 0 | 0 | 0 | 1 |
| OR51B6 | 2 | 0 | 0 | 0 | 0 | 0 | 0 | 0 | 1 |
| NR1I3 | 1 | 0 | 0 | 0 | 1 | 0 | 0 | 0 | 1 |
| WNT5B | 2 | 0 | 0 | 0 | 0 | 0 | 0 | 0 | 1 |
| KLHDC3 | 2 | 0 | 0 | 0 | 0 | 0 | 0 | 0 | 1 |
| HSD17B6 | 2 | 0 | 0 | 0 | 0 | 0 | 0 | 0 | 1 |
| OR5M9 | 2 | 0 | 0 | 0 | 0 | 0 | 0 | 0 | 1 |
| TTLL10 | 1 | 1 | 0 | 0 | 0 | 0 | 0 | 0 | 1 |
| SLAMF8 | 2 | 0 | 0 | 0 | 0 | 0 | 0 | 0 | 1 |
| PI4K2A | 1 | 1 | 0 | 0 | 0 | 0 | 0 | 0 | 1 |
| FUBP1 | 1 | 1 | 0 | 0 | 0 | 0 | 0 | 0 | 1 |
| STMN2 | 2 | 0 | 0 | 0 | 0 | 0 | 0 | 0 | 1 |
| KPTN | 1 | 1 | 0 | 0 | 0 | 0 | 0 | 0 | 1 |
| MYL6B | 2 | 0 | 0 | 0 | 0 | 0 | 0 | 0 | 1 |
| CHMP4B | 2 | 0 | 0 | 0 | 0 | 0 | 0 | 0 | 1 |
| GRAMD2 | 2 | 0 | 0 | 0 | 0 | 0 | 0 | 0 | 1 |
| FAM199X | 2 | 0 | 0 | 0 | 0 | 0 | 0 | 0 | 1 |
| PTPLA | 2 | 0 | 0 | 0 | 0 | 0 | 0 | 0 | 1 |
| DUSP22 | 2 | 0 | 0 | 0 | 0 | 0 | 0 | 0 | 1 |
| MFAP4 | 2 | 0 | 0 | 0 | 0 | 0 | 0 | 0 | 1 |
| ZC3HC1 | 1 | 1 | 0 | 0 | 0 | 0 | 0 | 0 | 1 |
| LACTB2 | 2 | 0 | 0 | 0 | 0 | 0 | 0 | 0 | 1 |
| CDK5 | 2 | 0 | 0 | 0 | 0 | 0 | 0 | 0 | 1 |
| GPATCH4 | 2 | 0 | 0 | 0 | 0 | 0 | 0 | 0 | 1 |
| CA1 | 2 | 0 | 0 | 0 | 0 | 0 | 0 | 0 | 1 |
| RPL3 | 2 | 0 | 0 | 0 | 0 | 0 | 0 | 0 | 1 |
| ATF7IP2 | 1 | 0 | 1 | 0 | 0 | 0 | 0 | 0 | 1 |
| DPY19L4 | 1 | 0 | 1 | 0 | 0 | 0 | 0 | 0 | 1 |
| ARHGDIG | 2 | 0 | 0 | 0 | 0 | 0 | 0 | 0 | 1 |
| ENO3 | 2 | 0 | 0 | 0 | 0 | 0 | 0 | 0 | 1 |
| SERPINB6 | 2 | 0 | 0 | 0 | 0 | 0 | 0 | 0 | 1 |
| IKZF5 | 1 | 1 | 0 | 0 | 0 | 0 | 0 | 0 | 1 |
| TAAR5 | 2 | 0 | 0 | 0 | 0 | 0 | 0 | 0 | 1 |
| TNFRSF13B | 2 | 0 | 0 | 0 | 0 | 0 | 0 | 0 | 1 |
| CLRN2 | 2 | 0 | 0 | 0 | 0 | 0 | 0 | 0 | 1 |
| HMBOX1 | 1 | 0 | 0 | 0 | 1 | 0 | 0 | 0 | 1 |
| IMPACT | 2 | 0 | 0 | 0 | 0 | 0 | 0 | 0 | 1 |
| PINX1 | 2 | 0 | 0 | 0 | 0 | 0 | 0 | 0 | 1 |
| ZNF193 | 2 | 0 | 0 | 0 | 0 | 0 | 0 | 0 | 1 |
| ACBD4 | 2 | 0 | 0 | 0 | 0 | 0 | 0 | 0 | 1 |
| BIN1 | 1 | 0 | 1 | 0 | 0 | 0 | 0 | 0 | 1 |
| CST8 | 2 | 0 | 0 | 0 | 0 | 0 | 0 | 0 | 1 |
| RAD51 | 2 | 0 | 0 | 0 | 0 | 0 | 0 | 0 | 1 |
| TAS2R16 | 2 | 0 | 0 | 0 | 0 | 0 | 0 | 0 | 1 |
| KCNIP2 | 2 | 0 | 0 | 0 | 0 | 0 | 0 | 0 | 1 |

| | | | | | | | | | |
|---|---|---|---|---|---|---|---|---|---|
| OR10A3 | 2 | 0 | 0 | 0 | 0 | 0 | 0 | 0 | 1 |
| RTF1 | 1 | 1 | 0 | 0 | 0 | 0 | 0 | 0 | 1 |
| C15orf32 | 2 | 0 | 0 | 0 | 0 | 0 | 0 | 0 | 1 |
| DNAJC11 | 2 | 0 | 0 | 0 | 0 | 0 | 0 | 0 | 1 |
| PPP4C | 2 | 0 | 0 | 0 | 0 | 0 | 0 | 0 | 1 |
| STK39 | 0 | 2 | 0 | 0 | 0 | 0 | 0 | 0 | 1 |
| ZNF213 | 1 | 1 | 0 | 0 | 0 | 0 | 0 | 0 | 1 |
| DGAT2L6 | 2 | 0 | 0 | 0 | 0 | 0 | 0 | 0 | 1 |
| RGS17 | 2 | 0 | 0 | 0 | 0 | 0 | 0 | 0 | 1 |
| NTF3 | 2 | 0 | 0 | 0 | 0 | 0 | 0 | 0 | 1 |
| TMEM39A | 1 | 0 | 1 | 0 | 0 | 0 | 0 | 0 | 1 |
| PHB2 | 2 | 0 | 0 | 0 | 0 | 0 | 0 | 0 | 1 |
| PRR5L | 2 | 0 | 0 | 0 | 0 | 0 | 0 | 0 | 1 |
| CAPSL | 2 | 0 | 0 | 0 | 0 | 0 | 0 | 0 | 1 |
| VASH1 | 2 | 0 | 0 | 0 | 0 | 0 | 0 | 0 | 1 |
| ZNF434 | 2 | 0 | 0 | 0 | 0 | 0 | 0 | 0 | 1 |
| MTRR | 0 | 2 | 0 | 0 | 0 | 0 | 0 | 0 | 1 |
| GAS7 | 1 | 1 | 0 | 0 | 0 | 0 | 0 | 0 | 1 |
| OR56A5 | 2 | 0 | 0 | 0 | 0 | 0 | 0 | 0 | 1 |
| APPL2 | 1 | 0 | 1 | 0 | 0 | 0 | 0 | 0 | 1 |
| C4orf51 | 2 | 0 | 0 | 0 | 0 | 0 | 0 | 0 | 1 |
| PTCD2 | 2 | 0 | 0 | 0 | 0 | 0 | 0 | 0 | 1 |
| MTFR1 | 2 | 0 | 0 | 0 | 0 | 0 | 0 | 0 | 1 |
| HCN3 | 1 | 1 | 0 | 0 | 0 | 0 | 0 | 0 | 1 |
| ATP1B2 | 2 | 0 | 0 | 0 | 0 | 0 | 0 | 0 | 1 |
| OR10G8 | 2 | 0 | 0 | 0 | 0 | 0 | 0 | 0 | 1 |
| PKM2 | 1 | 1 | 0 | 0 | 0 | 0 | 0 | 0 | 1 |
| KRCC1 | 2 | 0 | 0 | 0 | 0 | 0 | 0 | 0 | 1 |
| RNASET2 | 2 | 0 | 0 | 0 | 0 | 0 | 0 | 0 | 1 |
| OR7C2 | 2 | 0 | 0 | 0 | 0 | 0 | 0 | 0 | 1 |
| WFDC3 | 2 | 0 | 0 | 0 | 0 | 0 | 0 | 0 | 1 |
| SNRPN | 2 | 0 | 0 | 0 | 0 | 0 | 0 | 0 | 1 |
| CD80 | 2 | 0 | 0 | 0 | 0 | 0 | 0 | 0 | 1 |
| PMPCA | 2 | 0 | 0 | 0 | 0 | 0 | 0 | 0 | 1 |
| OR9I1 | 2 | 0 | 0 | 0 | 0 | 0 | 0 | 0 | 1 |
| PRDX6 | 2 | 0 | 0 | 0 | 0 | 0 | 0 | 0 | 1 |
| FYTTD1 | 1 | 1 | 0 | 0 | 0 | 0 | 0 | 0 | 1 |
| EZH1 | 1 | 1 | 0 | 0 | 0 | 0 | 0 | 0 | 1 |
| C1orf43 | 2 | 0 | 0 | 0 | 0 | 0 | 0 | 0 | 1 |
| VTCN1 | 2 | 0 | 0 | 0 | 0 | 0 | 0 | 0 | 1 |
| TBPL2 | 2 | 0 | 0 | 0 | 0 | 0 | 0 | 0 | 1 |
| RTDR1 | 2 | 0 | 0 | 0 | 0 | 0 | 0 | 0 | 1 |
| FGF3 | 2 | 0 | 0 | 0 | 0 | 0 | 0 | 0 | 1 |
| SELPLG | 2 | 0 | 0 | 0 | 0 | 0 | 0 | 0 | 1 |
| FRRS1 | 1 | 1 | 0 | 0 | 0 | 0 | 0 | 0 | 1 |
| PPAP2A | 2 | 0 | 0 | 0 | 0 | 0 | 0 | 0 | 1 |

| | | | | | | | | | |
|---|---|---|---|---|---|---|---|---|---|
| KDM1B | 1 | 1 | 0 | 0 | 0 | 0 | 0 | 0 | 1 |
| GPX5 | 2 | 0 | 0 | 0 | 0 | 0 | 0 | 0 | 1 |
| FST | 2 | 0 | 0 | 0 | 0 | 0 | 0 | 0 | 1 |
| TGDS | 2 | 0 | 0 | 0 | 0 | 0 | 0 | 0 | 1 |
| IL2RA | 2 | 0 | 0 | 0 | 0 | 0 | 0 | 0 | 1 |
| WHSC2 | 2 | 0 | 0 | 0 | 0 | 0 | 0 | 0 | 1 |
| MAGEA11 | 1 | 1 | 0 | 0 | 0 | 0 | 0 | 0 | 1 |
| MS4A3 | 2 | 0 | 0 | 0 | 0 | 0 | 0 | 0 | 1 |
| USP39 | 2 | 0 | 0 | 0 | 0 | 0 | 0 | 0 | 1 |
| SERPINB9 | 2 | 0 | 0 | 0 | 0 | 0 | 0 | 0 | 1 |
| ADRBK1 | 1 | 0 | 1 | 0 | 0 | 0 | 0 | 0 | 1 |
| CD2 | 2 | 0 | 0 | 0 | 0 | 0 | 0 | 0 | 1 |
| PIH1D1 | 2 | 0 | 0 | 0 | 0 | 0 | 0 | 0 | 1 |
| CCDC109B | 2 | 0 | 0 | 0 | 0 | 0 | 0 | 0 | 1 |
| PORCN | 1 | 0 | 1 | 0 | 0 | 0 | 0 | 0 | 1 |
| ZNF74 | 1 | 1 | 0 | 0 | 0 | 0 | 0 | 0 | 1 |
| PRPH2 | 2 | 0 | 0 | 0 | 0 | 0 | 0 | 0 | 1 |
| AADACL3 | 2 | 0 | 0 | 0 | 0 | 0 | 0 | 0 | 1 |
| TNFAIP8L3 | 2 | 0 | 0 | 0 | 0 | 0 | 0 | 0 | 1 |
| C3orf67 | 1 | 0 | 1 | 0 | 0 | 0 | 0 | 0 | 1 |
| GPR19 | 2 | 0 | 0 | 0 | 0 | 0 | 0 | 0 | 1 |
| SRD5A2 | 2 | 0 | 0 | 0 | 0 | 0 | 0 | 0 | 1 |
| ACTL6B | 2 | 0 | 0 | 0 | 0 | 0 | 0 | 0 | 1 |
| WNT2 | 2 | 0 | 0 | 0 | 0 | 0 | 0 | 0 | 1 |
| SAE1 | 2 | 0 | 0 | 0 | 0 | 0 | 0 | 0 | 1 |
| OTC | 1 | 0 | 0 | 0 | 1 | 0 | 0 | 0 | 1 |
| FANCC | 1 | 0 | 1 | 0 | 0 | 0 | 0 | 0 | 1 |
| TDGF1 | 2 | 0 | 0 | 0 | 0 | 0 | 0 | 0 | 1 |
| RNF115 | 2 | 0 | 0 | 0 | 0 | 0 | 0 | 0 | 1 |
| SCN1B | 1 | 1 | 0 | 0 | 0 | 0 | 0 | 0 | 1 |
| TSG101 | 2 | 0 | 0 | 0 | 0 | 0 | 0 | 0 | 1 |
| C1orf177 | 1 | 0 | 1 | 0 | 0 | 0 | 0 | 0 | 1 |
| SLC25A18 | 2 | 0 | 0 | 0 | 0 | 0 | 0 | 0 | 1 |
| NT5DC3 | 2 | 0 | 0 | 0 | 0 | 0 | 0 | 0 | 1 |
| ARGFX | 2 | 0 | 0 | 0 | 0 | 0 | 0 | 0 | 1 |
| ACSM3 | 1 | 0 | 1 | 0 | 0 | 0 | 0 | 0 | 1 |
| ARR3 | 2 | 0 | 0 | 0 | 0 | 0 | 0 | 0 | 1 |
| PAX4 | 2 | 0 | 0 | 0 | 0 | 0 | 0 | 0 | 1 |
| MPPE1 | 2 | 0 | 0 | 0 | 0 | 0 | 0 | 0 | 1 |
| A4GNT | 2 | 0 | 0 | 0 | 0 | 0 | 0 | 0 | 1 |
| HORMAD1 | 1 | 1 | 0 | 0 | 0 | 0 | 0 | 0 | 1 |
| ITPK1 | 2 | 0 | 0 | 0 | 0 | 0 | 0 | 0 | 1 |
| GABRR3 | 1 | 1 | 0 | 0 | 0 | 0 | 0 | 0 | 1 |
| IL12B | 2 | 0 | 0 | 0 | 0 | 0 | 0 | 0 | 1 |
| SLC25A14 | 2 | 0 | 0 | 0 | 0 | 0 | 0 | 0 | 1 |
| DSTYK | 1 | 1 | 0 | 0 | 0 | 0 | 0 | 0 | 1 |

| | | | | | | | | | |
|---|---|---|---|---|---|---|---|---|---|
| CENPP | 2 | 0 | 0 | 0 | 0 | 0 | 0 | 0 | 1 |
| NR1H4 | 1 | 1 | 0 | 0 | 0 | 0 | 0 | 0 | 1 |
| VPS45 | 2 | 0 | 0 | 0 | 0 | 0 | 0 | 0 | 1 |
| OR51Q1 | 2 | 0 | 0 | 0 | 0 | 0 | 0 | 0 | 1 |
| CLRN1 | 2 | 0 | 0 | 0 | 0 | 0 | 0 | 0 | 1 |
| GPR107 | 1 | 1 | 0 | 0 | 0 | 0 | 0 | 0 | 1 |
| PRSS37 | 2 | 0 | 0 | 0 | 0 | 0 | 0 | 0 | 1 |
| C10orf2 | 1 | 1 | 0 | 0 | 0 | 0 | 0 | 0 | 1 |
| RELL1 | 2 | 0 | 0 | 0 | 0 | 0 | 0 | 0 | 1 |
| DEM1 | 2 | 0 | 0 | 0 | 0 | 0 | 0 | 0 | 1 |
| OR13A1 | 2 | 0 | 0 | 0 | 0 | 0 | 0 | 0 | 1 |
| ALDOA | 2 | 0 | 0 | 0 | 0 | 0 | 0 | 0 | 1 |
| FBXO39 | 2 | 0 | 0 | 0 | 0 | 0 | 0 | 0 | 1 |
| SGCE | 2 | 0 | 0 | 0 | 0 | 0 | 0 | 0 | 1 |
| SLC25A13 | 1 | 1 | 0 | 0 | 0 | 0 | 0 | 0 | 1 |
| GPR63 | 2 | 0 | 0 | 0 | 0 | 0 | 0 | 0 | 1 |
| PTOV1 | 2 | 0 | 0 | 0 | 0 | 0 | 0 | 0 | 1 |
| ZNF134 | 1 | 1 | 0 | 0 | 0 | 0 | 0 | 0 | 1 |
| MGEA5 | 1 | 1 | 0 | 0 | 0 | 0 | 0 | 0 | 1 |
| OR52E6 | 2 | 0 | 0 | 0 | 0 | 0 | 0 | 0 | 1 |
| STIP1 | 2 | 0 | 0 | 0 | 0 | 0 | 0 | 0 | 1 |
| TAF7 | 2 | 0 | 0 | 0 | 0 | 0 | 0 | 0 | 1 |
| LRRC41 | 1 | 0 | 1 | 0 | 0 | 0 | 0 | 0 | 1 |
| SFN | 2 | 0 | 0 | 0 | 0 | 0 | 0 | 0 | 1 |
| AQP4 | 2 | 0 | 0 | 0 | 0 | 0 | 0 | 0 | 1 |
| STYXL1 | 2 | 0 | 0 | 0 | 0 | 0 | 0 | 0 | 1 |
| REG3G | 2 | 0 | 0 | 0 | 0 | 0 | 0 | 0 | 1 |
| ZNF70 | 2 | 0 | 0 | 0 | 0 | 0 | 0 | 0 | 1 |
| CDC45 | 1 | 1 | 0 | 0 | 0 | 0 | 0 | 0 | 1 |
| PRKAB2 | 2 | 0 | 0 | 0 | 0 | 0 | 0 | 0 | 1 |
| CHRNB3 | 2 | 0 | 0 | 0 | 0 | 0 | 0 | 0 | 1 |
| C6orf89 | 2 | 0 | 0 | 0 | 0 | 0 | 0 | 0 | 1 |
| OR52M1 | 2 | 0 | 0 | 0 | 0 | 0 | 0 | 0 | 1 |
| EAF2 | 2 | 0 | 0 | 0 | 0 | 0 | 0 | 0 | 1 |
| CXorf1 | 2 | 0 | 0 | 0 | 0 | 0 | 0 | 0 | 1 |
| PLVAP | 2 | 0 | 0 | 0 | 0 | 0 | 0 | 0 | 1 |
| C12orf70 | 2 | 0 | 0 | 0 | 0 | 0 | 0 | 0 | 1 |
| DIO2 | 2 | 0 | 0 | 0 | 0 | 0 | 0 | 0 | 1 |
| RPUSD2 | 1 | 1 | 0 | 0 | 0 | 0 | 0 | 0 | 1 |
| LRRC58 | 2 | 0 | 0 | 0 | 0 | 0 | 0 | 0 | 1 |
| SLC29A1 | 2 | 0 | 0 | 0 | 0 | 0 | 0 | 0 | 1 |
| F2RL2 | 2 | 0 | 0 | 0 | 0 | 0 | 0 | 0 | 1 |
| ZNF263 | 2 | 0 | 0 | 0 | 0 | 0 | 0 | 0 | 1 |
| CASP10 | 1 | 1 | 0 | 0 | 0 | 0 | 0 | 0 | 1 |
| DHX15 | 0 | 1 | 0 | 0 | 1 | 0 | 0 | 0 | 1 |
| ACTA1 | 2 | 0 | 0 | 0 | 0 | 0 | 0 | 0 | 1 |

| | | | | | | | | | |
|---|---|---|---|---|---|---|---|---|---|
| ZCCHC16 | 2 | 0 | 0 | 0 | 0 | 0 | 0 | 0 | 1 |
| SDHC | 2 | 0 | 0 | 0 | 0 | 0 | 0 | 0 | 1 |
| PRR21 | 2 | 0 | 0 | 0 | 0 | 0 | 0 | 0 | 1 |
| SYPL2 | 2 | 0 | 0 | 0 | 0 | 0 | 0 | 0 | 1 |
| KLK7 | 2 | 0 | 0 | 0 | 0 | 0 | 0 | 0 | 1 |
| C17orf56 | 2 | 0 | 0 | 0 | 0 | 0 | 0 | 0 | 1 |
| DBNL | 2 | 0 | 0 | 0 | 0 | 0 | 0 | 0 | 1 |
| CYP7B1 | 2 | 0 | 0 | 0 | 0 | 0 | 0 | 0 | 1 |
| OR2W5 | 1 | 1 | 0 | 0 | 0 | 0 | 0 | 0 | 1 |
| TIMM44 | 2 | 0 | 0 | 0 | 0 | 0 | 0 | 0 | 1 |
| SEC62 | 2 | 0 | 0 | 0 | 0 | 0 | 0 | 0 | 1 |
| RNF113A | 2 | 0 | 0 | 0 | 0 | 0 | 0 | 0 | 1 |
| NGF | 2 | 0 | 0 | 0 | 0 | 0 | 0 | 0 | 1 |
| ADCK1 | 2 | 0 | 0 | 0 | 0 | 0 | 0 | 0 | 1 |
| KATNA1 | 2 | 0 | 0 | 0 | 0 | 0 | 0 | 0 | 1 |
| APOF | 2 | 0 | 0 | 0 | 0 | 0 | 0 | 0 | 1 |
| ARHGAP4 | 1 | 1 | 0 | 0 | 0 | 0 | 0 | 0 | 1 |
| RAB3IL1 | 2 | 0 | 0 | 0 | 0 | 0 | 0 | 0 | 1 |
| ME1 | 1 | 0 | 1 | 0 | 0 | 0 | 0 | 0 | 1 |
| CCDC34 | 2 | 0 | 0 | 0 | 0 | 0 | 0 | 0 | 1 |
| REG3A | 2 | 0 | 0 | 0 | 0 | 0 | 0 | 0 | 1 |
| FBXW2 | 2 | 0 | 0 | 0 | 0 | 0 | 0 | 0 | 1 |
| PRSS45 | 2 | 0 | 0 | 0 | 0 | 0 | 0 | 0 | 1 |
| ANKS4B | 2 | 0 | 0 | 0 | 0 | 0 | 0 | 0 | 1 |
| IGFBP3 | 2 | 0 | 0 | 0 | 0 | 0 | 0 | 0 | 1 |
| PCGF2 | 2 | 0 | 0 | 0 | 0 | 0 | 0 | 0 | 1 |
| ZNF80 | 2 | 0 | 0 | 0 | 0 | 0 | 0 | 0 | 1 |
| DIO1 | 2 | 0 | 0 | 0 | 0 | 0 | 0 | 0 | 1 |
| LHX3 | 2 | 0 | 0 | 0 | 0 | 0 | 0 | 0 | 1 |
| MTO1 | 1 | 1 | 0 | 0 | 0 | 0 | 0 | 0 | 1 |
| SIX6 | 2 | 0 | 0 | 0 | 0 | 0 | 0 | 0 | 1 |
| SERINC2 | 2 | 0 | 0 | 0 | 0 | 0 | 0 | 0 | 1 |
| FASTKD5 | 1 | 1 | 0 | 0 | 0 | 0 | 0 | 0 | 1 |
| PPM1H | 2 | 0 | 0 | 0 | 0 | 0 | 0 | 0 | 1 |
| MKNK2 | 1 | 0 | 0 | 0 | 1 | 0 | 0 | 0 | 1 |
| LAT | 2 | 0 | 0 | 0 | 0 | 0 | 0 | 0 | 1 |
| PRR4 | 2 | 0 | 0 | 0 | 0 | 0 | 0 | 0 | 1 |
| WDR46 | 2 | 0 | 0 | 0 | 0 | 0 | 0 | 0 | 1 |
| PHGDH | 1 | 0 | 1 | 0 | 0 | 0 | 0 | 0 | 1 |
| PLK1S1 | 1 | 0 | 1 | 0 | 0 | 0 | 0 | 0 | 1 |
| AJAP1 | 2 | 0 | 0 | 0 | 0 | 0 | 0 | 0 | 1 |
| PROP1 | 2 | 0 | 0 | 0 | 0 | 0 | 0 | 0 | 1 |
| CCND2 | 2 | 0 | 0 | 0 | 0 | 0 | 0 | 0 | 1 |
| ADRA2C | 2 | 0 | 0 | 0 | 0 | 0 | 0 | 0 | 1 |
| CPA5 | 2 | 0 | 0 | 0 | 0 | 0 | 0 | 0 | 1 |
| ZDHHC19 | 2 | 0 | 0 | 0 | 0 | 0 | 0 | 0 | 1 |

| | | | | | | | | | |
|---|---|---|---|---|---|---|---|---|---|
| OR51S1 | 2 | 0 | 0 | 0 | 0 | 0 | 0 | 0 | 1 |
| NXPH1 | 2 | 0 | 0 | 0 | 0 | 0 | 0 | 0 | 1 |
| MAB21L1 | 2 | 0 | 0 | 0 | 0 | 0 | 0 | 0 | 1 |
| ZNF572 | 2 | 0 | 0 | 0 | 0 | 0 | 0 | 0 | 1 |
| OR4F15 | 2 | 0 | 0 | 0 | 0 | 0 | 0 | 0 | 1 |
| GPC4 | 2 | 0 | 0 | 0 | 0 | 0 | 0 | 0 | 1 |
| CCDC83 | 2 | 0 | 0 | 0 | 0 | 0 | 0 | 0 | 1 |
| GLB1 | 1 | 1 | 0 | 0 | 0 | 0 | 0 | 0 | 1 |
| SERPINB8 | 2 | 0 | 0 | 0 | 0 | 0 | 0 | 0 | 1 |
| RBM23 | 2 | 0 | 0 | 0 | 0 | 0 | 0 | 0 | 1 |
| LECT1 | 2 | 0 | 0 | 0 | 0 | 0 | 0 | 0 | 1 |
| CTSG | 2 | 0 | 0 | 0 | 0 | 0 | 0 | 0 | 1 |
| SERINC3 | 2 | 0 | 0 | 0 | 0 | 0 | 0 | 0 | 1 |
| MEIS3 | 2 | 0 | 0 | 0 | 0 | 0 | 0 | 0 | 1 |
| C3AR1 | 2 | 0 | 0 | 0 | 0 | 0 | 0 | 0 | 1 |
| C1orf174 | 2 | 0 | 0 | 0 | 0 | 0 | 0 | 0 | 1 |
| AIPL1 | 2 | 0 | 0 | 0 | 0 | 0 | 0 | 0 | 1 |
| KRT12 | 2 | 0 | 0 | 0 | 0 | 0 | 0 | 0 | 1 |
| C2orf77 | 2 | 0 | 0 | 0 | 0 | 0 | 0 | 0 | 1 |
| HLF | 2 | 0 | 0 | 0 | 0 | 0 | 0 | 0 | 1 |
| RARG | 2 | 0 | 0 | 0 | 0 | 0 | 0 | 0 | 1 |
| KCNK15 | 2 | 0 | 0 | 0 | 0 | 0 | 0 | 0 | 1 |
| EPHA10 | 1 | 1 | 0 | 0 | 0 | 0 | 0 | 0 | 1 |
| CCDC79 | 1 | 0 | 1 | 0 | 0 | 0 | 0 | 0 | 1 |
| NAT2 | 2 | 0 | 0 | 0 | 0 | 0 | 0 | 0 | 1 |
| TRIM62 | 2 | 0 | 0 | 0 | 0 | 0 | 0 | 0 | 1 |
| CUL1 | 1 | 1 | 0 | 0 | 0 | 0 | 0 | 0 | 1 |
| BMP6 | 2 | 0 | 0 | 0 | 0 | 0 | 0 | 0 | 1 |
| CUL3 | 1 | 1 | 0 | 0 | 0 | 0 | 0 | 0 | 1 |
| UMPS | 2 | 0 | 0 | 0 | 0 | 0 | 0 | 0 | 1 |
| PSMC6 | 2 | 0 | 0 | 0 | 0 | 0 | 0 | 0 | 1 |
| EXD1 | 2 | 0 | 0 | 0 | 0 | 0 | 0 | 0 | 1 |
| RALGPS1 | 2 | 0 | 0 | 0 | 0 | 0 | 0 | 0 | 1 |
| NR1H2 | 1 | 1 | 0 | 0 | 0 | 0 | 0 | 0 | 1 |
| C10orf118 | 1 | 1 | 0 | 0 | 0 | 0 | 0 | 0 | 1 |
| ACTL7A | 2 | 0 | 0 | 0 | 0 | 0 | 0 | 0 | 1 |
| PDK2 | 2 | 0 | 0 | 0 | 0 | 0 | 0 | 0 | 1 |
| SERPINC1 | 2 | 0 | 0 | 0 | 0 | 0 | 0 | 0 | 1 |
| METTL11B | 2 | 0 | 0 | 0 | 0 | 0 | 0 | 0 | 1 |
| ST3GAL5 | 2 | 0 | 0 | 0 | 0 | 0 | 0 | 0 | 1 |
| POLD3 | 2 | 0 | 0 | 0 | 0 | 0 | 0 | 0 | 1 |
| SLC28A1 | 1 | 0 | 1 | 0 | 0 | 0 | 0 | 0 | 1 |
| ZNF264 | 1 | 1 | 0 | 0 | 0 | 0 | 0 | 0 | 1 |
| TPCN2 | 1 | 1 | 0 | 0 | 0 | 0 | 0 | 0 | 1 |
| SLC47A2 | 1 | 1 | 0 | 0 | 0 | 0 | 0 | 0 | 1 |
| GSDMA | 2 | 0 | 0 | 0 | 0 | 0 | 0 | 0 | 1 |

| | | | | | | | | | |
|---|---|---|---|---|---|---|---|---|---|
| OR2A5 | 2 | 0 | 0 | 0 | 0 | 0 | 0 | 0 | 1 |
| RBM39 | 2 | 0 | 0 | 0 | 0 | 0 | 0 | 0 | 1 |
| SLAMF9 | 2 | 0 | 0 | 0 | 0 | 0 | 0 | 0 | 1 |
| CYP26C1 | 2 | 0 | 0 | 0 | 0 | 0 | 0 | 0 | 1 |
| TNMD | 1 | 0 | 1 | 0 | 0 | 0 | 0 | 0 | 1 |
| TAS2R20 | 2 | 0 | 0 | 0 | 0 | 0 | 0 | 0 | 1 |
| C7orf72 | 1 | 0 | 1 | 0 | 0 | 0 | 0 | 0 | 1 |
| OR7G3 | 2 | 0 | 0 | 0 | 0 | 0 | 0 | 0 | 1 |
| FIGF | 2 | 0 | 0 | 0 | 0 | 0 | 0 | 0 | 1 |
| DDX55 | 2 | 0 | 0 | 0 | 0 | 0 | 0 | 0 | 1 |
| HTR3C | 2 | 0 | 0 | 0 | 0 | 0 | 0 | 0 | 1 |
| IKBKE | 1 | 1 | 0 | 0 | 0 | 0 | 0 | 0 | 1 |
| RUFY4 | 2 | 0 | 0 | 0 | 0 | 0 | 0 | 0 | 1 |
| CA4 | 2 | 0 | 0 | 0 | 0 | 0 | 0 | 0 | 1 |
| NR2C1 | 1 | 0 | 1 | 0 | 0 | 0 | 0 | 0 | 1 |
| CRYAB | 2 | 0 | 0 | 0 | 0 | 0 | 0 | 0 | 1 |
| TDG | 2 | 0 | 0 | 0 | 0 | 0 | 0 | 0 | 1 |
| OR52E8 | 2 | 0 | 0 | 0 | 0 | 0 | 0 | 0 | 1 |
| C9orf171 | 2 | 0 | 0 | 0 | 0 | 0 | 0 | 0 | 1 |
| GOT1L1 | 2 | 0 | 0 | 0 | 0 | 0 | 0 | 0 | 1 |
| MFSD7 | 1 | 1 | 0 | 0 | 0 | 0 | 0 | 0 | 1 |
| NHLRC3 | 2 | 0 | 0 | 0 | 0 | 0 | 0 | 0 | 1 |
| C9orf64 | 2 | 0 | 0 | 0 | 0 | 0 | 0 | 0 | 1 |
| MKRN2 | 2 | 0 | 0 | 0 | 0 | 0 | 0 | 0 | 1 |
| TFDP3 | 2 | 0 | 0 | 0 | 0 | 0 | 0 | 0 | 1 |
| FBXO7 | 2 | 0 | 0 | 0 | 0 | 0 | 0 | 0 | 1 |
| ASAH1 | 2 | 0 | 0 | 0 | 0 | 0 | 0 | 0 | 1 |
| PLA2G6 | 1 | 1 | 0 | 0 | 0 | 0 | 0 | 0 | 1 |
| AGPAT4 | 2 | 0 | 0 | 0 | 0 | 0 | 0 | 0 | 1 |
| LIN9 | 2 | 0 | 0 | 0 | 0 | 0 | 0 | 0 | 1 |
| BSPRY | 2 | 0 | 0 | 0 | 0 | 0 | 0 | 0 | 1 |
| EPHA2 | 1 | 1 | 0 | 0 | 0 | 0 | 0 | 0 | 1 |
| PSMG2 | 2 | 0 | 0 | 0 | 0 | 0 | 0 | 0 | 1 |
| NKAIN2 | 2 | 0 | 0 | 0 | 0 | 0 | 0 | 0 | 1 |
| TMEM171 | 2 | 0 | 0 | 0 | 0 | 0 | 0 | 0 | 1 |
| CCDC42 | 2 | 0 | 0 | 0 | 0 | 0 | 0 | 0 | 1 |
| DPRX | 2 | 0 | 0 | 0 | 0 | 0 | 0 | 0 | 1 |
| C1QTNF2 | 2 | 0 | 0 | 0 | 0 | 0 | 0 | 0 | 1 |
| P2RY13 | 2 | 0 | 0 | 0 | 0 | 0 | 0 | 0 | 1 |
| E2F5 | 2 | 0 | 0 | 0 | 0 | 0 | 0 | 0 | 1 |
| FBXO5 | 2 | 0 | 0 | 0 | 0 | 0 | 0 | 0 | 1 |
| SERPINB5 | 2 | 0 | 0 | 0 | 0 | 0 | 0 | 0 | 1 |
| CELF2 | 1 | 1 | 0 | 0 | 0 | 0 | 0 | 0 | 1 |
| TRIB1 | 2 | 0 | 0 | 0 | 0 | 0 | 0 | 0 | 1 |
| KLHDC4 | 2 | 0 | 0 | 0 | 0 | 0 | 0 | 0 | 1 |
| FIBCD1 | 2 | 0 | 0 | 0 | 0 | 0 | 0 | 0 | 1 |

| | | | | | | | | | |
|---|---|---|---|---|---|---|---|---|---|
| CCT3 | 2 | 0 | 0 | 0 | 0 | 0 | 0 | 0 | 1 |
| KLHL18 | 2 | 0 | 0 | 0 | 0 | 0 | 0 | 0 | 1 |
| STX19 | 2 | 0 | 0 | 0 | 0 | 0 | 0 | 0 | 1 |
| CA3 | 2 | 0 | 0 | 0 | 0 | 0 | 0 | 0 | 1 |
| ZNF396 | 2 | 0 | 0 | 0 | 0 | 0 | 0 | 0 | 1 |
| SAP30BP | 2 | 0 | 0 | 0 | 0 | 0 | 0 | 0 | 1 |
| B3GALNT1 | 2 | 0 | 0 | 0 | 0 | 0 | 0 | 0 | 1 |
| EEFSEC | 2 | 0 | 0 | 0 | 0 | 0 | 0 | 0 | 1 |
| DCAF4 | 2 | 0 | 0 | 0 | 0 | 0 | 0 | 0 | 1 |
| CA8 | 2 | 0 | 0 | 0 | 0 | 0 | 0 | 0 | 1 |
| MS4A13 | 1 | 1 | 0 | 0 | 0 | 0 | 0 | 0 | 1 |
| AP3M1 | 2 | 0 | 0 | 0 | 0 | 0 | 0 | 0 | 1 |
| TM6SF1 | 2 | 0 | 0 | 0 | 0 | 0 | 0 | 0 | 1 |
| RNF121 | 2 | 0 | 0 | 0 | 0 | 0 | 0 | 0 | 1 |
| RIMKLA | 2 | 0 | 0 | 0 | 0 | 0 | 0 | 0 | 1 |
| SNX4 | 2 | 0 | 0 | 0 | 0 | 0 | 0 | 0 | 1 |
| PRSS41 | 2 | 0 | 0 | 0 | 0 | 0 | 0 | 0 | 1 |
| B4GALT1 | 2 | 0 | 0 | 0 | 0 | 0 | 0 | 0 | 1 |
| PAPL | 2 | 0 | 0 | 0 | 0 | 0 | 0 | 0 | 1 |
| CREB3L4 | 2 | 0 | 0 | 0 | 0 | 0 | 0 | 0 | 1 |
| SLC7A11 | 1 | 0 | 0 | 0 | 1 | 0 | 0 | 0 | 1 |
| OR1N1 | 2 | 0 | 0 | 0 | 0 | 0 | 0 | 0 | 1 |
| NCR1 | 2 | 0 | 0 | 0 | 0 | 0 | 0 | 0 | 1 |
| CPNE5 | 2 | 0 | 0 | 0 | 0 | 0 | 0 | 0 | 1 |
| PIK3R3 | 2 | 0 | 0 | 0 | 0 | 0 | 0 | 0 | 1 |
| PCBP3 | 2 | 0 | 0 | 0 | 0 | 0 | 0 | 0 | 1 |
| PKNOX1 | 2 | 0 | 0 | 0 | 0 | 0 | 0 | 0 | 1 |
| CDADC1 | 2 | 0 | 0 | 0 | 0 | 0 | 0 | 0 | 1 |
| C14orf166B | 2 | 0 | 0 | 0 | 0 | 0 | 0 | 0 | 1 |
| MLC1 | 2 | 0 | 0 | 0 | 0 | 0 | 0 | 0 | 1 |
| OR4K14 | 2 | 0 | 0 | 0 | 0 | 0 | 0 | 0 | 1 |
| TAF4B | 2 | 0 | 0 | 0 | 0 | 0 | 0 | 0 | 1 |
| ASPSCR1 | 2 | 0 | 0 | 0 | 0 | 0 | 0 | 0 | 1 |
| AQP9 | 2 | 0 | 0 | 0 | 0 | 0 | 0 | 0 | 1 |
| C9orf3 | 1 | 1 | 0 | 0 | 0 | 0 | 0 | 0 | 1 |
| PRKACA | 2 | 0 | 0 | 0 | 0 | 0 | 0 | 0 | 1 |
| NCAPH | 2 | 0 | 0 | 0 | 0 | 0 | 0 | 0 | 1 |
| GNA12 | 2 | 0 | 0 | 0 | 0 | 0 | 0 | 0 | 1 |
| RABEP2 | 2 | 0 | 0 | 0 | 0 | 0 | 0 | 0 | 1 |
| CLEC10A | 2 | 0 | 0 | 0 | 0 | 0 | 0 | 0 | 1 |
| CYP17A1 | 2 | 0 | 0 | 0 | 0 | 0 | 0 | 0 | 1 |
| SLC13A4 | 1 | 1 | 0 | 0 | 0 | 0 | 0 | 0 | 1 |
| CADM2 | 1 | 0 | 1 | 0 | 0 | 0 | 0 | 0 | 1 |
| CHEK2 | 1 | 1 | 0 | 0 | 0 | 0 | 0 | 0 | 1 |
| FGF14 | 2 | 0 | 0 | 0 | 0 | 0 | 0 | 0 | 1 |
| OR5P3 | 2 | 0 | 0 | 0 | 0 | 0 | 0 | 0 | 1 |

| | | | | | | | | | |
|---|---|---|---|---|---|---|---|---|---|
| TMEM183A | 2 | 0 | 0 | 0 | 0 | 0 | 0 | 0 | 1 |
| ARMCX1 | 2 | 0 | 0 | 0 | 0 | 0 | 0 | 0 | 1 |
| SLC1A5 | 2 | 0 | 0 | 0 | 0 | 0 | 0 | 0 | 1 |
| C22orf31 | 2 | 0 | 0 | 0 | 0 | 0 | 0 | 0 | 1 |
| GPM6A | 2 | 0 | 0 | 0 | 0 | 0 | 0 | 0 | 1 |
| XPC | 2 | 0 | 0 | 0 | 0 | 0 | 0 | 0 | 1 |
| C4orf47 | 2 | 0 | 0 | 0 | 0 | 0 | 0 | 0 | 1 |
| CALR | 2 | 0 | 0 | 0 | 0 | 0 | 0 | 0 | 1 |
| NIT2 | 2 | 0 | 0 | 0 | 0 | 0 | 0 | 0 | 1 |
| SH3GL2 | 2 | 0 | 0 | 0 | 0 | 0 | 0 | 0 | 1 |
| PYGO2 | 2 | 0 | 0 | 0 | 0 | 0 | 0 | 0 | 1 |
| CHUK | 1 | 1 | 0 | 0 | 0 | 0 | 0 | 0 | 1 |
| AHSG | 2 | 0 | 0 | 0 | 0 | 0 | 0 | 0 | 1 |
| PRR19 | 2 | 0 | 0 | 0 | 0 | 0 | 0 | 0 | 1 |
| ACTRT2 | 1 | 1 | 0 | 0 | 0 | 0 | 0 | 0 | 1 |
| MORC3 | 1 | 1 | 0 | 0 | 0 | 0 | 0 | 0 | 1 |
| TGOLN2 | 1 | 0 | 0 | 0 | 1 | 0 | 0 | 0 | 1 |
| CTCF | 2 | 0 | 0 | 0 | 0 | 0 | 0 | 0 | 1 |
| GPR174 | 2 | 0 | 0 | 0 | 0 | 0 | 0 | 0 | 1 |
| PVRL4 | 2 | 0 | 0 | 0 | 0 | 0 | 0 | 0 | 1 |
| PDIA4 | 2 | 0 | 0 | 0 | 0 | 0 | 0 | 0 | 1 |
| C12orf53 | 2 | 0 | 0 | 0 | 0 | 0 | 0 | 0 | 1 |
| MYOG | 2 | 0 | 0 | 0 | 0 | 0 | 0 | 0 | 1 |
| ATP6V1A | 2 | 0 | 0 | 0 | 0 | 0 | 0 | 0 | 1 |
| SLC7A1 | 2 | 0 | 0 | 0 | 0 | 0 | 0 | 0 | 1 |
| PROC | 2 | 0 | 0 | 0 | 0 | 0 | 0 | 0 | 1 |
| IFNAR2 | 2 | 0 | 0 | 0 | 0 | 0 | 0 | 0 | 1 |
| AKR7A2 | 2 | 0 | 0 | 0 | 0 | 0 | 0 | 0 | 1 |
| NCBP1 | 1 | 1 | 0 | 0 | 0 | 0 | 0 | 0 | 1 |
| CDH3 | 1 | 0 | 1 | 0 | 0 | 0 | 0 | 0 | 1 |
| TEX13B | 2 | 0 | 0 | 0 | 0 | 0 | 0 | 0 | 1 |
| TOP1MT | 2 | 0 | 0 | 0 | 0 | 0 | 0 | 0 | 1 |
| PTDSS1 | 2 | 0 | 0 | 0 | 0 | 0 | 0 | 0 | 1 |
| PATZ1 | 1 | 1 | 0 | 0 | 0 | 0 | 0 | 0 | 1 |
| FAM55C | 2 | 0 | 0 | 0 | 0 | 0 | 0 | 0 | 1 |
| MAP2K1 | 2 | 0 | 0 | 0 | 0 | 0 | 0 | 0 | 1 |
| KIF3A | 1 | 0 | 1 | 0 | 0 | 0 | 0 | 0 | 1 |
| APPBP2 | 2 | 0 | 0 | 0 | 0 | 0 | 0 | 0 | 1 |
| AGPAT9 | 2 | 0 | 0 | 0 | 0 | 0 | 0 | 0 | 1 |
| CRNN | 1 | 1 | 0 | 0 | 0 | 0 | 0 | 0 | 1 |
| ASPHD2 | 2 | 0 | 0 | 0 | 0 | 0 | 0 | 0 | 1 |
| OR2A2 | 2 | 0 | 0 | 0 | 0 | 0 | 0 | 0 | 1 |
| ZNF416 | 1 | 0 | 0 | 0 | 0 | 1 | 0 | 0 | 1 |
| RIMS4 | 2 | 0 | 0 | 0 | 0 | 0 | 0 | 0 | 1 |
| CPA3 | 2 | 0 | 0 | 0 | 0 | 0 | 0 | 0 | 1 |
| NECAB2 | 2 | 0 | 0 | 0 | 0 | 0 | 0 | 0 | 1 |

| | | | | | | | | | |
|---|---|---|---|---|---|---|---|---|---|
| EIF2A | 1 | 1 | 0 | 0 | 0 | 0 | 0 | 0 | 1 |
| P2RX6 | 2 | 0 | 0 | 0 | 0 | 0 | 0 | 0 | 1 |
| PIPOX | 2 | 0 | 0 | 0 | 0 | 0 | 0 | 0 | 1 |
| FAM19A5 | 2 | 0 | 0 | 0 | 0 | 0 | 0 | 0 | 1 |
| WBSCR28 | 2 | 0 | 0 | 0 | 0 | 0 | 0 | 0 | 1 |
| EXOG | 2 | 0 | 0 | 0 | 0 | 0 | 0 | 0 | 1 |
| ZNF789 | 2 | 0 | 0 | 0 | 0 | 0 | 0 | 0 | 1 |
| ZNF239 | 1 | 1 | 0 | 0 | 0 | 0 | 0 | 0 | 1 |
| MTF1 | 1 | 0 | 1 | 0 | 0 | 0 | 0 | 0 | 1 |
| EIF4B | 2 | 0 | 0 | 0 | 0 | 0 | 0 | 0 | 1 |
| NPHS2 | 2 | 0 | 0 | 0 | 0 | 0 | 0 | 0 | 1 |
| PPWD1 | 2 | 0 | 0 | 0 | 0 | 0 | 0 | 0 | 1 |
| PRSS42 | 2 | 0 | 0 | 0 | 0 | 0 | 0 | 0 | 1 |
| RCN1 | 2 | 0 | 0 | 0 | 0 | 0 | 0 | 0 | 1 |
| OR5B17 | 2 | 0 | 0 | 0 | 0 | 0 | 0 | 0 | 1 |
| KRT85 | 2 | 0 | 0 | 0 | 0 | 0 | 0 | 0 | 1 |
| ONECUT2 | 2 | 0 | 0 | 0 | 0 | 0 | 0 | 0 | 1 |
| LRRC15 | 2 | 0 | 0 | 0 | 0 | 0 | 0 | 0 | 1 |
| GDPD5 | 2 | 0 | 0 | 0 | 0 | 0 | 0 | 0 | 1 |
| PPARG | 2 | 0 | 0 | 0 | 0 | 0 | 0 | 0 | 1 |
| OTUD3 | 2 | 0 | 0 | 0 | 0 | 0 | 0 | 0 | 1 |
| OR4F6 | 2 | 0 | 0 | 0 | 0 | 0 | 0 | 0 | 1 |
| NEIL2 | 2 | 0 | 0 | 0 | 0 | 0 | 0 | 0 | 1 |
| DPP9 | 1 | 1 | 0 | 0 | 0 | 0 | 0 | 0 | 1 |
| ZNF143 | 2 | 0 | 0 | 0 | 0 | 0 | 0 | 0 | 1 |
| PCGF6 | 2 | 0 | 0 | 0 | 0 | 0 | 0 | 0 | 1 |
| LIX1L | 2 | 0 | 0 | 0 | 0 | 0 | 0 | 0 | 1 |
| KIR2DL4 | 2 | 0 | 0 | 0 | 0 | 0 | 0 | 0 | 1 |
| ASPA | 2 | 0 | 0 | 0 | 0 | 0 | 0 | 0 | 1 |
| LRRTM2 | 2 | 0 | 0 | 0 | 0 | 0 | 0 | 0 | 1 |
| PRKAG2 | 2 | 0 | 0 | 0 | 0 | 0 | 0 | 0 | 1 |
| COPS4 | 2 | 0 | 0 | 0 | 0 | 0 | 0 | 0 | 1 |
| CCIN | 2 | 0 | 0 | 0 | 0 | 0 | 0 | 0 | 1 |
| TMEM163 | 2 | 0 | 0 | 0 | 0 | 0 | 0 | 0 | 1 |
| MCAT | 2 | 0 | 0 | 0 | 0 | 0 | 0 | 0 | 1 |
| DCP1A | 2 | 0 | 0 | 0 | 0 | 0 | 0 | 0 | 1 |
| PACSIN1 | 2 | 0 | 0 | 0 | 0 | 0 | 0 | 0 | 1 |
| SIRT2 | 2 | 0 | 0 | 0 | 0 | 0 | 0 | 0 | 1 |
| SLC46A3 | 2 | 0 | 0 | 0 | 0 | 0 | 0 | 0 | 1 |
| KMO | 2 | 0 | 0 | 0 | 0 | 0 | 0 | 0 | 1 |
| MTMR7 | 2 | 0 | 0 | 0 | 0 | 0 | 0 | 0 | 1 |
| IVNS1ABP | 1 | 1 | 0 | 0 | 0 | 0 | 0 | 0 | 1 |
| RAB19 | 1 | 1 | 0 | 0 | 0 | 0 | 0 | 0 | 1 |
| BBS1 | 2 | 0 | 0 | 0 | 0 | 0 | 0 | 0 | 1 |
| HDX | 1 | 1 | 0 | 0 | 0 | 0 | 0 | 0 | 1 |
| PSAP | 2 | 0 | 0 | 0 | 0 | 0 | 0 | 0 | 1 |

| | | | | | | | | | |
|---|---|---|---|---|---|---|---|---|---|
| RUNX2 | 2 | 0 | 0 | 0 | 0 | 0 | 0 | 0 | 1 |
| RCL1 | 2 | 0 | 0 | 0 | 0 | 0 | 0 | 0 | 1 |
| GPR176 | 2 | 0 | 0 | 0 | 0 | 0 | 0 | 0 | 1 |
| CCM2 | 2 | 0 | 0 | 0 | 0 | 0 | 0 | 0 | 1 |
| SPRY1 | 2 | 0 | 0 | 0 | 0 | 0 | 0 | 0 | 1 |
| OR10P1 | 2 | 0 | 0 | 0 | 0 | 0 | 0 | 0 | 1 |
| FAM123C | 2 | 0 | 0 | 0 | 0 | 0 | 0 | 0 | 1 |
| FOXRED1 | 2 | 0 | 0 | 0 | 0 | 0 | 0 | 0 | 1 |
| GZMA | 2 | 0 | 0 | 0 | 0 | 0 | 0 | 0 | 1 |
| ELK4 | 2 | 0 | 0 | 0 | 0 | 0 | 0 | 0 | 1 |
| LIPG | 2 | 0 | 0 | 0 | 0 | 0 | 0 | 0 | 1 |
| OR4S2 | 2 | 0 | 0 | 0 | 0 | 0 | 0 | 0 | 1 |
| TBCE | 2 | 0 | 0 | 0 | 0 | 0 | 0 | 0 | 1 |
| OR5K2 | 2 | 0 | 0 | 0 | 0 | 0 | 0 | 0 | 1 |
| KCNK5 | 2 | 0 | 0 | 0 | 0 | 0 | 0 | 0 | 1 |
| FOXP3 | 2 | 0 | 0 | 0 | 0 | 0 | 0 | 0 | 1 |
| IQCK | 2 | 0 | 0 | 0 | 0 | 0 | 0 | 0 | 1 |
| MTM1 | 2 | 0 | 0 | 0 | 0 | 0 | 0 | 0 | 1 |
| CSN3 | 2 | 0 | 0 | 0 | 0 | 0 | 0 | 0 | 1 |
| HELT | 2 | 0 | 0 | 0 | 0 | 0 | 0 | 0 | 1 |
| CD47 | 2 | 0 | 0 | 0 | 0 | 0 | 0 | 0 | 1 |
| ARHGEF1 | 1 | 0 | 1 | 0 | 0 | 0 | 0 | 0 | 1 |
| FFAR1 | 2 | 0 | 0 | 0 | 0 | 0 | 0 | 0 | 1 |
| GATA1 | 2 | 0 | 0 | 0 | 0 | 0 | 0 | 0 | 1 |
| GNB5 | 2 | 0 | 0 | 0 | 0 | 0 | 0 | 0 | 1 |
| ZNF79 | 2 | 0 | 0 | 0 | 0 | 0 | 0 | 0 | 1 |
| PDLIM3 | 2 | 0 | 0 | 0 | 0 | 0 | 0 | 0 | 1 |
| LHX6 | 2 | 0 | 0 | 0 | 0 | 0 | 0 | 0 | 1 |
| SYT2 | 2 | 0 | 0 | 0 | 0 | 0 | 0 | 0 | 1 |
| BTN3A3 | 2 | 0 | 0 | 0 | 0 | 0 | 0 | 0 | 1 |
| SSTR3 | 2 | 0 | 0 | 0 | 0 | 0 | 0 | 0 | 1 |
| C11orf80 | 1 | 0 | 1 | 0 | 0 | 0 | 0 | 0 | 1 |
| PRR11 | 2 | 0 | 0 | 0 | 0 | 0 | 0 | 0 | 1 |
| SLC22A7 | 2 | 0 | 0 | 0 | 0 | 0 | 0 | 0 | 1 |
| KRT4 | 2 | 0 | 0 | 0 | 0 | 0 | 0 | 0 | 1 |
| PRAME | 2 | 0 | 0 | 0 | 0 | 0 | 0 | 0 | 1 |
| MFSD2A | 2 | 0 | 0 | 0 | 0 | 0 | 0 | 0 | 1 |
| SLC39A5 | 2 | 0 | 0 | 0 | 0 | 0 | 0 | 0 | 1 |
| LIPK | 2 | 0 | 0 | 0 | 0 | 0 | 0 | 0 | 1 |
| LRCH4 | 2 | 0 | 0 | 0 | 0 | 0 | 0 | 0 | 1 |
| SGSH | 2 | 0 | 0 | 0 | 0 | 0 | 0 | 0 | 1 |
| HYOU1 | 1 | 1 | 0 | 0 | 0 | 0 | 0 | 0 | 1 |
| GPR56 | 2 | 0 | 0 | 0 | 0 | 0 | 0 | 0 | 1 |
| OR4X2 | 2 | 0 | 0 | 0 | 0 | 0 | 0 | 0 | 1 |
| TRO | 1 | 0 | 1 | 0 | 0 | 0 | 0 | 0 | 1 |
| ACTR3B | 2 | 0 | 0 | 0 | 0 | 0 | 0 | 0 | 1 |

| | | | | | | | | | |
|---|---|---|---|---|---|---|---|---|---|
| SLC7A9 | 2 | 0 | 0 | 0 | 0 | 0 | 0 | 0 | 1 |
| OR2AG1 | 2 | 0 | 0 | 0 | 0 | 0 | 0 | 0 | 1 |
| MAPK12 | 2 | 0 | 0 | 0 | 0 | 0 | 0 | 0 | 1 |
| NSUN7 | 2 | 0 | 0 | 0 | 0 | 0 | 0 | 0 | 1 |
| WDR88 | 1 | 1 | 0 | 0 | 0 | 0 | 0 | 0 | 1 |
| GAS2 | 2 | 0 | 0 | 0 | 0 | 0 | 0 | 0 | 1 |
| RPS6KL1 | 2 | 0 | 0 | 0 | 0 | 0 | 0 | 0 | 1 |
| IKZF4 | 2 | 0 | 0 | 0 | 0 | 0 | 0 | 0 | 1 |
| SPNS1 | 2 | 0 | 0 | 0 | 0 | 0 | 0 | 0 | 1 |
| KATNAL1 | 2 | 0 | 0 | 0 | 0 | 0 | 0 | 0 | 1 |
| GBP4 | 1 | 0 | 1 | 0 | 0 | 0 | 0 | 0 | 1 |
| GJC1 | 2 | 0 | 0 | 0 | 0 | 0 | 0 | 0 | 1 |
| FCAR | 2 | 0 | 0 | 0 | 0 | 0 | 0 | 0 | 1 |
| PDE11A | 1 | 1 | 0 | 0 | 0 | 0 | 0 | 0 | 1 |
| SLC13A5 | 2 | 0 | 0 | 0 | 0 | 0 | 0 | 0 | 1 |
| CCNJL | 2 | 0 | 0 | 0 | 0 | 0 | 0 | 0 | 1 |
| KIFC1 | 2 | 0 | 0 | 0 | 0 | 0 | 0 | 0 | 1 |
| PPM1L | 2 | 0 | 0 | 0 | 0 | 0 | 0 | 0 | 1 |
| EML3 | 1 | 0 | 1 | 0 | 0 | 0 | 0 | 0 | 1 |
| GSK3A | 1 | 1 | 0 | 0 | 0 | 0 | 0 | 0 | 1 |
| IFIT2 | 2 | 0 | 0 | 0 | 0 | 0 | 0 | 0 | 1 |
| RTKN | 2 | 0 | 0 | 0 | 0 | 0 | 0 | 0 | 1 |
| DLX5 | 2 | 0 | 0 | 0 | 0 | 0 | 0 | 0 | 1 |
| RIC8B | 2 | 0 | 0 | 0 | 0 | 0 | 0 | 0 | 1 |
| EIF3E | 2 | 0 | 0 | 0 | 0 | 0 | 0 | 0 | 1 |
| TAF8 | 2 | 0 | 0 | 0 | 0 | 0 | 0 | 0 | 1 |
| LUZP4 | 2 | 0 | 0 | 0 | 0 | 0 | 0 | 0 | 1 |
| TSPAN12 | 2 | 0 | 0 | 0 | 0 | 0 | 0 | 0 | 1 |
| SLA | 2 | 0 | 0 | 0 | 0 | 0 | 0 | 0 | 1 |
| CLK2 | 2 | 0 | 0 | 0 | 0 | 0 | 0 | 0 | 1 |
| WDR6 | 1 | 1 | 0 | 0 | 0 | 0 | 0 | 0 | 1 |
| CEACAM6 | 2 | 0 | 0 | 0 | 0 | 0 | 0 | 0 | 1 |
| NFATC2IP | 2 | 0 | 0 | 0 | 0 | 0 | 0 | 0 | 1 |
| WDR69 | 2 | 0 | 0 | 0 | 0 | 0 | 0 | 0 | 1 |
| C16orf53 | 2 | 0 | 0 | 0 | 0 | 0 | 0 | 0 | 1 |
| NEXN | 2 | 0 | 0 | 0 | 0 | 0 | 0 | 0 | 1 |
| CSAD | 2 | 0 | 0 | 0 | 0 | 0 | 0 | 0 | 1 |
| ASB4 | 2 | 0 | 0 | 0 | 0 | 0 | 0 | 0 | 1 |
| NKX1-2 | 2 | 0 | 0 | 0 | 0 | 0 | 0 | 0 | 1 |
| DDX3X | 1 | 1 | 0 | 0 | 0 | 0 | 0 | 0 | 1 |
| SNX1 | 2 | 0 | 0 | 0 | 0 | 0 | 0 | 0 | 1 |
| ZMYND12 | 2 | 0 | 0 | 0 | 0 | 0 | 0 | 0 | 1 |
| PLA2G3 | 2 | 0 | 0 | 0 | 0 | 0 | 0 | 0 | 1 |
| GIMAP7 | 1 | 1 | 0 | 0 | 0 | 0 | 0 | 0 | 1 |
| YBX2 | 2 | 0 | 0 | 0 | 0 | 0 | 0 | 0 | 1 |
| MGMT | 2 | 0 | 0 | 0 | 0 | 0 | 0 | 0 | 1 |

| | | | | | | | | | |
|---|---|---|---|---|---|---|---|---|---|
| CCDC38 | 2 | 0 | 0 | 0 | 0 | 0 | 0 | 0 | 1 |
| ALS2CR12 | 2 | 0 | 0 | 0 | 0 | 0 | 0 | 0 | 1 |
| TUBG1 | 2 | 0 | 0 | 0 | 0 | 0 | 0 | 0 | 1 |
| RASSF2 | 2 | 0 | 0 | 0 | 0 | 0 | 0 | 0 | 1 |
| HTR1E | 2 | 0 | 0 | 0 | 0 | 0 | 0 | 0 | 1 |
| TPRA1 | 2 | 0 | 0 | 0 | 0 | 0 | 0 | 0 | 1 |
| ARHGAP27 | 2 | 0 | 0 | 0 | 0 | 0 | 0 | 0 | 1 |
| TRPV2 | 2 | 0 | 0 | 0 | 0 | 0 | 0 | 0 | 1 |
| RDH16 | 2 | 0 | 0 | 0 | 0 | 0 | 0 | 0 | 1 |
| DYNC1I2 | 2 | 0 | 0 | 0 | 0 | 0 | 0 | 0 | 1 |
| SELE | 1 | 1 | 0 | 0 | 0 | 0 | 0 | 0 | 1 |
| GTDC1 | 2 | 0 | 0 | 0 | 0 | 0 | 0 | 0 | 1 |
| KIAA0020 | 2 | 0 | 0 | 0 | 0 | 0 | 0 | 0 | 1 |
| TMTC4 | 2 | 0 | 0 | 0 | 0 | 0 | 0 | 0 | 1 |
| TMPRSS4 | 2 | 0 | 0 | 0 | 0 | 0 | 0 | 0 | 1 |
| MFSD9 | 2 | 0 | 0 | 0 | 0 | 0 | 0 | 0 | 1 |
| GIPC2 | 1 | 1 | 0 | 0 | 0 | 0 | 0 | 0 | 1 |
| CLDN8 | 2 | 0 | 0 | 0 | 0 | 0 | 0 | 0 | 1 |
| HTRA1 | 2 | 0 | 0 | 0 | 0 | 0 | 0 | 0 | 1 |
| TMEM106B | 1 | 1 | 0 | 0 | 0 | 0 | 0 | 0 | 1 |
| DGKE | 2 | 0 | 0 | 0 | 0 | 0 | 0 | 0 | 1 |
| SLC39A8 | 2 | 0 | 0 | 0 | 0 | 0 | 0 | 0 | 1 |
| ALDH1A1 | 2 | 0 | 0 | 0 | 0 | 0 | 0 | 0 | 1 |
| CKAP2 | 2 | 0 | 0 | 0 | 0 | 0 | 0 | 0 | 1 |
| LHX5 | 2 | 0 | 0 | 0 | 0 | 0 | 0 | 0 | 1 |
| THEG | 2 | 0 | 0 | 0 | 0 | 0 | 0 | 0 | 1 |
| TIAL1 | 2 | 0 | 0 | 0 | 0 | 0 | 0 | 0 | 1 |
| KCNC1 | 2 | 0 | 0 | 0 | 0 | 0 | 0 | 0 | 1 |
| ZNF426 | 1 | 1 | 0 | 0 | 0 | 0 | 0 | 0 | 1 |
| SLC25A25 | 2 | 0 | 0 | 0 | 0 | 0 | 0 | 0 | 1 |
| SHQ1 | 2 | 0 | 0 | 0 | 0 | 0 | 0 | 0 | 1 |
| HACL1 | 2 | 0 | 0 | 0 | 0 | 0 | 0 | 0 | 1 |
| MBL2 | 2 | 0 | 0 | 0 | 0 | 0 | 0 | 0 | 1 |
| CPZ | 2 | 0 | 0 | 0 | 0 | 0 | 0 | 0 | 1 |
| TSC1 | 1 | 0 | 1 | 0 | 0 | 0 | 0 | 0 | 1 |
| RBAK | 2 | 0 | 0 | 0 | 0 | 0 | 0 | 0 | 1 |
| DEPDC1B | 2 | 0 | 0 | 0 | 0 | 0 | 0 | 0 | 1 |
| INTS6 | 1 | 1 | 0 | 0 | 0 | 0 | 0 | 0 | 1 |
| SUCLA2 | 2 | 0 | 0 | 0 | 0 | 0 | 0 | 0 | 1 |
| OPN3 | 2 | 0 | 0 | 0 | 0 | 0 | 0 | 0 | 1 |
| CHM | 1 | 0 | 1 | 0 | 0 | 0 | 0 | 0 | 1 |
| SMYD1 | 2 | 0 | 0 | 0 | 0 | 0 | 0 | 0 | 1 |
| DSG3 | 1 | 1 | 0 | 0 | 0 | 0 | 0 | 0 | 1 |
| GAPDHS | 2 | 0 | 0 | 0 | 0 | 0 | 0 | 0 | 1 |
| TRAF3 | 2 | 0 | 0 | 0 | 0 | 0 | 0 | 0 | 1 |
| ZC4H2 | 2 | 0 | 0 | 0 | 0 | 0 | 0 | 0 | 1 |

| | | | | | | | | | |
|---|---|---|---|---|---|---|---|---|---|
| RUVBL1 | 2 | 0 | 0 | 0 | 0 | 0 | 0 | 0 | 1 |
| CCDC8 | 2 | 0 | 0 | 0 | 0 | 0 | 0 | 0 | 1 |
| ASPN | 2 | 0 | 0 | 0 | 0 | 0 | 0 | 0 | 1 |
| XRCC4 | 2 | 0 | 0 | 0 | 0 | 0 | 0 | 0 | 1 |
| CLDN19 | 1 | 1 | 0 | 0 | 0 | 0 | 0 | 0 | 1 |
| EFHC2 | 1 | 0 | 1 | 0 | 0 | 0 | 0 | 0 | 1 |
| FAM193B | 1 | 1 | 0 | 0 | 0 | 0 | 0 | 0 | 1 |
| VSIG2 | 2 | 0 | 0 | 0 | 0 | 0 | 0 | 0 | 1 |
| TGFBI | 2 | 0 | 0 | 0 | 0 | 0 | 0 | 0 | 1 |
| CAT | 2 | 0 | 0 | 0 | 0 | 0 | 0 | 0 | 1 |
| PAX8 | 2 | 0 | 0 | 0 | 0 | 0 | 0 | 0 | 1 |
| SERPINB13 | 2 | 0 | 0 | 0 | 0 | 0 | 0 | 0 | 1 |
| RORA | 1 | 1 | 0 | 0 | 0 | 0 | 0 | 0 | 1 |
| DPH1 | 2 | 0 | 0 | 0 | 0 | 0 | 0 | 0 | 1 |
| PLEKHA2 | 2 | 0 | 0 | 0 | 0 | 0 | 0 | 0 | 1 |
| TBX4 | 2 | 0 | 0 | 0 | 0 | 0 | 0 | 0 | 1 |
| STK4 | 2 | 0 | 0 | 0 | 0 | 0 | 0 | 0 | 1 |
| NPDC1 | 2 | 0 | 0 | 0 | 0 | 0 | 0 | 0 | 1 |
| TRIM6 | 2 | 0 | 0 | 0 | 0 | 0 | 0 | 0 | 1 |
| CCDC85A | 1 | 1 | 0 | 0 | 0 | 0 | 0 | 0 | 1 |
| SLC39A11 | 2 | 0 | 0 | 0 | 0 | 0 | 0 | 0 | 1 |
| NF2 | 2 | 0 | 0 | 0 | 0 | 0 | 0 | 0 | 1 |
| FBXL3 | 2 | 0 | 0 | 0 | 0 | 0 | 0 | 0 | 1 |
| EMID2 | 2 | 0 | 0 | 0 | 0 | 0 | 0 | 0 | 1 |
| KRT40 | 2 | 0 | 0 | 0 | 0 | 0 | 0 | 0 | 1 |
| SCML4 | 2 | 0 | 0 | 0 | 0 | 0 | 0 | 0 | 1 |
| TMPRSS11E | 2 | 0 | 0 | 0 | 0 | 0 | 0 | 0 | 1 |
| WTAP | 2 | 0 | 0 | 0 | 0 | 0 | 0 | 0 | 1 |
| KIAA1467 | 2 | 0 | 0 | 0 | 0 | 0 | 0 | 0 | 1 |
| PSMD12 | 1 | 1 | 0 | 0 | 0 | 0 | 0 | 0 | 1 |
| C14orf93 | 2 | 0 | 0 | 0 | 0 | 0 | 0 | 0 | 1 |
| ANGEL2 | 2 | 0 | 0 | 0 | 0 | 0 | 0 | 0 | 1 |
| ZFP82 | 2 | 0 | 0 | 0 | 0 | 0 | 0 | 0 | 1 |
| PANX3 | 2 | 0 | 0 | 0 | 0 | 0 | 0 | 0 | 1 |
| TRAPPC10 | 1 | 0 | 1 | 0 | 0 | 0 | 0 | 0 | 1 |
| MFAP5 | 2 | 0 | 0 | 0 | 0 | 0 | 0 | 0 | 1 |
| FDXR | 2 | 0 | 0 | 0 | 0 | 0 | 0 | 0 | 1 |
| OR4D5 | 2 | 0 | 0 | 0 | 0 | 0 | 0 | 0 | 1 |
| DAO | 2 | 0 | 0 | 0 | 0 | 0 | 0 | 0 | 1 |
| FMOD | 2 | 0 | 0 | 0 | 0 | 0 | 0 | 0 | 1 |
| PGGT1B | 2 | 0 | 0 | 0 | 0 | 0 | 0 | 0 | 1 |
| DEDD2 | 2 | 0 | 0 | 0 | 0 | 0 | 0 | 0 | 1 |
| HSD3B1 | 2 | 0 | 0 | 0 | 0 | 0 | 0 | 0 | 1 |
| RASSF6 | 2 | 0 | 0 | 0 | 0 | 0 | 0 | 0 | 1 |
| ADH1A | 2 | 0 | 0 | 0 | 0 | 0 | 0 | 0 | 1 |
| HAT1 | 2 | 0 | 0 | 0 | 0 | 0 | 0 | 0 | 1 |

| | | | | | | | | | |
|---|---|---|---|---|---|---|---|---|---|
| RIT2 | 2 | 0 | 0 | 0 | 0 | 0 | 0 | 0 | 1 |
| SETD4 | 2 | 0 | 0 | 0 | 0 | 0 | 0 | 0 | 1 |
| EXOC8 | 2 | 0 | 0 | 0 | 0 | 0 | 0 | 0 | 1 |
| SATB1 | 1 | 1 | 0 | 0 | 0 | 0 | 0 | 0 | 1 |
| VSIG10 | 2 | 0 | 0 | 0 | 0 | 0 | 0 | 0 | 1 |
| PINK1 | 2 | 0 | 0 | 0 | 0 | 0 | 0 | 0 | 1 |
| C5orf54 | 2 | 0 | 0 | 0 | 0 | 0 | 0 | 0 | 1 |
| TKTL1 | 2 | 0 | 0 | 0 | 0 | 0 | 0 | 0 | 1 |
| PALM2 | 2 | 0 | 0 | 0 | 0 | 0 | 0 | 0 | 1 |
| ARFGAP3 | 2 | 0 | 0 | 0 | 0 | 0 | 0 | 0 | 1 |
| PRDM11 | 2 | 0 | 0 | 0 | 0 | 0 | 0 | 0 | 1 |
| P4HA3 | 2 | 0 | 0 | 0 | 0 | 0 | 0 | 0 | 1 |
| PROCR | 2 | 0 | 0 | 0 | 0 | 0 | 0 | 0 | 1 |
| PARP6 | 2 | 0 | 0 | 0 | 0 | 0 | 0 | 0 | 1 |
| ANKZF1 | 2 | 0 | 0 | 0 | 0 | 0 | 0 | 0 | 1 |
| DTNB | 2 | 0 | 0 | 0 | 0 | 0 | 0 | 0 | 1 |
| LMCD1 | 2 | 0 | 0 | 0 | 0 | 0 | 0 | 0 | 1 |
| IGF2BP1 | 2 | 0 | 0 | 0 | 0 | 0 | 0 | 0 | 1 |
| PARM1 | 2 | 0 | 0 | 0 | 0 | 0 | 0 | 0 | 1 |
| FUS | 2 | 0 | 0 | 0 | 0 | 0 | 0 | 0 | 1 |
| AGAP3 | 1 | 1 | 0 | 0 | 0 | 0 | 0 | 0 | 1 |
| RBBP4 | 2 | 0 | 0 | 0 | 0 | 0 | 0 | 0 | 1 |
| NOSTRIN | 2 | 0 | 0 | 0 | 0 | 0 | 0 | 0 | 1 |
| MBOAT2 | 2 | 0 | 0 | 0 | 0 | 0 | 0 | 0 | 1 |
| SLC2A9 | 2 | 0 | 0 | 0 | 0 | 0 | 0 | 0 | 1 |
| KIAA1804 | 1 | 1 | 0 | 0 | 0 | 0 | 0 | 0 | 1 |
| ACTR5 | 2 | 0 | 0 | 0 | 0 | 0 | 0 | 0 | 1 |
| ETV7 | 2 | 0 | 0 | 0 | 0 | 0 | 0 | 0 | 1 |
| PDE4C | 2 | 0 | 0 | 0 | 0 | 0 | 0 | 0 | 1 |
| TACR1 | 2 | 0 | 0 | 0 | 0 | 0 | 0 | 0 | 1 |
| TRMT2B | 2 | 0 | 0 | 0 | 0 | 0 | 0 | 0 | 1 |
| GABRG3 | 2 | 0 | 0 | 0 | 0 | 0 | 0 | 0 | 1 |
| GPR39 | 2 | 0 | 0 | 0 | 0 | 0 | 0 | 0 | 1 |
| CCDC87 | 1 | 1 | 0 | 0 | 0 | 0 | 0 | 0 | 1 |
| FAM126A | 2 | 0 | 0 | 0 | 0 | 0 | 0 | 0 | 1 |
| ZMYND10 | 2 | 0 | 0 | 0 | 0 | 0 | 0 | 0 | 1 |
| DRP2 | 1 | 1 | 0 | 0 | 0 | 0 | 0 | 0 | 1 |
| MYOZ2 | 2 | 0 | 0 | 0 | 0 | 0 | 0 | 0 | 1 |
| MDFIC | 2 | 0 | 0 | 0 | 0 | 0 | 0 | 0 | 1 |
| RSC1A1 | 2 | 0 | 0 | 0 | 0 | 0 | 0 | 0 | 1 |
| CYP3A43 | 2 | 0 | 0 | 0 | 0 | 0 | 0 | 0 | 1 |
| OR11H4 | 2 | 0 | 0 | 0 | 0 | 0 | 0 | 0 | 1 |
| C7orf10 | 2 | 0 | 0 | 0 | 0 | 0 | 0 | 0 | 1 |
| OR7D4 | 2 | 0 | 0 | 0 | 0 | 0 | 0 | 0 | 1 |
| FAM116B | 2 | 0 | 0 | 0 | 0 | 0 | 0 | 0 | 1 |
| GRK4 | 2 | 0 | 0 | 0 | 0 | 0 | 0 | 0 | 1 |

| | | | | | | | | | |
|---|---|---|---|---|---|---|---|---|---|
| LAG3 | 2 | 0 | 0 | 0 | 0 | 0 | 0 | 0 | 1 |
| ZNF662 | 2 | 0 | 0 | 0 | 0 | 0 | 0 | 0 | 1 |
| VNN2 | 2 | 0 | 0 | 0 | 0 | 0 | 0 | 0 | 1 |
| FNBP1 | 2 | 0 | 0 | 0 | 0 | 0 | 0 | 0 | 1 |
| VEPH1 | 1 | 1 | 0 | 0 | 0 | 0 | 0 | 0 | 1 |
| ELMO3 | 2 | 0 | 0 | 0 | 0 | 0 | 0 | 0 | 1 |
| GRHL2 | 1 | 0 | 0 | 0 | 1 | 0 | 0 | 0 | 1 |
| KCNA10 | 2 | 0 | 0 | 0 | 0 | 0 | 0 | 0 | 1 |
| OR6C2 | 2 | 0 | 0 | 0 | 0 | 0 | 0 | 0 | 1 |
| TSKS | 2 | 0 | 0 | 0 | 0 | 0 | 0 | 0 | 1 |
| SMARCC1 | 1 | 1 | 0 | 0 | 0 | 0 | 0 | 0 | 1 |
| C3orf23 | 2 | 0 | 0 | 0 | 0 | 0 | 0 | 0 | 1 |
| GABPB1 | 2 | 0 | 0 | 0 | 0 | 0 | 0 | 0 | 1 |
| SYCE1 | 2 | 0 | 0 | 0 | 0 | 0 | 0 | 0 | 1 |
| PRAMEF12 | 1 | 1 | 0 | 0 | 0 | 0 | 0 | 0 | 1 |
| TNFAIP2 | 2 | 0 | 0 | 0 | 0 | 0 | 0 | 0 | 1 |
| CCDC117 | 2 | 0 | 0 | 0 | 0 | 0 | 0 | 0 | 1 |
| IFRD2 | 2 | 0 | 0 | 0 | 0 | 0 | 0 | 0 | 1 |
| ZNF169 | 2 | 0 | 0 | 0 | 0 | 0 | 0 | 0 | 1 |
| APOH | 2 | 0 | 0 | 0 | 0 | 0 | 0 | 0 | 1 |
| OTOP3 | 2 | 0 | 0 | 0 | 0 | 0 | 0 | 0 | 1 |
| CORO6 | 2 | 0 | 0 | 0 | 0 | 0 | 0 | 0 | 1 |
| RRM2B | 2 | 0 | 0 | 0 | 0 | 0 | 0 | 0 | 1 |
| NRP2 | 1 | 0 | 1 | 0 | 0 | 0 | 0 | 0 | 1 |
| ZNF692 | 1 | 1 | 0 | 0 | 0 | 0 | 0 | 0 | 1 |
| SLC37A3 | 2 | 0 | 0 | 0 | 0 | 0 | 0 | 0 | 1 |
| TMEM174 | 2 | 0 | 0 | 0 | 0 | 0 | 0 | 0 | 1 |
| TECTB | 2 | 0 | 0 | 0 | 0 | 0 | 0 | 0 | 1 |
| BUB1 | 1 | 1 | 0 | 0 | 0 | 0 | 0 | 0 | 1 |
| GORASP2 | 2 | 0 | 0 | 0 | 0 | 0 | 0 | 0 | 1 |
| IL22RA1 | 2 | 0 | 0 | 0 | 0 | 0 | 0 | 0 | 1 |
| BSDC1 | 2 | 0 | 0 | 0 | 0 | 0 | 0 | 0 | 1 |
| C4orf17 | 1 | 0 | 0 | 0 | 1 | 0 | 0 | 0 | 1 |
| ULK3 | 2 | 0 | 0 | 0 | 0 | 0 | 0 | 0 | 1 |
| RNF103 | 2 | 0 | 0 | 0 | 0 | 0 | 0 | 0 | 1 |
| ISX | 2 | 0 | 0 | 0 | 0 | 0 | 0 | 0 | 1 |
| CRTC3 | 2 | 0 | 0 | 0 | 0 | 0 | 0 | 0 | 1 |
| KRT13 | 1 | 1 | 0 | 0 | 0 | 0 | 0 | 0 | 1 |
| KATNAL2 | 2 | 0 | 0 | 0 | 0 | 0 | 0 | 0 | 1 |
| SLC22A8 | 2 | 0 | 0 | 0 | 0 | 0 | 0 | 0 | 1 |
| BTN2A2 | 2 | 0 | 0 | 0 | 0 | 0 | 0 | 0 | 1 |
| SLC43A2 | 2 | 0 | 0 | 0 | 0 | 0 | 0 | 0 | 1 |
| ELP4 | 2 | 0 | 0 | 0 | 0 | 0 | 0 | 0 | 1 |
| CARM1 | 2 | 0 | 0 | 0 | 0 | 0 | 0 | 0 | 1 |
| KIAA1033 | 1 | 1 | 0 | 0 | 0 | 0 | 0 | 0 | 1 |
| GABRG1 | 2 | 0 | 0 | 0 | 0 | 0 | 0 | 0 | 1 |

| | | | | | | | | | |
|---|---|---|---|---|---|---|---|---|---|
| BFSP2 | 2 | 0 | 0 | 0 | 0 | 0 | 0 | 0 | 1 |
| CNST | 2 | 0 | 0 | 0 | 0 | 0 | 0 | 0 | 1 |
| UROD | 1 | 0 | 0 | 0 | 0 | 0 | 1 | 0 | 1 |
| ITGA2B | 0 | 1 | 0 | 0 | 1 | 0 | 0 | 0 | 1 |
| FAM63B | 1 | 1 | 0 | 0 | 0 | 0 | 0 | 0 | 1 |
| FAAH2 | 2 | 0 | 0 | 0 | 0 | 0 | 0 | 0 | 1 |
| DPEP3 | 2 | 0 | 0 | 0 | 0 | 0 | 0 | 0 | 1 |
| PPM1B | 2 | 0 | 0 | 0 | 0 | 0 | 0 | 0 | 1 |
| C10orf47 | 2 | 0 | 0 | 0 | 0 | 0 | 0 | 0 | 1 |
| KIAA0141 | 2 | 0 | 0 | 0 | 0 | 0 | 0 | 0 | 1 |
| DCX | 2 | 0 | 0 | 0 | 0 | 0 | 0 | 0 | 1 |
| FPR2 | 2 | 0 | 0 | 0 | 0 | 0 | 0 | 0 | 1 |
| TRIP4 | 2 | 0 | 0 | 0 | 0 | 0 | 0 | 0 | 1 |
| MDM1 | 2 | 0 | 0 | 0 | 0 | 0 | 0 | 0 | 1 |
| CLCN4 | 2 | 0 | 0 | 0 | 0 | 0 | 0 | 0 | 1 |
| HBP1 | 2 | 0 | 0 | 0 | 0 | 0 | 0 | 0 | 1 |
| ADRA1D | 2 | 0 | 0 | 0 | 0 | 0 | 0 | 0 | 1 |
| OC90 | 2 | 0 | 0 | 0 | 0 | 0 | 0 | 0 | 1 |
| PKD2L2 | 2 | 0 | 0 | 0 | 0 | 0 | 0 | 0 | 1 |
| OFD1 | 2 | 0 | 0 | 0 | 0 | 0 | 0 | 0 | 1 |
| VWA1 | 2 | 0 | 0 | 0 | 0 | 0 | 0 | 0 | 1 |
| NR3C1 | 2 | 0 | 0 | 0 | 0 | 0 | 0 | 0 | 1 |
| CCDC77 | 1 | 1 | 0 | 0 | 0 | 0 | 0 | 0 | 1 |
| TRAF2 | 2 | 0 | 0 | 0 | 0 | 0 | 0 | 0 | 1 |
| SLC2A4 | 2 | 0 | 0 | 0 | 0 | 0 | 0 | 0 | 1 |
| ECM2 | 2 | 0 | 0 | 0 | 0 | 0 | 0 | 0 | 1 |
| LACTB | 2 | 0 | 0 | 0 | 0 | 0 | 0 | 0 | 1 |
| PRC1 | 2 | 0 | 0 | 0 | 0 | 0 | 0 | 0 | 1 |
| ADPRH | 2 | 0 | 0 | 0 | 0 | 0 | 0 | 0 | 1 |
| RSPRY1 | 2 | 0 | 0 | 0 | 0 | 0 | 0 | 0 | 1 |
| MAGED2 | 1 | 1 | 0 | 0 | 0 | 0 | 0 | 0 | 1 |
| MOXD1 | 2 | 0 | 0 | 0 | 0 | 0 | 0 | 0 | 1 |
| KIAA0907 | 2 | 0 | 0 | 0 | 0 | 0 | 0 | 0 | 1 |
| ACPT | 2 | 0 | 0 | 0 | 0 | 0 | 0 | 0 | 1 |
| IL1RL1 | 2 | 0 | 0 | 0 | 0 | 0 | 0 | 0 | 1 |
| PNLIPRP2 | 2 | 0 | 0 | 0 | 0 | 0 | 0 | 0 | 1 |
| BCL2L14 | 2 | 0 | 0 | 0 | 0 | 0 | 0 | 0 | 1 |
| SLC7A4 | 2 | 0 | 0 | 0 | 0 | 0 | 0 | 0 | 1 |
| TRA2B | 2 | 0 | 0 | 0 | 0 | 0 | 0 | 0 | 1 |
| ENTPD6 | 2 | 0 | 0 | 0 | 0 | 0 | 0 | 0 | 1 |
| ANKLE1 | 2 | 0 | 0 | 0 | 0 | 0 | 0 | 0 | 1 |
| UST | 2 | 0 | 0 | 0 | 0 | 0 | 0 | 0 | 1 |
| AZIN1 | 2 | 0 | 0 | 0 | 0 | 0 | 0 | 0 | 1 |
| OR4K17 | 2 | 0 | 0 | 0 | 0 | 0 | 0 | 0 | 1 |
| ZNF791 | 2 | 0 | 0 | 0 | 0 | 0 | 0 | 0 | 1 |
| TMOD2 | 2 | 0 | 0 | 0 | 0 | 0 | 0 | 0 | 1 |

| | | | | | | | | | |
|---|---|---|---|---|---|---|---|---|---|
| MNDA | 2 | 0 | 0 | 0 | 0 | 0 | 0 | 0 | 1 |
| WT1 | 2 | 0 | 0 | 0 | 0 | 0 | 0 | 0 | 1 |
| SAMSN1 | 1 | 1 | 0 | 0 | 0 | 0 | 0 | 0 | 1 |
| WIF1 | 2 | 0 | 0 | 0 | 0 | 0 | 0 | 0 | 1 |
| SLC17A7 | 2 | 0 | 0 | 0 | 0 | 0 | 0 | 0 | 1 |
| ZDHHC2 | 2 | 0 | 0 | 0 | 0 | 0 | 0 | 0 | 1 |
| MEOX2 | 2 | 0 | 0 | 0 | 0 | 0 | 0 | 0 | 1 |
| DDB2 | 2 | 0 | 0 | 0 | 0 | 0 | 0 | 0 | 1 |
| CAMK2G | 2 | 0 | 0 | 0 | 0 | 0 | 0 | 0 | 1 |
| DCAF6 | 1 | 1 | 0 | 0 | 0 | 0 | 0 | 0 | 1 |
| OR52N5 | 2 | 0 | 0 | 0 | 0 | 0 | 0 | 0 | 1 |
| MMP1 | 2 | 0 | 0 | 0 | 0 | 0 | 0 | 0 | 1 |
| MUC15 | 2 | 0 | 0 | 0 | 0 | 0 | 0 | 0 | 1 |
| JRK | 2 | 0 | 0 | 0 | 0 | 0 | 0 | 0 | 1 |
| ZNF26 | 2 | 0 | 0 | 0 | 0 | 0 | 0 | 0 | 1 |
| SMAD4 | 2 | 0 | 0 | 0 | 0 | 0 | 0 | 0 | 1 |
| PACRG | 2 | 0 | 0 | 0 | 0 | 0 | 0 | 0 | 1 |
| ADD3 | 2 | 0 | 0 | 0 | 0 | 0 | 0 | 0 | 1 |
| PIGK | 2 | 0 | 0 | 0 | 0 | 0 | 0 | 0 | 1 |
| C15orf52 | 2 | 0 | 0 | 0 | 0 | 0 | 0 | 0 | 1 |
| CPN1 | 2 | 0 | 0 | 0 | 0 | 0 | 0 | 0 | 1 |
| GTF2E1 | 2 | 0 | 0 | 0 | 0 | 0 | 0 | 0 | 1 |
| TMEM201 | 2 | 0 | 0 | 0 | 0 | 0 | 0 | 0 | 1 |
| CWF19L1 | 2 | 0 | 0 | 0 | 0 | 0 | 0 | 0 | 1 |
| POFUT1 | 2 | 0 | 0 | 0 | 0 | 0 | 0 | 0 | 1 |
| PRDM1 | 2 | 0 | 0 | 0 | 0 | 0 | 0 | 0 | 1 |
| CAMK2D | 2 | 0 | 0 | 0 | 0 | 0 | 0 | 0 | 1 |
| FBXO31 | 2 | 0 | 0 | 0 | 0 | 0 | 0 | 0 | 1 |
| IPP | 2 | 0 | 0 | 0 | 0 | 0 | 0 | 0 | 1 |
| QKI | 2 | 0 | 0 | 0 | 0 | 0 | 0 | 0 | 1 |
| EVX2 | 2 | 0 | 0 | 0 | 0 | 0 | 0 | 0 | 1 |
| GABRA2 | 2 | 0 | 0 | 0 | 0 | 0 | 0 | 0 | 1 |
| LONRF1 | 1 | 1 | 0 | 0 | 0 | 0 | 0 | 0 | 1 |
| PARD6G | 2 | 0 | 0 | 0 | 0 | 0 | 0 | 0 | 1 |
| MKS1 | 2 | 0 | 0 | 0 | 0 | 0 | 0 | 0 | 1 |
| CLUL1 | 2 | 0 | 0 | 0 | 0 | 0 | 0 | 0 | 1 |
| SPTY2D1 | 2 | 0 | 0 | 0 | 0 | 0 | 0 | 0 | 1 |
| NUFIP1 | 2 | 0 | 0 | 0 | 0 | 0 | 0 | 0 | 1 |
| DARS | 2 | 0 | 0 | 0 | 0 | 0 | 0 | 0 | 1 |
| CBS | 2 | 0 | 0 | 0 | 0 | 0 | 0 | 0 | 1 |
| TAS1R1 | 2 | 0 | 0 | 0 | 0 | 0 | 0 | 0 | 1 |
| MARVELD2 | 2 | 0 | 0 | 0 | 0 | 0 | 0 | 0 | 1 |
| SLC25A24 | 2 | 0 | 0 | 0 | 0 | 0 | 0 | 0 | 1 |
| COCH | 2 | 0 | 0 | 0 | 0 | 0 | 0 | 0 | 1 |
| ARG1 | 2 | 0 | 0 | 0 | 0 | 0 | 0 | 0 | 1 |
| IKBIP | 2 | 0 | 0 | 0 | 0 | 0 | 0 | 0 | 1 |

| | | | | | | | | | |
|---|---|---|---|---|---|---|---|---|---|
| ZNF630 | 2 | 0 | 0 | 0 | 0 | 0 | 0 | 0 | 1 |
| ACRBP | 2 | 0 | 0 | 0 | 0 | 0 | 0 | 0 | 1 |
| KRT32 | 2 | 0 | 0 | 0 | 0 | 0 | 0 | 0 | 1 |
| MYF5 | 2 | 0 | 0 | 0 | 0 | 0 | 0 | 0 | 1 |
| MBTPS2 | 2 | 0 | 0 | 0 | 0 | 0 | 0 | 0 | 1 |
| CLIP4 | 2 | 0 | 0 | 0 | 0 | 0 | 0 | 0 | 1 |
| KRT71 | 2 | 0 | 0 | 0 | 0 | 0 | 0 | 0 | 1 |
| SYT5 | 2 | 0 | 0 | 0 | 0 | 0 | 0 | 0 | 1 |
| PDE9A | 2 | 0 | 0 | 0 | 0 | 0 | 0 | 0 | 1 |
| OR9A2 | 2 | 0 | 0 | 0 | 0 | 0 | 0 | 0 | 1 |
| C16orf78 | 2 | 0 | 0 | 0 | 0 | 0 | 0 | 0 | 1 |
| SLC22A12 | 2 | 0 | 0 | 0 | 0 | 0 | 0 | 0 | 1 |
| ARHGAP30 | 1 | 1 | 0 | 0 | 0 | 0 | 0 | 0 | 1 |
| NKAP | 2 | 0 | 0 | 0 | 0 | 0 | 0 | 0 | 1 |
| PRKAA1 | 2 | 0 | 0 | 0 | 0 | 0 | 0 | 0 | 1 |
| TSPYL5 | 2 | 0 | 0 | 0 | 0 | 0 | 0 | 0 | 1 |
| FOXN4 | 2 | 0 | 0 | 0 | 0 | 0 | 0 | 0 | 1 |
| TGFB1I1 | 2 | 0 | 0 | 0 | 0 | 0 | 0 | 0 | 1 |
| PLA2G7 | 2 | 0 | 0 | 0 | 0 | 0 | 0 | 0 | 1 |
| NR0B1 | 1 | 1 | 0 | 0 | 0 | 0 | 0 | 0 | 1 |
| MCRS1 | 2 | 0 | 0 | 0 | 0 | 0 | 0 | 0 | 1 |
| LMAN1L | 2 | 0 | 0 | 0 | 0 | 0 | 0 | 0 | 1 |
| ATG16L2 | 2 | 0 | 0 | 0 | 0 | 0 | 0 | 0 | 1 |
| NCKIPSD | 2 | 0 | 0 | 0 | 0 | 0 | 0 | 0 | 1 |
| FZD10 | 2 | 0 | 0 | 0 | 0 | 0 | 0 | 0 | 1 |
| MBIP | 2 | 0 | 0 | 0 | 0 | 0 | 0 | 0 | 1 |
| FOXJ3 | 2 | 0 | 0 | 0 | 0 | 0 | 0 | 0 | 1 |
| PANK2 | 2 | 0 | 0 | 0 | 0 | 0 | 0 | 0 | 1 |
| PASD1 | 2 | 0 | 0 | 0 | 0 | 0 | 0 | 0 | 1 |
| NAAA | 2 | 0 | 0 | 0 | 0 | 0 | 0 | 0 | 1 |
| MATN1 | 2 | 0 | 0 | 0 | 0 | 0 | 0 | 0 | 1 |
| MRGPRX2 | 2 | 0 | 0 | 0 | 0 | 0 | 0 | 0 | 1 |
| BBOX1 | 2 | 0 | 0 | 0 | 0 | 0 | 0 | 0 | 1 |
| OR52R1 | 2 | 0 | 0 | 0 | 0 | 0 | 0 | 0 | 1 |
| OR8G5 | 2 | 0 | 0 | 0 | 0 | 0 | 0 | 0 | 1 |
| KIAA1984 | 2 | 0 | 0 | 0 | 0 | 0 | 0 | 0 | 1 |
| SPRY4 | 2 | 0 | 0 | 0 | 0 | 0 | 0 | 0 | 1 |
| TNIP1 | 2 | 0 | 0 | 0 | 0 | 0 | 0 | 0 | 1 |
| TERF2 | 2 | 0 | 0 | 0 | 0 | 0 | 0 | 0 | 1 |
| GTF3C5 | 2 | 0 | 0 | 0 | 0 | 0 | 0 | 0 | 1 |
| FAM117B | 2 | 0 | 0 | 0 | 0 | 0 | 0 | 0 | 1 |
| CHRNG | 2 | 0 | 0 | 0 | 0 | 0 | 0 | 0 | 1 |
| LOC646851 | 2 | 0 | 0 | 0 | 0 | 0 | 0 | 0 | 1 |
| ADH7 | 2 | 0 | 0 | 0 | 0 | 0 | 0 | 0 | 1 |
| ACTBL2 | 2 | 0 | 0 | 0 | 0 | 0 | 0 | 0 | 1 |
| PPP1R9B | 2 | 0 | 0 | 0 | 0 | 0 | 0 | 0 | 1 |

| | | | | | | | | | |
|---|---|---|---|---|---|---|---|---|---|
| HIP1 | 1 | 1 | 0 | 0 | 0 | 0 | 0 | 0 | 1 |
| PHACTR2 | 2 | 0 | 0 | 0 | 0 | 0 | 0 | 0 | 1 |
| SHISA6 | 2 | 0 | 0 | 0 | 0 | 0 | 0 | 0 | 1 |
| RASGEF1C | 2 | 0 | 0 | 0 | 0 | 0 | 0 | 0 | 1 |
| ZNF790 | 2 | 0 | 0 | 0 | 0 | 0 | 0 | 0 | 1 |
| ENAH | 2 | 0 | 0 | 0 | 0 | 0 | 0 | 0 | 1 |
| C10orf137 | 1 | 1 | 0 | 0 | 0 | 0 | 0 | 0 | 1 |
| DEPDC7 | 2 | 0 | 0 | 0 | 0 | 0 | 0 | 0 | 1 |
| SPG20 | 1 | 1 | 0 | 0 | 0 | 0 | 0 | 0 | 1 |
| IGF1 | 2 | 0 | 0 | 0 | 0 | 0 | 0 | 0 | 1 |
| MMEL1 | 1 | 0 | 1 | 0 | 0 | 0 | 0 | 0 | 1 |
| CAP2 | 2 | 0 | 0 | 0 | 0 | 0 | 0 | 0 | 1 |
| OR51E1 | 2 | 0 | 0 | 0 | 0 | 0 | 0 | 0 | 1 |
| OR5A2 | 2 | 0 | 0 | 0 | 0 | 0 | 0 | 0 | 1 |
| ZFP57 | 2 | 0 | 0 | 0 | 0 | 0 | 0 | 0 | 1 |
| UBASH3A | 2 | 0 | 0 | 0 | 0 | 0 | 0 | 0 | 1 |
| SPDYC | 2 | 0 | 0 | 0 | 0 | 0 | 0 | 0 | 1 |
| LSG1 | 2 | 0 | 0 | 0 | 0 | 0 | 0 | 0 | 1 |
| FAM200B | 2 | 0 | 0 | 0 | 0 | 0 | 0 | 0 | 1 |
| OR10A4 | 1 | 0 | 0 | 0 | 1 | 0 | 0 | 0 | 1 |
| TSNARE1 | 2 | 0 | 0 | 0 | 0 | 0 | 0 | 0 | 1 |
| COASY | 2 | 0 | 0 | 0 | 0 | 0 | 0 | 0 | 1 |
| TNFSF4 | 2 | 0 | 0 | 0 | 0 | 0 | 0 | 0 | 1 |
| C6orf165 | 2 | 0 | 0 | 0 | 0 | 0 | 0 | 0 | 1 |
| KLC4 | 2 | 0 | 0 | 0 | 0 | 0 | 0 | 0 | 1 |
| ACPP | 2 | 0 | 0 | 0 | 0 | 0 | 0 | 0 | 1 |
| FAM124B | 2 | 0 | 0 | 0 | 0 | 0 | 0 | 0 | 1 |
| CAMK2B | 2 | 0 | 0 | 0 | 0 | 0 | 0 | 0 | 1 |
| REPS1 | 2 | 0 | 0 | 0 | 0 | 0 | 0 | 0 | 1 |
| SEC61A1 | 2 | 0 | 0 | 0 | 0 | 0 | 0 | 0 | 1 |
| GPR85 | 2 | 0 | 0 | 0 | 0 | 0 | 0 | 0 | 1 |
| NOM1 | 1 | 0 | 0 | 0 | 1 | 0 | 0 | 0 | 1 |
| XPOT | 2 | 0 | 0 | 0 | 0 | 0 | 0 | 0 | 1 |
| UHRF1 | 2 | 0 | 0 | 0 | 0 | 0 | 0 | 0 | 1 |
| F2 | 2 | 0 | 0 | 0 | 0 | 0 | 0 | 0 | 1 |
| MCCC1 | 2 | 0 | 0 | 0 | 0 | 0 | 0 | 0 | 1 |
| TMEM8A | 2 | 0 | 0 | 0 | 0 | 0 | 0 | 0 | 1 |
| OXSR1 | 2 | 0 | 0 | 0 | 0 | 0 | 0 | 0 | 1 |
| SNX29 | 2 | 0 | 0 | 0 | 0 | 0 | 0 | 0 | 1 |
| ZCWPW1 | 2 | 0 | 0 | 0 | 0 | 0 | 0 | 0 | 1 |
| PDP1 | 2 | 0 | 0 | 0 | 0 | 0 | 0 | 0 | 1 |
| ZNF486 | 2 | 0 | 0 | 0 | 0 | 0 | 0 | 0 | 1 |
| CCDC22 | 2 | 0 | 0 | 0 | 0 | 0 | 0 | 0 | 1 |
| CFHR2 | 2 | 0 | 0 | 0 | 0 | 0 | 0 | 0 | 1 |
| TMEM63A | 2 | 0 | 0 | 0 | 0 | 0 | 0 | 0 | 1 |
| ZMAT4 | 2 | 0 | 0 | 0 | 0 | 0 | 0 | 0 | 1 |

| | | | | | | | | | |
|---|---|---|---|---|---|---|---|---|---|
| CCDC149 | 2 | 0 | 0 | 0 | 0 | 0 | 0 | 0 | 1 |
| CHRNA2 | 2 | 0 | 0 | 0 | 0 | 0 | 0 | 0 | 1 |
| F9 | 2 | 0 | 0 | 0 | 0 | 0 | 0 | 0 | 1 |
| KRT35 | 2 | 0 | 0 | 0 | 0 | 0 | 0 | 0 | 1 |
| DPEP2 | 2 | 0 | 0 | 0 | 0 | 0 | 0 | 0 | 1 |
| ALPL | 2 | 0 | 0 | 0 | 0 | 0 | 0 | 0 | 1 |
| PGM1 | 2 | 0 | 0 | 0 | 0 | 0 | 0 | 0 | 1 |
| COL23A1 | 2 | 0 | 0 | 0 | 0 | 0 | 0 | 0 | 1 |
| LRTM2 | 2 | 0 | 0 | 0 | 0 | 0 | 0 | 0 | 1 |
| WDR63 | 1 | 1 | 0 | 0 | 0 | 0 | 0 | 0 | 1 |
| SLC25A45 | 2 | 0 | 0 | 0 | 0 | 0 | 0 | 0 | 1 |
| TUBAL3 | 2 | 0 | 0 | 0 | 0 | 0 | 0 | 0 | 1 |
| EDA2R | 2 | 0 | 0 | 0 | 0 | 0 | 0 | 0 | 1 |
| PIP5K1B | 2 | 0 | 0 | 0 | 0 | 0 | 0 | 0 | 1 |
| TMEM62 | 2 | 0 | 0 | 0 | 0 | 0 | 0 | 0 | 1 |
| CECR1 | 2 | 0 | 0 | 0 | 0 | 0 | 0 | 0 | 1 |
| PLBD1 | 2 | 0 | 0 | 0 | 0 | 0 | 0 | 0 | 1 |
| TMEM181 | 2 | 0 | 0 | 0 | 0 | 0 | 0 | 0 | 1 |
| SRP54 | 2 | 0 | 0 | 0 | 0 | 0 | 0 | 0 | 1 |
| IPO5 | 1 | 1 | 0 | 0 | 0 | 0 | 0 | 0 | 1 |
| CYP2S1 | 2 | 0 | 0 | 0 | 0 | 0 | 0 | 0 | 1 |
| ZNF766 | 2 | 0 | 0 | 0 | 0 | 0 | 0 | 0 | 1 |
| CRX | 2 | 0 | 0 | 0 | 0 | 0 | 0 | 0 | 1 |
| LUZP2 | 2 | 0 | 0 | 0 | 0 | 0 | 0 | 0 | 1 |
| ZC3H18 | 1 | 1 | 0 | 0 | 0 | 0 | 0 | 0 | 1 |
| GCKR | 2 | 0 | 0 | 0 | 0 | 0 | 0 | 0 | 1 |
| CPA6 | 2 | 0 | 0 | 0 | 0 | 0 | 0 | 0 | 1 |
| HNRNPM | 1 | 0 | 0 | 0 | 1 | 0 | 0 | 0 | 1 |
| DMRT2 | 2 | 0 | 0 | 0 | 0 | 0 | 0 | 0 | 1 |
| PAK3 | 2 | 0 | 0 | 0 | 0 | 0 | 0 | 0 | 1 |
| CSTF1 | 2 | 0 | 0 | 0 | 0 | 0 | 0 | 0 | 1 |
| CHN2 | 2 | 0 | 0 | 0 | 0 | 0 | 0 | 0 | 1 |
| ITGB1 | 1 | 0 | 1 | 0 | 0 | 0 | 0 | 0 | 1 |
| AADAT | 2 | 0 | 0 | 0 | 0 | 0 | 0 | 0 | 1 |
| PEPD | 2 | 0 | 0 | 0 | 0 | 0 | 0 | 0 | 1 |
| PRICKLE3 | 2 | 0 | 0 | 0 | 0 | 0 | 0 | 0 | 1 |
| SIRPA | 2 | 0 | 0 | 0 | 0 | 0 | 0 | 0 | 1 |
| ZSCAN2 | 2 | 0 | 0 | 0 | 0 | 0 | 0 | 0 | 1 |
| SLC25A16 | 2 | 0 | 0 | 0 | 0 | 0 | 0 | 0 | 1 |
| KPRP | 2 | 0 | 0 | 0 | 0 | 0 | 0 | 0 | 1 |
| ERMAP | 2 | 0 | 0 | 0 | 0 | 0 | 0 | 0 | 1 |
| RAI2 | 2 | 0 | 0 | 0 | 0 | 0 | 0 | 0 | 1 |
| CCDC155 | 2 | 0 | 0 | 0 | 0 | 0 | 0 | 0 | 1 |
| KIAA0391 | 2 | 0 | 0 | 0 | 0 | 0 | 0 | 0 | 1 |
| ZNF565 | 2 | 0 | 0 | 0 | 0 | 0 | 0 | 0 | 1 |
| OR6K3 | 2 | 0 | 0 | 0 | 0 | 0 | 0 | 0 | 1 |

| | | | | | | | | | |
|---|---|---|---|---|---|---|---|---|---|
| POM121L12 | 2 | 0 | 0 | 0 | 0 | 0 | 0 | 0 | 1 |
| AFM | 2 | 0 | 0 | 0 | 0 | 0 | 0 | 0 | 1 |
| CHRDL2 | 2 | 0 | 0 | 0 | 0 | 0 | 0 | 0 | 1 |
| PTGS1 | 2 | 0 | 0 | 0 | 0 | 0 | 0 | 0 | 1 |
| ZNF502 | 2 | 0 | 0 | 0 | 0 | 0 | 0 | 0 | 1 |
| SMARCD1 | 1 | 0 | 1 | 0 | 0 | 0 | 0 | 0 | 1 |
| BEND5 | 2 | 0 | 0 | 0 | 0 | 0 | 0 | 0 | 1 |
| LRRN3 | 2 | 0 | 0 | 0 | 0 | 0 | 0 | 0 | 1 |
| KCNA2 | 2 | 0 | 0 | 0 | 0 | 0 | 0 | 0 | 1 |
| DMWD | 2 | 0 | 0 | 0 | 0 | 0 | 0 | 0 | 1 |
| DLGAP4 | 2 | 0 | 0 | 0 | 0 | 0 | 0 | 0 | 1 |
| ASB15 | 2 | 0 | 0 | 0 | 0 | 0 | 0 | 0 | 1 |
| HCK | 2 | 0 | 0 | 0 | 0 | 0 | 0 | 0 | 1 |
| CHST15 | 2 | 0 | 0 | 0 | 0 | 0 | 0 | 0 | 1 |
| MTA2 | 2 | 0 | 0 | 0 | 0 | 0 | 0 | 0 | 1 |
| MID1 | 2 | 0 | 0 | 0 | 0 | 0 | 0 | 0 | 1 |
| WDR65 | 2 | 0 | 0 | 0 | 0 | 0 | 0 | 0 | 1 |
| SMOC1 | 2 | 0 | 0 | 0 | 0 | 0 | 0 | 0 | 1 |
| FH | 2 | 0 | 0 | 0 | 0 | 0 | 0 | 0 | 1 |
| TBRG4 | 2 | 0 | 0 | 0 | 0 | 0 | 0 | 0 | 1 |
| PABPC4 | 2 | 0 | 0 | 0 | 0 | 0 | 0 | 0 | 1 |
| DHTKD1 | 2 | 0 | 0 | 0 | 0 | 0 | 0 | 0 | 1 |
| RGS21 | 2 | 0 | 0 | 0 | 0 | 0 | 0 | 0 | 1 |
| KIAA1715 | 2 | 0 | 0 | 0 | 0 | 0 | 0 | 0 | 1 |
| OR5A1 | 1 | 1 | 0 | 0 | 0 | 0 | 0 | 0 | 1 |
| TBC1D12 | 2 | 0 | 0 | 0 | 0 | 0 | 0 | 0 | 1 |
| KRT77 | 2 | 0 | 0 | 0 | 0 | 0 | 0 | 0 | 1 |
| MTMR1 | 2 | 0 | 0 | 0 | 0 | 0 | 0 | 0 | 1 |
| RACGAP1 | 2 | 0 | 0 | 0 | 0 | 0 | 0 | 0 | 1 |
| CDKAL1 | 2 | 0 | 0 | 0 | 0 | 0 | 0 | 0 | 1 |
| LRRC34 | 2 | 0 | 0 | 0 | 0 | 0 | 0 | 0 | 1 |
| OLFML1 | 2 | 0 | 0 | 0 | 0 | 0 | 0 | 0 | 1 |
| MYCL1 | 2 | 0 | 0 | 0 | 0 | 0 | 0 | 0 | 1 |
| PRKACG | 2 | 0 | 0 | 0 | 0 | 0 | 0 | 0 | 1 |
| ALS2CR8 | 2 | 0 | 0 | 0 | 0 | 0 | 0 | 0 | 1 |
| OR8D2 | 2 | 0 | 0 | 0 | 0 | 0 | 0 | 0 | 1 |
| ZNF438 | 1 | 1 | 0 | 0 | 0 | 0 | 0 | 0 | 1 |
| ERCC2 | 2 | 0 | 0 | 0 | 0 | 0 | 0 | 0 | 1 |
| ACVR1C | 2 | 0 | 0 | 0 | 0 | 0 | 0 | 0 | 1 |
| WWC1 | 1 | 1 | 0 | 0 | 0 | 0 | 0 | 0 | 1 |
| ATIC | 2 | 0 | 0 | 0 | 0 | 0 | 0 | 0 | 1 |
| ECM1 | 2 | 0 | 0 | 0 | 0 | 0 | 0 | 0 | 1 |
| C16orf7 | 2 | 0 | 0 | 0 | 0 | 0 | 0 | 0 | 1 |
| BCAM | 2 | 0 | 0 | 0 | 0 | 0 | 0 | 0 | 1 |
| ZNF233 | 1 | 0 | 0 | 1 | 0 | 0 | 0 | 0 | 1 |
| STRN4 | 2 | 0 | 0 | 0 | 0 | 0 | 0 | 0 | 1 |

| | | | | | | | | | |
|---|---|---|---|---|---|---|---|---|---|
| GBP7 | 2 | 0 | 0 | 0 | 0 | 0 | 0 | 0 | 1 |
| DNAI1 | 2 | 0 | 0 | 0 | 0 | 0 | 0 | 0 | 1 |
| C18orf8 | 2 | 0 | 0 | 0 | 0 | 0 | 0 | 0 | 1 |
| MX1 | 2 | 0 | 0 | 0 | 0 | 0 | 0 | 0 | 1 |
| RPN2 | 2 | 0 | 0 | 0 | 0 | 0 | 0 | 0 | 1 |
| B3GALT1 | 2 | 0 | 0 | 0 | 0 | 0 | 0 | 0 | 1 |
| FAM40B | 2 | 0 | 0 | 0 | 0 | 0 | 0 | 0 | 1 |
| ZNF764 | 2 | 0 | 0 | 0 | 0 | 0 | 0 | 0 | 1 |
| CCDC50 | 2 | 0 | 0 | 0 | 0 | 0 | 0 | 0 | 1 |
| OXA1L | 2 | 0 | 0 | 0 | 0 | 0 | 0 | 0 | 1 |
| C9 | 2 | 0 | 0 | 0 | 0 | 0 | 0 | 0 | 1 |
| BHMT2 | 2 | 0 | 0 | 0 | 0 | 0 | 0 | 0 | 1 |
| CASP5 | 2 | 0 | 0 | 0 | 0 | 0 | 0 | 0 | 1 |
| CHST4 | 2 | 0 | 0 | 0 | 0 | 0 | 0 | 0 | 1 |
| KBTBD4 | 2 | 0 | 0 | 0 | 0 | 0 | 0 | 0 | 1 |
| CNR2 | 2 | 0 | 0 | 0 | 0 | 0 | 0 | 0 | 1 |
| KIAA1958 | 2 | 0 | 0 | 0 | 0 | 0 | 0 | 0 | 1 |
| ZNF93 | 2 | 0 | 0 | 0 | 0 | 0 | 0 | 0 | 1 |
| WARS2 | 2 | 0 | 0 | 0 | 0 | 0 | 0 | 0 | 1 |
| GNS | 2 | 0 | 0 | 0 | 0 | 0 | 0 | 0 | 1 |
| ACOXL | 2 | 0 | 0 | 0 | 0 | 0 | 0 | 0 | 1 |
| PGM3 | 2 | 0 | 0 | 0 | 0 | 0 | 0 | 0 | 1 |
| MEIS1 | 2 | 0 | 0 | 0 | 0 | 0 | 0 | 0 | 1 |
| SESTD1 | 2 | 0 | 0 | 0 | 0 | 0 | 0 | 0 | 1 |
| ZNF880 | 2 | 0 | 0 | 0 | 0 | 0 | 0 | 0 | 1 |
| RECK | 2 | 0 | 0 | 0 | 0 | 0 | 0 | 0 | 1 |
| SKAP2 | 1 | 1 | 0 | 0 | 0 | 0 | 0 | 0 | 1 |
| PKLR | 2 | 0 | 0 | 0 | 0 | 0 | 0 | 0 | 1 |
| IL1R1 | 2 | 0 | 0 | 0 | 0 | 0 | 0 | 0 | 1 |
| ZNF365 | 1 | 1 | 0 | 0 | 0 | 0 | 0 | 0 | 1 |
| TXNRD1 | 2 | 0 | 0 | 0 | 0 | 0 | 0 | 0 | 1 |
| ITGAX | 1 | 1 | 0 | 0 | 0 | 0 | 0 | 0 | 1 |
| SNX27 | 2 | 0 | 0 | 0 | 0 | 0 | 0 | 0 | 1 |
| TBC1D15 | 2 | 0 | 0 | 0 | 0 | 0 | 0 | 0 | 1 |
| ACHE | 2 | 0 | 0 | 0 | 0 | 0 | 0 | 0 | 1 |
| APCDD1L | 2 | 0 | 0 | 0 | 0 | 0 | 0 | 0 | 1 |
| FANCA | 1 | 0 | 1 | 0 | 0 | 0 | 0 | 0 | 1 |
| DAPP1 | 2 | 0 | 0 | 0 | 0 | 0 | 0 | 0 | 1 |
| SENP5 | 2 | 0 | 0 | 0 | 0 | 0 | 0 | 0 | 1 |
| ITGB6 | 2 | 0 | 0 | 0 | 0 | 0 | 0 | 0 | 1 |
| TRMT61B | 2 | 0 | 0 | 0 | 0 | 0 | 0 | 0 | 1 |
| ZBED2 | 2 | 0 | 0 | 0 | 0 | 0 | 0 | 0 | 1 |
| MBTD1 | 2 | 0 | 0 | 0 | 0 | 0 | 0 | 0 | 1 |
| PTK7 | 2 | 0 | 0 | 0 | 0 | 0 | 0 | 0 | 1 |
| SNX9 | 2 | 0 | 0 | 0 | 0 | 0 | 0 | 0 | 1 |
| LRIG2 | 2 | 0 | 0 | 0 | 0 | 0 | 0 | 0 | 1 |

| | | | | | | | | | |
|---|---|---|---|---|---|---|---|---|---|
| KIAA0895 | 2 | 0 | 0 | 0 | 0 | 0 | 0 | 0 | 1 |
| DDX24 | 2 | 0 | 0 | 0 | 0 | 0 | 0 | 0 | 1 |
| ZNF639 | 2 | 0 | 0 | 0 | 0 | 0 | 0 | 0 | 1 |
| SLC22A1 | 2 | 0 | 0 | 0 | 0 | 0 | 0 | 0 | 1 |
| DCDC1 | 2 | 0 | 0 | 0 | 0 | 0 | 0 | 0 | 1 |
| POC5 | 2 | 0 | 0 | 0 | 0 | 0 | 0 | 0 | 1 |
| ZCCHC7 | 2 | 0 | 0 | 0 | 0 | 0 | 0 | 0 | 1 |
| IKZF1 | 2 | 0 | 0 | 0 | 0 | 0 | 0 | 0 | 1 |
| TOR2A | 2 | 0 | 0 | 0 | 0 | 0 | 0 | 0 | 1 |
| SCML1 | 2 | 0 | 0 | 0 | 0 | 0 | 0 | 0 | 1 |
| ZNF800 | 2 | 0 | 0 | 0 | 0 | 0 | 0 | 0 | 1 |
| SEMA3F | 2 | 0 | 0 | 0 | 0 | 0 | 0 | 0 | 1 |
| BTNL3 | 2 | 0 | 0 | 0 | 0 | 0 | 0 | 0 | 1 |
| PLEKHM3 | 1 | 1 | 0 | 0 | 0 | 0 | 0 | 0 | 1 |
| CCDC48 | 2 | 0 | 0 | 0 | 0 | 0 | 0 | 0 | 1 |
| ALDH3A1 | 2 | 0 | 0 | 0 | 0 | 0 | 0 | 0 | 1 |
| RASGRP1 | 2 | 0 | 0 | 0 | 0 | 0 | 0 | 0 | 1 |
| NOL9 | 2 | 0 | 0 | 0 | 0 | 0 | 0 | 0 | 1 |
| ASL | 2 | 0 | 0 | 0 | 0 | 0 | 0 | 0 | 1 |
| TMEM87B | 2 | 0 | 0 | 0 | 0 | 0 | 0 | 0 | 1 |
| SPATA18 | 2 | 0 | 0 | 0 | 0 | 0 | 0 | 0 | 1 |
| OR13G1 | 2 | 0 | 0 | 0 | 0 | 0 | 0 | 0 | 1 |
| KRT74 | 2 | 0 | 0 | 0 | 0 | 0 | 0 | 0 | 1 |
| ZNF567 | 2 | 0 | 0 | 0 | 0 | 0 | 0 | 0 | 1 |
| STAU2 | 2 | 0 | 0 | 0 | 0 | 0 | 0 | 0 | 1 |
| SPOPL | 2 | 0 | 0 | 0 | 0 | 0 | 0 | 0 | 1 |
| TBC1D10A | 2 | 0 | 0 | 0 | 0 | 0 | 0 | 0 | 1 |
| RGAG4 | 2 | 0 | 0 | 0 | 0 | 0 | 0 | 0 | 1 |
| NPAS1 | 2 | 0 | 0 | 0 | 0 | 0 | 0 | 0 | 1 |
| AIM1L | 2 | 0 | 0 | 0 | 0 | 0 | 0 | 0 | 1 |
| PLCG1 | 1 | 1 | 0 | 0 | 0 | 0 | 0 | 0 | 1 |
| DCAF5 | 2 | 0 | 0 | 0 | 0 | 0 | 0 | 0 | 1 |
| RAD54L | 1 | 1 | 0 | 0 | 0 | 0 | 0 | 0 | 1 |
| FAM71A | 2 | 0 | 0 | 0 | 0 | 0 | 0 | 0 | 1 |
| FOXP4 | 2 | 0 | 0 | 0 | 0 | 0 | 0 | 0 | 1 |
| FAM113A | 2 | 0 | 0 | 0 | 0 | 0 | 0 | 0 | 1 |
| KLRG1 | 2 | 0 | 0 | 0 | 0 | 0 | 0 | 0 | 1 |
| SHB | 2 | 0 | 0 | 0 | 0 | 0 | 0 | 0 | 1 |
| TNFRSF21 | 2 | 0 | 0 | 0 | 0 | 0 | 0 | 0 | 1 |
| HDC | 2 | 0 | 0 | 0 | 0 | 0 | 0 | 0 | 1 |
| SYT11 | 2 | 0 | 0 | 0 | 0 | 0 | 0 | 0 | 1 |
| DLL3 | 2 | 0 | 0 | 0 | 0 | 0 | 0 | 0 | 1 |
| TMCO4 | 2 | 0 | 0 | 0 | 0 | 0 | 0 | 0 | 1 |
| IWS1 | 2 | 0 | 0 | 0 | 0 | 0 | 0 | 0 | 1 |
| GLCCI1 | 2 | 0 | 0 | 0 | 0 | 0 | 0 | 0 | 1 |
| MIER2 | 2 | 0 | 0 | 0 | 0 | 0 | 0 | 0 | 1 |

| | | | | | | | | | |
|---|---|---|---|---|---|---|---|---|---|
| ZNF777 | 2 | 0 | 0 | 0 | 0 | 0 | 0 | 0 | 1 |
| CSDA | 2 | 0 | 0 | 0 | 0 | 0 | 0 | 0 | 1 |
| PIWIL4 | 2 | 0 | 0 | 0 | 0 | 0 | 0 | 0 | 1 |
| DMKN | 2 | 0 | 0 | 0 | 0 | 0 | 0 | 0 | 1 |
| STXBP2 | 2 | 0 | 0 | 0 | 0 | 0 | 0 | 0 | 1 |
| MTHFR | 2 | 0 | 0 | 0 | 0 | 0 | 0 | 0 | 1 |
| DGCR14 | 2 | 0 | 0 | 0 | 0 | 0 | 0 | 0 | 1 |
| MPL | 2 | 0 | 0 | 0 | 0 | 0 | 0 | 0 | 1 |
| PGM5 | 2 | 0 | 0 | 0 | 0 | 0 | 0 | 0 | 1 |
| MFN2 | 2 | 0 | 0 | 0 | 0 | 0 | 0 | 0 | 1 |
| LEPREL2 | 2 | 0 | 0 | 0 | 0 | 0 | 0 | 0 | 1 |
| C2orf42 | 2 | 0 | 0 | 0 | 0 | 0 | 0 | 0 | 1 |
| CPNE1 | 2 | 0 | 0 | 0 | 0 | 0 | 0 | 0 | 1 |
| CCDC91 | 2 | 0 | 0 | 0 | 0 | 0 | 0 | 0 | 1 |
| DENND4C | 1 | 1 | 0 | 0 | 0 | 0 | 0 | 0 | 1 |
| ASCC2 | 2 | 0 | 0 | 0 | 0 | 0 | 0 | 0 | 1 |
| STK38L | 2 | 0 | 0 | 0 | 0 | 0 | 0 | 0 | 1 |
| IRAK2 | 2 | 0 | 0 | 0 | 0 | 0 | 0 | 0 | 1 |
| LEO1 | 2 | 0 | 0 | 0 | 0 | 0 | 0 | 0 | 1 |
| DDX59 | 2 | 0 | 0 | 0 | 0 | 0 | 0 | 0 | 1 |
| C14orf49 | 2 | 0 | 0 | 0 | 0 | 0 | 0 | 0 | 1 |
| CNGA2 | 2 | 0 | 0 | 0 | 0 | 0 | 0 | 0 | 1 |
| GALNT1 | 2 | 0 | 0 | 0 | 0 | 0 | 0 | 0 | 1 |
| USP37 | 2 | 0 | 0 | 0 | 0 | 0 | 0 | 0 | 1 |
| ZNF223 | 1 | 1 | 0 | 0 | 0 | 0 | 0 | 0 | 1 |
| FAM129C | 2 | 0 | 0 | 0 | 0 | 0 | 0 | 0 | 1 |
| IGF2BP2 | 2 | 0 | 0 | 0 | 0 | 0 | 0 | 0 | 1 |
| TLR1 | 2 | 0 | 0 | 0 | 0 | 0 | 0 | 0 | 1 |
| JPH4 | 2 | 0 | 0 | 0 | 0 | 0 | 0 | 0 | 1 |
| PYROXD2 | 2 | 0 | 0 | 0 | 0 | 0 | 0 | 0 | 1 |
| STAT3 | 2 | 0 | 0 | 0 | 0 | 0 | 0 | 0 | 1 |
| LZTR1 | 2 | 0 | 0 | 0 | 0 | 0 | 0 | 0 | 1 |
| KRT15 | 2 | 0 | 0 | 0 | 0 | 0 | 0 | 0 | 1 |
| HOXD3 | 2 | 0 | 0 | 0 | 0 | 0 | 0 | 0 | 1 |
| MAP3K11 | 2 | 0 | 0 | 0 | 0 | 0 | 0 | 0 | 1 |
| RBM28 | 2 | 0 | 0 | 0 | 0 | 0 | 0 | 0 | 1 |
| HMHA1 | 2 | 0 | 0 | 0 | 0 | 0 | 0 | 0 | 1 |
| GABRA3 | 2 | 0 | 0 | 0 | 0 | 0 | 0 | 0 | 1 |
| PRKAA2 | 2 | 0 | 0 | 0 | 0 | 0 | 0 | 0 | 1 |
| ZNF512B | 2 | 0 | 0 | 0 | 0 | 0 | 0 | 0 | 1 |
| MMD2 | 1 | 1 | 0 | 0 | 0 | 0 | 0 | 0 | 1 |
| CCDC81 | 2 | 0 | 0 | 0 | 0 | 0 | 0 | 0 | 1 |
| TMEM232 | 2 | 0 | 0 | 0 | 0 | 0 | 0 | 0 | 1 |
| ZNF197 | 2 | 0 | 0 | 0 | 0 | 0 | 0 | 0 | 1 |
| MCM5 | 2 | 0 | 0 | 0 | 0 | 0 | 0 | 0 | 1 |
| SYT14 | 2 | 0 | 0 | 0 | 0 | 0 | 0 | 0 | 1 |

| | | | | | | | | | |
|---|---|---|---|---|---|---|---|---|---|
| CNGA3 | 2 | 0 | 0 | 0 | 0 | 0 | 0 | 0 | 1 |
| ZNF512 | 2 | 0 | 0 | 0 | 0 | 0 | 0 | 0 | 1 |
| SRPK2 | 2 | 0 | 0 | 0 | 0 | 0 | 0 | 0 | 1 |
| RAI14 | 1 | 0 | 0 | 0 | 1 | 0 | 0 | 0 | 1 |
| DAG1 | 2 | 0 | 0 | 0 | 0 | 0 | 0 | 0 | 1 |
| FCRLA | 2 | 0 | 0 | 0 | 0 | 0 | 0 | 0 | 1 |
| PPP1R15A | 2 | 0 | 0 | 0 | 0 | 0 | 0 | 0 | 1 |
| EPHB3 | 2 | 0 | 0 | 0 | 0 | 0 | 0 | 0 | 1 |
| MAK | 2 | 0 | 0 | 0 | 0 | 0 | 0 | 0 | 1 |
| YTHDC1 | 2 | 0 | 0 | 0 | 0 | 0 | 0 | 0 | 1 |
| MANBA | 2 | 0 | 0 | 0 | 0 | 0 | 0 | 0 | 1 |
| ZNF846 | 2 | 0 | 0 | 0 | 0 | 0 | 0 | 0 | 1 |
| CYP4V2 | 2 | 0 | 0 | 0 | 0 | 0 | 0 | 0 | 1 |
| CD1B | 2 | 0 | 0 | 0 | 0 | 0 | 0 | 0 | 1 |
| ADCY3 | 2 | 0 | 0 | 0 | 0 | 0 | 0 | 0 | 1 |
| ZNF90 | 2 | 0 | 0 | 0 | 0 | 0 | 0 | 0 | 1 |
| ZNF48 | 2 | 0 | 0 | 0 | 0 | 0 | 0 | 0 | 1 |
| RANBP3 | 2 | 0 | 0 | 0 | 0 | 0 | 0 | 0 | 1 |
| WDR76 | 2 | 0 | 0 | 0 | 0 | 0 | 0 | 0 | 1 |
| SLC41A3 | 2 | 0 | 0 | 0 | 0 | 0 | 0 | 0 | 1 |
| MMP9 | 2 | 0 | 0 | 0 | 0 | 0 | 0 | 0 | 1 |
| GLB1L3 | 2 | 0 | 0 | 0 | 0 | 0 | 0 | 0 | 1 |
| WDR60 | 1 | 0 | 0 | 0 | 1 | 0 | 0 | 0 | 1 |
| ZNF805 | 2 | 0 | 0 | 0 | 0 | 0 | 0 | 0 | 1 |
| RAP1GDS1 | 2 | 0 | 0 | 0 | 0 | 0 | 0 | 0 | 1 |
| OR8A1 | 2 | 0 | 0 | 0 | 0 | 0 | 0 | 0 | 1 |
| ZNF558 | 2 | 0 | 0 | 0 | 0 | 0 | 0 | 0 | 1 |
| NBPF7 | 2 | 0 | 0 | 0 | 0 | 0 | 0 | 0 | 1 |
| TEX13A | 2 | 0 | 0 | 0 | 0 | 0 | 0 | 0 | 1 |
| FAM55D | 2 | 0 | 0 | 0 | 0 | 0 | 0 | 0 | 1 |
| SLC25A46 | 2 | 0 | 0 | 0 | 0 | 0 | 0 | 0 | 1 |
| ZC3H7B | 1 | 0 | 0 | 0 | 1 | 0 | 0 | 0 | 1 |
| EMR3 | 2 | 0 | 0 | 0 | 0 | 0 | 0 | 0 | 1 |
| RAP1GAP2 | 2 | 0 | 0 | 0 | 0 | 0 | 0 | 0 | 1 |
| ZSCAN1 | 2 | 0 | 0 | 0 | 0 | 0 | 0 | 0 | 1 |
| LSS | 2 | 0 | 0 | 0 | 0 | 0 | 0 | 0 | 1 |
| HHIPL1 | 2 | 0 | 0 | 0 | 0 | 0 | 0 | 0 | 1 |
| TGM1 | 2 | 0 | 0 | 0 | 0 | 0 | 0 | 0 | 1 |
| PHF21B | 1 | 0 | 0 | 0 | 1 | 0 | 0 | 0 | 1 |
| NR4A1 | 2 | 0 | 0 | 0 | 0 | 0 | 0 | 0 | 1 |
| KDM4B | 1 | 1 | 0 | 0 | 0 | 0 | 0 | 0 | 1 |
| PTPN5 | 1 | 1 | 0 | 0 | 0 | 0 | 0 | 0 | 1 |
| MAGEE2 | 2 | 0 | 0 | 0 | 0 | 0 | 0 | 0 | 1 |
| TCF3 | 2 | 0 | 0 | 0 | 0 | 0 | 0 | 0 | 1 |
| KCTD19 | 2 | 0 | 0 | 0 | 0 | 0 | 0 | 0 | 1 |
| VEZT | 2 | 0 | 0 | 0 | 0 | 0 | 0 | 0 | 1 |

| | | | | | | | | | |
|---|---|---|---|---|---|---|---|---|---|
| MAS1L | 2 | 0 | 0 | 0 | 0 | 0 | 0 | 0 | 1 |
| IRX2 | 2 | 0 | 0 | 0 | 0 | 0 | 0 | 0 | 1 |
| CTNND1 | 2 | 0 | 0 | 0 | 0 | 0 | 0 | 0 | 1 |
| FBXL5 | 2 | 0 | 0 | 0 | 0 | 0 | 0 | 0 | 1 |
| MYLK2 | 2 | 0 | 0 | 0 | 0 | 0 | 0 | 0 | 1 |
| RG9MTD2 | 2 | 0 | 0 | 0 | 0 | 0 | 0 | 0 | 1 |
| UNC13D | 2 | 0 | 0 | 0 | 0 | 0 | 0 | 0 | 1 |
| NKD1 | 2 | 0 | 0 | 0 | 0 | 0 | 0 | 0 | 1 |
| RIPK1 | 2 | 0 | 0 | 0 | 0 | 0 | 0 | 0 | 1 |
| CYP4F3 | 2 | 0 | 0 | 0 | 0 | 0 | 0 | 0 | 1 |
| PTPN12 | 2 | 0 | 0 | 0 | 0 | 0 | 0 | 0 | 1 |
| SERPINI2 | 2 | 0 | 0 | 0 | 0 | 0 | 0 | 0 | 1 |
| SLC26A7 | 2 | 0 | 0 | 0 | 0 | 0 | 0 | 0 | 1 |
| WASF1 | 2 | 0 | 0 | 0 | 0 | 0 | 0 | 0 | 1 |
| EIF2C3 | 2 | 0 | 0 | 0 | 0 | 0 | 0 | 0 | 1 |
| RCOR3 | 2 | 0 | 0 | 0 | 0 | 0 | 0 | 0 | 1 |
| CARD8 | 2 | 0 | 0 | 0 | 0 | 0 | 0 | 0 | 1 |
| FAM170A | 2 | 0 | 0 | 0 | 0 | 0 | 0 | 0 | 1 |
| NAPRT1 | 2 | 0 | 0 | 0 | 0 | 0 | 0 | 0 | 1 |
| NHLRC2 | 2 | 0 | 0 | 0 | 0 | 0 | 0 | 0 | 1 |
| RNF150 | 2 | 0 | 0 | 0 | 0 | 0 | 0 | 0 | 1 |
| SVOPL | 2 | 0 | 0 | 0 | 0 | 0 | 0 | 0 | 1 |
| POMGNT1 | 2 | 0 | 0 | 0 | 0 | 0 | 0 | 0 | 1 |
| ATP4B | 1 | 1 | 0 | 0 | 0 | 0 | 0 | 0 | 1 |
| FAM118A | 2 | 0 | 0 | 0 | 0 | 0 | 0 | 0 | 1 |
| C16orf88 | 2 | 0 | 0 | 0 | 0 | 0 | 0 | 0 | 1 |
| ZNF598 | 2 | 0 | 0 | 0 | 0 | 0 | 0 | 0 | 1 |
| FAM169A | 2 | 0 | 0 | 0 | 0 | 0 | 0 | 0 | 1 |
| CDH22 | 2 | 0 | 0 | 0 | 0 | 0 | 0 | 0 | 1 |
| TMEM26 | 2 | 0 | 0 | 0 | 0 | 0 | 0 | 0 | 1 |
| EIF2C4 | 2 | 0 | 0 | 0 | 0 | 0 | 0 | 0 | 1 |
| COX7B2 | 1 | 1 | 0 | 0 | 0 | 0 | 0 | 0 | 1 |
| DRG2 | 2 | 0 | 0 | 0 | 0 | 0 | 0 | 0 | 1 |
| EIF2C2 | 2 | 0 | 0 | 0 | 0 | 0 | 0 | 0 | 1 |
| SYT3 | 2 | 0 | 0 | 0 | 0 | 0 | 0 | 0 | 1 |
| WSCD1 | 2 | 0 | 0 | 0 | 0 | 0 | 0 | 0 | 1 |
| FAM189B | 2 | 0 | 0 | 0 | 0 | 0 | 0 | 0 | 1 |
| GRIK5 | 1 | 1 | 0 | 0 | 0 | 0 | 0 | 0 | 1 |
| PPARGC1A | 2 | 0 | 0 | 0 | 0 | 0 | 0 | 0 | 1 |
| OSMR | 2 | 0 | 0 | 0 | 0 | 0 | 0 | 0 | 1 |
| KIAA0317 | 2 | 0 | 0 | 0 | 0 | 0 | 0 | 0 | 1 |
| PTH2R | 2 | 0 | 0 | 0 | 0 | 0 | 0 | 0 | 1 |
| PNPT1 | 2 | 0 | 0 | 0 | 0 | 0 | 0 | 0 | 1 |
| FAM117A | 2 | 0 | 0 | 0 | 0 | 0 | 0 | 0 | 1 |
| TRPV6 | 2 | 0 | 0 | 0 | 0 | 0 | 0 | 0 | 1 |
| ADAM9 | 2 | 0 | 0 | 0 | 0 | 0 | 0 | 0 | 1 |

| | | | | | | | | | |
|---|---|---|---|---|---|---|---|---|---|
| VPS11 | 2 | 0 | 0 | 0 | 0 | 0 | 0 | 0 | 1 |
| C17orf53 | 2 | 0 | 0 | 0 | 0 | 0 | 0 | 0 | 1 |
| ELL2 | 2 | 0 | 0 | 0 | 0 | 0 | 0 | 0 | 1 |
| CDC42BPG | 1 | 1 | 0 | 0 | 0 | 0 | 0 | 0 | 1 |
| SLC17A2 | 2 | 0 | 0 | 0 | 0 | 0 | 0 | 0 | 1 |
| NLN | 2 | 0 | 0 | 0 | 0 | 0 | 0 | 0 | 1 |
| TUBGCP4 | 2 | 0 | 0 | 0 | 0 | 0 | 0 | 0 | 1 |
| SMYD4 | 2 | 0 | 0 | 0 | 0 | 0 | 0 | 0 | 1 |
| GGCX | 2 | 0 | 0 | 0 | 0 | 0 | 0 | 0 | 1 |
| ATP6V1B1 | 2 | 0 | 0 | 0 | 0 | 0 | 0 | 0 | 1 |
| RAB11FIP3 | 2 | 0 | 0 | 0 | 0 | 0 | 0 | 0 | 1 |
| SETDB2 | 1 | 1 | 0 | 0 | 0 | 0 | 0 | 0 | 1 |
| ZNF527 | 2 | 0 | 0 | 0 | 0 | 0 | 0 | 0 | 1 |
| HK3 | 2 | 0 | 0 | 0 | 0 | 0 | 0 | 0 | 1 |
| OPHN1 | 2 | 0 | 0 | 0 | 0 | 0 | 0 | 0 | 1 |
| UBE3C | 2 | 0 | 0 | 0 | 0 | 0 | 0 | 0 | 1 |
| WDR66 | 2 | 0 | 0 | 0 | 0 | 0 | 0 | 0 | 1 |
| ENPP6 | 2 | 0 | 0 | 0 | 0 | 0 | 0 | 0 | 1 |
| VPS16 | 2 | 0 | 0 | 0 | 0 | 0 | 0 | 0 | 1 |
| SLC44A2 | 2 | 0 | 0 | 0 | 0 | 0 | 0 | 0 | 1 |
| SPRED1 | 2 | 0 | 0 | 0 | 0 | 0 | 0 | 0 | 1 |
| SLC26A11 | 2 | 0 | 0 | 0 | 0 | 0 | 0 | 0 | 1 |
| ZNF19 | 2 | 0 | 0 | 0 | 0 | 0 | 0 | 0 | 1 |
| MCHR2 | 1 | 1 | 0 | 0 | 0 | 0 | 0 | 0 | 1 |
| ATP6V0A1 | 2 | 0 | 0 | 0 | 0 | 0 | 0 | 0 | 1 |
| PLA2G4A | 2 | 0 | 0 | 0 | 0 | 0 | 0 | 0 | 1 |
| TOP3B | 2 | 0 | 0 | 0 | 0 | 0 | 0 | 0 | 1 |
| NBR1 | 2 | 0 | 0 | 0 | 0 | 0 | 0 | 0 | 1 |
| ZFP90 | 2 | 0 | 0 | 0 | 0 | 0 | 0 | 0 | 1 |
| SECISBP2L | 2 | 0 | 0 | 0 | 0 | 0 | 0 | 0 | 1 |
| SYN3 | 2 | 0 | 0 | 0 | 0 | 0 | 0 | 0 | 1 |
| BANK1 | 2 | 0 | 0 | 0 | 0 | 0 | 0 | 0 | 1 |
| NUP107 | 2 | 0 | 0 | 0 | 0 | 0 | 0 | 0 | 1 |
| ERCC3 | 2 | 0 | 0 | 0 | 0 | 0 | 0 | 0 | 1 |
| BTK | 2 | 0 | 0 | 0 | 0 | 0 | 0 | 0 | 1 |
| CCDC116 | 2 | 0 | 0 | 0 | 0 | 0 | 0 | 0 | 1 |
| SIX4 | 2 | 0 | 0 | 0 | 0 | 0 | 0 | 0 | 1 |
| SHC2 | 2 | 0 | 0 | 0 | 0 | 0 | 0 | 0 | 1 |
| GLRA2 | 2 | 0 | 0 | 0 | 0 | 0 | 0 | 0 | 1 |
| C1orf87 | 2 | 0 | 0 | 0 | 0 | 0 | 0 | 0 | 1 |
| LILRB3 | 2 | 0 | 0 | 0 | 0 | 0 | 0 | 0 | 1 |
| SLC6A5 | 1 | 0 | 1 | 0 | 0 | 0 | 0 | 0 | 1 |
| FXR1 | 2 | 0 | 0 | 0 | 0 | 0 | 0 | 0 | 1 |
| SP100 | 0 | 2 | 0 | 0 | 0 | 0 | 0 | 0 | 1 |
| TPX2 | 2 | 0 | 0 | 0 | 0 | 0 | 0 | 0 | 1 |
| PGC | 2 | 0 | 0 | 0 | 0 | 0 | 0 | 0 | 1 |

| | | | | | | | | | |
|---|---|---|---|---|---|---|---|---|---|
| EIF5B | 1 | 1 | 0 | 0 | 0 | 0 | 0 | 0 | 1 |
| C12orf12 | 2 | 0 | 0 | 0 | 0 | 0 | 0 | 0 | 1 |
| ADCY1 | 1 | 1 | 0 | 0 | 0 | 0 | 0 | 0 | 1 |
| GOLGA1 | 2 | 0 | 0 | 0 | 0 | 0 | 0 | 0 | 1 |
| SLC35F3 | 2 | 0 | 0 | 0 | 0 | 0 | 0 | 0 | 1 |
| DCBLD2 | 2 | 0 | 0 | 0 | 0 | 0 | 0 | 0 | 1 |
| EGFR | 1 | 1 | 0 | 0 | 0 | 0 | 0 | 0 | 1 |
| SMG6 | 1 | 1 | 0 | 0 | 0 | 0 | 0 | 0 | 1 |
| HPS1 | 2 | 0 | 0 | 0 | 0 | 0 | 0 | 0 | 1 |
| DDX31 | 1 | 1 | 0 | 0 | 0 | 0 | 0 | 0 | 1 |
| MAP7D2 | 2 | 0 | 0 | 0 | 0 | 0 | 0 | 0 | 1 |
| C9orf43 | 2 | 0 | 0 | 0 | 0 | 0 | 0 | 0 | 1 |
| KBTBD3 | 2 | 0 | 0 | 0 | 0 | 0 | 0 | 0 | 1 |
| HGFAC | 2 | 0 | 0 | 0 | 0 | 0 | 0 | 0 | 1 |
| ZBTB47 | 2 | 0 | 0 | 0 | 0 | 0 | 0 | 0 | 1 |
| ARHGEF18 | 2 | 0 | 0 | 0 | 0 | 0 | 0 | 0 | 1 |
| INTS3 | 2 | 0 | 0 | 0 | 0 | 0 | 0 | 0 | 1 |
| CA12 | 2 | 0 | 0 | 0 | 0 | 0 | 0 | 0 | 1 |
| ADCY7 | 2 | 0 | 0 | 0 | 0 | 0 | 0 | 0 | 1 |
| ELFN2 | 2 | 0 | 0 | 0 | 0 | 0 | 0 | 0 | 1 |
| WDR25 | 2 | 0 | 0 | 0 | 0 | 0 | 0 | 0 | 1 |
| PAPD5 | 2 | 0 | 0 | 0 | 0 | 0 | 0 | 0 | 1 |
| FGD1 | 2 | 0 | 0 | 0 | 0 | 0 | 0 | 0 | 1 |
| KIAA0090 | 2 | 0 | 0 | 0 | 0 | 0 | 0 | 0 | 1 |
| DNAJC10 | 2 | 0 | 0 | 0 | 0 | 0 | 0 | 0 | 1 |
| LMBRD1 | 2 | 0 | 0 | 0 | 0 | 0 | 0 | 0 | 1 |
| FRMD3 | 2 | 0 | 0 | 0 | 0 | 0 | 0 | 0 | 1 |
| GSG2 | 2 | 0 | 0 | 0 | 0 | 0 | 0 | 0 | 1 |
| PDZD7 | 2 | 0 | 0 | 0 | 0 | 0 | 0 | 0 | 1 |
| KLHL33 | 2 | 0 | 0 | 0 | 0 | 0 | 0 | 0 | 1 |
| KIF1C | 2 | 0 | 0 | 0 | 0 | 0 | 0 | 0 | 1 |
| NUDCD1 | 2 | 0 | 0 | 0 | 0 | 0 | 0 | 0 | 1 |
| TBKBP1 | 2 | 0 | 0 | 0 | 0 | 0 | 0 | 0 | 1 |
| PRMT10 | 2 | 0 | 0 | 0 | 0 | 0 | 0 | 0 | 1 |
| TP63 | 2 | 0 | 0 | 0 | 0 | 0 | 0 | 0 | 1 |
| ZFP42 | 2 | 0 | 0 | 0 | 0 | 0 | 0 | 0 | 1 |
| PYGM | 2 | 0 | 0 | 0 | 0 | 0 | 0 | 0 | 1 |
| USP51 | 2 | 0 | 0 | 0 | 0 | 0 | 0 | 0 | 1 |
| B4GALNT2 | 1 | 1 | 0 | 0 | 0 | 0 | 0 | 0 | 1 |
| ZNF697 | 2 | 0 | 0 | 0 | 0 | 0 | 0 | 0 | 1 |
| DYTN | 2 | 0 | 0 | 0 | 0 | 0 | 0 | 0 | 1 |
| NTRK2 | 2 | 0 | 0 | 0 | 0 | 0 | 0 | 0 | 1 |
| GRHL3 | 2 | 0 | 0 | 0 | 0 | 0 | 0 | 0 | 1 |
| ZNF559 | 2 | 0 | 0 | 0 | 0 | 0 | 0 | 0 | 1 |
| SLC22A16 | 2 | 0 | 0 | 0 | 0 | 0 | 0 | 0 | 1 |
| MTMR4 | 2 | 0 | 0 | 0 | 0 | 0 | 0 | 0 | 1 |

| | | | | | | | | | |
|---|---|---|---|---|---|---|---|---|---|
| CASD1 | 2 | 0 | 0 | 0 | 0 | 0 | 0 | 0 | 1 |
| MPP2 | 2 | 0 | 0 | 0 | 0 | 0 | 0 | 0 | 1 |
| ST8SIA2 | 2 | 0 | 0 | 0 | 0 | 0 | 0 | 0 | 1 |
| NPAS2 | 2 | 0 | 0 | 0 | 0 | 0 | 0 | 0 | 1 |
| RXFP3 | 2 | 0 | 0 | 0 | 0 | 0 | 0 | 0 | 1 |
| FMO2 | 1 | 1 | 0 | 0 | 0 | 0 | 0 | 0 | 1 |
| RSPH3 | 2 | 0 | 0 | 0 | 0 | 0 | 0 | 0 | 1 |
| MAP6 | 2 | 0 | 0 | 0 | 0 | 0 | 0 | 0 | 1 |
| ZNF460 | 2 | 0 | 0 | 0 | 0 | 0 | 0 | 0 | 1 |
| API5 | 2 | 0 | 0 | 0 | 0 | 0 | 0 | 0 | 1 |
| PCDHB3 | 2 | 0 | 0 | 0 | 0 | 0 | 0 | 0 | 1 |
| FNBP1L | 2 | 0 | 0 | 0 | 0 | 0 | 0 | 0 | 1 |
| ARHGAP24 | 2 | 0 | 0 | 0 | 0 | 0 | 0 | 0 | 1 |
| IRF2 | 2 | 0 | 0 | 0 | 0 | 0 | 0 | 0 | 1 |
| DGKA | 2 | 0 | 0 | 0 | 0 | 0 | 0 | 0 | 1 |
| PRKCB | 2 | 0 | 0 | 0 | 0 | 0 | 0 | 0 | 1 |
| C10orf76 | 2 | 0 | 0 | 0 | 0 | 0 | 0 | 0 | 1 |
| SLC2A10 | 2 | 0 | 0 | 0 | 0 | 0 | 0 | 0 | 1 |
| MPEG1 | 2 | 0 | 0 | 0 | 0 | 0 | 0 | 0 | 1 |
| ZHX1 | 1 | 1 | 0 | 0 | 0 | 0 | 0 | 0 | 1 |
| GTF3C3 | 2 | 0 | 0 | 0 | 0 | 0 | 0 | 0 | 1 |
| PELI2 | 2 | 0 | 0 | 0 | 0 | 0 | 0 | 0 | 1 |
| ALCAM | 2 | 0 | 0 | 0 | 0 | 0 | 0 | 0 | 1 |
| ZCCHC14 | 2 | 0 | 0 | 0 | 0 | 0 | 0 | 0 | 1 |
| OR13C3 | 2 | 0 | 0 | 0 | 0 | 0 | 0 | 0 | 1 |
| FSCN3 | 2 | 0 | 0 | 0 | 0 | 0 | 0 | 0 | 1 |
| SDCCAG8 | 2 | 0 | 0 | 0 | 0 | 0 | 0 | 0 | 1 |
| ZC3HAV1 | 2 | 0 | 0 | 0 | 0 | 0 | 0 | 0 | 1 |
| SLC12A8 | 2 | 0 | 0 | 0 | 0 | 0 | 0 | 0 | 1 |
| IGSF5 | 2 | 0 | 0 | 0 | 0 | 0 | 0 | 0 | 1 |
| RALGDS | 2 | 0 | 0 | 0 | 0 | 0 | 0 | 0 | 1 |
| C7orf58 | 2 | 0 | 0 | 0 | 0 | 0 | 0 | 0 | 1 |
| SPERT | 2 | 0 | 0 | 0 | 0 | 0 | 0 | 0 | 1 |
| DCLRE1A | 2 | 0 | 0 | 0 | 0 | 0 | 0 | 0 | 1 |
| NSUN2 | 2 | 0 | 0 | 0 | 0 | 0 | 0 | 0 | 1 |
| SLC45A2 | 2 | 0 | 0 | 0 | 0 | 0 | 0 | 0 | 1 |
| TMEM63C | 2 | 0 | 0 | 0 | 0 | 0 | 0 | 0 | 1 |
| RFC1 | 2 | 0 | 0 | 0 | 0 | 0 | 0 | 0 | 1 |
| URGCP | 1 | 1 | 0 | 0 | 0 | 0 | 0 | 0 | 1 |
| RBM27 | 2 | 0 | 0 | 0 | 0 | 0 | 0 | 0 | 1 |
| TREM1 | 2 | 0 | 0 | 0 | 0 | 0 | 0 | 0 | 1 |
| SLC38A5 | 2 | 0 | 0 | 0 | 0 | 0 | 0 | 0 | 1 |
| BTBD7 | 2 | 0 | 0 | 0 | 0 | 0 | 0 | 0 | 1 |
| SLC9A9 | 2 | 0 | 0 | 0 | 0 | 0 | 0 | 0 | 1 |
| KCNAB1 | 2 | 0 | 0 | 0 | 0 | 0 | 0 | 0 | 1 |
| HSP90B1 | 2 | 0 | 0 | 0 | 0 | 0 | 0 | 0 | 1 |

| | | | | | | | | | |
|---|---|---|---|---|---|---|---|---|---|
| GBE1 | 2 | 0 | 0 | 0 | 0 | 0 | 0 | 0 | 1 |
| SLC5A9 | 2 | 0 | 0 | 0 | 0 | 0 | 0 | 0 | 1 |
| LRRC8D | 2 | 0 | 0 | 0 | 0 | 0 | 0 | 0 | 1 |
| DGKQ | 2 | 0 | 0 | 0 | 0 | 0 | 0 | 0 | 1 |
| CCDC15 | 1 | 1 | 0 | 0 | 0 | 0 | 0 | 0 | 1 |
| IQSEC1 | 1 | 0 | 1 | 0 | 0 | 0 | 0 | 0 | 1 |
| CDC14A | 2 | 0 | 0 | 0 | 0 | 0 | 0 | 0 | 1 |
| SLC12A3 | 2 | 0 | 0 | 0 | 0 | 0 | 0 | 0 | 1 |
| HSD17B4 | 2 | 0 | 0 | 0 | 0 | 0 | 0 | 0 | 1 |
| MKL1 | 2 | 0 | 0 | 0 | 0 | 0 | 0 | 0 | 1 |
| HPS4 | 2 | 0 | 0 | 0 | 0 | 0 | 0 | 0 | 1 |
| CBLL1 | 1 | 1 | 0 | 0 | 0 | 0 | 0 | 0 | 1 |
| FAM71B | 2 | 0 | 0 | 0 | 0 | 0 | 0 | 0 | 1 |
| SF3B1 | 2 | 0 | 0 | 0 | 0 | 0 | 0 | 0 | 1 |
| MAP3K14 | 1 | 0 | 0 | 0 | 1 | 0 | 0 | 0 | 1 |
| PTGS2 | 2 | 0 | 0 | 0 | 0 | 0 | 0 | 0 | 1 |
| SH3RF2 | 2 | 0 | 0 | 0 | 0 | 0 | 0 | 0 | 1 |
| PRDM4 | 2 | 0 | 0 | 0 | 0 | 0 | 0 | 0 | 1 |
| OR5D18 | 2 | 0 | 0 | 0 | 0 | 0 | 0 | 0 | 1 |
| CCDC17 | 2 | 0 | 0 | 0 | 0 | 0 | 0 | 0 | 1 |
| ARHGEF33 | 2 | 0 | 0 | 0 | 0 | 0 | 0 | 0 | 1 |
| ANKRD53 | 2 | 0 | 0 | 0 | 0 | 0 | 0 | 0 | 1 |
| CPT1B | 2 | 0 | 0 | 0 | 0 | 0 | 0 | 0 | 1 |
| SORBS3 | 2 | 0 | 0 | 0 | 0 | 0 | 0 | 0 | 1 |
| ZNF257 | 2 | 0 | 0 | 0 | 0 | 0 | 0 | 0 | 1 |
| FBXO10 | 2 | 0 | 0 | 0 | 0 | 0 | 0 | 0 | 1 |
| RBM46 | 2 | 0 | 0 | 0 | 0 | 0 | 0 | 0 | 1 |
| COMP | 2 | 0 | 0 | 0 | 0 | 0 | 0 | 0 | 1 |
| PGBD1 | 1 | 1 | 0 | 0 | 0 | 0 | 0 | 0 | 1 |
| SERPINA9 | 2 | 0 | 0 | 0 | 0 | 0 | 0 | 0 | 1 |
| MLXIPL | 2 | 0 | 0 | 0 | 0 | 0 | 0 | 0 | 1 |
| NCOA1 | 2 | 0 | 0 | 0 | 0 | 0 | 0 | 0 | 1 |
| SRRM4 | 2 | 0 | 0 | 0 | 0 | 0 | 0 | 0 | 1 |
| RB1CC1 | 1 | 0 | 0 | 0 | 0 | 1 | 0 | 0 | 1 |
| LCORL | 2 | 0 | 0 | 0 | 0 | 0 | 0 | 0 | 1 |
| LGR5 | 2 | 0 | 0 | 0 | 0 | 0 | 0 | 0 | 1 |
| PKN1 | 2 | 0 | 0 | 0 | 0 | 0 | 0 | 0 | 1 |
| NEGR1 | 2 | 0 | 0 | 0 | 0 | 0 | 0 | 0 | 1 |
| TRPM8 | 1 | 1 | 0 | 0 | 0 | 0 | 0 | 0 | 1 |
| FCHO1 | 2 | 0 | 0 | 0 | 0 | 0 | 0 | 0 | 1 |
| CATSPERG | 2 | 0 | 0 | 0 | 0 | 0 | 0 | 0 | 1 |
| PALM3 | 2 | 0 | 0 | 0 | 0 | 0 | 0 | 0 | 1 |
| SEMA6B | 2 | 0 | 0 | 0 | 0 | 0 | 0 | 0 | 1 |
| MAP3K2 | 2 | 0 | 0 | 0 | 0 | 0 | 0 | 0 | 1 |
| SIRPB1 | 2 | 0 | 0 | 0 | 0 | 0 | 0 | 0 | 1 |
| ALAS2 | 2 | 0 | 0 | 0 | 0 | 0 | 0 | 0 | 1 |

| | | | | | | | | | |
|---|---|---|---|---|---|---|---|---|---|
| FGD2 | 2 | 0 | 0 | 0 | 0 | 0 | 0 | 0 | 1 |
| SIRT1 | 2 | 0 | 0 | 0 | 0 | 0 | 0 | 0 | 1 |
| DNAJC1 | 2 | 0 | 0 | 0 | 0 | 0 | 0 | 0 | 1 |
| KIF3B | 2 | 0 | 0 | 0 | 0 | 0 | 0 | 0 | 1 |
| NPHP1 | 2 | 0 | 0 | 0 | 0 | 0 | 0 | 0 | 1 |
| ZSWIM2 | 2 | 0 | 0 | 0 | 0 | 0 | 0 | 0 | 1 |
| CCDC36 | 2 | 0 | 0 | 0 | 0 | 0 | 0 | 0 | 1 |
| EPB41L5 | 1 | 0 | 0 | 0 | 1 | 0 | 0 | 0 | 1 |
| BCHE | 2 | 0 | 0 | 0 | 0 | 0 | 0 | 0 | 1 |
| DMP1 | 2 | 0 | 0 | 0 | 0 | 0 | 0 | 0 | 1 |
| DPY19L3 | 2 | 0 | 0 | 0 | 0 | 0 | 0 | 0 | 1 |
| RFX2 | 2 | 0 | 0 | 0 | 0 | 0 | 0 | 0 | 1 |
| CHAF1A | 2 | 0 | 0 | 0 | 0 | 0 | 0 | 0 | 1 |
| EXOC6 | 2 | 0 | 0 | 0 | 0 | 0 | 0 | 0 | 1 |
| USP33 | 2 | 0 | 0 | 0 | 0 | 0 | 0 | 0 | 1 |
| SPATA20 | 2 | 0 | 0 | 0 | 0 | 0 | 0 | 0 | 1 |
| CLEC6A | 0 | 2 | 0 | 0 | 0 | 0 | 0 | 0 | 1 |
| CDAN1 | 2 | 0 | 0 | 0 | 0 | 0 | 0 | 0 | 1 |
| CAPS2 | 2 | 0 | 0 | 0 | 0 | 0 | 0 | 0 | 1 |
| USP4 | 2 | 0 | 0 | 0 | 0 | 0 | 0 | 0 | 1 |
| BBS10 | 2 | 0 | 0 | 0 | 0 | 0 | 0 | 0 | 1 |
| BUB1B | 2 | 0 | 0 | 0 | 0 | 0 | 0 | 0 | 1 |
| RFX4 | 2 | 0 | 0 | 0 | 0 | 0 | 0 | 0 | 1 |
| OR4C15 | 1 | 1 | 0 | 0 | 0 | 0 | 0 | 0 | 1 |
| ADAMTS8 | 2 | 0 | 0 | 0 | 0 | 0 | 0 | 0 | 1 |
| SLC39A12 | 2 | 0 | 0 | 0 | 0 | 0 | 0 | 0 | 1 |
| PRKAG3 | 2 | 0 | 0 | 0 | 0 | 0 | 0 | 0 | 1 |
| TACR3 | 2 | 0 | 0 | 0 | 0 | 0 | 0 | 0 | 1 |
| TUT1 | 2 | 0 | 0 | 0 | 0 | 0 | 0 | 0 | 1 |
| ABCF3 | 2 | 0 | 0 | 0 | 0 | 0 | 0 | 0 | 1 |
| SMR3A | 2 | 0 | 0 | 0 | 0 | 0 | 0 | 0 | 1 |
| CELF4 | 2 | 0 | 0 | 0 | 0 | 0 | 0 | 0 | 1 |
| FAM120B | 2 | 0 | 0 | 0 | 0 | 0 | 0 | 0 | 1 |
| PRICKLE1 | 1 | 1 | 0 | 0 | 0 | 0 | 0 | 0 | 1 |
| PLAT | 2 | 0 | 0 | 0 | 0 | 0 | 0 | 0 | 1 |
| AEBP1 | 1 | 1 | 0 | 0 | 0 | 0 | 0 | 0 | 1 |
| FANCI | 2 | 0 | 0 | 0 | 0 | 0 | 0 | 0 | 1 |
| AIRE | 2 | 0 | 0 | 0 | 0 | 0 | 0 | 0 | 1 |
| ARHGAP40 | 2 | 0 | 0 | 0 | 0 | 0 | 0 | 0 | 1 |
| DIAPH2 | 2 | 0 | 0 | 0 | 0 | 0 | 0 | 0 | 1 |
| OR6P1 | 2 | 0 | 0 | 0 | 0 | 0 | 0 | 0 | 1 |
| BIN2 | 2 | 0 | 0 | 0 | 0 | 0 | 0 | 0 | 1 |
| SLC4A4 | 1 | 0 | 1 | 0 | 0 | 0 | 0 | 0 | 1 |
| RTN3 | 2 | 0 | 0 | 0 | 0 | 0 | 0 | 0 | 1 |
| ABR | 2 | 0 | 0 | 0 | 0 | 0 | 0 | 0 | 1 |
| PCDHA5 | 2 | 0 | 0 | 0 | 0 | 0 | 0 | 0 | 1 |

| | | | | | | | | | |
|---|---|---|---|---|---|---|---|---|---|
| KIAA1274 | 2 | 0 | 0 | 0 | 0 | 0 | 0 | 0 | 1 |
| PKD2L1 | 2 | 0 | 0 | 0 | 0 | 0 | 0 | 0 | 1 |
| NELL1 | 2 | 0 | 0 | 0 | 0 | 0 | 0 | 0 | 1 |
| KRT34 | 2 | 0 | 0 | 0 | 0 | 0 | 0 | 0 | 1 |
| ADAMTS5 | 1 | 0 | 0 | 0 | 1 | 0 | 0 | 0 | 1 |
| SCUBE2 | 2 | 0 | 0 | 0 | 0 | 0 | 0 | 0 | 1 |
| ZNF546 | 2 | 0 | 0 | 0 | 0 | 0 | 0 | 0 | 1 |
| PRKACB | 1 | 1 | 0 | 0 | 0 | 0 | 0 | 0 | 1 |
| TF | 2 | 0 | 0 | 0 | 0 | 0 | 0 | 0 | 1 |
| KRT10 | 2 | 0 | 0 | 0 | 0 | 0 | 0 | 0 | 1 |
| TTC27 | 2 | 0 | 0 | 0 | 0 | 0 | 0 | 0 | 1 |
| RMI1 | 2 | 0 | 0 | 0 | 0 | 0 | 0 | 0 | 1 |
| OTUD4 | 2 | 0 | 0 | 0 | 0 | 0 | 0 | 0 | 1 |
| ZDHHC8 | 2 | 0 | 0 | 0 | 0 | 0 | 0 | 0 | 1 |
| PWP1 | 2 | 0 | 0 | 0 | 0 | 0 | 0 | 0 | 1 |
| KIAA1530 | 2 | 0 | 0 | 0 | 0 | 0 | 0 | 0 | 1 |
| ZNF626 | 2 | 0 | 0 | 0 | 0 | 0 | 0 | 0 | 1 |
| STK10 | 2 | 0 | 0 | 0 | 0 | 0 | 0 | 0 | 1 |
| ICA1 | 2 | 0 | 0 | 0 | 0 | 0 | 0 | 0 | 1 |
| R3HDM1 | 2 | 0 | 0 | 0 | 0 | 0 | 0 | 0 | 1 |
| SYDE2 | 2 | 0 | 0 | 0 | 0 | 0 | 0 | 0 | 1 |
| SCNN1G | 2 | 0 | 0 | 0 | 0 | 0 | 0 | 0 | 1 |
| TMPO | 1 | 0 | 0 | 0 | 1 | 0 | 0 | 0 | 1 |
| IPO11 | 1 | 1 | 0 | 0 | 0 | 0 | 0 | 0 | 1 |
| ARFIP2 | 2 | 0 | 0 | 0 | 0 | 0 | 0 | 0 | 1 |
| ACSL1 | 1 | 1 | 0 | 0 | 0 | 0 | 0 | 0 | 1 |
| C8B | 2 | 0 | 0 | 0 | 0 | 0 | 0 | 0 | 1 |
| VAC14 | 2 | 0 | 0 | 0 | 0 | 0 | 0 | 0 | 1 |
| DUSP16 | 2 | 0 | 0 | 0 | 0 | 0 | 0 | 0 | 1 |
| C2orf63 | 2 | 0 | 0 | 0 | 0 | 0 | 0 | 0 | 1 |
| DDB1 | 2 | 0 | 0 | 0 | 0 | 0 | 0 | 0 | 1 |
| ABTB2 | 2 | 0 | 0 | 0 | 0 | 0 | 0 | 0 | 1 |
| XKR5 | 2 | 0 | 0 | 0 | 0 | 0 | 0 | 0 | 1 |
| USP7 | 2 | 0 | 0 | 0 | 0 | 0 | 0 | 0 | 1 |
| TGS1 | 2 | 0 | 0 | 0 | 0 | 0 | 0 | 0 | 1 |
| CCDC60 | 1 | 1 | 0 | 0 | 0 | 0 | 0 | 0 | 1 |
| RAPGEF4 | 1 | 1 | 0 | 0 | 0 | 0 | 0 | 0 | 1 |
| OASL | 0 | 2 | 0 | 0 | 0 | 0 | 0 | 0 | 1 |
| ADAMTS4 | 2 | 0 | 0 | 0 | 0 | 0 | 0 | 0 | 1 |
| CLGN | 2 | 0 | 0 | 0 | 0 | 0 | 0 | 0 | 1 |
| PRDM8 | 2 | 0 | 0 | 0 | 0 | 0 | 0 | 0 | 1 |
| PHLDB3 | 2 | 0 | 0 | 0 | 0 | 0 | 0 | 0 | 1 |
| CLCN5 | 2 | 0 | 0 | 0 | 0 | 0 | 0 | 0 | 1 |
| GRAMD1A | 2 | 0 | 0 | 0 | 0 | 0 | 0 | 0 | 1 |
| SREBF1 | 2 | 0 | 0 | 0 | 0 | 0 | 0 | 0 | 1 |
| HBE1 | 2 | 0 | 0 | 0 | 0 | 0 | 0 | 0 | 1 |

| | | | | | | | | | |
|---|---|---|---|---|---|---|---|---|---|
| SLC17A6 | 2 | 0 | 0 | 0 | 0 | 0 | 0 | 0 | 1 |
| VTN | 2 | 0 | 0 | 0 | 0 | 0 | 0 | 0 | 1 |
| KIAA1324L | 2 | 0 | 0 | 0 | 0 | 0 | 0 | 0 | 1 |
| KIAA0408 | 2 | 0 | 0 | 0 | 0 | 0 | 0 | 0 | 1 |
| SLFN14 | 2 | 0 | 0 | 0 | 0 | 0 | 0 | 0 | 1 |
| PCDHB1 | 2 | 0 | 0 | 0 | 0 | 0 | 0 | 0 | 1 |
| ZNF540 | 2 | 0 | 0 | 0 | 0 | 0 | 0 | 0 | 1 |
| DDX26B | 2 | 0 | 0 | 0 | 0 | 0 | 0 | 0 | 1 |
| TYRO3 | 2 | 0 | 0 | 0 | 0 | 0 | 0 | 0 | 1 |
| KIF9 | 2 | 0 | 0 | 0 | 0 | 0 | 0 | 0 | 1 |
| MFSD6L | 2 | 0 | 0 | 0 | 0 | 0 | 0 | 0 | 1 |
| ARHGEF7 | 2 | 0 | 0 | 0 | 0 | 0 | 0 | 0 | 1 |
| GUF1 | 2 | 0 | 0 | 0 | 0 | 0 | 0 | 0 | 1 |
| PRPF6 | 2 | 0 | 0 | 0 | 0 | 0 | 0 | 0 | 1 |
| PCDHB5 | 2 | 0 | 0 | 0 | 0 | 0 | 0 | 0 | 1 |
| ZC3H6 | 2 | 0 | 0 | 0 | 0 | 0 | 0 | 0 | 1 |
| MEI1 | 2 | 0 | 0 | 0 | 0 | 0 | 0 | 0 | 1 |
| DSC3 | 2 | 0 | 0 | 0 | 0 | 0 | 0 | 0 | 1 |
| HBB | 2 | 0 | 0 | 0 | 0 | 0 | 0 | 0 | 1 |
| AMOT | 2 | 0 | 0 | 0 | 0 | 0 | 0 | 0 | 1 |
| ZMYND15 | 2 | 0 | 0 | 0 | 0 | 0 | 0 | 0 | 1 |
| TRPC5 | 2 | 0 | 0 | 0 | 0 | 0 | 0 | 0 | 1 |
| VAT1L | 2 | 0 | 0 | 0 | 0 | 0 | 0 | 0 | 1 |
| PIP5K1C | 2 | 0 | 0 | 0 | 0 | 0 | 0 | 0 | 1 |
| SND1 | 2 | 0 | 0 | 0 | 0 | 0 | 0 | 0 | 1 |
| BTNL9 | 2 | 0 | 0 | 0 | 0 | 0 | 0 | 0 | 1 |
| HNRNPUL2 | 2 | 0 | 0 | 0 | 0 | 0 | 0 | 0 | 1 |
| MYRIP | 1 | 1 | 0 | 0 | 0 | 0 | 0 | 0 | 1 |
| OGDH | 2 | 0 | 0 | 0 | 0 | 0 | 0 | 0 | 1 |
| VLDLR | 2 | 0 | 0 | 0 | 0 | 0 | 0 | 0 | 1 |
| UBAP2L | 2 | 0 | 0 | 0 | 0 | 0 | 0 | 0 | 1 |
| DIS3L2 | 2 | 0 | 0 | 0 | 0 | 0 | 0 | 0 | 1 |
| C8orf34 | 2 | 0 | 0 | 0 | 0 | 0 | 0 | 0 | 1 |
| ATP13A1 | 2 | 0 | 0 | 0 | 0 | 0 | 0 | 0 | 1 |
| PJA2 | 2 | 0 | 0 | 0 | 0 | 0 | 0 | 0 | 1 |
| TTC39B | 1 | 0 | 0 | 0 | 1 | 0 | 0 | 0 | 1 |
| ESF1 | 2 | 0 | 0 | 0 | 0 | 0 | 0 | 0 | 1 |
| MIB2 | 2 | 0 | 0 | 0 | 0 | 0 | 0 | 0 | 1 |
| ZNF217 | 2 | 0 | 0 | 0 | 0 | 0 | 0 | 0 | 1 |
| MFRP | 2 | 0 | 0 | 0 | 0 | 0 | 0 | 0 | 1 |
| ARHGEF2 | 2 | 0 | 0 | 0 | 0 | 0 | 0 | 0 | 1 |
| PARP1 | 2 | 0 | 0 | 0 | 0 | 0 | 0 | 0 | 1 |
| DZIP1L | 2 | 0 | 0 | 0 | 0 | 0 | 0 | 0 | 1 |
| C7orf66 | 2 | 0 | 0 | 0 | 0 | 0 | 0 | 0 | 1 |
| HKR1 | 2 | 0 | 0 | 0 | 0 | 0 | 0 | 0 | 1 |
| HEATR7A | 2 | 0 | 0 | 0 | 0 | 0 | 0 | 0 | 1 |

| | | | | | | | | | |
|---|---|---|---|---|---|---|---|---|---|
| RHBDF1 | 2 | 0 | 0 | 0 | 0 | 0 | 0 | 0 | 1 |
| USP13 | 2 | 0 | 0 | 0 | 0 | 0 | 0 | 0 | 1 |
| PLEKHA7 | 2 | 0 | 0 | 0 | 0 | 0 | 0 | 0 | 1 |
| SCYL3 | 2 | 0 | 0 | 0 | 0 | 0 | 0 | 0 | 1 |
| ANKS1A | 2 | 0 | 0 | 0 | 0 | 0 | 0 | 0 | 1 |
| IL1RAPL2 | 1 | 0 | 0 | 0 | 1 | 0 | 0 | 0 | 1 |
| ADCY4 | 2 | 0 | 0 | 0 | 0 | 0 | 0 | 0 | 1 |
| SERPINA4 | 2 | 0 | 0 | 0 | 0 | 0 | 0 | 0 | 1 |
| FAM59B | 2 | 0 | 0 | 0 | 0 | 0 | 0 | 0 | 1 |
| PLCB2 | 2 | 0 | 0 | 0 | 0 | 0 | 0 | 0 | 1 |
| AARSD1 | 2 | 0 | 0 | 0 | 0 | 0 | 0 | 0 | 1 |
| CDCA2 | 2 | 0 | 0 | 0 | 0 | 0 | 0 | 0 | 1 |
| ACCSL | 2 | 0 | 0 | 0 | 0 | 0 | 0 | 0 | 1 |
| MYO1G | 2 | 0 | 0 | 0 | 0 | 0 | 0 | 0 | 1 |
| MGAT5 | 2 | 0 | 0 | 0 | 0 | 0 | 0 | 0 | 1 |
| ELAC2 | 2 | 0 | 0 | 0 | 0 | 0 | 0 | 0 | 1 |
| FMR1 | 2 | 0 | 0 | 0 | 0 | 0 | 0 | 0 | 1 |
| AMBRA1 | 2 | 0 | 0 | 0 | 0 | 0 | 0 | 0 | 1 |
| KAL1 | 2 | 0 | 0 | 0 | 0 | 0 | 0 | 0 | 1 |
| PCDHGB3 | 2 | 0 | 0 | 0 | 0 | 0 | 0 | 0 | 1 |
| KIF2B | 2 | 0 | 0 | 0 | 0 | 0 | 0 | 0 | 1 |
| TAF5 | 2 | 0 | 0 | 0 | 0 | 0 | 0 | 0 | 1 |
| ZNF420 | 2 | 0 | 0 | 0 | 0 | 0 | 0 | 0 | 1 |
| HAS1 | 2 | 0 | 0 | 0 | 0 | 0 | 0 | 0 | 1 |
| SRRM5 | 2 | 0 | 0 | 0 | 0 | 0 | 0 | 0 | 1 |
| DHX30 | 2 | 0 | 0 | 0 | 0 | 0 | 0 | 0 | 1 |
| PDE4A | 2 | 0 | 0 | 0 | 0 | 0 | 0 | 0 | 1 |
| COL6A1 | 2 | 0 | 0 | 0 | 0 | 0 | 0 | 0 | 1 |
| POLR3B | 2 | 0 | 0 | 0 | 0 | 0 | 0 | 0 | 1 |
| SEC31A | 2 | 0 | 0 | 0 | 0 | 0 | 0 | 0 | 1 |
| MRVI1 | 2 | 0 | 0 | 0 | 0 | 0 | 0 | 0 | 1 |
| ANKIB1 | 2 | 0 | 0 | 0 | 0 | 0 | 0 | 0 | 1 |
| KIAA1522 | 2 | 0 | 0 | 0 | 0 | 0 | 0 | 0 | 1 |
| PREPL | 2 | 0 | 0 | 0 | 0 | 0 | 0 | 0 | 1 |
| CWC22 | 2 | 0 | 0 | 0 | 0 | 0 | 0 | 0 | 1 |
| SLC26A4 | 2 | 0 | 0 | 0 | 0 | 0 | 0 | 0 | 1 |
| LIPJ | 2 | 0 | 0 | 0 | 0 | 0 | 0 | 0 | 1 |
| GAA | 2 | 0 | 0 | 0 | 0 | 0 | 0 | 0 | 1 |
| PHC1 | 1 | 1 | 0 | 0 | 0 | 0 | 0 | 0 | 1 |
| EFCAB7 | 2 | 0 | 0 | 0 | 0 | 0 | 0 | 0 | 1 |
| ZNF107 | 2 | 0 | 0 | 0 | 0 | 0 | 0 | 0 | 1 |
| CNKSR2 | 2 | 0 | 0 | 0 | 0 | 0 | 0 | 0 | 1 |
| STIL | 2 | 0 | 0 | 0 | 0 | 0 | 0 | 0 | 1 |
| RNF180 | 2 | 0 | 0 | 0 | 0 | 0 | 0 | 0 | 1 |
| ELP2 | 2 | 0 | 0 | 0 | 0 | 0 | 0 | 0 | 1 |
| C1orf101 | 2 | 0 | 0 | 0 | 0 | 0 | 0 | 0 | 1 |

| | | | | | | | | | |
|---|---|---|---|---|---|---|---|---|---|
| TIMELESS | 1 | 1 | 0 | 0 | 0 | 0 | 0 | 0 | 1 |
| PRKAR2B | 2 | 0 | 0 | 0 | 0 | 0 | 0 | 0 | 1 |
| CDC20B | 2 | 0 | 0 | 0 | 0 | 0 | 0 | 0 | 1 |
| SKIV2L | 2 | 0 | 0 | 0 | 0 | 0 | 0 | 0 | 1 |
| IDE | 2 | 0 | 0 | 0 | 0 | 0 | 0 | 0 | 1 |
| MEX3B | 2 | 0 | 0 | 0 | 0 | 0 | 0 | 0 | 1 |
| MAP3K10 | 2 | 0 | 0 | 0 | 0 | 0 | 0 | 0 | 1 |
| ZRANB3 | 2 | 0 | 0 | 0 | 0 | 0 | 0 | 0 | 1 |
| MTA3 | 2 | 0 | 0 | 0 | 0 | 0 | 0 | 0 | 1 |
| MYO1C | 2 | 0 | 0 | 0 | 0 | 0 | 0 | 0 | 1 |
| FAM13A | 2 | 0 | 0 | 0 | 0 | 0 | 0 | 0 | 1 |
| FAM83C | 2 | 0 | 0 | 0 | 0 | 0 | 0 | 0 | 1 |
| TCERG1 | 2 | 0 | 0 | 0 | 0 | 0 | 0 | 0 | 1 |
| C1orf116 | 2 | 0 | 0 | 0 | 0 | 0 | 0 | 0 | 1 |
| ST6GAL2 | 2 | 0 | 0 | 0 | 0 | 0 | 0 | 0 | 1 |
| GPR113 | 2 | 0 | 0 | 0 | 0 | 0 | 0 | 0 | 1 |
| METAP2 | 2 | 0 | 0 | 0 | 0 | 0 | 0 | 0 | 1 |
| PIK3C2B | 1 | 1 | 0 | 0 | 0 | 0 | 0 | 0 | 1 |
| FOXM1 | 2 | 0 | 0 | 0 | 0 | 0 | 0 | 0 | 1 |
| ABP1 | 2 | 0 | 0 | 0 | 0 | 0 | 0 | 0 | 1 |
| IRS4 | 2 | 0 | 0 | 0 | 0 | 0 | 0 | 0 | 1 |
| ZNF248 | 2 | 0 | 0 | 0 | 0 | 0 | 0 | 0 | 1 |
| CDK12 | 2 | 0 | 0 | 0 | 0 | 0 | 0 | 0 | 1 |
| OGT | 2 | 0 | 0 | 0 | 0 | 0 | 0 | 0 | 1 |
| OSGEP | 2 | 0 | 0 | 0 | 0 | 0 | 0 | 0 | 1 |
| HPX | 2 | 0 | 0 | 0 | 0 | 0 | 0 | 0 | 1 |
| ZNF518B | 1 | 1 | 0 | 0 | 0 | 0 | 0 | 0 | 1 |
| GABRG2 | 2 | 0 | 0 | 0 | 0 | 0 | 0 | 0 | 1 |
| COL9A3 | 2 | 0 | 0 | 0 | 0 | 0 | 0 | 0 | 1 |
| MLLT6 | 2 | 0 | 0 | 0 | 0 | 0 | 0 | 0 | 1 |
| ATP2A3 | 2 | 0 | 0 | 0 | 0 | 0 | 0 | 0 | 1 |
| VPS53 | 2 | 0 | 0 | 0 | 0 | 0 | 0 | 0 | 1 |
| NELF | 2 | 0 | 0 | 0 | 0 | 0 | 0 | 0 | 1 |
| MYBPC3 | 2 | 0 | 0 | 0 | 0 | 0 | 0 | 0 | 1 |
| FAM160B1 | 2 | 0 | 0 | 0 | 0 | 0 | 0 | 0 | 1 |
| MAP7D1 | 2 | 0 | 0 | 0 | 0 | 0 | 0 | 0 | 1 |
| TTLL7 | 2 | 0 | 0 | 0 | 0 | 0 | 0 | 0 | 1 |
| OR2M5 | 1 | 0 | 0 | 0 | 1 | 0 | 0 | 0 | 1 |
| TMEM156 | 1 | 0 | 0 | 0 | 1 | 0 | 0 | 0 | 1 |
| TLE2 | 2 | 0 | 0 | 0 | 0 | 0 | 0 | 0 | 1 |
| SPTBN2 | 0 | 1 | 0 | 0 | 0 | 0 | 1 | 0 | 1 |
| LHX9 | 2 | 0 | 0 | 0 | 0 | 0 | 0 | 0 | 1 |
| GTSE1 | 2 | 0 | 0 | 0 | 0 | 0 | 0 | 0 | 1 |
| AZI1 | 2 | 0 | 0 | 0 | 0 | 0 | 0 | 0 | 1 |
| IRX1 | 2 | 0 | 0 | 0 | 0 | 0 | 0 | 0 | 1 |
| SCMH1 | 2 | 0 | 0 | 0 | 0 | 0 | 0 | 0 | 1 |

| | | | | | | | | | |
|---|---|---|---|---|---|---|---|---|---|
| NRP1 | 2 | 0 | 0 | 0 | 0 | 0 | 0 | 0 | 1 |
| MAN2B2 | 2 | 0 | 0 | 0 | 0 | 0 | 0 | 0 | 1 |
| POLR3A | 2 | 0 | 0 | 0 | 0 | 0 | 0 | 0 | 1 |
| ANO8 | 1 | 1 | 0 | 0 | 0 | 0 | 0 | 0 | 1 |
| STT3A | 2 | 0 | 0 | 0 | 0 | 0 | 0 | 0 | 1 |
| ZNF274 | 2 | 0 | 0 | 0 | 0 | 0 | 0 | 0 | 1 |
| UBQLN1 | 1 | 1 | 0 | 0 | 0 | 0 | 0 | 0 | 1 |
| PHACTR1 | 2 | 0 | 0 | 0 | 0 | 0 | 0 | 0 | 1 |
| ZNF548 | 2 | 0 | 0 | 0 | 0 | 0 | 0 | 0 | 1 |
| ALG13 | 2 | 0 | 0 | 0 | 0 | 0 | 0 | 0 | 1 |
| CIITA | 2 | 0 | 0 | 0 | 0 | 0 | 0 | 0 | 1 |
| RBM6 | 2 | 0 | 0 | 0 | 0 | 0 | 0 | 0 | 1 |
| CBLB | 2 | 0 | 0 | 0 | 0 | 0 | 0 | 0 | 1 |
| IL4I1 | 2 | 0 | 0 | 0 | 0 | 0 | 0 | 0 | 1 |
| NEK11 | 2 | 0 | 0 | 0 | 0 | 0 | 0 | 0 | 1 |
| XKR6 | 2 | 0 | 0 | 0 | 0 | 0 | 0 | 0 | 1 |
| UBE3B | 2 | 0 | 0 | 0 | 0 | 0 | 0 | 0 | 1 |
| SLC8A3 | 2 | 0 | 0 | 0 | 0 | 0 | 0 | 0 | 1 |
| MFAP1 | 2 | 0 | 0 | 0 | 0 | 0 | 0 | 0 | 1 |
| ZNF507 | 2 | 0 | 0 | 0 | 0 | 0 | 0 | 0 | 1 |
| KNG1 | 2 | 0 | 0 | 0 | 0 | 0 | 0 | 0 | 1 |
| SH3PXD2B | 2 | 0 | 0 | 0 | 0 | 0 | 0 | 0 | 1 |
| CCL14 | 2 | 0 | 0 | 0 | 0 | 0 | 0 | 0 | 1 |
| ZSWIM6 | 2 | 0 | 0 | 0 | 0 | 0 | 0 | 0 | 1 |
| ZNF526 | 2 | 0 | 0 | 0 | 0 | 0 | 0 | 0 | 1 |
| COL8A1 | 2 | 0 | 0 | 0 | 0 | 0 | 0 | 0 | 1 |
| STK36 | 2 | 0 | 0 | 0 | 0 | 0 | 0 | 0 | 1 |
| APP | 2 | 0 | 0 | 0 | 0 | 0 | 0 | 0 | 1 |
| CRNKL1 | 2 | 0 | 0 | 0 | 0 | 0 | 0 | 0 | 1 |
| CRB2 | 2 | 0 | 0 | 0 | 0 | 0 | 0 | 0 | 1 |
| LPIN2 | 2 | 0 | 0 | 0 | 0 | 0 | 0 | 0 | 1 |
| IRS1 | 2 | 0 | 0 | 0 | 0 | 0 | 0 | 0 | 1 |
| TTC39C | 2 | 0 | 0 | 0 | 0 | 0 | 0 | 0 | 1 |
| JHDM1D | 2 | 0 | 0 | 0 | 0 | 0 | 0 | 0 | 1 |
| AAK1 | 2 | 0 | 0 | 0 | 0 | 0 | 0 | 0 | 1 |
| SPATA8 | 2 | 0 | 0 | 0 | 0 | 0 | 0 | 0 | 1 |
| ITGA5 | 2 | 0 | 0 | 0 | 0 | 0 | 0 | 0 | 1 |
| EIF4G2 | 2 | 0 | 0 | 0 | 0 | 0 | 0 | 0 | 1 |
| EVC | 2 | 0 | 0 | 0 | 0 | 0 | 0 | 0 | 1 |
| ATF6 | 2 | 0 | 0 | 0 | 0 | 0 | 0 | 0 | 1 |
| ZNF189 | 2 | 0 | 0 | 0 | 0 | 0 | 0 | 0 | 1 |
| GABRR2 | 2 | 0 | 0 | 0 | 0 | 0 | 0 | 0 | 1 |
| C9orf131 | 2 | 0 | 0 | 0 | 0 | 0 | 0 | 0 | 1 |
| MARK1 | 2 | 0 | 0 | 0 | 0 | 0 | 0 | 0 | 1 |
| BAZ1B | 2 | 0 | 0 | 0 | 0 | 0 | 0 | 0 | 1 |
| PTPN23 | 1 | 0 | 0 | 0 | 1 | 0 | 0 | 0 | 1 |

| | | | | | | | | | |
|---|---|---|---|---|---|---|---|---|---|
| HMGXB3 | 2 | 0 | 0 | 0 | 0 | 0 | 0 | 0 | 1 |
| MPHOSPH9 | 2 | 0 | 0 | 0 | 0 | 0 | 0 | 0 | 1 |
| EFHA2 | 2 | 0 | 0 | 0 | 0 | 0 | 0 | 0 | 1 |
| CHERP | 2 | 0 | 0 | 0 | 0 | 0 | 0 | 0 | 1 |
| SGSM2 | 2 | 0 | 0 | 0 | 0 | 0 | 0 | 0 | 1 |
| KIFAP3 | 2 | 0 | 0 | 0 | 0 | 0 | 0 | 0 | 1 |
| CSRNP3 | 2 | 0 | 0 | 0 | 0 | 0 | 0 | 0 | 1 |
| ABCB10 | 2 | 0 | 0 | 0 | 0 | 0 | 0 | 0 | 1 |
| KL | 2 | 0 | 0 | 0 | 0 | 0 | 0 | 0 | 1 |
| DOT1L | 2 | 0 | 0 | 0 | 0 | 0 | 0 | 0 | 1 |
| RBM19 | 2 | 0 | 0 | 0 | 0 | 0 | 0 | 0 | 1 |
| DDHD1 | 2 | 0 | 0 | 0 | 0 | 0 | 0 | 0 | 1 |
| LIM2 | 2 | 0 | 0 | 0 | 0 | 0 | 0 | 0 | 1 |
| ZC3H13 | 2 | 0 | 0 | 0 | 0 | 0 | 0 | 0 | 1 |
| ZNF687 | 2 | 0 | 0 | 0 | 0 | 0 | 0 | 0 | 1 |
| NPR2 | 2 | 0 | 0 | 0 | 0 | 0 | 0 | 0 | 1 |
| SENP6 | 1 | 0 | 0 | 0 | 1 | 0 | 0 | 0 | 1 |
| KIAA1683 | 2 | 0 | 0 | 0 | 0 | 0 | 0 | 0 | 1 |
| SERGEF | 1 | 1 | 0 | 0 | 0 | 0 | 0 | 0 | 1 |
| KLHL14 | 2 | 0 | 0 | 0 | 0 | 0 | 0 | 0 | 1 |
| DIAPH1 | 2 | 0 | 0 | 0 | 0 | 0 | 0 | 0 | 1 |
| NOS1 | 2 | 0 | 0 | 0 | 0 | 0 | 0 | 0 | 1 |
| MDGA1 | 2 | 0 | 0 | 0 | 0 | 0 | 0 | 0 | 1 |
| PDE3B | 2 | 0 | 0 | 0 | 0 | 0 | 0 | 0 | 1 |
| C10orf28 | 2 | 0 | 0 | 0 | 0 | 0 | 0 | 0 | 1 |
| ZNF700 | 2 | 0 | 0 | 0 | 0 | 0 | 0 | 0 | 1 |
| EHBP1L1 | 2 | 0 | 0 | 0 | 0 | 0 | 0 | 0 | 1 |
| FAM184B | 2 | 0 | 0 | 0 | 0 | 0 | 0 | 0 | 1 |
| RTN4 | 2 | 0 | 0 | 0 | 0 | 0 | 0 | 0 | 1 |
| MTTP | 1 | 1 | 0 | 0 | 0 | 0 | 0 | 0 | 1 |
| KIAA0232 | 2 | 0 | 0 | 0 | 0 | 0 | 0 | 0 | 1 |
| TRERF1 | 2 | 0 | 0 | 0 | 0 | 0 | 0 | 0 | 1 |
| SNX31 | 2 | 0 | 0 | 0 | 0 | 0 | 0 | 0 | 1 |
| DIP2A | 2 | 0 | 0 | 0 | 0 | 0 | 0 | 0 | 1 |
| TJP2 | 2 | 0 | 0 | 0 | 0 | 0 | 0 | 0 | 1 |
| ANKFY1 | 2 | 0 | 0 | 0 | 0 | 0 | 0 | 0 | 1 |
| OPA1 | 2 | 0 | 0 | 0 | 0 | 0 | 0 | 0 | 1 |
| ULK2 | 2 | 0 | 0 | 0 | 0 | 0 | 0 | 0 | 1 |
| PADI3 | 2 | 0 | 0 | 0 | 0 | 0 | 0 | 0 | 1 |
| ANKRD35 | 2 | 0 | 0 | 0 | 0 | 0 | 0 | 0 | 1 |
| RET | 2 | 0 | 0 | 0 | 0 | 0 | 0 | 0 | 1 |
| PDZD4 | 2 | 0 | 0 | 0 | 0 | 0 | 0 | 0 | 1 |
| SPATA5 | 1 | 1 | 0 | 0 | 0 | 0 | 0 | 0 | 1 |
| OR13C8 | 2 | 0 | 0 | 0 | 0 | 0 | 0 | 0 | 1 |
| ATHL1 | 2 | 0 | 0 | 0 | 0 | 0 | 0 | 0 | 1 |
| PRIC285 | 1 | 1 | 0 | 0 | 0 | 0 | 0 | 0 | 1 |

| | | | | | | | | | |
|---|---|---|---|---|---|---|---|---|---|
| BMP1 | 2 | 0 | 0 | 0 | 0 | 0 | 0 | 0 | 1 |
| MYO1F | 2 | 0 | 0 | 0 | 0 | 0 | 0 | 0 | 1 |
| ZC3H12C | 2 | 0 | 0 | 0 | 0 | 0 | 0 | 0 | 1 |
| CASC1 | 2 | 0 | 0 | 0 | 0 | 0 | 0 | 0 | 1 |
| EPB41L2 | 2 | 0 | 0 | 0 | 0 | 0 | 0 | 0 | 1 |
| TLR9 | 2 | 0 | 0 | 0 | 0 | 0 | 0 | 0 | 1 |
| TP53BP2 | 2 | 0 | 0 | 0 | 0 | 0 | 0 | 0 | 1 |
| VCAM1 | 2 | 0 | 0 | 0 | 0 | 0 | 0 | 0 | 1 |
| CLSPN | 2 | 0 | 0 | 0 | 0 | 0 | 0 | 0 | 1 |
| TLE1 | 2 | 0 | 0 | 0 | 0 | 0 | 0 | 0 | 1 |
| SLC23A2 | 2 | 0 | 0 | 0 | 0 | 0 | 0 | 0 | 1 |
| TMEM144 | 2 | 0 | 0 | 0 | 0 | 0 | 0 | 0 | 1 |
| MAGEB16 | 2 | 0 | 0 | 0 | 0 | 0 | 0 | 0 | 1 |
| MYLK3 | 1 | 0 | 1 | 0 | 0 | 0 | 0 | 0 | 1 |
| OR6N2 | 2 | 0 | 0 | 0 | 0 | 0 | 0 | 0 | 1 |
| SBNO1 | 2 | 0 | 0 | 0 | 0 | 0 | 0 | 0 | 1 |
| NKTR | 2 | 0 | 0 | 0 | 0 | 0 | 0 | 0 | 1 |
| ESPL1 | 2 | 0 | 0 | 0 | 0 | 0 | 0 | 0 | 1 |
| USP20 | 2 | 0 | 0 | 0 | 0 | 0 | 0 | 0 | 1 |
| LRMP | 2 | 0 | 0 | 0 | 0 | 0 | 0 | 0 | 1 |
| SFI1 | 2 | 0 | 0 | 0 | 0 | 0 | 0 | 0 | 1 |
| R3HDM2 | 2 | 0 | 0 | 0 | 0 | 0 | 0 | 0 | 1 |
| ATP9B | 2 | 0 | 0 | 0 | 0 | 0 | 0 | 0 | 1 |
| MICAL1 | 2 | 0 | 0 | 0 | 0 | 0 | 0 | 0 | 1 |
| CHTF18 | 2 | 0 | 0 | 0 | 0 | 0 | 0 | 0 | 1 |
| S100A7L2 | 2 | 0 | 0 | 0 | 0 | 0 | 0 | 0 | 1 |
| C14orf43 | 2 | 0 | 0 | 0 | 0 | 0 | 0 | 0 | 1 |
| TGM5 | 2 | 0 | 0 | 0 | 0 | 0 | 0 | 0 | 1 |
| GLRB | 2 | 0 | 0 | 0 | 0 | 0 | 0 | 0 | 1 |
| USP26 | 1 | 1 | 0 | 0 | 0 | 0 | 0 | 0 | 1 |
| INPPL1 | 2 | 0 | 0 | 0 | 0 | 0 | 0 | 0 | 1 |
| OR2D2 | 2 | 0 | 0 | 0 | 0 | 0 | 0 | 0 | 1 |
| KIF15 | 2 | 0 | 0 | 0 | 0 | 0 | 0 | 0 | 1 |
| PHF2 | 2 | 0 | 0 | 0 | 0 | 0 | 0 | 0 | 1 |
| EP300 | 2 | 0 | 0 | 0 | 0 | 0 | 0 | 0 | 1 |
| ATP2B2 | 2 | 0 | 0 | 0 | 0 | 0 | 0 | 0 | 1 |
| CTR9 | 2 | 0 | 0 | 0 | 0 | 0 | 0 | 0 | 1 |
| STRN3 | 2 | 0 | 0 | 0 | 0 | 0 | 0 | 0 | 1 |
| LAS1L | 2 | 0 | 0 | 0 | 0 | 0 | 0 | 0 | 1 |
| CDKL5 | 2 | 0 | 0 | 0 | 0 | 0 | 0 | 0 | 1 |
| CDK5RAP3 | 2 | 0 | 0 | 0 | 0 | 0 | 0 | 0 | 1 |
| ARHGAP12 | 2 | 0 | 0 | 0 | 0 | 0 | 0 | 0 | 1 |
| TRIM66 | 2 | 0 | 0 | 0 | 0 | 0 | 0 | 0 | 1 |
| SEMA4G | 2 | 0 | 0 | 0 | 0 | 0 | 0 | 0 | 1 |
| PVRL1 | 2 | 0 | 0 | 0 | 0 | 0 | 0 | 0 | 1 |
| RAD54L2 | 2 | 0 | 0 | 0 | 0 | 0 | 0 | 0 | 1 |

| | | | | | | | | | |
|---|---|---|---|---|---|---|---|---|---|
| ERC2 | 2 | 0 | 0 | 0 | 0 | 0 | 0 | 0 | 1 |
| PALLD | 2 | 0 | 0 | 0 | 0 | 0 | 0 | 0 | 1 |
| LARS2 | 2 | 0 | 0 | 0 | 0 | 0 | 0 | 0 | 1 |
| SMARCAL1 | 2 | 0 | 0 | 0 | 0 | 0 | 0 | 0 | 1 |
| PHACTR4 | 2 | 0 | 0 | 0 | 0 | 0 | 0 | 0 | 1 |
| EGF | 2 | 0 | 0 | 0 | 0 | 0 | 0 | 0 | 1 |
| ASAP3 | 2 | 0 | 0 | 0 | 0 | 0 | 0 | 0 | 1 |
| MTMR10 | 2 | 0 | 0 | 0 | 0 | 0 | 0 | 0 | 1 |
| POLK | 1 | 1 | 0 | 0 | 0 | 0 | 0 | 0 | 1 |
| GPR124 | 2 | 0 | 0 | 0 | 0 | 0 | 0 | 0 | 1 |
| NAIF1 | 2 | 0 | 0 | 0 | 0 | 0 | 0 | 0 | 1 |
| RBM15 | 2 | 0 | 0 | 0 | 0 | 0 | 0 | 0 | 1 |
| KIF23 | 2 | 0 | 0 | 0 | 0 | 0 | 0 | 0 | 1 |
| CANX | 2 | 0 | 0 | 0 | 0 | 0 | 0 | 0 | 1 |
| BRPF1 | 2 | 0 | 0 | 0 | 0 | 0 | 0 | 0 | 1 |
| BRD3 | 2 | 0 | 0 | 0 | 0 | 0 | 0 | 0 | 1 |
| HERPUD2 | 1 | 1 | 0 | 0 | 0 | 0 | 0 | 0 | 1 |
| TLL1 | 2 | 0 | 0 | 0 | 0 | 0 | 0 | 0 | 1 |
| RARB | 2 | 0 | 0 | 0 | 0 | 0 | 0 | 0 | 1 |
| REN | 1 | 0 | 1 | 0 | 0 | 0 | 0 | 0 | 1 |
| PRLR | 2 | 0 | 0 | 0 | 0 | 0 | 0 | 0 | 1 |
| TMEM130 | 2 | 0 | 0 | 0 | 0 | 0 | 0 | 0 | 1 |
| PODN | 2 | 0 | 0 | 0 | 0 | 0 | 0 | 0 | 1 |
| DCTN1 | 2 | 0 | 0 | 0 | 0 | 0 | 0 | 0 | 1 |
| ATP1B4 | 2 | 0 | 0 | 0 | 0 | 0 | 0 | 0 | 1 |
| LRP8 | 2 | 0 | 0 | 0 | 0 | 0 | 0 | 0 | 1 |
| KIAA0528 | 2 | 0 | 0 | 0 | 0 | 0 | 0 | 0 | 1 |
| BBX | 2 | 0 | 0 | 0 | 0 | 0 | 0 | 0 | 1 |
| OR10G4 | 2 | 0 | 0 | 0 | 0 | 0 | 0 | 0 | 1 |
| FAM193A | 2 | 0 | 0 | 0 | 0 | 0 | 0 | 0 | 1 |
| LRRFIP1 | 2 | 0 | 0 | 0 | 0 | 0 | 0 | 0 | 1 |
| TTK | 2 | 0 | 0 | 0 | 0 | 0 | 0 | 0 | 1 |
| CAMSAP1 | 2 | 0 | 0 | 0 | 0 | 0 | 0 | 0 | 1 |
| NLRX1 | 1 | 1 | 0 | 0 | 0 | 0 | 0 | 0 | 1 |
| PWWP2A | 2 | 0 | 0 | 0 | 0 | 0 | 0 | 0 | 1 |
| ZNF623 | 2 | 0 | 0 | 0 | 0 | 0 | 0 | 0 | 1 |
| CCDC146 | 2 | 0 | 0 | 0 | 0 | 0 | 0 | 0 | 1 |
| ABCG2 | 2 | 0 | 0 | 0 | 0 | 0 | 0 | 0 | 1 |
| AXL | 2 | 0 | 0 | 0 | 0 | 0 | 0 | 0 | 1 |
| SLC8A1 | 2 | 0 | 0 | 0 | 0 | 0 | 0 | 0 | 1 |
| DLG4 | 2 | 0 | 0 | 0 | 0 | 0 | 0 | 0 | 1 |
| MSH2 | 1 | 1 | 0 | 0 | 0 | 0 | 0 | 0 | 1 |
| FYN | 2 | 0 | 0 | 0 | 0 | 0 | 0 | 0 | 1 |
| CDHR1 | 2 | 0 | 0 | 0 | 0 | 0 | 0 | 0 | 1 |
| CFI | 2 | 0 | 0 | 0 | 0 | 0 | 0 | 0 | 1 |
| BNC2 | 2 | 0 | 0 | 0 | 0 | 0 | 0 | 0 | 1 |

| | | | | | | | | |
|---|---|---|---|---|---|---|---|---|
| ADAMTS14 | 2 | 0 | 0 | 0 | 0 | 0 | 0 | 0 | 1 |
| TUSC3 | 2 | 0 | 0 | 0 | 0 | 0 | 0 | 0 | 1 |
| SLC9A1 | 2 | 0 | 0 | 0 | 0 | 0 | 0 | 0 | 1 |
| SLITRK1 | 2 | 0 | 0 | 0 | 0 | 0 | 0 | 0 | 1 |
| SSFA2 | 2 | 0 | 0 | 0 | 0 | 0 | 0 | 0 | 1 |
| BAI2 | 2 | 0 | 0 | 0 | 0 | 0 | 0 | 0 | 1 |
| SLC4A2 | 2 | 0 | 0 | 0 | 0 | 0 | 0 | 0 | 1 |
| SCGN | 2 | 0 | 0 | 0 | 0 | 0 | 0 | 0 | 1 |
| NHSL1 | 1 | 0 | 0 | 0 | 1 | 0 | 0 | 0 | 1 |
| ABCC6 | 2 | 0 | 0 | 0 | 0 | 0 | 0 | 0 | 1 |
| ZNF493 | 2 | 0 | 0 | 0 | 0 | 0 | 0 | 0 | 1 |
| RABGAP1L | 2 | 0 | 0 | 0 | 0 | 0 | 0 | 0 | 1 |
| TDRD7 | 2 | 0 | 0 | 0 | 0 | 0 | 0 | 0 | 1 |
| ZNF236 | 2 | 0 | 0 | 0 | 0 | 0 | 0 | 0 | 1 |
| PHF16 | 2 | 0 | 0 | 0 | 0 | 0 | 0 | 0 | 1 |
| ESYT3 | 1 | 1 | 0 | 0 | 0 | 0 | 0 | 0 | 1 |
| ADD2 | 2 | 0 | 0 | 0 | 0 | 0 | 0 | 0 | 1 |
| ARHGAP21 | 2 | 0 | 0 | 0 | 0 | 0 | 0 | 0 | 1 |
| OCA2 | 2 | 0 | 0 | 0 | 0 | 0 | 0 | 0 | 1 |
| SLTM | 2 | 0 | 0 | 0 | 0 | 0 | 0 | 0 | 1 |
| GOLGA3 | 2 | 0 | 0 | 0 | 0 | 0 | 0 | 0 | 1 |
| TTLL4 | 2 | 0 | 0 | 0 | 0 | 0 | 0 | 0 | 1 |
| PRKD3 | 2 | 0 | 0 | 0 | 0 | 0 | 0 | 0 | 1 |
| NSMAF | 2 | 0 | 0 | 0 | 0 | 0 | 0 | 0 | 1 |
| DRD3 | 2 | 0 | 0 | 0 | 0 | 0 | 0 | 0 | 1 |
| ZHX2 | 2 | 0 | 0 | 0 | 0 | 0 | 0 | 0 | 1 |
| HMMR | 1 | 1 | 0 | 0 | 0 | 0 | 0 | 0 | 1 |
| TERT | 2 | 0 | 0 | 0 | 0 | 0 | 0 | 0 | 1 |
| DAK | 2 | 0 | 0 | 0 | 0 | 0 | 0 | 0 | 1 |
| GRID2IP | 2 | 0 | 0 | 0 | 0 | 0 | 0 | 0 | 1 |
| MUT | 2 | 0 | 0 | 0 | 0 | 0 | 0 | 0 | 1 |
| PIK3CB | 2 | 0 | 0 | 0 | 0 | 0 | 0 | 0 | 1 |
| DIXDC1 | 2 | 0 | 0 | 0 | 0 | 0 | 0 | 0 | 1 |
| HTR4 | 2 | 0 | 0 | 0 | 0 | 0 | 0 | 0 | 1 |
| OR2D3 | 2 | 0 | 0 | 0 | 0 | 0 | 0 | 0 | 1 |
| SCRIB | 2 | 0 | 0 | 0 | 0 | 0 | 0 | 0 | 1 |
| PTPRJ | 2 | 0 | 0 | 0 | 0 | 0 | 0 | 0 | 1 |
| NLRP14 | 0 | 1 | 0 | 0 | 1 | 0 | 0 | 0 | 1 |
| SERTAD3 | 2 | 0 | 0 | 0 | 0 | 0 | 0 | 0 | 1 |
| BOC | 2 | 0 | 0 | 0 | 0 | 0 | 0 | 0 | 1 |
| LCE2A | 2 | 0 | 0 | 0 | 0 | 0 | 0 | 0 | 1 |
| HDAC6 | 1 | 1 | 0 | 0 | 0 | 0 | 0 | 0 | 1 |
| OR2A25 | 2 | 0 | 0 | 0 | 0 | 0 | 0 | 0 | 1 |
| CD300E | 2 | 0 | 0 | 0 | 0 | 0 | 0 | 0 | 1 |
| ZFP112 | 2 | 0 | 0 | 0 | 0 | 0 | 0 | 0 | 1 |
| SIDT1 | 2 | 0 | 0 | 0 | 0 | 0 | 0 | 0 | 1 |

| | | | | | | | | | |
|---|---|---|---|---|---|---|---|---|---|
| ADH1B | 2 | 0 | 0 | 0 | 0 | 0 | 0 | 0 | 1 |
| ARMC9 | 2 | 0 | 0 | 0 | 0 | 0 | 0 | 0 | 1 |
| THSD1 | 2 | 0 | 0 | 0 | 0 | 0 | 0 | 0 | 1 |
| ZNF451 | 2 | 0 | 0 | 0 | 0 | 0 | 0 | 0 | 1 |
| GPATCH8 | 2 | 0 | 0 | 0 | 0 | 0 | 0 | 0 | 1 |
| AHDC1 | 2 | 0 | 0 | 0 | 0 | 0 | 0 | 0 | 1 |
| GRM4 | 2 | 0 | 0 | 0 | 0 | 0 | 0 | 0 | 1 |
| OR8K1 | 2 | 0 | 0 | 0 | 0 | 0 | 0 | 0 | 1 |
| PBRM1 | 2 | 0 | 0 | 0 | 0 | 0 | 0 | 0 | 1 |
| NGEF | 2 | 0 | 0 | 0 | 0 | 0 | 0 | 0 | 1 |
| ACAD11 | 2 | 0 | 0 | 0 | 0 | 0 | 0 | 0 | 1 |
| PDE8A | 2 | 0 | 0 | 0 | 0 | 0 | 0 | 0 | 1 |
| PCSK9 | 2 | 0 | 0 | 0 | 0 | 0 | 0 | 0 | 1 |
| TNKS1BP1 | 2 | 0 | 0 | 0 | 0 | 0 | 0 | 0 | 1 |
| CHD1 | 2 | 0 | 0 | 0 | 0 | 0 | 0 | 0 | 1 |
| PLEKHG4B | 2 | 0 | 0 | 0 | 0 | 0 | 0 | 0 | 1 |
| CYP39A1 | 2 | 0 | 0 | 0 | 0 | 0 | 0 | 0 | 1 |
| ZNF41 | 2 | 0 | 0 | 0 | 0 | 0 | 0 | 0 | 1 |
| TOP3A | 2 | 0 | 0 | 0 | 0 | 0 | 0 | 0 | 1 |
| FSTL5 | 1 | 0 | 1 | 0 | 0 | 0 | 0 | 0 | 1 |
| NEUROD1 | 2 | 0 | 0 | 0 | 0 | 0 | 0 | 0 | 1 |
| GRIK4 | 2 | 0 | 0 | 0 | 0 | 0 | 0 | 0 | 1 |
| TBX20 | 2 | 0 | 0 | 0 | 0 | 0 | 0 | 0 | 1 |
| SPATA6 | 2 | 0 | 0 | 0 | 0 | 0 | 0 | 0 | 1 |
| GCC2 | 2 | 0 | 0 | 0 | 0 | 0 | 0 | 0 | 1 |
| MIA3 | 2 | 0 | 0 | 0 | 0 | 0 | 0 | 0 | 1 |
| CCNI | 2 | 0 | 0 | 0 | 0 | 0 | 0 | 0 | 1 |
| FMNL2 | 1 | 0 | 0 | 0 | 0 | 0 | 1 | 0 | 1 |
| TMCO7 | 2 | 0 | 0 | 0 | 0 | 0 | 0 | 0 | 1 |
| NLRC5 | 2 | 0 | 0 | 0 | 0 | 0 | 0 | 0 | 1 |
| OR2W3 | 2 | 0 | 0 | 0 | 0 | 0 | 0 | 0 | 1 |
| PLEKHH2 | 2 | 0 | 0 | 0 | 0 | 0 | 0 | 0 | 1 |
| TFAP2D | 2 | 0 | 0 | 0 | 0 | 0 | 0 | 0 | 1 |
| NEK8 | 2 | 0 | 0 | 0 | 0 | 0 | 0 | 0 | 1 |
| RABGAP1 | 1 | 1 | 0 | 0 | 0 | 0 | 0 | 0 | 1 |
| USP31 | 2 | 0 | 0 | 0 | 0 | 0 | 0 | 0 | 1 |
| MDC1 | 2 | 0 | 0 | 0 | 0 | 0 | 0 | 0 | 1 |
| EML4 | 2 | 0 | 0 | 0 | 0 | 0 | 0 | 0 | 1 |
| PSME4 | 2 | 0 | 0 | 0 | 0 | 0 | 0 | 0 | 1 |
| SIPA1L3 | 2 | 0 | 0 | 0 | 0 | 0 | 0 | 0 | 1 |
| ARPP21 | 2 | 0 | 0 | 0 | 0 | 0 | 0 | 0 | 1 |
| TNFRSF10D | 2 | 0 | 0 | 0 | 0 | 0 | 0 | 0 | 1 |
| PLXNA3 | 2 | 0 | 0 | 0 | 0 | 0 | 0 | 0 | 1 |
| TRIM6-TRIM34 | 2 | 0 | 0 | 0 | 0 | 0 | 0 | 0 | 1 |
| ESCO2 | 2 | 0 | 0 | 0 | 0 | 0 | 0 | 0 | 1 |
| ITGA6 | 2 | 0 | 0 | 0 | 0 | 0 | 0 | 0 | 1 |

| | | | | | | | | | |
|---|---|---|---|---|---|---|---|---|---|
| SLC12A6 | 2 | 0 | 0 | 0 | 0 | 0 | 0 | 0 | 1 |
| TSHZ2 | 2 | 0 | 0 | 0 | 0 | 0 | 0 | 0 | 1 |
| MYSM1 | 1 | 1 | 0 | 0 | 0 | 0 | 0 | 0 | 1 |
| KCNC3 | 2 | 0 | 0 | 0 | 0 | 0 | 0 | 0 | 1 |
| YME1L1 | 1 | 1 | 0 | 0 | 0 | 0 | 0 | 0 | 1 |
| MADD | 2 | 0 | 0 | 0 | 0 | 0 | 0 | 0 | 1 |
| RAG1 | 2 | 0 | 0 | 0 | 0 | 0 | 0 | 0 | 1 |
| LRIG1 | 2 | 0 | 0 | 0 | 0 | 0 | 0 | 0 | 1 |
| MCM7 | 2 | 0 | 0 | 0 | 0 | 0 | 0 | 0 | 1 |
| CHD4 | 2 | 0 | 0 | 0 | 0 | 0 | 0 | 0 | 1 |
| SRGAP3 | 2 | 0 | 0 | 0 | 0 | 0 | 0 | 0 | 1 |
| BRD8 | 1 | 1 | 0 | 0 | 0 | 0 | 0 | 0 | 1 |
| PCDH19 | 2 | 0 | 0 | 0 | 0 | 0 | 0 | 0 | 1 |
| FLNB | 2 | 0 | 0 | 0 | 0 | 0 | 0 | 0 | 1 |
| KIAA1549 | 1 | 1 | 0 | 0 | 0 | 0 | 0 | 0 | 1 |
| GALNT12 | 2 | 0 | 0 | 0 | 0 | 0 | 0 | 0 | 1 |
| USO1 | 2 | 0 | 0 | 0 | 0 | 0 | 0 | 0 | 1 |
| WRN | 2 | 0 | 0 | 0 | 0 | 0 | 0 | 0 | 1 |
| NUP133 | 2 | 0 | 0 | 0 | 0 | 0 | 0 | 0 | 1 |
| OR3A3 | 2 | 0 | 0 | 0 | 0 | 0 | 0 | 0 | 1 |
| GNPTAB | 2 | 0 | 0 | 0 | 0 | 0 | 0 | 0 | 1 |
| TOP2A | 2 | 0 | 0 | 0 | 0 | 0 | 0 | 0 | 1 |
| HR | 2 | 0 | 0 | 0 | 0 | 0 | 0 | 0 | 1 |
| C14orf135 | 1 | 1 | 0 | 0 | 0 | 0 | 0 | 0 | 1 |
| MYO1D | 2 | 0 | 0 | 0 | 0 | 0 | 0 | 0 | 1 |
| TM9SF4 | 2 | 0 | 0 | 0 | 0 | 0 | 0 | 0 | 1 |
| L1TD1 | 2 | 0 | 0 | 0 | 0 | 0 | 0 | 0 | 1 |
| ANO1 | 2 | 0 | 0 | 0 | 0 | 0 | 0 | 0 | 1 |
| GNAS | 2 | 0 | 0 | 0 | 0 | 0 | 0 | 0 | 1 |
| NEK1 | 2 | 0 | 0 | 0 | 0 | 0 | 0 | 0 | 1 |
| NSD1 | 1 | 1 | 0 | 0 | 0 | 0 | 0 | 0 | 1 |
| CADPS | 2 | 0 | 0 | 0 | 0 | 0 | 0 | 0 | 1 |
| GP2 | 1 | 1 | 0 | 0 | 0 | 0 | 0 | 0 | 1 |
| AIFM2 | 2 | 0 | 0 | 0 | 0 | 0 | 0 | 0 | 1 |
| PRDM10 | 2 | 0 | 0 | 0 | 0 | 0 | 0 | 0 | 1 |
| THBS4 | 2 | 0 | 0 | 0 | 0 | 0 | 0 | 0 | 1 |
| GPATCH1 | 2 | 0 | 0 | 0 | 0 | 0 | 0 | 0 | 1 |
| PLEKHA6 | 2 | 0 | 0 | 0 | 0 | 0 | 0 | 0 | 1 |
| ZNRF3 | 2 | 0 | 0 | 0 | 0 | 0 | 0 | 0 | 1 |
| ADCY6 | 2 | 0 | 0 | 0 | 0 | 0 | 0 | 0 | 1 |
| USP8 | 2 | 0 | 0 | 0 | 0 | 0 | 0 | 0 | 1 |
| SLC4A11 | 2 | 0 | 0 | 0 | 0 | 0 | 0 | 0 | 1 |
| USP43 | 2 | 0 | 0 | 0 | 0 | 0 | 0 | 0 | 1 |
| BTBD11 | 2 | 0 | 0 | 0 | 0 | 0 | 0 | 0 | 1 |
| CREBBP | 2 | 0 | 0 | 0 | 0 | 0 | 0 | 0 | 1 |
| OBSL1 | 2 | 0 | 0 | 0 | 0 | 0 | 0 | 0 | 1 |

| | | | | | | | | | |
|---|---|---|---|---|---|---|---|---|---|
| MIA2 | 2 | 0 | 0 | 0 | 0 | 0 | 0 | 0 | 1 |
| OR8B4 | 2 | 0 | 0 | 0 | 0 | 0 | 0 | 0 | 1 |
| DENND4A | 2 | 0 | 0 | 0 | 0 | 0 | 0 | 0 | 1 |
| NFXL1 | 2 | 0 | 0 | 0 | 0 | 0 | 0 | 0 | 1 |
| SV2B | 2 | 0 | 0 | 0 | 0 | 0 | 0 | 0 | 1 |
| ABCD2 | 2 | 0 | 0 | 0 | 0 | 0 | 0 | 0 | 1 |
| GUCY1A3 | 2 | 0 | 0 | 0 | 0 | 0 | 0 | 0 | 1 |
| NPAS3 | 2 | 0 | 0 | 0 | 0 | 0 | 0 | 0 | 1 |
| MAP3K1 | 0 | 2 | 0 | 0 | 0 | 0 | 0 | 0 | 1 |
| DENND2A | 2 | 0 | 0 | 0 | 0 | 0 | 0 | 0 | 1 |
| TLN1 | 2 | 0 | 0 | 0 | 0 | 0 | 0 | 0 | 1 |
| LARGE | 2 | 0 | 0 | 0 | 0 | 0 | 0 | 0 | 1 |
| ATP11B | 2 | 0 | 0 | 0 | 0 | 0 | 0 | 0 | 1 |
| GABBR1 | 2 | 0 | 0 | 0 | 0 | 0 | 0 | 0 | 1 |
| ADAM29 | 2 | 0 | 0 | 0 | 0 | 0 | 0 | 0 | 1 |
| PAK6 | 1 | 1 | 0 | 0 | 0 | 0 | 0 | 0 | 1 |
| MLL4 | 2 | 0 | 0 | 0 | 0 | 0 | 0 | 0 | 1 |
| MYO9B | 2 | 0 | 0 | 0 | 0 | 0 | 0 | 0 | 1 |
| HEATR2 | 2 | 0 | 0 | 0 | 0 | 0 | 0 | 0 | 1 |
| OR5D13 | 2 | 0 | 0 | 0 | 0 | 0 | 0 | 0 | 1 |
| YLPM1 | 2 | 0 | 0 | 0 | 0 | 0 | 0 | 0 | 1 |
| OR5J2 | 2 | 0 | 0 | 0 | 0 | 0 | 0 | 0 | 1 |
| UACA | 2 | 0 | 0 | 0 | 0 | 0 | 0 | 0 | 1 |
| INHBA | 2 | 0 | 0 | 0 | 0 | 0 | 0 | 0 | 1 |
| AK7 | 2 | 0 | 0 | 0 | 0 | 0 | 0 | 0 | 1 |
| SIK3 | 2 | 0 | 0 | 0 | 0 | 0 | 0 | 0 | 1 |
| SUSD4 | 2 | 0 | 0 | 0 | 0 | 0 | 0 | 0 | 1 |
| TNRC6B | 2 | 0 | 0 | 0 | 0 | 0 | 0 | 0 | 1 |
| LRP12 | 2 | 0 | 0 | 0 | 0 | 0 | 0 | 0 | 1 |
| ITGAE | 2 | 0 | 0 | 0 | 0 | 0 | 0 | 0 | 1 |
| RBL2 | 2 | 0 | 0 | 0 | 0 | 0 | 0 | 0 | 1 |
| ATP4A | 1 | 0 | 1 | 0 | 0 | 0 | 0 | 0 | 1 |
| MAP3K12 | 2 | 0 | 0 | 0 | 0 | 0 | 0 | 0 | 1 |
| PHRF1 | 1 | 1 | 0 | 0 | 0 | 0 | 0 | 0 | 1 |
| MAP3K6 | 2 | 0 | 0 | 0 | 0 | 0 | 0 | 0 | 1 |
| RALGAPB | 2 | 0 | 0 | 0 | 0 | 0 | 0 | 0 | 1 |
| ANXA11 | 2 | 0 | 0 | 0 | 0 | 0 | 0 | 0 | 1 |
| TTC37 | 2 | 0 | 0 | 0 | 0 | 0 | 0 | 0 | 1 |
| AASS | 2 | 0 | 0 | 0 | 0 | 0 | 0 | 0 | 1 |
| MLH3 | 1 | 1 | 0 | 0 | 0 | 0 | 0 | 0 | 1 |
| HDGFL1 | 2 | 0 | 0 | 0 | 0 | 0 | 0 | 0 | 1 |
| NRG1 | 2 | 0 | 0 | 0 | 0 | 0 | 0 | 0 | 1 |
| NACAD | 2 | 0 | 0 | 0 | 0 | 0 | 0 | 0 | 1 |
| CHD9 | 2 | 0 | 0 | 0 | 0 | 0 | 0 | 0 | 1 |
| NPC1 | 2 | 0 | 0 | 0 | 0 | 0 | 0 | 0 | 1 |
| MRE11A | 1 | 1 | 0 | 0 | 0 | 0 | 0 | 0 | 1 |

| | | | | | | | | | |
|---|---|---|---|---|---|---|---|---|---|
| APC2 | 2 | 0 | 0 | 0 | 0 | 0 | 0 | 0 | 1 |
| WDR85 | 1 | 1 | 0 | 0 | 0 | 0 | 0 | 0 | 1 |
| NLRP1 | 2 | 0 | 0 | 0 | 0 | 0 | 0 | 0 | 1 |
| ANKRD11 | 2 | 0 | 0 | 0 | 0 | 0 | 0 | 0 | 1 |
| MCF2L2 | 2 | 0 | 0 | 0 | 0 | 0 | 0 | 0 | 1 |
| CDKL1 | 1 | 0 | 1 | 0 | 0 | 0 | 0 | 0 | 1 |
| MAST1 | 2 | 0 | 0 | 0 | 0 | 0 | 0 | 0 | 1 |
| ANKAR | 2 | 0 | 0 | 0 | 0 | 0 | 0 | 0 | 1 |
| STARD8 | 1 | 1 | 0 | 0 | 0 | 0 | 0 | 0 | 1 |
| HIRA | 2 | 0 | 0 | 0 | 0 | 0 | 0 | 0 | 1 |
| PLEKHG7 | 2 | 0 | 0 | 0 | 0 | 0 | 0 | 0 | 1 |
| KDM4D | 1 | 1 | 0 | 0 | 0 | 0 | 0 | 0 | 1 |
| PRSS12 | 2 | 0 | 0 | 0 | 0 | 0 | 0 | 0 | 1 |
| ALS2 | 2 | 0 | 0 | 0 | 0 | 0 | 0 | 0 | 1 |
| ARID1A | 2 | 0 | 0 | 0 | 0 | 0 | 0 | 0 | 1 |
| WDR75 | 2 | 0 | 0 | 0 | 0 | 0 | 0 | 0 | 1 |
| SAP130 | 2 | 0 | 0 | 0 | 0 | 0 | 0 | 0 | 1 |
| CTNNA3 | 2 | 0 | 0 | 0 | 0 | 0 | 0 | 0 | 1 |
| JAG1 | 2 | 0 | 0 | 0 | 0 | 0 | 0 | 0 | 1 |
| TENC1 | 2 | 0 | 0 | 0 | 0 | 0 | 0 | 0 | 1 |
| SYMPK | 2 | 0 | 0 | 0 | 0 | 0 | 0 | 0 | 1 |
| LAMB2 | 2 | 0 | 0 | 0 | 0 | 0 | 0 | 0 | 1 |
| KAT2B | 2 | 0 | 0 | 0 | 0 | 0 | 0 | 0 | 1 |
| NACA | 2 | 0 | 0 | 0 | 0 | 0 | 0 | 0 | 1 |
| PTPRU | 2 | 0 | 0 | 0 | 0 | 0 | 0 | 0 | 1 |
| BPTF | 2 | 0 | 0 | 0 | 0 | 0 | 0 | 0 | 1 |
| CARD6 | 1 | 1 | 0 | 0 | 0 | 0 | 0 | 0 | 1 |
| FCN2 | 2 | 0 | 0 | 0 | 0 | 0 | 0 | 0 | 1 |
| ENGASE | 2 | 0 | 0 | 0 | 0 | 0 | 0 | 0 | 1 |
| LYSMD1 | 2 | 0 | 0 | 0 | 0 | 0 | 0 | 0 | 1 |
| KLHDC7A | 1 | 1 | 0 | 0 | 0 | 0 | 0 | 0 | 1 |
| SPTBN5 | 2 | 0 | 0 | 0 | 0 | 0 | 0 | 0 | 1 |
| NLGN3 | 2 | 0 | 0 | 0 | 0 | 0 | 0 | 0 | 1 |
| AGXT2 | 1 | 1 | 0 | 0 | 0 | 0 | 0 | 0 | 1 |
| ABCD3 | 2 | 0 | 0 | 0 | 0 | 0 | 0 | 0 | 1 |
| CEP192 | 2 | 0 | 0 | 0 | 0 | 0 | 0 | 0 | 1 |
| CYP4F2 | 2 | 0 | 0 | 0 | 0 | 0 | 0 | 0 | 1 |
| TANC2 | 1 | 1 | 0 | 0 | 0 | 0 | 0 | 0 | 1 |
| YEATS2 | 2 | 0 | 0 | 0 | 0 | 0 | 0 | 0 | 1 |
| PGK2 | 2 | 0 | 0 | 0 | 0 | 0 | 0 | 0 | 1 |
| SMARCA1 | 2 | 0 | 0 | 0 | 0 | 0 | 0 | 0 | 1 |
| SLC45A4 | 1 | 1 | 0 | 0 | 0 | 0 | 0 | 0 | 1 |
| KIF26A | 2 | 0 | 0 | 0 | 0 | 0 | 0 | 0 | 1 |
| SIPA1L2 | 1 | 1 | 0 | 0 | 0 | 0 | 0 | 0 | 1 |
| WDR35 | 2 | 0 | 0 | 0 | 0 | 0 | 0 | 0 | 1 |
| CIT | 2 | 0 | 0 | 0 | 0 | 0 | 0 | 0 | 1 |

| | | | | | | | | | |
|---|---|---|---|---|---|---|---|---|---|
| MYH14 | 2 | 0 | 0 | 0 | 0 | 0 | 0 | 0 | 1 |
| RASEF | 1 | 0 | 0 | 0 | 1 | 0 | 0 | 0 | 1 |
| PLA2R1 | 2 | 0 | 0 | 0 | 0 | 0 | 0 | 0 | 1 |
| GCFC1 | 2 | 0 | 0 | 0 | 0 | 0 | 0 | 0 | 1 |
| SH3TC1 | 2 | 0 | 0 | 0 | 0 | 0 | 0 | 0 | 1 |
| TBC1D9B | 2 | 0 | 0 | 0 | 0 | 0 | 0 | 0 | 1 |
| TANC1 | 1 | 1 | 0 | 0 | 0 | 0 | 0 | 0 | 1 |
| SMC4 | 2 | 0 | 0 | 0 | 0 | 0 | 0 | 0 | 1 |
| MYO1B | 2 | 0 | 0 | 0 | 0 | 0 | 0 | 0 | 1 |
| OR2K2 | 2 | 0 | 0 | 0 | 0 | 0 | 0 | 0 | 1 |
| CNKSR3 | 2 | 0 | 0 | 0 | 0 | 0 | 0 | 0 | 1 |
| BTAF1 | 1 | 0 | 1 | 0 | 0 | 0 | 0 | 0 | 1 |
| NARF | 2 | 0 | 0 | 0 | 0 | 0 | 0 | 0 | 1 |
| AIM1 | 2 | 0 | 0 | 0 | 0 | 0 | 0 | 0 | 1 |
| MCM3AP | 2 | 0 | 0 | 0 | 0 | 0 | 0 | 0 | 1 |
| NCAPG | 2 | 0 | 0 | 0 | 0 | 0 | 0 | 0 | 1 |
| OR1C1 | 2 | 0 | 0 | 0 | 0 | 0 | 0 | 0 | 1 |
| CACNA1I | 2 | 0 | 0 | 0 | 0 | 0 | 0 | 0 | 1 |
| FBXO11 | 1 | 1 | 0 | 0 | 0 | 0 | 0 | 0 | 1 |
| SLC19A1 | 2 | 0 | 0 | 0 | 0 | 0 | 0 | 0 | 1 |
| HERC3 | 2 | 0 | 0 | 0 | 0 | 0 | 0 | 0 | 1 |
| DGKZ | 2 | 0 | 0 | 0 | 0 | 0 | 0 | 0 | 1 |
| TIAM1 | 2 | 0 | 0 | 0 | 0 | 0 | 0 | 0 | 1 |
| FRYL | 2 | 0 | 0 | 0 | 0 | 0 | 0 | 0 | 1 |
| NIN | 1 | 1 | 0 | 0 | 0 | 0 | 0 | 0 | 1 |
| AHNAK2 | 2 | 0 | 0 | 0 | 0 | 0 | 0 | 0 | 1 |
| GEMIN5 | 2 | 0 | 0 | 0 | 0 | 0 | 0 | 0 | 1 |
| PKD1 | 2 | 0 | 0 | 0 | 0 | 0 | 0 | 0 | 1 |
| PPRC1 | 2 | 0 | 0 | 0 | 0 | 0 | 0 | 0 | 1 |
| CTBP2 | 2 | 0 | 0 | 0 | 0 | 0 | 0 | 0 | 1 |
| GPR123 | 2 | 0 | 0 | 0 | 0 | 0 | 0 | 0 | 1 |
| TECPR1 | 2 | 0 | 0 | 0 | 0 | 0 | 0 | 0 | 1 |
| PCNXL2 | 2 | 0 | 0 | 0 | 0 | 0 | 0 | 0 | 1 |
| SYNM | 2 | 0 | 0 | 0 | 0 | 0 | 0 | 0 | 1 |
| IQSEC2 | 2 | 0 | 0 | 0 | 0 | 0 | 0 | 0 | 1 |
| PGAM5 | 1 | 0 | 1 | 0 | 0 | 0 | 0 | 0 | 1 |
| ST6GALNAC3 | 2 | 0 | 0 | 0 | 0 | 0 | 0 | 0 | 1 |
| GOLPH3 | 2 | 0 | 0 | 0 | 0 | 0 | 0 | 0 | 1 |
| ZNF295 | 1 | 1 | 0 | 0 | 0 | 0 | 0 | 0 | 1 |
| ZBTB20 | 2 | 0 | 0 | 0 | 0 | 0 | 0 | 0 | 1 |
| PTPRZ1 | 2 | 0 | 0 | 0 | 0 | 0 | 0 | 0 | 1 |
| SHROOM2 | 2 | 0 | 0 | 0 | 0 | 0 | 0 | 0 | 1 |
| PAN3 | 2 | 0 | 0 | 0 | 0 | 0 | 0 | 0 | 1 |
| KIAA0196 | 2 | 0 | 0 | 0 | 0 | 0 | 0 | 0 | 1 |
| FGD5 | 2 | 0 | 0 | 0 | 0 | 0 | 0 | 0 | 1 |
| CLCA4 | 2 | 0 | 0 | 0 | 0 | 0 | 0 | 0 | 1 |

| | | | | | | | | | |
|---|---|---|---|---|---|---|---|---|---|
| PKNOX2 | 2 | 0 | 0 | 0 | 0 | 0 | 0 | 0 | 1 |
| LRCH1 | 2 | 0 | 0 | 0 | 0 | 0 | 0 | 0 | 1 |
| DRD2 | 1 | 1 | 0 | 0 | 0 | 0 | 0 | 0 | 1 |
| FAM120A | 2 | 0 | 0 | 0 | 0 | 0 | 0 | 0 | 1 |
| NF1 | 2 | 0 | 0 | 0 | 0 | 0 | 0 | 0 | 1 |
| DHX29 | 2 | 0 | 0 | 0 | 0 | 0 | 0 | 0 | 1 |
| TSPAN17 | 2 | 0 | 0 | 0 | 0 | 0 | 0 | 0 | 1 |
| PKN2 | 2 | 0 | 0 | 0 | 0 | 0 | 0 | 0 | 1 |
| INTS8 | 2 | 0 | 0 | 0 | 0 | 0 | 0 | 0 | 1 |
| BIRC2 | 2 | 0 | 0 | 0 | 0 | 0 | 0 | 0 | 1 |
| ATRNL1 | 2 | 0 | 0 | 0 | 0 | 0 | 0 | 0 | 1 |
| THBS1 | 2 | 0 | 0 | 0 | 0 | 0 | 0 | 0 | 1 |
| UTP20 | 1 | 0 | 1 | 0 | 0 | 0 | 0 | 0 | 1 |
| KIAA0913 | 2 | 0 | 0 | 0 | 0 | 0 | 0 | 0 | 1 |
| ALDOC | 2 | 0 | 0 | 0 | 0 | 0 | 0 | 0 | 1 |
| XPO7 | 2 | 0 | 0 | 0 | 0 | 0 | 0 | 0 | 1 |
| KIAA1429 | 2 | 0 | 0 | 0 | 0 | 0 | 0 | 0 | 1 |
| PDPR | 2 | 0 | 0 | 0 | 0 | 0 | 0 | 0 | 1 |
| ARNTL2 | 1 | 0 | 1 | 0 | 0 | 0 | 0 | 0 | 1 |
| OR5M10 | 2 | 0 | 0 | 0 | 0 | 0 | 0 | 0 | 1 |
| ARL8B | 2 | 0 | 0 | 0 | 0 | 0 | 0 | 0 | 1 |
| ZPLD1 | 2 | 0 | 0 | 0 | 0 | 0 | 0 | 0 | 1 |
| DDX54 | 2 | 0 | 0 | 0 | 0 | 0 | 0 | 0 | 1 |
| C3orf22 | 2 | 0 | 0 | 0 | 0 | 0 | 0 | 0 | 1 |
| TMEM57 | 2 | 0 | 0 | 0 | 0 | 0 | 0 | 0 | 1 |
| OR4N5 | 2 | 0 | 0 | 0 | 0 | 0 | 0 | 0 | 1 |
| SMR3B | 2 | 0 | 0 | 0 | 0 | 0 | 0 | 0 | 1 |
| RORB | 2 | 0 | 0 | 0 | 0 | 0 | 0 | 0 | 1 |
| FAM81B | 2 | 0 | 0 | 0 | 0 | 0 | 0 | 0 | 1 |
| APCS | 1 | 1 | 0 | 0 | 0 | 0 | 0 | 0 | 1 |
| TIMP2 | 2 | 0 | 0 | 0 | 0 | 0 | 0 | 0 | 1 |
| CDH12 | 2 | 0 | 0 | 0 | 0 | 0 | 0 | 0 | 1 |
| PLCD4 | 2 | 0 | 0 | 0 | 0 | 0 | 0 | 0 | 1 |
| JAKMIP1 | 2 | 0 | 0 | 0 | 0 | 0 | 0 | 0 | 1 |
| CMKLR1 | 2 | 0 | 0 | 0 | 0 | 0 | 0 | 0 | 1 |
| AHRR | 2 | 0 | 0 | 0 | 0 | 0 | 0 | 0 | 1 |
| SBSN | 2 | 0 | 0 | 0 | 0 | 0 | 0 | 0 | 1 |
| PTCHD2 | 2 | 0 | 0 | 0 | 0 | 0 | 0 | 0 | 1 |
| Sep-15 | 2 | 0 | 0 | 0 | 0 | 0 | 0 | 0 | 1 |
| LENEP | 0 | 1 | 0 | 0 | 0 | 0 | 0 | 0 | 1 |
| ELOF1 | 0 | 1 | 0 | 0 | 0 | 0 | 0 | 0 | 1 |
| SCGB1D4 | 0 | 1 | 0 | 0 | 0 | 0 | 0 | 0 | 1 |
| ECSCR | 0 | 1 | 0 | 0 | 0 | 0 | 0 | 0 | 1 |
| C15orf63 | 0 | 1 | 0 | 0 | 0 | 0 | 0 | 0 | 1 |
| NDUFS5 | 0 | 1 | 0 | 0 | 0 | 0 | 0 | 0 | 1 |
| SPRR1B | 0 | 1 | 0 | 0 | 0 | 0 | 0 | 0 | 1 |

| | | | | | | | | | |
|---|---|---|---|---|---|---|---|---|---|
| CSTA | 0 | 1 | 0 | 0 | 0 | 0 | 0 | 0 | 1 |
| KRTAP3-2 | 0 | 1 | 0 | 0 | 0 | 0 | 0 | 0 | 1 |
| ORMDL2 | 0 | 1 | 0 | 0 | 0 | 0 | 0 | 0 | 1 |
| KLRC4 | 0 | 1 | 0 | 0 | 0 | 0 | 0 | 0 | 1 |
| HIST1H4B | 0 | 1 | 0 | 0 | 0 | 0 | 0 | 0 | 1 |
| SFTA3 | 0 | 0 | 1 | 0 | 0 | 0 | 0 | 0 | 1 |
| C7orf69 | 0 | 1 | 0 | 0 | 0 | 0 | 0 | 0 | 1 |
| C1orf144 | 0 | 1 | 0 | 0 | 0 | 0 | 0 | 0 | 1 |
| ARL8A | 0 | 1 | 0 | 0 | 0 | 0 | 0 | 0 | 1 |
| CXCL13 | 0 | 1 | 0 | 0 | 0 | 0 | 0 | 0 | 1 |
| NGB | 0 | 0 | 1 | 0 | 0 | 0 | 0 | 0 | 1 |
| MRPS14 | 0 | 1 | 0 | 0 | 0 | 0 | 0 | 0 | 1 |
| HIST1H2BJ | 0 | 1 | 0 | 0 | 0 | 0 | 0 | 0 | 1 |
| CLIC1 | 0 | 1 | 0 | 0 | 0 | 0 | 0 | 0 | 1 |
| OCLM | 1 | 0 | 0 | 0 | 0 | 0 | 0 | 0 | 1 |
| IQCF2 | 0 | 1 | 0 | 0 | 0 | 0 | 0 | 0 | 1 |
| IL10 | 0 | 1 | 0 | 0 | 0 | 0 | 0 | 0 | 1 |
| NDUFS6 | 0 | 0 | 1 | 0 | 0 | 0 | 0 | 0 | 1 |
| RBP2 | 0 | 0 | 1 | 0 | 0 | 0 | 0 | 0 | 1 |
| SNRPA1 | 0 | 1 | 0 | 0 | 0 | 0 | 0 | 0 | 1 |
| LYRM1 | 0 | 1 | 0 | 0 | 0 | 0 | 0 | 0 | 1 |
| MED21 | 0 | 1 | 0 | 0 | 0 | 0 | 0 | 0 | 1 |
| PF4V1 | 0 | 0 | 0 | 0 | 1 | 0 | 0 | 0 | 1 |
| TMEM80 | 0 | 0 | 1 | 0 | 0 | 0 | 0 | 0 | 1 |
| GNG8 | 1 | 0 | 0 | 0 | 0 | 0 | 0 | 0 | 1 |
| AKAP14 | 0 | 1 | 0 | 0 | 0 | 0 | 0 | 0 | 1 |
| OBFC2A | 0 | 1 | 0 | 0 | 0 | 0 | 0 | 0 | 1 |
| BUD31 | 0 | 1 | 0 | 0 | 0 | 0 | 0 | 0 | 1 |
| POLR2C | 0 | 0 | 0 | 0 | 0 | 1 | 0 | 0 | 1 |
| C14orf109 | 0 | 1 | 0 | 0 | 0 | 0 | 0 | 0 | 1 |
| NME1 | 0 | 1 | 0 | 0 | 0 | 0 | 0 | 0 | 1 |
| RNF125 | 0 | 0 | 1 | 0 | 0 | 0 | 0 | 0 | 1 |
| C8orf37 | 0 | 0 | 1 | 0 | 0 | 0 | 0 | 0 | 1 |
| HBM | 0 | 1 | 0 | 0 | 0 | 0 | 0 | 0 | 1 |
| GCG | 0 | 0 | 1 | 0 | 0 | 0 | 0 | 0 | 1 |
| TNP1 | 0 | 0 | 0 | 0 | 1 | 0 | 0 | 0 | 1 |
| CMBL | 0 | 0 | 1 | 0 | 0 | 0 | 0 | 0 | 1 |
| C12orf69 | 0 | 1 | 0 | 0 | 0 | 0 | 0 | 0 | 1 |
| DNAJC5G | 0 | 1 | 0 | 0 | 0 | 0 | 0 | 0 | 1 |
| HIST2H2AC | 0 | 1 | 0 | 0 | 0 | 0 | 0 | 0 | 1 |
| FAM174A | 0 | 0 | 1 | 0 | 0 | 0 | 0 | 0 | 1 |
| BCL2A1 | 0 | 1 | 0 | 0 | 0 | 0 | 0 | 0 | 1 |
| PLN | 1 | 0 | 0 | 0 | 0 | 0 | 0 | 0 | 1 |
| IFI30 | 0 | 1 | 0 | 0 | 0 | 0 | 0 | 0 | 1 |
| TAGLN3 | 0 | 1 | 0 | 0 | 0 | 0 | 0 | 0 | 1 |
| RTP1 | 0 | 1 | 0 | 0 | 0 | 0 | 0 | 0 | 1 |

| | | | | | | | | | |
|---|---|---|---|---|---|---|---|---|---|
| TFF3 | 0 | 0 | 0 | 0 | 1 | 0 | 0 | 0 | 1 |
| FTL | 0 | 0 | 1 | 0 | 0 | 0 | 0 | 0 | 1 |
| MT1H | 1 | 0 | 0 | 0 | 0 | 0 | 0 | 0 | 1 |
| CRYBA1 | 0 | 1 | 0 | 0 | 0 | 0 | 0 | 0 | 1 |
| C19orf59 | 0 | 1 | 0 | 0 | 0 | 0 | 0 | 0 | 1 |
| C19orf12 | 0 | 1 | 0 | 0 | 0 | 0 | 0 | 0 | 1 |
| RAB13 | 0 | 1 | 0 | 0 | 0 | 0 | 0 | 0 | 1 |
| SLC25A17 | 0 | 1 | 0 | 0 | 0 | 0 | 0 | 0 | 1 |
| ASF1B | 0 | 1 | 0 | 0 | 0 | 0 | 0 | 0 | 1 |
| STX6 | 0 | 0 | 1 | 0 | 0 | 0 | 0 | 0 | 1 |
| BARX1 | 0 | 0 | 1 | 0 | 0 | 0 | 0 | 0 | 1 |
| MRPS7 | 0 | 1 | 0 | 0 | 0 | 0 | 0 | 0 | 1 |
| TSPAN9 | 0 | 1 | 0 | 0 | 0 | 0 | 0 | 0 | 1 |
| ANAPC13 | 1 | 0 | 0 | 0 | 0 | 0 | 0 | 0 | 1 |
| TMEM116 | 0 | 1 | 0 | 0 | 0 | 0 | 0 | 0 | 1 |
| MAD2L1 | 0 | 0 | 1 | 0 | 0 | 0 | 0 | 0 | 1 |
| KRTAP22-1 | 1 | 0 | 0 | 0 | 0 | 0 | 0 | 0 | 1 |
| ULBP3 | 0 | 1 | 0 | 0 | 0 | 0 | 0 | 0 | 1 |
| GLIPR1 | 0 | 1 | 0 | 0 | 0 | 0 | 0 | 0 | 1 |
| SURF6 | 0 | 1 | 0 | 0 | 0 | 0 | 0 | 0 | 1 |
| PDE6H | 1 | 0 | 0 | 0 | 0 | 0 | 0 | 0 | 1 |
| HUS1 | 0 | 0 | 1 | 0 | 0 | 0 | 0 | 0 | 1 |
| C4orf6 | 1 | 0 | 0 | 0 | 0 | 0 | 0 | 0 | 1 |
| NCR2 | 0 | 1 | 0 | 0 | 0 | 0 | 0 | 0 | 1 |
| RFNG | 0 | 1 | 0 | 0 | 0 | 0 | 0 | 0 | 1 |
| UTP11L | 0 | 0 | 1 | 0 | 0 | 0 | 0 | 0 | 1 |
| USMG5 | 1 | 0 | 0 | 0 | 0 | 0 | 0 | 0 | 1 |
| IFI27L1 | 0 | 0 | 0 | 0 | 1 | 0 | 0 | 0 | 1 |
| PLAG1 | 0 | 0 | 0 | 0 | 0 | 0 | 1 | 0 | 1 |
| TSPAN11 | 0 | 1 | 0 | 0 | 0 | 0 | 0 | 0 | 1 |
| LCE3A | 1 | 0 | 0 | 0 | 0 | 0 | 0 | 0 | 1 |
| LRRC59 | 0 | 1 | 0 | 0 | 0 | 0 | 0 | 0 | 1 |
| C10orf113 | 0 | 1 | 0 | 0 | 0 | 0 | 0 | 0 | 1 |
| IFNA5 | 0 | 1 | 0 | 0 | 0 | 0 | 0 | 0 | 1 |
| CKS1B | 1 | 0 | 0 | 0 | 0 | 0 | 0 | 0 | 1 |
| KRTAP20-1 | 1 | 0 | 0 | 0 | 0 | 0 | 0 | 0 | 1 |
| ZNF593 | 0 | 0 | 0 | 0 | 1 | 0 | 0 | 0 | 1 |
| TIMM17A | 0 | 1 | 0 | 0 | 0 | 0 | 0 | 0 | 1 |
| ARRDC5 | 0 | 1 | 0 | 0 | 0 | 0 | 0 | 0 | 1 |
| HN1 | 0 | 1 | 0 | 0 | 0 | 0 | 0 | 0 | 1 |
| CYB5A | 0 | 0 | 0 | 0 | 1 | 0 | 0 | 0 | 1 |
| CCDC43 | 0 | 1 | 0 | 0 | 0 | 0 | 0 | 0 | 1 |
| CETN2 | 0 | 1 | 0 | 0 | 0 | 0 | 0 | 0 | 1 |
| B3GNT1 | 0 | 1 | 0 | 0 | 0 | 0 | 0 | 0 | 1 |
| PAFAH1B3 | 0 | 1 | 0 | 0 | 0 | 0 | 0 | 0 | 1 |
| JMJD8 | 0 | 0 | 0 | 1 | 0 | 0 | 0 | 0 | 1 |

| | | | | | | | | | |
|---|---|---|---|---|---|---|---|---|---|
| GLRX | 1 | 0 | 0 | 0 | 0 | 0 | 0 | 0 | 1 |
| CCL17 | 1 | 0 | 0 | 0 | 0 | 0 | 0 | 0 | 1 |
| ASB7 | 0 | 1 | 0 | 0 | 0 | 0 | 0 | 0 | 1 |
| AHSA2 | 1 | 0 | 0 | 0 | 0 | 0 | 0 | 0 | 1 |
| SLC25A29 | 0 | 1 | 0 | 0 | 0 | 0 | 0 | 0 | 1 |
| TMEM51 | 0 | 1 | 0 | 0 | 0 | 0 | 0 | 0 | 1 |
| PPPDE1 | 0 | 1 | 0 | 0 | 0 | 0 | 0 | 0 | 1 |
| NAPG | 0 | 0 | 1 | 0 | 0 | 0 | 0 | 0 | 1 |
| ZNF200 | 0 | 1 | 0 | 0 | 0 | 0 | 0 | 0 | 1 |
| DNAJC19 | 1 | 0 | 0 | 0 | 0 | 0 | 0 | 0 | 1 |
| TESC | 0 | 1 | 0 | 0 | 0 | 0 | 0 | 0 | 1 |
| TMEM176A | 0 | 1 | 0 | 0 | 0 | 0 | 0 | 0 | 1 |
| SIRT5 | 0 | 0 | 1 | 0 | 0 | 0 | 0 | 0 | 1 |
| CPLX1 | 1 | 0 | 0 | 0 | 0 | 0 | 0 | 0 | 1 |
| ZCCHC13 | 0 | 0 | 0 | 0 | 1 | 0 | 0 | 0 | 1 |
| SLC25A26 | 0 | 1 | 0 | 0 | 0 | 0 | 0 | 0 | 1 |
| DCTN2 | 0 | 1 | 0 | 0 | 0 | 0 | 0 | 0 | 1 |
| BZW1 | 0 | 1 | 0 | 0 | 0 | 0 | 0 | 0 | 1 |
| RPL30 | 1 | 0 | 0 | 0 | 0 | 0 | 0 | 0 | 1 |
| TMEM190 | 0 | 1 | 0 | 0 | 0 | 0 | 0 | 0 | 1 |
| IMPA2 | 0 | 0 | 1 | 0 | 0 | 0 | 0 | 0 | 1 |
| MRPS21 | 1 | 0 | 0 | 0 | 0 | 0 | 0 | 0 | 1 |
| RAB5C | 0 | 1 | 0 | 0 | 0 | 0 | 0 | 0 | 1 |
| MGST3 | 1 | 0 | 0 | 0 | 0 | 0 | 0 | 0 | 1 |
| PCBP2 | 0 | 0 | 1 | 0 | 0 | 0 | 0 | 0 | 1 |
| FAM32A | 1 | 0 | 0 | 0 | 0 | 0 | 0 | 0 | 1 |
| VASH2 | 0 | 1 | 0 | 0 | 0 | 0 | 0 | 0 | 1 |
| HIST1H4D | 1 | 0 | 0 | 0 | 0 | 0 | 0 | 0 | 1 |
| SCGB1D1 | 1 | 0 | 0 | 0 | 0 | 0 | 0 | 0 | 1 |
| DEFB134 | 1 | 0 | 0 | 0 | 0 | 0 | 0 | 0 | 1 |
| TAAR1 | 0 | 1 | 0 | 0 | 0 | 0 | 0 | 0 | 1 |
| PTEN | 0 | 0 | 0 | 0 | 0 | 1 | 0 | 0 | 1 |
| KRTAP7-1 | 1 | 0 | 0 | 0 | 0 | 0 | 0 | 0 | 1 |
| SELK | 1 | 0 | 0 | 0 | 0 | 0 | 0 | 0 | 1 |
| EFNB3 | 0 | 1 | 0 | 0 | 0 | 0 | 0 | 0 | 1 |
| TMEM150B | 0 | 1 | 0 | 0 | 0 | 0 | 0 | 0 | 1 |
| KIAA1045 | 0 | 1 | 0 | 0 | 0 | 0 | 0 | 0 | 1 |
| MAPK11 | 0 | 1 | 0 | 0 | 0 | 0 | 0 | 0 | 1 |
| IFNGR2 | 0 | 1 | 0 | 0 | 0 | 0 | 0 | 0 | 1 |
| EIF5A2 | 1 | 0 | 0 | 0 | 0 | 0 | 0 | 0 | 1 |
| GNRH2 | 1 | 0 | 0 | 0 | 0 | 0 | 0 | 0 | 1 |
| RPP40 | 0 | 1 | 0 | 0 | 0 | 0 | 0 | 0 | 1 |
| PDZK1IP1 | 1 | 0 | 0 | 0 | 0 | 0 | 0 | 0 | 1 |
| POLR2H | 1 | 0 | 0 | 0 | 0 | 0 | 0 | 0 | 1 |
| CCS | 0 | 0 | 1 | 0 | 0 | 0 | 0 | 0 | 1 |
| GLIPR1L1 | 0 | 0 | 1 | 0 | 0 | 0 | 0 | 0 | 1 |

| | | | | | | | | | |
|---|---|---|---|---|---|---|---|---|---|
| C17orf61 | 1 | 0 | 0 | 0 | 0 | 0 | 0 | 0 | 1 |
| MRGPRX1 | 0 | 1 | 0 | 0 | 0 | 0 | 0 | 0 | 1 |
| C8orf83 | 1 | 0 | 0 | 0 | 0 | 0 | 0 | 0 | 1 |
| FAM50A | 0 | 1 | 0 | 0 | 0 | 0 | 0 | 0 | 1 |
| HLA-B | 0 | 1 | 0 | 0 | 0 | 0 | 0 | 0 | 1 |
| HS1BP3 | 0 | 0 | 0 | 0 | 0 | 1 | 0 | 0 | 1 |
| RPL36A | 1 | 0 | 0 | 0 | 0 | 0 | 0 | 0 | 1 |
| MORN2 | 1 | 0 | 0 | 0 | 0 | 0 | 0 | 0 | 1 |
| PBOV1 | 1 | 0 | 0 | 0 | 0 | 0 | 0 | 0 | 1 |
| FXC1 | 1 | 0 | 0 | 0 | 0 | 0 | 0 | 0 | 1 |
| TXNDC17 | 1 | 0 | 0 | 0 | 0 | 0 | 0 | 0 | 1 |
| VAMP5 | 1 | 0 | 0 | 0 | 0 | 0 | 0 | 0 | 1 |
| UBXN1 | 0 | 0 | 1 | 0 | 0 | 0 | 0 | 0 | 1 |
| YIF1B | 0 | 1 | 0 | 0 | 0 | 0 | 0 | 0 | 1 |
| MAD2L1BP | 0 | 1 | 0 | 0 | 0 | 0 | 0 | 0 | 1 |
| MAS1 | 0 | 1 | 0 | 0 | 0 | 0 | 0 | 0 | 1 |
| APOC4 | 1 | 0 | 0 | 0 | 0 | 0 | 0 | 0 | 1 |
| SPINK9 | 1 | 0 | 0 | 0 | 0 | 0 | 0 | 0 | 1 |
| LYPD6 | 0 | 0 | 0 | 0 | 1 | 0 | 0 | 0 | 1 |
| DEDD | 0 | 1 | 0 | 0 | 0 | 0 | 0 | 0 | 1 |
| EMID1 | 0 | 0 | 1 | 0 | 0 | 0 | 0 | 0 | 1 |
| UBAC2 | 0 | 1 | 0 | 0 | 0 | 0 | 0 | 0 | 1 |
| SS18L2 | 1 | 0 | 0 | 0 | 0 | 0 | 0 | 0 | 1 |
| HSD11B1 | 0 | 1 | 0 | 0 | 0 | 0 | 0 | 0 | 1 |
| ZNF597 | 0 | 1 | 0 | 0 | 0 | 0 | 0 | 0 | 1 |
| PPP1R1C | 1 | 0 | 0 | 0 | 0 | 0 | 0 | 0 | 1 |
| CXorf61 | 1 | 0 | 0 | 0 | 0 | 0 | 0 | 0 | 1 |
| HIST4H4 | 1 | 0 | 0 | 0 | 0 | 0 | 0 | 0 | 1 |
| CALM3 | 1 | 0 | 0 | 0 | 0 | 0 | 0 | 0 | 1 |
| SSU72 | 1 | 0 | 0 | 0 | 0 | 0 | 0 | 0 | 1 |
| TRIP13 | 0 | 1 | 0 | 0 | 0 | 0 | 0 | 0 | 1 |
| MGAT1 | 0 | 1 | 0 | 0 | 0 | 0 | 0 | 0 | 1 |
| LSM7 | 1 | 0 | 0 | 0 | 0 | 0 | 0 | 0 | 1 |
| PEX3 | 0 | 1 | 0 | 0 | 0 | 0 | 0 | 0 | 1 |
| FBXO48 | 1 | 0 | 0 | 0 | 0 | 0 | 0 | 0 | 1 |
| CRYM | 0 | 1 | 0 | 0 | 0 | 0 | 0 | 0 | 1 |
| GNGT2 | 1 | 0 | 0 | 0 | 0 | 0 | 0 | 0 | 1 |
| XCR1 | 0 | 1 | 0 | 0 | 0 | 0 | 0 | 0 | 1 |
| BMI1 | 0 | 1 | 0 | 0 | 0 | 0 | 0 | 0 | 1 |
| MRPL45 | 0 | 1 | 0 | 0 | 0 | 0 | 0 | 0 | 1 |
| TMPRSS11D | 0 | 1 | 0 | 0 | 0 | 0 | 0 | 0 | 1 |
| MKX | 0 | 0 | 1 | 0 | 0 | 0 | 0 | 0 | 1 |
| GNRH1 | 1 | 0 | 0 | 0 | 0 | 0 | 0 | 0 | 1 |
| SPRR2G | 1 | 0 | 0 | 0 | 0 | 0 | 0 | 0 | 1 |
| DEFA6 | 1 | 0 | 0 | 0 | 0 | 0 | 0 | 0 | 1 |
| DYX1C1 | 0 | 1 | 0 | 0 | 0 | 0 | 0 | 0 | 1 |

| | | | | | | | | | |
|---|---|---|---|---|---|---|---|---|---|
| DHFR | 1 | 0 | 0 | 0 | 0 | 0 | 0 | 0 | 1 |
| DHRS13 | 0 | 1 | 0 | 0 | 0 | 0 | 0 | 0 | 1 |
| HMGB3 | 1 | 0 | 0 | 0 | 0 | 0 | 0 | 0 | 1 |
| UBL3 | 1 | 0 | 0 | 0 | 0 | 0 | 0 | 0 | 1 |
| IL5 | 1 | 0 | 0 | 0 | 0 | 0 | 0 | 0 | 1 |
| SH2D1B | 1 | 0 | 0 | 0 | 0 | 0 | 0 | 0 | 1 |
| RPS27 | 1 | 0 | 0 | 0 | 0 | 0 | 0 | 0 | 1 |
| NPFF | 1 | 0 | 0 | 0 | 0 | 0 | 0 | 0 | 1 |
| LCMT1 | 0 | 0 | 1 | 0 | 0 | 0 | 0 | 0 | 1 |
| PRSS54 | 0 | 0 | 1 | 0 | 0 | 0 | 0 | 0 | 1 |
| WFDC9 | 1 | 0 | 0 | 0 | 0 | 0 | 0 | 0 | 1 |
| C3orf78 | 1 | 0 | 0 | 0 | 0 | 0 | 0 | 0 | 1 |
| LPL | 0 | 1 | 0 | 0 | 0 | 0 | 0 | 0 | 1 |
| C2orf54 | 0 | 1 | 0 | 0 | 0 | 0 | 0 | 0 | 1 |
| C17orf79 | 1 | 0 | 0 | 0 | 0 | 0 | 0 | 0 | 1 |
| PRM3 | 1 | 0 | 0 | 0 | 0 | 0 | 0 | 0 | 1 |
| SCGB1A1 | 1 | 0 | 0 | 0 | 0 | 0 | 0 | 0 | 1 |
| C9orf153 | 1 | 0 | 0 | 0 | 0 | 0 | 0 | 0 | 1 |
| CCT4 | 0 | 0 | 1 | 0 | 0 | 0 | 0 | 0 | 1 |
| CLEC2B | 1 | 0 | 0 | 0 | 0 | 0 | 0 | 0 | 1 |
| KIAA1191 | 0 | 1 | 0 | 0 | 0 | 0 | 0 | 0 | 1 |
| OPALIN | 1 | 0 | 0 | 0 | 0 | 0 | 0 | 0 | 1 |
| PFDN6 | 1 | 0 | 0 | 0 | 0 | 0 | 0 | 0 | 1 |
| RPL10A | 1 | 0 | 0 | 0 | 0 | 0 | 0 | 0 | 1 |
| HBA2 | 1 | 0 | 0 | 0 | 0 | 0 | 0 | 0 | 1 |
| BLID | 1 | 0 | 0 | 0 | 0 | 0 | 0 | 0 | 1 |
| WDFY2 | 0 | 1 | 0 | 0 | 0 | 0 | 0 | 0 | 1 |
| HTRA2 | 0 | 1 | 0 | 0 | 0 | 0 | 0 | 0 | 1 |
| ERH | 1 | 0 | 0 | 0 | 0 | 0 | 0 | 0 | 1 |
| LY6G6C | 1 | 0 | 0 | 0 | 0 | 0 | 0 | 0 | 1 |
| TTLL13 | 0 | 1 | 0 | 0 | 0 | 0 | 0 | 0 | 1 |
| P2RX3 | 0 | 1 | 0 | 0 | 0 | 0 | 0 | 0 | 1 |
| NUP50 | 0 | 0 | 1 | 0 | 0 | 0 | 0 | 0 | 1 |
| LYSMD3 | 0 | 1 | 0 | 0 | 0 | 0 | 0 | 0 | 1 |
| RAD51C | 0 | 1 | 0 | 0 | 0 | 0 | 0 | 0 | 1 |
| MEIG1 | 1 | 0 | 0 | 0 | 0 | 0 | 0 | 0 | 1 |
| CD99L2 | 0 | 1 | 0 | 0 | 0 | 0 | 0 | 0 | 1 |
| SLC44A4 | 0 | 1 | 0 | 0 | 0 | 0 | 0 | 0 | 1 |
| FAM175B | 0 | 1 | 0 | 0 | 0 | 0 | 0 | 0 | 1 |
| VSIG1 | 0 | 0 | 1 | 0 | 0 | 0 | 0 | 0 | 1 |
| ZNF781 | 0 | 1 | 0 | 0 | 0 | 0 | 0 | 0 | 1 |
| TYROBP | 1 | 0 | 0 | 0 | 0 | 0 | 0 | 0 | 1 |
| MED6 | 0 | 1 | 0 | 0 | 0 | 0 | 0 | 0 | 1 |
| CER1 | 0 | 1 | 0 | 0 | 0 | 0 | 0 | 0 | 1 |
| PAIP2 | 1 | 0 | 0 | 0 | 0 | 0 | 0 | 0 | 1 |
| NDUFB10 | 1 | 0 | 0 | 0 | 0 | 0 | 0 | 0 | 1 |

| | | | | | | | | | |
|---|---|---|---|---|---|---|---|---|---|
| PTGES | 1 | 0 | 0 | 0 | 0 | 0 | 0 | 0 | 1 |
| ARPC3 | 1 | 0 | 0 | 0 | 0 | 0 | 0 | 0 | 1 |
| PATE4 | 1 | 0 | 0 | 0 | 0 | 0 | 0 | 0 | 1 |
| DDX47 | 0 | 1 | 0 | 0 | 0 | 0 | 0 | 0 | 1 |
| MYL9 | 1 | 0 | 0 | 0 | 0 | 0 | 0 | 0 | 1 |
| RETNLB | 1 | 0 | 0 | 0 | 0 | 0 | 0 | 0 | 1 |
| IMPA1 | 0 | 0 | 1 | 0 | 0 | 0 | 0 | 0 | 1 |
| BVES | 0 | 1 | 0 | 0 | 0 | 0 | 0 | 0 | 1 |
| LOC653486 | 1 | 0 | 0 | 0 | 0 | 0 | 0 | 0 | 1 |
| AVEN | 0 | 1 | 0 | 0 | 0 | 0 | 0 | 0 | 1 |
| CDK15 | 0 | 1 | 0 | 0 | 0 | 0 | 0 | 0 | 1 |
| EN2 | 0 | 1 | 0 | 0 | 0 | 0 | 0 | 0 | 1 |
| HOXD13 | 0 | 0 | 1 | 0 | 0 | 0 | 0 | 0 | 1 |
| CASP9 | 0 | 0 | 1 | 0 | 0 | 0 | 0 | 0 | 1 |
| WFDC10B | 1 | 0 | 0 | 0 | 0 | 0 | 0 | 0 | 1 |
| MAFK | 1 | 0 | 0 | 0 | 0 | 0 | 0 | 0 | 1 |
| CLPS | 1 | 0 | 0 | 0 | 0 | 0 | 0 | 0 | 1 |
| UBE2G1 | 1 | 0 | 0 | 0 | 0 | 0 | 0 | 0 | 1 |
| PNMAL1 | 0 | 1 | 0 | 0 | 0 | 0 | 0 | 0 | 1 |
| PDZD11 | 1 | 0 | 0 | 0 | 0 | 0 | 0 | 0 | 1 |
| NODAL | 0 | 1 | 0 | 0 | 0 | 0 | 0 | 0 | 1 |
| BLVRB | 0 | 0 | 0 | 0 | 1 | 0 | 0 | 0 | 1 |
| ZDHHC16 | 0 | 1 | 0 | 0 | 0 | 0 | 0 | 0 | 1 |
| IGLL5 | 0 | 0 | 0 | 0 | 1 | 0 | 0 | 0 | 1 |
| CFL2 | 1 | 0 | 0 | 0 | 0 | 0 | 0 | 0 | 1 |
| GADD45B | 1 | 0 | 0 | 0 | 0 | 0 | 0 | 0 | 1 |
| NUTF2 | 1 | 0 | 0 | 0 | 0 | 0 | 0 | 0 | 1 |
| LY86 | 1 | 0 | 0 | 0 | 0 | 0 | 0 | 0 | 1 |
| POP5 | 1 | 0 | 0 | 0 | 0 | 0 | 0 | 0 | 1 |
| TMEM55A | 0 | 1 | 0 | 0 | 0 | 0 | 0 | 0 | 1 |
| CLLU1 | 1 | 0 | 0 | 0 | 0 | 0 | 0 | 0 | 1 |
| HIST1H2AM | 1 | 0 | 0 | 0 | 0 | 0 | 0 | 0 | 1 |
| SCRG1 | 1 | 0 | 0 | 0 | 0 | 0 | 0 | 0 | 1 |
| SNAPIN | 1 | 0 | 0 | 0 | 0 | 0 | 0 | 0 | 1 |
| AMDHD1 | 0 | 1 | 0 | 0 | 0 | 0 | 0 | 0 | 1 |
| NIF3L1 | 0 | 1 | 0 | 0 | 0 | 0 | 0 | 0 | 1 |
| C10orf131 | 1 | 0 | 0 | 0 | 0 | 0 | 0 | 0 | 1 |
| CHCHD5 | 1 | 0 | 0 | 0 | 0 | 0 | 0 | 0 | 1 |
| HIST1H4G | 1 | 0 | 0 | 0 | 0 | 0 | 0 | 0 | 1 |
| SPRED3 | 0 | 1 | 0 | 0 | 0 | 0 | 0 | 0 | 1 |
| PLEKHA3 | 0 | 1 | 0 | 0 | 0 | 0 | 0 | 0 | 1 |
| QDPR | 0 | 1 | 0 | 0 | 0 | 0 | 0 | 0 | 1 |
| TMEM14B | 1 | 0 | 0 | 0 | 0 | 0 | 0 | 0 | 1 |
| OR52H1 | 0 | 1 | 0 | 0 | 0 | 0 | 0 | 0 | 1 |
| DUSP14 | 1 | 0 | 0 | 0 | 0 | 0 | 0 | 0 | 1 |
| C1orf124 | 0 | 0 | 1 | 0 | 0 | 0 | 0 | 0 | 1 |

| | | | | | | | | | |
|---|---|---|---|---|---|---|---|---|---|
| BRI3 | 1 | 0 | 0 | 0 | 0 | 0 | 0 | 0 | 1 |
| MRPL11 | 1 | 0 | 0 | 0 | 0 | 0 | 0 | 0 | 1 |
| PARS2 | 0 | 0 | 0 | 0 | 0 | 1 | 0 | 0 | 1 |
| CXCL5 | 1 | 0 | 0 | 0 | 0 | 0 | 0 | 0 | 1 |
| INPP5K | 0 | 1 | 0 | 0 | 0 | 0 | 0 | 0 | 1 |
| HIST1H4L | 1 | 0 | 0 | 0 | 0 | 0 | 0 | 0 | 1 |
| C5orf46 | 1 | 0 | 0 | 0 | 0 | 0 | 0 | 0 | 1 |
| S100A8 | 1 | 0 | 0 | 0 | 0 | 0 | 0 | 0 | 1 |
| CLEC14A | 0 | 1 | 0 | 0 | 0 | 0 | 0 | 0 | 1 |
| C22orf40 | 1 | 0 | 0 | 0 | 0 | 0 | 0 | 0 | 1 |
| STX17 | 0 | 1 | 0 | 0 | 0 | 0 | 0 | 0 | 1 |
| GOLGA7 | 1 | 0 | 0 | 0 | 0 | 0 | 0 | 0 | 1 |
| PHYHD1 | 0 | 1 | 0 | 0 | 0 | 0 | 0 | 0 | 1 |
| C1orf162 | 1 | 0 | 0 | 0 | 0 | 0 | 0 | 0 | 1 |
| C8orf31 | 1 | 0 | 0 | 0 | 0 | 0 | 0 | 0 | 1 |
| STOML2 | 0 | 1 | 0 | 0 | 0 | 0 | 0 | 0 | 1 |
| SPINT3 | 1 | 0 | 0 | 0 | 0 | 0 | 0 | 0 | 1 |
| RCVRN | 1 | 0 | 0 | 0 | 0 | 0 | 0 | 0 | 1 |
| CYP1B1 | 0 | 1 | 0 | 0 | 0 | 0 | 0 | 0 | 1 |
| GKN2 | 1 | 0 | 0 | 0 | 0 | 0 | 0 | 0 | 1 |
| ECHDC2 | 1 | 0 | 0 | 0 | 0 | 0 | 0 | 0 | 1 |
| SRCRB4D | 0 | 1 | 0 | 0 | 0 | 0 | 0 | 0 | 1 |
| C1orf182 | 1 | 0 | 0 | 0 | 0 | 0 | 0 | 0 | 1 |
| FKBP11 | 1 | 0 | 0 | 0 | 0 | 0 | 0 | 0 | 1 |
| FBXO6 | 1 | 0 | 0 | 0 | 0 | 0 | 0 | 0 | 1 |
| DNAL4 | 1 | 0 | 0 | 0 | 0 | 0 | 0 | 0 | 1 |
| CALML6 | 1 | 0 | 0 | 0 | 0 | 0 | 0 | 0 | 1 |
| ZNF141 | 0 | 1 | 0 | 0 | 0 | 0 | 0 | 0 | 1 |
| VASN | 0 | 1 | 0 | 0 | 0 | 0 | 0 | 0 | 1 |
| DYDC2 | 1 | 0 | 0 | 0 | 0 | 0 | 0 | 0 | 1 |
| KRTAP19-8 | 1 | 0 | 0 | 0 | 0 | 0 | 0 | 0 | 1 |
| DNAJC8 | 1 | 0 | 0 | 0 | 0 | 0 | 0 | 0 | 1 |
| C16orf55 | 1 | 0 | 0 | 0 | 0 | 0 | 0 | 0 | 1 |
| ALKBH3 | 0 | 0 | 1 | 0 | 0 | 0 | 0 | 0 | 1 |
| SLC10A7 | 0 | 0 | 1 | 0 | 0 | 0 | 0 | 0 | 1 |
| DEFB110 | 1 | 0 | 0 | 0 | 0 | 0 | 0 | 0 | 1 |
| RPS23 | 1 | 0 | 0 | 0 | 0 | 0 | 0 | 0 | 1 |
| SPP2 | 1 | 0 | 0 | 0 | 0 | 0 | 0 | 0 | 1 |
| C12orf77 | 1 | 0 | 0 | 0 | 0 | 0 | 0 | 0 | 1 |
| RBM38 | 1 | 0 | 0 | 0 | 0 | 0 | 0 | 0 | 1 |
| IFT52 | 0 | 1 | 0 | 0 | 0 | 0 | 0 | 0 | 1 |
| C12orf59 | 1 | 0 | 0 | 0 | 0 | 0 | 0 | 0 | 1 |
| HIST1H2AE | 1 | 0 | 0 | 0 | 0 | 0 | 0 | 0 | 1 |
| SPINK13 | 1 | 0 | 0 | 0 | 0 | 0 | 0 | 0 | 1 |
| C3orf79 | 1 | 0 | 0 | 0 | 0 | 0 | 0 | 0 | 1 |
| C9orf116 | 1 | 0 | 0 | 0 | 0 | 0 | 0 | 0 | 1 |

| | | | | | | | | | |
|---|---|---|---|---|---|---|---|---|---|
| SNAPC2 | 0 | 1 | 0 | 0 | 0 | 0 | 0 | 0 | 1 |
| CENPA | 1 | 0 | 0 | 0 | 0 | 0 | 0 | 0 | 1 |
| SPINT1 | 0 | 1 | 0 | 0 | 0 | 0 | 0 | 0 | 1 |
| RPL22 | 1 | 0 | 0 | 0 | 0 | 0 | 0 | 0 | 1 |
| TAPBPL | 0 | 1 | 0 | 0 | 0 | 0 | 0 | 0 | 1 |
| CD2BP2 | 0 | 0 | 1 | 0 | 0 | 0 | 0 | 0 | 1 |
| RBP7 | 1 | 0 | 0 | 0 | 0 | 0 | 0 | 0 | 1 |
| PABPN1L | 1 | 0 | 0 | 0 | 0 | 0 | 0 | 0 | 1 |
| LRTOMT | 0 | 1 | 0 | 0 | 0 | 0 | 0 | 0 | 1 |
| CTDSPL2 | 0 | 0 | 1 | 0 | 0 | 0 | 0 | 0 | 1 |
| PHAX | 0 | 1 | 0 | 0 | 0 | 0 | 0 | 0 | 1 |
| CLDN4 | 0 | 0 | 0 | 0 | 1 | 0 | 0 | 0 | 1 |
| KRTAP6-2 | 1 | 0 | 0 | 0 | 0 | 0 | 0 | 0 | 1 |
| B2M | 1 | 0 | 0 | 0 | 0 | 0 | 0 | 0 | 1 |
| FAM174B | 1 | 0 | 0 | 0 | 0 | 0 | 0 | 0 | 1 |
| NR2E3 | 0 | 0 | 1 | 0 | 0 | 0 | 0 | 0 | 1 |
| FAM163A | 1 | 0 | 0 | 0 | 0 | 0 | 0 | 0 | 1 |
| DCP2 | 0 | 1 | 0 | 0 | 0 | 0 | 0 | 0 | 1 |
| TTR | 1 | 0 | 0 | 0 | 0 | 0 | 0 | 0 | 1 |
| FABP2 | 1 | 0 | 0 | 0 | 0 | 0 | 0 | 0 | 1 |
| KLHL21 | 0 | 1 | 0 | 0 | 0 | 0 | 0 | 0 | 1 |
| C4orf45 | 1 | 0 | 0 | 0 | 0 | 0 | 0 | 0 | 1 |
| FSTL1 | 0 | 1 | 0 | 0 | 0 | 0 | 0 | 0 | 1 |
| SLC37A4 | 0 | 1 | 0 | 0 | 0 | 0 | 0 | 0 | 1 |
| PKIA | 1 | 0 | 0 | 0 | 0 | 0 | 0 | 0 | 1 |
| HIST1H2BO | 1 | 0 | 0 | 0 | 0 | 0 | 0 | 0 | 1 |
| LMAN2 | 1 | 0 | 0 | 0 | 0 | 0 | 0 | 0 | 1 |
| LEPROT | 1 | 0 | 0 | 0 | 0 | 0 | 0 | 0 | 1 |
| C20orf166 | 1 | 0 | 0 | 0 | 0 | 0 | 0 | 0 | 1 |
| COX7A2L | 1 | 0 | 0 | 0 | 0 | 0 | 0 | 0 | 1 |
| FAM46A | 0 | 1 | 0 | 0 | 0 | 0 | 0 | 0 | 1 |
| C3orf62 | 1 | 0 | 0 | 0 | 0 | 0 | 0 | 0 | 1 |
| MXD1 | 1 | 0 | 0 | 0 | 0 | 0 | 0 | 0 | 1 |
| UBE2L3 | 1 | 0 | 0 | 0 | 0 | 0 | 0 | 0 | 1 |
| PIGF | 1 | 0 | 0 | 0 | 0 | 0 | 0 | 0 | 1 |
| TM4SF4 | 1 | 0 | 0 | 0 | 0 | 0 | 0 | 0 | 1 |
| TM2D1 | 1 | 0 | 0 | 0 | 0 | 0 | 0 | 0 | 1 |
| ASB18 | 0 | 1 | 0 | 0 | 0 | 0 | 0 | 0 | 1 |
| EEF1B2 | 0 | 0 | 0 | 0 | 1 | 0 | 0 | 0 | 1 |
| ZNF474 | 0 | 1 | 0 | 0 | 0 | 0 | 0 | 0 | 1 |
| HIST1H3B | 1 | 0 | 0 | 0 | 0 | 0 | 0 | 0 | 1 |
| PLA2G2C | 1 | 0 | 0 | 0 | 0 | 0 | 0 | 0 | 1 |
| GABARAPL1 | 1 | 0 | 0 | 0 | 0 | 0 | 0 | 0 | 1 |
| CHRAC1 | 1 | 0 | 0 | 0 | 0 | 0 | 0 | 0 | 1 |
| REEP6 | 1 | 0 | 0 | 0 | 0 | 0 | 0 | 0 | 1 |
| C6orf195 | 1 | 0 | 0 | 0 | 0 | 0 | 0 | 0 | 1 |

| | | | | | | | | | |
|---|---|---|---|---|---|---|---|---|---|
| CLDN1 | 1 | 0 | 0 | 0 | 0 | 0 | 0 | 0 | 1 |
| SCN3B | 1 | 0 | 0 | 0 | 0 | 0 | 0 | 0 | 1 |
| SFTPA2 | 1 | 0 | 0 | 0 | 0 | 0 | 0 | 0 | 1 |
| UBE2K | 1 | 0 | 0 | 0 | 0 | 0 | 0 | 0 | 1 |
| PAGE4 | 1 | 0 | 0 | 0 | 0 | 0 | 0 | 0 | 1 |
| C15orf57 | 1 | 0 | 0 | 0 | 0 | 0 | 0 | 0 | 1 |
| RBM18 | 1 | 0 | 0 | 0 | 0 | 0 | 0 | 0 | 1 |
| RTP2 | 1 | 0 | 0 | 0 | 0 | 0 | 0 | 0 | 1 |
| FAM49B | 0 | 0 | 0 | 0 | 1 | 0 | 0 | 0 | 1 |
| NRF1 | 0 | 1 | 0 | 0 | 0 | 0 | 0 | 0 | 1 |
| RHOF | 1 | 0 | 0 | 0 | 0 | 0 | 0 | 0 | 1 |
| HSD17B10 | 1 | 0 | 0 | 0 | 0 | 0 | 0 | 0 | 1 |
| H2BFWT | 1 | 0 | 0 | 0 | 0 | 0 | 0 | 0 | 1 |
| LTA | 1 | 0 | 0 | 0 | 0 | 0 | 0 | 0 | 1 |
| C2orf80 | 1 | 0 | 0 | 0 | 0 | 0 | 0 | 0 | 1 |
| ATP5F1 | 0 | 0 | 0 | 0 | 1 | 0 | 0 | 0 | 1 |
| TSPAN16 | 1 | 0 | 0 | 0 | 0 | 0 | 0 | 0 | 1 |
| IFT20 | 1 | 0 | 0 | 0 | 0 | 0 | 0 | 0 | 1 |
| MED19 | 1 | 0 | 0 | 0 | 0 | 0 | 0 | 0 | 1 |
| C10orf82 | 1 | 0 | 0 | 0 | 0 | 0 | 0 | 0 | 1 |
| GATA3 | 0 | 1 | 0 | 0 | 0 | 0 | 0 | 0 | 1 |
| MRPL27 | 1 | 0 | 0 | 0 | 0 | 0 | 0 | 0 | 1 |
| C17orf85 | 0 | 1 | 0 | 0 | 0 | 0 | 0 | 0 | 1 |
| CHAC2 | 1 | 0 | 0 | 0 | 0 | 0 | 0 | 0 | 1 |
| IL13 | 1 | 0 | 0 | 0 | 0 | 0 | 0 | 0 | 1 |
| ENKUR | 1 | 0 | 0 | 0 | 0 | 0 | 0 | 0 | 1 |
| PDP2 | 0 | 1 | 0 | 0 | 0 | 0 | 0 | 0 | 1 |
| CT62 | 1 | 0 | 0 | 0 | 0 | 0 | 0 | 0 | 1 |
| CD59 | 1 | 0 | 0 | 0 | 0 | 0 | 0 | 0 | 1 |
| ISCA1 | 1 | 0 | 0 | 0 | 0 | 0 | 0 | 0 | 1 |
| KCNMB1 | 1 | 0 | 0 | 0 | 0 | 0 | 0 | 0 | 1 |
| KRTAP4-4 | 1 | 0 | 0 | 0 | 0 | 0 | 0 | 0 | 1 |
| DEFB112 | 1 | 0 | 0 | 0 | 0 | 0 | 0 | 0 | 1 |
| OSTBETA | 1 | 0 | 0 | 0 | 0 | 0 | 0 | 0 | 1 |
| NT5M | 1 | 0 | 0 | 0 | 0 | 0 | 0 | 0 | 1 |
| C10orf67 | 1 | 0 | 0 | 0 | 0 | 0 | 0 | 0 | 1 |
| VPREB3 | 1 | 0 | 0 | 0 | 0 | 0 | 0 | 0 | 1 |
| CLDN17 | 0 | 1 | 0 | 0 | 0 | 0 | 0 | 0 | 1 |
| OR2V2 | 0 | 1 | 0 | 0 | 0 | 0 | 0 | 0 | 1 |
| C21orf33 | 1 | 0 | 0 | 0 | 0 | 0 | 0 | 0 | 1 |
| WDR26 | 0 | 1 | 0 | 0 | 0 | 0 | 0 | 0 | 1 |
| ATP6V0D2 | 0 | 0 | 1 | 0 | 0 | 0 | 0 | 0 | 1 |
| C6orf52 | 1 | 0 | 0 | 0 | 0 | 0 | 0 | 0 | 1 |
| PPBP | 1 | 0 | 0 | 0 | 0 | 0 | 0 | 0 | 1 |
| UBL4A | 1 | 0 | 0 | 0 | 0 | 0 | 0 | 0 | 1 |
| C11orf20 | 1 | 0 | 0 | 0 | 0 | 0 | 0 | 0 | 1 |

| | | | | | | | | | |
|---|---|---|---|---|---|---|---|---|---|
| CHMP4A | 1 | 0 | 0 | 0 | 0 | 0 | 0 | 0 | 1 |
| HIST1H2BH | 1 | 0 | 0 | 0 | 0 | 0 | 0 | 0 | 1 |
| TXN2 | 1 | 0 | 0 | 0 | 0 | 0 | 0 | 0 | 1 |
| APOBEC3C | 1 | 0 | 0 | 0 | 0 | 0 | 0 | 0 | 1 |
| SUB1 | 1 | 0 | 0 | 0 | 0 | 0 | 0 | 0 | 1 |
| RNF145 | 0 | 0 | 1 | 0 | 0 | 0 | 0 | 0 | 1 |
| MRPL17 | 1 | 0 | 0 | 0 | 0 | 0 | 0 | 0 | 1 |
| TIMM17B | 1 | 0 | 0 | 0 | 0 | 0 | 0 | 0 | 1 |
| PTPN2 | 0 | 1 | 0 | 0 | 0 | 0 | 0 | 0 | 1 |
| SGPP2 | 0 | 1 | 0 | 0 | 0 | 0 | 0 | 0 | 1 |
| TCF15 | 1 | 0 | 0 | 0 | 0 | 0 | 0 | 0 | 1 |
| METTL10 | 1 | 0 | 0 | 0 | 0 | 0 | 0 | 0 | 1 |
| COPZ2 | 1 | 0 | 0 | 0 | 0 | 0 | 0 | 0 | 1 |
| C22orf15 | 1 | 0 | 0 | 0 | 0 | 0 | 0 | 0 | 1 |
| OCIAD2 | 1 | 0 | 0 | 0 | 0 | 0 | 0 | 0 | 1 |
| RPRD1A | 1 | 0 | 0 | 0 | 0 | 0 | 0 | 0 | 1 |
| FAM169B | 1 | 0 | 0 | 0 | 0 | 0 | 0 | 0 | 1 |
| PSMB3 | 1 | 0 | 0 | 0 | 0 | 0 | 0 | 0 | 1 |
| CLEC12B | 1 | 0 | 0 | 0 | 0 | 0 | 0 | 0 | 1 |
| TMEM92 | 1 | 0 | 0 | 0 | 0 | 0 | 0 | 0 | 1 |
| MMGT1 | 1 | 0 | 0 | 0 | 0 | 0 | 0 | 0 | 1 |
| TAF5L | 0 | 1 | 0 | 0 | 0 | 0 | 0 | 0 | 1 |
| FAM167B | 1 | 0 | 0 | 0 | 0 | 0 | 0 | 0 | 1 |
| FAM177B | 1 | 0 | 0 | 0 | 0 | 0 | 0 | 0 | 1 |
| UBE2U | 1 | 0 | 0 | 0 | 0 | 0 | 0 | 0 | 1 |
| ANKRD33B | 0 | 1 | 0 | 0 | 0 | 0 | 0 | 0 | 1 |
| C9orf89 | 1 | 0 | 0 | 0 | 0 | 0 | 0 | 0 | 1 |
| CXorf26 | 1 | 0 | 0 | 0 | 0 | 0 | 0 | 0 | 1 |
| SYN2 | 0 | 0 | 1 | 0 | 0 | 0 | 0 | 0 | 1 |
| ATG12 | 1 | 0 | 0 | 0 | 0 | 0 | 0 | 0 | 1 |
| C20orf118 | 1 | 0 | 0 | 0 | 0 | 0 | 0 | 0 | 1 |
| C5orf28 | 1 | 0 | 0 | 0 | 0 | 0 | 0 | 0 | 1 |
| FBXO32 | 1 | 0 | 0 | 0 | 0 | 0 | 0 | 0 | 1 |
| WDR12 | 0 | 1 | 0 | 0 | 0 | 0 | 0 | 0 | 1 |
| FAR2 | 0 | 0 | 1 | 0 | 0 | 0 | 0 | 0 | 1 |
| SLC2A4RG | 0 | 1 | 0 | 0 | 0 | 0 | 0 | 0 | 1 |
| FNTB | 0 | 1 | 0 | 0 | 0 | 0 | 0 | 0 | 1 |
| SLCO4A1 | 0 | 1 | 0 | 0 | 0 | 0 | 0 | 0 | 1 |
| RBM24 | 1 | 0 | 0 | 0 | 0 | 0 | 0 | 0 | 1 |
| LMO3 | 1 | 0 | 0 | 0 | 0 | 0 | 0 | 0 | 1 |
| RAX | 1 | 0 | 0 | 0 | 0 | 0 | 0 | 0 | 1 |
| FGF19 | 1 | 0 | 0 | 0 | 0 | 0 | 0 | 0 | 1 |
| SPATA3 | 1 | 0 | 0 | 0 | 0 | 0 | 0 | 0 | 1 |
| SECTM1 | 1 | 0 | 0 | 0 | 0 | 0 | 0 | 0 | 1 |
| BCAP29 | 0 | 1 | 0 | 0 | 0 | 0 | 0 | 0 | 1 |
| CD302 | 1 | 0 | 0 | 0 | 0 | 0 | 0 | 0 | 1 |

| | | | | | | | | | |
|---|---|---|---|---|---|---|---|---|---|
| PTPN11 | 0 | 0 | 1 | 0 | 0 | 0 | 0 | 0 | 1 |
| MAGOH | 1 | 0 | 0 | 0 | 0 | 0 | 0 | 0 | 1 |
| MGST1 | 1 | 0 | 0 | 0 | 0 | 0 | 0 | 0 | 1 |
| NSA2 | 0 | 0 | 0 | 0 | 1 | 0 | 0 | 0 | 1 |
| ZNF384 | 0 | 1 | 0 | 0 | 0 | 0 | 0 | 0 | 1 |
| MRPL36 | 1 | 0 | 0 | 0 | 0 | 0 | 0 | 0 | 1 |
| C1D | 1 | 0 | 0 | 0 | 0 | 0 | 0 | 0 | 1 |
| STXBP3 | 0 | 1 | 0 | 0 | 0 | 0 | 0 | 0 | 1 |
| MLN | 1 | 0 | 0 | 0 | 0 | 0 | 0 | 0 | 1 |
| TMEM17 | 1 | 0 | 0 | 0 | 0 | 0 | 0 | 0 | 1 |
| NDUFB9 | 1 | 0 | 0 | 0 | 0 | 0 | 0 | 0 | 1 |
| POLR3F | 1 | 0 | 0 | 0 | 0 | 0 | 0 | 0 | 1 |
| KHDC1 | 1 | 0 | 0 | 0 | 0 | 0 | 0 | 0 | 1 |
| UPP1 | 1 | 0 | 0 | 0 | 0 | 0 | 0 | 0 | 1 |
| BCL10 | 1 | 0 | 0 | 0 | 0 | 0 | 0 | 0 | 1 |
| LYVE1 | 1 | 0 | 0 | 0 | 0 | 0 | 0 | 0 | 1 |
| PAICS | 0 | 1 | 0 | 0 | 0 | 0 | 0 | 0 | 1 |
| LDOC1L | 1 | 0 | 0 | 0 | 0 | 0 | 0 | 0 | 1 |
| PPP2R5C | 0 | 1 | 0 | 0 | 0 | 0 | 0 | 0 | 1 |
| GSTCD | 0 | 0 | 1 | 0 | 0 | 0 | 0 | 0 | 1 |
| FAM168A | 1 | 0 | 0 | 0 | 0 | 0 | 0 | 0 | 1 |
| SLMO2 | 1 | 0 | 0 | 0 | 0 | 0 | 0 | 0 | 1 |
| EDC3 | 0 | 1 | 0 | 0 | 0 | 0 | 0 | 0 | 1 |
| C1QTNF3 | 0 | 1 | 0 | 0 | 0 | 0 | 0 | 0 | 1 |
| LYG1 | 1 | 0 | 0 | 0 | 0 | 0 | 0 | 0 | 1 |
| TSPO | 1 | 0 | 0 | 0 | 0 | 0 | 0 | 0 | 1 |
| ANKRD22 | 1 | 0 | 0 | 0 | 0 | 0 | 0 | 0 | 1 |
| SENP8 | 1 | 0 | 0 | 0 | 0 | 0 | 0 | 0 | 1 |
| CYB5D1 | 1 | 0 | 0 | 0 | 0 | 0 | 0 | 0 | 1 |
| IL17C | 1 | 0 | 0 | 0 | 0 | 0 | 0 | 0 | 1 |
| RBPJL | 0 | 0 | 1 | 0 | 0 | 0 | 0 | 0 | 1 |
| OMP | 1 | 0 | 0 | 0 | 0 | 0 | 0 | 0 | 1 |
| TRAFD1 | 0 | 1 | 0 | 0 | 0 | 0 | 0 | 0 | 1 |
| RNASE7 | 1 | 0 | 0 | 0 | 0 | 0 | 0 | 0 | 1 |
| ZNF720 | 1 | 0 | 0 | 0 | 0 | 0 | 0 | 0 | 1 |
| TCEAL7 | 1 | 0 | 0 | 0 | 0 | 0 | 0 | 0 | 1 |
| TMEM126A | 1 | 0 | 0 | 0 | 0 | 0 | 0 | 0 | 1 |
| ZNF75A | 1 | 0 | 0 | 0 | 0 | 0 | 0 | 0 | 1 |
| YWHAQ | 1 | 0 | 0 | 0 | 0 | 0 | 0 | 0 | 1 |
| IDI1 | 1 | 0 | 0 | 0 | 0 | 0 | 0 | 0 | 1 |
| MAOB | 0 | 1 | 0 | 0 | 0 | 0 | 0 | 0 | 1 |
| C14orf142 | 1 | 0 | 0 | 0 | 0 | 0 | 0 | 0 | 1 |
| PRKRA | 1 | 0 | 0 | 0 | 0 | 0 | 0 | 0 | 1 |
| OR8G1 | 0 | 0 | 1 | 0 | 0 | 0 | 0 | 0 | 1 |
| CD274 | 1 | 0 | 0 | 0 | 0 | 0 | 0 | 0 | 1 |
| KRTAP1-1 | 1 | 0 | 0 | 0 | 0 | 0 | 0 | 0 | 1 |

| | | | | | | | | | |
|---|---|---|---|---|---|---|---|---|---|
| ELL3 | 0 | 0 | 1 | 0 | 0 | 0 | 0 | 0 | 1 |
| TCN2 | 0 | 0 | 1 | 0 | 0 | 0 | 0 | 0 | 1 |
| PLEKHB2 | 1 | 0 | 0 | 0 | 0 | 0 | 0 | 0 | 1 |
| TMEM9B | 1 | 0 | 0 | 0 | 0 | 0 | 0 | 0 | 1 |
| HSPB8 | 1 | 0 | 0 | 0 | 0 | 0 | 0 | 0 | 1 |
| LETM2 | 1 | 0 | 0 | 0 | 0 | 0 | 0 | 0 | 1 |
| PPAPDC1B | 1 | 0 | 0 | 0 | 0 | 0 | 0 | 0 | 1 |
| KLRD1 | 1 | 0 | 0 | 0 | 0 | 0 | 0 | 0 | 1 |
| COMMD7 | 1 | 0 | 0 | 0 | 0 | 0 | 0 | 0 | 1 |
| ARF3 | 1 | 0 | 0 | 0 | 0 | 0 | 0 | 0 | 1 |
| EIF4E | 1 | 0 | 0 | 0 | 0 | 0 | 0 | 0 | 1 |
| PRPS2 | 1 | 0 | 0 | 0 | 0 | 0 | 0 | 0 | 1 |
| FAM57A | 1 | 0 | 0 | 0 | 0 | 0 | 0 | 0 | 1 |
| RBPMS2 | 1 | 0 | 0 | 0 | 0 | 0 | 0 | 0 | 1 |
| ANKS3 | 0 | 1 | 0 | 0 | 0 | 0 | 0 | 0 | 1 |
| C16orf5 | 1 | 0 | 0 | 0 | 0 | 0 | 0 | 0 | 1 |
| TTC8 | 0 | 0 | 1 | 0 | 0 | 0 | 0 | 0 | 1 |
| KRTAP6-1 | 1 | 0 | 0 | 0 | 0 | 0 | 0 | 0 | 1 |
| MRPS23 | 1 | 0 | 0 | 0 | 0 | 0 | 0 | 0 | 1 |
| APOD | 1 | 0 | 0 | 0 | 0 | 0 | 0 | 0 | 1 |
| ATP5G1 | 1 | 0 | 0 | 0 | 0 | 0 | 0 | 0 | 1 |
| P2RY12 | 1 | 0 | 0 | 0 | 0 | 0 | 0 | 0 | 1 |
| GPR160 | 1 | 0 | 0 | 0 | 0 | 0 | 0 | 0 | 1 |
| RSL24D1 | 1 | 0 | 0 | 0 | 0 | 0 | 0 | 0 | 1 |
| GCSH | 1 | 0 | 0 | 0 | 0 | 0 | 0 | 0 | 1 |
| ABT1 | 1 | 0 | 0 | 0 | 0 | 0 | 0 | 0 | 1 |
| RAB33A | 1 | 0 | 0 | 0 | 0 | 0 | 0 | 0 | 1 |
| FGD4 | 0 | 0 | 1 | 0 | 0 | 0 | 0 | 0 | 1 |
| STXBP6 | 1 | 0 | 0 | 0 | 0 | 0 | 0 | 0 | 1 |
| LCE1C | 1 | 0 | 0 | 0 | 0 | 0 | 0 | 0 | 1 |
| PGLS | 1 | 0 | 0 | 0 | 0 | 0 | 0 | 0 | 1 |
| PLA2G2D | 1 | 0 | 0 | 0 | 0 | 0 | 0 | 0 | 1 |
| BTC | 1 | 0 | 0 | 0 | 0 | 0 | 0 | 0 | 1 |
| C4orf36 | 1 | 0 | 0 | 0 | 0 | 0 | 0 | 0 | 1 |
| PRIMA1 | 1 | 0 | 0 | 0 | 0 | 0 | 0 | 0 | 1 |
| C5orf34 | 0 | 1 | 0 | 0 | 0 | 0 | 0 | 0 | 1 |
| C9orf85 | 1 | 0 | 0 | 0 | 0 | 0 | 0 | 0 | 1 |
| RBM41 | 0 | 1 | 0 | 0 | 0 | 0 | 0 | 0 | 1 |
| FERMT3 | 0 | 1 | 0 | 0 | 0 | 0 | 0 | 0 | 1 |
| LSM5 | 1 | 0 | 0 | 0 | 0 | 0 | 0 | 0 | 1 |
| THAP11 | 1 | 0 | 0 | 0 | 0 | 0 | 0 | 0 | 1 |
| TMEM186 | 1 | 0 | 0 | 0 | 0 | 0 | 0 | 0 | 1 |
| MPZL2 | 1 | 0 | 0 | 0 | 0 | 0 | 0 | 0 | 1 |
| OTOR | 1 | 0 | 0 | 0 | 0 | 0 | 0 | 0 | 1 |
| NAA50 | 1 | 0 | 0 | 0 | 0 | 0 | 0 | 0 | 1 |
| MRPS28 | 1 | 0 | 0 | 0 | 0 | 0 | 0 | 0 | 1 |

| | | | | | | | | | |
|---|---|---|---|---|---|---|---|---|---|
| PDE6D | 1 | 0 | 0 | 0 | 0 | 0 | 0 | 0 | 1 |
| LCE3B | 1 | 0 | 0 | 0 | 0 | 0 | 0 | 0 | 1 |
| C9orf170 | 1 | 0 | 0 | 0 | 0 | 0 | 0 | 0 | 1 |
| RPLP0 | 1 | 0 | 0 | 0 | 0 | 0 | 0 | 0 | 1 |
| ZNF555 | 0 | 0 | 1 | 0 | 0 | 0 | 0 | 0 | 1 |
| LCLAT1 | 0 | 1 | 0 | 0 | 0 | 0 | 0 | 0 | 1 |
| RAB11A | 1 | 0 | 0 | 0 | 0 | 0 | 0 | 0 | 1 |
| FCF1 | 1 | 0 | 0 | 0 | 0 | 0 | 0 | 0 | 1 |
| NUDT21 | 1 | 0 | 0 | 0 | 0 | 0 | 0 | 0 | 1 |
| IFNW1 | 1 | 0 | 0 | 0 | 0 | 0 | 0 | 0 | 1 |
| THEM5 | 1 | 0 | 0 | 0 | 0 | 0 | 0 | 0 | 1 |
| STARD5 | 1 | 0 | 0 | 0 | 0 | 0 | 0 | 0 | 1 |
| HMOX2 | 1 | 0 | 0 | 0 | 0 | 0 | 0 | 0 | 1 |
| C5orf24 | 1 | 0 | 0 | 0 | 0 | 0 | 0 | 0 | 1 |
| PATL2 | 0 | 1 | 0 | 0 | 0 | 0 | 0 | 0 | 1 |
| DEFB124 | 1 | 0 | 0 | 0 | 0 | 0 | 0 | 0 | 1 |
| CLEC2L | 1 | 0 | 0 | 0 | 0 | 0 | 0 | 0 | 1 |
| CHIC2 | 1 | 0 | 0 | 0 | 0 | 0 | 0 | 0 | 1 |
| PRR3 | 1 | 0 | 0 | 0 | 0 | 0 | 0 | 0 | 1 |
| GFRA3 | 1 | 0 | 0 | 0 | 0 | 0 | 0 | 0 | 1 |
| ACOX3 | 0 | 1 | 0 | 0 | 0 | 0 | 0 | 0 | 1 |
| RNLS | 1 | 0 | 0 | 0 | 0 | 0 | 0 | 0 | 1 |
| UBE2R2 | 1 | 0 | 0 | 0 | 0 | 0 | 0 | 0 | 1 |
| CENPK | 1 | 0 | 0 | 0 | 0 | 0 | 0 | 0 | 1 |
| CRCP | 1 | 0 | 0 | 0 | 0 | 0 | 0 | 0 | 1 |
| ABHD4 | 1 | 0 | 0 | 0 | 0 | 0 | 0 | 0 | 1 |
| SLC24A6 | 0 | 0 | 1 | 0 | 0 | 0 | 0 | 0 | 1 |
| SH2D4A | 0 | 1 | 0 | 0 | 0 | 0 | 0 | 0 | 1 |
| CPLX3 | 1 | 0 | 0 | 0 | 0 | 0 | 0 | 0 | 1 |
| C14orf126 | 1 | 0 | 0 | 0 | 0 | 0 | 0 | 0 | 1 |
| BCDIN3D | 1 | 0 | 0 | 0 | 0 | 0 | 0 | 0 | 1 |
| DDAH1 | 1 | 0 | 0 | 0 | 0 | 0 | 0 | 0 | 1 |
| REM1 | 0 | 1 | 0 | 0 | 0 | 0 | 0 | 0 | 1 |
| PAGE1 | 1 | 0 | 0 | 0 | 0 | 0 | 0 | 0 | 1 |
| CD70 | 1 | 0 | 0 | 0 | 0 | 0 | 0 | 0 | 1 |
| RPA3 | 1 | 0 | 0 | 0 | 0 | 0 | 0 | 0 | 1 |
| CSTL1 | 1 | 0 | 0 | 0 | 0 | 0 | 0 | 0 | 1 |
| NTS | 1 | 0 | 0 | 0 | 0 | 0 | 0 | 0 | 1 |
| TMCO2 | 1 | 0 | 0 | 0 | 0 | 0 | 0 | 0 | 1 |
| C9orf11 | 1 | 0 | 0 | 0 | 0 | 0 | 0 | 0 | 1 |
| ARPC1B | 1 | 0 | 0 | 0 | 0 | 0 | 0 | 0 | 1 |
| PAQR7 | 1 | 0 | 0 | 0 | 0 | 0 | 0 | 0 | 1 |
| PGRMC1 | 1 | 0 | 0 | 0 | 0 | 0 | 0 | 0 | 1 |
| TRIM39 | 1 | 0 | 0 | 0 | 0 | 0 | 0 | 0 | 1 |
| SOD2 | 1 | 0 | 0 | 0 | 0 | 0 | 0 | 0 | 1 |
| MRPL35 | 1 | 0 | 0 | 0 | 0 | 0 | 0 | 0 | 1 |

| | | | | | | | | | |
|---|---|---|---|---|---|---|---|---|---|
| UBE2G2 | 1 | 0 | 0 | 0 | 0 | 0 | 0 | 0 | 1 |
| TMED5 | 1 | 0 | 0 | 0 | 0 | 0 | 0 | 0 | 1 |
| SUZ12 | 0 | 1 | 0 | 0 | 0 | 0 | 0 | 0 | 1 |
| PIK3IP1 | 1 | 0 | 0 | 0 | 0 | 0 | 0 | 0 | 1 |
| MAP1LC3C | 1 | 0 | 0 | 0 | 0 | 0 | 0 | 0 | 1 |
| TIPIN | 1 | 0 | 0 | 0 | 0 | 0 | 0 | 0 | 1 |
| SUN1 | 0 | 0 | 1 | 0 | 0 | 0 | 0 | 0 | 1 |
| CYB561 | 1 | 0 | 0 | 0 | 0 | 0 | 0 | 0 | 1 |
| MYL6 | 1 | 0 | 0 | 0 | 0 | 0 | 0 | 0 | 1 |
| NMU | 1 | 0 | 0 | 0 | 0 | 0 | 0 | 0 | 1 |
| NFIC | 0 | 1 | 0 | 0 | 0 | 0 | 0 | 0 | 1 |
| SERPINB12 | 0 | 1 | 0 | 0 | 0 | 0 | 0 | 0 | 1 |
| C8orf76 | 1 | 0 | 0 | 0 | 0 | 0 | 0 | 0 | 1 |
| TOMM22 | 1 | 0 | 0 | 0 | 0 | 0 | 0 | 0 | 1 |
| PHLDA1 | 1 | 0 | 0 | 0 | 0 | 0 | 0 | 0 | 1 |
| ZDHHC22 | 1 | 0 | 0 | 0 | 0 | 0 | 0 | 0 | 1 |
| C2orf62 | 1 | 0 | 0 | 0 | 0 | 0 | 0 | 0 | 1 |
| LCN2 | 1 | 0 | 0 | 0 | 0 | 0 | 0 | 0 | 1 |
| PDAP1 | 1 | 0 | 0 | 0 | 0 | 0 | 0 | 0 | 1 |
| C2orf51 | 1 | 0 | 0 | 0 | 0 | 0 | 0 | 0 | 1 |
| ZNF586 | 0 | 1 | 0 | 0 | 0 | 0 | 0 | 0 | 1 |
| HOXA6 | 1 | 0 | 0 | 0 | 0 | 0 | 0 | 0 | 1 |
| TMEM140 | 1 | 0 | 0 | 0 | 0 | 0 | 0 | 0 | 1 |
| OAZ3 | 1 | 0 | 0 | 0 | 0 | 0 | 0 | 0 | 1 |
| CREB5 | 0 | 1 | 0 | 0 | 0 | 0 | 0 | 0 | 1 |
| BAIAP2L1 | 1 | 0 | 0 | 0 | 0 | 0 | 0 | 0 | 1 |
| LY96 | 1 | 0 | 0 | 0 | 0 | 0 | 0 | 0 | 1 |
| UBE2H | 1 | 0 | 0 | 0 | 0 | 0 | 0 | 0 | 1 |
| VGLL1 | 1 | 0 | 0 | 0 | 0 | 0 | 0 | 0 | 1 |
| PDDC1 | 1 | 0 | 0 | 0 | 0 | 0 | 0 | 0 | 1 |
| CELA1 | 1 | 0 | 0 | 0 | 0 | 0 | 0 | 0 | 1 |
| COQ5 | 1 | 0 | 0 | 0 | 0 | 0 | 0 | 0 | 1 |
| ORMDL3 | 1 | 0 | 0 | 0 | 0 | 0 | 0 | 0 | 1 |
| CIAO1 | 1 | 0 | 0 | 0 | 0 | 0 | 0 | 0 | 1 |
| PPCDC | 1 | 0 | 0 | 0 | 0 | 0 | 0 | 0 | 1 |
| RABIF | 1 | 0 | 0 | 0 | 0 | 0 | 0 | 0 | 1 |
| TAP2 | 0 | 0 | 1 | 0 | 0 | 0 | 0 | 0 | 1 |
| GKN1 | 1 | 0 | 0 | 0 | 0 | 0 | 0 | 0 | 1 |
| STX1A | 1 | 0 | 0 | 0 | 0 | 0 | 0 | 0 | 1 |
| FIBIN | 1 | 0 | 0 | 0 | 0 | 0 | 0 | 0 | 1 |
| NACA2 | 1 | 0 | 0 | 0 | 0 | 0 | 0 | 0 | 1 |
| HIRIP3 | 0 | 0 | 1 | 0 | 0 | 0 | 0 | 0 | 1 |
| NIPSNAP1 | 1 | 0 | 0 | 0 | 0 | 0 | 0 | 0 | 1 |
| GGPS1 | 1 | 0 | 0 | 0 | 0 | 0 | 0 | 0 | 1 |
| MRPL22 | 1 | 0 | 0 | 0 | 0 | 0 | 0 | 0 | 1 |
| GJB5 | 1 | 0 | 0 | 0 | 0 | 0 | 0 | 0 | 1 |

| | | | | | | | | | |
|---|---|---|---|---|---|---|---|---|---|
| IGSF6 | 1 | 0 | 0 | 0 | 0 | 0 | 0 | 0 | 1 |
| LIF | 1 | 0 | 0 | 0 | 0 | 0 | 0 | 0 | 1 |
| RNF167 | 1 | 0 | 0 | 0 | 0 | 0 | 0 | 0 | 1 |
| CD9 | 1 | 0 | 0 | 0 | 0 | 0 | 0 | 0 | 1 |
| SLC9A5 | 0 | 1 | 0 | 0 | 0 | 0 | 0 | 0 | 1 |
| LCNL1 | 1 | 0 | 0 | 0 | 0 | 0 | 0 | 0 | 1 |
| NFYA | 0 | 0 | 0 | 0 | 1 | 0 | 0 | 0 | 1 |
| IGFBP2 | 1 | 0 | 0 | 0 | 0 | 0 | 0 | 0 | 1 |
| DCXR | 1 | 0 | 0 | 0 | 0 | 0 | 0 | 0 | 1 |
| PSMB2 | 1 | 0 | 0 | 0 | 0 | 0 | 0 | 0 | 1 |
| CCK | 1 | 0 | 0 | 0 | 0 | 0 | 0 | 0 | 1 |
| SSR2 | 1 | 0 | 0 | 0 | 0 | 0 | 0 | 0 | 1 |
| RHOA | 1 | 0 | 0 | 0 | 0 | 0 | 0 | 0 | 1 |
| DHRS7 | 1 | 0 | 0 | 0 | 0 | 0 | 0 | 0 | 1 |
| FMO5 | 0 | 1 | 0 | 0 | 0 | 0 | 0 | 0 | 1 |
| MED9 | 1 | 0 | 0 | 0 | 0 | 0 | 0 | 0 | 1 |
| MED7 | 1 | 0 | 0 | 0 | 0 | 0 | 0 | 0 | 1 |
| TMED2 | 1 | 0 | 0 | 0 | 0 | 0 | 0 | 0 | 1 |
| CRYGC | 1 | 0 | 0 | 0 | 0 | 0 | 0 | 0 | 1 |
| CARD18 | 1 | 0 | 0 | 0 | 0 | 0 | 0 | 0 | 1 |
| DBNDD1 | 1 | 0 | 0 | 0 | 0 | 0 | 0 | 0 | 1 |
| HPCAL1 | 1 | 0 | 0 | 0 | 0 | 0 | 0 | 0 | 1 |
| C1orf61 | 1 | 0 | 0 | 0 | 0 | 0 | 0 | 0 | 1 |
| C18orf21 | 1 | 0 | 0 | 0 | 0 | 0 | 0 | 0 | 1 |
| ABHD5 | 1 | 0 | 0 | 0 | 0 | 0 | 0 | 0 | 1 |
| NUP54 | 0 | 1 | 0 | 0 | 0 | 0 | 0 | 0 | 1 |
| JUNB | 1 | 0 | 0 | 0 | 0 | 0 | 0 | 0 | 1 |
| C9orf169 | 1 | 0 | 0 | 0 | 0 | 0 | 0 | 0 | 1 |
| AGPAT3 | 1 | 0 | 0 | 0 | 0 | 0 | 0 | 0 | 1 |
| SLC30A10 | 0 | 1 | 0 | 0 | 0 | 0 | 0 | 0 | 1 |
| SLC39A2 | 1 | 0 | 0 | 0 | 0 | 0 | 0 | 0 | 1 |
| HRASLS | 1 | 0 | 0 | 0 | 0 | 0 | 0 | 0 | 1 |
| MOSPD1 | 1 | 0 | 0 | 0 | 0 | 0 | 0 | 0 | 1 |
| DRAM2 | 1 | 0 | 0 | 0 | 0 | 0 | 0 | 0 | 1 |
| VPS26A | 1 | 0 | 0 | 0 | 0 | 0 | 0 | 0 | 1 |
| FAM125B | 1 | 0 | 0 | 0 | 0 | 0 | 0 | 0 | 1 |
| SAMD10 | 1 | 0 | 0 | 0 | 0 | 0 | 0 | 0 | 1 |
| CKMT2 | 1 | 0 | 0 | 0 | 0 | 0 | 0 | 0 | 1 |
| KRTAP5-9 | 1 | 0 | 0 | 0 | 0 | 0 | 0 | 0 | 1 |
| REG4 | 1 | 0 | 0 | 0 | 0 | 0 | 0 | 0 | 1 |
| ANAPC11 | 1 | 0 | 0 | 0 | 0 | 0 | 0 | 0 | 1 |
| NAB1 | 0 | 1 | 0 | 0 | 0 | 0 | 0 | 0 | 1 |
| PRPS1 | 0 | 1 | 0 | 0 | 0 | 0 | 0 | 0 | 1 |
| VEGFB | 1 | 0 | 0 | 0 | 0 | 0 | 0 | 0 | 1 |
| RNF166 | 1 | 0 | 0 | 0 | 0 | 0 | 0 | 0 | 1 |
| ZNRF2 | 1 | 0 | 0 | 0 | 0 | 0 | 0 | 0 | 1 |

| | | | | | | | | | |
|---|---|---|---|---|---|---|---|---|---|
| RQCD1 | 1 | 0 | 0 | 0 | 0 | 0 | 0 | 0 | 1 |
| FXYD3 | 1 | 0 | 0 | 0 | 0 | 0 | 0 | 0 | 1 |
| C20orf96 | 1 | 0 | 0 | 0 | 0 | 0 | 0 | 0 | 1 |
| GPRC5A | 1 | 0 | 0 | 0 | 0 | 0 | 0 | 0 | 1 |
| ZFP36L1 | 0 | 0 | 0 | 0 | 1 | 0 | 0 | 0 | 1 |
| ACOT13 | 1 | 0 | 0 | 0 | 0 | 0 | 0 | 0 | 1 |
| CRIP3 | 1 | 0 | 0 | 0 | 0 | 0 | 0 | 0 | 1 |
| CIB1 | 1 | 0 | 0 | 0 | 0 | 0 | 0 | 0 | 1 |
| PIAS2 | 0 | 1 | 0 | 0 | 0 | 0 | 0 | 0 | 1 |
| VPREB1 | 1 | 0 | 0 | 0 | 0 | 0 | 0 | 0 | 1 |
| CEP57 | 0 | 1 | 0 | 0 | 0 | 0 | 0 | 0 | 1 |
| PTGDS | 1 | 0 | 0 | 0 | 0 | 0 | 0 | 0 | 1 |
| GRTP1 | 1 | 0 | 0 | 0 | 0 | 0 | 0 | 0 | 1 |
| PGS1 | 0 | 1 | 0 | 0 | 0 | 0 | 0 | 0 | 1 |
| MUCL1 | 1 | 0 | 0 | 0 | 0 | 0 | 0 | 0 | 1 |
| PANK3 | 0 | 0 | 0 | 0 | 1 | 0 | 0 | 0 | 1 |
| C1QTNF1 | 1 | 0 | 0 | 0 | 0 | 0 | 0 | 0 | 1 |
| DNAJB11 | 1 | 0 | 0 | 0 | 0 | 0 | 0 | 0 | 1 |
| SNCA | 1 | 0 | 0 | 0 | 0 | 0 | 0 | 0 | 1 |
| HNRNPAB | 1 | 0 | 0 | 0 | 0 | 0 | 0 | 0 | 1 |
| UBE2E3 | 1 | 0 | 0 | 0 | 0 | 0 | 0 | 0 | 1 |
| CCL13 | 1 | 0 | 0 | 0 | 0 | 0 | 0 | 0 | 1 |
| TIFAB | 1 | 0 | 0 | 0 | 0 | 0 | 0 | 0 | 1 |
| EFHD2 | 1 | 0 | 0 | 0 | 0 | 0 | 0 | 0 | 1 |
| INS-IGF2 | 1 | 0 | 0 | 0 | 0 | 0 | 0 | 0 | 1 |
| COPZ1 | 1 | 0 | 0 | 0 | 0 | 0 | 0 | 0 | 1 |
| TAF6 | 0 | 0 | 1 | 0 | 0 | 0 | 0 | 0 | 1 |
| CBX3 | 1 | 0 | 0 | 0 | 0 | 0 | 0 | 0 | 1 |
| DMC1 | 1 | 0 | 0 | 0 | 0 | 0 | 0 | 0 | 1 |
| NRN1 | 1 | 0 | 0 | 0 | 0 | 0 | 0 | 0 | 1 |
| PPP1R14D | 1 | 0 | 0 | 0 | 0 | 0 | 0 | 0 | 1 |
| C11orf86 | 1 | 0 | 0 | 0 | 0 | 0 | 0 | 0 | 1 |
| FXN | 1 | 0 | 0 | 0 | 0 | 0 | 0 | 0 | 1 |
| VN1R1 | 0 | 1 | 0 | 0 | 0 | 0 | 0 | 0 | 1 |
| PLEKHF2 | 1 | 0 | 0 | 0 | 0 | 0 | 0 | 0 | 1 |
| FABP9 | 1 | 0 | 0 | 0 | 0 | 0 | 0 | 0 | 1 |
| LCN9 | 1 | 0 | 0 | 0 | 0 | 0 | 0 | 0 | 1 |
| CD3D | 1 | 0 | 0 | 0 | 0 | 0 | 0 | 0 | 1 |
| C2orf40 | 1 | 0 | 0 | 0 | 0 | 0 | 0 | 0 | 1 |
| HLA-DOB | 1 | 0 | 0 | 0 | 0 | 0 | 0 | 0 | 1 |
| JDP2 | 1 | 0 | 0 | 0 | 0 | 0 | 0 | 0 | 1 |
| DRAM1 | 1 | 0 | 0 | 0 | 0 | 0 | 0 | 0 | 1 |
| RAB17 | 1 | 0 | 0 | 0 | 0 | 0 | 0 | 0 | 1 |
| C22orf39 | 1 | 0 | 0 | 0 | 0 | 0 | 0 | 0 | 1 |
| PNMT | 1 | 0 | 0 | 0 | 0 | 0 | 0 | 0 | 1 |
| TPRG1 | 1 | 0 | 0 | 0 | 0 | 0 | 0 | 0 | 1 |

| | | | | | | | | | |
|---|---|---|---|---|---|---|---|---|---|
| OVCA2 | 1 | 0 | 0 | 0 | 0 | 0 | 0 | 0 | 1 |
| RHOXF1 | 1 | 0 | 0 | 0 | 0 | 0 | 0 | 0 | 1 |
| SMS | 1 | 0 | 0 | 0 | 0 | 0 | 0 | 0 | 1 |
| SNAI3 | 1 | 0 | 0 | 0 | 0 | 0 | 0 | 0 | 1 |
| ART4 | 1 | 0 | 0 | 0 | 0 | 0 | 0 | 0 | 1 |
| C18orf62 | 1 | 0 | 0 | 0 | 0 | 0 | 0 | 0 | 1 |
| KRTCAP2 | 1 | 0 | 0 | 0 | 0 | 0 | 0 | 0 | 1 |
| PYCR1 | 1 | 0 | 0 | 0 | 0 | 0 | 0 | 0 | 1 |
| PPIL3 | 1 | 0 | 0 | 0 | 0 | 0 | 0 | 0 | 1 |
| C1orf111 | 1 | 0 | 0 | 0 | 0 | 0 | 0 | 0 | 1 |
| CLK1 | 0 | 1 | 0 | 0 | 0 | 0 | 0 | 0 | 1 |
| FAM131A | 1 | 0 | 0 | 0 | 0 | 0 | 0 | 0 | 1 |
| UTS2 | 1 | 0 | 0 | 0 | 0 | 0 | 0 | 0 | 1 |
| IL7 | 1 | 0 | 0 | 0 | 0 | 0 | 0 | 0 | 1 |
| RGS16 | 0 | 0 | 0 | 0 | 1 | 0 | 0 | 0 | 1 |
| PPP1R1A | 1 | 0 | 0 | 0 | 0 | 0 | 0 | 0 | 1 |
| TRAF3IP3 | 0 | 1 | 0 | 0 | 0 | 0 | 0 | 0 | 1 |
| C6orf130 | 1 | 0 | 0 | 0 | 0 | 0 | 0 | 0 | 1 |
| TMEM187 | 1 | 0 | 0 | 0 | 0 | 0 | 0 | 0 | 1 |
| RNF182 | 1 | 0 | 0 | 0 | 0 | 0 | 0 | 0 | 1 |
| TSPAN10 | 1 | 0 | 0 | 0 | 0 | 0 | 0 | 0 | 1 |
| C1orf150 | 1 | 0 | 0 | 0 | 0 | 0 | 0 | 0 | 1 |
| MLEC | 1 | 0 | 0 | 0 | 0 | 0 | 0 | 0 | 1 |
| FOLR3 | 1 | 0 | 0 | 0 | 0 | 0 | 0 | 0 | 1 |
| PRSS48 | 0 | 1 | 0 | 0 | 0 | 0 | 0 | 0 | 1 |
| ALG14 | 1 | 0 | 0 | 0 | 0 | 0 | 0 | 0 | 1 |
| GPR82 | 1 | 0 | 0 | 0 | 0 | 0 | 0 | 0 | 1 |
| C16orf92 | 1 | 0 | 0 | 0 | 0 | 0 | 0 | 0 | 1 |
| MMD | 1 | 0 | 0 | 0 | 0 | 0 | 0 | 0 | 1 |
| C9orf142 | 1 | 0 | 0 | 0 | 0 | 0 | 0 | 0 | 1 |
| LIX1 | 1 | 0 | 0 | 0 | 0 | 0 | 0 | 0 | 1 |
| GCET2 | 1 | 0 | 0 | 0 | 0 | 0 | 0 | 0 | 1 |
| HAAO | 1 | 0 | 0 | 0 | 0 | 0 | 0 | 0 | 1 |
| CSF3 | 1 | 0 | 0 | 0 | 0 | 0 | 0 | 0 | 1 |
| NUDT15 | 1 | 0 | 0 | 0 | 0 | 0 | 0 | 0 | 1 |
| RIPPLY1 | 1 | 0 | 0 | 0 | 0 | 0 | 0 | 0 | 1 |
| CRP | 1 | 0 | 0 | 0 | 0 | 0 | 0 | 0 | 1 |
| PDZD9 | 1 | 0 | 0 | 0 | 0 | 0 | 0 | 0 | 1 |
| CD160 | 1 | 0 | 0 | 0 | 0 | 0 | 0 | 0 | 1 |
| LCN8 | 1 | 0 | 0 | 0 | 0 | 0 | 0 | 0 | 1 |
| CLDN11 | 1 | 0 | 0 | 0 | 0 | 0 | 0 | 0 | 1 |
| FABP12 | 1 | 0 | 0 | 0 | 0 | 0 | 0 | 0 | 1 |
| HNRPDL | 0 | 0 | 0 | 0 | 1 | 0 | 0 | 0 | 1 |
| PSMB7 | 1 | 0 | 0 | 0 | 0 | 0 | 0 | 0 | 1 |
| C11orf45 | 1 | 0 | 0 | 0 | 0 | 0 | 0 | 0 | 1 |
| FBXO27 | 1 | 0 | 0 | 0 | 0 | 0 | 0 | 0 | 1 |

| | | | | | | | | | |
|---|---|---|---|---|---|---|---|---|---|
| TCF21 | 1 | 0 | 0 | 0 | 0 | 0 | 0 | 0 | 1 |
| FDX1L | 1 | 0 | 0 | 0 | 0 | 0 | 0 | 0 | 1 |
| CD3G | 1 | 0 | 0 | 0 | 0 | 0 | 0 | 0 | 1 |
| KLK13 | 1 | 0 | 0 | 0 | 0 | 0 | 0 | 0 | 1 |
| MIOX | 1 | 0 | 0 | 0 | 0 | 0 | 0 | 0 | 1 |
| INSL4 | 1 | 0 | 0 | 0 | 0 | 0 | 0 | 0 | 1 |
| TMEM33 | 1 | 0 | 0 | 0 | 0 | 0 | 0 | 0 | 1 |
| C22orf24 | 1 | 0 | 0 | 0 | 0 | 0 | 0 | 0 | 1 |
| NTN1 | 0 | 1 | 0 | 0 | 0 | 0 | 0 | 0 | 1 |
| DNLZ | 1 | 0 | 0 | 0 | 0 | 0 | 0 | 0 | 1 |
| SLC22A18AS | 1 | 0 | 0 | 0 | 0 | 0 | 0 | 0 | 1 |
| MRPL42 | 1 | 0 | 0 | 0 | 0 | 0 | 0 | 0 | 1 |
| KCNAB2 | 1 | 0 | 0 | 0 | 0 | 0 | 0 | 0 | 1 |
| ASB11 | 1 | 0 | 0 | 0 | 0 | 0 | 0 | 0 | 1 |
| MYCT1 | 1 | 0 | 0 | 0 | 0 | 0 | 0 | 0 | 1 |
| CD8B | 1 | 0 | 0 | 0 | 0 | 0 | 0 | 0 | 1 |
| CCDC94 | 1 | 0 | 0 | 0 | 0 | 0 | 0 | 0 | 1 |
| MGLL | 1 | 0 | 0 | 0 | 0 | 0 | 0 | 0 | 1 |
| NOSIP | 1 | 0 | 0 | 0 | 0 | 0 | 0 | 0 | 1 |
| PILRB | 1 | 0 | 0 | 0 | 0 | 0 | 0 | 0 | 1 |
| TMEM139 | 1 | 0 | 0 | 0 | 0 | 0 | 0 | 0 | 1 |
| HAPLN1 | 1 | 0 | 0 | 0 | 0 | 0 | 0 | 0 | 1 |
| PTAFR | 1 | 0 | 0 | 0 | 0 | 0 | 0 | 0 | 1 |
| TADA2A | 0 | 1 | 0 | 0 | 0 | 0 | 0 | 0 | 1 |
| PHYH | 0 | 0 | 0 | 0 | 1 | 0 | 0 | 0 | 1 |
| CRYBA4 | 1 | 0 | 0 | 0 | 0 | 0 | 0 | 0 | 1 |
| SLC25A20 | 1 | 0 | 0 | 0 | 0 | 0 | 0 | 0 | 1 |
| C1orf96 | 1 | 0 | 0 | 0 | 0 | 0 | 0 | 0 | 1 |
| EXOSC9 | 0 | 1 | 0 | 0 | 0 | 0 | 0 | 0 | 1 |
| RABAC1 | 1 | 0 | 0 | 0 | 0 | 0 | 0 | 0 | 1 |
| DNAL1 | 1 | 0 | 0 | 0 | 0 | 0 | 0 | 0 | 1 |
| ZBTB1 | 0 | 1 | 0 | 0 | 0 | 0 | 0 | 0 | 1 |
| IL11 | 1 | 0 | 0 | 0 | 0 | 0 | 0 | 0 | 1 |
| ARV1 | 1 | 0 | 0 | 0 | 0 | 0 | 0 | 0 | 1 |
| FAM19A2 | 1 | 0 | 0 | 0 | 0 | 0 | 0 | 0 | 1 |
| RG9MTD3 | 1 | 0 | 0 | 0 | 0 | 0 | 0 | 0 | 1 |
| STYK1 | 1 | 0 | 0 | 0 | 0 | 0 | 0 | 0 | 1 |
| ST6GALNAC4 | 1 | 0 | 0 | 0 | 0 | 0 | 0 | 0 | 1 |
| CAPZB | 1 | 0 | 0 | 0 | 0 | 0 | 0 | 0 | 1 |
| AICDA | 1 | 0 | 0 | 0 | 0 | 0 | 0 | 0 | 1 |
| RBM10 | 0 | 1 | 0 | 0 | 0 | 0 | 0 | 0 | 1 |
| GNPTG | 1 | 0 | 0 | 0 | 0 | 0 | 0 | 0 | 1 |
| SULT4A1 | 1 | 0 | 0 | 0 | 0 | 0 | 0 | 0 | 1 |
| PSMB1 | 1 | 0 | 0 | 0 | 0 | 0 | 0 | 0 | 1 |
| MAP6D1 | 1 | 0 | 0 | 0 | 0 | 0 | 0 | 0 | 1 |
| GNPDA2 | 1 | 0 | 0 | 0 | 0 | 0 | 0 | 0 | 1 |

| | | | | | | | | | |
|---|---|---|---|---|---|---|---|---|---|
| C5orf52 | 1 | 0 | 0 | 0 | 0 | 0 | 0 | 0 | 1 |
| PGLYRP1 | 1 | 0 | 0 | 0 | 0 | 0 | 0 | 0 | 1 |
| CABP2 | 1 | 0 | 0 | 0 | 0 | 0 | 0 | 0 | 1 |
| HOXD8 | 1 | 0 | 0 | 0 | 0 | 0 | 0 | 0 | 1 |
| RPS20 | 1 | 0 | 0 | 0 | 0 | 0 | 0 | 0 | 1 |
| HSPB9 | 1 | 0 | 0 | 0 | 0 | 0 | 0 | 0 | 1 |
| KLK15 | 1 | 0 | 0 | 0 | 0 | 0 | 0 | 0 | 1 |
| RCN2 | 1 | 0 | 0 | 0 | 0 | 0 | 0 | 0 | 1 |
| TFPI2 | 1 | 0 | 0 | 0 | 0 | 0 | 0 | 0 | 1 |
| FUT5 | 1 | 0 | 0 | 0 | 0 | 0 | 0 | 0 | 1 |
| THAP10 | 1 | 0 | 0 | 0 | 0 | 0 | 0 | 0 | 1 |
| C17orf49 | 1 | 0 | 0 | 0 | 0 | 0 | 0 | 0 | 1 |
| BNIP1 | 1 | 0 | 0 | 0 | 0 | 0 | 0 | 0 | 1 |
| TM4SF19 | 1 | 0 | 0 | 0 | 0 | 0 | 0 | 0 | 1 |
| DCN | 0 | 1 | 0 | 0 | 0 | 0 | 0 | 0 | 1 |
| FGF18 | 1 | 0 | 0 | 0 | 0 | 0 | 0 | 0 | 1 |
| CA13 | 1 | 0 | 0 | 0 | 0 | 0 | 0 | 0 | 1 |
| ODF1 | 1 | 0 | 0 | 0 | 0 | 0 | 0 | 0 | 1 |
| B3GAT2 | 1 | 0 | 0 | 0 | 0 | 0 | 0 | 0 | 1 |
| LIN28B | 1 | 0 | 0 | 0 | 0 | 0 | 0 | 0 | 1 |
| NT5C3L | 1 | 0 | 0 | 0 | 0 | 0 | 0 | 0 | 1 |
| C20orf11 | 1 | 0 | 0 | 0 | 0 | 0 | 0 | 0 | 1 |
| HIST1H1B | 1 | 0 | 0 | 0 | 0 | 0 | 0 | 0 | 1 |
| C12orf49 | 1 | 0 | 0 | 0 | 0 | 0 | 0 | 0 | 1 |
| RAP2A | 1 | 0 | 0 | 0 | 0 | 0 | 0 | 0 | 1 |
| C5AR1 | 1 | 0 | 0 | 0 | 0 | 0 | 0 | 0 | 1 |
| NDUFA5 | 1 | 0 | 0 | 0 | 0 | 0 | 0 | 0 | 1 |
| SLC25A19 | 1 | 0 | 0 | 0 | 0 | 0 | 0 | 0 | 1 |
| CHST6 | 1 | 0 | 0 | 0 | 0 | 0 | 0 | 0 | 1 |
| RNF212 | 1 | 0 | 0 | 0 | 0 | 0 | 0 | 0 | 1 |
| SDC4 | 1 | 0 | 0 | 0 | 0 | 0 | 0 | 0 | 1 |
| RNF185 | 1 | 0 | 0 | 0 | 0 | 0 | 0 | 0 | 1 |
| UBLCP1 | 1 | 0 | 0 | 0 | 0 | 0 | 0 | 0 | 1 |
| SNAP29 | 1 | 0 | 0 | 0 | 0 | 0 | 0 | 0 | 1 |
| LHFPL5 | 1 | 0 | 0 | 0 | 0 | 0 | 0 | 0 | 1 |
| COPS7A | 1 | 0 | 0 | 0 | 0 | 0 | 0 | 0 | 1 |
| TMEM86B | 1 | 0 | 0 | 0 | 0 | 0 | 0 | 0 | 1 |
| FAM18A | 1 | 0 | 0 | 0 | 0 | 0 | 0 | 0 | 1 |
| SLC25A23 | 1 | 0 | 0 | 0 | 0 | 0 | 0 | 0 | 1 |
| SRI | 1 | 0 | 0 | 0 | 0 | 0 | 0 | 0 | 1 |
| ESM1 | 1 | 0 | 0 | 0 | 0 | 0 | 0 | 0 | 1 |
| MIF4GD | 1 | 0 | 0 | 0 | 0 | 0 | 0 | 0 | 1 |
| CRH | 1 | 0 | 0 | 0 | 0 | 0 | 0 | 0 | 1 |
| CD247 | 1 | 0 | 0 | 0 | 0 | 0 | 0 | 0 | 1 |
| REPS2 | 0 | 0 | 1 | 0 | 0 | 0 | 0 | 0 | 1 |
| SDCBP2 | 1 | 0 | 0 | 0 | 0 | 0 | 0 | 0 | 1 |

| | | | | | | | | | |
|---|---|---|---|---|---|---|---|---|---|
| MTAP | 1 | 0 | 0 | 0 | 0 | 0 | 0 | 0 | 1 |
| METTL6 | 1 | 0 | 0 | 0 | 0 | 0 | 0 | 0 | 1 |
| DTNBP1 | 1 | 0 | 0 | 0 | 0 | 0 | 0 | 0 | 1 |
| GIMAP1 | 1 | 0 | 0 | 0 | 0 | 0 | 0 | 0 | 1 |
| FGF21 | 1 | 0 | 0 | 0 | 0 | 0 | 0 | 0 | 1 |
| CIAPIN1 | 1 | 0 | 0 | 0 | 0 | 0 | 0 | 0 | 1 |
| TTC33 | 1 | 0 | 0 | 0 | 0 | 0 | 0 | 0 | 1 |
| SNX24 | 1 | 0 | 0 | 0 | 0 | 0 | 0 | 0 | 1 |
| RCAN1 | 1 | 0 | 0 | 0 | 0 | 0 | 0 | 0 | 1 |
| PIGM | 1 | 0 | 0 | 0 | 0 | 0 | 0 | 0 | 1 |
| RPS8 | 1 | 0 | 0 | 0 | 0 | 0 | 0 | 0 | 1 |
| TSPAN6 | 1 | 0 | 0 | 0 | 0 | 0 | 0 | 0 | 1 |
| SRGN | 1 | 0 | 0 | 0 | 0 | 0 | 0 | 0 | 1 |
| WIBG | 1 | 0 | 0 | 0 | 0 | 0 | 0 | 0 | 1 |
| VWC2L | 1 | 0 | 0 | 0 | 0 | 0 | 0 | 0 | 1 |
| EIF2B5 | 0 | 1 | 0 | 0 | 0 | 0 | 0 | 0 | 1 |
| ARHGEF38 | 1 | 0 | 0 | 0 | 0 | 0 | 0 | 0 | 1 |
| INTS12 | 0 | 0 | 0 | 0 | 1 | 0 | 0 | 0 | 1 |
| NAT6 | 1 | 0 | 0 | 0 | 0 | 0 | 0 | 0 | 1 |
| C13orf33 | 1 | 0 | 0 | 0 | 0 | 0 | 0 | 0 | 1 |
| UBE2V2 | 1 | 0 | 0 | 0 | 0 | 0 | 0 | 0 | 1 |
| UFD1L | 1 | 0 | 0 | 0 | 0 | 0 | 0 | 0 | 1 |
| GTPBP8 | 1 | 0 | 0 | 0 | 0 | 0 | 0 | 0 | 1 |
| TMEM215 | 1 | 0 | 0 | 0 | 0 | 0 | 0 | 0 | 1 |
| HEMK1 | 1 | 0 | 0 | 0 | 0 | 0 | 0 | 0 | 1 |
| RNASE1 | 1 | 0 | 0 | 0 | 0 | 0 | 0 | 0 | 1 |
| AKR1B10 | 1 | 0 | 0 | 0 | 0 | 0 | 0 | 0 | 1 |
| GPX3 | 1 | 0 | 0 | 0 | 0 | 0 | 0 | 0 | 1 |
| DNAJB8 | 1 | 0 | 0 | 0 | 0 | 0 | 0 | 0 | 1 |
| GBP2 | 0 | 1 | 0 | 0 | 0 | 0 | 0 | 0 | 1 |
| SOST | 1 | 0 | 0 | 0 | 0 | 0 | 0 | 0 | 1 |
| MS4A6E | 1 | 0 | 0 | 0 | 0 | 0 | 0 | 0 | 1 |
| STRA8 | 1 | 0 | 0 | 0 | 0 | 0 | 0 | 0 | 1 |
| KLHDC8B | 1 | 0 | 0 | 0 | 0 | 0 | 0 | 0 | 1 |
| HSD17B3 | 1 | 0 | 0 | 0 | 0 | 0 | 0 | 0 | 1 |
| VSTM2A | 1 | 0 | 0 | 0 | 0 | 0 | 0 | 0 | 1 |
| C11orf65 | 1 | 0 | 0 | 0 | 0 | 0 | 0 | 0 | 1 |
| CYYR1 | 1 | 0 | 0 | 0 | 0 | 0 | 0 | 0 | 1 |
| IFNA6 | 1 | 0 | 0 | 0 | 0 | 0 | 0 | 0 | 1 |
| NECAP2 | 1 | 0 | 0 | 0 | 0 | 0 | 0 | 0 | 1 |
| BCL2L10 | 1 | 0 | 0 | 0 | 0 | 0 | 0 | 0 | 1 |
| CRHR2 | 1 | 0 | 0 | 0 | 0 | 0 | 0 | 0 | 1 |
| B9D1 | 1 | 0 | 0 | 0 | 0 | 0 | 0 | 0 | 1 |
| GNB2 | 1 | 0 | 0 | 0 | 0 | 0 | 0 | 0 | 1 |
| PISD | 1 | 0 | 0 | 0 | 0 | 0 | 0 | 0 | 1 |
| TBCC | 1 | 0 | 0 | 0 | 0 | 0 | 0 | 0 | 1 |

| | | | | | | | | | |
|---|---|---|---|---|---|---|---|---|---|
| FTHL17 | 1 | 0 | 0 | 0 | 0 | 0 | 0 | 0 | 1 |
| MMADHC | 1 | 0 | 0 | 0 | 0 | 0 | 0 | 0 | 1 |
| IMMP2L | 1 | 0 | 0 | 0 | 0 | 0 | 0 | 0 | 1 |
| DNAJB5 | 1 | 0 | 0 | 0 | 0 | 0 | 0 | 0 | 1 |
| C1orf201 | 1 | 0 | 0 | 0 | 0 | 0 | 0 | 0 | 1 |
| ACAP1 | 0 | 1 | 0 | 0 | 0 | 0 | 0 | 0 | 1 |
| GXYLT1 | 1 | 0 | 0 | 0 | 0 | 0 | 0 | 0 | 1 |
| KLRF1 | 1 | 0 | 0 | 0 | 0 | 0 | 0 | 0 | 1 |
| GPR182 | 1 | 0 | 0 | 0 | 0 | 0 | 0 | 0 | 1 |
| ZACN | 0 | 0 | 0 | 0 | 1 | 0 | 0 | 0 | 1 |
| TMEM11 | 1 | 0 | 0 | 0 | 0 | 0 | 0 | 0 | 1 |
| TANK | 1 | 0 | 0 | 0 | 0 | 0 | 0 | 0 | 1 |
| SFTPA1 | 1 | 0 | 0 | 0 | 0 | 0 | 0 | 0 | 1 |
| VBP1 | 1 | 0 | 0 | 0 | 0 | 0 | 0 | 0 | 1 |
| IL25 | 1 | 0 | 0 | 0 | 0 | 0 | 0 | 0 | 1 |
| ARMC1 | 1 | 0 | 0 | 0 | 0 | 0 | 0 | 0 | 1 |
| GJA4 | 1 | 0 | 0 | 0 | 0 | 0 | 0 | 0 | 1 |
| HSF1 | 1 | 0 | 0 | 0 | 0 | 0 | 0 | 0 | 1 |
| LBX1 | 1 | 0 | 0 | 0 | 0 | 0 | 0 | 0 | 1 |
| RBM4 | 1 | 0 | 0 | 0 | 0 | 0 | 0 | 0 | 1 |
| BCL7C | 1 | 0 | 0 | 0 | 0 | 0 | 0 | 0 | 1 |
| COMMD9 | 1 | 0 | 0 | 0 | 0 | 0 | 0 | 0 | 1 |
| SLC25A21 | 1 | 0 | 0 | 0 | 0 | 0 | 0 | 0 | 1 |
| C1orf187 | 1 | 0 | 0 | 0 | 0 | 0 | 0 | 0 | 1 |
| HIST1H4K | 1 | 0 | 0 | 0 | 0 | 0 | 0 | 0 | 1 |
| NPBWR2 | 1 | 0 | 0 | 0 | 0 | 0 | 0 | 0 | 1 |
| KCNJ15 | 1 | 0 | 0 | 0 | 0 | 0 | 0 | 0 | 1 |
| TNFRSF14 | 1 | 0 | 0 | 0 | 0 | 0 | 0 | 0 | 1 |
| SYNGR1 | 1 | 0 | 0 | 0 | 0 | 0 | 0 | 0 | 1 |
| RND1 | 1 | 0 | 0 | 0 | 0 | 0 | 0 | 0 | 1 |
| FAM92A1 | 1 | 0 | 0 | 0 | 0 | 0 | 0 | 0 | 1 |
| CYGB | 1 | 0 | 0 | 0 | 0 | 0 | 0 | 0 | 1 |
| C14orf79 | 1 | 0 | 0 | 0 | 0 | 0 | 0 | 0 | 1 |
| RND3 | 1 | 0 | 0 | 0 | 0 | 0 | 0 | 0 | 1 |
| GUCA1A | 1 | 0 | 0 | 0 | 0 | 0 | 0 | 0 | 1 |
| ADSS | 0 | 0 | 1 | 0 | 0 | 0 | 0 | 0 | 1 |
| CEP97 | 0 | 1 | 0 | 0 | 0 | 0 | 0 | 0 | 1 |
| ATP5O | 1 | 0 | 0 | 0 | 0 | 0 | 0 | 0 | 1 |
| NOL3 | 1 | 0 | 0 | 0 | 0 | 0 | 0 | 0 | 1 |
| HDAC2 | 1 | 0 | 0 | 0 | 0 | 0 | 0 | 0 | 1 |
| UBE2J2 | 1 | 0 | 0 | 0 | 0 | 0 | 0 | 0 | 1 |
| KCNK4 | 1 | 0 | 0 | 0 | 0 | 0 | 0 | 0 | 1 |
| YY2 | 1 | 0 | 0 | 0 | 0 | 0 | 0 | 0 | 1 |
| FAM180A | 1 | 0 | 0 | 0 | 0 | 0 | 0 | 0 | 1 |
| CPA4 | 1 | 0 | 0 | 0 | 0 | 0 | 0 | 0 | 1 |
| MCAM | 0 | 1 | 0 | 0 | 0 | 0 | 0 | 0 | 1 |

| | | | | | | | | | |
|---|---|---|---|---|---|---|---|---|---|
| NFYB | 1 | 0 | 0 | 0 | 0 | 0 | 0 | 0 | 1 |
| CYBRD1 | 1 | 0 | 0 | 0 | 0 | 0 | 0 | 0 | 1 |
| WRAP53 | 0 | 1 | 0 | 0 | 0 | 0 | 0 | 0 | 1 |
| SUSD3 | 1 | 0 | 0 | 0 | 0 | 0 | 0 | 0 | 1 |
| SDC2 | 1 | 0 | 0 | 0 | 0 | 0 | 0 | 0 | 1 |
| ACAT2 | 1 | 0 | 0 | 0 | 0 | 0 | 0 | 0 | 1 |
| S1PR4 | 1 | 0 | 0 | 0 | 0 | 0 | 0 | 0 | 1 |
| CLIC2 | 1 | 0 | 0 | 0 | 0 | 0 | 0 | 0 | 1 |
| ANXA5 | 1 | 0 | 0 | 0 | 0 | 0 | 0 | 0 | 1 |
| STOML3 | 0 | 1 | 0 | 0 | 0 | 0 | 0 | 0 | 1 |
| GTF2F2 | 1 | 0 | 0 | 0 | 0 | 0 | 0 | 0 | 1 |
| ETFDH | 0 | 1 | 0 | 0 | 0 | 0 | 0 | 0 | 1 |
| FAM82B | 1 | 0 | 0 | 0 | 0 | 0 | 0 | 0 | 1 |
| LPAR4 | 1 | 0 | 0 | 0 | 0 | 0 | 0 | 0 | 1 |
| CKB | 1 | 0 | 0 | 0 | 0 | 0 | 0 | 0 | 1 |
| SAAL1 | 1 | 0 | 0 | 0 | 0 | 0 | 0 | 0 | 1 |
| CCDC75 | 1 | 0 | 0 | 0 | 0 | 0 | 0 | 0 | 1 |
| LRRC55 | 1 | 0 | 0 | 0 | 0 | 0 | 0 | 0 | 1 |
| C11orf88 | 1 | 0 | 0 | 0 | 0 | 0 | 0 | 0 | 1 |
| PNRC1 | 1 | 0 | 0 | 0 | 0 | 0 | 0 | 0 | 1 |
| UPRT | 1 | 0 | 0 | 0 | 0 | 0 | 0 | 0 | 1 |
| ACTG2 | 1 | 0 | 0 | 0 | 0 | 0 | 0 | 0 | 1 |
| RAD23A | 1 | 0 | 0 | 0 | 0 | 0 | 0 | 0 | 1 |
| RWDD1 | 1 | 0 | 0 | 0 | 0 | 0 | 0 | 0 | 1 |
| MTX2 | 1 | 0 | 0 | 0 | 0 | 0 | 0 | 0 | 1 |
| GZMH | 1 | 0 | 0 | 0 | 0 | 0 | 0 | 0 | 1 |
| TMEM64 | 0 | 0 | 0 | 0 | 1 | 0 | 0 | 0 | 1 |
| ELOVL7 | 1 | 0 | 0 | 0 | 0 | 0 | 0 | 0 | 1 |
| C7orf45 | 1 | 0 | 0 | 0 | 0 | 0 | 0 | 0 | 1 |
| FUT4 | 0 | 1 | 0 | 0 | 0 | 0 | 0 | 0 | 1 |
| SGCG | 1 | 0 | 0 | 0 | 0 | 0 | 0 | 0 | 1 |
| TWSG1 | 1 | 0 | 0 | 0 | 0 | 0 | 0 | 0 | 1 |
| TMEM176B | 1 | 0 | 0 | 0 | 0 | 0 | 0 | 0 | 1 |
| ATP6AP2 | 1 | 0 | 0 | 0 | 0 | 0 | 0 | 0 | 1 |
| MESP1 | 1 | 0 | 0 | 0 | 0 | 0 | 0 | 0 | 1 |
| GPR137C | 1 | 0 | 0 | 0 | 0 | 0 | 0 | 0 | 1 |
| OR5C1 | 1 | 0 | 0 | 0 | 0 | 0 | 0 | 0 | 1 |
| SFXN1 | 1 | 0 | 0 | 0 | 0 | 0 | 0 | 0 | 1 |
| CCR8 | 1 | 0 | 0 | 0 | 0 | 0 | 0 | 0 | 1 |
| C4orf49 | 1 | 0 | 0 | 0 | 0 | 0 | 0 | 0 | 1 |
| ERRFI1 | 1 | 0 | 0 | 0 | 0 | 0 | 0 | 0 | 1 |
| CLEC4A | 1 | 0 | 0 | 0 | 0 | 0 | 0 | 0 | 1 |
| C4orf3 | 1 | 0 | 0 | 0 | 0 | 0 | 0 | 0 | 1 |
| U2AF1 | 1 | 0 | 0 | 0 | 0 | 0 | 0 | 0 | 1 |
| GATAD1 | 1 | 0 | 0 | 0 | 0 | 0 | 0 | 0 | 1 |
| C5orf44 | 1 | 0 | 0 | 0 | 0 | 0 | 0 | 0 | 1 |

| | | | | | | | | | |
|---|---|---|---|---|---|---|---|---|---|
| SNRPB | 1 | 0 | 0 | 0 | 0 | 0 | 0 | 0 | 1 |
| NXPH3 | 1 | 0 | 0 | 0 | 0 | 0 | 0 | 0 | 1 |
| BET3L | 1 | 0 | 0 | 0 | 0 | 0 | 0 | 0 | 1 |
| CXCR2 | 1 | 0 | 0 | 0 | 0 | 0 | 0 | 0 | 1 |
| TNNI2 | 1 | 0 | 0 | 0 | 0 | 0 | 0 | 0 | 1 |
| HIST1H1D | 1 | 0 | 0 | 0 | 0 | 0 | 0 | 0 | 1 |
| ZDHHC23 | 1 | 0 | 0 | 0 | 0 | 0 | 0 | 0 | 1 |
| IL18BP | 1 | 0 | 0 | 0 | 0 | 0 | 0 | 0 | 1 |
| NKX3-2 | 1 | 0 | 0 | 0 | 0 | 0 | 0 | 0 | 1 |
| BTN3A2 | 1 | 0 | 0 | 0 | 0 | 0 | 0 | 0 | 1 |
| FAM170B | 1 | 0 | 0 | 0 | 0 | 0 | 0 | 0 | 1 |
| ELOVL3 | 1 | 0 | 0 | 0 | 0 | 0 | 0 | 0 | 1 |
| DAND5 | 1 | 0 | 0 | 0 | 0 | 0 | 0 | 0 | 1 |
| CD40LG | 1 | 0 | 0 | 0 | 0 | 0 | 0 | 0 | 1 |
| POLDIP2 | 1 | 0 | 0 | 0 | 0 | 0 | 0 | 0 | 1 |
| FOLR1 | 1 | 0 | 0 | 0 | 0 | 0 | 0 | 0 | 1 |
| STK16 | 1 | 0 | 0 | 0 | 0 | 0 | 0 | 0 | 1 |
| C22orf23 | 1 | 0 | 0 | 0 | 0 | 0 | 0 | 0 | 1 |
| CCR1 | 1 | 0 | 0 | 0 | 0 | 0 | 0 | 0 | 1 |
| BAMBI | 1 | 0 | 0 | 0 | 0 | 0 | 0 | 0 | 1 |
| ISG20 | 1 | 0 | 0 | 0 | 0 | 0 | 0 | 0 | 1 |
| TRIM40 | 1 | 0 | 0 | 0 | 0 | 0 | 0 | 0 | 1 |
| ZNF670 | 1 | 0 | 0 | 0 | 0 | 0 | 0 | 0 | 1 |
| DCUN1D4 | 1 | 0 | 0 | 0 | 0 | 0 | 0 | 0 | 1 |
| GPR35 | 1 | 0 | 0 | 0 | 0 | 0 | 0 | 0 | 1 |
| RBM34 | 1 | 0 | 0 | 0 | 0 | 0 | 0 | 0 | 1 |
| SNAI1 | 1 | 0 | 0 | 0 | 0 | 0 | 0 | 0 | 1 |
| FAM20B | 1 | 0 | 0 | 0 | 0 | 0 | 0 | 0 | 1 |
| WNT7A | 1 | 0 | 0 | 0 | 0 | 0 | 0 | 0 | 1 |
| INMT | 1 | 0 | 0 | 0 | 0 | 0 | 0 | 0 | 1 |
| MLST8 | 1 | 0 | 0 | 0 | 0 | 0 | 0 | 0 | 1 |
| CKM | 1 | 0 | 0 | 0 | 0 | 0 | 0 | 0 | 1 |
| STX11 | 1 | 0 | 0 | 0 | 0 | 0 | 0 | 0 | 1 |
| EMD | 1 | 0 | 0 | 0 | 0 | 0 | 0 | 0 | 1 |
| CXorf66 | 1 | 0 | 0 | 0 | 0 | 0 | 0 | 0 | 1 |
| TSPAN2 | 1 | 0 | 0 | 0 | 0 | 0 | 0 | 0 | 1 |
| ROGDI | 1 | 0 | 0 | 0 | 0 | 0 | 0 | 0 | 1 |
| C5orf47 | 1 | 0 | 0 | 0 | 0 | 0 | 0 | 0 | 1 |
| SOCS4 | 1 | 0 | 0 | 0 | 0 | 0 | 0 | 0 | 1 |
| C10orf62 | 1 | 0 | 0 | 0 | 0 | 0 | 0 | 0 | 1 |
| SGCA | 1 | 0 | 0 | 0 | 0 | 0 | 0 | 0 | 1 |
| TRAF7 | 0 | 1 | 0 | 0 | 0 | 0 | 0 | 0 | 1 |
| BPHL | 1 | 0 | 0 | 0 | 0 | 0 | 0 | 0 | 1 |
| ZNF514 | 1 | 0 | 0 | 0 | 0 | 0 | 0 | 0 | 1 |
| HSPB2 | 1 | 0 | 0 | 0 | 0 | 0 | 0 | 0 | 1 |
| TPD52 | 1 | 0 | 0 | 0 | 0 | 0 | 0 | 0 | 1 |

| | | | | | | | | | |
|---|---|---|---|---|---|---|---|---|---|
| KCTD13 | 1 | 0 | 0 | 0 | 0 | 0 | 0 | 0 | 1 |
| STX1B | 1 | 0 | 0 | 0 | 0 | 0 | 0 | 0 | 1 |
| ATP5H | 1 | 0 | 0 | 0 | 0 | 0 | 0 | 0 | 1 |
| CLUAP1 | 1 | 0 | 0 | 0 | 0 | 0 | 0 | 0 | 1 |
| PTTG2 | 1 | 0 | 0 | 0 | 0 | 0 | 0 | 0 | 1 |
| GPR114 | 0 | 0 | 0 | 0 | 1 | 0 | 0 | 0 | 1 |
| NAGK | 1 | 0 | 0 | 0 | 0 | 0 | 0 | 0 | 1 |
| MRGPRG | 1 | 0 | 0 | 0 | 0 | 0 | 0 | 0 | 1 |
| SFT2D2 | 1 | 0 | 0 | 0 | 0 | 0 | 0 | 0 | 1 |
| NKAPL | 1 | 0 | 0 | 0 | 0 | 0 | 0 | 0 | 1 |
| LDLRAD1 | 1 | 0 | 0 | 0 | 0 | 0 | 0 | 0 | 1 |
| C19orf18 | 1 | 0 | 0 | 0 | 0 | 0 | 0 | 0 | 1 |
| ALKBH7 | 1 | 0 | 0 | 0 | 0 | 0 | 0 | 0 | 1 |
| GZMB | 1 | 0 | 0 | 0 | 0 | 0 | 0 | 0 | 1 |
| AANAT | 1 | 0 | 0 | 0 | 0 | 0 | 0 | 0 | 1 |
| PSMA3 | 1 | 0 | 0 | 0 | 0 | 0 | 0 | 0 | 1 |
| HEY1 | 1 | 0 | 0 | 0 | 0 | 0 | 0 | 0 | 1 |
| CSE1L | 0 | 1 | 0 | 0 | 0 | 0 | 0 | 0 | 1 |
| PPARD | 1 | 0 | 0 | 0 | 0 | 0 | 0 | 0 | 1 |
| RWDD3 | 1 | 0 | 0 | 0 | 0 | 0 | 0 | 0 | 1 |
| TEAD1 | 1 | 0 | 0 | 0 | 0 | 0 | 0 | 0 | 1 |
| TAF10 | 1 | 0 | 0 | 0 | 0 | 0 | 0 | 0 | 1 |
| CAMK4 | 1 | 0 | 0 | 0 | 0 | 0 | 0 | 0 | 1 |
| PIP4K2B | 1 | 0 | 0 | 0 | 0 | 0 | 0 | 0 | 1 |
| CHRNA6 | 1 | 0 | 0 | 0 | 0 | 0 | 0 | 0 | 1 |
| OSTF1 | 1 | 0 | 0 | 0 | 0 | 0 | 0 | 0 | 1 |
| NRIP2 | 1 | 0 | 0 | 0 | 0 | 0 | 0 | 0 | 1 |
| NOS1AP | 1 | 0 | 0 | 0 | 0 | 0 | 0 | 0 | 1 |
| ABHD8 | 1 | 0 | 0 | 0 | 0 | 0 | 0 | 0 | 1 |
| CUTC | 1 | 0 | 0 | 0 | 0 | 0 | 0 | 0 | 1 |
| GOPC | 0 | 0 | 0 | 0 | 1 | 0 | 0 | 0 | 1 |
| ARHGAP11B | 1 | 0 | 0 | 0 | 0 | 0 | 0 | 0 | 1 |
| GTF2B | 1 | 0 | 0 | 0 | 0 | 0 | 0 | 0 | 1 |
| SYP | 1 | 0 | 0 | 0 | 0 | 0 | 0 | 0 | 1 |
| TMEM106C | 1 | 0 | 0 | 0 | 0 | 0 | 0 | 0 | 1 |
| DLX4 | 1 | 0 | 0 | 0 | 0 | 0 | 0 | 0 | 1 |
| IKBKB | 0 | 1 | 0 | 0 | 0 | 0 | 0 | 0 | 1 |
| SMYD5 | 1 | 0 | 0 | 0 | 0 | 0 | 0 | 0 | 1 |
| SYAP1 | 1 | 0 | 0 | 0 | 0 | 0 | 0 | 0 | 1 |
| DPAGT1 | 1 | 0 | 0 | 0 | 0 | 0 | 0 | 0 | 1 |
| YBX1 | 1 | 0 | 0 | 0 | 0 | 0 | 0 | 0 | 1 |
| RASL12 | 1 | 0 | 0 | 0 | 0 | 0 | 0 | 0 | 1 |
| TNFRSF10B | 1 | 0 | 0 | 0 | 0 | 0 | 0 | 0 | 1 |
| MRGPRE | 1 | 0 | 0 | 0 | 0 | 0 | 0 | 0 | 1 |
| ACMSD | 1 | 0 | 0 | 0 | 0 | 0 | 0 | 0 | 1 |
| LACE1 | 1 | 0 | 0 | 0 | 0 | 0 | 0 | 0 | 1 |

| | | | | | | | | | |
|---|---|---|---|---|---|---|---|---|---|
| NEU2 | 1 | 0 | 0 | 0 | 0 | 0 | 0 | 0 | 1 |
| NCALD | 1 | 0 | 0 | 0 | 0 | 0 | 0 | 0 | 1 |
| NNMT | 1 | 0 | 0 | 0 | 0 | 0 | 0 | 0 | 1 |
| GDE1 | 1 | 0 | 0 | 0 | 0 | 0 | 0 | 0 | 1 |
| NIPA1 | 1 | 0 | 0 | 0 | 0 | 0 | 0 | 0 | 1 |
| WNT3 | 1 | 0 | 0 | 0 | 0 | 0 | 0 | 0 | 1 |
| FGL1 | 1 | 0 | 0 | 0 | 0 | 0 | 0 | 0 | 1 |
| DNASE1L1 | 1 | 0 | 0 | 0 | 0 | 0 | 0 | 0 | 1 |
| ARL15 | 1 | 0 | 0 | 0 | 0 | 0 | 0 | 0 | 1 |
| POLR1C | 1 | 0 | 0 | 0 | 0 | 0 | 0 | 0 | 1 |
| DDX1 | 0 | 0 | 1 | 0 | 0 | 0 | 0 | 0 | 1 |
| RNF144A | 1 | 0 | 0 | 0 | 0 | 0 | 0 | 0 | 1 |
| COPE | 1 | 0 | 0 | 0 | 0 | 0 | 0 | 0 | 1 |
| PLEK | 1 | 0 | 0 | 0 | 0 | 0 | 0 | 0 | 1 |
| RELL2 | 1 | 0 | 0 | 0 | 0 | 0 | 0 | 0 | 1 |
| SLC38A2 | 1 | 0 | 0 | 0 | 0 | 0 | 0 | 0 | 1 |
| TMEM81 | 1 | 0 | 0 | 0 | 0 | 0 | 0 | 0 | 1 |
| HDAC3 | 1 | 0 | 0 | 0 | 0 | 0 | 0 | 0 | 1 |
| MRAS | 1 | 0 | 0 | 0 | 0 | 0 | 0 | 0 | 1 |
| PSMB11 | 1 | 0 | 0 | 0 | 0 | 0 | 0 | 0 | 1 |
| MMP20 | 1 | 0 | 0 | 0 | 0 | 0 | 0 | 0 | 1 |
| RIBC2 | 1 | 0 | 0 | 0 | 0 | 0 | 0 | 0 | 1 |
| ANGPTL3 | 1 | 0 | 0 | 0 | 0 | 0 | 0 | 0 | 1 |
| HHLA2 | 0 | 1 | 0 | 0 | 0 | 0 | 0 | 0 | 1 |
| EFNB1 | 1 | 0 | 0 | 0 | 0 | 0 | 0 | 0 | 1 |
| RAB5B | 1 | 0 | 0 | 0 | 0 | 0 | 0 | 0 | 1 |
| PAQR3 | 1 | 0 | 0 | 0 | 0 | 0 | 0 | 0 | 1 |
| GEMIN7 | 1 | 0 | 0 | 0 | 0 | 0 | 0 | 0 | 1 |
| DEFB127 | 1 | 0 | 0 | 0 | 0 | 0 | 0 | 0 | 1 |
| ZMAT3 | 1 | 0 | 0 | 0 | 0 | 0 | 0 | 0 | 1 |
| DNAJA4 | 1 | 0 | 0 | 0 | 0 | 0 | 0 | 0 | 1 |
| CWC25 | 1 | 0 | 0 | 0 | 0 | 0 | 0 | 0 | 1 |
| IFT27 | 1 | 0 | 0 | 0 | 0 | 0 | 0 | 0 | 1 |
| FGF5 | 1 | 0 | 0 | 0 | 0 | 0 | 0 | 0 | 1 |
| C10orf81 | 1 | 0 | 0 | 0 | 0 | 0 | 0 | 0 | 1 |
| PECR | 1 | 0 | 0 | 0 | 0 | 0 | 0 | 0 | 1 |
| SPATA4 | 1 | 0 | 0 | 0 | 0 | 0 | 0 | 0 | 1 |
| DCPS | 1 | 0 | 0 | 0 | 0 | 0 | 0 | 0 | 1 |
| IHH | 1 | 0 | 0 | 0 | 0 | 0 | 0 | 0 | 1 |
| GNAZ | 1 | 0 | 0 | 0 | 0 | 0 | 0 | 0 | 1 |
| CCDC153 | 1 | 0 | 0 | 0 | 0 | 0 | 0 | 0 | 1 |
| C10orf55 | 1 | 0 | 0 | 0 | 0 | 0 | 0 | 0 | 1 |
| NCF4 | 1 | 0 | 0 | 0 | 0 | 0 | 0 | 0 | 1 |
| FAM108B1 | 1 | 0 | 0 | 0 | 0 | 0 | 0 | 0 | 1 |
| C1QB | 1 | 0 | 0 | 0 | 0 | 0 | 0 | 0 | 1 |
| UCN3 | 1 | 0 | 0 | 0 | 0 | 0 | 0 | 0 | 1 |

| | | | | | | | | | |
|---|---|---|---|---|---|---|---|---|---|
| TPSG1 | 1 | 0 | 0 | 0 | 0 | 0 | 0 | 0 | 1 |
| HMBS | 1 | 0 | 0 | 0 | 0 | 0 | 0 | 0 | 1 |
| RTP3 | 1 | 0 | 0 | 0 | 0 | 0 | 0 | 0 | 1 |
| C19orf38 | 1 | 0 | 0 | 0 | 0 | 0 | 0 | 0 | 1 |
| RASSF7 | 0 | 1 | 0 | 0 | 0 | 0 | 0 | 0 | 1 |
| EIF2B2 | 1 | 0 | 0 | 0 | 0 | 0 | 0 | 0 | 1 |
| ICAM2 | 1 | 0 | 0 | 0 | 0 | 0 | 0 | 0 | 1 |
| FBXO45 | 1 | 0 | 0 | 0 | 0 | 0 | 0 | 0 | 1 |
| PDCD1LG2 | 1 | 0 | 0 | 0 | 0 | 0 | 0 | 0 | 1 |
| GALK1 | 1 | 0 | 0 | 0 | 0 | 0 | 0 | 0 | 1 |
| ASB1 | 1 | 0 | 0 | 0 | 0 | 0 | 0 | 0 | 1 |
| KRTAP6-3 | 1 | 0 | 0 | 0 | 0 | 0 | 0 | 0 | 1 |
| TMEM69 | 1 | 0 | 0 | 0 | 0 | 0 | 0 | 0 | 1 |
| ALDH1A3 | 1 | 0 | 0 | 0 | 0 | 0 | 0 | 0 | 1 |
| FAM69A | 1 | 0 | 0 | 0 | 0 | 0 | 0 | 0 | 1 |
| MRPL32 | 1 | 0 | 0 | 0 | 0 | 0 | 0 | 0 | 1 |
| SCRN1 | 1 | 0 | 0 | 0 | 0 | 0 | 0 | 0 | 1 |
| EBI3 | 1 | 0 | 0 | 0 | 0 | 0 | 0 | 0 | 1 |
| RCN3 | 1 | 0 | 0 | 0 | 0 | 0 | 0 | 0 | 1 |
| JUN | 1 | 0 | 0 | 0 | 0 | 0 | 0 | 0 | 1 |
| GPR148 | 1 | 0 | 0 | 0 | 0 | 0 | 0 | 0 | 1 |
| KCNMB2 | 1 | 0 | 0 | 0 | 0 | 0 | 0 | 0 | 1 |
| KCNK17 | 1 | 0 | 0 | 0 | 0 | 0 | 0 | 0 | 1 |
| PRR5 | 1 | 0 | 0 | 0 | 0 | 0 | 0 | 0 | 1 |
| CHCHD3 | 1 | 0 | 0 | 0 | 0 | 0 | 0 | 0 | 1 |
| CENPQ | 1 | 0 | 0 | 0 | 0 | 0 | 0 | 0 | 1 |
| STARD6 | 1 | 0 | 0 | 0 | 0 | 0 | 0 | 0 | 1 |
| KCTD14 | 1 | 0 | 0 | 0 | 0 | 0 | 0 | 0 | 1 |
| MAGEB2 | 1 | 0 | 0 | 0 | 0 | 0 | 0 | 0 | 1 |
| ART5 | 1 | 0 | 0 | 0 | 0 | 0 | 0 | 0 | 1 |
| LYPD4 | 1 | 0 | 0 | 0 | 0 | 0 | 0 | 0 | 1 |
| SVOP | 1 | 0 | 0 | 0 | 0 | 0 | 0 | 0 | 1 |
| C9orf139 | 1 | 0 | 0 | 0 | 0 | 0 | 0 | 0 | 1 |
| DOK5 | 0 | 1 | 0 | 0 | 0 | 0 | 0 | 0 | 1 |
| SP4 | 0 | 0 | 0 | 0 | 1 | 0 | 0 | 0 | 1 |
| TNFRSF10C | 1 | 0 | 0 | 0 | 0 | 0 | 0 | 0 | 1 |
| PEMT | 1 | 0 | 0 | 0 | 0 | 0 | 0 | 0 | 1 |
| DCTPP1 | 1 | 0 | 0 | 0 | 0 | 0 | 0 | 0 | 1 |
| SCG5 | 1 | 0 | 0 | 0 | 0 | 0 | 0 | 0 | 1 |
| RBM17 | 1 | 0 | 0 | 0 | 0 | 0 | 0 | 0 | 1 |
| ANKRA2 | 1 | 0 | 0 | 0 | 0 | 0 | 0 | 0 | 1 |
| P2RY6 | 1 | 0 | 0 | 0 | 0 | 0 | 0 | 0 | 1 |
| CNOT8 | 1 | 0 | 0 | 0 | 0 | 0 | 0 | 0 | 1 |
| KIAA0513 | 1 | 0 | 0 | 0 | 0 | 0 | 0 | 0 | 1 |
| TSGA13 | 1 | 0 | 0 | 0 | 0 | 0 | 0 | 0 | 1 |
| PTGIR | 1 | 0 | 0 | 0 | 0 | 0 | 0 | 0 | 1 |

| | | | | | | | | | |
|---|---|---|---|---|---|---|---|---|---|
| NFKBIA | 1 | 0 | 0 | 0 | 0 | 0 | 0 | 0 | 1 |
| KRTAP13-3 | 1 | 0 | 0 | 0 | 0 | 0 | 0 | 0 | 1 |
| HAGH | 1 | 0 | 0 | 0 | 0 | 0 | 0 | 0 | 1 |
| GSX2 | 1 | 0 | 0 | 0 | 0 | 0 | 0 | 0 | 1 |
| HYI | 1 | 0 | 0 | 0 | 0 | 0 | 0 | 0 | 1 |
| NPRL2 | 1 | 0 | 0 | 0 | 0 | 0 | 0 | 0 | 1 |
| KCNJ14 | 1 | 0 | 0 | 0 | 0 | 0 | 0 | 0 | 1 |
| CCDC104 | 1 | 0 | 0 | 0 | 0 | 0 | 0 | 0 | 1 |
| LZTFL1 | 1 | 0 | 0 | 0 | 0 | 0 | 0 | 0 | 1 |
| MORC4 | 0 | 0 | 1 | 0 | 0 | 0 | 0 | 0 | 1 |
| AGPHD1 | 1 | 0 | 0 | 0 | 0 | 0 | 0 | 0 | 1 |
| SERHL2 | 1 | 0 | 0 | 0 | 0 | 0 | 0 | 0 | 1 |
| ELOVL6 | 1 | 0 | 0 | 0 | 0 | 0 | 0 | 0 | 1 |
| ARGLU1 | 1 | 0 | 0 | 0 | 0 | 0 | 0 | 0 | 1 |
| STX5 | 1 | 0 | 0 | 0 | 0 | 0 | 0 | 0 | 1 |
| PPT1 | 1 | 0 | 0 | 0 | 0 | 0 | 0 | 0 | 1 |
| OLIG3 | 1 | 0 | 0 | 0 | 0 | 0 | 0 | 0 | 1 |
| BID | 1 | 0 | 0 | 0 | 0 | 0 | 0 | 0 | 1 |
| SIAH1 | 1 | 0 | 0 | 0 | 0 | 0 | 0 | 0 | 1 |
| GALM | 1 | 0 | 0 | 0 | 0 | 0 | 0 | 0 | 1 |
| INSL6 | 1 | 0 | 0 | 0 | 0 | 0 | 0 | 0 | 1 |
| LDHC | 1 | 0 | 0 | 0 | 0 | 0 | 0 | 0 | 1 |
| BTF3 | 1 | 0 | 0 | 0 | 0 | 0 | 0 | 0 | 1 |
| NARS2 | 1 | 0 | 0 | 0 | 0 | 0 | 0 | 0 | 1 |
| CHST1 | 1 | 0 | 0 | 0 | 0 | 0 | 0 | 0 | 1 |
| GPR45 | 1 | 0 | 0 | 0 | 0 | 0 | 0 | 0 | 1 |
| SLC25A44 | 1 | 0 | 0 | 0 | 0 | 0 | 0 | 0 | 1 |
| EXOSC3 | 1 | 0 | 0 | 0 | 0 | 0 | 0 | 0 | 1 |
| C19orf47 | 1 | 0 | 0 | 0 | 0 | 0 | 0 | 0 | 1 |
| UHMK1 | 1 | 0 | 0 | 0 | 0 | 0 | 0 | 0 | 1 |
| DPM1 | 1 | 0 | 0 | 0 | 0 | 0 | 0 | 0 | 1 |
| RPL22L1 | 1 | 0 | 0 | 0 | 0 | 0 | 0 | 0 | 1 |
| OMD | 1 | 0 | 0 | 0 | 0 | 0 | 0 | 0 | 1 |
| CITED1 | 1 | 0 | 0 | 0 | 0 | 0 | 0 | 0 | 1 |
| C11orf53 | 1 | 0 | 0 | 0 | 0 | 0 | 0 | 0 | 1 |
| C12orf10 | 1 | 0 | 0 | 0 | 0 | 0 | 0 | 0 | 1 |
| STEAP1 | 1 | 0 | 0 | 0 | 0 | 0 | 0 | 0 | 1 |
| RHOC | 1 | 0 | 0 | 0 | 0 | 0 | 0 | 0 | 1 |
| SPATA22 | 1 | 0 | 0 | 0 | 0 | 0 | 0 | 0 | 1 |
| DPH2 | 1 | 0 | 0 | 0 | 0 | 0 | 0 | 0 | 1 |
| ZNF268 | 1 | 0 | 0 | 0 | 0 | 0 | 0 | 0 | 1 |
| ANKRD1 | 1 | 0 | 0 | 0 | 0 | 0 | 0 | 0 | 1 |
| QPCTL | 1 | 0 | 0 | 0 | 0 | 0 | 0 | 0 | 1 |
| ZSCAN5A | 0 | 1 | 0 | 0 | 0 | 0 | 0 | 0 | 1 |
| WNT4 | 0 | 0 | 0 | 0 | 1 | 0 | 0 | 0 | 1 |
| LCN10 | 1 | 0 | 0 | 0 | 0 | 0 | 0 | 0 | 1 |

| | | | | | | | | | |
|---|---|---|---|---|---|---|---|---|---|
| WNT11 | 1 | 0 | 0 | 0 | 0 | 0 | 0 | 0 | 1 |
| CAMLG | 1 | 0 | 0 | 0 | 0 | 0 | 0 | 0 | 1 |
| CST5 | 1 | 0 | 0 | 0 | 0 | 0 | 0 | 0 | 1 |
| TMEM101 | 1 | 0 | 0 | 0 | 0 | 0 | 0 | 0 | 1 |
| FNDC4 | 1 | 0 | 0 | 0 | 0 | 0 | 0 | 0 | 1 |
| CCDC110 | 0 | 1 | 0 | 0 | 0 | 0 | 0 | 0 | 1 |
| C7orf57 | 1 | 0 | 0 | 0 | 0 | 0 | 0 | 0 | 1 |
| RAB21 | 1 | 0 | 0 | 0 | 0 | 0 | 0 | 0 | 1 |
| AHCY | 1 | 0 | 0 | 0 | 0 | 0 | 0 | 0 | 1 |
| CYB561D1 | 1 | 0 | 0 | 0 | 0 | 0 | 0 | 0 | 1 |
| CHCHD7 | 1 | 0 | 0 | 0 | 0 | 0 | 0 | 0 | 1 |
| OR13D1 | 1 | 0 | 0 | 0 | 0 | 0 | 0 | 0 | 1 |
| FAM166A | 1 | 0 | 0 | 0 | 0 | 0 | 0 | 0 | 1 |
| VTI1B | 1 | 0 | 0 | 0 | 0 | 0 | 0 | 0 | 1 |
| CXorf41 | 1 | 0 | 0 | 0 | 0 | 0 | 0 | 0 | 1 |
| FKBP3 | 1 | 0 | 0 | 0 | 0 | 0 | 0 | 0 | 1 |
| DEPDC4 | 1 | 0 | 0 | 0 | 0 | 0 | 0 | 0 | 1 |
| TGM4 | 1 | 0 | 0 | 0 | 0 | 0 | 0 | 0 | 1 |
| AMELX | 1 | 0 | 0 | 0 | 0 | 0 | 0 | 0 | 1 |
| KCNJ11 | 0 | 0 | 0 | 1 | 0 | 0 | 0 | 0 | 1 |
| MAGEA1 | 1 | 0 | 0 | 0 | 0 | 0 | 0 | 0 | 1 |
| TSKU | 1 | 0 | 0 | 0 | 0 | 0 | 0 | 0 | 1 |
| MTHFS | 1 | 0 | 0 | 0 | 0 | 0 | 0 | 0 | 1 |
| RIPPLY2 | 1 | 0 | 0 | 0 | 0 | 0 | 0 | 0 | 1 |
| CCDC122 | 1 | 0 | 0 | 0 | 0 | 0 | 0 | 0 | 1 |
| ASB12 | 1 | 0 | 0 | 0 | 0 | 0 | 0 | 0 | 1 |
| C20orf79 | 1 | 0 | 0 | 0 | 0 | 0 | 0 | 0 | 1 |
| GNA15 | 1 | 0 | 0 | 0 | 0 | 0 | 0 | 0 | 1 |
| TOMM34 | 1 | 0 | 0 | 0 | 0 | 0 | 0 | 0 | 1 |
| TSSC4 | 1 | 0 | 0 | 0 | 0 | 0 | 0 | 0 | 1 |
| SIRT4 | 1 | 0 | 0 | 0 | 0 | 0 | 0 | 0 | 1 |
| PCOLCE | 1 | 0 | 0 | 0 | 0 | 0 | 0 | 0 | 1 |
| ZBTB43 | 1 | 0 | 0 | 0 | 0 | 0 | 0 | 0 | 1 |
| LYPD6B | 1 | 0 | 0 | 0 | 0 | 0 | 0 | 0 | 1 |
| TMEM38B | 1 | 0 | 0 | 0 | 0 | 0 | 0 | 0 | 1 |
| DNAJB9 | 1 | 0 | 0 | 0 | 0 | 0 | 0 | 0 | 1 |
| OR10A6 | 1 | 0 | 0 | 0 | 0 | 0 | 0 | 0 | 1 |
| OR3A2 | 1 | 0 | 0 | 0 | 0 | 0 | 0 | 0 | 1 |
| CXXC4 | 1 | 0 | 0 | 0 | 0 | 0 | 0 | 0 | 1 |
| SLC35D2 | 1 | 0 | 0 | 0 | 0 | 0 | 0 | 0 | 1 |
| CHCHD6 | 1 | 0 | 0 | 0 | 0 | 0 | 0 | 0 | 1 |
| CACNG6 | 1 | 0 | 0 | 0 | 0 | 0 | 0 | 0 | 1 |
| C20orf43 | 1 | 0 | 0 | 0 | 0 | 0 | 0 | 0 | 1 |
| ODF3L1 | 1 | 0 | 0 | 0 | 0 | 0 | 0 | 0 | 1 |
| SPESP1 | 1 | 0 | 0 | 0 | 0 | 0 | 0 | 0 | 1 |
| SIX3 | 1 | 0 | 0 | 0 | 0 | 0 | 0 | 0 | 1 |

| | | | | | | | | | |
|---|---|---|---|---|---|---|---|---|---|
| GLT6D1 | 1 | 0 | 0 | 0 | 0 | 0 | 0 | 0 | 1 |
| PHF13 | 1 | 0 | 0 | 0 | 0 | 0 | 0 | 0 | 1 |
| TMED8 | 1 | 0 | 0 | 0 | 0 | 0 | 0 | 0 | 1 |
| JMJD7 | 1 | 0 | 0 | 0 | 0 | 0 | 0 | 0 | 1 |
| NFAM1 | 1 | 0 | 0 | 0 | 0 | 0 | 0 | 0 | 1 |
| MTCH2 | 1 | 0 | 0 | 0 | 0 | 0 | 0 | 0 | 1 |
| HIST1H2AA | 1 | 0 | 0 | 0 | 0 | 0 | 0 | 0 | 1 |
| ATXN7L3 | 1 | 0 | 0 | 0 | 0 | 0 | 0 | 0 | 1 |
| VPS37A | 1 | 0 | 0 | 0 | 0 | 0 | 0 | 0 | 1 |
| ZNF664 | 1 | 0 | 0 | 0 | 0 | 0 | 0 | 0 | 1 |
| PSMA2 | 1 | 0 | 0 | 0 | 0 | 0 | 0 | 0 | 1 |
| GALP | 1 | 0 | 0 | 0 | 0 | 0 | 0 | 0 | 1 |
| FAM187B | 1 | 0 | 0 | 0 | 0 | 0 | 0 | 0 | 1 |
| LNP1 | 1 | 0 | 0 | 0 | 0 | 0 | 0 | 0 | 1 |
| SNUPN | 1 | 0 | 0 | 0 | 0 | 0 | 0 | 0 | 1 |
| HEXIM2 | 1 | 0 | 0 | 0 | 0 | 0 | 0 | 0 | 1 |
| PDXK | 1 | 0 | 0 | 0 | 0 | 0 | 0 | 0 | 1 |
| CLEC3B | 1 | 0 | 0 | 0 | 0 | 0 | 0 | 0 | 1 |
| VSTM2L | 1 | 0 | 0 | 0 | 0 | 0 | 0 | 0 | 1 |
| TOMM40 | 1 | 0 | 0 | 0 | 0 | 0 | 0 | 0 | 1 |
| ARHGAP22 | 0 | 1 | 0 | 0 | 0 | 0 | 0 | 0 | 1 |
| ATL3 | 1 | 0 | 0 | 0 | 0 | 0 | 0 | 0 | 1 |
| RPAP2 | 0 | 0 | 0 | 0 | 1 | 0 | 0 | 0 | 1 |
| MED8 | 1 | 0 | 0 | 0 | 0 | 0 | 0 | 0 | 1 |
| OR14I1 | 1 | 0 | 0 | 0 | 0 | 0 | 0 | 0 | 1 |
| OR1L1 | 1 | 0 | 0 | 0 | 0 | 0 | 0 | 0 | 1 |
| CA14 | 1 | 0 | 0 | 0 | 0 | 0 | 0 | 0 | 1 |
| HTR6 | 1 | 0 | 0 | 0 | 0 | 0 | 0 | 0 | 1 |
| AGPAT6 | 1 | 0 | 0 | 0 | 0 | 0 | 0 | 0 | 1 |
| NTSR1 | 1 | 0 | 0 | 0 | 0 | 0 | 0 | 0 | 1 |
| SLC35D3 | 1 | 0 | 0 | 0 | 0 | 0 | 0 | 0 | 1 |
| TMEM72 | 1 | 0 | 0 | 0 | 0 | 0 | 0 | 0 | 1 |
| SLC16A1 | 1 | 0 | 0 | 0 | 0 | 0 | 0 | 0 | 1 |
| GRIPAP1 | 0 | 1 | 0 | 0 | 0 | 0 | 0 | 0 | 1 |
| C10orf10 | 1 | 0 | 0 | 0 | 0 | 0 | 0 | 0 | 1 |
| ZNF776 | 0 | 1 | 0 | 0 | 0 | 0 | 0 | 0 | 1 |
| CLDN12 | 1 | 0 | 0 | 0 | 0 | 0 | 0 | 0 | 1 |
| SLC25A4 | 1 | 0 | 0 | 0 | 0 | 0 | 0 | 0 | 1 |
| NDUFS3 | 1 | 0 | 0 | 0 | 0 | 0 | 0 | 0 | 1 |
| UCHL1 | 1 | 0 | 0 | 0 | 0 | 0 | 0 | 0 | 1 |
| ZNF187 | 1 | 0 | 0 | 0 | 0 | 0 | 0 | 0 | 1 |
| EDN1 | 1 | 0 | 0 | 0 | 0 | 0 | 0 | 0 | 1 |
| IL17REL | 1 | 0 | 0 | 0 | 0 | 0 | 0 | 0 | 1 |
| ASTE1 | 1 | 0 | 0 | 0 | 0 | 0 | 0 | 0 | 1 |
| CLECL1 | 1 | 0 | 0 | 0 | 0 | 0 | 0 | 0 | 1 |
| IGFBP4 | 1 | 0 | 0 | 0 | 0 | 0 | 0 | 0 | 1 |

| | | | | | | | | | |
|---|---|---|---|---|---|---|---|---|---|
| ZSCAN21 | 1 | 0 | 0 | 0 | 0 | 0 | 0 | 0 | 1 |
| ADI1 | 0 | 0 | 1 | 0 | 0 | 0 | 0 | 0 | 1 |
| RNF44 | 1 | 0 | 0 | 0 | 0 | 0 | 0 | 0 | 1 |
| CRHBP | 1 | 0 | 0 | 0 | 0 | 0 | 0 | 0 | 1 |
| CENPH | 1 | 0 | 0 | 0 | 0 | 0 | 0 | 0 | 1 |
| PPP1R1B | 1 | 0 | 0 | 0 | 0 | 0 | 0 | 0 | 1 |
| ZNF843 | 1 | 0 | 0 | 0 | 0 | 0 | 0 | 0 | 1 |
| STX4 | 1 | 0 | 0 | 0 | 0 | 0 | 0 | 0 | 1 |
| HCCS | 1 | 0 | 0 | 0 | 0 | 0 | 0 | 0 | 1 |
| OR2Y1 | 1 | 0 | 0 | 0 | 0 | 0 | 0 | 0 | 1 |
| SERTAD1 | 1 | 0 | 0 | 0 | 0 | 0 | 0 | 0 | 1 |
| MSI1 | 1 | 0 | 0 | 0 | 0 | 0 | 0 | 0 | 1 |
| OR8D4 | 1 | 0 | 0 | 0 | 0 | 0 | 0 | 0 | 1 |
| DYNC2LI1 | 1 | 0 | 0 | 0 | 0 | 0 | 0 | 0 | 1 |
| TMPRSS12 | 1 | 0 | 0 | 0 | 0 | 0 | 0 | 0 | 1 |
| DTWD2 | 1 | 0 | 0 | 0 | 0 | 0 | 0 | 0 | 1 |
| GNA13 | 1 | 0 | 0 | 0 | 0 | 0 | 0 | 0 | 1 |
| FAM181A | 1 | 0 | 0 | 0 | 0 | 0 | 0 | 0 | 1 |
| FAM83E | 1 | 0 | 0 | 0 | 0 | 0 | 0 | 0 | 1 |
| IL13RA2 | 1 | 0 | 0 | 0 | 0 | 0 | 0 | 0 | 1 |
| PAK1IP1 | 1 | 0 | 0 | 0 | 0 | 0 | 0 | 0 | 1 |
| LRRC17 | 1 | 0 | 0 | 0 | 0 | 0 | 0 | 0 | 1 |
| CC2D2B | 1 | 0 | 0 | 0 | 0 | 0 | 0 | 0 | 1 |
| NAT9 | 1 | 0 | 0 | 0 | 0 | 0 | 0 | 0 | 1 |
| CCDC103 | 1 | 0 | 0 | 0 | 0 | 0 | 0 | 0 | 1 |
| CNPY4 | 1 | 0 | 0 | 0 | 0 | 0 | 0 | 0 | 1 |
| H2AFY | 1 | 0 | 0 | 0 | 0 | 0 | 0 | 0 | 1 |
| KIAA1704 | 1 | 0 | 0 | 0 | 0 | 0 | 0 | 0 | 1 |
| LIN28A | 1 | 0 | 0 | 0 | 0 | 0 | 0 | 0 | 1 |
| OR2B6 | 1 | 0 | 0 | 0 | 0 | 0 | 0 | 0 | 1 |
| FRZB | 1 | 0 | 0 | 0 | 0 | 0 | 0 | 0 | 1 |
| PRSS33 | 1 | 0 | 0 | 0 | 0 | 0 | 0 | 0 | 1 |
| ITLN2 | 1 | 0 | 0 | 0 | 0 | 0 | 0 | 0 | 1 |
| S100PBP | 1 | 0 | 0 | 0 | 0 | 0 | 0 | 0 | 1 |
| DUSP11 | 1 | 0 | 0 | 0 | 0 | 0 | 0 | 0 | 1 |
| ICAM3 | 1 | 0 | 0 | 0 | 0 | 0 | 0 | 0 | 1 |
| RALBP1 | 0 | 1 | 0 | 0 | 0 | 0 | 0 | 0 | 1 |
| ELOVL2 | 1 | 0 | 0 | 0 | 0 | 0 | 0 | 0 | 1 |
| C22orf42 | 1 | 0 | 0 | 0 | 0 | 0 | 0 | 0 | 1 |
| POU3F1 | 1 | 0 | 0 | 0 | 0 | 0 | 0 | 0 | 1 |
| FOLR2 | 1 | 0 | 0 | 0 | 0 | 0 | 0 | 0 | 1 |
| VIM | 1 | 0 | 0 | 0 | 0 | 0 | 0 | 0 | 1 |
| DUS4L | 1 | 0 | 0 | 0 | 0 | 0 | 0 | 0 | 1 |
| NEK7 | 1 | 0 | 0 | 0 | 0 | 0 | 0 | 0 | 1 |
| PKMYT1 | 1 | 0 | 0 | 0 | 0 | 0 | 0 | 0 | 1 |
| PRRG1 | 1 | 0 | 0 | 0 | 0 | 0 | 0 | 0 | 1 |

| | | | | | | | | | |
|---|---|---|---|---|---|---|---|---|---|
| ZNF259 | 1 | 0 | 0 | 0 | 0 | 0 | 0 | 0 | 1 |
| COMMD10 | 1 | 0 | 0 | 0 | 0 | 0 | 0 | 0 | 1 |
| MOSPD2 | 1 | 0 | 0 | 0 | 0 | 0 | 0 | 0 | 1 |
| PARP2 | 0 | 1 | 0 | 0 | 0 | 0 | 0 | 0 | 1 |
| TADA1 | 1 | 0 | 0 | 0 | 0 | 0 | 0 | 0 | 1 |
| CITED2 | 1 | 0 | 0 | 0 | 0 | 0 | 0 | 0 | 1 |
| ZCRB1 | 1 | 0 | 0 | 0 | 0 | 0 | 0 | 0 | 1 |
| CNTD1 | 1 | 0 | 0 | 0 | 0 | 0 | 0 | 0 | 1 |
| GPR32 | 1 | 0 | 0 | 0 | 0 | 0 | 0 | 0 | 1 |
| OR4D1 | 1 | 0 | 0 | 0 | 0 | 0 | 0 | 0 | 1 |
| C20orf173 | 1 | 0 | 0 | 0 | 0 | 0 | 0 | 0 | 1 |
| SLA2 | 1 | 0 | 0 | 0 | 0 | 0 | 0 | 0 | 1 |
| ARL1 | 1 | 0 | 0 | 0 | 0 | 0 | 0 | 0 | 1 |
| ZFAND1 | 1 | 0 | 0 | 0 | 0 | 0 | 0 | 0 | 1 |
| ING3 | 1 | 0 | 0 | 0 | 0 | 0 | 0 | 0 | 1 |
| APOA1 | 1 | 0 | 0 | 0 | 0 | 0 | 0 | 0 | 1 |
| C14orf149 | 1 | 0 | 0 | 0 | 0 | 0 | 0 | 0 | 1 |
| POLR3G | 1 | 0 | 0 | 0 | 0 | 0 | 0 | 0 | 1 |
| TPRG1L | 1 | 0 | 0 | 0 | 0 | 0 | 0 | 0 | 1 |
| TRIM63 | 1 | 0 | 0 | 0 | 0 | 0 | 0 | 0 | 1 |
| PGPEP1L | 1 | 0 | 0 | 0 | 0 | 0 | 0 | 0 | 1 |
| PPIL6 | 1 | 0 | 0 | 0 | 0 | 0 | 0 | 0 | 1 |
| DRGX | 1 | 0 | 0 | 0 | 0 | 0 | 0 | 0 | 1 |
| TMEM220 | 1 | 0 | 0 | 0 | 0 | 0 | 0 | 0 | 1 |
| EIF3M | 1 | 0 | 0 | 0 | 0 | 0 | 0 | 0 | 1 |
| RPL8 | 1 | 0 | 0 | 0 | 0 | 0 | 0 | 0 | 1 |
| TMEM198 | 1 | 0 | 0 | 0 | 0 | 0 | 0 | 0 | 1 |
| ERLIN2 | 1 | 0 | 0 | 0 | 0 | 0 | 0 | 0 | 1 |
| TSC22D4 | 1 | 0 | 0 | 0 | 0 | 0 | 0 | 0 | 1 |
| SLC7A6 | 1 | 0 | 0 | 0 | 0 | 0 | 0 | 0 | 1 |
| GNAT3 | 1 | 0 | 0 | 0 | 0 | 0 | 0 | 0 | 1 |
| CCND1 | 1 | 0 | 0 | 0 | 0 | 0 | 0 | 0 | 1 |
| YIF1A | 1 | 0 | 0 | 0 | 0 | 0 | 0 | 0 | 1 |
| MS4A7 | 1 | 0 | 0 | 0 | 0 | 0 | 0 | 0 | 1 |
| ERGIC2 | 1 | 0 | 0 | 0 | 0 | 0 | 0 | 0 | 1 |
| BTLA | 1 | 0 | 0 | 0 | 0 | 0 | 0 | 0 | 1 |
| C1orf55 | 1 | 0 | 0 | 0 | 0 | 0 | 0 | 0 | 1 |
| NPTX2 | 1 | 0 | 0 | 0 | 0 | 0 | 0 | 0 | 1 |
| HRAS | 1 | 0 | 0 | 0 | 0 | 0 | 0 | 0 | 1 |
| LYSMD4 | 1 | 0 | 0 | 0 | 0 | 0 | 0 | 0 | 1 |
| BLVRA | 1 | 0 | 0 | 0 | 0 | 0 | 0 | 0 | 1 |
| ISYNA1 | 1 | 0 | 0 | 0 | 0 | 0 | 0 | 0 | 1 |
| CYR61 | 1 | 0 | 0 | 0 | 0 | 0 | 0 | 0 | 1 |
| PCYT2 | 1 | 0 | 0 | 0 | 0 | 0 | 0 | 0 | 1 |
| CPM | 1 | 0 | 0 | 0 | 0 | 0 | 0 | 0 | 1 |
| TMEM19 | 1 | 0 | 0 | 0 | 0 | 0 | 0 | 0 | 1 |

| | | | | | | | | | |
|---|---|---|---|---|---|---|---|---|---|
| GREM2 | 1 | 0 | 0 | 0 | 0 | 0 | 0 | 0 | 1 |
| HPD | 1 | 0 | 0 | 0 | 0 | 0 | 0 | 0 | 1 |
| CLOCK | 0 | 1 | 0 | 0 | 0 | 0 | 0 | 0 | 1 |
| AADAC | 1 | 0 | 0 | 0 | 0 | 0 | 0 | 0 | 1 |
| ECHDC3 | 1 | 0 | 0 | 0 | 0 | 0 | 0 | 0 | 1 |
| PSME3 | 1 | 0 | 0 | 0 | 0 | 0 | 0 | 0 | 1 |
| DNAJC3 | 1 | 0 | 0 | 0 | 0 | 0 | 0 | 0 | 1 |
| ARID3B | 1 | 0 | 0 | 0 | 0 | 0 | 0 | 0 | 1 |
| SLC35C1 | 1 | 0 | 0 | 0 | 0 | 0 | 0 | 0 | 1 |
| XRRA1 | 0 | 0 | 1 | 0 | 0 | 0 | 0 | 0 | 1 |
| RLBP1 | 1 | 0 | 0 | 0 | 0 | 0 | 0 | 0 | 1 |
| IDH3A | 1 | 0 | 0 | 0 | 0 | 0 | 0 | 0 | 1 |
| MS4A15 | 1 | 0 | 0 | 0 | 0 | 0 | 0 | 0 | 1 |
| CHRNB2 | 1 | 0 | 0 | 0 | 0 | 0 | 0 | 0 | 1 |
| TMEM200B | 1 | 0 | 0 | 0 | 0 | 0 | 0 | 0 | 1 |
| AP3M2 | 1 | 0 | 0 | 0 | 0 | 0 | 0 | 0 | 1 |
| FGF17 | 1 | 0 | 0 | 0 | 0 | 0 | 0 | 0 | 1 |
| KLHL8 | 1 | 0 | 0 | 0 | 0 | 0 | 0 | 0 | 1 |
| CDX2 | 1 | 0 | 0 | 0 | 0 | 0 | 0 | 0 | 1 |
| NDRG4 | 1 | 0 | 0 | 0 | 0 | 0 | 0 | 0 | 1 |
| ZNF580 | 1 | 0 | 0 | 0 | 0 | 0 | 0 | 0 | 1 |
| ACTN1 | 0 | 0 | 1 | 0 | 0 | 0 | 0 | 0 | 1 |
| SLC25A28 | 1 | 0 | 0 | 0 | 0 | 0 | 0 | 0 | 1 |
| MTX3 | 1 | 0 | 0 | 0 | 0 | 0 | 0 | 0 | 1 |
| OR4D2 | 1 | 0 | 0 | 0 | 0 | 0 | 0 | 0 | 1 |
| SLC10A1 | 1 | 0 | 0 | 0 | 0 | 0 | 0 | 0 | 1 |
| ADPRHL1 | 1 | 0 | 0 | 0 | 0 | 0 | 0 | 0 | 1 |
| GALE | 1 | 0 | 0 | 0 | 0 | 0 | 0 | 0 | 1 |
| ETF1 | 1 | 0 | 0 | 0 | 0 | 0 | 0 | 0 | 1 |
| RNASEH2B | 1 | 0 | 0 | 0 | 0 | 0 | 0 | 0 | 1 |
| CCDC106 | 1 | 0 | 0 | 0 | 0 | 0 | 0 | 0 | 1 |
| TMEM54 | 1 | 0 | 0 | 0 | 0 | 0 | 0 | 0 | 1 |
| POFUT2 | 1 | 0 | 0 | 0 | 0 | 0 | 0 | 0 | 1 |
| HAUS4 | 1 | 0 | 0 | 0 | 0 | 0 | 0 | 0 | 1 |
| TOB1 | 1 | 0 | 0 | 0 | 0 | 0 | 0 | 0 | 1 |
| FZR1 | 1 | 0 | 0 | 0 | 0 | 0 | 0 | 0 | 1 |
| FCN3 | 1 | 0 | 0 | 0 | 0 | 0 | 0 | 0 | 1 |
| PHF12 | 0 | 0 | 1 | 0 | 0 | 0 | 0 | 0 | 1 |
| EDNRB | 1 | 0 | 0 | 0 | 0 | 0 | 0 | 0 | 1 |
| FAM69B | 1 | 0 | 0 | 0 | 0 | 0 | 0 | 0 | 1 |
| C1orf68 | 1 | 0 | 0 | 0 | 0 | 0 | 0 | 0 | 1 |
| MAPKAPK2 | 1 | 0 | 0 | 0 | 0 | 0 | 0 | 0 | 1 |
| GATAD2B | 1 | 0 | 0 | 0 | 0 | 0 | 0 | 0 | 1 |
| RNF208 | 1 | 0 | 0 | 0 | 0 | 0 | 0 | 0 | 1 |
| DBP | 1 | 0 | 0 | 0 | 0 | 0 | 0 | 0 | 1 |
| CDNF | 1 | 0 | 0 | 0 | 0 | 0 | 0 | 0 | 1 |

| | | | | | | | | | |
|---|---|---|---|---|---|---|---|---|---|
| PPP1CA | 1 | 0 | 0 | 0 | 0 | 0 | 0 | 0 | 1 |
| STX12 | 1 | 0 | 0 | 0 | 0 | 0 | 0 | 0 | 1 |
| ERGIC3 | 1 | 0 | 0 | 0 | 0 | 0 | 0 | 0 | 1 |
| SEC61A2 | 1 | 0 | 0 | 0 | 0 | 0 | 0 | 0 | 1 |
| SLC29A2 | 1 | 0 | 0 | 0 | 0 | 0 | 0 | 0 | 1 |
| MORF4L2 | 1 | 0 | 0 | 0 | 0 | 0 | 0 | 0 | 1 |
| WDR55 | 1 | 0 | 0 | 0 | 0 | 0 | 0 | 0 | 1 |
| C11orf91 | 1 | 0 | 0 | 0 | 0 | 0 | 0 | 0 | 1 |
| C22orf25 | 1 | 0 | 0 | 0 | 0 | 0 | 0 | 0 | 1 |
| TELO2 | 0 | 1 | 0 | 0 | 0 | 0 | 0 | 0 | 1 |
| CADM4 | 1 | 0 | 0 | 0 | 0 | 0 | 0 | 0 | 1 |
| PCYT1A | 1 | 0 | 0 | 0 | 0 | 0 | 0 | 0 | 1 |
| CCL7 | 1 | 0 | 0 | 0 | 0 | 0 | 0 | 0 | 1 |
| TSFM | 1 | 0 | 0 | 0 | 0 | 0 | 0 | 0 | 1 |
| MPI | 1 | 0 | 0 | 0 | 0 | 0 | 0 | 0 | 1 |
| CLDN18 | 1 | 0 | 0 | 0 | 0 | 0 | 0 | 0 | 1 |
| C1QBP | 1 | 0 | 0 | 0 | 0 | 0 | 0 | 0 | 1 |
| E2F4 | 1 | 0 | 0 | 0 | 0 | 0 | 0 | 0 | 1 |
| CLDN10 | 1 | 0 | 0 | 0 | 0 | 0 | 0 | 0 | 1 |
| MRPS35 | 1 | 0 | 0 | 0 | 0 | 0 | 0 | 0 | 1 |
| RASSF3 | 1 | 0 | 0 | 0 | 0 | 0 | 0 | 0 | 1 |
| FAM83G | 0 | 1 | 0 | 0 | 0 | 0 | 0 | 0 | 1 |
| NUP37 | 1 | 0 | 0 | 0 | 0 | 0 | 0 | 0 | 1 |
| CLEC17A | 1 | 0 | 0 | 0 | 0 | 0 | 0 | 0 | 1 |
| SPCS2 | 1 | 0 | 0 | 0 | 0 | 0 | 0 | 0 | 1 |
| CCDC130 | 1 | 0 | 0 | 0 | 0 | 0 | 0 | 0 | 1 |
| FOSB | 1 | 0 | 0 | 0 | 0 | 0 | 0 | 0 | 1 |
| SLC6A2 | 0 | 0 | 1 | 0 | 0 | 0 | 0 | 0 | 1 |
| KLF14 | 1 | 0 | 0 | 0 | 0 | 0 | 0 | 0 | 1 |
| TNFSF14 | 1 | 0 | 0 | 0 | 0 | 0 | 0 | 0 | 1 |
| C12orf74 | 1 | 0 | 0 | 0 | 0 | 0 | 0 | 0 | 1 |
| ITGB1BP3 | 1 | 0 | 0 | 0 | 0 | 0 | 0 | 0 | 1 |
| CRLF3 | 1 | 0 | 0 | 0 | 0 | 0 | 0 | 0 | 1 |
| OR5AR1 | 1 | 0 | 0 | 0 | 0 | 0 | 0 | 0 | 1 |
| RNF26 | 1 | 0 | 0 | 0 | 0 | 0 | 0 | 0 | 1 |
| TMEM184A | 1 | 0 | 0 | 0 | 0 | 0 | 0 | 0 | 1 |
| CCR7 | 1 | 0 | 0 | 0 | 0 | 0 | 0 | 0 | 1 |
| KRTAP5-5 | 1 | 0 | 0 | 0 | 0 | 0 | 0 | 0 | 1 |
| SELV | 1 | 0 | 0 | 0 | 0 | 0 | 0 | 0 | 1 |
| DHDDS | 1 | 0 | 0 | 0 | 0 | 0 | 0 | 0 | 1 |
| P2RY4 | 1 | 0 | 0 | 0 | 0 | 0 | 0 | 0 | 1 |
| LYPD3 | 1 | 0 | 0 | 0 | 0 | 0 | 0 | 0 | 1 |
| C16orf70 | 1 | 0 | 0 | 0 | 0 | 0 | 0 | 0 | 1 |
| OAT | 1 | 0 | 0 | 0 | 0 | 0 | 0 | 0 | 1 |
| LRRC61 | 1 | 0 | 0 | 0 | 0 | 0 | 0 | 0 | 1 |
| CDKL3 | 1 | 0 | 0 | 0 | 0 | 0 | 0 | 0 | 1 |

| | | | | | | | | | |
|---|---|---|---|---|---|---|---|---|---|
| SRM | 1 | 0 | 0 | 0 | 0 | 0 | 0 | 0 | 1 |
| LRRC2 | 1 | 0 | 0 | 0 | 0 | 0 | 0 | 0 | 1 |
| TRIM29 | 1 | 0 | 0 | 0 | 0 | 0 | 0 | 0 | 1 |
| CBR3 | 1 | 0 | 0 | 0 | 0 | 0 | 0 | 0 | 1 |
| PIGA | 1 | 0 | 0 | 0 | 0 | 0 | 0 | 0 | 1 |
| COQ9 | 1 | 0 | 0 | 0 | 0 | 0 | 0 | 0 | 1 |
| NDEL1 | 1 | 0 | 0 | 0 | 0 | 0 | 0 | 0 | 1 |
| PIGB | 0 | 0 | 0 | 0 | 1 | 0 | 0 | 0 | 1 |
| EPS8L1 | 0 | 1 | 0 | 0 | 0 | 0 | 0 | 0 | 1 |
| TBRG1 | 1 | 0 | 0 | 0 | 0 | 0 | 0 | 0 | 1 |
| GPR132 | 1 | 0 | 0 | 0 | 0 | 0 | 0 | 0 | 1 |
| DLX6 | 1 | 0 | 0 | 0 | 0 | 0 | 0 | 0 | 1 |
| HTR2B | 1 | 0 | 0 | 0 | 0 | 0 | 0 | 0 | 1 |
| RHAG | 1 | 0 | 0 | 0 | 0 | 0 | 0 | 0 | 1 |
| LARP4B | 0 | 1 | 0 | 0 | 0 | 0 | 0 | 0 | 1 |
| TBX10 | 1 | 0 | 0 | 0 | 0 | 0 | 0 | 0 | 1 |
| CCDC47 | 1 | 0 | 0 | 0 | 0 | 0 | 0 | 0 | 1 |
| FAM81A | 1 | 0 | 0 | 0 | 0 | 0 | 0 | 0 | 1 |
| SAMD8 | 1 | 0 | 0 | 0 | 0 | 0 | 0 | 0 | 1 |
| TMEM59 | 1 | 0 | 0 | 0 | 0 | 0 | 0 | 0 | 1 |
| UGDH | 1 | 0 | 0 | 0 | 0 | 0 | 0 | 0 | 1 |
| WASF2 | 0 | 0 | 0 | 0 | 1 | 0 | 0 | 0 | 1 |
| CALR3 | 1 | 0 | 0 | 0 | 0 | 0 | 0 | 0 | 1 |
| CPSF4L | 1 | 0 | 0 | 0 | 0 | 0 | 0 | 0 | 1 |
| KDELR2 | 1 | 0 | 0 | 0 | 0 | 0 | 0 | 0 | 1 |
| BAX | 1 | 0 | 0 | 0 | 0 | 0 | 0 | 0 | 1 |
| CXADR | 1 | 0 | 0 | 0 | 0 | 0 | 0 | 0 | 1 |
| CAMKV | 1 | 0 | 0 | 0 | 0 | 0 | 0 | 0 | 1 |
| HMGB4 | 1 | 0 | 0 | 0 | 0 | 0 | 0 | 0 | 1 |
| LRRC69 | 1 | 0 | 0 | 0 | 0 | 0 | 0 | 0 | 1 |
| ADAP2 | 1 | 0 | 0 | 0 | 0 | 0 | 0 | 0 | 1 |
| GPR6 | 1 | 0 | 0 | 0 | 0 | 0 | 0 | 0 | 1 |
| MEF2A | 1 | 0 | 0 | 0 | 0 | 0 | 0 | 0 | 1 |
| DNAJC12 | 1 | 0 | 0 | 0 | 0 | 0 | 0 | 0 | 1 |
| KCNN2 | 0 | 1 | 0 | 0 | 0 | 0 | 0 | 0 | 1 |
| IGFBP1 | 1 | 0 | 0 | 0 | 0 | 0 | 0 | 0 | 1 |
| ATPAF2 | 1 | 0 | 0 | 0 | 0 | 0 | 0 | 0 | 1 |
| GDNF | 1 | 0 | 0 | 0 | 0 | 0 | 0 | 0 | 1 |
| MAT2A | 1 | 0 | 0 | 0 | 0 | 0 | 0 | 0 | 1 |
| PFKFB4 | 1 | 0 | 0 | 0 | 0 | 0 | 0 | 0 | 1 |
| B4GALT5 | 1 | 0 | 0 | 0 | 0 | 0 | 0 | 0 | 1 |
| NCAPH2 | 1 | 0 | 0 | 0 | 0 | 0 | 0 | 0 | 1 |
| HLA-C | 1 | 0 | 0 | 0 | 0 | 0 | 0 | 0 | 1 |
| LIME1 | 1 | 0 | 0 | 0 | 0 | 0 | 0 | 0 | 1 |
| IL1B | 1 | 0 | 0 | 0 | 0 | 0 | 0 | 0 | 1 |
| OR4D6 | 1 | 0 | 0 | 0 | 0 | 0 | 0 | 0 | 1 |

| | | | | | | | | | |
|---|---|---|---|---|---|---|---|---|---|
| SYK | 1 | 0 | 0 | 0 | 0 | 0 | 0 | 0 | 1 |
| HDHD3 | 1 | 0 | 0 | 0 | 0 | 0 | 0 | 0 | 1 |
| IQCJ | 1 | 0 | 0 | 0 | 0 | 0 | 0 | 0 | 1 |
| PAIP1 | 1 | 0 | 0 | 0 | 0 | 0 | 0 | 0 | 1 |
| CANT1 | 1 | 0 | 0 | 0 | 0 | 0 | 0 | 0 | 1 |
| E2F6 | 1 | 0 | 0 | 0 | 0 | 0 | 0 | 0 | 1 |
| CDX1 | 1 | 0 | 0 | 0 | 0 | 0 | 0 | 0 | 1 |
| FOXA3 | 1 | 0 | 0 | 0 | 0 | 0 | 0 | 0 | 1 |
| RNF217 | 1 | 0 | 0 | 0 | 0 | 0 | 0 | 0 | 1 |
| RAB36 | 1 | 0 | 0 | 0 | 0 | 0 | 0 | 0 | 1 |
| GNB1 | 1 | 0 | 0 | 0 | 0 | 0 | 0 | 0 | 1 |
| CRKL | 1 | 0 | 0 | 0 | 0 | 0 | 0 | 0 | 1 |
| RFPL4B | 1 | 0 | 0 | 0 | 0 | 0 | 0 | 0 | 1 |
| GOT1 | 1 | 0 | 0 | 0 | 0 | 0 | 0 | 0 | 1 |
| SLC30A6 | 1 | 0 | 0 | 0 | 0 | 0 | 0 | 0 | 1 |
| GPN3 | 1 | 0 | 0 | 0 | 0 | 0 | 0 | 0 | 1 |
| ELAC1 | 1 | 0 | 0 | 0 | 0 | 0 | 0 | 0 | 1 |
| SGCZ | 1 | 0 | 0 | 0 | 0 | 0 | 0 | 0 | 1 |
| LDHAL6A | 1 | 0 | 0 | 0 | 0 | 0 | 0 | 0 | 1 |
| KCTD17 | 1 | 0 | 0 | 0 | 0 | 0 | 0 | 0 | 1 |
| SPDYE4 | 1 | 0 | 0 | 0 | 0 | 0 | 0 | 0 | 1 |
| ZFAND3 | 1 | 0 | 0 | 0 | 0 | 0 | 0 | 0 | 1 |
| SGCD | 1 | 0 | 0 | 0 | 0 | 0 | 0 | 0 | 1 |
| TCP1 | 1 | 0 | 0 | 0 | 0 | 0 | 0 | 0 | 1 |
| PENK | 1 | 0 | 0 | 0 | 0 | 0 | 0 | 0 | 1 |
| LIN7A | 1 | 0 | 0 | 0 | 0 | 0 | 0 | 0 | 1 |
| CRYBB1 | 1 | 0 | 0 | 0 | 0 | 0 | 0 | 0 | 1 |
| FGF2 | 1 | 0 | 0 | 0 | 0 | 0 | 0 | 0 | 1 |
| NUBPL | 1 | 0 | 0 | 0 | 0 | 0 | 0 | 0 | 1 |
| RMND5B | 1 | 0 | 0 | 0 | 0 | 0 | 0 | 0 | 1 |
| CACNG1 | 1 | 0 | 0 | 0 | 0 | 0 | 0 | 0 | 1 |
| SPN | 1 | 0 | 0 | 0 | 0 | 0 | 0 | 0 | 1 |
| RRAGD | 1 | 0 | 0 | 0 | 0 | 0 | 0 | 0 | 1 |
| ZNF287 | 0 | 1 | 0 | 0 | 0 | 0 | 0 | 0 | 1 |
| ATPAF1 | 1 | 0 | 0 | 0 | 0 | 0 | 0 | 0 | 1 |
| AGTR1 | 1 | 0 | 0 | 0 | 0 | 0 | 0 | 0 | 1 |
| CDK2 | 1 | 0 | 0 | 0 | 0 | 0 | 0 | 0 | 1 |
| GNB3 | 1 | 0 | 0 | 0 | 0 | 0 | 0 | 0 | 1 |
| LGALS8 | 1 | 0 | 0 | 0 | 0 | 0 | 0 | 0 | 1 |
| NUB1 | 1 | 0 | 0 | 0 | 0 | 0 | 0 | 0 | 1 |
| KRT23 | 1 | 0 | 0 | 0 | 0 | 0 | 0 | 0 | 1 |
| TTC9B | 1 | 0 | 0 | 0 | 0 | 0 | 0 | 0 | 1 |
| ENPP4 | 1 | 0 | 0 | 0 | 0 | 0 | 0 | 0 | 1 |
| LSM11 | 1 | 0 | 0 | 0 | 0 | 0 | 0 | 0 | 1 |
| POU6F1 | 1 | 0 | 0 | 0 | 0 | 0 | 0 | 0 | 1 |
| BPGM | 1 | 0 | 0 | 0 | 0 | 0 | 0 | 0 | 1 |

| | | | | | | | | | |
|---|---|---|---|---|---|---|---|---|---|
| PACSIN2 | 1 | 0 | 0 | 0 | 0 | 0 | 0 | 0 | 1 |
| CEACAM1 | 1 | 0 | 0 | 0 | 0 | 0 | 0 | 0 | 1 |
| PRPF38B | 1 | 0 | 0 | 0 | 0 | 0 | 0 | 0 | 1 |
| OR1J1 | 1 | 0 | 0 | 0 | 0 | 0 | 0 | 0 | 1 |
| P2RY14 | 1 | 0 | 0 | 0 | 0 | 0 | 0 | 0 | 1 |
| CAPG | 1 | 0 | 0 | 0 | 0 | 0 | 0 | 0 | 1 |
| TREM2 | 1 | 0 | 0 | 0 | 0 | 0 | 0 | 0 | 1 |
| C21orf59 | 1 | 0 | 0 | 0 | 0 | 0 | 0 | 0 | 1 |
| F2R | 1 | 0 | 0 | 0 | 0 | 0 | 0 | 0 | 1 |
| ZDHHC14 | 1 | 0 | 0 | 0 | 0 | 0 | 0 | 0 | 1 |
| C2orf53 | 1 | 0 | 0 | 0 | 0 | 0 | 0 | 0 | 1 |
| PIGQ | 0 | 0 | 1 | 0 | 0 | 0 | 0 | 0 | 1 |
| DUSP9 | 1 | 0 | 0 | 0 | 0 | 0 | 0 | 0 | 1 |
| IFIT3 | 1 | 0 | 0 | 0 | 0 | 0 | 0 | 0 | 1 |
| EVI2B | 1 | 0 | 0 | 0 | 0 | 0 | 0 | 0 | 1 |
| ZNF500 | 1 | 0 | 0 | 0 | 0 | 0 | 0 | 0 | 1 |
| RAX2 | 1 | 0 | 0 | 0 | 0 | 0 | 0 | 0 | 1 |
| GIMAP6 | 1 | 0 | 0 | 0 | 0 | 0 | 0 | 0 | 1 |
| CDC20 | 1 | 0 | 0 | 0 | 0 | 0 | 0 | 0 | 1 |
| RTN4RL1 | 1 | 0 | 0 | 0 | 0 | 0 | 0 | 0 | 1 |
| GNB1L | 1 | 0 | 0 | 0 | 0 | 0 | 0 | 0 | 1 |
| KDELC1 | 1 | 0 | 0 | 0 | 0 | 0 | 0 | 0 | 1 |
| ZNF81 | 1 | 0 | 0 | 0 | 0 | 0 | 0 | 0 | 1 |
| FKBP5 | 1 | 0 | 0 | 0 | 0 | 0 | 0 | 0 | 1 |
| ILF2 | 1 | 0 | 0 | 0 | 0 | 0 | 0 | 0 | 1 |
| MYL4 | 1 | 0 | 0 | 0 | 0 | 0 | 0 | 0 | 1 |
| AP1S3 | 1 | 0 | 0 | 0 | 0 | 0 | 0 | 0 | 1 |
| SQSTM1 | 1 | 0 | 0 | 0 | 0 | 0 | 0 | 0 | 1 |
| COX15 | 1 | 0 | 0 | 0 | 0 | 0 | 0 | 0 | 1 |
| RESP18 | 1 | 0 | 0 | 0 | 0 | 0 | 0 | 0 | 1 |
| CCDC97 | 1 | 0 | 0 | 0 | 0 | 0 | 0 | 0 | 1 |
| AQP6 | 1 | 0 | 0 | 0 | 0 | 0 | 0 | 0 | 1 |
| LOXL2 | 0 | 1 | 0 | 0 | 0 | 0 | 0 | 0 | 1 |
| CHRM5 | 1 | 0 | 0 | 0 | 0 | 0 | 0 | 0 | 1 |
| SIRT7 | 1 | 0 | 0 | 0 | 0 | 0 | 0 | 0 | 1 |
| RAB37 | 1 | 0 | 0 | 0 | 0 | 0 | 0 | 0 | 1 |
| CEACAM19 | 1 | 0 | 0 | 0 | 0 | 0 | 0 | 0 | 1 |
| SLC30A7 | 1 | 0 | 0 | 0 | 0 | 0 | 0 | 0 | 1 |
| ATAD3C | 1 | 0 | 0 | 0 | 0 | 0 | 0 | 0 | 1 |
| NUS1 | 1 | 0 | 0 | 0 | 0 | 0 | 0 | 0 | 1 |
| HSD17B13 | 1 | 0 | 0 | 0 | 0 | 0 | 0 | 0 | 1 |
| RPL7 | 1 | 0 | 0 | 0 | 0 | 0 | 0 | 0 | 1 |
| USP50 | 1 | 0 | 0 | 0 | 0 | 0 | 0 | 0 | 1 |
| KRTAP1-3 | 1 | 0 | 0 | 0 | 0 | 0 | 0 | 0 | 1 |
| FLOT2 | 1 | 0 | 0 | 0 | 0 | 0 | 0 | 0 | 1 |
| WNT1 | 1 | 0 | 0 | 0 | 0 | 0 | 0 | 0 | 1 |

| | | | | | | | | | |
|---|---|---|---|---|---|---|---|---|---|
| IDH3G | 1 | 0 | 0 | 0 | 0 | 0 | 0 | 0 | 1 |
| FOLR4 | 1 | 0 | 0 | 0 | 0 | 0 | 0 | 0 | 1 |
| C9orf173 | 1 | 0 | 0 | 0 | 0 | 0 | 0 | 0 | 1 |
| TCEA2 | 1 | 0 | 0 | 0 | 0 | 0 | 0 | 0 | 1 |
| NKD2 | 1 | 0 | 0 | 0 | 0 | 0 | 0 | 0 | 1 |
| NKAIN3 | 1 | 0 | 0 | 0 | 0 | 0 | 0 | 0 | 1 |
| XPNPEP3 | 1 | 0 | 0 | 0 | 0 | 0 | 0 | 0 | 1 |
| TBP | 1 | 0 | 0 | 0 | 0 | 0 | 0 | 0 | 1 |
| CEPT1 | 1 | 0 | 0 | 0 | 0 | 0 | 0 | 0 | 1 |
| RAB40C | 1 | 0 | 0 | 0 | 0 | 0 | 0 | 0 | 1 |
| GPR31 | 1 | 0 | 0 | 0 | 0 | 0 | 0 | 0 | 1 |
| NARS | 1 | 0 | 0 | 0 | 0 | 0 | 0 | 0 | 1 |
| RAPGEF1 | 0 | 1 | 0 | 0 | 0 | 0 | 0 | 0 | 1 |
| C11orf48 | 1 | 0 | 0 | 0 | 0 | 0 | 0 | 0 | 1 |
| C17orf51 | 1 | 0 | 0 | 0 | 0 | 0 | 0 | 0 | 1 |
| TROAP | 0 | 1 | 0 | 0 | 0 | 0 | 0 | 0 | 1 |
| DNTTIP2 | 1 | 0 | 0 | 0 | 0 | 0 | 0 | 0 | 1 |
| NAMPT | 1 | 0 | 0 | 0 | 0 | 0 | 0 | 0 | 1 |
| SUDS3 | 1 | 0 | 0 | 0 | 0 | 0 | 0 | 0 | 1 |
| RIT1 | 1 | 0 | 0 | 0 | 0 | 0 | 0 | 0 | 1 |
| ZSCAN23 | 1 | 0 | 0 | 0 | 0 | 0 | 0 | 0 | 1 |
| LCAT | 1 | 0 | 0 | 0 | 0 | 0 | 0 | 0 | 1 |
| MYOZ1 | 1 | 0 | 0 | 0 | 0 | 0 | 0 | 0 | 1 |
| SLC39A1 | 1 | 0 | 0 | 0 | 0 | 0 | 0 | 0 | 1 |
| NFKBIB | 1 | 0 | 0 | 0 | 0 | 0 | 0 | 0 | 1 |
| TMEM47 | 1 | 0 | 0 | 0 | 0 | 0 | 0 | 0 | 1 |
| OTUD6B | 1 | 0 | 0 | 0 | 0 | 0 | 0 | 0 | 1 |
| OR2T10 | 1 | 0 | 0 | 0 | 0 | 0 | 0 | 0 | 1 |
| OPN1SW | 1 | 0 | 0 | 0 | 0 | 0 | 0 | 0 | 1 |
| ZNF385A | 1 | 0 | 0 | 0 | 0 | 0 | 0 | 0 | 1 |
| CNOT6 | 1 | 0 | 0 | 0 | 0 | 0 | 0 | 0 | 1 |
| DAPK2 | 1 | 0 | 0 | 0 | 0 | 0 | 0 | 0 | 1 |
| HIAT1 | 1 | 0 | 0 | 0 | 0 | 0 | 0 | 0 | 1 |
| GABRD | 1 | 0 | 0 | 0 | 0 | 0 | 0 | 0 | 1 |
| KRT20 | 1 | 0 | 0 | 0 | 0 | 0 | 0 | 0 | 1 |
| PGBD4 | 1 | 0 | 0 | 0 | 0 | 0 | 0 | 0 | 1 |
| HMGN5 | 1 | 0 | 0 | 0 | 0 | 0 | 0 | 0 | 1 |
| PUF60 | 1 | 0 | 0 | 0 | 0 | 0 | 0 | 0 | 1 |
| POLL | 1 | 0 | 0 | 0 | 0 | 0 | 0 | 0 | 1 |
| FBXO24 | 1 | 0 | 0 | 0 | 0 | 0 | 0 | 0 | 1 |
| KDSR | 1 | 0 | 0 | 0 | 0 | 0 | 0 | 0 | 1 |
| TMEM119 | 1 | 0 | 0 | 0 | 0 | 0 | 0 | 0 | 1 |
| ZP1 | 1 | 0 | 0 | 0 | 0 | 0 | 0 | 0 | 1 |
| WNT9B | 1 | 0 | 0 | 0 | 0 | 0 | 0 | 0 | 1 |
| SLC25A32 | 1 | 0 | 0 | 0 | 0 | 0 | 0 | 0 | 1 |
| GATA5 | 1 | 0 | 0 | 0 | 0 | 0 | 0 | 0 | 1 |

| | | | | | | | | | |
|---|---|---|---|---|---|---|---|---|---|
| C3orf52 | 1 | 0 | 0 | 0 | 0 | 0 | 0 | 0 | 1 |
| CXCR4 | 1 | 0 | 0 | 0 | 0 | 0 | 0 | 0 | 1 |
| RSAD2 | 1 | 0 | 0 | 0 | 0 | 0 | 0 | 0 | 1 |
| TPH1 | 1 | 0 | 0 | 0 | 0 | 0 | 0 | 0 | 1 |
| OPN4 | 1 | 0 | 0 | 0 | 0 | 0 | 0 | 0 | 1 |
| SERPINB10 | 1 | 0 | 0 | 0 | 0 | 0 | 0 | 0 | 1 |
| TSPAN32 | 1 | 0 | 0 | 0 | 0 | 0 | 0 | 0 | 1 |
| HS3ST5 | 1 | 0 | 0 | 0 | 0 | 0 | 0 | 0 | 1 |
| ST3GAL4 | 1 | 0 | 0 | 0 | 0 | 0 | 0 | 0 | 1 |
| LARP6 | 1 | 0 | 0 | 0 | 0 | 0 | 0 | 0 | 1 |
| MAP2K5 | 1 | 0 | 0 | 0 | 0 | 0 | 0 | 0 | 1 |
| PIRT | 1 | 0 | 0 | 0 | 0 | 0 | 0 | 0 | 1 |
| CARS2 | 0 | 0 | 0 | 0 | 1 | 0 | 0 | 0 | 1 |
| CD79A | 1 | 0 | 0 | 0 | 0 | 0 | 0 | 0 | 1 |
| FAM110B | 1 | 0 | 0 | 0 | 0 | 0 | 0 | 0 | 1 |
| CST9L | 1 | 0 | 0 | 0 | 0 | 0 | 0 | 0 | 1 |
| CARS | 0 | 0 | 1 | 0 | 0 | 0 | 0 | 0 | 1 |
| EED | 1 | 0 | 0 | 0 | 0 | 0 | 0 | 0 | 1 |
| PON3 | 1 | 0 | 0 | 0 | 0 | 0 | 0 | 0 | 1 |
| SNX8 | 1 | 0 | 0 | 0 | 0 | 0 | 0 | 0 | 1 |
| C14orf28 | 1 | 0 | 0 | 0 | 0 | 0 | 0 | 0 | 1 |
| LYAR | 1 | 0 | 0 | 0 | 0 | 0 | 0 | 0 | 1 |
| IFLTD1 | 1 | 0 | 0 | 0 | 0 | 0 | 0 | 0 | 1 |
| MRPL16 | 1 | 0 | 0 | 0 | 0 | 0 | 0 | 0 | 1 |
| HSPA14 | 1 | 0 | 0 | 0 | 0 | 0 | 0 | 0 | 1 |
| CDK7 | 1 | 0 | 0 | 0 | 0 | 0 | 0 | 0 | 1 |
| VPS52 | 1 | 0 | 0 | 0 | 0 | 0 | 0 | 0 | 1 |
| CAMK2A | 1 | 0 | 0 | 0 | 0 | 0 | 0 | 0 | 1 |
| ART1 | 1 | 0 | 0 | 0 | 0 | 0 | 0 | 0 | 1 |
| C11orf74 | 1 | 0 | 0 | 0 | 0 | 0 | 0 | 0 | 1 |
| CDYL | 1 | 0 | 0 | 0 | 0 | 0 | 0 | 0 | 1 |
| GLRA4 | 1 | 0 | 0 | 0 | 0 | 0 | 0 | 0 | 1 |
| RPF1 | 1 | 0 | 0 | 0 | 0 | 0 | 0 | 0 | 1 |
| PPP2R3C | 1 | 0 | 0 | 0 | 0 | 0 | 0 | 0 | 1 |
| DDI2 | 1 | 0 | 0 | 0 | 0 | 0 | 0 | 0 | 1 |
| CA11 | 1 | 0 | 0 | 0 | 0 | 0 | 0 | 0 | 1 |
| C20orf4 | 1 | 0 | 0 | 0 | 0 | 0 | 0 | 0 | 1 |
| KIF2A | 1 | 0 | 0 | 0 | 0 | 0 | 0 | 0 | 1 |
| SLC25A3 | 1 | 0 | 0 | 0 | 0 | 0 | 0 | 0 | 1 |
| CTSL1 | 1 | 0 | 0 | 0 | 0 | 0 | 0 | 0 | 1 |
| PSMD4 | 1 | 0 | 0 | 0 | 0 | 0 | 0 | 0 | 1 |
| SPZ1 | 1 | 0 | 0 | 0 | 0 | 0 | 0 | 0 | 1 |
| CD226 | 1 | 0 | 0 | 0 | 0 | 0 | 0 | 0 | 1 |
| APOBEC4 | 1 | 0 | 0 | 0 | 0 | 0 | 0 | 0 | 1 |
| GSTM4 | 1 | 0 | 0 | 0 | 0 | 0 | 0 | 0 | 1 |
| SYCE1L | 1 | 0 | 0 | 0 | 0 | 0 | 0 | 0 | 1 |

| | | | | | | | | | |
|---|---|---|---|---|---|---|---|---|---|
| IP6K3 | 1 | 0 | 0 | 0 | 0 | 0 | 0 | 0 | 1 |
| PRSS23 | 1 | 0 | 0 | 0 | 0 | 0 | 0 | 0 | 1 |
| KRTAP11-1 | 1 | 0 | 0 | 0 | 0 | 0 | 0 | 0 | 1 |
| TNFRSF10A | 1 | 0 | 0 | 0 | 0 | 0 | 0 | 0 | 1 |
| GPR84 | 1 | 0 | 0 | 0 | 0 | 0 | 0 | 0 | 1 |
| ALDH3B1 | 1 | 0 | 0 | 0 | 0 | 0 | 0 | 0 | 1 |
| ACTL6A | 1 | 0 | 0 | 0 | 0 | 0 | 0 | 0 | 1 |
| TMEM184C | 1 | 0 | 0 | 0 | 0 | 0 | 0 | 0 | 1 |
| CCDC126 | 1 | 0 | 0 | 0 | 0 | 0 | 0 | 0 | 1 |
| PLEKHA8 | 1 | 0 | 0 | 0 | 0 | 0 | 0 | 0 | 1 |
| NR0B2 | 1 | 0 | 0 | 0 | 0 | 0 | 0 | 0 | 1 |
| C10orf46 | 1 | 0 | 0 | 0 | 0 | 0 | 0 | 0 | 1 |
| OPRD1 | 1 | 0 | 0 | 0 | 0 | 0 | 0 | 0 | 1 |
| PRPF18 | 1 | 0 | 0 | 0 | 0 | 0 | 0 | 0 | 1 |
| G6PD | 1 | 0 | 0 | 0 | 0 | 0 | 0 | 0 | 1 |
| TOX2 | 1 | 0 | 0 | 0 | 0 | 0 | 0 | 0 | 1 |
| TMEM30A | 1 | 0 | 0 | 0 | 0 | 0 | 0 | 0 | 1 |
| NFKBIE | 1 | 0 | 0 | 0 | 0 | 0 | 0 | 0 | 1 |
| MREG | 1 | 0 | 0 | 0 | 0 | 0 | 0 | 0 | 1 |
| UTP6 | 1 | 0 | 0 | 0 | 0 | 0 | 0 | 0 | 1 |
| ETFB | 1 | 0 | 0 | 0 | 0 | 0 | 0 | 0 | 1 |
| SYNGAP1 | 0 | 1 | 0 | 0 | 0 | 0 | 0 | 0 | 1 |
| MAMLD1 | 0 | 1 | 0 | 0 | 0 | 0 | 0 | 0 | 1 |
| B3GALT2 | 1 | 0 | 0 | 0 | 0 | 0 | 0 | 0 | 1 |
| LANCL1 | 1 | 0 | 0 | 0 | 0 | 0 | 0 | 0 | 1 |
| C6orf106 | 1 | 0 | 0 | 0 | 0 | 0 | 0 | 0 | 1 |
| PCID2 | 1 | 0 | 0 | 0 | 0 | 0 | 0 | 0 | 1 |
| WNT8B | 1 | 0 | 0 | 0 | 0 | 0 | 0 | 0 | 1 |
| KIAA0494 | 1 | 0 | 0 | 0 | 0 | 0 | 0 | 0 | 1 |
| FAM122A | 1 | 0 | 0 | 0 | 0 | 0 | 0 | 0 | 1 |
| TACR2 | 1 | 0 | 0 | 0 | 0 | 0 | 0 | 0 | 1 |
| FUT11 | 1 | 0 | 0 | 0 | 0 | 0 | 0 | 0 | 1 |
| MMP14 | 1 | 0 | 0 | 0 | 0 | 0 | 0 | 0 | 1 |
| DNAJB13 | 1 | 0 | 0 | 0 | 0 | 0 | 0 | 0 | 1 |
| OMA1 | 1 | 0 | 0 | 0 | 0 | 0 | 0 | 0 | 1 |
| MPG | 1 | 0 | 0 | 0 | 0 | 0 | 0 | 0 | 1 |
| MIPOL1 | 1 | 0 | 0 | 0 | 0 | 0 | 0 | 0 | 1 |
| VPS72 | 1 | 0 | 0 | 0 | 0 | 0 | 0 | 0 | 1 |
| P2RY1 | 1 | 0 | 0 | 0 | 0 | 0 | 0 | 0 | 1 |
| RG9MTD1 | 1 | 0 | 0 | 0 | 0 | 0 | 0 | 0 | 1 |
| PLIN1 | 1 | 0 | 0 | 0 | 0 | 0 | 0 | 0 | 1 |
| SLC36A2 | 1 | 0 | 0 | 0 | 0 | 0 | 0 | 0 | 1 |
| CRYBB3 | 1 | 0 | 0 | 0 | 0 | 0 | 0 | 0 | 1 |
| DCAF4L1 | 1 | 0 | 0 | 0 | 0 | 0 | 0 | 0 | 1 |
| OXGR1 | 1 | 0 | 0 | 0 | 0 | 0 | 0 | 0 | 1 |
| KCTD21 | 1 | 0 | 0 | 0 | 0 | 0 | 0 | 0 | 1 |

| | | | | | | | | | |
|---|---|---|---|---|---|---|---|---|---|
| SMAD5 | 1 | 0 | 0 | 0 | 0 | 0 | 0 | 0 | 1 |
| PSPH | 1 | 0 | 0 | 0 | 0 | 0 | 0 | 0 | 1 |
| PELI1 | 1 | 0 | 0 | 0 | 0 | 0 | 0 | 0 | 1 |
| MPPED1 | 1 | 0 | 0 | 0 | 0 | 0 | 0 | 0 | 1 |
| ADIPOR2 | 1 | 0 | 0 | 0 | 0 | 0 | 0 | 0 | 1 |
| NFE2 | 1 | 0 | 0 | 0 | 0 | 0 | 0 | 0 | 1 |
| ALG6 | 1 | 0 | 0 | 0 | 0 | 0 | 0 | 0 | 1 |
| GPKOW | 1 | 0 | 0 | 0 | 0 | 0 | 0 | 0 | 1 |
| DHRS2 | 1 | 0 | 0 | 0 | 0 | 0 | 0 | 0 | 1 |
| MFSD5 | 1 | 0 | 0 | 0 | 0 | 0 | 0 | 0 | 1 |
| TXNDC5 | 1 | 0 | 0 | 0 | 0 | 0 | 0 | 0 | 1 |
| KHK | 1 | 0 | 0 | 0 | 0 | 0 | 0 | 0 | 1 |
| TAS1R3 | 0 | 1 | 0 | 0 | 0 | 0 | 0 | 0 | 1 |
| PSMD8 | 1 | 0 | 0 | 0 | 0 | 0 | 0 | 0 | 1 |
| C12orf5 | 1 | 0 | 0 | 0 | 0 | 0 | 0 | 0 | 1 |
| NSFL1C | 1 | 0 | 0 | 0 | 0 | 0 | 0 | 0 | 1 |
| SEC11C | 1 | 0 | 0 | 0 | 0 | 0 | 0 | 0 | 1 |
| SLC22A17 | 1 | 0 | 0 | 0 | 0 | 0 | 0 | 0 | 1 |
| CXCR3 | 1 | 0 | 0 | 0 | 0 | 0 | 0 | 0 | 1 |
| DTX3 | 1 | 0 | 0 | 0 | 0 | 0 | 0 | 0 | 1 |
| C16orf73 | 1 | 0 | 0 | 0 | 0 | 0 | 0 | 0 | 1 |
| BCS1L | 1 | 0 | 0 | 0 | 0 | 0 | 0 | 0 | 1 |
| ZADH2 | 1 | 0 | 0 | 0 | 0 | 0 | 0 | 0 | 1 |
| SSBP2 | 1 | 0 | 0 | 0 | 0 | 0 | 0 | 0 | 1 |
| SIX1 | 1 | 0 | 0 | 0 | 0 | 0 | 0 | 0 | 1 |
| SLC35B1 | 1 | 0 | 0 | 0 | 0 | 0 | 0 | 0 | 1 |
| SPOCK2 | 1 | 0 | 0 | 0 | 0 | 0 | 0 | 0 | 1 |
| EIF4E1B | 1 | 0 | 0 | 0 | 0 | 0 | 0 | 0 | 1 |
| IZUMO1 | 1 | 0 | 0 | 0 | 0 | 0 | 0 | 0 | 1 |
| RGS20 | 1 | 0 | 0 | 0 | 0 | 0 | 0 | 0 | 1 |
| PGK1 | 1 | 0 | 0 | 0 | 0 | 0 | 0 | 0 | 1 |
| OLR1 | 1 | 0 | 0 | 0 | 0 | 0 | 0 | 0 | 1 |
| STK17A | 1 | 0 | 0 | 0 | 0 | 0 | 0 | 0 | 1 |
| LAMP1 | 1 | 0 | 0 | 0 | 0 | 0 | 0 | 0 | 1 |
| CTH | 1 | 0 | 0 | 0 | 0 | 0 | 0 | 0 | 1 |
| PSEN2 | 1 | 0 | 0 | 0 | 0 | 0 | 0 | 0 | 1 |
| CD48 | 1 | 0 | 0 | 0 | 0 | 0 | 0 | 0 | 1 |
| HYLS1 | 1 | 0 | 0 | 0 | 0 | 0 | 0 | 0 | 1 |
| TFEB | 1 | 0 | 0 | 0 | 0 | 0 | 0 | 0 | 1 |
| RSBN1 | 0 | 1 | 0 | 0 | 0 | 0 | 0 | 0 | 1 |
| TPST1 | 1 | 0 | 0 | 0 | 0 | 0 | 0 | 0 | 1 |
| DMRTB1 | 1 | 0 | 0 | 0 | 0 | 0 | 0 | 0 | 1 |
| STAU1 | 1 | 0 | 0 | 0 | 0 | 0 | 0 | 0 | 1 |
| DNAJB6 | 1 | 0 | 0 | 0 | 0 | 0 | 0 | 0 | 1 |
| SH2D2A | 1 | 0 | 0 | 0 | 0 | 0 | 0 | 0 | 1 |
| LUC7L3 | 1 | 0 | 0 | 0 | 0 | 0 | 0 | 0 | 1 |

| | | | | | | | | | |
|---|---|---|---|---|---|---|---|---|---|
| PARP11 | 1 | 0 | 0 | 0 | 0 | 0 | 0 | 0 | 1 |
| KLK5 | 1 | 0 | 0 | 0 | 0 | 0 | 0 | 0 | 1 |
| AP4M1 | 1 | 0 | 0 | 0 | 0 | 0 | 0 | 0 | 1 |
| KIAA1279 | 1 | 0 | 0 | 0 | 0 | 0 | 0 | 0 | 1 |
| RARS | 1 | 0 | 0 | 0 | 0 | 0 | 0 | 0 | 1 |
| ZNF774 | 1 | 0 | 0 | 0 | 0 | 0 | 0 | 0 | 1 |
| FOXN2 | 1 | 0 | 0 | 0 | 0 | 0 | 0 | 0 | 1 |
| CYTIP | 1 | 0 | 0 | 0 | 0 | 0 | 0 | 0 | 1 |
| ABHD12B | 1 | 0 | 0 | 0 | 0 | 0 | 0 | 0 | 1 |
| TSPYL6 | 1 | 0 | 0 | 0 | 0 | 0 | 0 | 0 | 1 |
| SUSD5 | 1 | 0 | 0 | 0 | 0 | 0 | 0 | 0 | 1 |
| SQLE | 1 | 0 | 0 | 0 | 0 | 0 | 0 | 0 | 1 |
| CETP | 1 | 0 | 0 | 0 | 0 | 0 | 0 | 0 | 1 |
| NADK | 1 | 0 | 0 | 0 | 0 | 0 | 0 | 0 | 1 |
| FUT2 | 1 | 0 | 0 | 0 | 0 | 0 | 0 | 0 | 1 |
| ST13 | 1 | 0 | 0 | 0 | 0 | 0 | 0 | 0 | 1 |
| VDR | 1 | 0 | 0 | 0 | 0 | 0 | 0 | 0 | 1 |
| LZIC | 1 | 0 | 0 | 0 | 0 | 0 | 0 | 0 | 1 |
| GPR17 | 1 | 0 | 0 | 0 | 0 | 0 | 0 | 0 | 1 |
| TPST2 | 1 | 0 | 0 | 0 | 0 | 0 | 0 | 0 | 1 |
| TMEM25 | 1 | 0 | 0 | 0 | 0 | 0 | 0 | 0 | 1 |
| TEAD3 | 1 | 0 | 0 | 0 | 0 | 0 | 0 | 0 | 1 |
| CDCP1 | 0 | 1 | 0 | 0 | 0 | 0 | 0 | 0 | 1 |
| FAM105B | 1 | 0 | 0 | 0 | 0 | 0 | 0 | 0 | 1 |
| C16orf46 | 1 | 0 | 0 | 0 | 0 | 0 | 0 | 0 | 1 |
| C1orf86 | 1 | 0 | 0 | 0 | 0 | 0 | 0 | 0 | 1 |
| FHL1 | 1 | 0 | 0 | 0 | 0 | 0 | 0 | 0 | 1 |
| PARVA | 1 | 0 | 0 | 0 | 0 | 0 | 0 | 0 | 1 |
| UBAC1 | 1 | 0 | 0 | 0 | 0 | 0 | 0 | 0 | 1 |
| FSD1 | 1 | 0 | 0 | 0 | 0 | 0 | 0 | 0 | 1 |
| C16orf13 | 1 | 0 | 0 | 0 | 0 | 0 | 0 | 0 | 1 |
| DLK1 | 1 | 0 | 0 | 0 | 0 | 0 | 0 | 0 | 1 |
| C3orf38 | 1 | 0 | 0 | 0 | 0 | 0 | 0 | 0 | 1 |
| MTHFD2 | 1 | 0 | 0 | 0 | 0 | 0 | 0 | 0 | 1 |
| COLEC11 | 1 | 0 | 0 | 0 | 0 | 0 | 0 | 0 | 1 |
| FAM113B | 1 | 0 | 0 | 0 | 0 | 0 | 0 | 0 | 1 |
| PALM | 1 | 0 | 0 | 0 | 0 | 0 | 0 | 0 | 1 |
| VAX1 | 1 | 0 | 0 | 0 | 0 | 0 | 0 | 0 | 1 |
| CRLS1 | 1 | 0 | 0 | 0 | 0 | 0 | 0 | 0 | 1 |
| MDH1 | 1 | 0 | 0 | 0 | 0 | 0 | 0 | 0 | 1 |
| SLC35F4 | 1 | 0 | 0 | 0 | 0 | 0 | 0 | 0 | 1 |
| PIGG | 0 | 0 | 0 | 0 | 0 | 1 | 0 | 0 | 1 |
| ACAD8 | 1 | 0 | 0 | 0 | 0 | 0 | 0 | 0 | 1 |
| C8orf84 | 1 | 0 | 0 | 0 | 0 | 0 | 0 | 0 | 1 |
| SNF8 | 1 | 0 | 0 | 0 | 0 | 0 | 0 | 0 | 1 |
| LUC7L | 1 | 0 | 0 | 0 | 0 | 0 | 0 | 0 | 1 |

| | | | | | | | | | |
|---|---|---|---|---|---|---|---|---|---|
| PIAS1 | 1 | 0 | 0 | 0 | 0 | 0 | 0 | 0 | 1 |
| GLYATL3 | 1 | 0 | 0 | 0 | 0 | 0 | 0 | 0 | 1 |
| ANKRD2 | 1 | 0 | 0 | 0 | 0 | 0 | 0 | 0 | 1 |
| SPEM1 | 1 | 0 | 0 | 0 | 0 | 0 | 0 | 0 | 1 |
| SGMS2 | 1 | 0 | 0 | 0 | 0 | 0 | 0 | 0 | 1 |
| CTSD | 1 | 0 | 0 | 0 | 0 | 0 | 0 | 0 | 1 |
| SHD | 1 | 0 | 0 | 0 | 0 | 0 | 0 | 0 | 1 |
| RHBDD1 | 1 | 0 | 0 | 0 | 0 | 0 | 0 | 0 | 1 |
| EPM2A | 1 | 0 | 0 | 0 | 0 | 0 | 0 | 0 | 1 |
| ZDHHC21 | 1 | 0 | 0 | 0 | 0 | 0 | 0 | 0 | 1 |
| ALX3 | 1 | 0 | 0 | 0 | 0 | 0 | 0 | 0 | 1 |
| KBTBD5 | 1 | 0 | 0 | 0 | 0 | 0 | 0 | 0 | 1 |
| DHRS9 | 1 | 0 | 0 | 0 | 0 | 0 | 0 | 0 | 1 |
| HSPA1L | 1 | 0 | 0 | 0 | 0 | 0 | 0 | 0 | 1 |
| TMCC3 | 1 | 0 | 0 | 0 | 0 | 0 | 0 | 0 | 1 |
| RCC1 | 1 | 0 | 0 | 0 | 0 | 0 | 0 | 0 | 1 |
| FBXL15 | 1 | 0 | 0 | 0 | 0 | 0 | 0 | 0 | 1 |
| ELMOD2 | 1 | 0 | 0 | 0 | 0 | 0 | 0 | 0 | 1 |
| CADM1 | 1 | 0 | 0 | 0 | 0 | 0 | 0 | 0 | 1 |
| EXOC3L2 | 1 | 0 | 0 | 0 | 0 | 0 | 0 | 0 | 1 |
| AHR | 1 | 0 | 0 | 0 | 0 | 0 | 0 | 0 | 1 |
| NPVF | 1 | 0 | 0 | 0 | 0 | 0 | 0 | 0 | 1 |
| GPR180 | 1 | 0 | 0 | 0 | 0 | 0 | 0 | 0 | 1 |
| OR5B2 | 1 | 0 | 0 | 0 | 0 | 0 | 0 | 0 | 1 |
| HELB | 0 | 1 | 0 | 0 | 0 | 0 | 0 | 0 | 1 |
| FLRT1 | 0 | 0 | 0 | 0 | 1 | 0 | 0 | 0 | 1 |
| MCM9 | 1 | 0 | 0 | 0 | 0 | 0 | 0 | 0 | 1 |
| C1QTNF4 | 1 | 0 | 0 | 0 | 0 | 0 | 0 | 0 | 1 |
| ART3 | 1 | 0 | 0 | 0 | 0 | 0 | 0 | 0 | 1 |
| ProSAPiP1 | 1 | 0 | 0 | 0 | 0 | 0 | 0 | 0 | 1 |
| MAP1LC3B2 | 1 | 0 | 0 | 0 | 0 | 0 | 0 | 0 | 1 |
| KPNA4 | 1 | 0 | 0 | 0 | 0 | 0 | 0 | 0 | 1 |
| MYBPHL | 1 | 0 | 0 | 0 | 0 | 0 | 0 | 0 | 1 |
| ARFGAP2 | 1 | 0 | 0 | 0 | 0 | 0 | 0 | 0 | 1 |
| WNT8A | 1 | 0 | 0 | 0 | 0 | 0 | 0 | 0 | 1 |
| MC3R | 1 | 0 | 0 | 0 | 0 | 0 | 0 | 0 | 1 |
| ABO | 1 | 0 | 0 | 0 | 0 | 0 | 0 | 0 | 1 |
| OR7G1 | 1 | 0 | 0 | 0 | 0 | 0 | 0 | 0 | 1 |
| KLK12 | 1 | 0 | 0 | 0 | 0 | 0 | 0 | 0 | 1 |
| ZBTB32 | 1 | 0 | 0 | 0 | 0 | 0 | 0 | 0 | 1 |
| FBXW12 | 1 | 0 | 0 | 0 | 0 | 0 | 0 | 0 | 1 |
| DMRT3 | 1 | 0 | 0 | 0 | 0 | 0 | 0 | 0 | 1 |
| C2orf56 | 1 | 0 | 0 | 0 | 0 | 0 | 0 | 0 | 1 |
| B4GALNT3 | 0 | 1 | 0 | 0 | 0 | 0 | 0 | 0 | 1 |
| CCDC6 | 1 | 0 | 0 | 0 | 0 | 0 | 0 | 0 | 1 |
| RNF32 | 1 | 0 | 0 | 0 | 0 | 0 | 0 | 0 | 1 |

| | | | | | | | | | |
|---|---|---|---|---|---|---|---|---|---|
| CASP3 | 1 | 0 | 0 | 0 | 0 | 0 | 0 | 0 | 1 |
| TMOD1 | 1 | 0 | 0 | 0 | 0 | 0 | 0 | 0 | 1 |
| P2RX4 | 1 | 0 | 0 | 0 | 0 | 0 | 0 | 0 | 1 |
| SH3GL3 | 1 | 0 | 0 | 0 | 0 | 0 | 0 | 0 | 1 |
| NRBP1 | 1 | 0 | 0 | 0 | 0 | 0 | 0 | 0 | 1 |
| ADRA1B | 1 | 0 | 0 | 0 | 0 | 0 | 0 | 0 | 1 |
| C11orf49 | 1 | 0 | 0 | 0 | 0 | 0 | 0 | 0 | 1 |
| AFF4 | 0 | 0 | 0 | 0 | 0 | 1 | 0 | 0 | 1 |
| STK19 | 1 | 0 | 0 | 0 | 0 | 0 | 0 | 0 | 1 |
| TMEM99 | 1 | 0 | 0 | 0 | 0 | 0 | 0 | 0 | 1 |
| EI24 | 1 | 0 | 0 | 0 | 0 | 0 | 0 | 0 | 1 |
| FUCA2 | 1 | 0 | 0 | 0 | 0 | 0 | 0 | 0 | 1 |
| TMEM114 | 1 | 0 | 0 | 0 | 0 | 0 | 0 | 0 | 1 |
| DDX51 | 0 | 1 | 0 | 0 | 0 | 0 | 0 | 0 | 1 |
| CDC42EP1 | 1 | 0 | 0 | 0 | 0 | 0 | 0 | 0 | 1 |
| USP49 | 1 | 0 | 0 | 0 | 0 | 0 | 0 | 0 | 1 |
| CDC16 | 1 | 0 | 0 | 0 | 0 | 0 | 0 | 0 | 1 |
| WDR74 | 1 | 0 | 0 | 0 | 0 | 0 | 0 | 0 | 1 |
| ATP6V1C2 | 1 | 0 | 0 | 0 | 0 | 0 | 0 | 0 | 1 |
| GTF2E2 | 1 | 0 | 0 | 0 | 0 | 0 | 0 | 0 | 1 |
| LINGO1 | 1 | 0 | 0 | 0 | 0 | 0 | 0 | 0 | 1 |
| PIM3 | 1 | 0 | 0 | 0 | 0 | 0 | 0 | 0 | 1 |
| SLC30A4 | 1 | 0 | 0 | 0 | 0 | 0 | 0 | 0 | 1 |
| GPD1 | 1 | 0 | 0 | 0 | 0 | 0 | 0 | 0 | 1 |
| HSP90AB1 | 1 | 0 | 0 | 0 | 0 | 0 | 0 | 0 | 1 |
| CHRNE | 1 | 0 | 0 | 0 | 0 | 0 | 0 | 0 | 1 |
| FLVCR1 | 1 | 0 | 0 | 0 | 0 | 0 | 0 | 0 | 1 |
| ABAT | 1 | 0 | 0 | 0 | 0 | 0 | 0 | 0 | 1 |
| CSNK2A2 | 1 | 0 | 0 | 0 | 0 | 0 | 0 | 0 | 1 |
| C19orf66 | 1 | 0 | 0 | 0 | 0 | 0 | 0 | 0 | 1 |
| TEX9 | 1 | 0 | 0 | 0 | 0 | 0 | 0 | 0 | 1 |
| SERTAD4 | 1 | 0 | 0 | 0 | 0 | 0 | 0 | 0 | 1 |
| ZNF284 | 0 | 1 | 0 | 0 | 0 | 0 | 0 | 0 | 1 |
| WWTR1 | 1 | 0 | 0 | 0 | 0 | 0 | 0 | 0 | 1 |
| KLK11 | 1 | 0 | 0 | 0 | 0 | 0 | 0 | 0 | 1 |
| MFSD2B | 1 | 0 | 0 | 0 | 0 | 0 | 0 | 0 | 1 |
| PLEKHB1 | 1 | 0 | 0 | 0 | 0 | 0 | 0 | 0 | 1 |
| FBXW11 | 1 | 0 | 0 | 0 | 0 | 0 | 0 | 0 | 1 |
| HARBI1 | 1 | 0 | 0 | 0 | 0 | 0 | 0 | 0 | 1 |
| UQCRC1 | 1 | 0 | 0 | 0 | 0 | 0 | 0 | 0 | 1 |
| BTBD19 | 1 | 0 | 0 | 0 | 0 | 0 | 0 | 0 | 1 |
| HTR2A | 1 | 0 | 0 | 0 | 0 | 0 | 0 | 0 | 1 |
| SLC22A3 | 1 | 0 | 0 | 0 | 0 | 0 | 0 | 0 | 1 |
| LRIT3 | 1 | 0 | 0 | 0 | 0 | 0 | 0 | 0 | 1 |
| PPA2 | 1 | 0 | 0 | 0 | 0 | 0 | 0 | 0 | 1 |
| SYNC | 1 | 0 | 0 | 0 | 0 | 0 | 0 | 0 | 1 |

| | | | | | | | | | |
|---|---|---|---|---|---|---|---|---|---|
| OR10H4 | 1 | 0 | 0 | 0 | 0 | 0 | 0 | 0 | 1 |
| ADORA2B | 1 | 0 | 0 | 0 | 0 | 0 | 0 | 0 | 1 |
| FCER2 | 1 | 0 | 0 | 0 | 0 | 0 | 0 | 0 | 1 |
| RANGRF | 1 | 0 | 0 | 0 | 0 | 0 | 0 | 0 | 1 |
| CYLD | 0 | 1 | 0 | 0 | 0 | 0 | 0 | 0 | 1 |
| LAP3 | 0 | 0 | 0 | 0 | 1 | 0 | 0 | 0 | 1 |
| TLX1 | 1 | 0 | 0 | 0 | 0 | 0 | 0 | 0 | 1 |
| GIPR | 1 | 0 | 0 | 0 | 0 | 0 | 0 | 0 | 1 |
| DCAF10 | 1 | 0 | 0 | 0 | 0 | 0 | 0 | 0 | 1 |
| MAP2K4 | 1 | 0 | 0 | 0 | 0 | 0 | 0 | 0 | 1 |
| MMP21 | 1 | 0 | 0 | 0 | 0 | 0 | 0 | 0 | 1 |
| QRFPR | 1 | 0 | 0 | 0 | 0 | 0 | 0 | 0 | 1 |
| FBXL16 | 1 | 0 | 0 | 0 | 0 | 0 | 0 | 0 | 1 |
| ENOX2 | 1 | 0 | 0 | 0 | 0 | 0 | 0 | 0 | 1 |
| ATG3 | 1 | 0 | 0 | 0 | 0 | 0 | 0 | 0 | 1 |
| RDH13 | 1 | 0 | 0 | 0 | 0 | 0 | 0 | 0 | 1 |
| SLC7A7 | 1 | 0 | 0 | 0 | 0 | 0 | 0 | 0 | 1 |
| KIAA1841 | 1 | 0 | 0 | 0 | 0 | 0 | 0 | 0 | 1 |
| CLP1 | 1 | 0 | 0 | 0 | 0 | 0 | 0 | 0 | 1 |
| OSTalpha | 1 | 0 | 0 | 0 | 0 | 0 | 0 | 0 | 1 |
| SIGLEC8 | 0 | 1 | 0 | 0 | 0 | 0 | 0 | 0 | 1 |
| FTCD | 1 | 0 | 0 | 0 | 0 | 0 | 0 | 0 | 1 |
| CD14 | 1 | 0 | 0 | 0 | 0 | 0 | 0 | 0 | 1 |
| SLC20A2 | 1 | 0 | 0 | 0 | 0 | 0 | 0 | 0 | 1 |
| INA | 1 | 0 | 0 | 0 | 0 | 0 | 0 | 0 | 1 |
| AGFG2 | 1 | 0 | 0 | 0 | 0 | 0 | 0 | 0 | 1 |
| DUSP5 | 1 | 0 | 0 | 0 | 0 | 0 | 0 | 0 | 1 |
| FBXO22 | 1 | 0 | 0 | 0 | 0 | 0 | 0 | 0 | 1 |
| POLA2 | 1 | 0 | 0 | 0 | 0 | 0 | 0 | 0 | 1 |
| PTGR2 | 1 | 0 | 0 | 0 | 0 | 0 | 0 | 0 | 1 |
| NSF | 1 | 0 | 0 | 0 | 0 | 0 | 0 | 0 | 1 |
| ZNF30 | 0 | 1 | 0 | 0 | 0 | 0 | 0 | 0 | 1 |
| OR51E2 | 1 | 0 | 0 | 0 | 0 | 0 | 0 | 0 | 1 |
| PTPN9 | 1 | 0 | 0 | 0 | 0 | 0 | 0 | 0 | 1 |
| KCNK9 | 1 | 0 | 0 | 0 | 0 | 0 | 0 | 0 | 1 |
| GFRAL | 1 | 0 | 0 | 0 | 0 | 0 | 0 | 0 | 1 |
| AGXT | 1 | 0 | 0 | 0 | 0 | 0 | 0 | 0 | 1 |
| HNRNPA2B1 | 1 | 0 | 0 | 0 | 0 | 0 | 0 | 0 | 1 |
| SOX2 | 1 | 0 | 0 | 0 | 0 | 0 | 0 | 0 | 1 |
| PDCD4 | 1 | 0 | 0 | 0 | 0 | 0 | 0 | 0 | 1 |
| GLRA1 | 1 | 0 | 0 | 0 | 0 | 0 | 0 | 0 | 1 |
| C8orf47 | 1 | 0 | 0 | 0 | 0 | 0 | 0 | 0 | 1 |
| GLUD2 | 1 | 0 | 0 | 0 | 0 | 0 | 0 | 0 | 1 |
| PON2 | 1 | 0 | 0 | 0 | 0 | 0 | 0 | 0 | 1 |
| PRKAR1B | 1 | 0 | 0 | 0 | 0 | 0 | 0 | 0 | 1 |
| RINL | 1 | 0 | 0 | 0 | 0 | 0 | 0 | 0 | 1 |

| | | | | | | | | | |
|---|---|---|---|---|---|---|---|---|---|
| STK11 | 1 | 0 | 0 | 0 | 0 | 0 | 0 | 0 | 1 |
| NAA11 | 1 | 0 | 0 | 0 | 0 | 0 | 0 | 0 | 1 |
| P2RX7 | 0 | 1 | 0 | 0 | 0 | 0 | 0 | 0 | 1 |
| BCAS4 | 1 | 0 | 0 | 0 | 0 | 0 | 0 | 0 | 1 |
| POLG2 | 1 | 0 | 0 | 0 | 0 | 0 | 0 | 0 | 1 |
| TAT | 1 | 0 | 0 | 0 | 0 | 0 | 0 | 0 | 1 |
| SLC46A2 | 1 | 0 | 0 | 0 | 0 | 0 | 0 | 0 | 1 |
| CD200R1 | 1 | 0 | 0 | 0 | 0 | 0 | 0 | 0 | 1 |
| OR10V1 | 1 | 0 | 0 | 0 | 0 | 0 | 0 | 0 | 1 |
| PLCXD2 | 1 | 0 | 0 | 0 | 0 | 0 | 0 | 0 | 1 |
| GPR101 | 1 | 0 | 0 | 0 | 0 | 0 | 0 | 0 | 1 |
| ZNF793 | 1 | 0 | 0 | 0 | 0 | 0 | 0 | 0 | 1 |
| EBNA1BP2 | 1 | 0 | 0 | 0 | 0 | 0 | 0 | 0 | 1 |
| PLS1 | 1 | 0 | 0 | 0 | 0 | 0 | 0 | 0 | 1 |
| SPINK7 | 0 | 1 | 0 | 0 | 0 | 0 | 0 | 0 | 1 |
| PTBP1 | 1 | 0 | 0 | 0 | 0 | 0 | 0 | 0 | 1 |
| SPIRE2 | 0 | 0 | 0 | 0 | 1 | 0 | 0 | 0 | 1 |
| SDC1 | 1 | 0 | 0 | 0 | 0 | 0 | 0 | 0 | 1 |
| MPPED2 | 1 | 0 | 0 | 0 | 0 | 0 | 0 | 0 | 1 |
| KLF3 | 1 | 0 | 0 | 0 | 0 | 0 | 0 | 0 | 1 |
| FBP2 | 1 | 0 | 0 | 0 | 0 | 0 | 0 | 0 | 1 |
| TGFA | 1 | 0 | 0 | 0 | 0 | 0 | 0 | 0 | 1 |
| SLC10A2 | 1 | 0 | 0 | 0 | 0 | 0 | 0 | 0 | 1 |
| MEOX1 | 1 | 0 | 0 | 0 | 0 | 0 | 0 | 0 | 1 |
| GDF5 | 1 | 0 | 0 | 0 | 0 | 0 | 0 | 0 | 1 |
| CRK | 1 | 0 | 0 | 0 | 0 | 0 | 0 | 0 | 1 |
| TFCP2L1 | 1 | 0 | 0 | 0 | 0 | 0 | 0 | 0 | 1 |
| FAM49A | 1 | 0 | 0 | 0 | 0 | 0 | 0 | 0 | 1 |
| SERINC5 | 1 | 0 | 0 | 0 | 0 | 0 | 0 | 0 | 1 |
| ESX1 | 1 | 0 | 0 | 0 | 0 | 0 | 0 | 0 | 1 |
| TMEM66 | 1 | 0 | 0 | 0 | 0 | 0 | 0 | 0 | 1 |
| PPP2R2B | 1 | 0 | 0 | 0 | 0 | 0 | 0 | 0 | 1 |
| CDK16 | 1 | 0 | 0 | 0 | 0 | 0 | 0 | 0 | 1 |
| ZNF652 | 1 | 0 | 0 | 0 | 0 | 0 | 0 | 0 | 1 |
| MRPL39 | 1 | 0 | 0 | 0 | 0 | 0 | 0 | 0 | 1 |
| RUNX1 | 1 | 0 | 0 | 0 | 0 | 0 | 0 | 0 | 1 |
| OR10K2 | 1 | 0 | 0 | 0 | 0 | 0 | 0 | 0 | 1 |
| HABP4 | 1 | 0 | 0 | 0 | 0 | 0 | 0 | 0 | 1 |
| SLC38A4 | 1 | 0 | 0 | 0 | 0 | 0 | 0 | 0 | 1 |
| RIBC1 | 1 | 0 | 0 | 0 | 0 | 0 | 0 | 0 | 1 |
| NPY1R | 1 | 0 | 0 | 0 | 0 | 0 | 0 | 0 | 1 |
| NSUN6 | 1 | 0 | 0 | 0 | 0 | 0 | 0 | 0 | 1 |
| FADS3 | 1 | 0 | 0 | 0 | 0 | 0 | 0 | 0 | 1 |
| GRB2 | 1 | 0 | 0 | 0 | 0 | 0 | 0 | 0 | 1 |
| LMBR1L | 1 | 0 | 0 | 0 | 0 | 0 | 0 | 0 | 1 |
| DHPS | 1 | 0 | 0 | 0 | 0 | 0 | 0 | 0 | 1 |

| | | | | | | | | | |
|---|---|---|---|---|---|---|---|---|---|
| TGFBR1 | 1 | 0 | 0 | 0 | 0 | 0 | 0 | 0 | 1 |
| TRHR | 1 | 0 | 0 | 0 | 0 | 0 | 0 | 0 | 1 |
| ANGPTL2 | 1 | 0 | 0 | 0 | 0 | 0 | 0 | 0 | 1 |
| ZCCHC8 | 1 | 0 | 0 | 0 | 0 | 0 | 0 | 0 | 1 |
| CCDC64B | 1 | 0 | 0 | 0 | 0 | 0 | 0 | 0 | 1 |
| BECN1 | 1 | 0 | 0 | 0 | 0 | 0 | 0 | 0 | 1 |
| ASB5 | 1 | 0 | 0 | 0 | 0 | 0 | 0 | 0 | 1 |
| KLF15 | 1 | 0 | 0 | 0 | 0 | 0 | 0 | 0 | 1 |
| KCNJ16 | 1 | 0 | 0 | 0 | 0 | 0 | 0 | 0 | 1 |
| TERF1 | 1 | 0 | 0 | 0 | 0 | 0 | 0 | 0 | 1 |
| ERO1LB | 1 | 0 | 0 | 0 | 0 | 0 | 0 | 0 | 1 |
| PREB | 1 | 0 | 0 | 0 | 0 | 0 | 0 | 0 | 1 |
| PRSS38 | 1 | 0 | 0 | 0 | 0 | 0 | 0 | 0 | 1 |
| NMBR | 1 | 0 | 0 | 0 | 0 | 0 | 0 | 0 | 1 |
| OR2A12 | 1 | 0 | 0 | 0 | 0 | 0 | 0 | 0 | 1 |
| MAPK6 | 1 | 0 | 0 | 0 | 0 | 0 | 0 | 0 | 1 |
| ZNF556 | 1 | 0 | 0 | 0 | 0 | 0 | 0 | 0 | 1 |
| KYNU | 1 | 0 | 0 | 0 | 0 | 0 | 0 | 0 | 1 |
| ANXA10 | 1 | 0 | 0 | 0 | 0 | 0 | 0 | 0 | 1 |
| TGFB3 | 1 | 0 | 0 | 0 | 0 | 0 | 0 | 0 | 1 |
| FBXL12 | 1 | 0 | 0 | 0 | 0 | 0 | 0 | 0 | 1 |
| OR6C4 | 1 | 0 | 0 | 0 | 0 | 0 | 0 | 0 | 1 |
| AKT1 | 1 | 0 | 0 | 0 | 0 | 0 | 0 | 0 | 1 |
| C5orf51 | 1 | 0 | 0 | 0 | 0 | 0 | 0 | 0 | 1 |
| C16orf59 | 1 | 0 | 0 | 0 | 0 | 0 | 0 | 0 | 1 |
| EPB49 | 1 | 0 | 0 | 0 | 0 | 0 | 0 | 0 | 1 |
| ACOX1 | 1 | 0 | 0 | 0 | 0 | 0 | 0 | 0 | 1 |
| PLAUR | 1 | 0 | 0 | 0 | 0 | 0 | 0 | 0 | 1 |
| DSCC1 | 1 | 0 | 0 | 0 | 0 | 0 | 0 | 0 | 1 |
| ABI1 | 1 | 0 | 0 | 0 | 0 | 0 | 0 | 0 | 1 |
| RUNX3 | 1 | 0 | 0 | 0 | 0 | 0 | 0 | 0 | 1 |
| OR52N1 | 1 | 0 | 0 | 0 | 0 | 0 | 0 | 0 | 1 |
| CLEC4M | 1 | 0 | 0 | 0 | 0 | 0 | 0 | 0 | 1 |
| DDX49 | 1 | 0 | 0 | 0 | 0 | 0 | 0 | 0 | 1 |
| ASH2L | 1 | 0 | 0 | 0 | 0 | 0 | 0 | 0 | 1 |
| B3GALNT2 | 1 | 0 | 0 | 0 | 0 | 0 | 0 | 0 | 1 |
| EPHX2 | 1 | 0 | 0 | 0 | 0 | 0 | 0 | 0 | 1 |
| SLC22A18 | 1 | 0 | 0 | 0 | 0 | 0 | 0 | 0 | 1 |
| TIGD4 | 1 | 0 | 0 | 0 | 0 | 0 | 0 | 0 | 1 |
| SLC22A5 | 1 | 0 | 0 | 0 | 0 | 0 | 0 | 0 | 1 |
| GATM | 1 | 0 | 0 | 0 | 0 | 0 | 0 | 0 | 1 |
| RSPO3 | 1 | 0 | 0 | 0 | 0 | 0 | 0 | 0 | 1 |
| ZDHHC3 | 1 | 0 | 0 | 0 | 0 | 0 | 0 | 0 | 1 |
| OLFML3 | 1 | 0 | 0 | 0 | 0 | 0 | 0 | 0 | 1 |
| GATSL3 | 1 | 0 | 0 | 0 | 0 | 0 | 0 | 0 | 1 |
| IER5L | 1 | 0 | 0 | 0 | 0 | 0 | 0 | 0 | 1 |

| | | | | | | | | | |
|---|---|---|---|---|---|---|---|---|---|
| GPRC5B | 1 | 0 | 0 | 0 | 0 | 0 | 0 | 0 | 1 |
| ITPRIP | 1 | 0 | 0 | 0 | 0 | 0 | 0 | 0 | 1 |
| POMT1 | 1 | 0 | 0 | 0 | 0 | 0 | 0 | 0 | 1 |
| CSNK1E | 1 | 0 | 0 | 0 | 0 | 0 | 0 | 0 | 1 |
| ASPG | 1 | 0 | 0 | 0 | 0 | 0 | 0 | 0 | 1 |
| PDCD1 | 1 | 0 | 0 | 0 | 0 | 0 | 0 | 0 | 1 |
| CLRN3 | 1 | 0 | 0 | 0 | 0 | 0 | 0 | 0 | 1 |
| OXSM | 1 | 0 | 0 | 0 | 0 | 0 | 0 | 0 | 1 |
| ALOX12B | 1 | 0 | 0 | 0 | 0 | 0 | 0 | 0 | 1 |
| AKT2 | 1 | 0 | 0 | 0 | 0 | 0 | 0 | 0 | 1 |
| TMEM151A | 1 | 0 | 0 | 0 | 0 | 0 | 0 | 0 | 1 |
| CX3CL1 | 1 | 0 | 0 | 0 | 0 | 0 | 0 | 0 | 1 |
| ARID3C | 1 | 0 | 0 | 0 | 0 | 0 | 0 | 0 | 1 |
| GLB1L | 1 | 0 | 0 | 0 | 0 | 0 | 0 | 0 | 1 |
| RAE1 | 1 | 0 | 0 | 0 | 0 | 0 | 0 | 0 | 1 |
| TIGD3 | 1 | 0 | 0 | 0 | 0 | 0 | 0 | 0 | 1 |
| TRIB2 | 1 | 0 | 0 | 0 | 0 | 0 | 0 | 0 | 1 |
| OCLN | 1 | 0 | 0 | 0 | 0 | 0 | 0 | 0 | 1 |
| PTH1R | 1 | 0 | 0 | 0 | 0 | 0 | 0 | 0 | 1 |
| PRUNE | 1 | 0 | 0 | 0 | 0 | 0 | 0 | 0 | 1 |
| PABPC1 | 1 | 0 | 0 | 0 | 0 | 0 | 0 | 0 | 1 |
| TBC1D14 | 1 | 0 | 0 | 0 | 0 | 0 | 0 | 0 | 1 |
| OLAH | 1 | 0 | 0 | 0 | 0 | 0 | 0 | 0 | 1 |
| MRPS9 | 1 | 0 | 0 | 0 | 0 | 0 | 0 | 0 | 1 |
| HARS | 1 | 0 | 0 | 0 | 0 | 0 | 0 | 0 | 1 |
| PIGU | 1 | 0 | 0 | 0 | 0 | 0 | 0 | 0 | 1 |
| LAMP3 | 1 | 0 | 0 | 0 | 0 | 0 | 0 | 0 | 1 |
| FADS2 | 1 | 0 | 0 | 0 | 0 | 0 | 0 | 0 | 1 |
| NRSN1 | 1 | 0 | 0 | 0 | 0 | 0 | 0 | 0 | 1 |
| IDH3B | 1 | 0 | 0 | 0 | 0 | 0 | 0 | 0 | 1 |
| ATP8B1 | 0 | 1 | 0 | 0 | 0 | 0 | 0 | 0 | 1 |
| TOR3A | 1 | 0 | 0 | 0 | 0 | 0 | 0 | 0 | 1 |
| OLFM1 | 1 | 0 | 0 | 0 | 0 | 0 | 0 | 0 | 1 |
| SKP2 | 1 | 0 | 0 | 0 | 0 | 0 | 0 | 0 | 1 |
| TNNT1 | 1 | 0 | 0 | 0 | 0 | 0 | 0 | 0 | 1 |
| CNOT6L | 1 | 0 | 0 | 0 | 0 | 0 | 0 | 0 | 1 |
| SLC10A5 | 1 | 0 | 0 | 0 | 0 | 0 | 0 | 0 | 1 |
| TUBG2 | 1 | 0 | 0 | 0 | 0 | 0 | 0 | 0 | 1 |
| MPND | 1 | 0 | 0 | 0 | 0 | 0 | 0 | 0 | 1 |
| TTC23L | 1 | 0 | 0 | 0 | 0 | 0 | 0 | 0 | 1 |
| ACSF2 | 1 | 0 | 0 | 0 | 0 | 0 | 0 | 0 | 1 |
| GGT6 | 1 | 0 | 0 | 0 | 0 | 0 | 0 | 0 | 1 |
| CSGALNACT1 | 1 | 0 | 0 | 0 | 0 | 0 | 0 | 0 | 1 |
| ZNF280A | 1 | 0 | 0 | 0 | 0 | 0 | 0 | 0 | 1 |
| SLC15A3 | 1 | 0 | 0 | 0 | 0 | 0 | 0 | 0 | 1 |
| VPS37B | 1 | 0 | 0 | 0 | 0 | 0 | 0 | 0 | 1 |

| | | | | | | | | | |
|---|---|---|---|---|---|---|---|---|---|
| UBE2Q2 | 1 | 0 | 0 | 0 | 0 | 0 | 0 | 0 | 1 |
| ZNF76 | 1 | 0 | 0 | 0 | 0 | 0 | 0 | 0 | 1 |
| KCNA7 | 1 | 0 | 0 | 0 | 0 | 0 | 0 | 0 | 1 |
| SMPDL3B | 1 | 0 | 0 | 0 | 0 | 0 | 0 | 0 | 1 |
| IRF5 | 1 | 0 | 0 | 0 | 0 | 0 | 0 | 0 | 1 |
| EGLN3 | 1 | 0 | 0 | 0 | 0 | 0 | 0 | 0 | 1 |
| AKR1B15 | 1 | 0 | 0 | 0 | 0 | 0 | 0 | 0 | 1 |
| ANKRD42 | 1 | 0 | 0 | 0 | 0 | 0 | 0 | 0 | 1 |
| UBXN8 | 1 | 0 | 0 | 0 | 0 | 0 | 0 | 0 | 1 |
| DEK | 1 | 0 | 0 | 0 | 0 | 0 | 0 | 0 | 1 |
| EID3 | 1 | 0 | 0 | 0 | 0 | 0 | 0 | 0 | 1 |
| SMTNL2 | 1 | 0 | 0 | 0 | 0 | 0 | 0 | 0 | 1 |
| FFAR2 | 1 | 0 | 0 | 0 | 0 | 0 | 0 | 0 | 1 |
| UQCRC2 | 1 | 0 | 0 | 0 | 0 | 0 | 0 | 0 | 1 |
| JRKL | 1 | 0 | 0 | 0 | 0 | 0 | 0 | 0 | 1 |
| RIOK1 | 1 | 0 | 0 | 0 | 0 | 0 | 0 | 0 | 1 |
| MAGEB3 | 1 | 0 | 0 | 0 | 0 | 0 | 0 | 0 | 1 |
| RCC2 | 1 | 0 | 0 | 0 | 0 | 0 | 0 | 0 | 1 |
| ISLR | 1 | 0 | 0 | 0 | 0 | 0 | 0 | 0 | 1 |
| ZC3H15 | 1 | 0 | 0 | 0 | 0 | 0 | 0 | 0 | 1 |
| SRPX | 1 | 0 | 0 | 0 | 0 | 0 | 0 | 0 | 1 |
| MAGIX | 1 | 0 | 0 | 0 | 0 | 0 | 0 | 0 | 1 |
| PNCK | 1 | 0 | 0 | 0 | 0 | 0 | 0 | 0 | 1 |
| LRRC6 | 1 | 0 | 0 | 0 | 0 | 0 | 0 | 0 | 1 |
| APBB3 | 1 | 0 | 0 | 0 | 0 | 0 | 0 | 0 | 1 |
| XKRX | 1 | 0 | 0 | 0 | 0 | 0 | 0 | 0 | 1 |
| NFKBIZ | 1 | 0 | 0 | 0 | 0 | 0 | 0 | 0 | 1 |
| ZNF485 | 1 | 0 | 0 | 0 | 0 | 0 | 0 | 0 | 1 |
| B4GALT3 | 1 | 0 | 0 | 0 | 0 | 0 | 0 | 0 | 1 |
| TNFRSF11A | 1 | 0 | 0 | 0 | 0 | 0 | 0 | 0 | 1 |
| SLC38A7 | 1 | 0 | 0 | 0 | 0 | 0 | 0 | 0 | 1 |
| JAM2 | 1 | 0 | 0 | 0 | 0 | 0 | 0 | 0 | 1 |
| SERPINE3 | 1 | 0 | 0 | 0 | 0 | 0 | 0 | 0 | 1 |
| P2RX2 | 1 | 0 | 0 | 0 | 0 | 0 | 0 | 0 | 1 |
| CCNH | 1 | 0 | 0 | 0 | 0 | 0 | 0 | 0 | 1 |
| UPF3A | 1 | 0 | 0 | 0 | 0 | 0 | 0 | 0 | 1 |
| RNF168 | 1 | 0 | 0 | 0 | 0 | 0 | 0 | 0 | 1 |
| HYAL3 | 1 | 0 | 0 | 0 | 0 | 0 | 0 | 0 | 1 |
| SLC19A3 | 1 | 0 | 0 | 0 | 0 | 0 | 0 | 0 | 1 |
| TMEM44 | 1 | 0 | 0 | 0 | 0 | 0 | 0 | 0 | 1 |
| ZRANB2 | 1 | 0 | 0 | 0 | 0 | 0 | 0 | 0 | 1 |
| GLRX3 | 1 | 0 | 0 | 0 | 0 | 0 | 0 | 0 | 1 |
| GCDH | 1 | 0 | 0 | 0 | 0 | 0 | 0 | 0 | 1 |
| GAPT | 1 | 0 | 0 | 0 | 0 | 0 | 0 | 0 | 1 |
| PGM2L1 | 1 | 0 | 0 | 0 | 0 | 0 | 0 | 0 | 1 |
| PIAS4 | 1 | 0 | 0 | 0 | 0 | 0 | 0 | 0 | 1 |

| | | | | | | | | | |
|---|---|---|---|---|---|---|---|---|---|
| VASP | 1 | 0 | 0 | 0 | 0 | 0 | 0 | 0 | 1 |
| ME2 | 1 | 0 | 0 | 0 | 0 | 0 | 0 | 0 | 1 |
| DDX25 | 1 | 0 | 0 | 0 | 0 | 0 | 0 | 0 | 1 |
| TMCO5A | 1 | 0 | 0 | 0 | 0 | 0 | 0 | 0 | 1 |
| HDHD2 | 1 | 0 | 0 | 0 | 0 | 0 | 0 | 0 | 1 |
| GMEB1 | 1 | 0 | 0 | 0 | 0 | 0 | 0 | 0 | 1 |
| MED27 | 1 | 0 | 0 | 0 | 0 | 0 | 0 | 0 | 1 |
| PRMT5 | 1 | 0 | 0 | 0 | 0 | 0 | 0 | 0 | 1 |
| WNT3A | 1 | 0 | 0 | 0 | 0 | 0 | 0 | 0 | 1 |
| C11orf24 | 1 | 0 | 0 | 0 | 0 | 0 | 0 | 0 | 1 |
| MANEA | 1 | 0 | 0 | 0 | 0 | 0 | 0 | 0 | 1 |
| PPP3CB | 1 | 0 | 0 | 0 | 0 | 0 | 0 | 0 | 1 |
| RSRC2 | 1 | 0 | 0 | 0 | 0 | 0 | 0 | 0 | 1 |
| CATSPER3 | 1 | 0 | 0 | 0 | 0 | 0 | 0 | 0 | 1 |
| GFAP | 1 | 0 | 0 | 0 | 0 | 0 | 0 | 0 | 1 |
| SPAG6 | 1 | 0 | 0 | 0 | 0 | 0 | 0 | 0 | 1 |
| MTNR1B | 1 | 0 | 0 | 0 | 0 | 0 | 0 | 0 | 1 |
| SLC22A20 | 1 | 0 | 0 | 0 | 0 | 0 | 0 | 0 | 1 |
| ARRB1 | 1 | 0 | 0 | 0 | 0 | 0 | 0 | 0 | 1 |
| AGAP1 | 0 | 1 | 0 | 0 | 0 | 0 | 0 | 0 | 1 |
| LPPR2 | 1 | 0 | 0 | 0 | 0 | 0 | 0 | 0 | 1 |
| HOXB8 | 1 | 0 | 0 | 0 | 0 | 0 | 0 | 0 | 1 |
| RCOR1 | 1 | 0 | 0 | 0 | 0 | 0 | 0 | 0 | 1 |
| PLAU | 1 | 0 | 0 | 0 | 0 | 0 | 0 | 0 | 1 |
| EIF3J | 1 | 0 | 0 | 0 | 0 | 0 | 0 | 0 | 1 |
| HRH1 | 1 | 0 | 0 | 0 | 0 | 0 | 0 | 0 | 1 |
| SULT1C2 | 1 | 0 | 0 | 0 | 0 | 0 | 0 | 0 | 1 |
| SLU7 | 1 | 0 | 0 | 0 | 0 | 0 | 0 | 0 | 1 |
| CSNK1D | 1 | 0 | 0 | 0 | 0 | 0 | 0 | 0 | 1 |
| RGS18 | 1 | 0 | 0 | 0 | 0 | 0 | 0 | 0 | 1 |
| ENO1 | 1 | 0 | 0 | 0 | 0 | 0 | 0 | 0 | 1 |
| OR6T1 | 1 | 0 | 0 | 0 | 0 | 0 | 0 | 0 | 1 |
| CHRM4 | 1 | 0 | 0 | 0 | 0 | 0 | 0 | 0 | 1 |
| PM20D1 | 1 | 0 | 0 | 0 | 0 | 0 | 0 | 0 | 1 |
| LPCAT4 | 1 | 0 | 0 | 0 | 0 | 0 | 0 | 0 | 1 |
| SH3BP2 | 1 | 0 | 0 | 0 | 0 | 0 | 0 | 0 | 1 |
| WDR37 | 1 | 0 | 0 | 0 | 0 | 0 | 0 | 0 | 1 |
| SULT1B1 | 1 | 0 | 0 | 0 | 0 | 0 | 0 | 0 | 1 |
| CYTH2 | 1 | 0 | 0 | 0 | 0 | 0 | 0 | 0 | 1 |
| KEAP1 | 1 | 0 | 0 | 0 | 0 | 0 | 0 | 0 | 1 |
| NPTX1 | 1 | 0 | 0 | 0 | 0 | 0 | 0 | 0 | 1 |
| TNFRSF6B | 1 | 0 | 0 | 0 | 0 | 0 | 0 | 0 | 1 |
| EPYC | 1 | 0 | 0 | 0 | 0 | 0 | 0 | 0 | 1 |
| IMPDH2 | 1 | 0 | 0 | 0 | 0 | 0 | 0 | 0 | 1 |
| CALHM3 | 1 | 0 | 0 | 0 | 0 | 0 | 0 | 0 | 1 |
| WNT6 | 1 | 0 | 0 | 0 | 0 | 0 | 0 | 0 | 1 |

| | | | | | | | | | |
|---|---|---|---|---|---|---|---|---|---|
| DNAJC22 | 1 | 0 | 0 | 0 | 0 | 0 | 0 | 0 | 1 |
| USP22 | 1 | 0 | 0 | 0 | 0 | 0 | 0 | 0 | 1 |
| ATP6V1C1 | 1 | 0 | 0 | 0 | 0 | 0 | 0 | 0 | 1 |
| CPXCR1 | 1 | 0 | 0 | 0 | 0 | 0 | 0 | 0 | 1 |
| PSMD5 | 1 | 0 | 0 | 0 | 0 | 0 | 0 | 0 | 1 |
| IFNGR1 | 1 | 0 | 0 | 0 | 0 | 0 | 0 | 0 | 1 |
| ADK | 1 | 0 | 0 | 0 | 0 | 0 | 0 | 0 | 1 |
| TTC7B | 1 | 0 | 0 | 0 | 0 | 0 | 0 | 0 | 1 |
| PLEKHO1 | 1 | 0 | 0 | 0 | 0 | 0 | 0 | 0 | 1 |
| PRMT7 | 1 | 0 | 0 | 0 | 0 | 0 | 0 | 0 | 1 |
| PPP2R2D | 1 | 0 | 0 | 0 | 0 | 0 | 0 | 0 | 1 |
| CDC25C | 1 | 0 | 0 | 0 | 0 | 0 | 0 | 0 | 1 |
| FDFT1 | 1 | 0 | 0 | 0 | 0 | 0 | 0 | 0 | 1 |
| B3GALTL | 1 | 0 | 0 | 0 | 0 | 0 | 0 | 0 | 1 |
| NT5E | 1 | 0 | 0 | 0 | 0 | 0 | 0 | 0 | 1 |
| CCT5 | 1 | 0 | 0 | 0 | 0 | 0 | 0 | 0 | 1 |
| QSOX2 | 1 | 0 | 0 | 0 | 0 | 0 | 0 | 0 | 1 |
| TEF | 1 | 0 | 0 | 0 | 0 | 0 | 0 | 0 | 1 |
| CTSO | 1 | 0 | 0 | 0 | 0 | 0 | 0 | 0 | 1 |
| GHSR | 1 | 0 | 0 | 0 | 0 | 0 | 0 | 0 | 1 |
| ALDH3B2 | 1 | 0 | 0 | 0 | 0 | 0 | 0 | 0 | 1 |
| HIBCH | 1 | 0 | 0 | 0 | 0 | 0 | 0 | 0 | 1 |
| ANGPTL7 | 1 | 0 | 0 | 0 | 0 | 0 | 0 | 0 | 1 |
| GPR143 | 1 | 0 | 0 | 0 | 0 | 0 | 0 | 0 | 1 |
| CNTF | 1 | 0 | 0 | 0 | 0 | 0 | 0 | 0 | 1 |
| STK40 | 1 | 0 | 0 | 0 | 0 | 0 | 0 | 0 | 1 |
| CAMKK2 | 1 | 0 | 0 | 0 | 0 | 0 | 0 | 0 | 1 |
| CDC40 | 1 | 0 | 0 | 0 | 0 | 0 | 0 | 0 | 1 |
| EME1 | 1 | 0 | 0 | 0 | 0 | 0 | 0 | 0 | 1 |
| PHYHIPL | 1 | 0 | 0 | 0 | 0 | 0 | 0 | 0 | 1 |
| CYP11A1 | 1 | 0 | 0 | 0 | 0 | 0 | 0 | 0 | 1 |
| OR51A7 | 1 | 0 | 0 | 0 | 0 | 0 | 0 | 0 | 1 |
| DONSON | 1 | 0 | 0 | 0 | 0 | 0 | 0 | 0 | 1 |
| TSSK2 | 1 | 0 | 0 | 0 | 0 | 0 | 0 | 0 | 1 |
| SUGT1 | 1 | 0 | 0 | 0 | 0 | 0 | 0 | 0 | 1 |
| ELMOD3 | 1 | 0 | 0 | 0 | 0 | 0 | 0 | 0 | 1 |
| STK32C | 1 | 0 | 0 | 0 | 0 | 0 | 0 | 0 | 1 |
| AASDHPPT | 1 | 0 | 0 | 0 | 0 | 0 | 0 | 0 | 1 |
| MFSD10 | 1 | 0 | 0 | 0 | 0 | 0 | 0 | 0 | 1 |
| QPCT | 1 | 0 | 0 | 0 | 0 | 0 | 0 | 0 | 1 |
| PDK1 | 1 | 0 | 0 | 0 | 0 | 0 | 0 | 0 | 1 |
| UPF3B | 1 | 0 | 0 | 0 | 0 | 0 | 0 | 0 | 1 |
| MVK | 1 | 0 | 0 | 0 | 0 | 0 | 0 | 0 | 1 |
| PIGV | 1 | 0 | 0 | 0 | 0 | 0 | 0 | 0 | 1 |
| EFHA1 | 1 | 0 | 0 | 0 | 0 | 0 | 0 | 0 | 1 |
| REEP2 | 1 | 0 | 0 | 0 | 0 | 0 | 0 | 0 | 1 |

| | | | | | | | | | |
|---|---|---|---|---|---|---|---|---|---|
| HSD17B12 | 1 | 0 | 0 | 0 | 0 | 0 | 0 | 0 | 1 |
| PCCB | 1 | 0 | 0 | 0 | 0 | 0 | 0 | 0 | 1 |
| C14orf105 | 1 | 0 | 0 | 0 | 0 | 0 | 0 | 0 | 1 |
| KLF13 | 1 | 0 | 0 | 0 | 0 | 0 | 0 | 0 | 1 |
| HOXA3 | 1 | 0 | 0 | 0 | 0 | 0 | 0 | 0 | 1 |
| NR2E1 | 1 | 0 | 0 | 0 | 0 | 0 | 0 | 0 | 1 |
| CCNK | 1 | 0 | 0 | 0 | 0 | 0 | 0 | 0 | 1 |
| LONRF3 | 0 | 0 | 1 | 0 | 0 | 0 | 0 | 0 | 1 |
| WBP2NL | 1 | 0 | 0 | 0 | 0 | 0 | 0 | 0 | 1 |
| TSNAXIP1 | 1 | 0 | 0 | 0 | 0 | 0 | 0 | 0 | 1 |
| KLHL9 | 1 | 0 | 0 | 0 | 0 | 0 | 0 | 0 | 1 |
| FANCL | 1 | 0 | 0 | 0 | 0 | 0 | 0 | 0 | 1 |
| MAGEB1 | 1 | 0 | 0 | 0 | 0 | 0 | 0 | 0 | 1 |
| NEU3 | 1 | 0 | 0 | 0 | 0 | 0 | 0 | 0 | 1 |
| GPC1 | 1 | 0 | 0 | 0 | 0 | 0 | 0 | 0 | 1 |
| SFRP1 | 1 | 0 | 0 | 0 | 0 | 0 | 0 | 0 | 1 |
| HNRNPL | 1 | 0 | 0 | 0 | 0 | 0 | 0 | 0 | 1 |
| FRAT1 | 1 | 0 | 0 | 0 | 0 | 0 | 0 | 0 | 1 |
| ABHD1 | 1 | 0 | 0 | 0 | 0 | 0 | 0 | 0 | 1 |
| C7orf25 | 1 | 0 | 0 | 0 | 0 | 0 | 0 | 0 | 1 |
| BTNL2 | 1 | 0 | 0 | 0 | 0 | 0 | 0 | 0 | 1 |
| HAPLN3 | 1 | 0 | 0 | 0 | 0 | 0 | 0 | 0 | 1 |
| KRT18 | 1 | 0 | 0 | 0 | 0 | 0 | 0 | 0 | 1 |
| SLC20A1 | 1 | 0 | 0 | 0 | 0 | 0 | 0 | 0 | 1 |
| HADHA | 1 | 0 | 0 | 0 | 0 | 0 | 0 | 0 | 1 |
| ADAM10 | 1 | 0 | 0 | 0 | 0 | 0 | 0 | 0 | 1 |
| IFNA8 | 1 | 0 | 0 | 0 | 0 | 0 | 0 | 0 | 1 |
| TAF1B | 1 | 0 | 0 | 0 | 0 | 0 | 0 | 0 | 1 |
| KCNN1 | 1 | 0 | 0 | 0 | 0 | 0 | 0 | 0 | 1 |
| ZMAT1 | 1 | 0 | 0 | 0 | 0 | 0 | 0 | 0 | 1 |
| ECSIT | 1 | 0 | 0 | 0 | 0 | 0 | 0 | 0 | 1 |
| PCYOX1L | 1 | 0 | 0 | 0 | 0 | 0 | 0 | 0 | 1 |
| OTX1 | 1 | 0 | 0 | 0 | 0 | 0 | 0 | 0 | 1 |
| ADARB1 | 1 | 0 | 0 | 0 | 0 | 0 | 0 | 0 | 1 |
| BRF2 | 1 | 0 | 0 | 0 | 0 | 0 | 0 | 0 | 1 |
| CD4 | 1 | 0 | 0 | 0 | 0 | 0 | 0 | 0 | 1 |
| C3orf64 | 1 | 0 | 0 | 0 | 0 | 0 | 0 | 0 | 1 |
| ADH6 | 1 | 0 | 0 | 0 | 0 | 0 | 0 | 0 | 1 |
| PFKFB3 | 1 | 0 | 0 | 0 | 0 | 0 | 0 | 0 | 1 |
| SARS | 1 | 0 | 0 | 0 | 0 | 0 | 0 | 0 | 1 |
| SEPN1 | 1 | 0 | 0 | 0 | 0 | 0 | 0 | 0 | 1 |
| SNTA1 | 1 | 0 | 0 | 0 | 0 | 0 | 0 | 0 | 1 |
| HS6ST2 | 1 | 0 | 0 | 0 | 0 | 0 | 0 | 0 | 1 |
| SERPINB7 | 1 | 0 | 0 | 0 | 0 | 0 | 0 | 0 | 1 |
| TMEM229A | 1 | 0 | 0 | 0 | 0 | 0 | 0 | 0 | 1 |
| PNMA5 | 1 | 0 | 0 | 0 | 0 | 0 | 0 | 0 | 1 |

| | | | | | | | | | |
|---|---|---|---|---|---|---|---|---|---|
| SHH | 1 | 0 | 0 | 0 | 0 | 0 | 0 | 0 | 1 |
| SLC43A3 | 1 | 0 | 0 | 0 | 0 | 0 | 0 | 0 | 1 |
| GIT2 | 0 | 1 | 0 | 0 | 0 | 0 | 0 | 0 | 1 |
| GALNT11 | 1 | 0 | 0 | 0 | 0 | 0 | 0 | 0 | 1 |
| GRK1 | 1 | 0 | 0 | 0 | 0 | 0 | 0 | 0 | 1 |
| TMEM71 | 1 | 0 | 0 | 0 | 0 | 0 | 0 | 0 | 1 |
| CREB3L1 | 1 | 0 | 0 | 0 | 0 | 0 | 0 | 0 | 1 |
| PPP3CC | 1 | 0 | 0 | 0 | 0 | 0 | 0 | 0 | 1 |
| GMPPA | 1 | 0 | 0 | 0 | 0 | 0 | 0 | 0 | 1 |
| ZNF362 | 1 | 0 | 0 | 0 | 0 | 0 | 0 | 0 | 1 |
| MELK | 1 | 0 | 0 | 0 | 0 | 0 | 0 | 0 | 1 |
| GNL2 | 1 | 0 | 0 | 0 | 0 | 0 | 0 | 0 | 1 |
| SMURF1 | 1 | 0 | 0 | 0 | 0 | 0 | 0 | 0 | 1 |
| SLC32A1 | 1 | 0 | 0 | 0 | 0 | 0 | 0 | 0 | 1 |
| MAP3K3 | 1 | 0 | 0 | 0 | 0 | 0 | 0 | 0 | 1 |
| FOS | 1 | 0 | 0 | 0 | 0 | 0 | 0 | 0 | 1 |
| MIER1 | 1 | 0 | 0 | 0 | 0 | 0 | 0 | 0 | 1 |
| DGAT2 | 1 | 0 | 0 | 0 | 0 | 0 | 0 | 0 | 1 |
| CIB4 | 1 | 0 | 0 | 0 | 0 | 0 | 0 | 0 | 1 |
| CDK10 | 1 | 0 | 0 | 0 | 0 | 0 | 0 | 0 | 1 |
| RPL28 | 1 | 0 | 0 | 0 | 0 | 0 | 0 | 0 | 1 |
| PPM1K | 1 | 0 | 0 | 0 | 0 | 0 | 0 | 0 | 1 |
| TTYH2 | 1 | 0 | 0 | 0 | 0 | 0 | 0 | 0 | 1 |
| ZCCHC18 | 1 | 0 | 0 | 0 | 0 | 0 | 0 | 0 | 1 |
| QTRTD1 | 1 | 0 | 0 | 0 | 0 | 0 | 0 | 0 | 1 |
| CYP27A1 | 1 | 0 | 0 | 0 | 0 | 0 | 0 | 0 | 1 |
| HRASLS2 | 1 | 0 | 0 | 0 | 0 | 0 | 0 | 0 | 1 |
| FLYWCH1 | 0 | 0 | 0 | 0 | 1 | 0 | 0 | 0 | 1 |
| TK2 | 1 | 0 | 0 | 0 | 0 | 0 | 0 | 0 | 1 |
| CSPG5 | 1 | 0 | 0 | 0 | 0 | 0 | 0 | 0 | 1 |
| APOL5 | 1 | 0 | 0 | 0 | 0 | 0 | 0 | 0 | 1 |
| ZNF283 | 1 | 0 | 0 | 0 | 0 | 0 | 0 | 0 | 1 |
| C1orf27 | 1 | 0 | 0 | 0 | 0 | 0 | 0 | 0 | 1 |
| THUMPD2 | 1 | 0 | 0 | 0 | 0 | 0 | 0 | 0 | 1 |
| OR5D14 | 0 | 1 | 0 | 0 | 0 | 0 | 0 | 0 | 1 |
| TMEM161A | 1 | 0 | 0 | 0 | 0 | 0 | 0 | 0 | 1 |
| NCL | 1 | 0 | 0 | 0 | 0 | 0 | 0 | 0 | 1 |
| FETUB | 1 | 0 | 0 | 0 | 0 | 0 | 0 | 0 | 1 |
| SESN3 | 1 | 0 | 0 | 0 | 0 | 0 | 0 | 0 | 1 |
| LCOR | 1 | 0 | 0 | 0 | 0 | 0 | 0 | 0 | 1 |
| PCIF1 | 1 | 0 | 0 | 0 | 0 | 0 | 0 | 0 | 1 |
| NDRG1 | 1 | 0 | 0 | 0 | 0 | 0 | 0 | 0 | 1 |
| HRASLS5 | 1 | 0 | 0 | 0 | 0 | 0 | 0 | 0 | 1 |
| RBM22 | 1 | 0 | 0 | 0 | 0 | 0 | 0 | 0 | 1 |
| RAVER2 | 1 | 0 | 0 | 0 | 0 | 0 | 0 | 0 | 1 |
| RHOT2 | 1 | 0 | 0 | 0 | 0 | 0 | 0 | 0 | 1 |

| | | | | | | | | | |
|---|---|---|---|---|---|---|---|---|---|
| PSMC2 | 1 | 0 | 0 | 0 | 0 | 0 | 0 | 0 | 1 |
| CDC123 | 1 | 0 | 0 | 0 | 0 | 0 | 0 | 0 | 1 |
| GPT2 | 1 | 0 | 0 | 0 | 0 | 0 | 0 | 0 | 1 |
| DDX27 | 1 | 0 | 0 | 0 | 0 | 0 | 0 | 0 | 1 |
| RPS6KB1 | 1 | 0 | 0 | 0 | 0 | 0 | 0 | 0 | 1 |
| CAMK1 | 1 | 0 | 0 | 0 | 0 | 0 | 0 | 0 | 1 |
| SERPINE2 | 1 | 0 | 0 | 0 | 0 | 0 | 0 | 0 | 1 |
| CD3EAP | 1 | 0 | 0 | 0 | 0 | 0 | 0 | 0 | 1 |
| ERBB2 | 0 | 0 | 1 | 0 | 0 | 0 | 0 | 0 | 1 |
| LPPR1 | 1 | 0 | 0 | 0 | 0 | 0 | 0 | 0 | 1 |
| WEE1 | 1 | 0 | 0 | 0 | 0 | 0 | 0 | 0 | 1 |
| PDIA6 | 1 | 0 | 0 | 0 | 0 | 0 | 0 | 0 | 1 |
| SYT6 | 1 | 0 | 0 | 0 | 0 | 0 | 0 | 0 | 1 |
| EHD4 | 1 | 0 | 0 | 0 | 0 | 0 | 0 | 0 | 1 |
| TMPRSS11B | 0 | 0 | 0 | 0 | 1 | 0 | 0 | 0 | 1 |
| SPATS2 | 1 | 0 | 0 | 0 | 0 | 0 | 0 | 0 | 1 |
| ZNF675 | 1 | 0 | 0 | 0 | 0 | 0 | 0 | 0 | 1 |
| CDR2L | 1 | 0 | 0 | 0 | 0 | 0 | 0 | 0 | 1 |
| ICA1L | 1 | 0 | 0 | 0 | 0 | 0 | 0 | 0 | 1 |
| ANTXR1 | 1 | 0 | 0 | 0 | 0 | 0 | 0 | 0 | 1 |
| PPIE | 1 | 0 | 0 | 0 | 0 | 0 | 0 | 0 | 1 |
| STAP2 | 1 | 0 | 0 | 0 | 0 | 0 | 0 | 0 | 1 |
| CHST10 | 1 | 0 | 0 | 0 | 0 | 0 | 0 | 0 | 1 |
| FAM82A2 | 1 | 0 | 0 | 0 | 0 | 0 | 0 | 0 | 1 |
| PEX10 | 1 | 0 | 0 | 0 | 0 | 0 | 0 | 0 | 1 |
| PABPC4L | 1 | 0 | 0 | 0 | 0 | 0 | 0 | 0 | 1 |
| ZNF643 | 1 | 0 | 0 | 0 | 0 | 0 | 0 | 0 | 1 |
| GSK3B | 1 | 0 | 0 | 0 | 0 | 0 | 0 | 0 | 1 |
| RGL2 | 1 | 0 | 0 | 0 | 0 | 0 | 0 | 0 | 1 |
| FAM73B | 1 | 0 | 0 | 0 | 0 | 0 | 0 | 0 | 1 |
| NKX2-5 | 1 | 0 | 0 | 0 | 0 | 0 | 0 | 0 | 1 |
| ZNF20 | 1 | 0 | 0 | 0 | 0 | 0 | 0 | 0 | 1 |
| ARRDC3 | 1 | 0 | 0 | 0 | 0 | 0 | 0 | 0 | 1 |
| TMEM192 | 1 | 0 | 0 | 0 | 0 | 0 | 0 | 0 | 1 |
| KCMF1 | 1 | 0 | 0 | 0 | 0 | 0 | 0 | 0 | 1 |
| RARA | 1 | 0 | 0 | 0 | 0 | 0 | 0 | 0 | 1 |
| TSTA3 | 1 | 0 | 0 | 0 | 0 | 0 | 0 | 0 | 1 |
| AP1M1 | 1 | 0 | 0 | 0 | 0 | 0 | 0 | 0 | 1 |
| ZNF510 | 1 | 0 | 0 | 0 | 0 | 0 | 0 | 0 | 1 |
| DNAJB14 | 1 | 0 | 0 | 0 | 0 | 0 | 0 | 0 | 1 |
| ZNF324B | 1 | 0 | 0 | 0 | 0 | 0 | 0 | 0 | 1 |
| NR4A2 | 1 | 0 | 0 | 0 | 0 | 0 | 0 | 0 | 1 |
| MBD2 | 1 | 0 | 0 | 0 | 0 | 0 | 0 | 0 | 1 |
| ALDH18A1 | 1 | 0 | 0 | 0 | 0 | 0 | 0 | 0 | 1 |
| SLC35A5 | 1 | 0 | 0 | 0 | 0 | 0 | 0 | 0 | 1 |
| OPN5 | 1 | 0 | 0 | 0 | 0 | 0 | 0 | 0 | 1 |

| | | | | | | | | | |
|---|---|---|---|---|---|---|---|---|---|
| SLC16A12 | 1 | 0 | 0 | 0 | 0 | 0 | 0 | 0 | 1 |
| LIAS | 1 | 0 | 0 | 0 | 0 | 0 | 0 | 0 | 1 |
| ARHGAP18 | 1 | 0 | 0 | 0 | 0 | 0 | 0 | 0 | 1 |
| SLC15A4 | 0 | 1 | 0 | 0 | 0 | 0 | 0 | 0 | 1 |
| RNF146 | 1 | 0 | 0 | 0 | 0 | 0 | 0 | 0 | 1 |
| DPEP1 | 1 | 0 | 0 | 0 | 0 | 0 | 0 | 0 | 1 |
| SHBG | 1 | 0 | 0 | 0 | 0 | 0 | 0 | 0 | 1 |
| CPSF3 | 1 | 0 | 0 | 0 | 0 | 0 | 0 | 0 | 1 |
| FAR1 | 1 | 0 | 0 | 0 | 0 | 0 | 0 | 0 | 1 |
| CEP76 | 1 | 0 | 0 | 0 | 0 | 0 | 0 | 0 | 1 |
| C20orf160 | 1 | 0 | 0 | 0 | 0 | 0 | 0 | 0 | 1 |
| TNK1 | 1 | 0 | 0 | 0 | 0 | 0 | 0 | 0 | 1 |
| TFAP2E | 1 | 0 | 0 | 0 | 0 | 0 | 0 | 0 | 1 |
| TRPC1 | 1 | 0 | 0 | 0 | 0 | 0 | 0 | 0 | 1 |
| EARS2 | 1 | 0 | 0 | 0 | 0 | 0 | 0 | 0 | 1 |
| SRC | 1 | 0 | 0 | 0 | 0 | 0 | 0 | 0 | 1 |
| CCDC64 | 1 | 0 | 0 | 0 | 0 | 0 | 0 | 0 | 1 |
| FANK1 | 1 | 0 | 0 | 0 | 0 | 0 | 0 | 0 | 1 |
| KRTAP13-4 | 1 | 0 | 0 | 0 | 0 | 0 | 0 | 0 | 1 |
| CD55 | 1 | 0 | 0 | 0 | 0 | 0 | 0 | 0 | 1 |
| DDX21 | 1 | 0 | 0 | 0 | 0 | 0 | 0 | 0 | 1 |
| ZNF138 | 1 | 0 | 0 | 0 | 0 | 0 | 0 | 0 | 1 |
| ADAM17 | 1 | 0 | 0 | 0 | 0 | 0 | 0 | 0 | 1 |
| IMP4 | 1 | 0 | 0 | 0 | 0 | 0 | 0 | 0 | 1 |
| CPNE3 | 1 | 0 | 0 | 0 | 0 | 0 | 0 | 0 | 1 |
| MAP2K7 | 1 | 0 | 0 | 0 | 0 | 0 | 0 | 0 | 1 |
| KLHL10 | 1 | 0 | 0 | 0 | 0 | 0 | 0 | 0 | 1 |
| MEPE | 1 | 0 | 0 | 0 | 0 | 0 | 0 | 0 | 1 |
| OPRK1 | 1 | 0 | 0 | 0 | 0 | 0 | 0 | 0 | 1 |
| RPS6KA4 | 1 | 0 | 0 | 0 | 0 | 0 | 0 | 0 | 1 |
| HTR3B | 1 | 0 | 0 | 0 | 0 | 0 | 0 | 0 | 1 |
| RIC3 | 1 | 0 | 0 | 0 | 0 | 0 | 0 | 0 | 1 |
| IGSF11 | 1 | 0 | 0 | 0 | 0 | 0 | 0 | 0 | 1 |
| BIRC3 | 1 | 0 | 0 | 0 | 0 | 0 | 0 | 0 | 1 |
| ZNF343 | 1 | 0 | 0 | 0 | 0 | 0 | 0 | 0 | 1 |
| GPRASP1 | 0 | 1 | 0 | 0 | 0 | 0 | 0 | 0 | 1 |
| NPC2 | 1 | 0 | 0 | 0 | 0 | 0 | 0 | 0 | 1 |
| RABGEF1 | 1 | 0 | 0 | 0 | 0 | 0 | 0 | 0 | 1 |
| C12orf66 | 1 | 0 | 0 | 0 | 0 | 0 | 0 | 0 | 1 |
| BCKDHA | 1 | 0 | 0 | 0 | 0 | 0 | 0 | 0 | 1 |
| FAM96A | 1 | 0 | 0 | 0 | 0 | 0 | 0 | 0 | 1 |
| PRICKLE2 | 0 | 1 | 0 | 0 | 0 | 0 | 0 | 0 | 1 |
| CAPN7 | 1 | 0 | 0 | 0 | 0 | 0 | 0 | 0 | 1 |
| AQP1 | 1 | 0 | 0 | 0 | 0 | 0 | 0 | 0 | 1 |
| HOXA4 | 1 | 0 | 0 | 0 | 0 | 0 | 0 | 0 | 1 |
| CYTH1 | 1 | 0 | 0 | 0 | 0 | 0 | 0 | 0 | 1 |

| | | | | | | | | | |
|---|---|---|---|---|---|---|---|---|---|
| DEF8 | 1 | 0 | 0 | 0 | 0 | 0 | 0 | 0 | 1 |
| ZUFSP | 1 | 0 | 0 | 0 | 0 | 0 | 0 | 0 | 1 |
| ANTXR2 | 1 | 0 | 0 | 0 | 0 | 0 | 0 | 0 | 1 |
| AIFM1 | 1 | 0 | 0 | 0 | 0 | 0 | 0 | 0 | 1 |
| BTBD16 | 1 | 0 | 0 | 0 | 0 | 0 | 0 | 0 | 1 |
| TTLL1 | 1 | 0 | 0 | 0 | 0 | 0 | 0 | 0 | 1 |
| CACNB3 | 1 | 0 | 0 | 0 | 0 | 0 | 0 | 0 | 1 |
| MAGEA4 | 1 | 0 | 0 | 0 | 0 | 0 | 0 | 0 | 1 |
| NRBP2 | 1 | 0 | 0 | 0 | 0 | 0 | 0 | 0 | 1 |
| CTSZ | 1 | 0 | 0 | 0 | 0 | 0 | 0 | 0 | 1 |
| SLC10A6 | 1 | 0 | 0 | 0 | 0 | 0 | 0 | 0 | 1 |
| A1BG | 1 | 0 | 0 | 0 | 0 | 0 | 0 | 0 | 1 |
| NOP56 | 1 | 0 | 0 | 0 | 0 | 0 | 0 | 0 | 1 |
| NCSTN | 0 | 1 | 0 | 0 | 0 | 0 | 0 | 0 | 1 |
| REEP3 | 1 | 0 | 0 | 0 | 0 | 0 | 0 | 0 | 1 |
| LOC375190 | 1 | 0 | 0 | 0 | 0 | 0 | 0 | 0 | 1 |
| ATP2B4 | 0 | 1 | 0 | 0 | 0 | 0 | 0 | 0 | 1 |
| EFS | 1 | 0 | 0 | 0 | 0 | 0 | 0 | 0 | 1 |
| ZBTB8A | 1 | 0 | 0 | 0 | 0 | 0 | 0 | 0 | 1 |
| GRAMD3 | 1 | 0 | 0 | 0 | 0 | 0 | 0 | 0 | 1 |
| RDX | 1 | 0 | 0 | 0 | 0 | 0 | 0 | 0 | 1 |
| MAGEB4 | 1 | 0 | 0 | 0 | 0 | 0 | 0 | 0 | 1 |
| G3BP2 | 1 | 0 | 0 | 0 | 0 | 0 | 0 | 0 | 1 |
| TMC6 | 1 | 0 | 0 | 0 | 0 | 0 | 0 | 0 | 1 |
| UEVLD | 1 | 0 | 0 | 0 | 0 | 0 | 0 | 0 | 1 |
| PDK4 | 1 | 0 | 0 | 0 | 0 | 0 | 0 | 0 | 1 |
| GPR22 | 1 | 0 | 0 | 0 | 0 | 0 | 0 | 0 | 1 |
| F10 | 1 | 0 | 0 | 0 | 0 | 0 | 0 | 0 | 1 |
| FGFR1OP | 1 | 0 | 0 | 0 | 0 | 0 | 0 | 0 | 1 |
| RGS11 | 1 | 0 | 0 | 0 | 0 | 0 | 0 | 0 | 1 |
| CCDC99 | 1 | 0 | 0 | 0 | 0 | 0 | 0 | 0 | 1 |
| CDS2 | 0 | 1 | 0 | 0 | 0 | 0 | 0 | 0 | 1 |
| FAM114A1 | 1 | 0 | 0 | 0 | 0 | 0 | 0 | 0 | 1 |
| KRTAP27-1 | 1 | 0 | 0 | 0 | 0 | 0 | 0 | 0 | 1 |
| SMAP1 | 1 | 0 | 0 | 0 | 0 | 0 | 0 | 0 | 1 |
| CDC7 | 1 | 0 | 0 | 0 | 0 | 0 | 0 | 0 | 1 |
| CDT1 | 1 | 0 | 0 | 0 | 0 | 0 | 0 | 0 | 1 |
| MTL5 | 1 | 0 | 0 | 0 | 0 | 0 | 0 | 0 | 1 |
| PGAP2 | 1 | 0 | 0 | 0 | 0 | 0 | 0 | 0 | 1 |
| FTO | 1 | 0 | 0 | 0 | 0 | 0 | 0 | 0 | 1 |
| HOMEZ | 1 | 0 | 0 | 0 | 0 | 0 | 0 | 0 | 1 |
| HOXD9 | 1 | 0 | 0 | 0 | 0 | 0 | 0 | 0 | 1 |
| TMEM194A | 1 | 0 | 0 | 0 | 0 | 0 | 0 | 0 | 1 |
| BTN3A1 | 1 | 0 | 0 | 0 | 0 | 0 | 0 | 0 | 1 |
| GSDMD | 1 | 0 | 0 | 0 | 0 | 0 | 0 | 0 | 1 |
| KRTAP10-11 | 1 | 0 | 0 | 0 | 0 | 0 | 0 | 0 | 1 |

| | | | | | | | | | |
|---|---|---|---|---|---|---|---|---|---|
| PAX7 | 1 | 0 | 0 | 0 | 0 | 0 | 0 | 0 | 1 |
| ZGPAT | 1 | 0 | 0 | 0 | 0 | 0 | 0 | 0 | 1 |
| MUC13 | 1 | 0 | 0 | 0 | 0 | 0 | 0 | 0 | 1 |
| HNRNPK | 1 | 0 | 0 | 0 | 0 | 0 | 0 | 0 | 1 |
| ZNF860 | 1 | 0 | 0 | 0 | 0 | 0 | 0 | 0 | 1 |
| NUDT13 | 1 | 0 | 0 | 0 | 0 | 0 | 0 | 0 | 1 |
| XKR7 | 1 | 0 | 0 | 0 | 0 | 0 | 0 | 0 | 1 |
| IL17RD | 1 | 0 | 0 | 0 | 0 | 0 | 0 | 0 | 1 |
| PPAP2C | 1 | 0 | 0 | 0 | 0 | 0 | 0 | 0 | 1 |
| C17orf96 | 1 | 0 | 0 | 0 | 0 | 0 | 0 | 0 | 1 |
| SLC6A20 | 1 | 0 | 0 | 0 | 0 | 0 | 0 | 0 | 1 |
| INTS9 | 1 | 0 | 0 | 0 | 0 | 0 | 0 | 0 | 1 |
| METTL4 | 1 | 0 | 0 | 0 | 0 | 0 | 0 | 0 | 1 |
| CYP4B1 | 1 | 0 | 0 | 0 | 0 | 0 | 0 | 0 | 1 |
| ZNF786 | 1 | 0 | 0 | 0 | 0 | 0 | 0 | 0 | 1 |
| SLC33A1 | 1 | 0 | 0 | 0 | 0 | 0 | 0 | 0 | 1 |
| RNF25 | 1 | 0 | 0 | 0 | 0 | 0 | 0 | 0 | 1 |
| PIGN | 1 | 0 | 0 | 0 | 0 | 0 | 0 | 0 | 1 |
| TEAD2 | 1 | 0 | 0 | 0 | 0 | 0 | 0 | 0 | 1 |
| DCBLD1 | 1 | 0 | 0 | 0 | 0 | 0 | 0 | 0 | 1 |
| NR1H3 | 1 | 0 | 0 | 0 | 0 | 0 | 0 | 0 | 1 |
| PODNL1 | 1 | 0 | 0 | 0 | 0 | 0 | 0 | 0 | 1 |
| ZBTB34 | 1 | 0 | 0 | 0 | 0 | 0 | 0 | 0 | 1 |
| SLC18A3 | 1 | 0 | 0 | 0 | 0 | 0 | 0 | 0 | 1 |
| CEBPZ | 0 | 1 | 0 | 0 | 0 | 0 | 0 | 0 | 1 |
| NEU4 | 1 | 0 | 0 | 0 | 0 | 0 | 0 | 0 | 1 |
| GNAT2 | 1 | 0 | 0 | 0 | 0 | 0 | 0 | 0 | 1 |
| STRADB | 1 | 0 | 0 | 0 | 0 | 0 | 0 | 0 | 1 |
| SNX15 | 1 | 0 | 0 | 0 | 0 | 0 | 0 | 0 | 1 |
| CHODL | 1 | 0 | 0 | 0 | 0 | 0 | 0 | 0 | 1 |
| MAPKAP1 | 1 | 0 | 0 | 0 | 0 | 0 | 0 | 0 | 1 |
| CHID1 | 1 | 0 | 0 | 0 | 0 | 0 | 0 | 0 | 1 |
| ZNF596 | 1 | 0 | 0 | 0 | 0 | 0 | 0 | 0 | 1 |
| NR4A3 | 1 | 0 | 0 | 0 | 0 | 0 | 0 | 0 | 1 |
| MS4A5 | 1 | 0 | 0 | 0 | 0 | 0 | 0 | 0 | 1 |
| GPR135 | 1 | 0 | 0 | 0 | 0 | 0 | 0 | 0 | 1 |
| CCHCR1 | 1 | 0 | 0 | 0 | 0 | 0 | 0 | 0 | 1 |
| PHF19 | 1 | 0 | 0 | 0 | 0 | 0 | 0 | 0 | 1 |
| DOLK | 1 | 0 | 0 | 0 | 0 | 0 | 0 | 0 | 1 |
| SYTL4 | 1 | 0 | 0 | 0 | 0 | 0 | 0 | 0 | 1 |
| PIGZ | 1 | 0 | 0 | 0 | 0 | 0 | 0 | 0 | 1 |
| IKZF3 | 1 | 0 | 0 | 0 | 0 | 0 | 0 | 0 | 1 |
| KBTBD12 | 1 | 0 | 0 | 0 | 0 | 0 | 0 | 0 | 1 |
| RFC4 | 1 | 0 | 0 | 0 | 0 | 0 | 0 | 0 | 1 |
| TIGD1 | 1 | 0 | 0 | 0 | 0 | 0 | 0 | 0 | 1 |
| PEF1 | 1 | 0 | 0 | 0 | 0 | 0 | 0 | 0 | 1 |

| | | | | | | | | | |
|---|---|---|---|---|---|---|---|---|---|
| CAMKK1 | 1 | 0 | 0 | 0 | 0 | 0 | 0 | 0 | 1 |
| LMAN1 | 1 | 0 | 0 | 0 | 0 | 0 | 0 | 0 | 1 |
| P4HA2 | 1 | 0 | 0 | 0 | 0 | 0 | 0 | 0 | 1 |
| ACTR3 | 1 | 0 | 0 | 0 | 0 | 0 | 0 | 0 | 1 |
| SOX13 | 1 | 0 | 0 | 0 | 0 | 0 | 0 | 0 | 1 |
| ENPP5 | 1 | 0 | 0 | 0 | 0 | 0 | 0 | 0 | 1 |
| POU2F3 | 1 | 0 | 0 | 0 | 0 | 0 | 0 | 0 | 1 |
| DCAF17 | 1 | 0 | 0 | 0 | 0 | 0 | 0 | 0 | 1 |
| PPP2R2C | 1 | 0 | 0 | 0 | 0 | 0 | 0 | 0 | 1 |
| CDKN2A | 1 | 0 | 0 | 0 | 0 | 0 | 0 | 0 | 1 |
| APOBEC3G | 1 | 0 | 0 | 0 | 0 | 0 | 0 | 0 | 1 |
| RAB31 | 1 | 0 | 0 | 0 | 0 | 0 | 0 | 0 | 1 |
| CHPT1 | 1 | 0 | 0 | 0 | 0 | 0 | 0 | 0 | 1 |
| WSB1 | 1 | 0 | 0 | 0 | 0 | 0 | 0 | 0 | 1 |
| SH2D7 | 1 | 0 | 0 | 0 | 0 | 0 | 0 | 0 | 1 |
| PURG | 1 | 0 | 0 | 0 | 0 | 0 | 0 | 0 | 1 |
| PLIN5 | 1 | 0 | 0 | 0 | 0 | 0 | 0 | 0 | 1 |
| CD36 | 1 | 0 | 0 | 0 | 0 | 0 | 0 | 0 | 1 |
| ANKMY2 | 1 | 0 | 0 | 0 | 0 | 0 | 0 | 0 | 1 |
| ZNF782 | 1 | 0 | 0 | 0 | 0 | 0 | 0 | 0 | 1 |
| TRIM69 | 1 | 0 | 0 | 0 | 0 | 0 | 0 | 0 | 1 |
| C12orf50 | 1 | 0 | 0 | 0 | 0 | 0 | 0 | 0 | 1 |
| CEACAM16 | 1 | 0 | 0 | 0 | 0 | 0 | 0 | 0 | 1 |
| ATL2 | 1 | 0 | 0 | 0 | 0 | 0 | 0 | 0 | 1 |
| FAM78B | 1 | 0 | 0 | 0 | 0 | 0 | 0 | 0 | 1 |
| IQCG | 1 | 0 | 0 | 0 | 0 | 0 | 0 | 0 | 1 |
| GDF9 | 1 | 0 | 0 | 0 | 0 | 0 | 0 | 0 | 1 |
| INPP5A | 1 | 0 | 0 | 0 | 0 | 0 | 0 | 0 | 1 |
| U2AF2 | 1 | 0 | 0 | 0 | 0 | 0 | 0 | 0 | 1 |
| ACSF3 | 1 | 0 | 0 | 0 | 0 | 0 | 0 | 0 | 1 |
| UGT8 | 1 | 0 | 0 | 0 | 0 | 0 | 0 | 0 | 1 |
| MTSS1L | 1 | 0 | 0 | 0 | 0 | 0 | 0 | 0 | 1 |
| PBX1 | 1 | 0 | 0 | 0 | 0 | 0 | 0 | 0 | 1 |
| CDS1 | 1 | 0 | 0 | 0 | 0 | 0 | 0 | 0 | 1 |
| OR52J3 | 1 | 0 | 0 | 0 | 0 | 0 | 0 | 0 | 1 |
| ZNF620 | 1 | 0 | 0 | 0 | 0 | 0 | 0 | 0 | 1 |
| CXorf64 | 1 | 0 | 0 | 0 | 0 | 0 | 0 | 0 | 1 |
| ANGPTL5 | 1 | 0 | 0 | 0 | 0 | 0 | 0 | 0 | 1 |
| SLC38A8 | 1 | 0 | 0 | 0 | 0 | 0 | 0 | 0 | 1 |
| MGRN1 | 1 | 0 | 0 | 0 | 0 | 0 | 0 | 0 | 1 |
| IL28RA | 1 | 0 | 0 | 0 | 0 | 0 | 0 | 0 | 1 |
| PSTK | 1 | 0 | 0 | 0 | 0 | 0 | 0 | 0 | 1 |
| MCCC2 | 1 | 0 | 0 | 0 | 0 | 0 | 0 | 0 | 1 |
| PFKFB2 | 0 | 0 | 1 | 0 | 0 | 0 | 0 | 0 | 1 |
| SENP2 | 1 | 0 | 0 | 0 | 0 | 0 | 0 | 0 | 1 |
| DRD1 | 1 | 0 | 0 | 0 | 0 | 0 | 0 | 0 | 1 |

| | | | | | | | | | |
|---|---|---|---|---|---|---|---|---|---|
| HLCS | 1 | 0 | 0 | 0 | 0 | 0 | 0 | 0 | 1 |
| HS2ST1 | 1 | 0 | 0 | 0 | 0 | 0 | 0 | 0 | 1 |
| TBCCD1 | 1 | 0 | 0 | 0 | 0 | 0 | 0 | 0 | 1 |
| CEACAM21 | 1 | 0 | 0 | 0 | 0 | 0 | 0 | 0 | 1 |
| NOXA1 | 1 | 0 | 0 | 0 | 0 | 0 | 0 | 0 | 1 |
| PYGO1 | 1 | 0 | 0 | 0 | 0 | 0 | 0 | 0 | 1 |
| ZNF696 | 1 | 0 | 0 | 0 | 0 | 0 | 0 | 0 | 1 |
| INHBC | 1 | 0 | 0 | 0 | 0 | 0 | 0 | 0 | 1 |
| UBE2L6 | 1 | 0 | 0 | 0 | 0 | 0 | 0 | 0 | 1 |
| IP6K1 | 1 | 0 | 0 | 0 | 0 | 0 | 0 | 0 | 1 |
| TRAF3IP1 | 1 | 0 | 0 | 0 | 0 | 0 | 0 | 0 | 1 |
| RNF43 | 1 | 0 | 0 | 0 | 0 | 0 | 0 | 0 | 1 |
| ELL | 1 | 0 | 0 | 0 | 0 | 0 | 0 | 0 | 1 |
| TRIM4 | 1 | 0 | 0 | 0 | 0 | 0 | 0 | 0 | 1 |
| GNL3L | 1 | 0 | 0 | 0 | 0 | 0 | 0 | 0 | 1 |
| MTERFD1 | 1 | 0 | 0 | 0 | 0 | 0 | 0 | 0 | 1 |
| ABCD4 | 1 | 0 | 0 | 0 | 0 | 0 | 0 | 0 | 1 |
| GALT | 1 | 0 | 0 | 0 | 0 | 0 | 0 | 0 | 1 |
| RAD17 | 1 | 0 | 0 | 0 | 0 | 0 | 0 | 0 | 1 |
| KRT26 | 1 | 0 | 0 | 0 | 0 | 0 | 0 | 0 | 1 |
| ZNF446 | 1 | 0 | 0 | 0 | 0 | 0 | 0 | 0 | 1 |
| DCST1 | 1 | 0 | 0 | 0 | 0 | 0 | 0 | 0 | 1 |
| SPRED2 | 1 | 0 | 0 | 0 | 0 | 0 | 0 | 0 | 1 |
| NR6A1 | 1 | 0 | 0 | 0 | 0 | 0 | 0 | 0 | 1 |
| SLC16A13 | 1 | 0 | 0 | 0 | 0 | 0 | 0 | 0 | 1 |
| FLRT3 | 1 | 0 | 0 | 0 | 0 | 0 | 0 | 0 | 1 |
| TRPC4AP | 1 | 0 | 0 | 0 | 0 | 0 | 0 | 0 | 1 |
| C19orf46 | 1 | 0 | 0 | 0 | 0 | 0 | 0 | 0 | 1 |
| RENBP | 0 | 1 | 0 | 0 | 0 | 0 | 0 | 0 | 1 |
| WDR93 | 1 | 0 | 0 | 0 | 0 | 0 | 0 | 0 | 1 |
| RASA3 | 1 | 0 | 0 | 0 | 0 | 0 | 0 | 0 | 1 |
| CHRND | 1 | 0 | 0 | 0 | 0 | 0 | 0 | 0 | 1 |
| EPB41L4A | 1 | 0 | 0 | 0 | 0 | 0 | 0 | 0 | 1 |
| STK38 | 1 | 0 | 0 | 0 | 0 | 0 | 0 | 0 | 1 |
| RASD2 | 1 | 0 | 0 | 0 | 0 | 0 | 0 | 0 | 1 |
| ZNF550 | 1 | 0 | 0 | 0 | 0 | 0 | 0 | 0 | 1 |
| FLAD1 | 1 | 0 | 0 | 0 | 0 | 0 | 0 | 0 | 1 |
| SERPINA3 | 1 | 0 | 0 | 0 | 0 | 0 | 0 | 0 | 1 |
| CDC37 | 1 | 0 | 0 | 0 | 0 | 0 | 0 | 0 | 1 |
| HTR7 | 1 | 0 | 0 | 0 | 0 | 0 | 0 | 0 | 1 |
| ENTPD8 | 1 | 0 | 0 | 0 | 0 | 0 | 0 | 0 | 1 |
| RUNDC3B | 1 | 0 | 0 | 0 | 0 | 0 | 0 | 0 | 1 |
| ANKH | 1 | 0 | 0 | 0 | 0 | 0 | 0 | 0 | 1 |
| TMEM87A | 1 | 0 | 0 | 0 | 0 | 0 | 0 | 0 | 1 |
| ATF7 | 1 | 0 | 0 | 0 | 0 | 0 | 0 | 0 | 1 |
| C15orf27 | 1 | 0 | 0 | 0 | 0 | 0 | 0 | 0 | 1 |

| | | | | | | | | | |
|---|---|---|---|---|---|---|---|---|---|
| ZNF682 | 1 | 0 | 0 | 0 | 0 | 0 | 0 | 0 | 1 |
| USP21 | 1 | 0 | 0 | 0 | 0 | 0 | 0 | 0 | 1 |
| FKBP7 | 1 | 0 | 0 | 0 | 0 | 0 | 0 | 0 | 1 |
| DDX52 | 1 | 0 | 0 | 0 | 0 | 0 | 0 | 0 | 1 |
| ENTPD4 | 1 | 0 | 0 | 0 | 0 | 0 | 0 | 0 | 1 |
| FGFRL1 | 1 | 0 | 0 | 0 | 0 | 0 | 0 | 0 | 1 |
| IGDCC3 | 1 | 0 | 0 | 0 | 0 | 0 | 0 | 0 | 1 |
| UBASH3B | 1 | 0 | 0 | 0 | 0 | 0 | 0 | 0 | 1 |
| FRS2 | 1 | 0 | 0 | 0 | 0 | 0 | 0 | 0 | 1 |
| FAM105A | 1 | 0 | 0 | 0 | 0 | 0 | 0 | 0 | 1 |
| TCEA3 | 1 | 0 | 0 | 0 | 0 | 0 | 0 | 0 | 1 |
| AMPD3 | 1 | 0 | 0 | 0 | 0 | 0 | 0 | 0 | 1 |
| KRT82 | 1 | 0 | 0 | 0 | 0 | 0 | 0 | 0 | 1 |
| DPH5 | 0 | 1 | 0 | 0 | 0 | 0 | 0 | 0 | 1 |
| TUBB1 | 1 | 0 | 0 | 0 | 0 | 0 | 0 | 0 | 1 |
| OR51L1 | 1 | 0 | 0 | 0 | 0 | 0 | 0 | 0 | 1 |
| CCDC82 | 1 | 0 | 0 | 0 | 0 | 0 | 0 | 0 | 1 |
| RHOBTB3 | 1 | 0 | 0 | 0 | 0 | 0 | 0 | 0 | 1 |
| FAM82A1 | 1 | 0 | 0 | 0 | 0 | 0 | 0 | 0 | 1 |
| MRS2 | 1 | 0 | 0 | 0 | 0 | 0 | 0 | 0 | 1 |
| WNT16 | 1 | 0 | 0 | 0 | 0 | 0 | 0 | 0 | 1 |
| DDA1 | 1 | 0 | 0 | 0 | 0 | 0 | 0 | 0 | 1 |
| GUSB | 1 | 0 | 0 | 0 | 0 | 0 | 0 | 0 | 1 |
| RPS6KB2 | 1 | 0 | 0 | 0 | 0 | 0 | 0 | 0 | 1 |
| RFWD3 | 1 | 0 | 0 | 0 | 0 | 0 | 0 | 0 | 1 |
| OR52E2 | 1 | 0 | 0 | 0 | 0 | 0 | 0 | 0 | 1 |
| MTDH | 1 | 0 | 0 | 0 | 0 | 0 | 0 | 0 | 1 |
| LILRA4 | 1 | 0 | 0 | 0 | 0 | 0 | 0 | 0 | 1 |
| FBXO33 | 1 | 0 | 0 | 0 | 0 | 0 | 0 | 0 | 1 |
| RELA | 1 | 0 | 0 | 0 | 0 | 0 | 0 | 0 | 1 |
| RIPK2 | 1 | 0 | 0 | 0 | 0 | 0 | 0 | 0 | 1 |
| FAM189A1 | 1 | 0 | 0 | 0 | 0 | 0 | 0 | 0 | 1 |
| LRRC10 | 1 | 0 | 0 | 0 | 0 | 0 | 0 | 0 | 1 |
| RANBP10 | 1 | 0 | 0 | 0 | 0 | 0 | 0 | 0 | 1 |
| TCP11 | 1 | 0 | 0 | 0 | 0 | 0 | 0 | 0 | 1 |
| TRIM11 | 1 | 0 | 0 | 0 | 0 | 0 | 0 | 0 | 1 |
| KCNK13 | 1 | 0 | 0 | 0 | 0 | 0 | 0 | 0 | 1 |
| DCT | 1 | 0 | 0 | 0 | 0 | 0 | 0 | 0 | 1 |
| KLHL5 | 1 | 0 | 0 | 0 | 0 | 0 | 0 | 0 | 1 |
| CCDC78 | 1 | 0 | 0 | 0 | 0 | 0 | 0 | 0 | 1 |
| QRSL1 | 1 | 0 | 0 | 0 | 0 | 0 | 0 | 0 | 1 |
| CYTH3 | 1 | 0 | 0 | 0 | 0 | 0 | 0 | 0 | 1 |
| CYP24A1 | 1 | 0 | 0 | 0 | 0 | 0 | 0 | 0 | 1 |
| LPCAT2 | 1 | 0 | 0 | 0 | 0 | 0 | 0 | 0 | 1 |
| PARN | 1 | 0 | 0 | 0 | 0 | 0 | 0 | 0 | 1 |
| OGFOD1 | 1 | 0 | 0 | 0 | 0 | 0 | 0 | 0 | 1 |

| | | | | | | | | | |
|---|---|---|---|---|---|---|---|---|---|
| HSF5 | 1 | 0 | 0 | 0 | 0 | 0 | 0 | 0 | 1 |
| IDUA | 1 | 0 | 0 | 0 | 0 | 0 | 0 | 0 | 1 |
| STRBP | 1 | 0 | 0 | 0 | 0 | 0 | 0 | 0 | 1 |
| MIDN | 1 | 0 | 0 | 0 | 0 | 0 | 0 | 0 | 1 |
| APEH | 1 | 0 | 0 | 0 | 0 | 0 | 0 | 0 | 1 |
| USP10 | 1 | 0 | 0 | 0 | 0 | 0 | 0 | 0 | 1 |
| PPP5C | 1 | 0 | 0 | 0 | 0 | 0 | 0 | 0 | 1 |
| TAAR6 | 1 | 0 | 0 | 0 | 0 | 0 | 0 | 0 | 1 |
| C9orf24 | 1 | 0 | 0 | 0 | 0 | 0 | 0 | 0 | 1 |
| PRR16 | 1 | 0 | 0 | 0 | 0 | 0 | 0 | 0 | 1 |
| PTDSS2 | 1 | 0 | 0 | 0 | 0 | 0 | 0 | 0 | 1 |
| HNF4A | 1 | 0 | 0 | 0 | 0 | 0 | 0 | 0 | 1 |
| ITGB5 | 1 | 0 | 0 | 0 | 0 | 0 | 0 | 0 | 1 |
| FAM132A | 1 | 0 | 0 | 0 | 0 | 0 | 0 | 0 | 1 |
| OTX2 | 1 | 0 | 0 | 0 | 0 | 0 | 0 | 0 | 1 |
| MARK3 | 1 | 0 | 0 | 0 | 0 | 0 | 0 | 0 | 1 |
| HIST1H2AB | 1 | 0 | 0 | 0 | 0 | 0 | 0 | 0 | 1 |
| CXorf58 | 1 | 0 | 0 | 0 | 0 | 0 | 0 | 0 | 1 |
| ST6GALNAC2 | 1 | 0 | 0 | 0 | 0 | 0 | 0 | 0 | 1 |
| CDC25A | 1 | 0 | 0 | 0 | 0 | 0 | 0 | 0 | 1 |
| AIMP1 | 1 | 0 | 0 | 0 | 0 | 0 | 0 | 0 | 1 |
| PRSS16 | 1 | 0 | 0 | 0 | 0 | 0 | 0 | 0 | 1 |
| BTN1A1 | 1 | 0 | 0 | 0 | 0 | 0 | 0 | 0 | 1 |
| MATR3 | 1 | 0 | 0 | 0 | 0 | 0 | 0 | 0 | 1 |
| FAM47E | 1 | 0 | 0 | 0 | 0 | 0 | 0 | 0 | 1 |
| RIOK2 | 1 | 0 | 0 | 0 | 0 | 0 | 0 | 0 | 1 |
| CHI3L1 | 1 | 0 | 0 | 0 | 0 | 0 | 0 | 0 | 1 |
| C6orf211 | 1 | 0 | 0 | 0 | 0 | 0 | 0 | 0 | 1 |
| APTX | 1 | 0 | 0 | 0 | 0 | 0 | 0 | 0 | 1 |
| ARHGDIB | 1 | 0 | 0 | 0 | 0 | 0 | 0 | 0 | 1 |
| SOX1 | 1 | 0 | 0 | 0 | 0 | 0 | 0 | 0 | 1 |
| RAB11FIP2 | 1 | 0 | 0 | 0 | 0 | 0 | 0 | 0 | 1 |
| CASP8 | 1 | 0 | 0 | 0 | 0 | 0 | 0 | 0 | 1 |
| ZBTB42 | 1 | 0 | 0 | 0 | 0 | 0 | 0 | 0 | 1 |
| FAM151A | 1 | 0 | 0 | 0 | 0 | 0 | 0 | 0 | 1 |
| MICALL2 | 1 | 0 | 0 | 0 | 0 | 0 | 0 | 0 | 1 |
| ABI3 | 1 | 0 | 0 | 0 | 0 | 0 | 0 | 0 | 1 |
| CALCOCO2 | 1 | 0 | 0 | 0 | 0 | 0 | 0 | 0 | 1 |
| SNAP47 | 1 | 0 | 0 | 0 | 0 | 0 | 0 | 0 | 1 |
| LRRC1 | 1 | 0 | 0 | 0 | 0 | 0 | 0 | 0 | 1 |
| DDX56 | 1 | 0 | 0 | 0 | 0 | 0 | 0 | 0 | 1 |
| SDCCAG3 | 1 | 0 | 0 | 0 | 0 | 0 | 0 | 0 | 1 |
| OR52E4 | 1 | 0 | 0 | 0 | 0 | 0 | 0 | 0 | 1 |
| OR7E24 | 1 | 0 | 0 | 0 | 0 | 0 | 0 | 0 | 1 |
| PPM1A | 1 | 0 | 0 | 0 | 0 | 0 | 0 | 0 | 1 |
| ZNF121 | 1 | 0 | 0 | 0 | 0 | 0 | 0 | 0 | 1 |

| | | | | | | | | | |
|---|---|---|---|---|---|---|---|---|---|
| C5orf22 | 1 | 0 | 0 | 0 | 0 | 0 | 0 | 0 | 1 |
| HTR3A | 0 | 1 | 0 | 0 | 0 | 0 | 0 | 0 | 1 |
| DPP8 | 1 | 0 | 0 | 0 | 0 | 0 | 0 | 0 | 1 |
| NAPB | 1 | 0 | 0 | 0 | 0 | 0 | 0 | 0 | 1 |
| XYLT2 | 1 | 0 | 0 | 0 | 0 | 0 | 0 | 0 | 1 |
| LINGO4 | 1 | 0 | 0 | 0 | 0 | 0 | 0 | 0 | 1 |
| ENDOD1 | 1 | 0 | 0 | 0 | 0 | 0 | 0 | 0 | 1 |
| TBC1D10C | 1 | 0 | 0 | 0 | 0 | 0 | 0 | 0 | 1 |
| CCDC76 | 1 | 0 | 0 | 0 | 0 | 0 | 0 | 0 | 1 |
| TBC1D16 | 1 | 0 | 0 | 0 | 0 | 0 | 0 | 0 | 1 |
| PNPLA3 | 1 | 0 | 0 | 0 | 0 | 0 | 0 | 0 | 1 |
| MUTYH | 1 | 0 | 0 | 0 | 0 | 0 | 0 | 0 | 1 |
| CAMK1D | 1 | 0 | 0 | 0 | 0 | 0 | 0 | 0 | 1 |
| SAMD1 | 1 | 0 | 0 | 0 | 0 | 0 | 0 | 0 | 1 |
| AHCYL2 | 1 | 0 | 0 | 0 | 0 | 0 | 0 | 0 | 1 |
| SUMF2 | 1 | 0 | 0 | 0 | 0 | 0 | 0 | 0 | 1 |
| FBXW5 | 1 | 0 | 0 | 0 | 0 | 0 | 0 | 0 | 1 |
| DMAP1 | 1 | 0 | 0 | 0 | 0 | 0 | 0 | 0 | 1 |
| CTPS2 | 1 | 0 | 0 | 0 | 0 | 0 | 0 | 0 | 1 |
| WBP11 | 1 | 0 | 0 | 0 | 0 | 0 | 0 | 0 | 1 |
| CREB3L3 | 1 | 0 | 0 | 0 | 0 | 0 | 0 | 0 | 1 |
| CPOX | 1 | 0 | 0 | 0 | 0 | 0 | 0 | 0 | 1 |
| DENND2D | 1 | 0 | 0 | 0 | 0 | 0 | 0 | 0 | 1 |
| ATF2 | 1 | 0 | 0 | 0 | 0 | 0 | 0 | 0 | 1 |
| IL20RA | 1 | 0 | 0 | 0 | 0 | 0 | 0 | 0 | 1 |
| FGG | 1 | 0 | 0 | 0 | 0 | 0 | 0 | 0 | 1 |
| NPTXR | 1 | 0 | 0 | 0 | 0 | 0 | 0 | 0 | 1 |
| FBXL2 | 1 | 0 | 0 | 0 | 0 | 0 | 0 | 0 | 1 |
| TMEM145 | 1 | 0 | 0 | 0 | 0 | 0 | 0 | 0 | 1 |
| BNIP2 | 1 | 0 | 0 | 0 | 0 | 0 | 0 | 0 | 1 |
| GALNTL4 | 1 | 0 | 0 | 0 | 0 | 0 | 0 | 0 | 1 |
| SLC34A1 | 0 | 1 | 0 | 0 | 0 | 0 | 0 | 0 | 1 |
| DPF1 | 1 | 0 | 0 | 0 | 0 | 0 | 0 | 0 | 1 |
| NKX6-1 | 1 | 0 | 0 | 0 | 0 | 0 | 0 | 0 | 1 |
| TBK1 | 1 | 0 | 0 | 0 | 0 | 0 | 0 | 0 | 1 |
| INTS10 | 0 | 0 | 0 | 0 | 1 | 0 | 0 | 0 | 1 |
| GALNT2 | 1 | 0 | 0 | 0 | 0 | 0 | 0 | 0 | 1 |
| ZNF398 | 1 | 0 | 0 | 0 | 0 | 0 | 0 | 0 | 1 |
| ELF2 | 1 | 0 | 0 | 0 | 0 | 0 | 0 | 0 | 1 |
| CCDC9 | 1 | 0 | 0 | 0 | 0 | 0 | 0 | 0 | 1 |
| MEX3C | 1 | 0 | 0 | 0 | 0 | 0 | 0 | 0 | 1 |
| HAO2 | 1 | 0 | 0 | 0 | 0 | 0 | 0 | 0 | 1 |
| KIAA0753 | 1 | 0 | 0 | 0 | 0 | 0 | 0 | 0 | 1 |
| TAAR2 | 1 | 0 | 0 | 0 | 0 | 0 | 0 | 0 | 1 |
| CHN1 | 1 | 0 | 0 | 0 | 0 | 0 | 0 | 0 | 1 |
| BSCL2 | 1 | 0 | 0 | 0 | 0 | 0 | 0 | 0 | 1 |

| | | | | | | | | | |
|---|---|---|---|---|---|---|---|---|---|
| TNFRSF25 | 1 | 0 | 0 | 0 | 0 | 0 | 0 | 0 | 1 |
| SF3A3 | 1 | 0 | 0 | 0 | 0 | 0 | 0 | 0 | 1 |
| GALNTL1 | 1 | 0 | 0 | 0 | 0 | 0 | 0 | 0 | 1 |
| NUDC | 1 | 0 | 0 | 0 | 0 | 0 | 0 | 0 | 1 |
| ZBTB22 | 1 | 0 | 0 | 0 | 0 | 0 | 0 | 0 | 1 |
| THRA | 1 | 0 | 0 | 0 | 0 | 0 | 0 | 0 | 1 |
| ADH4 | 1 | 0 | 0 | 0 | 0 | 0 | 0 | 0 | 1 |
| PPAN | 1 | 0 | 0 | 0 | 0 | 0 | 0 | 0 | 1 |
| MEPCE | 1 | 0 | 0 | 0 | 0 | 0 | 0 | 0 | 1 |
| NUAK1 | 1 | 0 | 0 | 0 | 0 | 0 | 0 | 0 | 1 |
| TMEM161B | 1 | 0 | 0 | 0 | 0 | 0 | 0 | 0 | 1 |
| MTHFSD | 1 | 0 | 0 | 0 | 0 | 0 | 0 | 0 | 1 |
| ZNF677 | 1 | 0 | 0 | 0 | 0 | 0 | 0 | 0 | 1 |
| PPFIBP2 | 0 | 1 | 0 | 0 | 0 | 0 | 0 | 0 | 1 |
| SEPSECS | 1 | 0 | 0 | 0 | 0 | 0 | 0 | 0 | 1 |
| TAB1 | 1 | 0 | 0 | 0 | 0 | 0 | 0 | 0 | 1 |
| EDA | 1 | 0 | 0 | 0 | 0 | 0 | 0 | 0 | 1 |
| SDAD1 | 1 | 0 | 0 | 0 | 0 | 0 | 0 | 0 | 1 |
| FADS1 | 1 | 0 | 0 | 0 | 0 | 0 | 0 | 0 | 1 |
| MRPL43 | 1 | 0 | 0 | 0 | 0 | 0 | 0 | 0 | 1 |
| RFFL | 1 | 0 | 0 | 0 | 0 | 0 | 0 | 0 | 1 |
| HFE | 1 | 0 | 0 | 0 | 0 | 0 | 0 | 0 | 1 |
| FAM129B | 1 | 0 | 0 | 0 | 0 | 0 | 0 | 0 | 1 |
| RBM45 | 1 | 0 | 0 | 0 | 0 | 0 | 0 | 0 | 1 |
| FAM20A | 1 | 0 | 0 | 0 | 0 | 0 | 0 | 0 | 1 |
| CDKL2 | 1 | 0 | 0 | 0 | 0 | 0 | 0 | 0 | 1 |
| MAPK10 | 1 | 0 | 0 | 0 | 0 | 0 | 0 | 0 | 1 |
| ENO2 | 1 | 0 | 0 | 0 | 0 | 0 | 0 | 0 | 1 |
| SERPING1 | 1 | 0 | 0 | 0 | 0 | 0 | 0 | 0 | 1 |
| RBBP5 | 1 | 0 | 0 | 0 | 0 | 0 | 0 | 0 | 1 |
| HSH2D | 1 | 0 | 0 | 0 | 0 | 0 | 0 | 0 | 1 |
| MIIP | 1 | 0 | 0 | 0 | 0 | 0 | 0 | 0 | 1 |
| SLC16A8 | 1 | 0 | 0 | 0 | 0 | 0 | 0 | 0 | 1 |
| NR2F2 | 1 | 0 | 0 | 0 | 0 | 0 | 0 | 0 | 1 |
| SWAP70 | 1 | 0 | 0 | 0 | 0 | 0 | 0 | 0 | 1 |
| UNKL | 1 | 0 | 0 | 0 | 0 | 0 | 0 | 0 | 1 |
| STK3 | 1 | 0 | 0 | 0 | 0 | 0 | 0 | 0 | 1 |
| UBXN7 | 1 | 0 | 0 | 0 | 0 | 0 | 0 | 0 | 1 |
| RAD51AP1 | 1 | 0 | 0 | 0 | 0 | 0 | 0 | 0 | 1 |
| ZNF678 | 1 | 0 | 0 | 0 | 0 | 0 | 0 | 0 | 1 |
| PRR22 | 1 | 0 | 0 | 0 | 0 | 0 | 0 | 0 | 1 |
| BLNK | 1 | 0 | 0 | 0 | 0 | 0 | 0 | 0 | 1 |
| TRIP10 | 1 | 0 | 0 | 0 | 0 | 0 | 0 | 0 | 1 |
| MBD4 | 1 | 0 | 0 | 0 | 0 | 0 | 0 | 0 | 1 |
| DNAJC16 | 1 | 0 | 0 | 0 | 0 | 0 | 0 | 0 | 1 |
| GABRB3 | 1 | 0 | 0 | 0 | 0 | 0 | 0 | 0 | 1 |

| | | | | | | | | | |
|---|---|---|---|---|---|---|---|---|---|
| LTA4H | 1 | 0 | 0 | 0 | 0 | 0 | 0 | 0 | 1 |
| SESN1 | 1 | 0 | 0 | 0 | 0 | 0 | 0 | 0 | 1 |
| TTC39A | 1 | 0 | 0 | 0 | 0 | 0 | 0 | 0 | 1 |
| TCP11L2 | 1 | 0 | 0 | 0 | 0 | 0 | 0 | 0 | 1 |
| SMYD3 | 1 | 0 | 0 | 0 | 0 | 0 | 0 | 0 | 1 |
| C9orf86 | 0 | 1 | 0 | 0 | 0 | 0 | 0 | 0 | 1 |
| GAB2 | 1 | 0 | 0 | 0 | 0 | 0 | 0 | 0 | 1 |
| SCARA5 | 1 | 0 | 0 | 0 | 0 | 0 | 0 | 0 | 1 |
| BLK | 1 | 0 | 0 | 0 | 0 | 0 | 0 | 0 | 1 |
| XPNPEP2 | 1 | 0 | 0 | 0 | 0 | 0 | 0 | 0 | 1 |
| SLAIN2 | 1 | 0 | 0 | 0 | 0 | 0 | 0 | 0 | 1 |
| FOXI3 | 1 | 0 | 0 | 0 | 0 | 0 | 0 | 0 | 1 |
| FOXP1 | 1 | 0 | 0 | 0 | 0 | 0 | 0 | 0 | 1 |
| RBPJ | 1 | 0 | 0 | 0 | 0 | 0 | 0 | 0 | 1 |
| EGR2 | 1 | 0 | 0 | 0 | 0 | 0 | 0 | 0 | 1 |
| SERPIND1 | 1 | 0 | 0 | 0 | 0 | 0 | 0 | 0 | 1 |
| MAGEC2 | 1 | 0 | 0 | 0 | 0 | 0 | 0 | 0 | 1 |
| KRT25 | 1 | 0 | 0 | 0 | 0 | 0 | 0 | 0 | 1 |
| LRRC32 | 1 | 0 | 0 | 0 | 0 | 0 | 0 | 0 | 1 |
| IKZF2 | 1 | 0 | 0 | 0 | 0 | 0 | 0 | 0 | 1 |
| LFNG | 1 | 0 | 0 | 0 | 0 | 0 | 0 | 0 | 1 |
| DOK3 | 1 | 0 | 0 | 0 | 0 | 0 | 0 | 0 | 1 |
| D2HGDH | 1 | 0 | 0 | 0 | 0 | 0 | 0 | 0 | 1 |
| ARHGAP15 | 1 | 0 | 0 | 0 | 0 | 0 | 0 | 0 | 1 |
| OSBPL11 | 1 | 0 | 0 | 0 | 0 | 0 | 0 | 0 | 1 |
| UPP2 | 1 | 0 | 0 | 0 | 0 | 0 | 0 | 0 | 1 |
| TDO2 | 1 | 0 | 0 | 0 | 0 | 0 | 0 | 0 | 1 |
| PXN | 1 | 0 | 0 | 0 | 0 | 0 | 0 | 0 | 1 |
| FOXF2 | 1 | 0 | 0 | 0 | 0 | 0 | 0 | 0 | 1 |
| SMAP2 | 1 | 0 | 0 | 0 | 0 | 0 | 0 | 0 | 1 |
| STAT2 | 1 | 0 | 0 | 0 | 0 | 0 | 0 | 0 | 1 |
| TRIM47 | 1 | 0 | 0 | 0 | 0 | 0 | 0 | 0 | 1 |
| IRX6 | 1 | 0 | 0 | 0 | 0 | 0 | 0 | 0 | 1 |
| SERAC1 | 1 | 0 | 0 | 0 | 0 | 0 | 0 | 0 | 1 |
| ASPHD1 | 1 | 0 | 0 | 0 | 0 | 0 | 0 | 0 | 1 |
| PSD2 | 1 | 0 | 0 | 0 | 0 | 0 | 0 | 0 | 1 |
| SLC34A3 | 1 | 0 | 0 | 0 | 0 | 0 | 0 | 0 | 1 |
| DDX50 | 1 | 0 | 0 | 0 | 0 | 0 | 0 | 0 | 1 |
| KLHL36 | 1 | 0 | 0 | 0 | 0 | 0 | 0 | 0 | 1 |
| PDLIM5 | 1 | 0 | 0 | 0 | 0 | 0 | 0 | 0 | 1 |
| AGXT2L2 | 1 | 0 | 0 | 0 | 0 | 0 | 0 | 0 | 1 |
| ANGPT4 | 1 | 0 | 0 | 0 | 0 | 0 | 0 | 0 | 1 |
| MARK4 | 1 | 0 | 0 | 0 | 0 | 0 | 0 | 0 | 1 |
| SHISA9 | 1 | 0 | 0 | 0 | 0 | 0 | 0 | 0 | 1 |
| DNAJC21 | 1 | 0 | 0 | 0 | 0 | 0 | 0 | 0 | 1 |
| SH2B2 | 1 | 0 | 0 | 0 | 0 | 0 | 0 | 0 | 1 |

| | | | | | | | | | |
|---|---|---|---|---|---|---|---|---|---|
| MFI2 | 1 | 0 | 0 | 0 | 0 | 0 | 0 | 0 | 1 |
| BCCIP | 1 | 0 | 0 | 0 | 0 | 0 | 0 | 0 | 1 |
| CA6 | 1 | 0 | 0 | 0 | 0 | 0 | 0 | 0 | 1 |
| POR | 1 | 0 | 0 | 0 | 0 | 0 | 0 | 0 | 1 |
| PPP3CA | 1 | 0 | 0 | 0 | 0 | 0 | 0 | 0 | 1 |
| FTMT | 1 | 0 | 0 | 0 | 0 | 0 | 0 | 0 | 1 |
| SGMS1 | 1 | 0 | 0 | 0 | 0 | 0 | 0 | 0 | 1 |
| PTX4 | 1 | 0 | 0 | 0 | 0 | 0 | 0 | 0 | 1 |
| TNIP2 | 1 | 0 | 0 | 0 | 0 | 0 | 0 | 0 | 1 |
| ZSCAN22 | 1 | 0 | 0 | 0 | 0 | 0 | 0 | 0 | 1 |
| EIF2B3 | 1 | 0 | 0 | 0 | 0 | 0 | 0 | 0 | 1 |
| ETV6 | 1 | 0 | 0 | 0 | 0 | 0 | 0 | 0 | 1 |
| LCMT2 | 1 | 0 | 0 | 0 | 0 | 0 | 0 | 0 | 1 |
| GYG2 | 1 | 0 | 0 | 0 | 0 | 0 | 0 | 0 | 1 |
| SELO | 1 | 0 | 0 | 0 | 0 | 0 | 0 | 0 | 1 |
| HGSNAT | 1 | 0 | 0 | 0 | 0 | 0 | 0 | 0 | 1 |
| SKA3 | 1 | 0 | 0 | 0 | 0 | 0 | 0 | 0 | 1 |
| PEX26 | 1 | 0 | 0 | 0 | 0 | 0 | 0 | 0 | 1 |
| ATP2A2 | 0 | 1 | 0 | 0 | 0 | 0 | 0 | 0 | 1 |
| E2F2 | 1 | 0 | 0 | 0 | 0 | 0 | 0 | 0 | 1 |
| POLR3D | 1 | 0 | 0 | 0 | 0 | 0 | 0 | 0 | 1 |
| C1orf106 | 1 | 0 | 0 | 0 | 0 | 0 | 0 | 0 | 1 |
| C22orf28 | 1 | 0 | 0 | 0 | 0 | 0 | 0 | 0 | 1 |
| ZNF570 | 1 | 0 | 0 | 0 | 0 | 0 | 0 | 0 | 1 |
| TAF1A | 1 | 0 | 0 | 0 | 0 | 0 | 0 | 0 | 1 |
| MMP17 | 1 | 0 | 0 | 0 | 0 | 0 | 0 | 0 | 1 |
| C1R | 1 | 0 | 0 | 0 | 0 | 0 | 0 | 0 | 1 |
| OR8B12 | 1 | 0 | 0 | 0 | 0 | 0 | 0 | 0 | 1 |
| SPAG8 | 1 | 0 | 0 | 0 | 0 | 0 | 0 | 0 | 1 |
| TMCC2 | 1 | 0 | 0 | 0 | 0 | 0 | 0 | 0 | 1 |
| ZNF732 | 1 | 0 | 0 | 0 | 0 | 0 | 0 | 0 | 1 |
| PLRG1 | 1 | 0 | 0 | 0 | 0 | 0 | 0 | 0 | 1 |
| MEIS2 | 1 | 0 | 0 | 0 | 0 | 0 | 0 | 0 | 1 |
| SEMA3B | 1 | 0 | 0 | 0 | 0 | 0 | 0 | 0 | 1 |
| ACVR2A | 1 | 0 | 0 | 0 | 0 | 0 | 0 | 0 | 1 |
| HOXB5 | 1 | 0 | 0 | 0 | 0 | 0 | 0 | 0 | 1 |
| DYRK2 | 1 | 0 | 0 | 0 | 0 | 0 | 0 | 0 | 1 |
| KPNA2 | 1 | 0 | 0 | 0 | 0 | 0 | 0 | 0 | 1 |
| SMTN | 1 | 0 | 0 | 0 | 0 | 0 | 0 | 0 | 1 |
| CSF1 | 1 | 0 | 0 | 0 | 0 | 0 | 0 | 0 | 1 |
| FAM71E1 | 1 | 0 | 0 | 0 | 0 | 0 | 0 | 0 | 1 |
| UPK1B | 1 | 0 | 0 | 0 | 0 | 0 | 0 | 0 | 1 |
| MLLT1 | 1 | 0 | 0 | 0 | 0 | 0 | 0 | 0 | 1 |
| SGK2 | 1 | 0 | 0 | 0 | 0 | 0 | 0 | 0 | 1 |
| OR2AE1 | 1 | 0 | 0 | 0 | 0 | 0 | 0 | 0 | 1 |
| NAP1L2 | 1 | 0 | 0 | 0 | 0 | 0 | 0 | 0 | 1 |

| | | | | | | | | | |
|---|---|---|---|---|---|---|---|---|---|
| EXTL1 | 1 | 0 | 0 | 0 | 0 | 0 | 0 | 0 | 1 |
| PRR25 | 1 | 0 | 0 | 0 | 0 | 0 | 0 | 0 | 1 |
| OR2T8 | 1 | 0 | 0 | 0 | 0 | 0 | 0 | 0 | 1 |
| TC2N | 1 | 0 | 0 | 0 | 0 | 0 | 0 | 0 | 1 |
| AGFG1 | 1 | 0 | 0 | 0 | 0 | 0 | 0 | 0 | 1 |
| KIF19 | 1 | 0 | 0 | 0 | 0 | 0 | 0 | 0 | 1 |
| CCDC7 | 1 | 0 | 0 | 0 | 0 | 0 | 0 | 0 | 1 |
| FOXA1 | 1 | 0 | 0 | 0 | 0 | 0 | 0 | 0 | 1 |
| KRT84 | 1 | 0 | 0 | 0 | 0 | 0 | 0 | 0 | 1 |
| DDX41 | 1 | 0 | 0 | 0 | 0 | 0 | 0 | 0 | 1 |
| CAPZA1 | 1 | 0 | 0 | 0 | 0 | 0 | 0 | 0 | 1 |
| CHIT1 | 1 | 0 | 0 | 0 | 0 | 0 | 0 | 0 | 1 |
| THAP4 | 1 | 0 | 0 | 0 | 0 | 0 | 0 | 0 | 1 |
| PARP12 | 1 | 0 | 0 | 0 | 0 | 0 | 0 | 0 | 1 |
| FBXO34 | 1 | 0 | 0 | 0 | 0 | 0 | 0 | 0 | 1 |
| MPHOSPH8 | 1 | 0 | 0 | 0 | 0 | 0 | 0 | 0 | 1 |
| RABEP1 | 1 | 0 | 0 | 0 | 0 | 0 | 0 | 0 | 1 |
| MAN1A2 | 1 | 0 | 0 | 0 | 0 | 0 | 0 | 0 | 1 |
| ENTPD3 | 1 | 0 | 0 | 0 | 0 | 0 | 0 | 0 | 1 |
| GPR50 | 1 | 0 | 0 | 0 | 0 | 0 | 0 | 0 | 1 |
| MCOLN1 | 1 | 0 | 0 | 0 | 0 | 0 | 0 | 0 | 1 |
| COQ6 | 1 | 0 | 0 | 0 | 0 | 0 | 0 | 0 | 1 |
| SKAP1 | 1 | 0 | 0 | 0 | 0 | 0 | 0 | 0 | 1 |
| C17orf74 | 1 | 0 | 0 | 0 | 0 | 0 | 0 | 0 | 1 |
| ZNF92 | 1 | 0 | 0 | 0 | 0 | 0 | 0 | 0 | 1 |
| TIGD2 | 1 | 0 | 0 | 0 | 0 | 0 | 0 | 0 | 1 |
| POLR3E | 1 | 0 | 0 | 0 | 0 | 0 | 0 | 0 | 1 |
| PADI1 | 1 | 0 | 0 | 0 | 0 | 0 | 0 | 0 | 1 |
| SIGLEC11 | 1 | 0 | 0 | 0 | 0 | 0 | 0 | 0 | 1 |
| SHC3 | 1 | 0 | 0 | 0 | 0 | 0 | 0 | 0 | 1 |
| IFT57 | 1 | 0 | 0 | 0 | 0 | 0 | 0 | 0 | 1 |
| GFRA2 | 1 | 0 | 0 | 0 | 0 | 0 | 0 | 0 | 1 |
| SPAST | 1 | 0 | 0 | 0 | 0 | 0 | 0 | 0 | 1 |
| ZNF155 | 1 | 0 | 0 | 0 | 0 | 0 | 0 | 0 | 1 |
| ACADSB | 1 | 0 | 0 | 0 | 0 | 0 | 0 | 0 | 1 |
| BRAP | 1 | 0 | 0 | 0 | 0 | 0 | 0 | 0 | 1 |
| AP1G1 | 1 | 0 | 0 | 0 | 0 | 0 | 0 | 0 | 1 |
| CKAP4 | 1 | 0 | 0 | 0 | 0 | 0 | 0 | 0 | 1 |
| WARS | 1 | 0 | 0 | 0 | 0 | 0 | 0 | 0 | 1 |
| ANXA2 | 1 | 0 | 0 | 0 | 0 | 0 | 0 | 0 | 1 |
| ARG2 | 1 | 0 | 0 | 0 | 0 | 0 | 0 | 0 | 1 |
| ALDH3A2 | 1 | 0 | 0 | 0 | 0 | 0 | 0 | 0 | 1 |
| CCNF | 1 | 0 | 0 | 0 | 0 | 0 | 0 | 0 | 1 |
| TCFL5 | 1 | 0 | 0 | 0 | 0 | 0 | 0 | 0 | 1 |
| SGOL2 | 1 | 0 | 0 | 0 | 0 | 0 | 0 | 0 | 1 |
| CERK | 1 | 0 | 0 | 0 | 0 | 0 | 0 | 0 | 1 |

| | | | | | | | | | |
|---|---|---|---|---|---|---|---|---|---|
| ADAMTS17 | 0 | 1 | 0 | 0 | 0 | 0 | 0 | 0 | 1 |
| MPP1 | 1 | 0 | 0 | 0 | 0 | 0 | 0 | 0 | 1 |
| C1orf114 | 1 | 0 | 0 | 0 | 0 | 0 | 0 | 0 | 1 |
| TBL1XR1 | 1 | 0 | 0 | 0 | 0 | 0 | 0 | 0 | 1 |
| PDPK1 | 1 | 0 | 0 | 0 | 0 | 0 | 0 | 0 | 1 |
| APEX2 | 1 | 0 | 0 | 0 | 0 | 0 | 0 | 0 | 1 |
| ALDH6A1 | 1 | 0 | 0 | 0 | 0 | 0 | 0 | 0 | 1 |
| RAB11FIP5 | 1 | 0 | 0 | 0 | 0 | 0 | 0 | 0 | 1 |
| GLT25D2 | 1 | 0 | 0 | 0 | 0 | 0 | 0 | 0 | 1 |
| KRT36 | 1 | 0 | 0 | 0 | 0 | 0 | 0 | 0 | 1 |
| HEXA | 1 | 0 | 0 | 0 | 0 | 0 | 0 | 0 | 1 |
| SLC26A10 | 1 | 0 | 0 | 0 | 0 | 0 | 0 | 0 | 1 |
| NMT2 | 1 | 0 | 0 | 0 | 0 | 0 | 0 | 0 | 1 |
| MIER3 | 1 | 0 | 0 | 0 | 0 | 0 | 0 | 0 | 1 |
| ABHD13 | 1 | 0 | 0 | 0 | 0 | 0 | 0 | 0 | 1 |
| PPIL4 | 1 | 0 | 0 | 0 | 0 | 0 | 0 | 0 | 1 |
| ADAMTSL5 | 1 | 0 | 0 | 0 | 0 | 0 | 0 | 0 | 1 |
| CHMP7 | 1 | 0 | 0 | 0 | 0 | 0 | 0 | 0 | 1 |
| CLPB | 1 | 0 | 0 | 0 | 0 | 0 | 0 | 0 | 1 |
| KRT33A | 1 | 0 | 0 | 0 | 0 | 0 | 0 | 0 | 1 |
| MRPS27 | 1 | 0 | 0 | 0 | 0 | 0 | 0 | 0 | 1 |
| MAOA | 1 | 0 | 0 | 0 | 0 | 0 | 0 | 0 | 1 |
| METTL14 | 1 | 0 | 0 | 0 | 0 | 0 | 0 | 0 | 1 |
| CIRH1A | 1 | 0 | 0 | 0 | 0 | 0 | 0 | 0 | 1 |
| NUP85 | 1 | 0 | 0 | 0 | 0 | 0 | 0 | 0 | 1 |
| RAD23B | 1 | 0 | 0 | 0 | 0 | 0 | 0 | 0 | 1 |
| LENG1 | 1 | 0 | 0 | 0 | 0 | 0 | 0 | 0 | 1 |
| FZD5 | 1 | 0 | 0 | 0 | 0 | 0 | 0 | 0 | 1 |
| OR10Z1 | 1 | 0 | 0 | 0 | 0 | 0 | 0 | 0 | 1 |
| ZNF12 | 1 | 0 | 0 | 0 | 0 | 0 | 0 | 0 | 1 |
| VAV1 | 1 | 0 | 0 | 0 | 0 | 0 | 0 | 0 | 1 |
| ZNF454 | 1 | 0 | 0 | 0 | 0 | 0 | 0 | 0 | 1 |
| THNSL2 | 1 | 0 | 0 | 0 | 0 | 0 | 0 | 0 | 1 |
| TKT | 1 | 0 | 0 | 0 | 0 | 0 | 0 | 0 | 1 |
| ONECUT1 | 1 | 0 | 0 | 0 | 0 | 0 | 0 | 0 | 1 |
| RHPN1 | 1 | 0 | 0 | 0 | 0 | 0 | 0 | 0 | 1 |
| CYP2E1 | 1 | 0 | 0 | 0 | 0 | 0 | 0 | 0 | 1 |
| SLFN5 | 0 | 1 | 0 | 0 | 0 | 0 | 0 | 0 | 1 |
| SERPINI1 | 1 | 0 | 0 | 0 | 0 | 0 | 0 | 0 | 1 |
| GPCPD1 | 1 | 0 | 0 | 0 | 0 | 0 | 0 | 0 | 1 |
| MAVS | 1 | 0 | 0 | 0 | 0 | 0 | 0 | 0 | 1 |
| TECRL | 1 | 0 | 0 | 0 | 0 | 0 | 0 | 0 | 1 |
| DPYSL4 | 1 | 0 | 0 | 0 | 0 | 0 | 0 | 0 | 1 |
| UTP14C | 1 | 0 | 0 | 0 | 0 | 0 | 0 | 0 | 1 |
| TBC1D19 | 1 | 0 | 0 | 0 | 0 | 0 | 0 | 0 | 1 |
| SEMA4B | 1 | 0 | 0 | 0 | 0 | 0 | 0 | 0 | 1 |

| | | | | | | | | | |
|---|---|---|---|---|---|---|---|---|---|
| NFKBID | 1 | 0 | 0 | 0 | 0 | 0 | 0 | 0 | 1 |
| NPNT | 1 | 0 | 0 | 0 | 0 | 0 | 0 | 0 | 1 |
| GPSM2 | 1 | 0 | 0 | 0 | 0 | 0 | 0 | 0 | 1 |
| ESR2 | 1 | 0 | 0 | 0 | 0 | 0 | 0 | 0 | 1 |
| GCOM1 | 1 | 0 | 0 | 0 | 0 | 0 | 0 | 0 | 1 |
| ZNF45 | 1 | 0 | 0 | 0 | 0 | 0 | 0 | 0 | 1 |
| NOX1 | 1 | 0 | 0 | 0 | 0 | 0 | 0 | 0 | 1 |
| ZAP70 | 1 | 0 | 0 | 0 | 0 | 0 | 0 | 0 | 1 |
| CDCA7L | 1 | 0 | 0 | 0 | 0 | 0 | 0 | 0 | 1 |
| IK | 1 | 0 | 0 | 0 | 0 | 0 | 0 | 0 | 1 |
| MYADM | 1 | 0 | 0 | 0 | 0 | 0 | 0 | 0 | 1 |
| CCDC114 | 1 | 0 | 0 | 0 | 0 | 0 | 0 | 0 | 1 |
| ESRRB | 1 | 0 | 0 | 0 | 0 | 0 | 0 | 0 | 1 |
| C9orf72 | 1 | 0 | 0 | 0 | 0 | 0 | 0 | 0 | 1 |
| ABHD12 | 1 | 0 | 0 | 0 | 0 | 0 | 0 | 0 | 1 |
| ARHGEF37 | 1 | 0 | 0 | 0 | 0 | 0 | 0 | 0 | 1 |
| KRT73 | 1 | 0 | 0 | 0 | 0 | 0 | 0 | 0 | 1 |
| LMBRD2 | 1 | 0 | 0 | 0 | 0 | 0 | 0 | 0 | 1 |
| USP16 | 1 | 0 | 0 | 0 | 0 | 0 | 0 | 0 | 1 |
| FNDC7 | 1 | 0 | 0 | 0 | 0 | 0 | 0 | 0 | 1 |
| KCNG4 | 1 | 0 | 0 | 0 | 0 | 0 | 0 | 0 | 1 |
| SLC38A9 | 1 | 0 | 0 | 0 | 0 | 0 | 0 | 0 | 1 |
| TGFB2 | 1 | 0 | 0 | 0 | 0 | 0 | 0 | 0 | 1 |
| LIPC | 1 | 0 | 0 | 0 | 0 | 0 | 0 | 0 | 1 |
| PIK3R2 | 1 | 0 | 0 | 0 | 0 | 0 | 0 | 0 | 1 |
| KRT79 | 1 | 0 | 0 | 0 | 0 | 0 | 0 | 0 | 1 |
| PAK4 | 1 | 0 | 0 | 0 | 0 | 0 | 0 | 0 | 1 |
| ZPBP | 1 | 0 | 0 | 0 | 0 | 0 | 0 | 0 | 1 |
| MPP3 | 1 | 0 | 0 | 0 | 0 | 0 | 0 | 0 | 1 |
| CHRNA9 | 1 | 0 | 0 | 0 | 0 | 0 | 0 | 0 | 1 |
| FAM65A | 1 | 0 | 0 | 0 | 0 | 0 | 0 | 0 | 1 |
| HMGCS2 | 1 | 0 | 0 | 0 | 0 | 0 | 0 | 0 | 1 |
| EML2 | 1 | 0 | 0 | 0 | 0 | 0 | 0 | 0 | 1 |
| LZTS1 | 1 | 0 | 0 | 0 | 0 | 0 | 0 | 0 | 1 |
| FAM55A | 1 | 0 | 0 | 0 | 0 | 0 | 0 | 0 | 1 |
| TMPRSS13 | 1 | 0 | 0 | 0 | 0 | 0 | 0 | 0 | 1 |
| PVRL2 | 1 | 0 | 0 | 0 | 0 | 0 | 0 | 0 | 1 |
| GDPD2 | 1 | 0 | 0 | 0 | 0 | 0 | 0 | 0 | 1 |
| ITFG2 | 1 | 0 | 0 | 0 | 0 | 0 | 0 | 0 | 1 |
| PTER | 1 | 0 | 0 | 0 | 0 | 0 | 0 | 0 | 1 |
| SS18 | 1 | 0 | 0 | 0 | 0 | 0 | 0 | 0 | 1 |
| MTMR6 | 1 | 0 | 0 | 0 | 0 | 0 | 0 | 0 | 1 |
| RTKN2 | 1 | 0 | 0 | 0 | 0 | 0 | 0 | 0 | 1 |
| HINFP | 1 | 0 | 0 | 0 | 0 | 0 | 0 | 0 | 1 |
| TBX21 | 1 | 0 | 0 | 0 | 0 | 0 | 0 | 0 | 1 |
| FUT8 | 1 | 0 | 0 | 0 | 0 | 0 | 0 | 0 | 1 |

| | | | | | | | | | |
|---|---|---|---|---|---|---|---|---|---|
| SF3B4 | 1 | 0 | 0 | 0 | 0 | 0 | 0 | 0 | 1 |
| ME3 | 1 | 0 | 0 | 0 | 0 | 0 | 0 | 0 | 1 |
| LANCL2 | 1 | 0 | 0 | 0 | 0 | 0 | 0 | 0 | 1 |
| KRT1 | 1 | 0 | 0 | 0 | 0 | 0 | 0 | 0 | 1 |
| ALDH5A1 | 1 | 0 | 0 | 0 | 0 | 0 | 0 | 0 | 1 |
| GRAMD4 | 1 | 0 | 0 | 0 | 0 | 0 | 0 | 0 | 1 |
| UTP3 | 1 | 0 | 0 | 0 | 0 | 0 | 0 | 0 | 1 |
| OR8I2 | 1 | 0 | 0 | 0 | 0 | 0 | 0 | 0 | 1 |
| LATS1 | 0 | 1 | 0 | 0 | 0 | 0 | 0 | 0 | 1 |
| IPMK | 1 | 0 | 0 | 0 | 0 | 0 | 0 | 0 | 1 |
| MICALCL | 1 | 0 | 0 | 0 | 0 | 0 | 0 | 0 | 1 |
| PLXDC1 | 1 | 0 | 0 | 0 | 0 | 0 | 0 | 0 | 1 |
| FSD1L | 1 | 0 | 0 | 0 | 0 | 0 | 0 | 0 | 1 |
| ZNF320 | 1 | 0 | 0 | 0 | 0 | 0 | 0 | 0 | 1 |
| ARPM1 | 1 | 0 | 0 | 0 | 0 | 0 | 0 | 0 | 1 |
| C6orf136 | 1 | 0 | 0 | 0 | 0 | 0 | 0 | 0 | 1 |
| FBXO4 | 1 | 0 | 0 | 0 | 0 | 0 | 0 | 0 | 1 |
| ARHGAP36 | 1 | 0 | 0 | 0 | 0 | 0 | 0 | 0 | 1 |
| ZBTB3 | 1 | 0 | 0 | 0 | 0 | 0 | 0 | 0 | 1 |
| TUBGCP2 | 1 | 0 | 0 | 0 | 0 | 0 | 0 | 0 | 1 |
| ANAPC7 | 1 | 0 | 0 | 0 | 0 | 0 | 0 | 0 | 1 |
| FANCG | 1 | 0 | 0 | 0 | 0 | 0 | 0 | 0 | 1 |
| CCT8 | 1 | 0 | 0 | 0 | 0 | 0 | 0 | 0 | 1 |
| THOP1 | 1 | 0 | 0 | 0 | 0 | 0 | 0 | 0 | 1 |
| PYGL | 1 | 0 | 0 | 0 | 0 | 0 | 0 | 0 | 1 |
| GNPAT | 1 | 0 | 0 | 0 | 0 | 0 | 0 | 0 | 1 |
| VANGL1 | 1 | 0 | 0 | 0 | 0 | 0 | 0 | 0 | 1 |
| SAMD14 | 1 | 0 | 0 | 0 | 0 | 0 | 0 | 0 | 1 |
| SAMM50 | 1 | 0 | 0 | 0 | 0 | 0 | 0 | 0 | 1 |
| FPGT | 1 | 0 | 0 | 0 | 0 | 0 | 0 | 0 | 1 |
| ESCO1 | 1 | 0 | 0 | 0 | 0 | 0 | 0 | 0 | 1 |
| FAM98A | 1 | 0 | 0 | 0 | 0 | 0 | 0 | 0 | 1 |
| CHRDL1 | 1 | 0 | 0 | 0 | 0 | 0 | 0 | 0 | 1 |
| DBH | 1 | 0 | 0 | 0 | 0 | 0 | 0 | 0 | 1 |
| GLB1L2 | 1 | 0 | 0 | 0 | 0 | 0 | 0 | 0 | 1 |
| IFT88 | 1 | 0 | 0 | 0 | 0 | 0 | 0 | 0 | 1 |
| PARP3 | 1 | 0 | 0 | 0 | 0 | 0 | 0 | 0 | 1 |
| AMDHD2 | 1 | 0 | 0 | 0 | 0 | 0 | 0 | 0 | 1 |
| ZBTB24 | 1 | 0 | 0 | 0 | 0 | 0 | 0 | 0 | 1 |
| ACSL3 | 1 | 0 | 0 | 0 | 0 | 0 | 0 | 0 | 1 |
| CCDC71 | 1 | 0 | 0 | 0 | 0 | 0 | 0 | 0 | 1 |
| ILVBL | 1 | 0 | 0 | 0 | 0 | 0 | 0 | 0 | 1 |
| IQCC | 1 | 0 | 0 | 0 | 0 | 0 | 0 | 0 | 1 |
| SLC30A5 | 1 | 0 | 0 | 0 | 0 | 0 | 0 | 0 | 1 |
| ADAT1 | 1 | 0 | 0 | 0 | 0 | 0 | 0 | 0 | 1 |
| CASP1 | 1 | 0 | 0 | 0 | 0 | 0 | 0 | 0 | 1 |

| | | | | | | | | | |
|---|---|---|---|---|---|---|---|---|---|
| L3MBTL2 | 1 | 0 | 0 | 0 | 0 | 0 | 0 | 0 | 1 |
| SH3GL1 | 1 | 0 | 0 | 0 | 0 | 0 | 0 | 0 | 1 |
| TRPM5 | 1 | 0 | 0 | 0 | 0 | 0 | 0 | 0 | 1 |
| CD44 | 1 | 0 | 0 | 0 | 0 | 0 | 0 | 0 | 1 |
| OR56A3 | 1 | 0 | 0 | 0 | 0 | 0 | 0 | 0 | 1 |
| B4GALT4 | 1 | 0 | 0 | 0 | 0 | 0 | 0 | 0 | 1 |
| OR4K13 | 1 | 0 | 0 | 0 | 0 | 0 | 0 | 0 | 1 |
| SUPV3L1 | 1 | 0 | 0 | 0 | 0 | 0 | 0 | 0 | 1 |
| ZNF671 | 1 | 0 | 0 | 0 | 0 | 0 | 0 | 0 | 1 |
| DPYSL5 | 1 | 0 | 0 | 0 | 0 | 0 | 0 | 0 | 1 |
| POU5F2 | 1 | 0 | 0 | 0 | 0 | 0 | 0 | 0 | 1 |
| HOOK2 | 1 | 0 | 0 | 0 | 0 | 0 | 0 | 0 | 1 |
| MAT1A | 1 | 0 | 0 | 0 | 0 | 0 | 0 | 0 | 1 |
| CRHR1 | 1 | 0 | 0 | 0 | 0 | 0 | 0 | 0 | 1 |
| STX16 | 1 | 0 | 0 | 0 | 0 | 0 | 0 | 0 | 1 |
| SNX3 | 1 | 0 | 0 | 0 | 0 | 0 | 0 | 0 | 1 |
| PRRC1 | 1 | 0 | 0 | 0 | 0 | 0 | 0 | 0 | 1 |
| RYK | 1 | 0 | 0 | 0 | 0 | 0 | 0 | 0 | 1 |
| ZNF468 | 1 | 0 | 0 | 0 | 0 | 0 | 0 | 0 | 1 |
| CD33 | 1 | 0 | 0 | 0 | 0 | 0 | 0 | 0 | 1 |
| NEURL | 1 | 0 | 0 | 0 | 0 | 0 | 0 | 0 | 1 |
| TCHP | 1 | 0 | 0 | 0 | 0 | 0 | 0 | 0 | 1 |
| PRODH2 | 1 | 0 | 0 | 0 | 0 | 0 | 0 | 0 | 1 |
| IL18R1 | 1 | 0 | 0 | 0 | 0 | 0 | 0 | 0 | 1 |
| AKR1C3 | 1 | 0 | 0 | 0 | 0 | 0 | 0 | 0 | 1 |
| WIPF2 | 1 | 0 | 0 | 0 | 0 | 0 | 0 | 0 | 1 |
| GPR75 | 1 | 0 | 0 | 0 | 0 | 0 | 0 | 0 | 1 |
| CPE | 1 | 0 | 0 | 0 | 0 | 0 | 0 | 0 | 1 |
| TCIRG1 | 0 | 1 | 0 | 0 | 0 | 0 | 0 | 0 | 1 |
| CTAGE5 | 1 | 0 | 0 | 0 | 0 | 0 | 0 | 0 | 1 |
| KLHL7 | 1 | 0 | 0 | 0 | 0 | 0 | 0 | 0 | 1 |
| MBOAT1 | 1 | 0 | 0 | 0 | 0 | 0 | 0 | 0 | 1 |
| ZNF498 | 1 | 0 | 0 | 0 | 0 | 0 | 0 | 0 | 1 |
| HS3ST2 | 1 | 0 | 0 | 0 | 0 | 0 | 0 | 0 | 1 |
| POU2AF1 | 1 | 0 | 0 | 0 | 0 | 0 | 0 | 0 | 1 |
| LRRC8E | 1 | 0 | 0 | 0 | 0 | 0 | 0 | 0 | 1 |
| USP1 | 1 | 0 | 0 | 0 | 0 | 0 | 0 | 0 | 1 |
| GPR61 | 1 | 0 | 0 | 0 | 0 | 0 | 0 | 0 | 1 |
| VILL | 1 | 0 | 0 | 0 | 0 | 0 | 0 | 0 | 1 |
| STS | 1 | 0 | 0 | 0 | 0 | 0 | 0 | 0 | 1 |
| FICD | 1 | 0 | 0 | 0 | 0 | 0 | 0 | 0 | 1 |
| PNLIPRP3 | 1 | 0 | 0 | 0 | 0 | 0 | 0 | 0 | 1 |
| ITLN1 | 1 | 0 | 0 | 0 | 0 | 0 | 0 | 0 | 1 |
| KLHL34 | 1 | 0 | 0 | 0 | 0 | 0 | 0 | 0 | 1 |
| ZNF331 | 1 | 0 | 0 | 0 | 0 | 0 | 0 | 0 | 1 |
| ARHGAP8 | 1 | 0 | 0 | 0 | 0 | 0 | 0 | 0 | 1 |

| | | | | | | | | | |
|---|---|---|---|---|---|---|---|---|---|
| GAS6 | 1 | 0 | 0 | 0 | 0 | 0 | 0 | 0 | 1 |
| SYT13 | 1 | 0 | 0 | 0 | 0 | 0 | 0 | 0 | 1 |
| WDR70 | 1 | 0 | 0 | 0 | 0 | 0 | 0 | 0 | 1 |
| RAB3GAP1 | 0 | 0 | 1 | 0 | 0 | 0 | 0 | 0 | 1 |
| NEK6 | 1 | 0 | 0 | 0 | 0 | 0 | 0 | 0 | 1 |
| GGNBP2 | 1 | 0 | 0 | 0 | 0 | 0 | 0 | 0 | 1 |
| GATA2 | 1 | 0 | 0 | 0 | 0 | 0 | 0 | 0 | 1 |
| CPSF2 | 1 | 0 | 0 | 0 | 0 | 0 | 0 | 0 | 1 |
| EPHB4 | 1 | 0 | 0 | 0 | 0 | 0 | 0 | 0 | 1 |
| PAK2 | 1 | 0 | 0 | 0 | 0 | 0 | 0 | 0 | 1 |
| PAPD4 | 1 | 0 | 0 | 0 | 0 | 0 | 0 | 0 | 1 |
| TNPO1 | 1 | 0 | 0 | 0 | 0 | 0 | 0 | 0 | 1 |
| CTNNBL1 | 1 | 0 | 0 | 0 | 0 | 0 | 0 | 0 | 1 |
| KLHL22 | 1 | 0 | 0 | 0 | 0 | 0 | 0 | 0 | 1 |
| FDPS | 1 | 0 | 0 | 0 | 0 | 0 | 0 | 0 | 1 |
| GRSF1 | 1 | 0 | 0 | 0 | 0 | 0 | 0 | 0 | 1 |
| TFG | 1 | 0 | 0 | 0 | 0 | 0 | 0 | 0 | 1 |
| ZC3H7A | 1 | 0 | 0 | 0 | 0 | 0 | 0 | 0 | 1 |
| EXOC3 | 1 | 0 | 0 | 0 | 0 | 0 | 0 | 0 | 1 |
| SCARA3 | 1 | 0 | 0 | 0 | 0 | 0 | 0 | 0 | 1 |
| PTK2 | 0 | 1 | 0 | 0 | 0 | 0 | 0 | 0 | 1 |
| TLX3 | 1 | 0 | 0 | 0 | 0 | 0 | 0 | 0 | 1 |
| SLC24A4 | 1 | 0 | 0 | 0 | 0 | 0 | 0 | 0 | 1 |
| ANAPC2 | 1 | 0 | 0 | 0 | 0 | 0 | 0 | 0 | 1 |
| KLHL6 | 1 | 0 | 0 | 0 | 0 | 0 | 0 | 0 | 1 |
| ZBTB46 | 1 | 0 | 0 | 0 | 0 | 0 | 0 | 0 | 1 |
| KIF3C | 1 | 0 | 0 | 0 | 0 | 0 | 0 | 0 | 1 |
| PODXL2 | 1 | 0 | 0 | 0 | 0 | 0 | 0 | 0 | 1 |
| HOXB2 | 1 | 0 | 0 | 0 | 0 | 0 | 0 | 0 | 1 |
| ACADL | 1 | 0 | 0 | 0 | 0 | 0 | 0 | 0 | 1 |
| AP2B1 | 1 | 0 | 0 | 0 | 0 | 0 | 0 | 0 | 1 |
| KDELC2 | 1 | 0 | 0 | 0 | 0 | 0 | 0 | 0 | 1 |
| LONP2 | 1 | 0 | 0 | 0 | 0 | 0 | 0 | 0 | 1 |
| TRMT2A | 1 | 0 | 0 | 0 | 0 | 0 | 0 | 0 | 1 |
| PCDHB10 | 1 | 0 | 0 | 0 | 0 | 0 | 0 | 0 | 1 |
| ARHGEF19 | 1 | 0 | 0 | 0 | 0 | 0 | 0 | 0 | 1 |
| DNAI2 | 1 | 0 | 0 | 0 | 0 | 0 | 0 | 0 | 1 |
| PEX6 | 1 | 0 | 0 | 0 | 0 | 0 | 0 | 0 | 1 |
| KY | 1 | 0 | 0 | 0 | 0 | 0 | 0 | 0 | 1 |
| RASGEF1B | 1 | 0 | 0 | 0 | 0 | 0 | 0 | 0 | 1 |
| TLR8 | 1 | 0 | 0 | 0 | 0 | 0 | 0 | 0 | 1 |
| NKRF | 1 | 0 | 0 | 0 | 0 | 0 | 0 | 0 | 1 |
| ARHGAP10 | 1 | 0 | 0 | 0 | 0 | 0 | 0 | 0 | 1 |
| OXCT1 | 1 | 0 | 0 | 0 | 0 | 0 | 0 | 0 | 1 |
| SEL1L | 1 | 0 | 0 | 0 | 0 | 0 | 0 | 0 | 1 |
| ADIPOQ | 1 | 0 | 0 | 0 | 0 | 0 | 0 | 0 | 1 |

| | | | | | | | | | |
|---|---|---|---|---|---|---|---|---|---|
| TRIM33 | 1 | 0 | 0 | 0 | 0 | 0 | 0 | 0 | 1 |
| ZNF184 | 1 | 0 | 0 | 0 | 0 | 0 | 0 | 0 | 1 |
| ABCB9 | 1 | 0 | 0 | 0 | 0 | 0 | 0 | 0 | 1 |
| RELB | 1 | 0 | 0 | 0 | 0 | 0 | 0 | 0 | 1 |
| PTCHD3 | 1 | 0 | 0 | 0 | 0 | 0 | 0 | 0 | 1 |
| OR1G1 | 1 | 0 | 0 | 0 | 0 | 0 | 0 | 0 | 1 |
| CELF6 | 1 | 0 | 0 | 0 | 0 | 0 | 0 | 0 | 1 |
| GALNT3 | 1 | 0 | 0 | 0 | 0 | 0 | 0 | 0 | 1 |
| CCNL2 | 1 | 0 | 0 | 0 | 0 | 0 | 0 | 0 | 1 |
| MBNL1 | 1 | 0 | 0 | 0 | 0 | 0 | 0 | 0 | 1 |
| ARMC2 | 1 | 0 | 0 | 0 | 0 | 0 | 0 | 0 | 1 |
| GGT7 | 1 | 0 | 0 | 0 | 0 | 0 | 0 | 0 | 1 |
| ZNF71 | 1 | 0 | 0 | 0 | 0 | 0 | 0 | 0 | 1 |
| EZH2 | 1 | 0 | 0 | 0 | 0 | 0 | 0 | 0 | 1 |
| DDHD2 | 1 | 0 | 0 | 0 | 0 | 0 | 0 | 0 | 1 |
| C14orf159 | 1 | 0 | 0 | 0 | 0 | 0 | 0 | 0 | 1 |
| FAM55B | 1 | 0 | 0 | 0 | 0 | 0 | 0 | 0 | 1 |
| CCBL2 | 1 | 0 | 0 | 0 | 0 | 0 | 0 | 0 | 1 |
| SLC6A14 | 1 | 0 | 0 | 0 | 0 | 0 | 0 | 0 | 1 |
| EEPD1 | 1 | 0 | 0 | 0 | 0 | 0 | 0 | 0 | 1 |
| EMILIN3 | 1 | 0 | 0 | 0 | 0 | 0 | 0 | 0 | 1 |
| SYTL3 | 1 | 0 | 0 | 0 | 0 | 0 | 0 | 0 | 1 |
| DNMT3B | 1 | 0 | 0 | 0 | 0 | 0 | 0 | 0 | 1 |
| TPRN | 1 | 0 | 0 | 0 | 0 | 0 | 0 | 0 | 1 |
| GLCE | 1 | 0 | 0 | 0 | 0 | 0 | 0 | 0 | 1 |
| PRSS53 | 1 | 0 | 0 | 0 | 0 | 0 | 0 | 0 | 1 |
| ZYG11A | 1 | 0 | 0 | 0 | 0 | 0 | 0 | 0 | 1 |
| FOXO4 | 1 | 0 | 0 | 0 | 0 | 0 | 0 | 0 | 1 |
| KLHL30 | 1 | 0 | 0 | 0 | 0 | 0 | 0 | 0 | 1 |
| CDC14B | 1 | 0 | 0 | 0 | 0 | 0 | 0 | 0 | 1 |
| SMCR7 | 1 | 0 | 0 | 0 | 0 | 0 | 0 | 0 | 1 |
| G6PC2 | 1 | 0 | 0 | 0 | 0 | 0 | 0 | 0 | 1 |
| BDNF | 1 | 0 | 0 | 0 | 0 | 0 | 0 | 0 | 1 |
| BRD7 | 1 | 0 | 0 | 0 | 0 | 0 | 0 | 0 | 1 |
| ZFP2 | 1 | 0 | 0 | 0 | 0 | 0 | 0 | 0 | 1 |
| GPRIN2 | 1 | 0 | 0 | 0 | 0 | 0 | 0 | 0 | 1 |
| GRB14 | 1 | 0 | 0 | 0 | 0 | 0 | 0 | 0 | 1 |
| LRRC48 | 1 | 0 | 0 | 0 | 0 | 0 | 0 | 0 | 1 |
| RPA1 | 1 | 0 | 0 | 0 | 0 | 0 | 0 | 0 | 1 |
| DTX3L | 1 | 0 | 0 | 0 | 0 | 0 | 0 | 0 | 1 |
| KLHL3 | 1 | 0 | 0 | 0 | 0 | 0 | 0 | 0 | 1 |
| ANKRD44 | 1 | 0 | 0 | 0 | 0 | 0 | 0 | 0 | 1 |
| BAG3 | 1 | 0 | 0 | 0 | 0 | 0 | 0 | 0 | 1 |
| PROKR2 | 1 | 0 | 0 | 0 | 0 | 0 | 0 | 0 | 1 |
| HMGXB4 | 1 | 0 | 0 | 0 | 0 | 0 | 0 | 0 | 1 |
| PLEKHO2 | 1 | 0 | 0 | 0 | 0 | 0 | 0 | 0 | 1 |

| | | | | | | | | | |
|---|---|---|---|---|---|---|---|---|---|
| KCNJ5 | 1 | 0 | 0 | 0 | 0 | 0 | 0 | 0 | 1 |
| QRICH1 | 1 | 0 | 0 | 0 | 0 | 0 | 0 | 0 | 1 |
| GPR155 | 1 | 0 | 0 | 0 | 0 | 0 | 0 | 0 | 1 |
| CALCOCO1 | 1 | 0 | 0 | 0 | 0 | 0 | 0 | 0 | 1 |
| LMBR1 | 1 | 0 | 0 | 0 | 0 | 0 | 0 | 0 | 1 |
| CLCN7 | 1 | 0 | 0 | 0 | 0 | 0 | 0 | 0 | 1 |
| PRSS21 | 1 | 0 | 0 | 0 | 0 | 0 | 0 | 0 | 1 |
| CARD9 | 1 | 0 | 0 | 0 | 0 | 0 | 0 | 0 | 1 |
| GADL1 | 1 | 0 | 0 | 0 | 0 | 0 | 0 | 0 | 1 |
| ADSSL1 | 1 | 0 | 0 | 0 | 0 | 0 | 0 | 0 | 1 |
| FAM124A | 1 | 0 | 0 | 0 | 0 | 0 | 0 | 0 | 1 |
| KIRREL | 1 | 0 | 0 | 0 | 0 | 0 | 0 | 0 | 1 |
| LGI4 | 1 | 0 | 0 | 0 | 0 | 0 | 0 | 0 | 1 |
| ZC3H14 | 1 | 0 | 0 | 0 | 0 | 0 | 0 | 0 | 1 |
| TTC5 | 1 | 0 | 0 | 0 | 0 | 0 | 0 | 0 | 1 |
| SPATA5L1 | 1 | 0 | 0 | 0 | 0 | 0 | 0 | 0 | 1 |
| NFE2L1 | 1 | 0 | 0 | 0 | 0 | 0 | 0 | 0 | 1 |
| GRAMD1C | 1 | 0 | 0 | 0 | 0 | 0 | 0 | 0 | 1 |
| KCNF1 | 1 | 0 | 0 | 0 | 0 | 0 | 0 | 0 | 1 |
| NOL11 | 1 | 0 | 0 | 0 | 0 | 0 | 0 | 0 | 1 |
| ARL13B | 1 | 0 | 0 | 0 | 0 | 0 | 0 | 0 | 1 |
| USP6NL | 1 | 0 | 0 | 0 | 0 | 0 | 0 | 0 | 1 |
| AP2A2 | 1 | 0 | 0 | 0 | 0 | 0 | 0 | 0 | 1 |
| AMFR | 1 | 0 | 0 | 0 | 0 | 0 | 0 | 0 | 1 |
| NFIA | 1 | 0 | 0 | 0 | 0 | 0 | 0 | 0 | 1 |
| RTBDN | 0 | 1 | 0 | 0 | 0 | 0 | 0 | 0 | 1 |
| OR52I2 | 1 | 0 | 0 | 0 | 0 | 0 | 0 | 0 | 1 |
| NAB2 | 1 | 0 | 0 | 0 | 0 | 0 | 0 | 0 | 1 |
| ZBTB7B | 1 | 0 | 0 | 0 | 0 | 0 | 0 | 0 | 1 |
| CTSC | 1 | 0 | 0 | 0 | 0 | 0 | 0 | 0 | 1 |
| TOR1AIP2 | 1 | 0 | 0 | 0 | 0 | 0 | 0 | 0 | 1 |
| CAPN5 | 1 | 0 | 0 | 0 | 0 | 0 | 0 | 0 | 1 |
| C17orf80 | 1 | 0 | 0 | 0 | 0 | 0 | 0 | 0 | 1 |
| ANKRD13D | 1 | 0 | 0 | 0 | 0 | 0 | 0 | 0 | 1 |
| DKC1 | 1 | 0 | 0 | 0 | 0 | 0 | 0 | 0 | 1 |
| ZNF763 | 1 | 0 | 0 | 0 | 0 | 0 | 0 | 0 | 1 |
| CRTAC1 | 1 | 0 | 0 | 0 | 0 | 0 | 0 | 0 | 1 |
| MOGAT2 | 0 | 1 | 0 | 0 | 0 | 0 | 0 | 0 | 1 |
| SMEK2 | 1 | 0 | 0 | 0 | 0 | 0 | 0 | 0 | 1 |
| APLP2 | 1 | 0 | 0 | 0 | 0 | 0 | 0 | 0 | 1 |
| C11orf42 | 1 | 0 | 0 | 0 | 0 | 0 | 0 | 0 | 1 |
| HEATR3 | 1 | 0 | 0 | 0 | 0 | 0 | 0 | 0 | 1 |
| STAM2 | 1 | 0 | 0 | 0 | 0 | 0 | 0 | 0 | 1 |
| ANKK1 | 1 | 0 | 0 | 0 | 0 | 0 | 0 | 0 | 1 |
| DACT3 | 1 | 0 | 0 | 0 | 0 | 0 | 0 | 0 | 1 |
| ZFP30 | 1 | 0 | 0 | 0 | 0 | 0 | 0 | 0 | 1 |

| | | | | | | | | | |
|---|---|---|---|---|---|---|---|---|---|
| KCNS2 | 1 | 0 | 0 | 0 | 0 | 0 | 0 | 0 | 1 |
| KTN1 | 1 | 0 | 0 | 0 | 0 | 0 | 0 | 0 | 1 |
| FAM40A | 1 | 0 | 0 | 0 | 0 | 0 | 0 | 0 | 1 |
| TRAM1L1 | 1 | 0 | 0 | 0 | 0 | 0 | 0 | 0 | 1 |
| RASA2 | 1 | 0 | 0 | 0 | 0 | 0 | 0 | 0 | 1 |
| COLQ | 1 | 0 | 0 | 0 | 0 | 0 | 0 | 0 | 1 |
| TMEM164 | 1 | 0 | 0 | 0 | 0 | 0 | 0 | 0 | 1 |
| FSIP1 | 1 | 0 | 0 | 0 | 0 | 0 | 0 | 0 | 1 |
| ACER3 | 1 | 0 | 0 | 0 | 0 | 0 | 0 | 0 | 1 |
| ZIM3 | 1 | 0 | 0 | 0 | 0 | 0 | 0 | 0 | 1 |
| RFWD2 | 1 | 0 | 0 | 0 | 0 | 0 | 0 | 0 | 1 |
| LDHB | 1 | 0 | 0 | 0 | 0 | 0 | 0 | 0 | 1 |
| JPH1 | 1 | 0 | 0 | 0 | 0 | 0 | 0 | 0 | 1 |
| ZIC1 | 1 | 0 | 0 | 0 | 0 | 0 | 0 | 0 | 1 |
| EXOSC10 | 1 | 0 | 0 | 0 | 0 | 0 | 0 | 0 | 1 |
| G6PC | 1 | 0 | 0 | 0 | 0 | 0 | 0 | 0 | 1 |
| HAUS3 | 1 | 0 | 0 | 0 | 0 | 0 | 0 | 0 | 1 |
| XPR1 | 1 | 0 | 0 | 0 | 0 | 0 | 0 | 0 | 1 |
| SCFD1 | 1 | 0 | 0 | 0 | 0 | 0 | 0 | 0 | 1 |
| SLC27A6 | 1 | 0 | 0 | 0 | 0 | 0 | 0 | 0 | 1 |
| LMX1B | 1 | 0 | 0 | 0 | 0 | 0 | 0 | 0 | 1 |
| MRPS11 | 1 | 0 | 0 | 0 | 0 | 0 | 0 | 0 | 1 |
| HCRTR2 | 1 | 0 | 0 | 0 | 0 | 0 | 0 | 0 | 1 |
| ZNF566 | 1 | 0 | 0 | 0 | 0 | 0 | 0 | 0 | 1 |
| BMP10 | 1 | 0 | 0 | 0 | 0 | 0 | 0 | 0 | 1 |
| CUL4B | 1 | 0 | 0 | 0 | 0 | 0 | 0 | 0 | 1 |
| ANKRD34B | 1 | 0 | 0 | 0 | 0 | 0 | 0 | 0 | 1 |
| GALNT7 | 1 | 0 | 0 | 0 | 0 | 0 | 0 | 0 | 1 |
| SLC23A3 | 1 | 0 | 0 | 0 | 0 | 0 | 0 | 0 | 1 |
| ZNF154 | 1 | 0 | 0 | 0 | 0 | 0 | 0 | 0 | 1 |
| FKTN | 1 | 0 | 0 | 0 | 0 | 0 | 0 | 0 | 1 |
| MAPK4 | 1 | 0 | 0 | 0 | 0 | 0 | 0 | 0 | 1 |
| CYP27B1 | 1 | 0 | 0 | 0 | 0 | 0 | 0 | 0 | 1 |
| C7orf31 | 1 | 0 | 0 | 0 | 0 | 0 | 0 | 0 | 1 |
| MAPK8 | 1 | 0 | 0 | 0 | 0 | 0 | 0 | 0 | 1 |
| GRB7 | 1 | 0 | 0 | 0 | 0 | 0 | 0 | 0 | 1 |
| NRIP3 | 1 | 0 | 0 | 0 | 0 | 0 | 0 | 0 | 1 |
| MPP7 | 1 | 0 | 0 | 0 | 0 | 0 | 0 | 0 | 1 |
| DBF4B | 1 | 0 | 0 | 0 | 0 | 0 | 0 | 0 | 1 |
| C6orf70 | 1 | 0 | 0 | 0 | 0 | 0 | 0 | 0 | 1 |
| PPARA | 1 | 0 | 0 | 0 | 0 | 0 | 0 | 0 | 1 |
| PODXL | 1 | 0 | 0 | 0 | 0 | 0 | 0 | 0 | 1 |
| JMY | 1 | 0 | 0 | 0 | 0 | 0 | 0 | 0 | 1 |
| ETNK1 | 1 | 0 | 0 | 0 | 0 | 0 | 0 | 0 | 1 |
| C19orf63 | 1 | 0 | 0 | 0 | 0 | 0 | 0 | 0 | 1 |
| GPR88 | 1 | 0 | 0 | 0 | 0 | 0 | 0 | 0 | 1 |

| | | | | | | | | | |
|---|---|---|---|---|---|---|---|---|---|
| ACSS1 | 1 | 0 | 0 | 0 | 0 | 0 | 0 | 0 | 1 |
| KIF12 | 1 | 0 | 0 | 0 | 0 | 0 | 0 | 0 | 1 |
| PAF1 | 1 | 0 | 0 | 0 | 0 | 0 | 0 | 0 | 1 |
| RBM47 | 1 | 0 | 0 | 0 | 0 | 0 | 0 | 0 | 1 |
| ACRC | 1 | 0 | 0 | 0 | 0 | 0 | 0 | 0 | 1 |
| ECD | 1 | 0 | 0 | 0 | 0 | 0 | 0 | 0 | 1 |
| MCOLN2 | 1 | 0 | 0 | 0 | 0 | 0 | 0 | 0 | 1 |
| ZNF256 | 1 | 0 | 0 | 0 | 0 | 0 | 0 | 0 | 1 |
| SNTB2 | 1 | 0 | 0 | 0 | 0 | 0 | 0 | 0 | 1 |
| CASP2 | 1 | 0 | 0 | 0 | 0 | 0 | 0 | 0 | 1 |
| PIP5K1A | 1 | 0 | 0 | 0 | 0 | 0 | 0 | 0 | 1 |
| IRF6 | 1 | 0 | 0 | 0 | 0 | 0 | 0 | 0 | 1 |
| SLC7A3 | 1 | 0 | 0 | 0 | 0 | 0 | 0 | 0 | 1 |
| GHRHR | 1 | 0 | 0 | 0 | 0 | 0 | 0 | 0 | 1 |
| MTA1 | 1 | 0 | 0 | 0 | 0 | 0 | 0 | 0 | 1 |
| KRT2 | 1 | 0 | 0 | 0 | 0 | 0 | 0 | 0 | 1 |
| ACADM | 1 | 0 | 0 | 0 | 0 | 0 | 0 | 0 | 1 |
| MLPH | 1 | 0 | 0 | 0 | 0 | 0 | 0 | 0 | 1 |
| CCPG1 | 1 | 0 | 0 | 0 | 0 | 0 | 0 | 0 | 1 |
| POTEA | 1 | 0 | 0 | 0 | 0 | 0 | 0 | 0 | 1 |
| UBA1 | 1 | 0 | 0 | 0 | 0 | 0 | 0 | 0 | 1 |
| MURC | 1 | 0 | 0 | 0 | 0 | 0 | 0 | 0 | 1 |
| TMPRSS6 | 1 | 0 | 0 | 0 | 0 | 0 | 0 | 0 | 1 |
| C19orf44 | 1 | 0 | 0 | 0 | 0 | 0 | 0 | 0 | 1 |
| CBX8 | 1 | 0 | 0 | 0 | 0 | 0 | 0 | 0 | 1 |
| PPP2CB | 1 | 0 | 0 | 0 | 0 | 0 | 0 | 0 | 1 |
| SNRNP70 | 1 | 0 | 0 | 0 | 0 | 0 | 0 | 0 | 1 |
| CCR4 | 1 | 0 | 0 | 0 | 0 | 0 | 0 | 0 | 1 |
| MBD1 | 1 | 0 | 0 | 0 | 0 | 0 | 0 | 0 | 1 |
| EXD3 | 1 | 0 | 0 | 0 | 0 | 0 | 0 | 0 | 1 |
| UAP1L1 | 1 | 0 | 0 | 0 | 0 | 0 | 0 | 0 | 1 |
| ZFP14 | 1 | 0 | 0 | 0 | 0 | 0 | 0 | 0 | 1 |
| CRYZ | 1 | 0 | 0 | 0 | 0 | 0 | 0 | 0 | 1 |
| SCARB2 | 1 | 0 | 0 | 0 | 0 | 0 | 0 | 0 | 1 |
| RANBP9 | 1 | 0 | 0 | 0 | 0 | 0 | 0 | 0 | 1 |
| EXOC1 | 1 | 0 | 0 | 0 | 0 | 0 | 0 | 0 | 1 |
| CDHR4 | 1 | 0 | 0 | 0 | 0 | 0 | 0 | 0 | 1 |
| CTCFL | 1 | 0 | 0 | 0 | 0 | 0 | 0 | 0 | 1 |
| TMCC1 | 1 | 0 | 0 | 0 | 0 | 0 | 0 | 0 | 1 |
| LGI2 | 1 | 0 | 0 | 0 | 0 | 0 | 0 | 0 | 1 |
| OR51G2 | 1 | 0 | 0 | 0 | 0 | 0 | 0 | 0 | 1 |
| SCAI | 1 | 0 | 0 | 0 | 0 | 0 | 0 | 0 | 1 |
| SLC6A7 | 1 | 0 | 0 | 0 | 0 | 0 | 0 | 0 | 1 |
| RBM44 | 1 | 0 | 0 | 0 | 0 | 0 | 0 | 0 | 1 |
| MMRN2 | 1 | 0 | 0 | 0 | 0 | 0 | 0 | 0 | 1 |
| ACADVL | 1 | 0 | 0 | 0 | 0 | 0 | 0 | 0 | 1 |

| | | | | | | | | | |
|---|---|---|---|---|---|---|---|---|---|
| ZNF337 | 1 | 0 | 0 | 0 | 0 | 0 | 0 | 0 | 1 |
| DDX18 | 1 | 0 | 0 | 0 | 0 | 0 | 0 | 0 | 1 |
| NUFIP2 | 1 | 0 | 0 | 0 | 0 | 0 | 0 | 0 | 1 |
| TESK1 | 1 | 0 | 0 | 0 | 0 | 0 | 0 | 0 | 1 |
| CORO2B | 1 | 0 | 0 | 0 | 0 | 0 | 0 | 0 | 1 |
| MGAT3 | 1 | 0 | 0 | 0 | 0 | 0 | 0 | 0 | 1 |
| FBLIM1 | 1 | 0 | 0 | 0 | 0 | 0 | 0 | 0 | 1 |
| MGAT5B | 1 | 0 | 0 | 0 | 0 | 0 | 0 | 0 | 1 |
| LRRC40 | 1 | 0 | 0 | 0 | 0 | 0 | 0 | 0 | 1 |
| HOXB3 | 1 | 0 | 0 | 0 | 0 | 0 | 0 | 0 | 1 |
| ADCK2 | 1 | 0 | 0 | 0 | 0 | 0 | 0 | 0 | 1 |
| FOXR2 | 0 | 1 | 0 | 0 | 0 | 0 | 0 | 0 | 1 |
| GHDC | 1 | 0 | 0 | 0 | 0 | 0 | 0 | 0 | 1 |
| KLHL31 | 1 | 0 | 0 | 0 | 0 | 0 | 0 | 0 | 1 |
| UBQLN4 | 1 | 0 | 0 | 0 | 0 | 0 | 0 | 0 | 1 |
| OR5K3 | 1 | 0 | 0 | 0 | 0 | 0 | 0 | 0 | 1 |
| ARSG | 1 | 0 | 0 | 0 | 0 | 0 | 0 | 0 | 1 |
| BCKDHB | 1 | 0 | 0 | 0 | 0 | 0 | 0 | 0 | 1 |
| ZZZ3 | 1 | 0 | 0 | 0 | 0 | 0 | 0 | 0 | 1 |
| IGFALS | 1 | 0 | 0 | 0 | 0 | 0 | 0 | 0 | 1 |
| RBMS3 | 1 | 0 | 0 | 0 | 0 | 0 | 0 | 0 | 1 |
| ABCB8 | 1 | 0 | 0 | 0 | 0 | 0 | 0 | 0 | 1 |
| POU3F2 | 1 | 0 | 0 | 0 | 0 | 0 | 0 | 0 | 1 |
| OVGP1 | 1 | 0 | 0 | 0 | 0 | 0 | 0 | 0 | 1 |
| ACTL8 | 1 | 0 | 0 | 0 | 0 | 0 | 0 | 0 | 1 |
| MACROD2 | 1 | 0 | 0 | 0 | 0 | 0 | 0 | 0 | 1 |
| CSTF2 | 1 | 0 | 0 | 0 | 0 | 0 | 0 | 0 | 1 |
| PRDM6 | 1 | 0 | 0 | 0 | 0 | 0 | 0 | 0 | 1 |
| VN1R5 | 1 | 0 | 0 | 0 | 0 | 0 | 0 | 0 | 1 |
| UBXN11 | 1 | 0 | 0 | 0 | 0 | 0 | 0 | 0 | 1 |
| SLC44A3 | 1 | 0 | 0 | 0 | 0 | 0 | 0 | 0 | 1 |
| ZDHHC4 | 0 | 1 | 0 | 0 | 0 | 0 | 0 | 0 | 1 |
| PRKD2 | 1 | 0 | 0 | 0 | 0 | 0 | 0 | 0 | 1 |
| ATXN7L1 | 1 | 0 | 0 | 0 | 0 | 0 | 0 | 0 | 1 |
| TINAGL1 | 1 | 0 | 0 | 0 | 0 | 0 | 0 | 0 | 1 |
| SLFN12 | 1 | 0 | 0 | 0 | 0 | 0 | 0 | 0 | 1 |
| HEATR4 | 1 | 0 | 0 | 0 | 0 | 0 | 0 | 0 | 1 |
| COL4A3BP | 1 | 0 | 0 | 0 | 0 | 0 | 0 | 0 | 1 |
| CDCA7 | 1 | 0 | 0 | 0 | 0 | 0 | 0 | 0 | 1 |
| ACSBG1 | 1 | 0 | 0 | 0 | 0 | 0 | 0 | 0 | 1 |
| FXR2 | 1 | 0 | 0 | 0 | 0 | 0 | 0 | 0 | 1 |
| SEMA6A | 1 | 0 | 0 | 0 | 0 | 0 | 0 | 0 | 1 |
| GPBP1 | 1 | 0 | 0 | 0 | 0 | 0 | 0 | 0 | 1 |
| COX10 | 1 | 0 | 0 | 0 | 0 | 0 | 0 | 0 | 1 |
| SIGLEC10 | 1 | 0 | 0 | 0 | 0 | 0 | 0 | 0 | 1 |
| NFIB | 1 | 0 | 0 | 0 | 0 | 0 | 0 | 0 | 1 |

| | | | | | | | | | |
|---|---|---|---|---|---|---|---|---|---|
| FOXK1 | 1 | 0 | 0 | 0 | 0 | 0 | 0 | 0 | 1 |
| SIX5 | 1 | 0 | 0 | 0 | 0 | 0 | 0 | 0 | 1 |
| CECR5 | 1 | 0 | 0 | 0 | 0 | 0 | 0 | 0 | 1 |
| ELFN1 | 1 | 0 | 0 | 0 | 0 | 0 | 0 | 0 | 1 |
| TMC8 | 1 | 0 | 0 | 0 | 0 | 0 | 0 | 0 | 1 |
| RSPH6A | 1 | 0 | 0 | 0 | 0 | 0 | 0 | 0 | 1 |
| FAF1 | 1 | 0 | 0 | 0 | 0 | 0 | 0 | 0 | 1 |
| PITPNM3 | 1 | 0 | 0 | 0 | 0 | 0 | 0 | 0 | 1 |
| UBA2 | 1 | 0 | 0 | 0 | 0 | 0 | 0 | 0 | 1 |
| SF3A1 | 1 | 0 | 0 | 0 | 0 | 0 | 0 | 0 | 1 |
| EIF2AK1 | 1 | 0 | 0 | 0 | 0 | 0 | 0 | 0 | 1 |
| CUZD1 | 1 | 0 | 0 | 0 | 0 | 0 | 0 | 0 | 1 |
| RGS9 | 1 | 0 | 0 | 0 | 0 | 0 | 0 | 0 | 1 |
| OR9K2 | 1 | 0 | 0 | 0 | 0 | 0 | 0 | 0 | 1 |
| RAPGEF3 | 1 | 0 | 0 | 0 | 0 | 0 | 0 | 0 | 1 |
| PHEX | 1 | 0 | 0 | 0 | 0 | 0 | 0 | 0 | 1 |
| SMO | 1 | 0 | 0 | 0 | 0 | 0 | 0 | 0 | 1 |
| TAS2R1 | 1 | 0 | 0 | 0 | 0 | 0 | 0 | 0 | 1 |
| SH2B1 | 1 | 0 | 0 | 0 | 0 | 0 | 0 | 0 | 1 |
| SLC47A1 | 1 | 0 | 0 | 0 | 0 | 0 | 0 | 0 | 1 |
| CYP4F12 | 1 | 0 | 0 | 0 | 0 | 0 | 0 | 0 | 1 |
| TUB | 1 | 0 | 0 | 0 | 0 | 0 | 0 | 0 | 1 |
| SLC9A8 | 1 | 0 | 0 | 0 | 0 | 0 | 0 | 0 | 1 |
| LRRC14B | 1 | 0 | 0 | 0 | 0 | 0 | 0 | 0 | 1 |
| ANKRD13A | 1 | 0 | 0 | 0 | 0 | 0 | 0 | 0 | 1 |
| KIF5B | 1 | 0 | 0 | 0 | 0 | 0 | 0 | 0 | 1 |
| PAK1 | 1 | 0 | 0 | 0 | 0 | 0 | 0 | 0 | 1 |
| CHPF2 | 1 | 0 | 0 | 0 | 0 | 0 | 0 | 0 | 1 |
| ARHGAP6 | 1 | 0 | 0 | 0 | 0 | 0 | 0 | 0 | 1 |
| PRR14 | 1 | 0 | 0 | 0 | 0 | 0 | 0 | 0 | 1 |
| CAPN12 | 1 | 0 | 0 | 0 | 0 | 0 | 0 | 0 | 1 |
| TBC1D25 | 1 | 0 | 0 | 0 | 0 | 0 | 0 | 0 | 1 |
| OR8D1 | 1 | 0 | 0 | 0 | 0 | 0 | 0 | 0 | 1 |
| C20orf151 | 1 | 0 | 0 | 0 | 0 | 0 | 0 | 0 | 1 |
| VIPR2 | 1 | 0 | 0 | 0 | 0 | 0 | 0 | 0 | 1 |
| DYRK4 | 1 | 0 | 0 | 0 | 0 | 0 | 0 | 0 | 1 |
| PRKCH | 1 | 0 | 0 | 0 | 0 | 0 | 0 | 0 | 1 |
| ZNF25 | 1 | 0 | 0 | 0 | 0 | 0 | 0 | 0 | 1 |
| CYP2U1 | 1 | 0 | 0 | 0 | 0 | 0 | 0 | 0 | 1 |
| JPH2 | 1 | 0 | 0 | 0 | 0 | 0 | 0 | 0 | 1 |
| UGT2B4 | 0 | 1 | 0 | 0 | 0 | 0 | 0 | 0 | 1 |
| SLC8A2 | 1 | 0 | 0 | 0 | 0 | 0 | 0 | 0 | 1 |
| OR13F1 | 1 | 0 | 0 | 0 | 0 | 0 | 0 | 0 | 1 |
| MAN2C1 | 1 | 0 | 0 | 0 | 0 | 0 | 0 | 0 | 1 |
| MYBL1 | 1 | 0 | 0 | 0 | 0 | 0 | 0 | 0 | 1 |
| NOP2 | 1 | 0 | 0 | 0 | 0 | 0 | 0 | 0 | 1 |

| | | | | | | | | | |
|---|---|---|---|---|---|---|---|---|---|
| KCNQ1 | 1 | 0 | 0 | 0 | 0 | 0 | 0 | 0 | 1 |
| BEND3 | 1 | 0 | 0 | 0 | 0 | 0 | 0 | 0 | 1 |
| TRIM2 | 1 | 0 | 0 | 0 | 0 | 0 | 0 | 0 | 1 |
| ENC1 | 1 | 0 | 0 | 0 | 0 | 0 | 0 | 0 | 1 |
| ZNF582 | 1 | 0 | 0 | 0 | 0 | 0 | 0 | 0 | 1 |
| TLE6 | 1 | 0 | 0 | 0 | 0 | 0 | 0 | 0 | 1 |
| MYO19 | 1 | 0 | 0 | 0 | 0 | 0 | 0 | 0 | 1 |
| AOX1 | 0 | 0 | 0 | 0 | 1 | 0 | 0 | 0 | 1 |
| SNAPC4 | 0 | 1 | 0 | 0 | 0 | 0 | 0 | 0 | 1 |
| VGLL3 | 1 | 0 | 0 | 0 | 0 | 0 | 0 | 0 | 1 |
| ERAP2 | 1 | 0 | 0 | 0 | 0 | 0 | 0 | 0 | 1 |
| FCHSD1 | 1 | 0 | 0 | 0 | 0 | 0 | 0 | 0 | 1 |
| RASSF4 | 1 | 0 | 0 | 0 | 0 | 0 | 0 | 0 | 1 |
| CPNE7 | 1 | 0 | 0 | 0 | 0 | 0 | 0 | 0 | 1 |
| ESYT2 | 1 | 0 | 0 | 0 | 0 | 0 | 0 | 0 | 1 |
| ZNF28 | 1 | 0 | 0 | 0 | 0 | 0 | 0 | 0 | 1 |
| RIN1 | 1 | 0 | 0 | 0 | 0 | 0 | 0 | 0 | 1 |
| DALRD3 | 1 | 0 | 0 | 0 | 0 | 0 | 0 | 0 | 1 |
| GABRE | 1 | 0 | 0 | 0 | 0 | 0 | 0 | 0 | 1 |
| CYP2J2 | 1 | 0 | 0 | 0 | 0 | 0 | 0 | 0 | 1 |
| KCNJ1 | 1 | 0 | 0 | 0 | 0 | 0 | 0 | 0 | 1 |
| CD300LB | 1 | 0 | 0 | 0 | 0 | 0 | 0 | 0 | 1 |
| MAGEB18 | 1 | 0 | 0 | 0 | 0 | 0 | 0 | 0 | 1 |
| CA2 | 1 | 0 | 0 | 0 | 0 | 0 | 0 | 0 | 1 |
| TMEM63B | 1 | 0 | 0 | 0 | 0 | 0 | 0 | 0 | 1 |
| KLKB1 | 1 | 0 | 0 | 0 | 0 | 0 | 0 | 0 | 1 |
| RRP8 | 1 | 0 | 0 | 0 | 0 | 0 | 0 | 0 | 1 |
| OSBP2 | 1 | 0 | 0 | 0 | 0 | 0 | 0 | 0 | 1 |
| TXNDC11 | 1 | 0 | 0 | 0 | 0 | 0 | 0 | 0 | 1 |
| FAM46C | 1 | 0 | 0 | 0 | 0 | 0 | 0 | 0 | 1 |
| GATA6 | 1 | 0 | 0 | 0 | 0 | 0 | 0 | 0 | 1 |
| CMAS | 1 | 0 | 0 | 0 | 0 | 0 | 0 | 0 | 1 |
| ZNF300 | 1 | 0 | 0 | 0 | 0 | 0 | 0 | 0 | 1 |
| KPNB1 | 1 | 0 | 0 | 0 | 0 | 0 | 0 | 0 | 1 |
| EPB41 | 1 | 0 | 0 | 0 | 0 | 0 | 0 | 0 | 1 |
| NYX | 1 | 0 | 0 | 0 | 0 | 0 | 0 | 0 | 1 |
| MSLN | 1 | 0 | 0 | 0 | 0 | 0 | 0 | 0 | 1 |
| PTCHD1 | 1 | 0 | 0 | 0 | 0 | 0 | 0 | 0 | 1 |
| TRIM68 | 1 | 0 | 0 | 0 | 0 | 0 | 0 | 0 | 1 |
| FBXW8 | 1 | 0 | 0 | 0 | 0 | 0 | 0 | 0 | 1 |
| RIPK3 | 1 | 0 | 0 | 0 | 0 | 0 | 0 | 0 | 1 |
| NASP | 1 | 0 | 0 | 0 | 0 | 0 | 0 | 0 | 1 |
| BRD2 | 1 | 0 | 0 | 0 | 0 | 0 | 0 | 0 | 1 |
| GPATCH3 | 1 | 0 | 0 | 0 | 0 | 0 | 0 | 0 | 1 |
| TLK2 | 1 | 0 | 0 | 0 | 0 | 0 | 0 | 0 | 1 |
| ARAF | 1 | 0 | 0 | 0 | 0 | 0 | 0 | 0 | 1 |

| | | | | | | | | | |
|---|---|---|---|---|---|---|---|---|---|
| SHCBP1 | 1 | 0 | 0 | 0 | 0 | 0 | 0 | 0 | 1 |
| TMC7 | 1 | 0 | 0 | 0 | 0 | 0 | 0 | 0 | 1 |
| RUSC1 | 1 | 0 | 0 | 0 | 0 | 0 | 0 | 0 | 1 |
| RAD21 | 1 | 0 | 0 | 0 | 0 | 0 | 0 | 0 | 1 |
| IGSF3 | 1 | 0 | 0 | 0 | 0 | 0 | 0 | 0 | 1 |
| OR5B3 | 1 | 0 | 0 | 0 | 0 | 0 | 0 | 0 | 1 |
| HHAT | 1 | 0 | 0 | 0 | 0 | 0 | 0 | 0 | 1 |
| GIT1 | 1 | 0 | 0 | 0 | 0 | 0 | 0 | 0 | 1 |
| TATDN2 | 1 | 0 | 0 | 0 | 0 | 0 | 0 | 0 | 1 |
| BBS12 | 1 | 0 | 0 | 0 | 0 | 0 | 0 | 0 | 1 |
| CHFR | 1 | 0 | 0 | 0 | 0 | 0 | 0 | 0 | 1 |
| OS9 | 1 | 0 | 0 | 0 | 0 | 0 | 0 | 0 | 1 |
| HERC5 | 1 | 0 | 0 | 0 | 0 | 0 | 0 | 0 | 1 |
| FNIP2 | 1 | 0 | 0 | 0 | 0 | 0 | 0 | 0 | 1 |
| ST6GALNAC5 | 1 | 0 | 0 | 0 | 0 | 0 | 0 | 0 | 1 |
| SIRT3 | 1 | 0 | 0 | 0 | 0 | 0 | 0 | 0 | 1 |
| CD300C | 1 | 0 | 0 | 0 | 0 | 0 | 0 | 0 | 1 |
| SEMA3C | 1 | 0 | 0 | 0 | 0 | 0 | 0 | 0 | 1 |
| SLC45A1 | 1 | 0 | 0 | 0 | 0 | 0 | 0 | 0 | 1 |
| HIF3A | 1 | 0 | 0 | 0 | 0 | 0 | 0 | 0 | 1 |
| UTP14A | 1 | 0 | 0 | 0 | 0 | 0 | 0 | 0 | 1 |
| EXTL3 | 1 | 0 | 0 | 0 | 0 | 0 | 0 | 0 | 1 |
| SCNN1B | 1 | 0 | 0 | 0 | 0 | 0 | 0 | 0 | 1 |
| CDK17 | 1 | 0 | 0 | 0 | 0 | 0 | 0 | 0 | 1 |
| CRY2 | 1 | 0 | 0 | 0 | 0 | 0 | 0 | 0 | 1 |
| TMEM173 | 1 | 0 | 0 | 0 | 0 | 0 | 0 | 0 | 1 |
| MKLN1 | 1 | 0 | 0 | 0 | 0 | 0 | 0 | 0 | 1 |
| OR56A1 | 1 | 0 | 0 | 0 | 0 | 0 | 0 | 0 | 1 |
| PCDHGC4 | 1 | 0 | 0 | 0 | 0 | 0 | 0 | 0 | 1 |
| CCDC112 | 1 | 0 | 0 | 0 | 0 | 0 | 0 | 0 | 1 |
| FAM161B | 1 | 0 | 0 | 0 | 0 | 0 | 0 | 0 | 1 |
| SNCAIP | 1 | 0 | 0 | 0 | 0 | 0 | 0 | 0 | 1 |
| TES | 0 | 1 | 0 | 0 | 0 | 0 | 0 | 0 | 1 |
| CNOT10 | 1 | 0 | 0 | 0 | 0 | 0 | 0 | 0 | 1 |
| ZNF445 | 1 | 0 | 0 | 0 | 0 | 0 | 0 | 0 | 1 |
| ZNF615 | 1 | 0 | 0 | 0 | 0 | 0 | 0 | 0 | 1 |
| CYLC2 | 1 | 0 | 0 | 0 | 0 | 0 | 0 | 0 | 1 |
| TSSC1 | 1 | 0 | 0 | 0 | 0 | 0 | 0 | 0 | 1 |
| ZMYM1 | 1 | 0 | 0 | 0 | 0 | 0 | 0 | 0 | 1 |
| C16orf11 | 1 | 0 | 0 | 0 | 0 | 0 | 0 | 0 | 1 |
| OR1E2 | 1 | 0 | 0 | 0 | 0 | 0 | 0 | 0 | 1 |
| NAT10 | 1 | 0 | 0 | 0 | 0 | 0 | 0 | 0 | 1 |
| GNL1 | 1 | 0 | 0 | 0 | 0 | 0 | 0 | 0 | 1 |
| BRDT | 1 | 0 | 0 | 0 | 0 | 0 | 0 | 0 | 1 |
| SKIV2L2 | 1 | 0 | 0 | 0 | 0 | 0 | 0 | 0 | 1 |
| GALNT14 | 1 | 0 | 0 | 0 | 0 | 0 | 0 | 0 | 1 |

| | | | | | | | | | |
|---|---|---|---|---|---|---|---|---|---|
| BMPR1B | 1 | 0 | 0 | 0 | 0 | 0 | 0 | 0 | 1 |
| EPB42 | 1 | 0 | 0 | 0 | 0 | 0 | 0 | 0 | 1 |
| CWH43 | 1 | 0 | 0 | 0 | 0 | 0 | 0 | 0 | 1 |
| PPP2R1A | 1 | 0 | 0 | 0 | 0 | 0 | 0 | 0 | 1 |
| UPF1 | 1 | 0 | 0 | 0 | 0 | 0 | 0 | 0 | 1 |
| DBF4 | 1 | 0 | 0 | 0 | 0 | 0 | 0 | 0 | 1 |
| DMTF1 | 1 | 0 | 0 | 0 | 0 | 0 | 0 | 0 | 1 |
| PPP1R12B | 0 | 0 | 0 | 0 | 1 | 0 | 0 | 0 | 1 |
| GPRIN3 | 1 | 0 | 0 | 0 | 0 | 0 | 0 | 0 | 1 |
| HELLS | 1 | 0 | 0 | 0 | 0 | 0 | 0 | 0 | 1 |
| ZBTB17 | 1 | 0 | 0 | 0 | 0 | 0 | 0 | 0 | 1 |
| MAPK7 | 1 | 0 | 0 | 0 | 0 | 0 | 0 | 0 | 1 |
| RGS13 | 1 | 0 | 0 | 0 | 0 | 0 | 0 | 0 | 1 |
| FRMD4A | 1 | 0 | 0 | 0 | 0 | 0 | 0 | 0 | 1 |
| ZNF571 | 1 | 0 | 0 | 0 | 0 | 0 | 0 | 0 | 1 |
| EXOC5 | 1 | 0 | 0 | 0 | 0 | 0 | 0 | 0 | 1 |
| PBLD | 1 | 0 | 0 | 0 | 0 | 0 | 0 | 0 | 1 |
| MTMR14 | 1 | 0 | 0 | 0 | 0 | 0 | 0 | 0 | 1 |
| CNGA1 | 1 | 0 | 0 | 0 | 0 | 0 | 0 | 0 | 1 |
| STIM2 | 1 | 0 | 0 | 0 | 0 | 0 | 0 | 0 | 1 |
| SLC44A1 | 1 | 0 | 0 | 0 | 0 | 0 | 0 | 0 | 1 |
| ZNF467 | 1 | 0 | 0 | 0 | 0 | 0 | 0 | 0 | 1 |
| CLSTN3 | 1 | 0 | 0 | 0 | 0 | 0 | 0 | 0 | 1 |
| PACS2 | 1 | 0 | 0 | 0 | 0 | 0 | 0 | 0 | 1 |
| ACAT1 | 1 | 0 | 0 | 0 | 0 | 0 | 0 | 0 | 1 |
| MTMR12 | 1 | 0 | 0 | 0 | 0 | 0 | 0 | 0 | 1 |
| TP73 | 1 | 0 | 0 | 0 | 0 | 0 | 0 | 0 | 1 |
| GPR65 | 1 | 0 | 0 | 0 | 0 | 0 | 0 | 0 | 1 |
| HIPK4 | 1 | 0 | 0 | 0 | 0 | 0 | 0 | 0 | 1 |
| ARMC4 | 1 | 0 | 0 | 0 | 0 | 0 | 0 | 0 | 1 |
| SH3RF1 | 1 | 0 | 0 | 0 | 0 | 0 | 0 | 0 | 1 |
| SCNN1A | 1 | 0 | 0 | 0 | 0 | 0 | 0 | 0 | 1 |
| PCDHGC3 | 1 | 0 | 0 | 0 | 0 | 0 | 0 | 0 | 1 |
| NLK | 1 | 0 | 0 | 0 | 0 | 0 | 0 | 0 | 1 |
| FAM160B2 | 1 | 0 | 0 | 0 | 0 | 0 | 0 | 0 | 1 |
| LRRC27 | 1 | 0 | 0 | 0 | 0 | 0 | 0 | 0 | 1 |
| ZNF418 | 1 | 0 | 0 | 0 | 0 | 0 | 0 | 0 | 1 |
| GGA2 | 1 | 0 | 0 | 0 | 0 | 0 | 0 | 0 | 1 |
| NIPSNAP3A | 1 | 0 | 0 | 0 | 0 | 0 | 0 | 0 | 1 |
| SH3RF3 | 1 | 0 | 0 | 0 | 0 | 0 | 0 | 0 | 1 |
| RASSF9 | 1 | 0 | 0 | 0 | 0 | 0 | 0 | 0 | 1 |
| POLM | 1 | 0 | 0 | 0 | 0 | 0 | 0 | 0 | 1 |
| NAA16 | 1 | 0 | 0 | 0 | 0 | 0 | 0 | 0 | 1 |
| PPM1J | 1 | 0 | 0 | 0 | 0 | 0 | 0 | 0 | 1 |
| RGR | 1 | 0 | 0 | 0 | 0 | 0 | 0 | 0 | 1 |
| ARHGEF6 | 1 | 0 | 0 | 0 | 0 | 0 | 0 | 0 | 1 |

| | | | | | | | | | |
|---|---|---|---|---|---|---|---|---|---|
| SSRP1 | 1 | 0 | 0 | 0 | 0 | 0 | 0 | 0 | 1 |
| CEP120 | 1 | 0 | 0 | 0 | 0 | 0 | 0 | 0 | 1 |
| CCDC138 | 1 | 0 | 0 | 0 | 0 | 0 | 0 | 0 | 1 |
| CYB5R4 | 1 | 0 | 0 | 0 | 0 | 0 | 0 | 0 | 1 |
| GIGYF2 | 1 | 0 | 0 | 0 | 0 | 0 | 0 | 0 | 1 |
| LRCH2 | 1 | 0 | 0 | 0 | 0 | 0 | 0 | 0 | 1 |
| SFPQ | 1 | 0 | 0 | 0 | 0 | 0 | 0 | 0 | 1 |
| PCDHB9 | 1 | 0 | 0 | 0 | 0 | 0 | 0 | 0 | 1 |
| OR6C3 | 1 | 0 | 0 | 0 | 0 | 0 | 0 | 0 | 1 |
| RBMX | 1 | 0 | 0 | 0 | 0 | 0 | 0 | 0 | 1 |
| PARP10 | 1 | 0 | 0 | 0 | 0 | 0 | 0 | 0 | 1 |
| ZNF8 | 1 | 0 | 0 | 0 | 0 | 0 | 0 | 0 | 1 |
| ZNF461 | 1 | 0 | 0 | 0 | 0 | 0 | 0 | 0 | 1 |
| RHBDF2 | 1 | 0 | 0 | 0 | 0 | 0 | 0 | 0 | 1 |
| ERO1L | 1 | 0 | 0 | 0 | 0 | 0 | 0 | 0 | 1 |
| ZNFX1 | 1 | 0 | 0 | 0 | 0 | 0 | 0 | 0 | 1 |
| TBR1 | 1 | 0 | 0 | 0 | 0 | 0 | 0 | 0 | 1 |
| METAP1 | 1 | 0 | 0 | 0 | 0 | 0 | 0 | 0 | 1 |
| CLK3 | 1 | 0 | 0 | 0 | 0 | 0 | 0 | 0 | 1 |
| PDYN | 1 | 0 | 0 | 0 | 0 | 0 | 0 | 0 | 1 |
| NADSYN1 | 1 | 0 | 0 | 0 | 0 | 0 | 0 | 0 | 1 |
| SLC18A2 | 1 | 0 | 0 | 0 | 0 | 0 | 0 | 0 | 1 |
| KIFC3 | 1 | 0 | 0 | 0 | 0 | 0 | 0 | 0 | 1 |
| PPP4R1 | 1 | 0 | 0 | 0 | 0 | 0 | 0 | 0 | 1 |
| NUP88 | 1 | 0 | 0 | 0 | 0 | 0 | 0 | 0 | 1 |
| IFT74 | 1 | 0 | 0 | 0 | 0 | 0 | 0 | 0 | 1 |
| IL3 | 1 | 0 | 0 | 0 | 0 | 0 | 0 | 0 | 1 |
| ELF4 | 1 | 0 | 0 | 0 | 0 | 0 | 0 | 0 | 1 |
| PRPF4 | 1 | 0 | 0 | 0 | 0 | 0 | 0 | 0 | 1 |
| TNS4 | 1 | 0 | 0 | 0 | 0 | 0 | 0 | 0 | 1 |
| LCA5L | 1 | 0 | 0 | 0 | 0 | 0 | 0 | 0 | 1 |
| SNX5 | 1 | 0 | 0 | 0 | 0 | 0 | 0 | 0 | 1 |
| SPTLC3 | 1 | 0 | 0 | 0 | 0 | 0 | 0 | 0 | 1 |
| PIGO | 1 | 0 | 0 | 0 | 0 | 0 | 0 | 0 | 1 |
| AKAP8 | 1 | 0 | 0 | 0 | 0 | 0 | 0 | 0 | 1 |
| KIAA1161 | 1 | 0 | 0 | 0 | 0 | 0 | 0 | 0 | 1 |
| ACD | 1 | 0 | 0 | 0 | 0 | 0 | 0 | 0 | 1 |
| PLOD2 | 1 | 0 | 0 | 0 | 0 | 0 | 0 | 0 | 1 |
| PCDHGA11 | 1 | 0 | 0 | 0 | 0 | 0 | 0 | 0 | 1 |
| SMCR8 | 1 | 0 | 0 | 0 | 0 | 0 | 0 | 0 | 1 |
| AXIN1 | 1 | 0 | 0 | 0 | 0 | 0 | 0 | 0 | 1 |
| ERF | 1 | 0 | 0 | 0 | 0 | 0 | 0 | 0 | 1 |
| PJA1 | 1 | 0 | 0 | 0 | 0 | 0 | 0 | 0 | 1 |
| IL17RC | 1 | 0 | 0 | 0 | 0 | 0 | 0 | 0 | 1 |
| DHX35 | 1 | 0 | 0 | 0 | 0 | 0 | 0 | 0 | 1 |
| ITIH1 | 1 | 0 | 0 | 0 | 0 | 0 | 0 | 0 | 1 |

| | | | | | | | | | |
|---|---|---|---|---|---|---|---|---|---|
| TGFBRAP1 | 1 | 0 | 0 | 0 | 0 | 0 | 0 | 0 | 1 |
| PITX1 | 1 | 0 | 0 | 0 | 0 | 0 | 0 | 0 | 1 |
| RFX3 | 1 | 0 | 0 | 0 | 0 | 0 | 0 | 0 | 1 |
| CHRM2 | 1 | 0 | 0 | 0 | 0 | 0 | 0 | 0 | 1 |
| AP3S1 | 1 | 0 | 0 | 0 | 0 | 0 | 0 | 0 | 1 |
| ICK | 1 | 0 | 0 | 0 | 0 | 0 | 0 | 0 | 1 |
| KDM2A | 1 | 0 | 0 | 0 | 0 | 0 | 0 | 0 | 1 |
| PNLIPRP1 | 1 | 0 | 0 | 0 | 0 | 0 | 0 | 0 | 1 |
| CSTF2T | 1 | 0 | 0 | 0 | 0 | 0 | 0 | 0 | 1 |
| GUCY2D | 1 | 0 | 0 | 0 | 0 | 0 | 0 | 0 | 1 |
| ZMIZ2 | 1 | 0 | 0 | 0 | 0 | 0 | 0 | 0 | 1 |
| FAM120C | 1 | 0 | 0 | 0 | 0 | 0 | 0 | 0 | 1 |
| GC | 1 | 0 | 0 | 0 | 0 | 0 | 0 | 0 | 1 |
| VPS41 | 1 | 0 | 0 | 0 | 0 | 0 | 0 | 0 | 1 |
| RAB34 | 1 | 0 | 0 | 0 | 0 | 0 | 0 | 0 | 1 |
| ANO7 | 1 | 0 | 0 | 0 | 0 | 0 | 0 | 0 | 1 |
| FLCN | 1 | 0 | 0 | 0 | 0 | 0 | 0 | 0 | 1 |
| TBC1D10B | 1 | 0 | 0 | 0 | 0 | 0 | 0 | 0 | 1 |
| SIK1 | 1 | 0 | 0 | 0 | 0 | 0 | 0 | 0 | 1 |
| SLC9A4 | 1 | 0 | 0 | 0 | 0 | 0 | 0 | 0 | 1 |
| DNAJC2 | 1 | 0 | 0 | 0 | 0 | 0 | 0 | 0 | 1 |
| EPHA4 | 1 | 0 | 0 | 0 | 0 | 0 | 0 | 0 | 1 |
| TCTE1 | 1 | 0 | 0 | 0 | 0 | 0 | 0 | 0 | 1 |
| HEPACAM | 1 | 0 | 0 | 0 | 0 | 0 | 0 | 0 | 1 |
| FAP | 1 | 0 | 0 | 0 | 0 | 0 | 0 | 0 | 1 |
| ARHGEF16 | 1 | 0 | 0 | 0 | 0 | 0 | 0 | 0 | 1 |
| TPCN1 | 1 | 0 | 0 | 0 | 0 | 0 | 0 | 0 | 1 |
| GOLGA2 | 0 | 1 | 0 | 0 | 0 | 0 | 0 | 0 | 1 |
| HAP1 | 1 | 0 | 0 | 0 | 0 | 0 | 0 | 0 | 1 |
| ZNF703 | 1 | 0 | 0 | 0 | 0 | 0 | 0 | 0 | 1 |
| FBLN1 | 1 | 0 | 0 | 0 | 0 | 0 | 0 | 0 | 1 |
| MED24 | 1 | 0 | 0 | 0 | 0 | 0 | 0 | 0 | 1 |
| NEDD9 | 1 | 0 | 0 | 0 | 0 | 0 | 0 | 0 | 1 |
| SUSD2 | 1 | 0 | 0 | 0 | 0 | 0 | 0 | 0 | 1 |
| ARHGAP42 | 1 | 0 | 0 | 0 | 0 | 0 | 0 | 0 | 1 |
| KIT | 1 | 0 | 0 | 0 | 0 | 0 | 0 | 0 | 1 |
| ZNF326 | 1 | 0 | 0 | 0 | 0 | 0 | 0 | 0 | 1 |
| KIF4A | 1 | 0 | 0 | 0 | 0 | 0 | 0 | 0 | 1 |
| KBTBD7 | 1 | 0 | 0 | 0 | 0 | 0 | 0 | 0 | 1 |
| PRF1 | 1 | 0 | 0 | 0 | 0 | 0 | 0 | 0 | 1 |
| DDN | 1 | 0 | 0 | 0 | 0 | 0 | 0 | 0 | 1 |
| SYN1 | 1 | 0 | 0 | 0 | 0 | 0 | 0 | 0 | 1 |
| GPC3 | 1 | 0 | 0 | 0 | 0 | 0 | 0 | 0 | 1 |
| SUN2 | 1 | 0 | 0 | 0 | 0 | 0 | 0 | 0 | 1 |
| CNGA4 | 1 | 0 | 0 | 0 | 0 | 0 | 0 | 0 | 1 |
| CHPF | 1 | 0 | 0 | 0 | 0 | 0 | 0 | 0 | 1 |

| | | | | | | | | | |
|---|---|---|---|---|---|---|---|---|---|
| RTEL1 | 1 | 0 | 0 | 0 | 0 | 0 | 0 | 0 | 1 |
| ZNF618 | 1 | 0 | 0 | 0 | 0 | 0 | 0 | 0 | 1 |
| OR2M4 | 1 | 0 | 0 | 0 | 0 | 0 | 0 | 0 | 1 |
| WDR91 | 1 | 0 | 0 | 0 | 0 | 0 | 0 | 0 | 1 |
| JARID2 | 0 | 0 | 0 | 0 | 1 | 0 | 0 | 0 | 1 |
| ATG9A | 1 | 0 | 0 | 0 | 0 | 0 | 0 | 0 | 1 |
| SNX14 | 1 | 0 | 0 | 0 | 0 | 0 | 0 | 0 | 1 |
| GZF1 | 1 | 0 | 0 | 0 | 0 | 0 | 0 | 0 | 1 |
| ACCS | 1 | 0 | 0 | 0 | 0 | 0 | 0 | 0 | 1 |
| ZNF577 | 1 | 0 | 0 | 0 | 0 | 0 | 0 | 0 | 1 |
| HIPK3 | 1 | 0 | 0 | 0 | 0 | 0 | 0 | 0 | 1 |
| USP40 | 1 | 0 | 0 | 0 | 0 | 0 | 0 | 0 | 1 |
| NPY5R | 1 | 0 | 0 | 0 | 0 | 0 | 0 | 0 | 1 |
| C1S | 1 | 0 | 0 | 0 | 0 | 0 | 0 | 0 | 1 |
| SUSD1 | 1 | 0 | 0 | 0 | 0 | 0 | 0 | 0 | 1 |
| ZNF43 | 1 | 0 | 0 | 0 | 0 | 0 | 0 | 0 | 1 |
| IGHMBP2 | 1 | 0 | 0 | 0 | 0 | 0 | 0 | 0 | 1 |
| PLA2G4F | 1 | 0 | 0 | 0 | 0 | 0 | 0 | 0 | 1 |
| STAT4 | 1 | 0 | 0 | 0 | 0 | 0 | 0 | 0 | 1 |
| YY1AP1 | 1 | 0 | 0 | 0 | 0 | 0 | 0 | 0 | 1 |
| PLEKHG6 | 1 | 0 | 0 | 0 | 0 | 0 | 0 | 0 | 1 |
| TOX | 1 | 0 | 0 | 0 | 0 | 0 | 0 | 0 | 1 |
| ITIH4 | 1 | 0 | 0 | 0 | 0 | 0 | 0 | 0 | 1 |
| PCCA | 1 | 0 | 0 | 0 | 0 | 0 | 0 | 0 | 1 |
| ZFPM1 | 1 | 0 | 0 | 0 | 0 | 0 | 0 | 0 | 1 |
| BTBD9 | 1 | 0 | 0 | 0 | 0 | 0 | 0 | 0 | 1 |
| LPIN3 | 1 | 0 | 0 | 0 | 0 | 0 | 0 | 0 | 1 |
| PLCD1 | 1 | 0 | 0 | 0 | 0 | 0 | 0 | 0 | 1 |
| FNIP1 | 1 | 0 | 0 | 0 | 0 | 0 | 0 | 0 | 1 |
| ANKRD32 | 1 | 0 | 0 | 0 | 0 | 0 | 0 | 0 | 1 |
| TACC1 | 1 | 0 | 0 | 0 | 0 | 0 | 0 | 0 | 1 |
| SNX18 | 1 | 0 | 0 | 0 | 0 | 0 | 0 | 0 | 1 |
| OR10S1 | 1 | 0 | 0 | 0 | 0 | 0 | 0 | 0 | 1 |
| CACNB1 | 1 | 0 | 0 | 0 | 0 | 0 | 0 | 0 | 1 |
| CAPNS1 | 1 | 0 | 0 | 0 | 0 | 0 | 0 | 0 | 1 |
| ZNF202 | 1 | 0 | 0 | 0 | 0 | 0 | 0 | 0 | 1 |
| DEFB116 | 1 | 0 | 0 | 0 | 0 | 0 | 0 | 0 | 1 |
| FBLN2 | 1 | 0 | 0 | 0 | 0 | 0 | 0 | 0 | 1 |
| MAG | 1 | 0 | 0 | 0 | 0 | 0 | 0 | 0 | 1 |
| LNX2 | 1 | 0 | 0 | 0 | 0 | 0 | 0 | 0 | 1 |
| ISM1 | 1 | 0 | 0 | 0 | 0 | 0 | 0 | 0 | 1 |
| STAT6 | 1 | 0 | 0 | 0 | 0 | 0 | 0 | 0 | 1 |
| DNER | 1 | 0 | 0 | 0 | 0 | 0 | 0 | 0 | 1 |
| SLC2A13 | 1 | 0 | 0 | 0 | 0 | 0 | 0 | 0 | 1 |
| TMEM206 | 1 | 0 | 0 | 0 | 0 | 0 | 0 | 0 | 1 |
| CDYL2 | 1 | 0 | 0 | 0 | 0 | 0 | 0 | 0 | 1 |

| | | | | | | | | | |
|---|---|---|---|---|---|---|---|---|---|
| CHEK1 | 1 | 0 | 0 | 0 | 0 | 0 | 0 | 0 | 1 |
| ZNF211 | 1 | 0 | 0 | 0 | 0 | 0 | 0 | 0 | 1 |
| CHST8 | 1 | 0 | 0 | 0 | 0 | 0 | 0 | 0 | 1 |
| NXNL1 | 1 | 0 | 0 | 0 | 0 | 0 | 0 | 0 | 1 |
| RAB3C | 1 | 0 | 0 | 0 | 0 | 0 | 0 | 0 | 1 |
| COPB1 | 1 | 0 | 0 | 0 | 0 | 0 | 0 | 0 | 1 |
| EFR3B | 1 | 0 | 0 | 0 | 0 | 0 | 0 | 0 | 1 |
| UBA7 | 1 | 0 | 0 | 0 | 0 | 0 | 0 | 0 | 1 |
| CORIN | 1 | 0 | 0 | 0 | 0 | 0 | 0 | 0 | 1 |
| SLC6A4 | 1 | 0 | 0 | 0 | 0 | 0 | 0 | 0 | 1 |
| EEF2 | 1 | 0 | 0 | 0 | 0 | 0 | 0 | 0 | 1 |
| CD2AP | 1 | 0 | 0 | 0 | 0 | 0 | 0 | 0 | 1 |
| EYA3 | 1 | 0 | 0 | 0 | 0 | 0 | 0 | 0 | 1 |
| DACT2 | 1 | 0 | 0 | 0 | 0 | 0 | 0 | 0 | 1 |
| FERMT2 | 1 | 0 | 0 | 0 | 0 | 0 | 0 | 0 | 1 |
| EBF3 | 1 | 0 | 0 | 0 | 0 | 0 | 0 | 0 | 1 |
| BBS4 | 1 | 0 | 0 | 0 | 0 | 0 | 0 | 0 | 1 |
| SRRT | 1 | 0 | 0 | 0 | 0 | 0 | 0 | 0 | 1 |
| TMEM67 | 1 | 0 | 0 | 0 | 0 | 0 | 0 | 0 | 1 |
| PIAS3 | 1 | 0 | 0 | 0 | 0 | 0 | 0 | 0 | 1 |
| CARD10 | 1 | 0 | 0 | 0 | 0 | 0 | 0 | 0 | 1 |
| PHF17 | 1 | 0 | 0 | 0 | 0 | 0 | 0 | 0 | 1 |
| PITRM1 | 1 | 0 | 0 | 0 | 0 | 0 | 0 | 0 | 1 |
| SERPINA10 | 1 | 0 | 0 | 0 | 0 | 0 | 0 | 0 | 1 |
| ADRA1A | 1 | 0 | 0 | 0 | 0 | 0 | 0 | 0 | 1 |
| GLO1 | 1 | 0 | 0 | 0 | 0 | 0 | 0 | 0 | 1 |
| CDK11A | 1 | 0 | 0 | 0 | 0 | 0 | 0 | 0 | 1 |
| GPC6 | 1 | 0 | 0 | 0 | 0 | 0 | 0 | 0 | 1 |
| FGF6 | 1 | 0 | 0 | 0 | 0 | 0 | 0 | 0 | 1 |
| NPAT | 0 | 1 | 0 | 0 | 0 | 0 | 0 | 0 | 1 |
| IL1RAP | 1 | 0 | 0 | 0 | 0 | 0 | 0 | 0 | 1 |
| SMPDL3A | 1 | 0 | 0 | 0 | 0 | 0 | 0 | 0 | 1 |
| ARMC3 | 1 | 0 | 0 | 0 | 0 | 0 | 0 | 0 | 1 |
| PCDHGA10 | 1 | 0 | 0 | 0 | 0 | 0 | 0 | 0 | 1 |
| PKD2 | 1 | 0 | 0 | 0 | 0 | 0 | 0 | 0 | 1 |
| HLTF | 1 | 0 | 0 | 0 | 0 | 0 | 0 | 0 | 1 |
| ZNF7 | 1 | 0 | 0 | 0 | 0 | 0 | 0 | 0 | 1 |
| OSBP | 1 | 0 | 0 | 0 | 0 | 0 | 0 | 0 | 1 |
| LIPE | 1 | 0 | 0 | 0 | 0 | 0 | 0 | 0 | 1 |
| ZNF441 | 1 | 0 | 0 | 0 | 0 | 0 | 0 | 0 | 1 |
| FNDC3A | 1 | 0 | 0 | 0 | 0 | 0 | 0 | 0 | 1 |
| C21orf56 | 1 | 0 | 0 | 0 | 0 | 0 | 0 | 0 | 1 |
| ROBO4 | 1 | 0 | 0 | 0 | 0 | 0 | 0 | 0 | 1 |
| OR56B4 | 1 | 0 | 0 | 0 | 0 | 0 | 0 | 0 | 1 |
| SIGLEC5 | 1 | 0 | 0 | 0 | 0 | 0 | 0 | 0 | 1 |
| ZIC4 | 1 | 0 | 0 | 0 | 0 | 0 | 0 | 0 | 1 |

| | | | | | | | | | |
|---|---|---|---|---|---|---|---|---|---|
| OSBPL5 | 1 | 0 | 0 | 0 | 0 | 0 | 0 | 0 | 1 |
| SYDE1 | 1 | 0 | 0 | 0 | 0 | 0 | 0 | 0 | 1 |
| RPS6KA3 | 1 | 0 | 0 | 0 | 0 | 0 | 0 | 0 | 1 |
| PLCH2 | 1 | 0 | 0 | 0 | 0 | 0 | 0 | 0 | 1 |
| ZFX | 1 | 0 | 0 | 0 | 0 | 0 | 0 | 0 | 1 |
| TTC13 | 1 | 0 | 0 | 0 | 0 | 0 | 0 | 0 | 1 |
| CABP5 | 1 | 0 | 0 | 0 | 0 | 0 | 0 | 0 | 1 |
| RAPGEF5 | 1 | 0 | 0 | 0 | 0 | 0 | 0 | 0 | 1 |
| INO80D | 1 | 0 | 0 | 0 | 0 | 0 | 0 | 0 | 1 |
| ZBTB16 | 1 | 0 | 0 | 0 | 0 | 0 | 0 | 0 | 1 |
| ST8SIA1 | 1 | 0 | 0 | 0 | 0 | 0 | 0 | 0 | 1 |
| BRF1 | 1 | 0 | 0 | 0 | 0 | 0 | 0 | 0 | 1 |
| ZNF839 | 1 | 0 | 0 | 0 | 0 | 0 | 0 | 0 | 1 |
| DEFB115 | 1 | 0 | 0 | 0 | 0 | 0 | 0 | 0 | 1 |
| SPOCK3 | 1 | 0 | 0 | 0 | 0 | 0 | 0 | 0 | 1 |
| MC5R | 1 | 0 | 0 | 0 | 0 | 0 | 0 | 0 | 1 |
| DARS2 | 1 | 0 | 0 | 0 | 0 | 0 | 0 | 0 | 1 |
| ZMIZ1 | 1 | 0 | 0 | 0 | 0 | 0 | 0 | 0 | 1 |
| HAS2 | 1 | 0 | 0 | 0 | 0 | 0 | 0 | 0 | 1 |
| RGS6 | 1 | 0 | 0 | 0 | 0 | 0 | 0 | 0 | 1 |
| IL18RAP | 1 | 0 | 0 | 0 | 0 | 0 | 0 | 0 | 1 |
| CLIP3 | 1 | 0 | 0 | 0 | 0 | 0 | 0 | 0 | 1 |
| CDK5RAP1 | 1 | 0 | 0 | 0 | 0 | 0 | 0 | 0 | 1 |
| SH2D4B | 1 | 0 | 0 | 0 | 0 | 0 | 0 | 0 | 1 |
| KDM1A | 1 | 0 | 0 | 0 | 0 | 0 | 0 | 0 | 1 |
| PKP4 | 1 | 0 | 0 | 0 | 0 | 0 | 0 | 0 | 1 |
| LOXL4 | 1 | 0 | 0 | 0 | 0 | 0 | 0 | 0 | 1 |
| PFKL | 1 | 0 | 0 | 0 | 0 | 0 | 0 | 0 | 1 |
| GAB3 | 1 | 0 | 0 | 0 | 0 | 0 | 0 | 0 | 1 |
| PARK2 | 1 | 0 | 0 | 0 | 0 | 0 | 0 | 0 | 1 |
| LENG8 | 1 | 0 | 0 | 0 | 0 | 0 | 0 | 0 | 1 |
| LMLN | 1 | 0 | 0 | 0 | 0 | 0 | 0 | 0 | 1 |
| TRPV3 | 1 | 0 | 0 | 0 | 0 | 0 | 0 | 0 | 1 |
| FARP2 | 1 | 0 | 0 | 0 | 0 | 0 | 0 | 0 | 1 |
| PNPLA8 | 1 | 0 | 0 | 0 | 0 | 0 | 0 | 0 | 1 |
| SYBU | 1 | 0 | 0 | 0 | 0 | 0 | 0 | 0 | 1 |
| FURIN | 1 | 0 | 0 | 0 | 0 | 0 | 0 | 0 | 1 |
| ANKS6 | 1 | 0 | 0 | 0 | 0 | 0 | 0 | 0 | 1 |
| DSC1 | 1 | 0 | 0 | 0 | 0 | 0 | 0 | 0 | 1 |
| NEDD1 | 1 | 0 | 0 | 0 | 0 | 0 | 0 | 0 | 1 |
| RUNDC1 | 1 | 0 | 0 | 0 | 0 | 0 | 0 | 0 | 1 |
| ZFP62 | 1 | 0 | 0 | 0 | 0 | 0 | 0 | 0 | 1 |
| TBCD | 1 | 0 | 0 | 0 | 0 | 0 | 0 | 0 | 1 |
| ZNF711 | 1 | 0 | 0 | 0 | 0 | 0 | 0 | 0 | 1 |
| FRMD5 | 1 | 0 | 0 | 0 | 0 | 0 | 0 | 0 | 1 |
| SLC30A8 | 1 | 0 | 0 | 0 | 0 | 0 | 0 | 0 | 1 |

| | | | | | | | | | |
|---|---|---|---|---|---|---|---|---|---|
| PSMD2 | 1 | 0 | 0 | 0 | 0 | 0 | 0 | 0 | 1 |
| DPYS | 1 | 0 | 0 | 0 | 0 | 0 | 0 | 0 | 1 |
| ETV3L | 1 | 0 | 0 | 0 | 0 | 0 | 0 | 0 | 1 |
| KDM4C | 1 | 0 | 0 | 0 | 0 | 0 | 0 | 0 | 1 |
| ZNF775 | 1 | 0 | 0 | 0 | 0 | 0 | 0 | 0 | 1 |
| ADAM15 | 1 | 0 | 0 | 0 | 0 | 0 | 0 | 0 | 1 |
| C12orf26 | 1 | 0 | 0 | 0 | 0 | 0 | 0 | 0 | 1 |
| NUP93 | 1 | 0 | 0 | 0 | 0 | 0 | 0 | 0 | 1 |
| ZNF792 | 1 | 0 | 0 | 0 | 0 | 0 | 0 | 0 | 1 |
| SMC6 | 1 | 0 | 0 | 0 | 0 | 0 | 0 | 0 | 1 |
| ZNF628 | 1 | 0 | 0 | 0 | 0 | 0 | 0 | 0 | 1 |
| HSPH1 | 1 | 0 | 0 | 0 | 0 | 0 | 0 | 0 | 1 |
| ZNF408 | 1 | 0 | 0 | 0 | 0 | 0 | 0 | 0 | 1 |
| RNF19B | 1 | 0 | 0 | 0 | 0 | 0 | 0 | 0 | 1 |
| ZC3H12B | 1 | 0 | 0 | 0 | 0 | 0 | 0 | 0 | 1 |
| ARMCX5 | 1 | 0 | 0 | 0 | 0 | 0 | 0 | 0 | 1 |
| AVL9 | 1 | 0 | 0 | 0 | 0 | 0 | 0 | 0 | 1 |
| PARP9 | 1 | 0 | 0 | 0 | 0 | 0 | 0 | 0 | 1 |
| OR51D1 | 1 | 0 | 0 | 0 | 0 | 0 | 0 | 0 | 1 |
| PLXDC2 | 1 | 0 | 0 | 0 | 0 | 0 | 0 | 0 | 1 |
| ZNF317 | 1 | 0 | 0 | 0 | 0 | 0 | 0 | 0 | 1 |
| SLC34A2 | 1 | 0 | 0 | 0 | 0 | 0 | 0 | 0 | 1 |
| ADRBK2 | 1 | 0 | 0 | 0 | 0 | 0 | 0 | 0 | 1 |
| FHDC1 | 1 | 0 | 0 | 0 | 0 | 0 | 0 | 0 | 1 |
| NPAS4 | 1 | 0 | 0 | 0 | 0 | 0 | 0 | 0 | 1 |
| OR2T1 | 0 | 1 | 0 | 0 | 0 | 0 | 0 | 0 | 1 |
| APBA1 | 1 | 0 | 0 | 0 | 0 | 0 | 0 | 0 | 1 |
| LIMK1 | 1 | 0 | 0 | 0 | 0 | 0 | 0 | 0 | 1 |
| MBTPS1 | 1 | 0 | 0 | 0 | 0 | 0 | 0 | 0 | 1 |
| BAHD1 | 1 | 0 | 0 | 0 | 0 | 0 | 0 | 0 | 1 |
| NOX3 | 0 | 1 | 0 | 0 | 0 | 0 | 0 | 0 | 1 |
| SNX19 | 1 | 0 | 0 | 0 | 0 | 0 | 0 | 0 | 1 |
| TRMT1 | 1 | 0 | 0 | 0 | 0 | 0 | 0 | 0 | 1 |
| CSGALNACT2 | 1 | 0 | 0 | 0 | 0 | 0 | 0 | 0 | 1 |
| CAND2 | 1 | 0 | 0 | 0 | 0 | 0 | 0 | 0 | 1 |
| IFIT1B | 1 | 0 | 0 | 0 | 0 | 0 | 0 | 0 | 1 |
| TYW3 | 1 | 0 | 0 | 0 | 0 | 0 | 0 | 0 | 1 |
| CCDC105 | 1 | 0 | 0 | 0 | 0 | 0 | 0 | 0 | 1 |
| TRIM24 | 1 | 0 | 0 | 0 | 0 | 0 | 0 | 0 | 1 |
| IL10RB | 1 | 0 | 0 | 0 | 0 | 0 | 0 | 0 | 1 |
| NEK10 | 1 | 0 | 0 | 0 | 0 | 0 | 0 | 0 | 1 |
| NEFM | 1 | 0 | 0 | 0 | 0 | 0 | 0 | 0 | 1 |
| C11orf35 | 1 | 0 | 0 | 0 | 0 | 0 | 0 | 0 | 1 |
| TNKS2 | 1 | 0 | 0 | 0 | 0 | 0 | 0 | 0 | 1 |
| IFIT1 | 1 | 0 | 0 | 0 | 0 | 0 | 0 | 0 | 1 |
| IL12RB1 | 1 | 0 | 0 | 0 | 0 | 0 | 0 | 0 | 1 |

| | | | | | | | | | |
|---|---|---|---|---|---|---|---|---|---|
| PRSS36 | 1 | 0 | 0 | 0 | 0 | 0 | 0 | 0 | 1 |
| CAPN3 | 1 | 0 | 0 | 0 | 0 | 0 | 0 | 0 | 1 |
| TKTL2 | 1 | 0 | 0 | 0 | 0 | 0 | 0 | 0 | 1 |
| ADAM23 | 1 | 0 | 0 | 0 | 0 | 0 | 0 | 0 | 1 |
| LRP5 | 0 | 0 | 0 | 0 | 1 | 0 | 0 | 0 | 1 |
| SUV420H2 | 1 | 0 | 0 | 0 | 0 | 0 | 0 | 0 | 1 |
| TOM1 | 1 | 0 | 0 | 0 | 0 | 0 | 0 | 0 | 1 |
| PIGS | 1 | 0 | 0 | 0 | 0 | 0 | 0 | 0 | 1 |
| MAX | 1 | 0 | 0 | 0 | 0 | 0 | 0 | 0 | 1 |
| LUZP1 | 1 | 0 | 0 | 0 | 0 | 0 | 0 | 0 | 1 |
| SRGAP2 | 1 | 0 | 0 | 0 | 0 | 0 | 0 | 0 | 1 |
| MUM1 | 1 | 0 | 0 | 0 | 0 | 0 | 0 | 0 | 1 |
| DUSP4 | 1 | 0 | 0 | 0 | 0 | 0 | 0 | 0 | 1 |
| PLD2 | 1 | 0 | 0 | 0 | 0 | 0 | 0 | 0 | 1 |
| WDR59 | 1 | 0 | 0 | 0 | 0 | 0 | 0 | 0 | 1 |
| EMR2 | 1 | 0 | 0 | 0 | 0 | 0 | 0 | 0 | 1 |
| IL17RB | 1 | 0 | 0 | 0 | 0 | 0 | 0 | 0 | 1 |
| DDX42 | 1 | 0 | 0 | 0 | 0 | 0 | 0 | 0 | 1 |
| OR1A1 | 1 | 0 | 0 | 0 | 0 | 0 | 0 | 0 | 1 |
| KCNH2 | 1 | 0 | 0 | 0 | 0 | 0 | 0 | 0 | 1 |
| ZNF266 | 1 | 0 | 0 | 0 | 0 | 0 | 0 | 0 | 1 |
| TBX22 | 1 | 0 | 0 | 0 | 0 | 0 | 0 | 0 | 1 |
| SAMD11 | 1 | 0 | 0 | 0 | 0 | 0 | 0 | 0 | 1 |
| PPP1R12A | 1 | 0 | 0 | 0 | 0 | 0 | 0 | 0 | 1 |
| SF1 | 1 | 0 | 0 | 0 | 0 | 0 | 0 | 0 | 1 |
| PPAPDC1A | 1 | 0 | 0 | 0 | 0 | 0 | 0 | 0 | 1 |
| JMJD7-PLA2G4B | 1 | 0 | 0 | 0 | 0 | 0 | 0 | 0 | 1 |
| BBS9 | 1 | 0 | 0 | 0 | 0 | 0 | 0 | 0 | 1 |
| CD300A | 1 | 0 | 0 | 0 | 0 | 0 | 0 | 0 | 1 |
| ESRP1 | 1 | 0 | 0 | 0 | 0 | 0 | 0 | 0 | 1 |
| PCDHB2 | 1 | 0 | 0 | 0 | 0 | 0 | 0 | 0 | 1 |
| HS6ST3 | 1 | 0 | 0 | 0 | 0 | 0 | 0 | 0 | 1 |
| E2F8 | 1 | 0 | 0 | 0 | 0 | 0 | 0 | 0 | 1 |
| TRPV4 | 1 | 0 | 0 | 0 | 0 | 0 | 0 | 0 | 1 |
| ZNF44 | 1 | 0 | 0 | 0 | 0 | 0 | 0 | 0 | 1 |
| TOP1 | 1 | 0 | 0 | 0 | 0 | 0 | 0 | 0 | 1 |
| STXBP4 | 1 | 0 | 0 | 0 | 0 | 0 | 0 | 0 | 1 |
| ZNF709 | 1 | 0 | 0 | 0 | 0 | 0 | 0 | 0 | 1 |
| ERCC4 | 1 | 0 | 0 | 0 | 0 | 0 | 0 | 0 | 1 |
| IL7R | 1 | 0 | 0 | 0 | 0 | 0 | 0 | 0 | 1 |
| POPDC2 | 1 | 0 | 0 | 0 | 0 | 0 | 0 | 0 | 1 |
| SSH1 | 1 | 0 | 0 | 0 | 0 | 0 | 0 | 0 | 1 |
| TTC16 | 1 | 0 | 0 | 0 | 0 | 0 | 0 | 0 | 1 |
| ADAM12 | 1 | 0 | 0 | 0 | 0 | 0 | 0 | 0 | 1 |
| SEZ6 | 1 | 0 | 0 | 0 | 0 | 0 | 0 | 0 | 1 |
| BRPF3 | 1 | 0 | 0 | 0 | 0 | 0 | 0 | 0 | 1 |

| | | | | | | | | | |
|---|---|---|---|---|---|---|---|---|---|
| GALNT8 | 1 | 0 | 0 | 0 | 0 | 0 | 0 | 0 | 1 |
| LEPRE1 | 1 | 0 | 0 | 0 | 0 | 0 | 0 | 0 | 1 |
| CCKAR | 1 | 0 | 0 | 0 | 0 | 0 | 0 | 0 | 1 |
| OTOL1 | 1 | 0 | 0 | 0 | 0 | 0 | 0 | 0 | 1 |
| CABP4 | 1 | 0 | 0 | 0 | 0 | 0 | 0 | 0 | 1 |
| LRRC8A | 1 | 0 | 0 | 0 | 0 | 0 | 0 | 0 | 1 |
| KIF17 | 1 | 0 | 0 | 0 | 0 | 0 | 0 | 0 | 1 |
| IQUB | 1 | 0 | 0 | 0 | 0 | 0 | 0 | 0 | 1 |
| ADAM33 | 1 | 0 | 0 | 0 | 0 | 0 | 0 | 0 | 1 |
| SIM1 | 1 | 0 | 0 | 0 | 0 | 0 | 0 | 0 | 1 |
| NFATC3 | 1 | 0 | 0 | 0 | 0 | 0 | 0 | 0 | 1 |
| ZNF175 | 1 | 0 | 0 | 0 | 0 | 0 | 0 | 0 | 1 |
| DLL1 | 1 | 0 | 0 | 0 | 0 | 0 | 0 | 0 | 1 |
| EPC1 | 1 | 0 | 0 | 0 | 0 | 0 | 0 | 0 | 1 |
| HABP2 | 1 | 0 | 0 | 0 | 0 | 0 | 0 | 0 | 1 |
| EDEM3 | 1 | 0 | 0 | 0 | 0 | 0 | 0 | 0 | 1 |
| PCDHGB4 | 1 | 0 | 0 | 0 | 0 | 0 | 0 | 0 | 1 |
| ABCC5 | 1 | 0 | 0 | 0 | 0 | 0 | 0 | 0 | 1 |
| GAB1 | 0 | 1 | 0 | 0 | 0 | 0 | 0 | 0 | 1 |
| ATMIN | 1 | 0 | 0 | 0 | 0 | 0 | 0 | 0 | 1 |
| GCC1 | 1 | 0 | 0 | 0 | 0 | 0 | 0 | 0 | 1 |
| ZNF699 | 1 | 0 | 0 | 0 | 0 | 0 | 0 | 0 | 1 |
| CCDC66 | 1 | 0 | 0 | 0 | 0 | 0 | 0 | 0 | 1 |
| KIF11 | 1 | 0 | 0 | 0 | 0 | 0 | 0 | 0 | 1 |
| ZFYVE28 | 1 | 0 | 0 | 0 | 0 | 0 | 0 | 0 | 1 |
| SLC6A18 | 1 | 0 | 0 | 0 | 0 | 0 | 0 | 0 | 1 |
| ATXN10 | 1 | 0 | 0 | 0 | 0 | 0 | 0 | 0 | 1 |
| MFGE8 | 1 | 0 | 0 | 0 | 0 | 0 | 0 | 0 | 1 |
| ZNF17 | 1 | 0 | 0 | 0 | 0 | 0 | 0 | 0 | 1 |
| CD1A | 1 | 0 | 0 | 0 | 0 | 0 | 0 | 0 | 1 |
| ELN | 1 | 0 | 0 | 0 | 0 | 0 | 0 | 0 | 1 |
| PNMA3 | 1 | 0 | 0 | 0 | 0 | 0 | 0 | 0 | 1 |
| ZPBP2 | 1 | 0 | 0 | 0 | 0 | 0 | 0 | 0 | 1 |
| TBC1D8 | 1 | 0 | 0 | 0 | 0 | 0 | 0 | 0 | 1 |
| ZNF280D | 1 | 0 | 0 | 0 | 0 | 0 | 0 | 0 | 1 |
| TARSL2 | 1 | 0 | 0 | 0 | 0 | 0 | 0 | 0 | 1 |
| SOX9 | 1 | 0 | 0 | 0 | 0 | 0 | 0 | 0 | 1 |
| FASTKD3 | 1 | 0 | 0 | 0 | 0 | 0 | 0 | 0 | 1 |
| KDM3A | 1 | 0 | 0 | 0 | 0 | 0 | 0 | 0 | 1 |
| MVP | 1 | 0 | 0 | 0 | 0 | 0 | 0 | 0 | 1 |
| PPIP5K1 | 1 | 0 | 0 | 0 | 0 | 0 | 0 | 0 | 1 |
| OR4D10 | 1 | 0 | 0 | 0 | 0 | 0 | 0 | 0 | 1 |
| MAN2A2 | 1 | 0 | 0 | 0 | 0 | 0 | 0 | 0 | 1 |
| EIF3B | 1 | 0 | 0 | 0 | 0 | 0 | 0 | 0 | 1 |
| EEA1 | 1 | 0 | 0 | 0 | 0 | 0 | 0 | 0 | 1 |
| TAS2R8 | 1 | 0 | 0 | 0 | 0 | 0 | 0 | 0 | 1 |

| | | | | | | | | | |
|---|---|---|---|---|---|---|---|---|---|
| ABCC4 | 1 | 0 | 0 | 0 | 0 | 0 | 0 | 0 | 1 |
| HDAC7 | 1 | 0 | 0 | 0 | 0 | 0 | 0 | 0 | 1 |
| TSR1 | 1 | 0 | 0 | 0 | 0 | 0 | 0 | 0 | 1 |
| SCARF2 | 1 | 0 | 0 | 0 | 0 | 0 | 0 | 0 | 1 |
| WAPAL | 1 | 0 | 0 | 0 | 0 | 0 | 0 | 0 | 1 |
| GRN | 1 | 0 | 0 | 0 | 0 | 0 | 0 | 0 | 1 |
| ESYT1 | 1 | 0 | 0 | 0 | 0 | 0 | 0 | 0 | 1 |
| GLIS1 | 1 | 0 | 0 | 0 | 0 | 0 | 0 | 0 | 1 |
| CSF2RB | 1 | 0 | 0 | 0 | 0 | 0 | 0 | 0 | 1 |
| KCND3 | 1 | 0 | 0 | 0 | 0 | 0 | 0 | 0 | 1 |
| USP15 | 1 | 0 | 0 | 0 | 0 | 0 | 0 | 0 | 1 |
| UBQLN3 | 1 | 0 | 0 | 0 | 0 | 0 | 0 | 0 | 1 |
| STAG1 | 1 | 0 | 0 | 0 | 0 | 0 | 0 | 0 | 1 |
| TTC21A | 1 | 0 | 0 | 0 | 0 | 0 | 0 | 0 | 1 |
| GPNMB | 1 | 0 | 0 | 0 | 0 | 0 | 0 | 0 | 1 |
| LRRN1 | 1 | 0 | 0 | 0 | 0 | 0 | 0 | 0 | 1 |
| MFSD1 | 1 | 0 | 0 | 0 | 0 | 0 | 0 | 0 | 1 |
| CRYGS | 1 | 0 | 0 | 0 | 0 | 0 | 0 | 0 | 1 |
| SEMG2 | 1 | 0 | 0 | 0 | 0 | 0 | 0 | 0 | 1 |
| EDC4 | 1 | 0 | 0 | 0 | 0 | 0 | 0 | 0 | 1 |
| VAV2 | 1 | 0 | 0 | 0 | 0 | 0 | 0 | 0 | 1 |
| AFAP1L1 | 1 | 0 | 0 | 0 | 0 | 0 | 0 | 0 | 1 |
| SGTA | 1 | 0 | 0 | 0 | 0 | 0 | 0 | 0 | 1 |
| CRMP1 | 1 | 0 | 0 | 0 | 0 | 0 | 0 | 0 | 1 |
| MYBBP1A | 1 | 0 | 0 | 0 | 0 | 0 | 0 | 0 | 1 |
| BEST1 | 1 | 0 | 0 | 0 | 0 | 0 | 0 | 0 | 1 |
| IRAK4 | 1 | 0 | 0 | 0 | 0 | 0 | 0 | 0 | 1 |
| NR5A2 | 1 | 0 | 0 | 0 | 0 | 0 | 0 | 0 | 1 |
| AR | 1 | 0 | 0 | 0 | 0 | 0 | 0 | 0 | 1 |
| HOOK1 | 1 | 0 | 0 | 0 | 0 | 0 | 0 | 0 | 1 |
| ATG9B | 1 | 0 | 0 | 0 | 0 | 0 | 0 | 0 | 1 |
| C12orf43 | 1 | 0 | 0 | 0 | 0 | 0 | 0 | 0 | 1 |
| ERCC6L | 1 | 0 | 0 | 0 | 0 | 0 | 0 | 0 | 1 |
| PDE5A | 1 | 0 | 0 | 0 | 0 | 0 | 0 | 0 | 1 |
| EXOSC2 | 1 | 0 | 0 | 0 | 0 | 0 | 0 | 0 | 1 |
| TYSND1 | 1 | 0 | 0 | 0 | 0 | 0 | 0 | 0 | 1 |
| RAPH1 | 1 | 0 | 0 | 0 | 0 | 0 | 0 | 0 | 1 |
| HERC4 | 1 | 0 | 0 | 0 | 0 | 0 | 0 | 0 | 1 |
| IRGC | 1 | 0 | 0 | 0 | 0 | 0 | 0 | 0 | 1 |
| BARD1 | 1 | 0 | 0 | 0 | 0 | 0 | 0 | 0 | 1 |
| RNF111 | 1 | 0 | 0 | 0 | 0 | 0 | 0 | 0 | 1 |
| HTATSF1 | 1 | 0 | 0 | 0 | 0 | 0 | 0 | 0 | 1 |
| SMARCAD1 | 1 | 0 | 0 | 0 | 0 | 0 | 0 | 0 | 1 |
| BCL11A | 1 | 0 | 0 | 0 | 0 | 0 | 0 | 0 | 1 |
| ADCY9 | 1 | 0 | 0 | 0 | 0 | 0 | 0 | 0 | 1 |
| PNPLA6 | 1 | 0 | 0 | 0 | 0 | 0 | 0 | 0 | 1 |

| | | | | | | | | | |
|---|---|---|---|---|---|---|---|---|---|
| COBLL1 | 1 | 0 | 0 | 0 | 0 | 0 | 0 | 0 | 1 |
| NCAN | 0 | 0 | 0 | 0 | 1 | 0 | 0 | 0 | 1 |
| DPYSL3 | 1 | 0 | 0 | 0 | 0 | 0 | 0 | 0 | 1 |
| C2orf78 | 1 | 0 | 0 | 0 | 0 | 0 | 0 | 0 | 1 |
| PON1 | 1 | 0 | 0 | 0 | 0 | 0 | 0 | 0 | 1 |
| GIN1 | 1 | 0 | 0 | 0 | 0 | 0 | 0 | 0 | 1 |
| COL8A2 | 1 | 0 | 0 | 0 | 0 | 0 | 0 | 0 | 1 |
| FARP1 | 1 | 0 | 0 | 0 | 0 | 0 | 0 | 0 | 1 |
| NRG2 | 1 | 0 | 0 | 0 | 0 | 0 | 0 | 0 | 1 |
| SGK223 | 1 | 0 | 0 | 0 | 0 | 0 | 0 | 0 | 1 |
| FBF1 | 1 | 0 | 0 | 0 | 0 | 0 | 0 | 0 | 1 |
| GEN1 | 1 | 0 | 0 | 0 | 0 | 0 | 0 | 0 | 1 |
| RASAL3 | 1 | 0 | 0 | 0 | 0 | 0 | 0 | 0 | 1 |
| CEACAM20 | 1 | 0 | 0 | 0 | 0 | 0 | 0 | 0 | 1 |
| GFM1 | 1 | 0 | 0 | 0 | 0 | 0 | 0 | 0 | 1 |
| TRIM32 | 1 | 0 | 0 | 0 | 0 | 0 | 0 | 0 | 1 |
| LIG1 | 1 | 0 | 0 | 0 | 0 | 0 | 0 | 0 | 1 |
| OR9G4 | 1 | 0 | 0 | 0 | 0 | 0 | 0 | 0 | 1 |
| NELL2 | 1 | 0 | 0 | 0 | 0 | 0 | 0 | 0 | 1 |
| ZNF304 | 1 | 0 | 0 | 0 | 0 | 0 | 0 | 0 | 1 |
| PDE2A | 1 | 0 | 0 | 0 | 0 | 0 | 0 | 0 | 1 |
| OR1A2 | 1 | 0 | 0 | 0 | 0 | 0 | 0 | 0 | 1 |
| CHD1L | 1 | 0 | 0 | 0 | 0 | 0 | 0 | 0 | 1 |
| IFNAR1 | 0 | 1 | 0 | 0 | 0 | 0 | 0 | 0 | 1 |
| LIG3 | 0 | 1 | 0 | 0 | 0 | 0 | 0 | 0 | 1 |
| CTNNB1 | 1 | 0 | 0 | 0 | 0 | 0 | 0 | 0 | 1 |
| ZNF225 | 1 | 0 | 0 | 0 | 0 | 0 | 0 | 0 | 1 |
| CCDC92 | 1 | 0 | 0 | 0 | 0 | 0 | 0 | 0 | 1 |
| ZNF708 | 1 | 0 | 0 | 0 | 0 | 0 | 0 | 0 | 1 |
| ZNF415 | 1 | 0 | 0 | 0 | 0 | 0 | 0 | 0 | 1 |
| TTC17 | 1 | 0 | 0 | 0 | 0 | 0 | 0 | 0 | 1 |
| SLCO3A1 | 1 | 0 | 0 | 0 | 0 | 0 | 0 | 0 | 1 |
| ANKLE2 | 1 | 0 | 0 | 0 | 0 | 0 | 0 | 0 | 1 |
| F12 | 1 | 0 | 0 | 0 | 0 | 0 | 0 | 0 | 1 |
| TSSK1B | 1 | 0 | 0 | 0 | 0 | 0 | 0 | 0 | 1 |
| RECQL | 1 | 0 | 0 | 0 | 0 | 0 | 0 | 0 | 1 |
| TLL2 | 1 | 0 | 0 | 0 | 0 | 0 | 0 | 0 | 1 |
| CASC3 | 1 | 0 | 0 | 0 | 0 | 0 | 0 | 0 | 1 |
| ESRP2 | 1 | 0 | 0 | 0 | 0 | 0 | 0 | 0 | 1 |
| NOD2 | 1 | 0 | 0 | 0 | 0 | 0 | 0 | 0 | 1 |
| N4BP2L2 | 1 | 0 | 0 | 0 | 0 | 0 | 0 | 0 | 1 |
| SLAMF6 | 1 | 0 | 0 | 0 | 0 | 0 | 0 | 0 | 1 |
| ZFP91 | 1 | 0 | 0 | 0 | 0 | 0 | 0 | 0 | 1 |
| RECQL4 | 1 | 0 | 0 | 0 | 0 | 0 | 0 | 0 | 1 |
| DLGAP5 | 1 | 0 | 0 | 0 | 0 | 0 | 0 | 0 | 1 |
| OTUD7A | 1 | 0 | 0 | 0 | 0 | 0 | 0 | 0 | 1 |

| | | | | | | | | | |
|---|---|---|---|---|---|---|---|---|---|
| FAM160A1 | 1 | 0 | 0 | 0 | 0 | 0 | 0 | 0 | 1 |
| PCDH12 | 1 | 0 | 0 | 0 | 0 | 0 | 0 | 0 | 1 |
| CNGB1 | 1 | 0 | 0 | 0 | 0 | 0 | 0 | 0 | 1 |
| FMO4 | 1 | 0 | 0 | 0 | 0 | 0 | 0 | 0 | 1 |
| PNMA2 | 1 | 0 | 0 | 0 | 0 | 0 | 0 | 0 | 1 |
| UBN1 | 1 | 0 | 0 | 0 | 0 | 0 | 0 | 0 | 1 |
| SH3BP4 | 1 | 0 | 0 | 0 | 0 | 0 | 0 | 0 | 1 |
| ZNF471 | 1 | 0 | 0 | 0 | 0 | 0 | 0 | 0 | 1 |
| PSMC1 | 1 | 0 | 0 | 0 | 0 | 0 | 0 | 0 | 1 |
| TEX10 | 1 | 0 | 0 | 0 | 0 | 0 | 0 | 0 | 1 |
| HBS1L | 1 | 0 | 0 | 0 | 0 | 0 | 0 | 0 | 1 |
| GJA10 | 1 | 0 | 0 | 0 | 0 | 0 | 0 | 0 | 1 |
| AGBL2 | 1 | 0 | 0 | 0 | 0 | 0 | 0 | 0 | 1 |
| RAB11FIP4 | 1 | 0 | 0 | 0 | 0 | 0 | 0 | 0 | 1 |
| PWP2 | 0 | 0 | 1 | 0 | 0 | 0 | 0 | 0 | 1 |
| MARS2 | 1 | 0 | 0 | 0 | 0 | 0 | 0 | 0 | 1 |
| FAM13C | 1 | 0 | 0 | 0 | 0 | 0 | 0 | 0 | 1 |
| TCOF1 | 1 | 0 | 0 | 0 | 0 | 0 | 0 | 0 | 1 |
| IFIH1 | 1 | 0 | 0 | 0 | 0 | 0 | 0 | 0 | 1 |
| PTPDC1 | 1 | 0 | 0 | 0 | 0 | 0 | 0 | 0 | 1 |
| RIN3 | 1 | 0 | 0 | 0 | 0 | 0 | 0 | 0 | 1 |
| KANK1 | 1 | 0 | 0 | 0 | 0 | 0 | 0 | 0 | 1 |
| COL10A1 | 1 | 0 | 0 | 0 | 0 | 0 | 0 | 0 | 1 |
| C6orf62 | 1 | 0 | 0 | 0 | 0 | 0 | 0 | 0 | 1 |
| C3orf27 | 1 | 0 | 0 | 0 | 0 | 0 | 0 | 0 | 1 |
| ZBTB37 | 1 | 0 | 0 | 0 | 0 | 0 | 0 | 0 | 1 |
| ZSCAN18 | 1 | 0 | 0 | 0 | 0 | 0 | 0 | 0 | 1 |
| COL6A2 | 1 | 0 | 0 | 0 | 0 | 0 | 0 | 0 | 1 |
| DSC2 | 1 | 0 | 0 | 0 | 0 | 0 | 0 | 0 | 1 |
| UBE3A | 1 | 0 | 0 | 0 | 0 | 0 | 0 | 0 | 1 |
| NOC2L | 1 | 0 | 0 | 0 | 0 | 0 | 0 | 0 | 1 |
| IMMT | 1 | 0 | 0 | 0 | 0 | 0 | 0 | 0 | 1 |
| TNRC6C | 1 | 0 | 0 | 0 | 0 | 0 | 0 | 0 | 1 |
| ZKSCAN1 | 1 | 0 | 0 | 0 | 0 | 0 | 0 | 0 | 1 |
| XPO5 | 1 | 0 | 0 | 0 | 0 | 0 | 0 | 0 | 1 |
| GPR133 | 1 | 0 | 0 | 0 | 0 | 0 | 0 | 0 | 1 |
| OR2S2 | 1 | 0 | 0 | 0 | 0 | 0 | 0 | 0 | 1 |
| ARID5B | 1 | 0 | 0 | 0 | 0 | 0 | 0 | 0 | 1 |
| ZNF543 | 1 | 0 | 0 | 0 | 0 | 0 | 0 | 0 | 1 |
| ATP11A | 1 | 0 | 0 | 0 | 0 | 0 | 0 | 0 | 1 |
| PLB1 | 0 | 0 | 1 | 0 | 0 | 0 | 0 | 0 | 1 |
| ZNF585B | 1 | 0 | 0 | 0 | 0 | 0 | 0 | 0 | 1 |
| BACE2 | 1 | 0 | 0 | 0 | 0 | 0 | 0 | 0 | 1 |
| IQCA1 | 1 | 0 | 0 | 0 | 0 | 0 | 0 | 0 | 1 |
| ZNF544 | 1 | 0 | 0 | 0 | 0 | 0 | 0 | 0 | 1 |
| KIAA0355 | 1 | 0 | 0 | 0 | 0 | 0 | 0 | 0 | 1 |

| | | | | | | | | | |
|---|---|---|---|---|---|---|---|---|---|
| ATXN7 | 1 | 0 | 0 | 0 | 0 | 0 | 0 | 0 | 1 |
| ALDH7A1 | 1 | 0 | 0 | 0 | 0 | 0 | 0 | 0 | 1 |
| MAP3K5 | 1 | 0 | 0 | 0 | 0 | 0 | 0 | 0 | 1 |
| TEX11 | 1 | 0 | 0 | 0 | 0 | 0 | 0 | 0 | 1 |
| TAF3 | 1 | 0 | 0 | 0 | 0 | 0 | 0 | 0 | 1 |
| PCK2 | 1 | 0 | 0 | 0 | 0 | 0 | 0 | 0 | 1 |
| TBC1D9 | 1 | 0 | 0 | 0 | 0 | 0 | 0 | 0 | 1 |
| C2orf44 | 1 | 0 | 0 | 0 | 0 | 0 | 0 | 0 | 1 |
| ATP2B1 | 1 | 0 | 0 | 0 | 0 | 0 | 0 | 0 | 1 |
| VWA5B2 | 1 | 0 | 0 | 0 | 0 | 0 | 0 | 0 | 1 |
| BATF | 1 | 0 | 0 | 0 | 0 | 0 | 0 | 0 | 1 |
| DNM2 | 1 | 0 | 0 | 0 | 0 | 0 | 0 | 0 | 1 |
| FBXO9 | 1 | 0 | 0 | 0 | 0 | 0 | 0 | 0 | 1 |
| RBP3 | 1 | 0 | 0 | 0 | 0 | 0 | 0 | 0 | 1 |
| RNF40 | 1 | 0 | 0 | 0 | 0 | 0 | 0 | 0 | 1 |
| ACSS3 | 1 | 0 | 0 | 0 | 0 | 0 | 0 | 0 | 1 |
| NCKAP1 | 1 | 0 | 0 | 0 | 0 | 0 | 0 | 0 | 1 |
| THOC1 | 1 | 0 | 0 | 0 | 0 | 0 | 0 | 0 | 1 |
| ZNF229 | 1 | 0 | 0 | 0 | 0 | 0 | 0 | 0 | 1 |
| RPAP1 | 1 | 0 | 0 | 0 | 0 | 0 | 0 | 0 | 1 |
| WDR47 | 1 | 0 | 0 | 0 | 0 | 0 | 0 | 0 | 1 |
| HESX1 | 1 | 0 | 0 | 0 | 0 | 0 | 0 | 0 | 1 |
| SPATA13 | 1 | 0 | 0 | 0 | 0 | 0 | 0 | 0 | 1 |
| PAQR6 | 1 | 0 | 0 | 0 | 0 | 0 | 0 | 0 | 1 |
| SCYL2 | 1 | 0 | 0 | 0 | 0 | 0 | 0 | 0 | 1 |
| TCERG1L | 1 | 0 | 0 | 0 | 0 | 0 | 0 | 0 | 1 |
| S100Z | 1 | 0 | 0 | 0 | 0 | 0 | 0 | 0 | 1 |
| GRIP1 | 1 | 0 | 0 | 0 | 0 | 0 | 0 | 0 | 1 |
| ZBTB10 | 1 | 0 | 0 | 0 | 0 | 0 | 0 | 0 | 1 |
| TTC21B | 1 | 0 | 0 | 0 | 0 | 0 | 0 | 0 | 1 |
| GSDMB | 1 | 0 | 0 | 0 | 0 | 0 | 0 | 0 | 1 |
| ZRANB1 | 1 | 0 | 0 | 0 | 0 | 0 | 0 | 0 | 1 |
| DNMT3A | 1 | 0 | 0 | 0 | 0 | 0 | 0 | 0 | 1 |
| HSPA12B | 1 | 0 | 0 | 0 | 0 | 0 | 0 | 0 | 1 |
| CAPN1 | 1 | 0 | 0 | 0 | 0 | 0 | 0 | 0 | 1 |
| BPI | 1 | 0 | 0 | 0 | 0 | 0 | 0 | 0 | 1 |
| CYFIP1 | 1 | 0 | 0 | 0 | 0 | 0 | 0 | 0 | 1 |
| ATRN | 1 | 0 | 0 | 0 | 0 | 0 | 0 | 0 | 1 |
| PLXNA1 | 1 | 0 | 0 | 0 | 0 | 0 | 0 | 0 | 1 |
| GPD2 | 1 | 0 | 0 | 0 | 0 | 0 | 0 | 0 | 1 |
| GFPT1 | 0 | 1 | 0 | 0 | 0 | 0 | 0 | 0 | 1 |
| KIAA1324 | 1 | 0 | 0 | 0 | 0 | 0 | 0 | 0 | 1 |
| INSRR | 1 | 0 | 0 | 0 | 0 | 0 | 0 | 0 | 1 |
| OR4C16 | 1 | 0 | 0 | 0 | 0 | 0 | 0 | 0 | 1 |
| TXNDC16 | 0 | 0 | 1 | 0 | 0 | 0 | 0 | 0 | 1 |
| EVC2 | 1 | 0 | 0 | 0 | 0 | 0 | 0 | 0 | 1 |

| | | | | | | | | | |
|---|---|---|---|---|---|---|---|---|---|
| ZCCHC12 | 1 | 0 | 0 | 0 | 0 | 0 | 0 | 0 | 1 |
| XPO1 | 1 | 0 | 0 | 0 | 0 | 0 | 0 | 0 | 1 |
| OR52N4 | 1 | 0 | 0 | 0 | 0 | 0 | 0 | 0 | 1 |
| DHX8 | 1 | 0 | 0 | 0 | 0 | 0 | 0 | 0 | 1 |
| VWA5A | 1 | 0 | 0 | 0 | 0 | 0 | 0 | 0 | 1 |
| KIAA0182 | 1 | 0 | 0 | 0 | 0 | 0 | 0 | 0 | 1 |
| GUCY1A2 | 1 | 0 | 0 | 0 | 0 | 0 | 0 | 0 | 1 |
| ACAP2 | 1 | 0 | 0 | 0 | 0 | 0 | 0 | 0 | 1 |
| UNC45B | 1 | 0 | 0 | 0 | 0 | 0 | 0 | 0 | 1 |
| FAM178A | 1 | 0 | 0 | 0 | 0 | 0 | 0 | 0 | 1 |
| HIP1R | 1 | 0 | 0 | 0 | 0 | 0 | 0 | 0 | 1 |
| ACVR1B | 1 | 0 | 0 | 0 | 0 | 0 | 0 | 0 | 1 |
| GFM2 | 1 | 0 | 0 | 0 | 0 | 0 | 0 | 0 | 1 |
| CCDC150 | 1 | 0 | 0 | 0 | 0 | 0 | 0 | 0 | 1 |
| ZHX3 | 1 | 0 | 0 | 0 | 0 | 0 | 0 | 0 | 1 |
| SALL2 | 1 | 0 | 0 | 0 | 0 | 0 | 0 | 0 | 1 |
| SERINC4 | 1 | 0 | 0 | 0 | 0 | 0 | 0 | 0 | 1 |
| ADNP2 | 1 | 0 | 0 | 0 | 0 | 0 | 0 | 0 | 1 |
| PLSCR5 | 1 | 0 | 0 | 0 | 0 | 0 | 0 | 0 | 1 |
| PIK3CD | 1 | 0 | 0 | 0 | 0 | 0 | 0 | 0 | 1 |
| NFATC1 | 1 | 0 | 0 | 0 | 0 | 0 | 0 | 0 | 1 |
| PRSS55 | 1 | 0 | 0 | 0 | 0 | 0 | 0 | 0 | 1 |
| TMC5 | 1 | 0 | 0 | 0 | 0 | 0 | 0 | 0 | 1 |
| TMEM132A | 1 | 0 | 0 | 0 | 0 | 0 | 0 | 0 | 1 |
| TMC4 | 0 | 1 | 0 | 0 | 0 | 0 | 0 | 0 | 1 |
| CCAR1 | 1 | 0 | 0 | 0 | 0 | 0 | 0 | 0 | 1 |
| N4BP1 | 1 | 0 | 0 | 0 | 0 | 0 | 0 | 0 | 1 |
| OR5V1 | 1 | 0 | 0 | 0 | 0 | 0 | 0 | 0 | 1 |
| CDK13 | 1 | 0 | 0 | 0 | 0 | 0 | 0 | 0 | 1 |
| HDAC5 | 1 | 0 | 0 | 0 | 0 | 0 | 0 | 0 | 1 |
| CHRNA5 | 1 | 0 | 0 | 0 | 0 | 0 | 0 | 0 | 1 |
| DNM3 | 1 | 0 | 0 | 0 | 0 | 0 | 0 | 0 | 1 |
| RGL1 | 1 | 0 | 0 | 0 | 0 | 0 | 0 | 0 | 1 |
| DHX37 | 1 | 0 | 0 | 0 | 0 | 0 | 0 | 0 | 1 |
| NOTCH4 | 1 | 0 | 0 | 0 | 0 | 0 | 0 | 0 | 1 |
| ZNF185 | 1 | 0 | 0 | 0 | 0 | 0 | 0 | 0 | 1 |
| SCAPER | 1 | 0 | 0 | 0 | 0 | 0 | 0 | 0 | 1 |
| DIS3 | 1 | 0 | 0 | 0 | 0 | 0 | 0 | 0 | 1 |
| NUP210 | 1 | 0 | 0 | 0 | 0 | 0 | 0 | 0 | 1 |
| TGM2 | 1 | 0 | 0 | 0 | 0 | 0 | 0 | 0 | 1 |
| MCPH1 | 1 | 0 | 0 | 0 | 0 | 0 | 0 | 0 | 1 |
| ATP13A4 | 1 | 0 | 0 | 0 | 0 | 0 | 0 | 0 | 1 |
| MLLT10 | 1 | 0 | 0 | 0 | 0 | 0 | 0 | 0 | 1 |
| POGZ | 1 | 0 | 0 | 0 | 0 | 0 | 0 | 0 | 1 |
| DACH1 | 1 | 0 | 0 | 0 | 0 | 0 | 0 | 0 | 1 |
| SPRYD3 | 0 | 1 | 0 | 0 | 0 | 0 | 0 | 0 | 1 |

| | | | | | | | | | |
|---|---|---|---|---|---|---|---|---|---|
| ATP2C1 | 1 | 0 | 0 | 0 | 0 | 0 | 0 | 0 | 1 |
| INVS | 1 | 0 | 0 | 0 | 0 | 0 | 0 | 0 | 1 |
| SPAG9 | 1 | 0 | 0 | 0 | 0 | 0 | 0 | 0 | 1 |
| DDX23 | 1 | 0 | 0 | 0 | 0 | 0 | 0 | 0 | 1 |
| TMIGD1 | 1 | 0 | 0 | 0 | 0 | 0 | 0 | 0 | 1 |
| C15orf39 | 1 | 0 | 0 | 0 | 0 | 0 | 0 | 0 | 1 |
| TRAK1 | 1 | 0 | 0 | 0 | 0 | 0 | 0 | 0 | 1 |
| KIAA1432 | 1 | 0 | 0 | 0 | 0 | 0 | 0 | 0 | 1 |
| AGRN | 1 | 0 | 0 | 0 | 0 | 0 | 0 | 0 | 1 |
| STARD13 | 1 | 0 | 0 | 0 | 0 | 0 | 0 | 0 | 1 |
| CEACAM3 | 1 | 0 | 0 | 0 | 0 | 0 | 0 | 0 | 1 |
| OR2F2 | 1 | 0 | 0 | 0 | 0 | 0 | 0 | 0 | 1 |
| IQSEC3 | 1 | 0 | 0 | 0 | 0 | 0 | 0 | 0 | 1 |
| PLXNB2 | 1 | 0 | 0 | 0 | 0 | 0 | 0 | 0 | 1 |
| TRAT1 | 1 | 0 | 0 | 0 | 0 | 0 | 0 | 0 | 1 |
| DCAF12L1 | 1 | 0 | 0 | 0 | 0 | 0 | 0 | 0 | 1 |
| TRIM3 | 1 | 0 | 0 | 0 | 0 | 0 | 0 | 0 | 1 |
| TFR2 | 1 | 0 | 0 | 0 | 0 | 0 | 0 | 0 | 1 |
| KIAA1609 | 1 | 0 | 0 | 0 | 0 | 0 | 0 | 0 | 1 |
| ZNF323 | 1 | 0 | 0 | 0 | 0 | 0 | 0 | 0 | 1 |
| CPSF7 | 1 | 0 | 0 | 0 | 0 | 0 | 0 | 0 | 1 |
| NLRP4 | 1 | 0 | 0 | 0 | 0 | 0 | 0 | 0 | 1 |
| ADAMTS2 | 0 | 0 | 0 | 0 | 1 | 0 | 0 | 0 | 1 |
| MORN5 | 1 | 0 | 0 | 0 | 0 | 0 | 0 | 0 | 1 |
| RIPK4 | 1 | 0 | 0 | 0 | 0 | 0 | 0 | 0 | 1 |
| LRIT1 | 1 | 0 | 0 | 0 | 0 | 0 | 0 | 0 | 1 |
| SEC24A | 1 | 0 | 0 | 0 | 0 | 0 | 0 | 0 | 1 |
| EXOC6B | 1 | 0 | 0 | 0 | 0 | 0 | 0 | 0 | 1 |
| OR4S1 | 1 | 0 | 0 | 0 | 0 | 0 | 0 | 0 | 1 |
| TRPC3 | 1 | 0 | 0 | 0 | 0 | 0 | 0 | 0 | 1 |
| RGP1 | 1 | 0 | 0 | 0 | 0 | 0 | 0 | 0 | 1 |
| GPRC5C | 1 | 0 | 0 | 0 | 0 | 0 | 0 | 0 | 1 |
| SMARCA5 | 1 | 0 | 0 | 0 | 0 | 0 | 0 | 0 | 1 |
| PRKG1 | 1 | 0 | 0 | 0 | 0 | 0 | 0 | 0 | 1 |
| CASS4 | 1 | 0 | 0 | 0 | 0 | 0 | 0 | 0 | 1 |
| GALC | 1 | 0 | 0 | 0 | 0 | 0 | 0 | 0 | 1 |
| ZC3H4 | 1 | 0 | 0 | 0 | 0 | 0 | 0 | 0 | 1 |
| OR5AK2 | 1 | 0 | 0 | 0 | 0 | 0 | 0 | 0 | 1 |
| APBB1 | 1 | 0 | 0 | 0 | 0 | 0 | 0 | 0 | 1 |
| FLII | 1 | 0 | 0 | 0 | 0 | 0 | 0 | 0 | 1 |
| SLC25A12 | 1 | 0 | 0 | 0 | 0 | 0 | 0 | 0 | 1 |
| NCDN | 1 | 0 | 0 | 0 | 0 | 0 | 0 | 0 | 1 |
| GBX2 | 1 | 0 | 0 | 0 | 0 | 0 | 0 | 0 | 1 |
| SERBP1 | 1 | 0 | 0 | 0 | 0 | 0 | 0 | 0 | 1 |
| FGF23 | 1 | 0 | 0 | 0 | 0 | 0 | 0 | 0 | 1 |
| PRSS35 | 1 | 0 | 0 | 0 | 0 | 0 | 0 | 0 | 1 |

| | | | | | | | | | |
|---|---|---|---|---|---|---|---|---|---|
| ZNF429 | 1 | 0 | 0 | 0 | 0 | 0 | 0 | 0 | 1 |
| ZNF238 | 1 | 0 | 0 | 0 | 0 | 0 | 0 | 0 | 1 |
| CAMK1G | 1 | 0 | 0 | 0 | 0 | 0 | 0 | 0 | 1 |
| TNS3 | 1 | 0 | 0 | 0 | 0 | 0 | 0 | 0 | 1 |
| RLTPR | 1 | 0 | 0 | 0 | 0 | 0 | 0 | 0 | 1 |
| EML1 | 1 | 0 | 0 | 0 | 0 | 0 | 0 | 0 | 1 |
| LBR | 1 | 0 | 0 | 0 | 0 | 0 | 0 | 0 | 1 |
| CC2D1B | 1 | 0 | 0 | 0 | 0 | 0 | 0 | 0 | 1 |
| KCNC2 | 1 | 0 | 0 | 0 | 0 | 0 | 0 | 0 | 1 |
| FGD6 | 1 | 0 | 0 | 0 | 0 | 0 | 0 | 0 | 1 |
| WAC | 1 | 0 | 0 | 0 | 0 | 0 | 0 | 0 | 1 |
| FUK | 1 | 0 | 0 | 0 | 0 | 0 | 0 | 0 | 1 |
| PACS1 | 1 | 0 | 0 | 0 | 0 | 0 | 0 | 0 | 1 |
| CLTC | 1 | 0 | 0 | 0 | 0 | 0 | 0 | 0 | 1 |
| KDM5C | 1 | 0 | 0 | 0 | 0 | 0 | 0 | 0 | 1 |
| TMEM2 | 1 | 0 | 0 | 0 | 0 | 0 | 0 | 0 | 1 |
| ST14 | 1 | 0 | 0 | 0 | 0 | 0 | 0 | 0 | 1 |
| ASNSD1 | 1 | 0 | 0 | 0 | 0 | 0 | 0 | 0 | 1 |
| ERN2 | 1 | 0 | 0 | 0 | 0 | 0 | 0 | 0 | 1 |
| RBM33 | 1 | 0 | 0 | 0 | 0 | 0 | 0 | 0 | 1 |
| SARDH | 1 | 0 | 0 | 0 | 0 | 0 | 0 | 0 | 1 |
| USP53 | 1 | 0 | 0 | 0 | 0 | 0 | 0 | 0 | 1 |
| OR8J1 | 1 | 0 | 0 | 0 | 0 | 0 | 0 | 0 | 1 |
| LRRCC1 | 1 | 0 | 0 | 0 | 0 | 0 | 0 | 0 | 1 |
| FAM196A | 1 | 0 | 0 | 0 | 0 | 0 | 0 | 0 | 1 |
| ACP1 | 1 | 0 | 0 | 0 | 0 | 0 | 0 | 0 | 1 |
| RGS12 | 1 | 0 | 0 | 0 | 0 | 0 | 0 | 0 | 1 |
| TSPAN5 | 1 | 0 | 0 | 0 | 0 | 0 | 0 | 0 | 1 |
| MAP4K4 | 1 | 0 | 0 | 0 | 0 | 0 | 0 | 0 | 1 |
| ARHGAP9 | 1 | 0 | 0 | 0 | 0 | 0 | 0 | 0 | 1 |
| FIGN | 1 | 0 | 0 | 0 | 0 | 0 | 0 | 0 | 1 |
| ZKSCAN2 | 1 | 0 | 0 | 0 | 0 | 0 | 0 | 0 | 1 |
| OR6C68 | 1 | 0 | 0 | 0 | 0 | 0 | 0 | 0 | 1 |
| LCE2D | 1 | 0 | 0 | 0 | 0 | 0 | 0 | 0 | 1 |
| GLDC | 1 | 0 | 0 | 0 | 0 | 0 | 0 | 0 | 1 |
| MPRIP | 1 | 0 | 0 | 0 | 0 | 0 | 0 | 0 | 1 |
| MS4A10 | 1 | 0 | 0 | 0 | 0 | 0 | 0 | 0 | 1 |
| SASH1 | 1 | 0 | 0 | 0 | 0 | 0 | 0 | 0 | 1 |
| TRIP11 | 1 | 0 | 0 | 0 | 0 | 0 | 0 | 0 | 1 |
| LDLR | 1 | 0 | 0 | 0 | 0 | 0 | 0 | 0 | 1 |
| ATP13A2 | 1 | 0 | 0 | 0 | 0 | 0 | 0 | 0 | 1 |
| OR13C4 | 1 | 0 | 0 | 0 | 0 | 0 | 0 | 0 | 1 |
| TRIM41 | 1 | 0 | 0 | 0 | 0 | 0 | 0 | 0 | 1 |
| GTPBP3 | 1 | 0 | 0 | 0 | 0 | 0 | 0 | 0 | 1 |
| NPFFR2 | 1 | 0 | 0 | 0 | 0 | 0 | 0 | 0 | 1 |
| VAPA | 1 | 0 | 0 | 0 | 0 | 0 | 0 | 0 | 1 |

| | | | | | | | | | |
|---|---|---|---|---|---|---|---|---|---|
| IBTK | 1 | 0 | 0 | 0 | 0 | 0 | 0 | 0 | 1 |
| NDUFB1 | 1 | 0 | 0 | 0 | 0 | 0 | 0 | 0 | 1 |
| FTSJD2 | 1 | 0 | 0 | 0 | 0 | 0 | 0 | 0 | 1 |
| ZNF549 | 1 | 0 | 0 | 0 | 0 | 0 | 0 | 0 | 1 |
| RSRC1 | 1 | 0 | 0 | 0 | 0 | 0 | 0 | 0 | 1 |
| FAM160A2 | 1 | 0 | 0 | 0 | 0 | 0 | 0 | 0 | 1 |
| IL23R | 0 | 1 | 0 | 0 | 0 | 0 | 0 | 0 | 1 |
| SHANK3 | 1 | 0 | 0 | 0 | 0 | 0 | 0 | 0 | 1 |
| TFAP2B | 1 | 0 | 0 | 0 | 0 | 0 | 0 | 0 | 1 |
| C10orf88 | 1 | 0 | 0 | 0 | 0 | 0 | 0 | 0 | 1 |
| MARK2 | 1 | 0 | 0 | 0 | 0 | 0 | 0 | 0 | 1 |
| OR8K3 | 1 | 0 | 0 | 0 | 0 | 0 | 0 | 0 | 1 |
| RPS6KC1 | 1 | 0 | 0 | 0 | 0 | 0 | 0 | 0 | 1 |
| CLASP1 | 1 | 0 | 0 | 0 | 0 | 0 | 0 | 0 | 1 |
| TRIM45 | 1 | 0 | 0 | 0 | 0 | 0 | 0 | 0 | 1 |
| ATXN2 | 1 | 0 | 0 | 0 | 0 | 0 | 0 | 0 | 1 |
| CREB3 | 1 | 0 | 0 | 0 | 0 | 0 | 0 | 0 | 1 |
| WDR31 | 1 | 0 | 0 | 0 | 0 | 0 | 0 | 0 | 1 |
| KCNJ8 | 1 | 0 | 0 | 0 | 0 | 0 | 0 | 0 | 1 |
| PHLPP1 | 1 | 0 | 0 | 0 | 0 | 0 | 0 | 0 | 1 |
| OR1L4 | 1 | 0 | 0 | 0 | 0 | 0 | 0 | 0 | 1 |
| GPS2 | 1 | 0 | 0 | 0 | 0 | 0 | 0 | 0 | 1 |
| VAV3 | 0 | 1 | 0 | 0 | 0 | 0 | 0 | 0 | 1 |
| PLA2G4E | 1 | 0 | 0 | 0 | 0 | 0 | 0 | 0 | 1 |
| PI15 | 1 | 0 | 0 | 0 | 0 | 0 | 0 | 0 | 1 |
| CPT2 | 1 | 0 | 0 | 0 | 0 | 0 | 0 | 0 | 1 |
| DDX20 | 1 | 0 | 0 | 0 | 0 | 0 | 0 | 0 | 1 |
| ARAP1 | 1 | 0 | 0 | 0 | 0 | 0 | 0 | 0 | 1 |
| RNASE11 | 1 | 0 | 0 | 0 | 0 | 0 | 0 | 0 | 1 |
| C9orf50 | 1 | 0 | 0 | 0 | 0 | 0 | 0 | 0 | 1 |
| C12orf4 | 1 | 0 | 0 | 0 | 0 | 0 | 0 | 0 | 1 |
| RAI1 | 1 | 0 | 0 | 0 | 0 | 0 | 0 | 0 | 1 |
| TMEM168 | 1 | 0 | 0 | 0 | 0 | 0 | 0 | 0 | 1 |
| PRRT3 | 1 | 0 | 0 | 0 | 0 | 0 | 0 | 0 | 1 |
| MSH6 | 1 | 0 | 0 | 0 | 0 | 0 | 0 | 0 | 1 |
| ZNF10 | 1 | 0 | 0 | 0 | 0 | 0 | 0 | 0 | 1 |
| ULK1 | 1 | 0 | 0 | 0 | 0 | 0 | 0 | 0 | 1 |
| ARMC5 | 1 | 0 | 0 | 0 | 0 | 0 | 0 | 0 | 1 |
| ARMC8 | 1 | 0 | 0 | 0 | 0 | 0 | 0 | 0 | 1 |
| SLC12A7 | 1 | 0 | 0 | 0 | 0 | 0 | 0 | 0 | 1 |
| PIGR | 1 | 0 | 0 | 0 | 0 | 0 | 0 | 0 | 1 |
| PLXND1 | 1 | 0 | 0 | 0 | 0 | 0 | 0 | 0 | 1 |
| LDHD | 1 | 0 | 0 | 0 | 0 | 0 | 0 | 0 | 1 |
| DUSP27 | 1 | 0 | 0 | 0 | 0 | 0 | 0 | 0 | 1 |
| TNRC18 | 1 | 0 | 0 | 0 | 0 | 0 | 0 | 0 | 1 |
| ERMN | 1 | 0 | 0 | 0 | 0 | 0 | 0 | 0 | 1 |

| | | | | | | | | | |
|---|---|---|---|---|---|---|---|---|---|
| HECTD3 | 1 | 0 | 0 | 0 | 0 | 0 | 0 | 0 | 1 |
| GPSM1 | 1 | 0 | 0 | 0 | 0 | 0 | 0 | 0 | 1 |
| DFNB31 | 1 | 0 | 0 | 0 | 0 | 0 | 0 | 0 | 1 |
| PDXDC1 | 1 | 0 | 0 | 0 | 0 | 0 | 0 | 0 | 1 |
| ZFP37 | 1 | 0 | 0 | 0 | 0 | 0 | 0 | 0 | 1 |
| GATA4 | 1 | 0 | 0 | 0 | 0 | 0 | 0 | 0 | 1 |
| AVPR2 | 1 | 0 | 0 | 0 | 0 | 0 | 0 | 0 | 1 |
| ARHGAP5 | 1 | 0 | 0 | 0 | 0 | 0 | 0 | 0 | 1 |
| ZNF2 | 1 | 0 | 0 | 0 | 0 | 0 | 0 | 0 | 1 |
| CC2D1A | 0 | 1 | 0 | 0 | 0 | 0 | 0 | 0 | 1 |
| NFKB2 | 1 | 0 | 0 | 0 | 0 | 0 | 0 | 0 | 1 |
| STXBP1 | 1 | 0 | 0 | 0 | 0 | 0 | 0 | 0 | 1 |
| ZNF516 | 1 | 0 | 0 | 0 | 0 | 0 | 0 | 0 | 1 |
| MOGAT3 | 1 | 0 | 0 | 0 | 0 | 0 | 0 | 0 | 1 |
| HSD17B2 | 1 | 0 | 0 | 0 | 0 | 0 | 0 | 0 | 1 |
| SEMA4A | 1 | 0 | 0 | 0 | 0 | 0 | 0 | 0 | 1 |
| INADL | 1 | 0 | 0 | 0 | 0 | 0 | 0 | 0 | 1 |
| CRISPLD2 | 1 | 0 | 0 | 0 | 0 | 0 | 0 | 0 | 1 |
| TMPRSS5 | 1 | 0 | 0 | 0 | 0 | 0 | 0 | 0 | 1 |
| HTR1A | 1 | 0 | 0 | 0 | 0 | 0 | 0 | 0 | 1 |
| PGM2 | 1 | 0 | 0 | 0 | 0 | 0 | 0 | 0 | 1 |
| PDE4B | 1 | 0 | 0 | 0 | 0 | 0 | 0 | 0 | 1 |
| KIDINS220 | 1 | 0 | 0 | 0 | 0 | 0 | 0 | 0 | 1 |
| NUP160 | 1 | 0 | 0 | 0 | 0 | 0 | 0 | 0 | 1 |
| ABI2 | 1 | 0 | 0 | 0 | 0 | 0 | 0 | 0 | 1 |
| AFMID | 1 | 0 | 0 | 0 | 0 | 0 | 0 | 0 | 1 |
| TRIM5 | 1 | 0 | 0 | 0 | 0 | 0 | 0 | 0 | 1 |
| FAM71F1 | 1 | 0 | 0 | 0 | 0 | 0 | 0 | 0 | 1 |
| TFIP11 | 1 | 0 | 0 | 0 | 0 | 0 | 0 | 0 | 1 |
| CEP164 | 1 | 0 | 0 | 0 | 0 | 0 | 0 | 0 | 1 |
| RANBP6 | 1 | 0 | 0 | 0 | 0 | 0 | 0 | 0 | 1 |
| MAML3 | 1 | 0 | 0 | 0 | 0 | 0 | 0 | 0 | 1 |
| SCN11A | 1 | 0 | 0 | 0 | 0 | 0 | 0 | 0 | 1 |
| TOP2B | 1 | 0 | 0 | 0 | 0 | 0 | 0 | 0 | 1 |
| MCM8 | 1 | 0 | 0 | 0 | 0 | 0 | 0 | 0 | 1 |
| JAG2 | 1 | 0 | 0 | 0 | 0 | 0 | 0 | 0 | 1 |
| IL2RG | 1 | 0 | 0 | 0 | 0 | 0 | 0 | 0 | 1 |
| MOV10 | 1 | 0 | 0 | 0 | 0 | 0 | 0 | 0 | 1 |
| MINPP1 | 1 | 0 | 0 | 0 | 0 | 0 | 0 | 0 | 1 |
| ODC1 | 1 | 0 | 0 | 0 | 0 | 0 | 0 | 0 | 1 |
| ATAD2B | 1 | 0 | 0 | 0 | 0 | 0 | 0 | 0 | 1 |
| KIAA1462 | 1 | 0 | 0 | 0 | 0 | 0 | 0 | 0 | 1 |
| ATP7A | 1 | 0 | 0 | 0 | 0 | 0 | 0 | 0 | 1 |
| SV2A | 1 | 0 | 0 | 0 | 0 | 0 | 0 | 0 | 1 |
| PCDH17 | 1 | 0 | 0 | 0 | 0 | 0 | 0 | 0 | 1 |
| XRCC1 | 1 | 0 | 0 | 0 | 0 | 0 | 0 | 0 | 1 |

| | | | | | | | | | |
|---|---|---|---|---|---|---|---|---|---|
| ABL2 | 1 | 0 | 0 | 0 | 0 | 0 | 0 | 0 | 1 |
| MBD6 | 0 | 0 | 1 | 0 | 0 | 0 | 0 | 0 | 1 |
| ROBO3 | 1 | 0 | 0 | 0 | 0 | 0 | 0 | 0 | 1 |
| AFAP1L2 | 1 | 0 | 0 | 0 | 0 | 0 | 0 | 0 | 1 |
| EIF3G | 1 | 0 | 0 | 0 | 0 | 0 | 0 | 0 | 1 |
| PTPN4 | 1 | 0 | 0 | 0 | 0 | 0 | 0 | 0 | 1 |
| LRRTM3 | 1 | 0 | 0 | 0 | 0 | 0 | 0 | 0 | 1 |
| IL33 | 1 | 0 | 0 | 0 | 0 | 0 | 0 | 0 | 1 |
| PPP2R5A | 1 | 0 | 0 | 0 | 0 | 0 | 0 | 0 | 1 |
| KLHL15 | 0 | 1 | 0 | 0 | 0 | 0 | 0 | 0 | 1 |
| MAP3K4 | 1 | 0 | 0 | 0 | 0 | 0 | 0 | 0 | 1 |
| SF3B3 | 1 | 0 | 0 | 0 | 0 | 0 | 0 | 0 | 1 |
| CEP55 | 1 | 0 | 0 | 0 | 0 | 0 | 0 | 0 | 1 |
| MAP4 | 1 | 0 | 0 | 0 | 0 | 0 | 0 | 0 | 1 |
| STAG3 | 1 | 0 | 0 | 0 | 0 | 0 | 0 | 0 | 1 |
| TET2 | 1 | 0 | 0 | 0 | 0 | 0 | 0 | 0 | 1 |
| NCOA7 | 1 | 0 | 0 | 0 | 0 | 0 | 0 | 0 | 1 |
| ITIH5 | 1 | 0 | 0 | 0 | 0 | 0 | 0 | 0 | 1 |
| DSPP | 1 | 0 | 0 | 0 | 0 | 0 | 0 | 0 | 1 |
| H2AFY2 | 1 | 0 | 0 | 0 | 0 | 0 | 0 | 0 | 1 |
| AKAP1 | 1 | 0 | 0 | 0 | 0 | 0 | 0 | 0 | 1 |
| PARP4 | 1 | 0 | 0 | 0 | 0 | 0 | 0 | 0 | 1 |
| C4orf40 | 1 | 0 | 0 | 0 | 0 | 0 | 0 | 0 | 1 |
| SRCIN1 | 1 | 0 | 0 | 0 | 0 | 0 | 0 | 0 | 1 |
| SATB2 | 1 | 0 | 0 | 0 | 0 | 0 | 0 | 0 | 1 |
| ZNF142 | 1 | 0 | 0 | 0 | 0 | 0 | 0 | 0 | 1 |
| OR10AG1 | 1 | 0 | 0 | 0 | 0 | 0 | 0 | 0 | 1 |
| RNPEP | 1 | 0 | 0 | 0 | 0 | 0 | 0 | 0 | 1 |
| NPHP4 | 1 | 0 | 0 | 0 | 0 | 0 | 0 | 0 | 1 |
| DCST2 | 1 | 0 | 0 | 0 | 0 | 0 | 0 | 0 | 1 |
| ASB16 | 1 | 0 | 0 | 0 | 0 | 0 | 0 | 0 | 1 |
| KIAA1217 | 1 | 0 | 0 | 0 | 0 | 0 | 0 | 0 | 1 |
| CCDC115 | 1 | 0 | 0 | 0 | 0 | 0 | 0 | 0 | 1 |
| CWF19L2 | 1 | 0 | 0 | 0 | 0 | 0 | 0 | 0 | 1 |
| THRAP3 | 1 | 0 | 0 | 0 | 0 | 0 | 0 | 0 | 1 |
| LEMD2 | 1 | 0 | 0 | 0 | 0 | 0 | 0 | 0 | 1 |
| ST8SIA4 | 1 | 0 | 0 | 0 | 0 | 0 | 0 | 0 | 1 |
| ANK1 | 1 | 0 | 0 | 0 | 0 | 0 | 0 | 0 | 1 |
| TMEM74 | 1 | 0 | 0 | 0 | 0 | 0 | 0 | 0 | 1 |
| CDK11B | 1 | 0 | 0 | 0 | 0 | 0 | 0 | 0 | 1 |
| PUM2 | 1 | 0 | 0 | 0 | 0 | 0 | 0 | 0 | 1 |
| RPL3L | 1 | 0 | 0 | 0 | 0 | 0 | 0 | 0 | 1 |
| MMAA | 1 | 0 | 0 | 0 | 0 | 0 | 0 | 0 | 1 |
| FBL | 1 | 0 | 0 | 0 | 0 | 0 | 0 | 0 | 1 |
| CSPG4 | 1 | 0 | 0 | 0 | 0 | 0 | 0 | 0 | 1 |
| C15orf42 | 1 | 0 | 0 | 0 | 0 | 0 | 0 | 0 | 1 |

| | | | | | | | | | |
|---|---|---|---|---|---|---|---|---|---|
| PHF8 | 1 | 0 | 0 | 0 | 0 | 0 | 0 | 0 | 1 |
| SLC22A23 | 1 | 0 | 0 | 0 | 0 | 0 | 0 | 0 | 1 |
| NLRC3 | 1 | 0 | 0 | 0 | 0 | 0 | 0 | 0 | 1 |
| UPK1A | 1 | 0 | 0 | 0 | 0 | 0 | 0 | 0 | 1 |
| TLK1 | 1 | 0 | 0 | 0 | 0 | 0 | 0 | 0 | 1 |
| KDM4A | 1 | 0 | 0 | 0 | 0 | 0 | 0 | 0 | 1 |
| PLA1A | 1 | 0 | 0 | 0 | 0 | 0 | 0 | 0 | 1 |
| GABRR1 | 1 | 0 | 0 | 0 | 0 | 0 | 0 | 0 | 1 |
| TTBK2 | 1 | 0 | 0 | 0 | 0 | 0 | 0 | 0 | 1 |
| C12orf35 | 1 | 0 | 0 | 0 | 0 | 0 | 0 | 0 | 1 |
| LRRC30 | 1 | 0 | 0 | 0 | 0 | 0 | 0 | 0 | 1 |
| WDR3 | 1 | 0 | 0 | 0 | 0 | 0 | 0 | 0 | 1 |
| GRM5 | 1 | 0 | 0 | 0 | 0 | 0 | 0 | 0 | 1 |
| TAGAP | 1 | 0 | 0 | 0 | 0 | 0 | 0 | 0 | 1 |
| UBAP2 | 1 | 0 | 0 | 0 | 0 | 0 | 0 | 0 | 1 |
| DNAJC28 | 1 | 0 | 0 | 0 | 0 | 0 | 0 | 0 | 1 |
| NLRP6 | 1 | 0 | 0 | 0 | 0 | 0 | 0 | 0 | 1 |
| SERPINA12 | 1 | 0 | 0 | 0 | 0 | 0 | 0 | 0 | 1 |
| HK2 | 1 | 0 | 0 | 0 | 0 | 0 | 0 | 0 | 1 |
| SELENBP1 | 1 | 0 | 0 | 0 | 0 | 0 | 0 | 0 | 1 |
| FREM3 | 1 | 0 | 0 | 0 | 0 | 0 | 0 | 0 | 1 |
| TGIF1 | 1 | 0 | 0 | 0 | 0 | 0 | 0 | 0 | 1 |
| SPG7 | 1 | 0 | 0 | 0 | 0 | 0 | 0 | 0 | 1 |
| SLC43A1 | 1 | 0 | 0 | 0 | 0 | 0 | 0 | 0 | 1 |
| XPO4 | 0 | 1 | 0 | 0 | 0 | 0 | 0 | 0 | 1 |
| IPO9 | 1 | 0 | 0 | 0 | 0 | 0 | 0 | 0 | 1 |
| MYH11 | 1 | 0 | 0 | 0 | 0 | 0 | 0 | 0 | 1 |
| SEMA3G | 1 | 0 | 0 | 0 | 0 | 0 | 0 | 0 | 1 |
| XKR4 | 1 | 0 | 0 | 0 | 0 | 0 | 0 | 0 | 1 |
| DUOX1 | 1 | 0 | 0 | 0 | 0 | 0 | 0 | 0 | 1 |
| ZNRF4 | 1 | 0 | 0 | 0 | 0 | 0 | 0 | 0 | 1 |
| ZNF215 | 1 | 0 | 0 | 0 | 0 | 0 | 0 | 0 | 1 |
| OSBPL1A | 1 | 0 | 0 | 0 | 0 | 0 | 0 | 0 | 1 |
| PHC2 | 1 | 0 | 0 | 0 | 0 | 0 | 0 | 0 | 1 |
| COL11A2 | 1 | 0 | 0 | 0 | 0 | 0 | 0 | 0 | 1 |
| FOXG1 | 1 | 0 | 0 | 0 | 0 | 0 | 0 | 0 | 1 |
| GUCY1B3 | 1 | 0 | 0 | 0 | 0 | 0 | 0 | 0 | 1 |
| MYO6 | 1 | 0 | 0 | 0 | 0 | 0 | 0 | 0 | 1 |
| CHI3L2 | 1 | 0 | 0 | 0 | 0 | 0 | 0 | 0 | 1 |
| GRIN3A | 1 | 0 | 0 | 0 | 0 | 0 | 0 | 0 | 1 |
| POMT2 | 1 | 0 | 0 | 0 | 0 | 0 | 0 | 0 | 1 |
| GRIN2C | 1 | 0 | 0 | 0 | 0 | 0 | 0 | 0 | 1 |
| UNC5A | 1 | 0 | 0 | 0 | 0 | 0 | 0 | 0 | 1 |
| SRPK1 | 1 | 0 | 0 | 0 | 0 | 0 | 0 | 0 | 1 |
| PRR12 | 1 | 0 | 0 | 0 | 0 | 0 | 0 | 0 | 1 |
| ZFAT | 1 | 0 | 0 | 0 | 0 | 0 | 0 | 0 | 1 |

| | | | | | | | | | |
|---|---|---|---|---|---|---|---|---|---|
| ZNF281 | 1 | 0 | 0 | 0 | 0 | 0 | 0 | 0 | 1 |
| ZNF532 | 0 | 1 | 0 | 0 | 0 | 0 | 0 | 0 | 1 |
| MSRA | 1 | 0 | 0 | 0 | 0 | 0 | 0 | 0 | 1 |
| DDO | 1 | 0 | 0 | 0 | 0 | 0 | 0 | 0 | 1 |
| ATP5A1 | 1 | 0 | 0 | 0 | 0 | 0 | 0 | 0 | 1 |
| SKIL | 1 | 0 | 0 | 0 | 0 | 0 | 0 | 0 | 1 |
| ST8SIA3 | 1 | 0 | 0 | 0 | 0 | 0 | 0 | 0 | 1 |
| USP35 | 1 | 0 | 0 | 0 | 0 | 0 | 0 | 0 | 1 |
| TCF20 | 1 | 0 | 0 | 0 | 0 | 0 | 0 | 0 | 1 |
| MAP4K3 | 1 | 0 | 0 | 0 | 0 | 0 | 0 | 0 | 1 |
| OR11G2 | 1 | 0 | 0 | 0 | 0 | 0 | 0 | 0 | 1 |
| SERPINA5 | 1 | 0 | 0 | 0 | 0 | 0 | 0 | 0 | 1 |
| PAX5 | 1 | 0 | 0 | 0 | 0 | 0 | 0 | 0 | 1 |
| MOGS | 1 | 0 | 0 | 0 | 0 | 0 | 0 | 0 | 1 |
| CEP290 | 1 | 0 | 0 | 0 | 0 | 0 | 0 | 0 | 1 |
| SEMA6C | 0 | 0 | 1 | 0 | 0 | 0 | 0 | 0 | 1 |
| HRNR | 0 | 1 | 0 | 0 | 0 | 0 | 0 | 0 | 1 |
| NUMBL | 1 | 0 | 0 | 0 | 0 | 0 | 0 | 0 | 1 |
| PID1 | 1 | 0 | 0 | 0 | 0 | 0 | 0 | 0 | 1 |
| ERC1 | 1 | 0 | 0 | 0 | 0 | 0 | 0 | 0 | 1 |
| PDCD6IP | 1 | 0 | 0 | 0 | 0 | 0 | 0 | 0 | 1 |
| MED16 | 0 | 0 | 1 | 0 | 0 | 0 | 0 | 0 | 1 |
| LLGL2 | 1 | 0 | 0 | 0 | 0 | 0 | 0 | 0 | 1 |
| CILP2 | 1 | 0 | 0 | 0 | 0 | 0 | 0 | 0 | 1 |
| PROM1 | 1 | 0 | 0 | 0 | 0 | 0 | 0 | 0 | 1 |
| SLC22A11 | 1 | 0 | 0 | 0 | 0 | 0 | 0 | 0 | 1 |
| PITPNM1 | 1 | 0 | 0 | 0 | 0 | 0 | 0 | 0 | 1 |
| ST8SIA6 | 0 | 0 | 1 | 0 | 0 | 0 | 0 | 0 | 1 |
| ZNF250 | 1 | 0 | 0 | 0 | 0 | 0 | 0 | 0 | 1 |
| ADARB2 | 1 | 0 | 0 | 0 | 0 | 0 | 0 | 0 | 1 |
| BMS1 | 1 | 0 | 0 | 0 | 0 | 0 | 0 | 0 | 1 |
| KDM6B | 1 | 0 | 0 | 0 | 0 | 0 | 0 | 0 | 1 |
| C11orf94 | 0 | 0 | 1 | 0 | 0 | 0 | 0 | 0 | 1 |
| PPAT | 1 | 0 | 0 | 0 | 0 | 0 | 0 | 0 | 1 |
| SYNJ2 | 1 | 0 | 0 | 0 | 0 | 0 | 0 | 0 | 1 |
| MEGF6 | 1 | 0 | 0 | 0 | 0 | 0 | 0 | 0 | 1 |
| SLC7A8 | 1 | 0 | 0 | 0 | 0 | 0 | 0 | 0 | 1 |
| KIAA1731 | 1 | 0 | 0 | 0 | 0 | 0 | 0 | 0 | 1 |
| RPGRIP1L | 1 | 0 | 0 | 0 | 0 | 0 | 0 | 0 | 1 |
| TRANK1 | 1 | 0 | 0 | 0 | 0 | 0 | 0 | 0 | 1 |
| PDHA2 | 1 | 0 | 0 | 0 | 0 | 0 | 0 | 0 | 1 |
| GON4L | 1 | 0 | 0 | 0 | 0 | 0 | 0 | 0 | 1 |
| TCF7L2 | 1 | 0 | 0 | 0 | 0 | 0 | 0 | 0 | 1 |
| TNFRSF9 | 1 | 0 | 0 | 0 | 0 | 0 | 0 | 0 | 1 |
| PHKB | 1 | 0 | 0 | 0 | 0 | 0 | 0 | 0 | 1 |
| UMOD | 1 | 0 | 0 | 0 | 0 | 0 | 0 | 0 | 1 |

| | | | | | | | | | |
|---|---|---|---|---|---|---|---|---|---|
| CARD11 | 1 | 0 | 0 | 0 | 0 | 0 | 0 | 0 | 1 |
| KANK2 | 1 | 0 | 0 | 0 | 0 | 0 | 0 | 0 | 1 |
| LEMD3 | 1 | 0 | 0 | 0 | 0 | 0 | 0 | 0 | 1 |
| FNBP4 | 1 | 0 | 0 | 0 | 0 | 0 | 0 | 0 | 1 |
| AARS | 1 | 0 | 0 | 0 | 0 | 0 | 0 | 0 | 1 |
| NOTCH3 | 1 | 0 | 0 | 0 | 0 | 0 | 0 | 0 | 1 |
| DLGAP1 | 1 | 0 | 0 | 0 | 0 | 0 | 0 | 0 | 1 |
| AFP | 1 | 0 | 0 | 0 | 0 | 0 | 0 | 0 | 1 |
| FAM98B | 1 | 0 | 0 | 0 | 0 | 0 | 0 | 0 | 1 |
| ABCB11 | 1 | 0 | 0 | 0 | 0 | 0 | 0 | 0 | 1 |
| DSEL | 1 | 0 | 0 | 0 | 0 | 0 | 0 | 0 | 1 |
| RSBN1L | 1 | 0 | 0 | 0 | 0 | 0 | 0 | 0 | 1 |
| CDC27 | 1 | 0 | 0 | 0 | 0 | 0 | 0 | 0 | 1 |
| COL7A1 | 1 | 0 | 0 | 0 | 0 | 0 | 0 | 0 | 1 |
| PSKH2 | 1 | 0 | 0 | 0 | 0 | 0 | 0 | 0 | 1 |
| SLFN11 | 1 | 0 | 0 | 0 | 0 | 0 | 0 | 0 | 1 |
| GYS1 | 1 | 0 | 0 | 0 | 0 | 0 | 0 | 0 | 1 |
| NOVA2 | 1 | 0 | 0 | 0 | 0 | 0 | 0 | 0 | 1 |
| OR10J5 | 1 | 0 | 0 | 0 | 0 | 0 | 0 | 0 | 1 |
| ZNF469 | 1 | 0 | 0 | 0 | 0 | 0 | 0 | 0 | 1 |
| OR7A10 | 1 | 0 | 0 | 0 | 0 | 0 | 0 | 0 | 1 |
| RAB41 | 1 | 0 | 0 | 0 | 0 | 0 | 0 | 0 | 1 |
| PGR | 1 | 0 | 0 | 0 | 0 | 0 | 0 | 0 | 1 |
| DFFB | 1 | 0 | 0 | 0 | 0 | 0 | 0 | 0 | 1 |
| ETS2 | 1 | 0 | 0 | 0 | 0 | 0 | 0 | 0 | 1 |
| NT5C1A | 1 | 0 | 0 | 0 | 0 | 0 | 0 | 0 | 1 |
| ITSN2 | 1 | 0 | 0 | 0 | 0 | 0 | 0 | 0 | 1 |
| ARHGAP33 | 1 | 0 | 0 | 0 | 0 | 0 | 0 | 0 | 1 |
| STAT5B | 1 | 0 | 0 | 0 | 0 | 0 | 0 | 0 | 1 |
| C1orf141 | 1 | 0 | 0 | 0 | 0 | 0 | 0 | 0 | 1 |
| CDCP2 | 1 | 0 | 0 | 0 | 0 | 0 | 0 | 0 | 1 |
| MAB21L2 | 1 | 0 | 0 | 0 | 0 | 0 | 0 | 0 | 1 |
| NFAT5 | 1 | 0 | 0 | 0 | 0 | 0 | 0 | 0 | 1 |
| SEC24C | 1 | 0 | 0 | 0 | 0 | 0 | 0 | 0 | 1 |
| LAMP2 | 1 | 0 | 0 | 0 | 0 | 0 | 0 | 0 | 1 |
| TOPORS | 1 | 0 | 0 | 0 | 0 | 0 | 0 | 0 | 1 |
| MAPK8IP1 | 1 | 0 | 0 | 0 | 0 | 0 | 0 | 0 | 1 |
| KIAA0100 | 1 | 0 | 0 | 0 | 0 | 0 | 0 | 0 | 1 |
| KLHDC8A | 1 | 0 | 0 | 0 | 0 | 0 | 0 | 0 | 1 |
| TMTC3 | 1 | 0 | 0 | 0 | 0 | 0 | 0 | 0 | 1 |
| INTS7 | 1 | 0 | 0 | 0 | 0 | 0 | 0 | 0 | 1 |
| MATN3 | 1 | 0 | 0 | 0 | 0 | 0 | 0 | 0 | 1 |
| ELAVL1 | 1 | 0 | 0 | 0 | 0 | 0 | 0 | 0 | 1 |
| ASAP1 | 1 | 0 | 0 | 0 | 0 | 0 | 0 | 0 | 1 |
| AFF1 | 1 | 0 | 0 | 0 | 0 | 0 | 0 | 0 | 1 |
| AP3B1 | 1 | 0 | 0 | 0 | 0 | 0 | 0 | 0 | 1 |

| | | | | | | | | | |
|---|---|---|---|---|---|---|---|---|---|
| SLC25A43 | 1 | 0 | 0 | 0 | 0 | 0 | 0 | 0 | 1 |
| BCLAF1 | 1 | 0 | 0 | 0 | 0 | 0 | 0 | 0 | 1 |
| PPY | 1 | 0 | 0 | 0 | 0 | 0 | 0 | 0 | 1 |
| ZNF394 | 1 | 0 | 0 | 0 | 0 | 0 | 0 | 0 | 1 |
| LHFPL4 | 1 | 0 | 0 | 0 | 0 | 0 | 0 | 0 | 1 |
| PAXIP1 | 1 | 0 | 0 | 0 | 0 | 0 | 0 | 0 | 1 |
| SAFB | 1 | 0 | 0 | 0 | 0 | 0 | 0 | 0 | 1 |
| EVPL | 1 | 0 | 0 | 0 | 0 | 0 | 0 | 0 | 1 |
| RPS15 | 1 | 0 | 0 | 0 | 0 | 0 | 0 | 0 | 1 |
| KCNJ6 | 0 | 1 | 0 | 0 | 0 | 0 | 0 | 0 | 1 |
| C8orf42 | 1 | 0 | 0 | 0 | 0 | 0 | 0 | 0 | 1 |
| INO80 | 1 | 0 | 0 | 0 | 0 | 0 | 0 | 0 | 1 |
| YJEFN3 | 1 | 0 | 0 | 0 | 0 | 0 | 0 | 0 | 1 |
| POLR2E | 1 | 0 | 0 | 0 | 0 | 0 | 0 | 0 | 1 |
| GPRC5D | 1 | 0 | 0 | 0 | 0 | 0 | 0 | 0 | 1 |
| C11orf84 | 1 | 0 | 0 | 0 | 0 | 0 | 0 | 0 | 1 |
| FAM176A | 1 | 0 | 0 | 0 | 0 | 0 | 0 | 0 | 1 |
| IL26 | 1 | 0 | 0 | 0 | 0 | 0 | 0 | 0 | 1 |
| C19orf55 | 1 | 0 | 0 | 0 | 0 | 0 | 0 | 0 | 1 |
| TTC32 | 1 | 0 | 0 | 0 | 0 | 0 | 0 | 0 | 1 |
| OR10G3 | 1 | 0 | 0 | 0 | 0 | 0 | 0 | 0 | 1 |
| TPP1 | 1 | 0 | 0 | 0 | 0 | 0 | 0 | 0 | 1 |
| PLA2G2A | 1 | 0 | 0 | 0 | 0 | 0 | 0 | 0 | 1 |
| MRAP2 | 1 | 0 | 0 | 0 | 0 | 0 | 0 | 0 | 1 |
| ZG16 | 1 | 0 | 0 | 0 | 0 | 0 | 0 | 0 | 1 |
| RNASE13 | 1 | 0 | 0 | 0 | 0 | 0 | 0 | 0 | 1 |
| C10orf53 | 1 | 0 | 0 | 0 | 0 | 0 | 0 | 0 | 1 |
| IL20 | 1 | 0 | 0 | 0 | 0 | 0 | 0 | 0 | 1 |
| ARHGAP28 | 1 | 0 | 0 | 0 | 0 | 0 | 0 | 0 | 1 |
| FAM111B | 1 | 0 | 0 | 0 | 0 | 0 | 0 | 0 | 1 |
| FSHB | 1 | 0 | 0 | 0 | 0 | 0 | 0 | 0 | 1 |
| CWC15 | 1 | 0 | 0 | 0 | 0 | 0 | 0 | 0 | 1 |
| ADORA3 | 1 | 0 | 0 | 0 | 0 | 0 | 0 | 0 | 1 |
| LALBA | 1 | 0 | 0 | 0 | 0 | 0 | 0 | 0 | 1 |
| AP4S1 | 1 | 0 | 0 | 0 | 0 | 0 | 0 | 0 | 1 |
| ZNF704 | 1 | 0 | 0 | 0 | 0 | 0 | 0 | 0 | 1 |
| ST3GAL3 | 1 | 0 | 0 | 0 | 0 | 0 | 0 | 0 | 1 |
| HSPBAP1 | 1 | 0 | 0 | 0 | 0 | 0 | 0 | 0 | 1 |
| TGM3 | 1 | 0 | 0 | 0 | 0 | 0 | 0 | 0 | 1 |
| OR7A17 | 1 | 0 | 0 | 0 | 0 | 0 | 0 | 0 | 1 |
| RGS8 | 1 | 0 | 0 | 0 | 0 | 0 | 0 | 0 | 1 |
| SPTAN1 | 1 | 0 | 0 | 0 | 0 | 0 | 0 | 0 | 1 |
| MORF4L1 | 1 | 0 | 0 | 0 | 0 | 0 | 0 | 0 | 1 |
| GPI | 1 | 0 | 0 | 0 | 0 | 0 | 0 | 0 | 1 |
| CD40 | 1 | 0 | 0 | 0 | 0 | 0 | 0 | 0 | 1 |
| GOLM1 | 1 | 0 | 0 | 0 | 0 | 0 | 0 | 0 | 1 |

| | | | | | | | | | |
|---|---|---|---|---|---|---|---|---|---|
| BCKDK | 1 | 0 | 0 | 0 | 0 | 0 | 0 | 0 | 1 |
| FBXW4 | 1 | 0 | 0 | 0 | 0 | 0 | 0 | 0 | 1 |
| RAD52 | 1 | 0 | 0 | 0 | 0 | 0 | 0 | 0 | 1 |
| RNF13 | 1 | 0 | 0 | 0 | 0 | 0 | 0 | 0 | 1 |
