# Supplementary material for: Mutational landscape of MCPyV-positive and MCPyV-negative Merkel cell carcinomas with implications for immunotherapy
Source: Oncotarget. 2015 Dec 7;7(3):3403–15. doi: 10.18632/oncotarget.6494 (PMC4823115; doi:10.18632/oncotarget.6494)
Supplement: Supplementary file 3 [file oncotarget-07-3403-s003.docx]

# Supplementary Table S11. Table of predicted neoantigens identified in MCCs.

| Sample | Gene | Amino acid substitution | Wild-type Nonamer | Mutant Nonamer | HLA allele | Ka |
| --- | --- | --- | --- | --- | --- | --- |
|  |  |  |  |  |  |  |
| MCC11 | A2M | p.L786F | gisstasLr | gisstasFr | A0301 | 389 |
| MCC11 | ABI3BP | p.S49C | ttsdSillk | ttsdCillk | A0301 | 74 |
| MCC11 | ACVRL1 | p.D388N | rtDcfesyk | rtNcfesyk | A0301 | 28 |
| MCC11 | ACVRL1 | p.E470K | sglaqmmrE | sglaqmmrK | A0301 | 257 |
| MCC11 | ADORA3 | p.T194I | kTrsckapk | kIrsckapk | A0301 | 53 |
| MCC11 | AJAP1 | p.L301V | alittLvlk | alittVvlk | A0301 | 53 |
| MCC11 | ANAPC10 | p.D82N | tlciyaDyk | tlciyaNyk | A0301 | 69 |
| MCC11 | ANAPC10 | p.R70T | lvniqfrRk | lvniqfrTk | A0301 | 310 |
| MCC11 | ARHGAP25 | p.E131K | vlmassqaE | vlmassqaK | A0301 | 19 |
| MCC11 | ARHGAP39 | p.L859M | kskLrkkpk | kskMrkkpk | A0301 | 444 |
| MCC11 | ARHGAP39 | p.L859M | kLrkkpkpy | kMrkkpkpy | A0301 | 304 |
| MCC11 | ASTE1 | p.E520Q | kmlyaEfqr | kmlyaQfqr | A0301 | 141 |
| MCC11 | ATP1B2 | p.G279D | kfaGrvafk | kfaDrvafk | A0301 | 127 |
| MCC11 | ATP9B | p.M438I | avygwMmmk | avygwImmk | A0301 | 18 |
| MCC11 | AXL | p.P195S | slhvPglnk | slhvSglnk | A0301 | 34 |
| MCC11 | BAMBI | p.D240N | rqadlsnDk | rqadlsnNk | A0301 | 400 |
| MCC11 | BCAS3 | p.E81K | tsrnlefhE | tsrnlefhK | A0301 | 291 |
| MCC11 | BSPRY | p.T251I | kTltfstkk | kIltfstkk | A0301 | 26 |
| MCC11 | BTBD11 | p.P483S | vllftasPr | vllftasSr | A0301 | 250 |
| MCC11 | C10orf118 | p.D472H | Dqlemhhak | Hqlemhhak | A0301 | 451 |
| MCC11 | C10orf2 | p.R196Q | tlkrfsvRy | tlkrfsvQy | A0301 | 395 |
| MCC11 | C10orf76 | p.S14F | llrkSsask | llrkFsask | A0301 | 29 |
| MCC11 | C15orf53 | p.P136S | Pvflrpsdr | Svflrpsdr | A0301 | 343 |
| MCC11 | C16orf92 | p.E36K | atasalgtE | atasalgtK | A0301 | 113 |
| MCC11 | C1QTNF1 | p.I138M | Ilfaqvgdr | Mlfaqvgdr | A0301 | 80 |
| MCC11 | C22orf24 | p.E142K | alrepgrsE | alrepgrsK | A0301 | 63 |
| MCC11 | C2orf53 | p.L312V | ralhLlpek | ralhVlpek | A0301 | 144 |
| MCC11 | C5orf42 | p.Q1297H | lsyscrqyQ | lsyscrqyH | A0301 | 497 |
| MCC11 | C5orf58 | p.P69A | ilhfnhPik | ilhfnhAik | A0301 | 41 |
| MCC11 | C9orf96 | p.E599K | gsglslikE | gsglslikK | A0301 | 342 |
| MCC11 | CACNA1A | p.R588H | llRifkvtk | llHifkvtk | A0301 | 148 |
| MCC11 | CACNA1A | p.R588H | vlralrllR | vlralrllH | A0301 | 144 |
| MCC11 | CACNA1A | p.R588H | alrllRifk | alrllHifk | A0301 | 31 |
| MCC11 | CACNA1C | p.E917K | lssislaaE | lssislaaK | A0301 | 338 |
| MCC11 | CACNA1E | p.E1724K | ilgphhldE | ilgphhldK | A0301 | 59 |
| MCC11 | CADM3 | p.R118Q | ftmpvRtak | ftmpvQtak | A0301 | 94 |
| MCC11 | CASZ1 | p.I635M | hIkddayak | hMkddayak | A0301 | 425 |
| MCC11 | CCBL1 | p.I333M | Ifysvphqk | Mfysvphqk | A0301 | 54 |
| MCC11 | CCK | p.P73L | Psgrmsivk | Lsgrmsivk | A0301 | 264 |
| MCC11 | CCSAP | p.D242Y | rahsvDvek | rahsvYvek | A0301 | 299 |
| MCC11 | CCSAP | p.D242Y | svDveknrk | svYveknrk | A0301 | 122 |
| MCC11 | CECR1 | p.A188V | sirmAmglr | sirmVmglr | A0301 | 456 |
| MCC11 | CECR1 | p.A188V | rmAmglrik | rmVmglrik | A0301 | 131 |
| MCC11 | CFHR5 | p.P90L | rmcsfPfvk | rmcsfLfvk | A0301 | 27 |
| MCC11 | CHRM2 | p.K370R | kmtKqpakk | kmtRqpakk | A0301 | 44 |
| MCC11 | CHRM2 | p.K370R | mtKqpakkk | mtRqpakkk | A0301 | 141 |
| MCC11 | CLCA2 | p.P621L | Pvmiyanvk | Lvmiyanvk | A0301 | 234 |
| MCC11 | CLEC4D | p.G144R | rlsyflGlr | rlsyflRlr | A0301 | 188 |
| MCC11 | COL6A3 | p.A133V | hltkAagsr | hltkVagsr | A0301 | 489 |
| MCC11 | COL6A5 | p.S282I | lmsysnSak | lmsysnIak | A0301 | 44 |
| MCC11 | COL6A5 | p.S282I | Saktisflk | Iaktisflk | A0301 | 114 |
| MCC11 | COPS2 | p.E109K | avtrnysEk | avtrnysKk | A0301 | 140 |
| MCC11 | COPZ2 | p.S143T | hmlrknvEk | hmlrknvTk | A0301 | 42 |
| MCC11 | COPZ2 | p.S143T | mlrknvEkr | mlrknvTkr | A0301 | 170 |
| MCC11 | CPB1 | p.R291C | aladfiRnk | aladfiCnk | A0301 | 41 |
| MCC11 | CRISPLD1 | p.R94K | mtwdveleR | mtwdveleK | A0301 | 188 |
| MCC11 | CUBN | p.E975K | Etfhlefhy | Ktfhlefhy | A0301 | 94 |
| MCC11 | DCSTAMP | p.L87F | lvfLscglr | lvfFscglr | A0301 | 221 |
| MCC11 | DENND3 | p.P395L | aPgdhqfyk | aLgdhqfyk | A0301 | 45 |
| MCC11 | DEPDC7 | p.E197K | vinevwqeE | vinevwqeK | A0301 | 297 |
| MCC11 | DNAH10 | p.E2018K | ksvlvmagE | ksvlvmagK | A0301 | 86 |
| MCC11 | DNAH10 | p.E2018K | vlvmagElk | vlvmagKlk | A0301 | 471 |
| MCC11 | DNAH10 | p.D2376H | rlaslstvD | rlaslstvH | A0301 | 156 |
| MCC11 | DNAH14 | p.R1842I | ttvRrilek | ttvIrilek | A0301 | 213 |
| MCC11 | DNAJC16 | p.Q639H | stktsllQk | stktsllHk | A0301 | 72 |
| MCC11 | DOCK2 | p.M535I | amsyvklMk | amsyvklIk | A0301 | 55 |
| MCC11 | DOCK4 | p.E1192Q | nfyktElnk | nfyktQlnk | A0301 | 309 |
| MCC11 | DPRX | p.E48K | slqkEmask | slqkKmask | A0301 | 80 |
| MCC11 | DPY19L4 | p.S301F | yifSlflgy | yifFlflgy | A0301 | 173 |
| MCC11 | DPY19L4 | p.S301F | kiyifSlfl | kiyifFlfl | A0301 | 363 |
| MCC11 | DSCC1 | p.G97R | mllfipGck | mllfipRck | A0301 | 38 |
| MCC11 | ECT2L | p.F844L | Fiasvalhr | Liasvalhr | A0301 | 396 |
| MCC11 | ELOVL2 | p.L145F | itfLhvyhh | itfFhvyhh | A0301 | 422 |
| MCC11 | ELOVL2 | p.L145F | ktsqitfLh | ktsqitfFh | A0301 | 150 |
| MCC11 | EPB41L3 | p.S92F | lsqksSssk | lsqksFssk | A0301 | 118 |
| MCC11 | EPB41L3 | p.S92F | ksSssklsr | ksFssklsr | A0301 | 70 |
| MCC11 | EPHA6 | p.F297L | lvsvrvFyk | lvsvrvLyk | A0301 | 33 |
| MCC11 | EPHA6 | p.F297L | vsvrvFykk | vsvrvLykk | A0301 | 395 |
| MCC11 | EPHA6 | p.F297L | alvsvrvFy | alvsvrvLy | A0301 | 298 |
| MCC11 | EPS15 | p.D270A | hiwslcDtk | hiwslcAtk | A0301 | 70 |
| MCC11 | ESPL1 | p.D206H | avaahqlfD | avaahqlfH | A0301 | 61 |
| MCC11 | EXOC2 | p.R336T | alRellldk | alTellldk | A0301 | 206 |
| MCC11 | FAM135A | p.P592S | rlfPqllmk | rlfSqllmk | A0301 | 17 |
| MCC11 | FAM189A1 | p.D411H | smagDassh | smagHassh | A0301 | 127 |
| MCC11 | FCGBP | p.R324K | llfgtgaiR | llfgtgaiK | A0301 | 30 |
| MCC11 | FEZF2 | p.H407Q | Hmhthndkk | Qmhthndkk | A0301 | 395 |
| MCC11 | FGB | p.E144K | silEnlrsk | silKnlrsk | A0301 | 133 |
| MCC11 | FOCAD | p.E1208Q | tEaedvmnk | tQaedvmnk | A0301 | 323 |
| MCC11 | FRMPD1 | p.S233F | siSrlhllh | siFrlhllh | A0301 | 144 |
| MCC11 | FRY | p.G704R | klviqtqGk | klviqtqRk | A0301 | 58 |
| MCC11 | FRY | p.E2407K | hvlslcgqE | hvlslcgqK | A0301 | 81 |
| MCC11 | FSHR | p.M302K | ssysrgfdM | ssysrgfdK | A0301 | 31 |
| MCC11 | FXR1 | p.E410K | Eshhstnrr | Kshhstnrr | A0301 | 495 |
| MCC11 | GALNT12 | p.S217F | rllgaSaar | rllgaFaar | A0301 | 125 |
| MCC11 | GAPVD1 | p.S928L | hsssSspsk | hsssLspsk | A0301 | 94 |
| MCC11 | GDF9 | p.E408K | mvqniiyEk | mvqniiyKk | A0301 | 152 |
| MCC11 | GDF9 | p.E408K | tmvqniiyE | tmvqniiyK | A0301 | 54 |
| MCC11 | GDPD4 | p.S356F | vvsvilaSk | vvsvilaFk | A0301 | 90 |
| MCC11 | GHSR | p.E291K | rylfsksfE | rylfsksfK | A0301 | 125 |
| MCC11 | GPC6 | p.E390Q | kEklklskk | kQklklskk | A0301 | 157 |
| MCC11 | GPC6 | p.E390Q | rlvtdikEk | rlvtdikQk | A0301 | 208 |
| MCC11 | GPR39 | p.P149S | avsgPcqvk | avsgScqvk | A0301 | 220 |
| MCC11 | GRID1 | p.E446K | tlkvvtvlE | tlkvvtvlK | A0301 | 52 |
| MCC11 | GRK4 | p.M347I | Miqghspfk | Iiqghspfk | A0301 | 36 |
| MCC11 | GSAP | p.R274L | hqyRdkfsk | hqyLdkfsk | A0301 | 80 |
| MCC11 | GTPBP8 | p.I127M | nvgksslIk | nvgksslMk | A0301 | 331 |
| MCC11 | HAO1 | p.E197K | stlsfspeE | stlsfspeK | A0301 | 108 |
| MCC11 | HCN4 | p.F470L | kqysyalFk | kqysyalLk | A0301 | 18 |
| MCC11 | HHLA1 | p.E183K | ilsvnqsnE | ilsvnqsnK | A0301 | 28 |
| MCC11 | IAH1 | p.E138K | litptplcE | litptplcK | A0301 | 443 |
| MCC11 | IGSF22 | p.H568Q | ivkqgavHk | ivkqgavQk | A0301 | 419 |
| MCC11 | INSL6 | p.E70K | rliaqasEk | rliaqasKk | A0301 | 22 |
| MCC11 | IRGC | p.E337K | virsplanE | virsplanK | A0301 | 39 |
| MCC11 | JAKMIP2 | p.E722K | rqmlrksrE | rqmlrksrK | A0301 | 39 |
| MCC11 | JAKMIP3 | p.E74K | vlltElktk | vlltKlktk | A0301 | 127 |
| MCC11 | KCNIP1 | p.K14T | vKfaqtifk | vTfaqtifk | A0301 | 18 |
| MCC11 | KCNJ5 | p.E246K | liksrqtkE | liksrqtkK | A0301 | 78 |
| MCC11 | KCNK18 | p.G376E | vmlffakGk | vmlffakEk | A0301 | 81 |
| MCC11 | KCNQ1 | p.G102S | Girflqilr | Sirflqilr | A0301 | 345 |
| MCC11 | KCNQ3 | p.P289S | svvsfPffr | svvsfSffr | A0301 | 269 |
| MCC11 | KCNQ3 | p.P289S | vvsfPffrk | vvsfSffrk | A0301 | 31 |
| MCC11 | KIAA1024 | p.R706K | slftRpssr | slftKpssr | A0301 | 54 |
| MCC11 | KIAA1024 | p.R706K | alkkslftR | alkkslftK | A0301 | 38 |
| MCC11 | KLK11 | p.E193K | tmvcasvqE | tmvcasvqK | A0301 | 237 |
| MCC11 | KNTC1 | p.E1325Q | rEnattllh | rQnattllh | A0301 | 248 |
| MCC11 | LAMA3 | p.E816Q | kEiiflpsk | kQiiflpsk | A0301 | 170 |
| MCC11 | LAMB4 | p.K1323N | hisssaeKk | hisssaeNk | A0301 | 99 |
| MCC11 | LAMC1 | p.D1531H | qldtvDlnk | qldtvHlnk | A0301 | 90 |
| MCC11 | LCA5 | p.R103Q | Rilsarllk | Qilsarllk | A0301 | 74 |
| MCC11 | LEF1 | p.G362R | klqesasgG | klqesasgR | A0301 | 397 |
| MCC11 | LMAN1L | p.M73I | rltpsMrnr | rltpsIrnr | A0301 | 465 |
| MCC11 | LOC81691 | p.R449T | ssRncqtik | ssTncqtik | A0301 | 379 |
| MCC11 | LRBA | p.P478L | Plfaqldyr | Llfaqldyr | A0301 | 449 |
| MCC11 | LYST | p.S1432L | rarvSrskk | rarvLrskk | A0301 | 136 |
| MCC11 | MAST2 | p.S578F | smfcSfdtk | smfcFfdtk | A0301 | 36 |
| MCC11 | MB21D1 | p.L311F | avtlLisek | avtlFisek | A0301 | 183 |
| MCC11 | MDH1 | p.E115K | gmErkdllk | gmKrkdllk | A0301 | 134 |
| MCC11 | MDH1 | p.E115K | smprregmE | smprregmK | A0301 | 188 |
| MCC11 | MOB1A | p.F156L | ktilkrlFr | ktilkrlLr | A0301 | 453 |
| MCC11 | MROH2B | p.S1075F | aiSqiasfh | aiFqiasfh | A0301 | 389 |
| MCC11 | MRPS35 | p.E302K | ksvvslknE | ksvvslknK | A0301 | 272 |
| MCC11 | MYH13 | p.E1380K | vaqwrtkyE | vaqwrtkyK | A0301 | 456 |
| MCC11 | MYH14 | p.S289L | kSrairqak | kLrairqak | A0301 | 28 |
| MCC11 | MYH4 | p.M365I | avmhygnMk | avmhygnIk | A0301 | 67 |
| MCC11 | MYO1F | p.E606Q | hqvEylglk | hqvQylglk | A0301 | 471 |
| MCC11 | MYPN | p.Q104H | rlspdQmkh | rlspdHmkh | A0301 | 431 |
| MCC11 | NAA25 | p.L88F | slqaltiLy | slqaltiFy | A0301 | 87 |
| MCC11 | NAA25 | p.L88F | tiLyremhr | tiFyremhr | A0301 | 140 |
| MCC11 | NEB | p.R668T | knfseaRyk | knfseaTyk | A0301 | 243 |
| MCC11 | NEB | p.R668T | aRykdlyvk | aTykdlyvk | A0301 | 61 |
| MCC11 | NEFM | p.G9S | slGnpsayr | slSnpsayr | A0301 | 102 |
| MCC11 | NEK7 | p.L119I | Lsrmikhfk | Isrmikhfk | A0301 | 99 |
| MCC11 | NOS3 | p.Q532H | Qsyaqqlgr | Hsyaqqlgr | A0301 | 65 |
| MCC11 | NR4A1 | p.E398K | lvlphfgkE | lvlphfgkK | A0301 | 39 |
| MCC11 | OCIAD2 | p.P28L | kqsllfcPk | kqsllfcLk | A0301 | 84 |
| MCC11 | OCIAD2 | p.P28L | sllfcPksk | sllfcLksk | A0301 | 43 |
| MCC11 | OR10V1 | p.P211S | Plslisisy | Slslisisy | A0301 | 194 |
| MCC11 | OR2L13 | p.A236V | kAfttisth | kVfttisth | A0301 | 158 |
| MCC11 | OR4C16 | p.P56S | nPmffflfy | nSmffflfy | A0301 | 333 |
| MCC11 | OR4K5 | p.D52H | Dtslhspmy | Htslhspmy | A0301 | 363 |
| MCC11 | OR4K5 | p.D52H | tvtsDtslh | tvtsHtslh | A0301 | 486 |
| MCC11 | OR6C1 | p.E109K | illgvtEfy | illgvtKfy | A0301 | 267 |
| MCC11 | OR6C75 | p.T222K | lsytniirT | lsytniirK | A0301 | 23 |
| MCC11 | OR6M1 | p.R290S | fiyslRnek | fiyslSnek | A0301 | 69 |
| MCC11 | PAK7 | p.R554K | atvclsvlR | atvclsvlK | A0301 | 301 |
| MCC11 | PCSK6 | p.V169L | mWylhcgdk | mLylhcgdk | A0301 | 17 |
| MCC11 | PEG3 | p.R264K | vsrkrvleR | vsrkrvleK | A0301 | 49 |
| MCC11 | PENK | p.R209Q | kRyggfmrr | kQyggfmrr | A0301 | 34 |
| MCC11 | PEX5L | p.E28K | mvmkeiprE | mvmkeiprK | A0301 | 47 |
| MCC11 | PGAP1 | p.S704F | gllssaSvr | gllssaFvr | A0301 | 324 |
| MCC11 | PHF3 | p.E897K | kaskpgthE | kaskpgthK | A0301 | 108 |
| MCC11 | PIK3R6 | p.E175K | svcsalllE | svcsalllK | A0301 | 80 |
| MCC11 | PKNOX1 | p.P65S | Pllallfek | Sllallfek | A0301 | 45 |
| MCC11 | PLA2R1 | p.T1383I | hTaealpek | hIaealpek | A0301 | 170 |
| MCC11 | PLCE1 | p.S1943C | iSfaselkk | iCfaselkk | A0301 | 155 |
| MCC11 | PLD2 | p.E443Q | vtlwahhEk | vtlwahhQk | A0301 | 196 |
| MCC11 | PNLDC1 | p.P413S | kinfsgPdy | kinfsgSdy | A0301 | 152 |
| MCC11 | POLR1A | p.E1055K | Evlsradpk | Kvlsradpk | A0301 | 80 |
| MCC11 | POLR1A | p.E1055K | imksqhlhE | imksqhlhK | A0301 | 31 |
| MCC11 | PPIP5K2 | p.P254S | kvytvgPdy | kvytvgSdy | A0301 | 69 |
| MCC11 | PRKCA | p.P202S | klkliPdpk | klkliSdpk | A0301 | 111 |
| MCC11 | PWP1 | p.F444L | Fiyafggqk | Liyafggqk | A0301 | 24 |
| MCC11 | PYGO1 | p.P111S | rmpphvPpr | rmpphvSpr | A0301 | 307 |
| MCC11 | RAB37 | p.V100I | rsVthayyr | rsIthayyr | A0301 | 383 |
| MCC11 | RAP1GDS1 | p.E314Q | tEavlkflk | tQavlkflk | A0301 | 452 |
| MCC11 | RBBP6 | p.S1000L | itfkSvsek | itfkLvsek | A0301 | 37 |
| MCC11 | RBM20 | p.I588F | kllIrmskr | kllFrmskr | A0301 | 26 |
| MCC11 | RBM20 | p.I588F | llIrmskry | llFrmskry | A0301 | 325 |
| MCC11 | RBM27 | p.S798L | ssnlktpSk | ssnlktpLk | A0301 | 101 |
| MCC11 | RBM28 | p.P533S | alPshrgpk | alSshrgpk | A0301 | 36 |
| MCC11 | RBM28 | p.P533S | kvlalPshr | kvlalSshr | A0301 | 129 |
| MCC11 | RBM45 | p.E43K | ytpesvlrE | ytpesvlrK | A0301 | 409 |
| MCC11 | RC3H1 | p.E1111K | ktsslnlsE | ktsslnlsK | A0301 | 51 |
| MCC11 | RFX8 | p.F286C | klaasFqlr | klaasCqlr | A0301 | 90 |
| MCC11 | RGS7 | p.R44C | Rtvksflsk | Ctvksflsk | A0301 | 54 |
| MCC11 | RMND5B | p.S62L | lvmSqccrk | lvmLqccrk | A0301 | 238 |
| MCC11 | RMND5B | p.S62L | mSqccrkik | mLqccrkik | A0301 | 225 |
| MCC11 | RP1 | p.S1544F | sldfcydSk | sldfcydFk | A0301 | 115 |
| MCC11 | SEL1L2 | p.L308I | gqlhLigrk | gqlhIigrk | A0301 | 233 |
| MCC11 | SEL1L2 | p.E176K | slakEgsck | slakKgsck | A0301 | 47 |
| MCC11 | SEL1L2 | p.E176K | qlyeslakE | qlyeslakK | A0301 | 25 |
| MCC11 | SETD4 | p.V280G | yvsreilVk | yvsreilGk | A0301 | 244 |
| MCC11 | SLC13A2 | p.E347K | lvllwftrE | lvllwftrK | A0301 | 87 |
| MCC11 | SLC22A16 | p.P436L | gvvmviPqk | gvvmviLqk | A0301 | 150 |
| MCC11 | SLC2A2 | p.S145L | gallmgfSk | gallmgfLk | A0301 | 356 |
| MCC11 | SLC44A1 | p.E369K | ttgspvqnE | ttgspvqnK | A0301 | 167 |
| MCC11 | SLC5A11 | p.M441K | ssssvqfeM | ssssvqfeK | A0301 | 165 |
| MCC11 | SLC5A5 | p.E619K | rlfflgqkE | rlfflgqkK | A0301 | 20 |
| MCC11 | SMO | p.I185M | ctnevqnIk | ctnevqnMk | A0301 | 222 |
| MCC11 | SND1 | p.Q882E | ylnaQesak | ylnaEesak | A0301 | 148 |
| MCC11 | SPATA16 | p.E204Q | rtalElcsk | rtalQlcsk | A0301 | 74 |
| MCC11 | SPRED1 | p.R16Q | Rvravvmtr | Qvravvmtr | A0301 | 427 |
| MCC11 | SPTA1 | p.E1898K | sqnkEissk | sqnkKissk | A0301 | 118 |
| MCC11 | SPTB | p.G106E | mlpkptkGk | mlpkptkEk | A0301 | 171 |
| MCC11 | SRGAP1 | p.E72K | klaErfmak | klaKrfmak | A0301 | 13 |
| MCC11 | ST7L | p.P410A | ilpPehilk | ilpAehilk | A0301 | 105 |
| MCC11 | STARD5 | p.K16N | avaeKmlqy | avaeNmlqy | A0301 | 412 |
| MCC11 | SUGP1 | p.M457I | qmydMimqh | qmydIimqh | A0301 | 104 |
| MCC11 | TCF3 | p.L98F | flgpgLggk | flgpgFggk | A0301 | 207 |
| MCC11 | TEKT5 | p.P91S | alfsrysPh | alfsrysSh | A0301 | 100 |
| MCC11 | TFCP2L1 | p.L26V | dvlaLpifk | dvlaVpifk | A0301 | 369 |
| MCC11 | TGFBRAP1 | p.E439K | ylnevrstE | ylnevrstK | A0301 | 52 |
| MCC11 | TGFBRAP1 | p.E439K | stEvangyk | stKvangyk | A0301 | 287 |
| MCC11 | THSD7A | p.E484K | qlsthknkE | qlsthknkK | A0301 | 98 |
| MCC11 | THSD7A | p.E484K | Easkpmdlk | Kaskpmdlk | A0301 | 195 |
| MCC11 | TLN2 | p.E850K | Eidmenskk | Kidmenskk | A0301 | 317 |
| MCC11 | TLN2 | p.E850K | amrsdaeaE | amrsdaeaK | A0301 | 356 |
| MCC11 | TMEM44 | p.L50V | llyLrcaqk | llyVrcaqk | A0301 | 21 |
| MCC11 | TNKS2 | p.P280S | tPlheaask | tSlheaask | A0301 | 356 |
| MCC11 | TNNI3K | p.E657K | fsyalclwE | fsyalclwK | A0301 | 36 |
| MCC11 | TNNT1 | p.P38S | vvpPlippk | vvpSlippk | A0301 | 459 |
| MCC11 | TOPBP1 | p.P889L | alsasPqlk | alsasLqlk | A0301 | 31 |
| MCC11 | TOPORS | p.S605L | rSrsrssdh | rLrsrssdh | A0301 | 115 |
| MCC11 | TP53 | p.R117P | Rvramaiyk | Pvramaiyk | A0301 | 152 |
| MCC11 | TRAPPC13 | p.K389N | rltdtflKr | rltdtflNr | A0301 | 297 |
| MCC11 | TRHDE | p.E772K | klldrmEny | klldrmKny | A0301 | 193 |
| MCC11 | TRPM6 | p.S1344C | fllvpSnlk | fllvpCnlk | A0301 | 185 |
| MCC11 | TSHZ2 | p.S811F | ssSrvppmk | ssFrvppmk | A0301 | 36 |
| MCC11 | TSTA3 | p.S100L | hSafevgar | hLafevgar | A0301 | 435 |
| MCC11 | TSTA3 | p.S100L | Safevgark | Lafevgark | A0301 | 225 |
| MCC11 | TTLL3 | p.P842S | pmkrlsPlk | pmkrlsSlk | A0301 | 175 |
| MCC11 | TTN | p.R13504Q | Rlkipikgk | Qlkipikgk | A0301 | 454 |
| MCC11 | TXLNB | p.E429Q | kEyecfvmk | kQyecfvmk | A0301 | 34 |
| MCC11 | TXLNB | p.G78E | gsaastaGk | gsaastaEk | A0301 | 124 |
| MCC11 | UBE3A | p.D663Y | Dlfgnpmmy | Ylfgnpmmy | A0301 | 61 |
| MCC11 | USP13 | p.S172F | lsskSpyrk | lsskFpyrk | A0301 | 257 |
| MCC11 | USP13 | p.S172F | vlsskSpyr | vlsskFpyr | A0301 | 106 |
| MCC11 | UTRN | p.D2301H | Dmrtaitek | Hmrtaitek | A0301 | 44 |
| MCC11 | VCAM1 | p.E128K | Eagkpitvk | Kagkpitvk | A0301 | 132 |
| MCC11 | WDR47 | p.E588K | gslsrskgE | gslsrskgK | A0301 | 329 |
| MCC11 | WDR52 | p.E1120Q | iivenqiEk | iivenqiQk | A0301 | 184 |
| MCC11 | XAB2 | p.G498S | slGtfqstk | slStfqstk | A0301 | 25 |
| MCC11 | YWHAQ | p.L172I | aLnfsvfyy | aInfsvfyy | A0301 | 57 |
| MCC11 | ZAP70 | p.E386K | mmrEaqimh | mmrKaqimh | A0301 | 209 |
| MCC11 | ZCCHC11 | p.Q213H | lqnsprsQk | lqnsprsHk | A0301 | 88 |
| MCC11 | ZDBF2 | p.M2339I | rmMtrlank | rmItrlank | A0301 | 19 |
| MCC11 | ZFAT | p.E801K | evsmstisE | evsmstisK | A0301 | 387 |
| MCC11 | ZFYVE26 | p.C2242Y | aaCqhlqkk | aaYqhlqkk | A0301 | 64 |
| MCC11 | ZFYVE26 | p.C2242Y | iaaCqhlqk | iaaYqhlqk | A0301 | 266 |
| MCC11 | ZNF20 | p.K222N | rihtgvKpy | rihtgvNpy | A0301 | 451 |
| MCC11 | ZNF326 | p.S315L | maftcSfck | maftcLfck | A0301 | 37 |
| MCC11 | ZNF436 | p.D18H | vtfeDmamy | vtfeHmamy | A0301 | 203 |
| MCC11 | ZNF566 | p.H221Y | hqkiHtgkk | hqkiYtgkk | A0301 | 308 |
| MCC11 | ZNF572 | p.I344N | ssnlIthqk | ssnlNthqk | A0301 | 326 |
| MCC11 | ZNF573 | p.G238C | hqrvhtGkk | hqrvhtCkk | A0301 | 120 |
| MCC11 | ZNF641 | p.P129S | iingfPipk | iingfSipk | A0301 | 38 |
| MCC11 | ZNF90 | p.L272F | ysstLtahk | ysstFtahk | A0301 | 154 |
| MCC15 | ABCA1 | p.A697D | llvsAgllv | llvsDgllv | A0201 | 81 |
| MCC15 | ABCA1 | p.A697D | lvsAgllvv | lvsDgllvv | A0201 | 74 |
| MCC15 | ABCC9 | p.R1154W | yfiqkyfRv | yfiqkyfWv | A0201 | 164 |
| MCC15 | ABCC9 | p.R1154W | fiqkyfRva | fiqkyfWva | A0201 | 39 |
| MCC15 | ATXN7L2 | p.K336M | aKqtypyca | aMqtypyca | A0201 | 42 |
| MCC15 | ATXN7L2 | p.K336M | fsvraKqty | fsvraMqty | B1501 | 52 |
| MCC15 | ATXN7L2 | p.K336M | Kqtypycal | Mqtypycal | B1501 | 390 |
| MCC15 | CLIC6 | p.I634V | hiikIvakk | hiikVvakk | A0301 | 52 |
| MCC15 | ITGA2B | p.P972R | slpyavpPl | slpyavpRl | A0201 | 52 |
| MCC15 | MAML1 | p.T300M | kTefspaaf | kMefspaaf | B1501 | 273 |
| MCC15 | MAP3K9 | p.T142I | ltTieesgf | ltIieesgf | B1501 | 472 |
| MCC15 | MAP3K9 | p.T142I | tTieesgff | tIieesgff | B1501 | 174 |
| MCC15 | SRBD1 | p.Q688K | yqhdvsQtl | yqhdvsKtl | B1501 | 56 |
| MCC15 | SRBD1 | p.Q688K | yqhdvsQtl | yqhdvsKtl | A0201 | 498 |
| MCC15 | SRBD1 | p.Q688K | gmyqhdvsQ | gmyqhdvsK | A0301 | 32 |
| MCC15 | TTC39B | p.V230L | lVlfyhari | lLlfyhari | A0201 | 67 |
| MCC15 | TTC39B | p.V230L | qqfpngslV | qqfpngslL | B1501 | 159 |
| MCC16 | NCOA2 | p.P573A | nmnppPlsk | nmnppAlsk | A0301 | 53 |
| MCC16 | PTPN23 | p.Q1499K | ssiQatiak | ssiKatiak | A0301 | 147 |
| MCC19 | SPTBN1 | p.R1728W | mlqerfRef | mlqerfWef | A0201 | 106 |
| MCC19 | SPTBN1 | p.R1728W | mlqerfRef | mlqerfWef | A2402 | 119 |
| MCC20 | DDX19A | p.K236M | iKvfvldea | iMvfvldea | A0201 | 142 |
| MCC20 | DDX19A | p.K236M | fidpkkiKv | fidpkkiMv | A0201 | 59 |
| MCC20 | ERMP1 | p.V851F | wqfwievqV | wqfwievqF | A2301 | 476 |
| MCC20 | NAV2 | p.P1855L | sqvsisasP | sqvsisasL | A0201 | 260 |
| MCC21 | ABCA13 | p.H4970L | Hlklyfpgi | Llklyfpgi | A0201 | 339 |
| MCC21 | ABCC11 | p.G1160S | vlhGinlti | vlhSinlti | A0201 | 55 |
| MCC21 | ACTL6B | p.E135K | Elmfeqyni | Klmfeqyni | A0201 | 7 |
| MCC21 | ADAMTS19 | p.G1166E | kclGdqwpv | kclEdqwpv | A0201 | 33 |
| MCC21 | AGL | p.S1275F | Savrwllel | Favrwllel | A0201 | 71 |
| MCC21 | ATP10B | p.T873A | llmeTaqhl | llmeAaqhl | A0201 | 10 |
| MCC21 | BBS1 | p.P83S | vmtesplPa | vmtesplSa | A0201 | 91 |
| MCC21 | BRSK1 | p.L221F | gviLfallv | gviFfallv | A0201 | 133 |
| MCC21 | C12orf42 | p.P86S | slhflnfPv | slhflnfSv | A0201 | 9 |
| MCC21 | C9orf116 | p.S92L | smftwrkaS | smftwrkaL | A0201 | 332 |
| MCC21 | CC2D2B | p.I308M | nIlsvwvyl | nMlsvwvyl | A0201 | 14 |
| MCC21 | CC2D2B | p.I308M | Ilsvwvyla | Mlsvwvyla | A0201 | 8 |
| MCC21 | CHN2 | p.P224S | klyfrdlPi | klyfrdlSi | A0201 | 71 |
| MCC21 | CHRNB3 | p.G441S | livsvtGsv | livsvtSsv | A0201 | 72 |
| MCC21 | CILP | p.D704N | klwslnpDt | klwslnpNt | A0201 | 32 |
| MCC21 | CLSTN2 | p.E873K | Emdwddsal | Kmdwddsal | A0201 | 128 |
| MCC21 | CYP4F2 | p.E328K | fmfEghdtt | fmfKghdtt | A0201 | 251 |
| MCC21 | E2F7 | p.P496S | Plahpvfsv | Slahpvfsv | A0201 | 8 |
| MCC21 | EEF1G | p.S286N | hlpkStfvl | hlpkNtfvl | A0201 | 331 |
| MCC21 | ETFA | p.D187N | llyDladql | llyNladql | A0201 | 97 |
| MCC21 | ETFA | p.D187N | Dladqlhaa | Nladqlhaa | A0201 | 39 |
| MCC21 | EXD1 | p.Q473E | smssfpQet | smssfpEet | A0201 | 457 |
| MCC21 | FAM73B | p.F495L | hFysvsehv | hLysvsehv | A0201 | 24 |
| MCC21 | FAM73B | p.F495L | gfishFysv | gfishLysv | A0201 | 269 |
| MCC21 | FAT1 | p.S4375L | slssfqseS | slssfqseL | A0201 | 28 |
| MCC21 | HEATR6 | p.V97L | kVsqlihhl | kLsqlihhl | A0201 | 13 |
| MCC21 | HEATR6 | p.V97L | hlvskVsql | hlvskLsql | A0201 | 371 |
| MCC21 | LAMA1 | p.P167L | clsrynitP | clsrynitL | A0201 | 91 |
| MCC21 | LGR6 | p.P150L | iPdyafqnl | iLdyafqnl | A0201 | 72 |
| MCC21 | LGR6 | p.P150L | alnrishiP | alnrishiL | A0201 | 260 |
| MCC21 | LPPR5 | p.L206V | Ltmyitnti | Vtmyitnti | A0201 | 319 |
| MCC21 | LPPR5 | p.L206V | amyLtmyit | amyVtmyit | A0201 | 422 |
| MCC21 | LPPR5 | p.L206V | aamyLtmyi | aamyVtmyi | A0201 | 98 |
| MCC21 | MAML2 | p.P678L | Pllrsplpl | Lllrsplpl | A0201 | 21 |
| MCC21 | MUC16 | p.L13327F | vllpftLnf | vllpftFnf | A0201 | 105 |
| MCC21 | NALCN | p.E1016K | Eiflvsill | Kiflvsill | A0201 | 124 |
| MCC21 | NPR2 | p.S942F | alldavSsf | alldavFsf | A0201 | 49 |
| MCC21 | OR2L2 | p.H53Y | llifldiHl | llifldiYl | A0201 | 18 |
| MCC21 | OR2L2 | p.H53Y | Hlhtpmyfl | Ylhtpmyfl | A0201 | 5 |
| MCC21 | PAQR7 | p.G145R | fldyvGvav | fldyvRvav | A0201 | 11 |
| MCC21 | PIEZO2 | p.G692E | yvcGgmfff | yvcEgmfff | A0201 | 452 |
| MCC21 | PIK3CG | p.V667L | yllqlvqaV | yllqlvqaL | A0201 | 10 |
| MCC21 | PLCB4 | p.T813A | slpTifcni | slpAifcni | A0201 | 93 |
| MCC21 | PLCZ1 | p.L284V | mlddfpdtL | mlddfpdtV | A0201 | 6 |
| MCC21 | RET | p.S457L | tSaedtsgi | tLaedtsgi | A0201 | 51 |
| MCC21 | RHBDF1 | p.S415L | hSlvtilav | hLlvtilav | A0201 | 32 |
| MCC21 | RHBDF1 | p.S415L | ywltfvhSl | ywltfvhLl | A0201 | 421 |
| MCC21 | RHBDF1 | p.S415L | wltfvhSlv | wltfvhLlv | A0201 | 74 |
| MCC21 | RNF216 | p.P774L | kPvekvqrv | kLvekvqrv | A0201 | 13 |
| MCC21 | RNF26 | p.S164F | nlfSlvlal | nlfFlvlal | A0201 | 51 |
| MCC21 | RNF26 | p.S164F | gtqnlfSlv | gtqnlfFlv | A0201 | 166 |
| MCC21 | RNF26 | p.S164F | Slvlalwda | Flvlalwda | A0201 | 25 |
| MCC21 | RQCD1 | p.P131L | rPfeylrlt | rLfeylrlt | A0201 | 56 |
| MCC21 | RTF1 | p.S354F | Slpeelnrv | Flpeelnrv | A0201 | 6 |
| MCC21 | SLC22A17 | p.L165P | fllgflLag | fllgflPag | A0201 | 39 |
| MCC21 | SLC22A17 | p.L165P | llgflLagv | llgflPagv | A0201 | 46 |
| MCC21 | SLC22A24 | p.M490I | llmtlMays | llmtlIays | A0201 | 60 |
| MCC21 | SLC22A24 | p.M490I | tlMaysphl | tlIaysphl | A0201 | 8 |
| MCC21 | SLC26A3 | p.P59S | rivlslfPi | rivlslfSi | A0201 | 211 |
| MCC21 | SLC26A3 | p.P59S | slfPiaswl | slfSiaswl | A0201 | 19 |
| MCC21 | SLC2A7 | p.G88D | smfplgGll | smfplgDll | A0201 | 44 |
| MCC21 | SLIT1 | p.S590F | Svselhlta | Fvselhlta | A0201 | 142 |
| MCC21 | SLIT1 | p.S590F | gafegaaSv | gafegaaFv | A0201 | 83 |
| MCC21 | SPOPL | p.L308F | lvLadlhsa | lvFadlhsa | A0201 | 109 |
| MCC21 | SYT4 | p.G155E | klGtlffsl | klEtlffsl | A0201 | 23 |
| MCC21 | TBCEL | p.T142M | asweTvhmi | asweMvhmi | A0201 | 310 |
| MCC21 | TBCEL | p.T142M | Tvhmilqel | Mvhmilqel | A0201 | 455 |
| MCC21 | TMEM87A | p.L476F | Lldsdeerm | Fldsdeerm | A0201 | 308 |
| MCC21 | TMPRSS9 | p.G729S | rlkGwilei | rlkSwilei | A0201 | 62 |
| MCC21 | TPK1 | p.V146A | cmqVtttgl | cmqAtttgl | A0201 | 277 |
| MCC21 | TRERF1 | p.P878L | hPlanyhya | hLlanyhya | A0201 | 27 |
| MCC21 | TRPM5 | p.V796M | lVkkftlyv | lMkkftlyv | A0201 | 31 |
| MCC21 | TRPM5 | p.V796M | ftdedthlV | ftdedthlM | A0201 | 144 |
| MCC21 | TYK2 | p.P69L | gitpPcfnl | gitpLcfnl | A0201 | 329 |
| MCC21 | UNC45B | p.E148K | illdEnsea | illdKnsea | A0201 | 30 |
| MCC21 | UNC80 | p.R360Q | ylqrlRhml | ylqrlQhml | A0201 | 19 |
| MCC21 | ZNF14 | p.S517F | ktfsfssSl | ktfsfssFl | A0201 | 31 |
| MCC21 | ZNF354C | p.S56L | Smpklihql | Lmpklihql | A0201 | 42 |
| MCC21 | ZNF354C | p.S56L | fSmpklihq | fLmpklihq | A0201 | 94 |
| MCC21 | ZNF354C | p.S56L | lvslgipfS | lvslgipfL | A0201 | 200 |
| MCC23 | C4orf17 | p.V291I | eVpkeaehk | eIpkeaehk | A6801 | 388 |
| MCC23 | DLG5 | p.R1120W | fRpklapvv | fWpklapvv | C1402 | 72 |
| MCC23 | DLG5 | p.R1120W | ksapsfRpk | ksapsfWpk | A0301 | 52 |
| MCC23 | DLG5 | p.R1120W | ksapsfRpk | ksapsfWpk | A6801 | 341 |
| MCC23 | DLG5 | p.R1120W | sfRpklapv | sfWpklapv | C1402 | 16 |
| MCC23 | DNAH5 | p.V3030I | fleymnnVl | fleymnnIl | C0802 | 55 |
| MCC23 | DNAH5 | p.V3030I | sfleymnnV | sfleymnnI | C1402 | 24 |
| MCC23 | DNAH5 | p.V3030I | fleymnnVl | fleymnnIl | C1402 | 149 |
| MCC23 | DNAH5 | p.V3030I | eymnnVlss | eymnnIlss | C1402 | 464 |
| MCC23 | NLRP14 | p.C372Y | vCwaactcl | vYwaactcl | C1402 | 4 |
| MCC23 | NLRP14 | p.C372Y | Cwaactclk | Ywaactclk | A6801 | 392 |
| MCC23 | NR1I3 | p.S54L | ilSaealal | ilLaealal | C1402 | 76 |
| MCC23 | NR1I3 | p.S54L | lSaealalr | lLaealalr | A6801 | 58 |
| MCC23 | OR10A4 | p.T259M | fystailTy | fystailMy | C1402 | 17 |
| MCC23 | OR10A4 | p.T259M | ystailTyf | ystailMyf | C1402 | 196 |
| MCC23 | OR10A4 | p.T259M | lfystailT | lfystailM | C1402 | 8 |
| MCC23 | OR10A4 | p.T259M | stailTyfr | stailMyfr | A6801 | 5 |
| MCC23 | OR10A4 | p.T259M | Tyfrpqssa | Myfrpqssa | C1402 | 59 |
| MCC23 | OR6F1 | p.R293H | ytlRnkevr | ytlHnkevr | A6801 | 46 |
| MCC23 | OR6F1 | p.R293H | iytlRnkev | iytlHnkev | C1402 | 12 |
| MCC23 | ROS1 | p.G1135V | ftskGpgpy | ftskVpgpy | A6801 | 489 |
| MCC23 | ZC3H7B | p.K694E | Kfvcgqcwr | Efvcgqcwr | A6801 | 197 |
| MCC23 | ZC3H7B | p.K694E | stfdlqmKf | stfdlqmEf | C1402 | 151 |
| MCC3 | 1-Mar | p.V146L | ifcsvtfhV | ifcsvtfhL | A2402 | 428 |
| MCC3 | 1-Mar | p.V146L | fhViaitcv | fhLiaitcv | C0602 | 408 |
| MCC3 | 15-Sep | p.V13L | clVpafglr | clLpafglr | A3301 | 182 |
| MCC3 | A4GNT | p.G41E | shqGleall | shqEleall | C0602 | 261 |
| MCC3 | A4GNT | p.G41E | Gleallshr | Eleallshr | A3301 | 49 |
| MCC3 | ABCA13 | p.D1637N | irDvfnslm | irNvfnslm | C0602 | 56 |
| MCC3 | ABCA13 | p.E3205K | hvfEylpef | hvfKylpef | C0702 | 377 |
| MCC3 | ACACA | p.S277F | vmrlakqSr | vmrlakqFr | A3301 | 60 |
| MCC3 | ACACA | p.S277F | Srhlevqil | Frhlevqil | C0702 | 33 |
| MCC3 | ACACA | p.S277F | Srhlevqil | Frhlevqil | C0602 | 13 |
| MCC3 | ACTR10 | p.G223R | fvsdlkrGl | fvsdlkrRl | C0702 | 385 |
| MCC3 | ACTR10 | p.G223R | fvsdlkrGl | fvsdlkrRl | C0602 | 307 |
| MCC3 | ACTR5 | p.D546N | Ddnevwitr | Ndnevwitr | A3301 | 170 |
| MCC3 | ADAM11 | p.R333K | dathlfsgR | dathlfsgK | A3301 | 396 |
| MCC3 | ADCY2 | p.E499K | ylEswgaak | ylKswgaak | A3301 | 337 |
| MCC3 | ADCY2 | p.E499K | svrmtrylE | svrmtrylK | A3301 | 391 |
| MCC3 | ADH6 | p.G216R | vvmgckaaG | vvmgckaaR | A3301 | 107 |
| MCC3 | ADSSL1 | p.S139F | Sfigngvvi | Ffigngvvi | C0702 | 447 |
| MCC3 | AGL | p.P36S | nypfPgetf | nypfSgetf | A2402 | 36 |
| MCC3 | AGL | p.R1080C | sgifRcwgr | sgifCcwgr | A3301 | 442 |
| MCC3 | AGO2 | p.F716L | qkrhhtrlF | qkrhhtrlL | C0602 | 155 |
| MCC3 | AK5 | p.H102Y | drlppiHqf | drlppiYqf | C0702 | 110 |
| MCC3 | AKNAD1 | p.I330F | Iqmepivhi | Fqmepivhi | C0602 | 224 |
| MCC3 | ALS2CR11 | p.E738K | dlnnwlsEk | dlnnwlsKk | A3301 | 373 |
| MCC3 | AMER3 | p.K157R | lkrpKkcfr | lkrpRkcfr | A3301 | 389 |
| MCC3 | ANK3 | p.P3257S | iefppPppl | iefppSppl | C0702 | 373 |
| MCC3 | APBB1IP | p.F49L | efnysvgFk | efnysvgLk | A3301 | 202 |
| MCC3 | ARHGAP28 | p.P308L | frelPtslf | frelLtslf | C0602 | 222 |
| MCC3 | ARHGAP28 | p.P308L | frelPtslf | frelLtslf | C0702 | 147 |
| MCC3 | ARHGAP28 | p.P308L | ffrelPtsl | ffrelLtsl | C0702 | 79 |
| MCC3 | ARHGEF16 | p.S208F | Sfkddpqly | Ffkddpqly | C0602 | 455 |
| MCC3 | ARHGEF16 | p.S208F | Sfkddpqly | Ffkddpqly | C0702 | 57 |
| MCC3 | ATP1A1 | p.R233G | vytgdRtvm | vytgdGtvm | C0702 | 268 |
| MCC3 | ATP6V1B1 | p.V39I | ithprVtyr | ithprItyr | A3301 | 16 |
| MCC3 | ATP6V1B2 | p.H73Y | pryaeivHl | pryaeivYl | C0602 | 82 |
| MCC3 | ATP6V1B2 | p.H73Y | ryaeivHlt | ryaeivYlt | A2402 | 241 |
| MCC3 | B4GALT1 | p.R185C | aiiipfRnr | aiiipfCnr | A3301 | 279 |
| MCC3 | BRAF | p.F707Y | erplFpqil | erplYpqil | C0602 | 246 |
| MCC3 | BRAF | p.F707Y | lFpqilasi | lYpqilasi | A2402 | 13 |
| MCC3 | BRCA2 | p.S2072F | Slhkvkgvl | Flhkvkgvl | C0702 | 400 |
| MCC3 | BTBD18 | p.E119K | arqlrvsEl | arqlrvsKl | C0702 | 221 |
| MCC3 | BTBD18 | p.E119K | lrvsElesl | lrvsKlesl | C0602 | 213 |
| MCC3 | BTBD18 | p.E119K | arqlrvsEl | arqlrvsKl | C0602 | 253 |
| MCC3 | C12orf4 | p.R360Q | fhfpRieeq | fhfpQieeq | C0602 | 49 |
| MCC3 | C12orf60 | p.L46F | srsmntqiL | srsmntqiF | C0702 | 199 |
| MCC3 | C12orf60 | p.L46F | srsmntqiL | srsmntqiF | C0602 | 56 |
| MCC3 | C1orf111 | p.P246S | fnsPaehlr | fnsSaehlr | A3301 | 414 |
| MCC3 | C1orf111 | p.P246S | sPaehlrqi | sSaehlrqi | C0602 | 154 |
| MCC3 | C20orf26 | p.R496H | dldRynkar | dldHynkar | A3301 | 455 |
| MCC3 | C3 | p.G1475R | fnveliqpG | fnveliqpR | A3301 | 212 |
| MCC3 | C3orf30 | p.D495Y | yeDpyqvsl | yeYpyqvsl | C0702 | 55 |
| MCC3 | C3orf30 | p.D495Y | ivyeDpyqv | ivyeYpyqv | C0602 | 265 |
| MCC3 | C8orf34 | p.G378R | lrmeGvttl | lrmeRvttl | C0602 | 6 |
| MCC3 | C8orf34 | p.G378R | lrmeGvttl | lrmeRvttl | C0702 | 50 |
| MCC3 | C8orf34 | p.G378R | dlndlrmeG | dlndlrmeR | A3301 | 11 |
| MCC3 | CACNB2 | p.S32F | srpsdsdvS | srpsdsdvF | C0702 | 156 |
| MCC3 | CACNG3 | p.S282F | itmgtllnS | itmgtllnF | A2402 | 376 |
| MCC3 | CADPS2 | p.R504T | Rwkkryfvl | Twkkryfvl | C0702 | 334 |
| MCC3 | CADPS2 | p.R504T | Rwkkryfvl | Twkkryfvl | A2402 | 51 |
| MCC3 | CAPN14 | p.G260R | ytltGirkv | ytltRirkv | C0602 | 236 |
| MCC3 | CAPN14 | p.G260R | haytltGir | haytltRir | A3301 | 154 |
| MCC3 | CCDC168 | p.S193F | etiqfSskk | etiqfFskk | A3301 | 208 |
| MCC3 | CCDC168 | p.S193F | fSskklfsm | fFskklfsm | C0702 | 320 |
| MCC3 | CCDC30 | p.S440F | mtcsqqqSr | mtcsqqqFr | A3301 | 26 |
| MCC3 | CCDC30 | p.S440F | Sriqqqeal | Friqqqeal | C0602 | 23 |
| MCC3 | CCDC30 | p.S440F | Sriqqqeal | Friqqqeal | C0702 | 68 |
| MCC3 | CCDC36 | p.R209K | emkkRfear | emkkKfear | A3301 | 7 |
| MCC3 | CCDC47 | p.C209G | iynlwCsgr | iynlwGsgr | A3301 | 28 |
| MCC3 | CCND2 | p.P156S | tPhdfiehi | tShdfiehi | C0602 | 425 |
| MCC3 | CCP110 | p.R893C | evRkekmlr | evCkekmlr | A3301 | 25 |
| MCC3 | CCR6 | p.S54F | lfvpiaySl | lfvpiayFl | C0702 | 368 |
| MCC3 | CCR6 | p.S54F | fvpiaySli | fvpiayFli | A2402 | 96 |
| MCC3 | CCR6 | p.S54F | ySlicvfgl | yFlicvfgl | C0702 | 80 |
| MCC3 | CD1D | p.P111S | lrlsyPlel | lrlsySlel | C0602 | 24 |
| MCC3 | CD1D | p.P111S | lrlsyPlel | lrlsySlel | C0702 | 195 |
| MCC3 | CDC20B | p.S438F | vwtcptvSr | vwtcptvFr | A3301 | 105 |
| MCC3 | CDH6 | p.E119K | drEekpvyi | drKekpvyi | C0602 | 73 |
| MCC3 | CFTR | p.G314E | ffsGffvvf | ffsEffvvf | C0702 | 64 |
| MCC3 | CFTR | p.G314E | Gffvvflsv | Effvvflsv | A3301 | 292 |
| MCC3 | CFTR | p.G314E | fffsGffvv | fffsEffvv | C0702 | 120 |
| MCC3 | CFTR | p.G314E | ffsGffvvf | ffsEffvvf | A2402 | 172 |
| MCC3 | CHEK2 | p.G102R | dGfanlecv | dRfanlecv | C0602 | 166 |
| MCC3 | CLEC4D | p.G179R | qGencvvlv | qRencvvlv | C0602 | 20 |
| MCC3 | CLEC4E | p.R203W | nyfRicemv | nyfWicemv | A2402 | 367 |
| MCC3 | CLGN | p.E398K | yfEddhpfl | yfKddhpfl | C0702 | 55 |
| MCC3 | CLGN | p.E398K | dyfEddhpf | dyfKddhpf | C0702 | 246 |
| MCC3 | CLGN | p.E398K | fEddhpfll | fKddhpfll | C0602 | 96 |
| MCC3 | CNR2 | p.S203F | lfSgiiyty | lfFgiiyty | C0702 | 450 |
| MCC3 | COL14A1 | p.P14S | rywllPpfl | rywllSpfl | A2402 | 124 |
| MCC3 | COL14A1 | p.P14S | mrywllPpf | mrywllSpf | C0702 | 206 |
| MCC3 | COL14A1 | p.P14S | rywllPpfl | rywllSpfl | C0702 | 188 |
| MCC3 | COL6A5 | p.S199F | fSqnmtqii | fFqnmtqii | A2402 | 213 |
| MCC3 | COL6A5 | p.S199F | fSqnmtqii | fFqnmtqii | C0602 | 338 |
| MCC3 | COL6A5 | p.S199F | fSqnmtqii | fFqnmtqii | C0702 | 409 |
| MCC3 | CPXM2 | p.P263L | elPvpmvar | elLvpmvar | A3301 | 11 |
| MCC3 | CRYGC | p.R37W | yfsrcnsiR | yfsrcnsiW | A2402 | 295 |
| MCC3 | CSMD2 | p.E1760K | vrfEcnsgy | vrfKcnsgy | C0602 | 101 |
| MCC3 | CTSG | p.R122Q | rrnRnvnpv | rrnQnvnpv | C0602 | 111 |
| MCC3 | CTSG | p.R122Q | rrvrrnRnv | rrvrrnQnv | C0602 | 185 |
| MCC3 | CUBN | p.H2810Y | ifHsdngti | ifYsdngti | A2402 | 475 |
| MCC3 | CUL7 | p.S329F | sarspgSif | sarspgFif | C0702 | 391 |
| MCC3 | DHTKD1 | p.H622Y | Hmdpnqkgf | Ymdpnqkgf | C0702 | 385 |
| MCC3 | DNAH6 | p.H1874Y | ltpqiHmlf | ltpqiYmlf | A2402 | 20 |
| MCC3 | DNAH6 | p.H1874Y | kltpqiHml | kltpqiYml | C0702 | 307 |
| MCC3 | DNTTIP1 | p.I78M | sIneeiqtv | sMneeiqtv | C0602 | 190 |
| MCC3 | DOCK3 | p.S1188F | iSfvtsvtr | iFfvtsvtr | A3301 | 20 |
| MCC3 | DOCK3 | p.S1188F | Sfvtsvtrl | Ffvtsvtrl | C0702 | 207 |
| MCC3 | DOK2 | p.G168R | hlrGsytlr | hlrRsytlr | A3301 | 29 |
| MCC3 | DOK2 | p.G168R | chlrGsytl | chlrRsytl | A2402 | 429 |
| MCC3 | DOK2 | p.G168R | chlrGsytl | chlrRsytl | C0602 | 306 |
| MCC3 | DYSF | p.Y578F | ylrrrkYsl | ylrrrkFsl | C0702 | 175 |
| MCC3 | EAF2 | p.G116E | eGsskiqyr | eEsskiqyr | A3301 | 313 |
| MCC3 | EAF2 | p.G116E | trveGsski | trveEsski | C0602 | 173 |
| MCC3 | EDN1 | p.R161K | lvRgrkirr | lvKgrkirr | A3301 | 156 |
| MCC3 | EEF1G | p.F384Y | vfrgqelaF | vfrgqelaY | C0702 | 418 |
| MCC3 | ELL | p.S126F | Syqkarqsm | Fyqkarqsm | C0602 | 223 |
| MCC3 | ELL | p.S126F | Syqkarqsm | Fyqkarqsm | C0702 | 163 |
| MCC3 | EMILIN2 | p.S613F | dfsflySql | dfsflyFql | A2402 | 398 |
| MCC3 | EPHA5 | p.G853R | yGerpywem | yRerpywem | C0702 | 150 |
| MCC3 | EPHA5 | p.G853R | yGerpywem | yRerpywem | C0602 | 318 |
| MCC3 | EPHA5 | p.G853R | vmwevvsyG | vmwevvsyR | A3301 | 9 |
| MCC3 | EPHB1 | p.A297P | eAspictcr | ePspictcr | A3301 | 87 |
| MCC3 | EPHX4 | p.A334V | frltilseA | frltilseV | C0602 | 31 |
| MCC3 | ETNPPL | p.V197A | rVgkhfwsf | rAgkhfwsf | A2402 | 86 |
| MCC3 | F13A1 | p.K55E | tsvhlfKer | tsvhlfEer | A3301 | 335 |
| MCC3 | FAAH | p.S211F | tvnpwksSk | tvnpwksFk | A3301 | 259 |
| MCC3 | FAM179A | p.L177F | lrvprpmpL | lrvprpmpF | C0702 | 42 |
| MCC3 | FAM196B | p.S34F | hrrskSqqv | hrrskFqqv | C0602 | 81 |
| MCC3 | FAM196B | p.S34F | hrrskSqqv | hrrskFqqv | C0702 | 425 |
| MCC3 | FAM200A | p.L389F | Lksnrpsyy | Fksnrpsyy | C0602 | 139 |
| MCC3 | FAM200A | p.L389F | tlllwqarL | tlllwqarF | A2402 | 313 |
| MCC3 | FAM83C | p.P326L | alrppPval | alrppLval | C0702 | 145 |
| MCC3 | FAT4 | p.S2683F | eilenlSpr | eilenlFpr | A3301 | 5 |
| MCC3 | FCRL1 | p.D57N | cffrDtral | cffrNtral | C0702 | 231 |
| MCC3 | FCRL1 | p.D57N | qfcffrDtr | qfcffrNtr | A3301 | 12 |
| MCC3 | FMN2 | p.S614L | dySegqfpr | dyLegqfpr | A3301 | 3 |
| MCC3 | FOXJ3 | p.L258H | nfedLsasf | nfedHsasf | C0702 | 256 |
| MCC3 | GCC2 | p.E254K | ihqeevkEl | ihqeevkKl | C0602 | 166 |
| MCC3 | GIF | p.A51V | sAypnpsil | sVypnpsil | C0702 | 89 |
| MCC3 | GIF | p.A51V | Aypnpsili | Vypnpsili | A2402 | 33 |
| MCC3 | GIF | p.A51V | sAypnpsil | sVypnpsil | C0602 | 150 |
| MCC3 | GIMAP1 | p.V224G | hysneVyel | hysneGyel | C0702 | 75 |
| MCC3 | GNB1 | p.S165F | Shdniicgi | Fhdniicgi | C0602 | 320 |
| MCC3 | GP6 | p.S494F | prnshphSi | prnshphFi | C0602 | 19 |
| MCC3 | GPN3 | p.S49F | nySvmadir | nyFvmadir | A3301 | 164 |
| MCC3 | GPN3 | p.S49F | Svmadirel | Fvmadirel | C0602 | 86 |
| MCC3 | GPN3 | p.S49F | Svmadirel | Fvmadirel | C0702 | 248 |
| MCC3 | GPR113 | p.G710E | lylpqGqyl | lylpqEqyl | C0702 | 148 |
| MCC3 | GPR113 | p.G710E | lylpqGqyl | lylpqEqyl | C0602 | 472 |
| MCC3 | GPR162 | p.L114F | hmlfppLer | hmlfppFer | A3301 | 9 |
| MCC3 | GPR45 | p.N320S | vfNpivycw | vfSpivycw | A2402 | 58 |
| MCC3 | GPR45 | p.N320S | vfNpivycw | vfSpivycw | C0702 | 413 |
| MCC3 | GPR98 | p.R3023K | Ryknvnimi | Kyknvnimi | A2402 | 210 |
| MCC3 | GRB10 | p.L127V | eiLadmtar | eiVadmtar | A3301 | 140 |
| MCC3 | HS6ST3 | p.D288N | Ddwsgvslr | Ndwsgvslr | A3301 | 240 |
| MCC3 | HSPA5 | p.L361F | ivLvggstr | ivFvggstr | A3301 | 134 |
| MCC3 | HTT | p.S783F | tlicsilSr | tlicsilFr | A3301 | 107 |
| MCC3 | HYDIN | p.H1726Y | nHlqvpcew | nYlqvpcew | A2402 | 169 |
| MCC3 | IGSF6 | p.S5R | Srsniarhl | Rrsniarhl | C0602 | 37 |
| MCC3 | IGSF6 | p.S5R | taSrsniar | taRrsniar | A3301 | 84 |
| MCC3 | IL36G | p.S107F | afpdwfiaS | afpdwfiaF | A2402 | 124 |
| MCC3 | IL36G | p.S107F | dwfiaSskr | dwfiaFskr | A3301 | 18 |
| MCC3 | IL36G | p.S107F | afpdwfiaS | afpdwfiaF | C0702 | 65 |
| MCC3 | KBTBD12 | p.M113I | amaayfmqM | amaayfmqI | A2402 | 284 |
| MCC3 | KBTBD12 | p.M113I | yfmqMeevf | yfmqIeevf | C0702 | 62 |
| MCC3 | KBTBD12 | p.M113I | yfmqMeevf | yfmqIeevf | A2402 | 99 |
| MCC3 | KCNJ14 | p.A295T | arAdfelvv | arTdfelvv | C0602 | 148 |
| MCC3 | KCNJ3 | p.P329S | hrffPvisl | hrffSvisl | C0602 | 146 |
| MCC3 | KCNJ3 | p.P329S | hrffPvisl | hrffSvisl | C0702 | 92 |
| MCC3 | KDM4E | p.S317F | fSmdpfvri | fFmdpfvri | C0702 | 86 |
| MCC3 | KDM4E | p.S317F | tfSmdpfvr | tfFmdpfvr | A3301 | 11 |
| MCC3 | KDM4E | p.S317F | Smdpfvriv | Fmdpfvriv | C0602 | 203 |
| MCC3 | KDM4E | p.S317F | fSmdpfvri | fFmdpfvri | A2402 | 174 |
| MCC3 | KIF20B | p.S116F | maqkfsfSk | maqkfsfFk | A3301 | 127 |
| MCC3 | KIF20B | p.S116F | qmaqkfsfS | qmaqkfsfF | A2402 | 190 |
| MCC3 | KRTAP1-3 | p.P121L | crvegtclP | crvegtclL | C0602 | 305 |
| MCC3 | LAMA4 | p.G1765R | dhrepvfvG | dhrepvfvR | A3301 | 46 |
| MCC3 | LBP | p.F78L | ryeFhslni | ryeLhslni | A2402 | 112 |
| MCC3 | LEMD2 | p.P135S | eryPyvgil | erySyvgil | C0702 | 58 |
| MCC3 | LEMD2 | p.P135S | Pyvgilhvr | Syvgilhvr | A3301 | 92 |
| MCC3 | LEMD2 | p.P135S | eryPyvgil | erySyvgil | C0602 | 23 |
| MCC3 | LEPR | p.S492R | epkdcylqS | epkdcylqR | A3301 | 79 |
| MCC3 | LGALS8 | p.P18S | iiynpviPf | iiynpviSf | C0702 | 451 |
| MCC3 | LGALS8 | p.P18S | iynpviPfv | iynpviSfv | A2402 | 231 |
| MCC3 | LHX6 | p.D159N | Dmdgplsnr | Nmdgplsnr | A3301 | 129 |
| MCC3 | LIN7A | p.G141R | yisriipgG | yisriipgR | A3301 | 218 |
| MCC3 | LOXHD1 | p.E1455K | vykeveEpl | vykeveKpl | C0702 | 119 |
| MCC3 | LPO | p.P348S | frdylPill | frdylSill | C0602 | 60 |
| MCC3 | LPO | p.P348S | tfrdylPil | tfrdylSil | C0702 | 44 |
| MCC3 | LRGUK | p.R528C | syfepRyil | syfepCyil | C0702 | 23 |
| MCC3 | LRGUK | p.R528C | yfepRyilv | yfepCyilv | C0702 | 91 |
| MCC3 | LRGUK | p.R528C | syfepRyil | syfepCyil | A2402 | 144 |
| MCC3 | LRP2 | p.D1091H | wrcdkrnDc | wrcdkrnHc | C0602 | 454 |
| MCC3 | MACF1 | p.P3877S | ehkPhidkl | ehkShidkl | C0602 | 269 |
| MCC3 | MACF1 | p.P3877S | Phidkllki | Shidkllki | C0602 | 88 |
| MCC3 | MACF1 | p.L4850F | Lidthkefm | Fidthkefm | C0602 | 427 |
| MCC3 | MACF1 | p.L4850F | sLidthkef | sFidthkef | C0702 | 394 |
| MCC3 | MDGA2 | p.R341Q | lraypiRvl | lraypiQvl | C0602 | 163 |
| MCC3 | MDN1 | p.P1708L | gihPffipr | gihLffipr | A3301 | 227 |
| MCC3 | MDN1 | p.P1708L | lwgihPffi | lwgihLffi | A2402 | 184 |
| MCC3 | MEP1B | p.R642T | ymgeRcekr | ymgeTcekr | A3301 | 254 |
| MCC3 | METAP1 | p.Y291D | vrsYcghgi | vrsDcghgi | C0602 | 24 |
| MCC3 | MMP10 | p.S317F | Shwnpepef | Fhwnpepef | C0702 | 128 |
| MCC3 | MMP10 | p.S317F | ryfwrrShw | ryfwrrFhw | A2402 | 40 |
| MCC3 | MMP10 | p.S317F | Shwnpepef | Fhwnpepef | C0602 | 150 |
| MCC3 | MS4A1 | p.S144F | Shflkmesl | Fhflkmesl | C0702 | 266 |
| MCC3 | MS4A1 | p.S144F | nikiShflk | nikiFhflk | A3301 | 93 |
| MCC3 | MS4A1 | p.S144F | Shflkmesl | Fhflkmesl | C0602 | 26 |
| MCC3 | MSH6 | p.D260H | rrrpdhpDf | rrrpdhpHf | C0702 | 43 |
| MCC3 | MSH6 | p.D260H | pDfdastly | pHfdastly | C0602 | 169 |
| MCC3 | MTAP | p.V170I | tmVtiegpr | tmItiegpr | A3301 | 45 |
| MCC3 | MYCBP2 | p.G526R | tGfdeesai | tRfdeesai | C0702 | 458 |
| MCC3 | MYCBP2 | p.G526R | tGfdeesai | tRfdeesai | C0602 | 50 |
| MCC3 | MYLK3 | p.E742K | dtfeglseE | dtfeglseK | A3301 | 78 |
| MCC3 | MYO10 | p.Q1594L | ilQtghdlr | ilLtghdlr | A3301 | 222 |
| MCC3 | MYO3A | p.R991W | hRilfanfi | hWilfanfi | A2402 | 230 |
| MCC3 | MYO5B | p.E1475K | yhkEdeall | yhkKdeall | C0602 | 181 |
| MCC3 | NAA50 | p.P28L | krlnqvifP | krlnqvifL | C0702 | 342 |
| MCC3 | NAA50 | p.P28L | krlnqvifP | krlnqvifL | C0602 | 142 |
| MCC3 | NCAPD3 | p.S511F | nssafSyqr | nssafFyqr | A3301 | 30 |
| MCC3 | NCAPD3 | p.S511F | Syqrqtsnr | Fyqrqtsnr | A3301 | 118 |
| MCC3 | NCR1 | p.R146W | lRmglaflv | lWmglaflv | A2402 | 161 |
| MCC3 | NEB | p.G357R | kGkadynvl | kRkadynvl | C0702 | 391 |
| MCC3 | NFASC | p.V1077A | tytlrVysr | tytlrAysr | A3301 | 11 |
| MCC3 | NFXL1 | p.H427Y | Hrcsqrchr | Yrcsqrchr | A3301 | 301 |
| MCC3 | NRD1 | p.I477S | rIfeeirki | rSfeeirki | C0602 | 270 |
| MCC3 | OCLM | p.I40L | wysfIllvl | wysfLllvl | A2402 | 372 |
| MCC3 | OCLM | p.I40L | iwlswysfI | iwlswysfL | C0702 | 282 |
| MCC3 | OCLM | p.I40L | iwlswysfI | iwlswysfL | A2402 | 17 |
| MCC3 | OR10A4 | p.P59L | qsPmyfflr | qsLmyfflr | A3301 | 6 |
| MCC3 | OR10A4 | p.P59L | lqsPmyffl | lqsLmyffl | A2402 | 396 |
| MCC3 | OR2M7 | p.S165F | fSfsycgsr | fFfsycgsr | A3301 | 7 |
| MCC3 | OR4C3 | p.A129V | Ahflggvei | Vhflggvei | C0602 | 277 |
| MCC3 | OR4C6 | p.M134I | hytiiMspr | hytiiIspr | A3301 | 8 |
| MCC3 | OR4C6 | p.M134I | iMsprvccl | iIsprvccl | C0702 | 399 |
| MCC3 | OR4P4 | p.R308K | illkRnqlf | illkKnqlf | A2402 | 348 |
| MCC3 | OR51B2 | p.P282S | iyflfPplm | iyflfSplm | A2402 | 151 |
| MCC3 | OR51B2 | p.P282S | iyflfPplm | iyflfSplm | C0702 | 343 |
| MCC3 | OR51B2 | p.P282S | yiyflfPpl | yiyflfSpl | C0702 | 447 |
| MCC3 | OR51B4 | p.G17S | lGseavhyr | lSseavhyr | A3301 | 191 |
| MCC3 | OR51Q1 | p.M186I | lhqdMirlv | lhqdIirlv | C0602 | 29 |
| MCC3 | OR5AN1 | p.P288S | lnPliyslr | lnSliyslr | A3301 | 170 |
| MCC3 | OR5W2 | p.N286S | mlNpliysl | mlSpliysl | C0702 | 450 |
| MCC3 | OR5W2 | p.N286S | lNpliyslr | lSpliyslr | A3301 | 350 |
| MCC3 | OR8H1 | p.G16R | tGlsdseev | tRlsdseev | C0602 | 46 |
| MCC3 | OR8H1 | p.G16R | nvpdfiltG | nvpdfiltR | A3301 | 480 |
| MCC3 | PAMR1 | p.S211C | yvlSgnekr | yvlCgnekr | A3301 | 312 |
| MCC3 | PAPLN | p.R1137W | esvniRwsr | esvniWwsr | A3301 | 4 |
| MCC3 | PARD6G | p.R30Q | efRrfsldr | efQrfsldr | A3301 | 71 |
| MCC3 | PARD6G | p.R30Q | kfgaefRrf | kfgaefQrf | A2402 | 60 |
| MCC3 | PCDH15 | p.D104N | nstgrvlDr | nstgrvlNr | A3301 | 118 |
| MCC3 | PCLO | p.Q3965K | qqkprQttl | qqkprKttl | C0702 | 458 |
| MCC3 | PCNX | p.R782C | fRrerstfr | fCrerstfr | A3301 | 26 |
| MCC3 | PCNX | p.R782C | eavsfRrer | eavsfCrer | A3301 | 33 |
| MCC3 | PCNX | p.R782C | sfRrerstf | sfCrerstf | C0702 | 364 |
| MCC3 | PDE1A | p.E291K | lrnlviEmv | lrnlviKmv | C0602 | 417 |
| MCC3 | PDE6A | p.E312K | prtpdgrEi | prtpdgrKi | C0602 | 360 |
| MCC3 | PDK2 | p.L116V | daLvtirnr | daVvtirnr | A3301 | 43 |
| MCC3 | PEX6 | p.G871W | rfdklvfvG | rfdklvfvW | A2402 | 338 |
| MCC3 | PGLYRP4 | p.E286K | iyEgvgwnv | iyKgvgwnv | A2402 | 238 |
| MCC3 | PGM2L1 | p.S490F | yhisktSyf | yhisktFyf | A2402 | 61 |
| MCC3 | PGM2L1 | p.S490F | yhisktSyf | yhisktFyf | C0602 | 55 |
| MCC3 | PGM2L1 | p.S490F | yhisktSyf | yhisktFyf | C0702 | 275 |
| MCC3 | PKD1L1 | p.L1957F | srylhtprL | srylhtprF | C0702 | 71 |
| MCC3 | PKD1L1 | p.L1957F | rLtvsfsll | rFtvsfsll | A2402 | 433 |
| MCC3 | PKD1L1 | p.L1957F | srylhtprL | srylhtprF | C0602 | 64 |
| MCC3 | PLA2G4C | p.T17I | lrrtenflT | lrrtenflI | C0602 | 344 |
| MCC3 | PLAC8 | p.R77Q | mrtlyrtRy | mrtlyrtQy | C0602 | 176 |
| MCC3 | PLEKHG4 | p.S861F | vrtgrhkSv | vrtgrhkFv | C0602 | 55 |
| MCC3 | PLEKHG4 | p.S861F | grhkSvrri | grhkFvrri | C0602 | 96 |
| MCC3 | PLEKHG4 | p.S861F | kSvrriflf | kFvrriflf | C0702 | 189 |
| MCC3 | PLEKHG4 | p.S861F | kSvrriflf | kFvrriflf | A2402 | 21 |
| MCC3 | PM20D2 | p.K321R | nKslwkaym | nRslwkaym | C0602 | 207 |
| MCC3 | PM20D2 | p.K321R | dyynvlpnK | dyynvlpnR | A3301 | 5 |
| MCC3 | POLN | p.D720N | ykkikDfar | ykkikNfar | A3301 | 287 |
| MCC3 | PRMT10 | p.P132L | fhkavklnP | fhkavklnL | C0602 | 251 |
| MCC3 | PRR21 | p.S327F | Stslhprpf | Ftslhprpf | C0702 | 364 |
| MCC3 | PRR21 | p.S327F | ssStslhpr | ssFtslhpr | A3301 | 73 |
| MCC3 | PRSS12 | p.G409E | yrgqwGtvc | yrgqwEtvc | C0602 | 232 |
| MCC3 | PRSS12 | p.G409E | yyrgqwGtv | yyrgqwEtv | A2402 | 96 |
| MCC3 | PRSS12 | p.G409E | yyrgqwGtv | yyrgqwEtv | C0702 | 166 |
| MCC3 | PRTG | p.L773F | yLqtsethm | yFqtsethm | C0702 | 431 |
| MCC3 | PRTG | p.L773F | Lqtsethml | Fqtsethml | C0602 | 337 |
| MCC3 | PTGS1 | p.S383F | eigapfSlk | eigapfFlk | A3301 | 134 |
| MCC3 | PTHLH | p.R144H | krrtRsawl | krrtHsawl | C0602 | 294 |
| MCC3 | PTPN13 | p.F469Y | yetpFegnl | yetpYegnl | C0702 | 360 |
| MCC3 | PTPRC | p.S297L | pytkyvlSl | pytkyvlLl | A2402 | 236 |
| MCC3 | PTPRF | p.P1304S | ahssdPvem | ahssdSvem | C0602 | 150 |
| MCC3 | RALYL | p.R132K | yyRddfynr | yyKddfynr | A3301 | 5 |
| MCC3 | RALYL | p.R132K | yvfdydyyR | yvfdydyyK | A3301 | 48 |
| MCC3 | RASA2 | p.P530L | tfhlrphhP | tfhlrphhL | C0702 | 202 |
| MCC3 | RASAL1 | p.L479F | Ltpklfdlr | Ftpklfdlr | A3301 | 397 |
| MCC3 | RASAL1 | p.L479F | iLtpklfdl | iFtpklfdl | C0702 | 154 |
| MCC3 | RASGEF1C | p.D434N | ifseDglyl | ifseNglyl | C0602 | 285 |
| MCC3 | RASGRP2 | p.E432K | ekmvEsvfr | ekmvKsvfr | A3301 | 51 |
| MCC3 | RB1CC1 | p.P1574S | vPlgtkfyr | vSlgtkfyr | A3301 | 45 |
| MCC3 | RBBP8NL | p.P560L | ePskaevlr | eLskaevlr | A3301 | 182 |
| MCC3 | RCOR3 | p.E338K | rrfnlEevl | rrfnlKevl | C0602 | 26 |
| MCC3 | RCOR3 | p.E338K | rrrfnlEev | rrrfnlKev | C0602 | 215 |
| MCC3 | RCOR3 | p.E338K | rrfnlEevl | rrfnlKevl | C0702 | 75 |
| MCC3 | RCOR3 | p.E338K | nyrrrfnlE | nyrrrfnlK | A3301 | 41 |
| MCC3 | RCVRN | p.T93I | eyvialhmT | eyvialhmI | A2402 | 86 |
| MCC3 | RELL1 | p.G71A | imGlfgvli | imAlfgvli | A2402 | 465 |
| MCC3 | RFX2 | p.P435S | eiliPdvlr | eiliSdvlr | A3301 | 57 |
| MCC3 | RGS9 | p.P473S | qhmaPsphl | qhmaSsphl | C0602 | 176 |
| MCC3 | RIMS1 | p.F1089L | ecFnstvlr | ecLnstvlr | A3301 | 204 |
| MCC3 | RP1 | p.E1225K | sEnertqgi | sKnertqgi | C0602 | 185 |
| MCC3 | RPGRIP1 | p.R981P | eagqyRskr | eagqyPskr | A3301 | 305 |
| MCC3 | RPL10L | p.C105F | mlsCagadr | mlsFagadr | A3301 | 477 |
| MCC3 | RPL10L | p.C105F | irinkmlsC | irinkmlsF | C0602 | 218 |
| MCC3 | RPL10L | p.C105F | irinkmlsC | irinkmlsF | C0702 | 285 |
| MCC3 | RPLP0 | p.D256E | Dytfplaek | Eytfplaek | A3301 | 78 |
| MCC3 | RUFY4 | p.M82I | nMepihfvr | nIepihfvr | A3301 | 66 |
| MCC3 | SCARF2 | p.S542F | qpspswSsr | qpspswFsr | A3301 | 478 |
| MCC3 | SCN11A | p.G752R | mGdfwhsfl | mRdfwhsfl | C0602 | 124 |
| MCC3 | SCN11A | p.G752R | hmGdfwhsf | hmRdfwhsf | A2402 | 478 |
| MCC3 | SCN11A | p.G752R | lrhwhmGdf | lrhwhmRdf | C0602 | 58 |
| MCC3 | SCN11A | p.G752R | hmGdfwhsf | hmRdfwhsf | C0702 | 89 |
| MCC3 | SCN11A | p.G752R | sclrhwhmG | sclrhwhmR | A3301 | 260 |
| MCC3 | SCN7A | p.D1437N | ifagwDgml | ifagwNgml | A2402 | 447 |
| MCC3 | SCN7A | p.P1184S | vflPlsmli | vflSlsmli | A2402 | 43 |
| MCC3 | SCN9A | p.G1651R | fGmsnfayv | fRmsnfayv | C0602 | 7 |
| MCC3 | SCN9A | p.G1651R | vmfiyaifG | vmfiyaifR | A3301 | 20 |
| MCC3 | SCN9A | p.G1651R | fGmsnfayv | fRmsnfayv | C0702 | 309 |
| MCC3 | SCN9A | p.S1557F | cvlkliSlr | cvlkliFlr | A3301 | 14 |
| MCC3 | SEC16B | p.P802S | fysvPethl | fysvSethl | C0702 | 414 |
| MCC3 | SESTD1 | p.Q335K | vyvelnQqi | vyvelnKqi | A2402 | 416 |
| MCC3 | SETD3 | p.R460C | Rakmaiklr | Cakmaiklr | A3301 | 135 |
| MCC3 | SGSM1 | p.P452S | awmmvPagr | awmmvSagr | A3301 | 165 |
| MCC3 | SLC12A6 | p.G142E | ifGvilflr | ifEvilflr | A3301 | 10 |
| MCC3 | SLC24A3 | p.G580R | rGliysvgl | rRliysvgl | C0702 | 388 |
| MCC3 | SLC24A3 | p.G580R | syirlnsrG | syirlnsrR | A3301 | 81 |
| MCC3 | SLC24A3 | p.G580R | irlnsrGli | irlnsrRli | C0602 | 22 |
| MCC3 | SLC24A3 | p.G580R | yirlnsrGl | yirlnsrRl | C0702 | 281 |
| MCC3 | SLC25A11 | p.R237C | gftpyyaRl | gftpyyaCl | C0702 | 94 |
| MCC3 | SLC2A13 | p.L588F | gfaavglLf | gfaavglFf | A2402 | 468 |
| MCC3 | SLC2A3 | p.P385S | Pwfivaelf | Swfivaelf | A2402 | 99 |
| MCC3 | SLC4A8 | p.L274F | vrlspavlL | vrlspavlF | C0702 | 67 |
| MCC3 | SLC5A6 | p.S22F | Stfsimdyv | Ftfsimdyv | C0602 | 367 |
| MCC3 | SMAP2 | p.E19K | eaylpEtfr | eaylpKtfr | A3301 | 4 |
| MCC3 | SMAP2 | p.E19K | aylpEtfrr | aylpKtfrr | A3301 | 238 |
| MCC3 | SP110 | p.G473E | eakGilykk | eakEilykk | A3301 | 102 |
| MCC3 | SPAM1 | p.P31L | Pccltlnfr | Lccltlnfr | A3301 | 364 |
| MCC3 | SPATA22 | p.E256K | ntlpcvfyE | ntlpcvfyK | A3301 | 59 |
| MCC3 | SPDYE4 | p.S237F | wardrtliS | wardrtliF | C0702 | 456 |
| MCC3 | SPEF2 | p.D411Y | krfhDqiav | krfhYqiav | C0702 | 481 |
| MCC3 | SPEF2 | p.D411Y | krfhDqiav | krfhYqiav | C0602 | 342 |
| MCC3 | SPIDR | p.Q297K | ifppwQkli | ifppwKkli | C0702 | 313 |
| MCC3 | SPIDR | p.Q297K | ifppwQkli | ifppwKkli | A2402 | 47 |
| MCC3 | SPIDR | p.Q297K | rifppwQkl | rifppwKkl | C0702 | 233 |
| MCC3 | SPRED2 | p.P83S | vytkanPtf | vytkanStf | C0702 | 432 |
| MCC3 | SPRED2 | p.P83S | vytkanPtf | vytkanStf | A2402 | 41 |
| MCC3 | SRGAP1 | p.S886F | Spslashpr | Fpslashpr | A3301 | 286 |
| MCC3 | STXBP5L | p.P70S | Phqptalaf | Shqptalaf | C0702 | 216 |
| MCC3 | STYXL1 | p.L62F | ylLpesvdl | ylFpesvdl | C0702 | 94 |
| MCC3 | SUMF2 | p.T128I | lrnkaTqpm | lrnkaIqpm | C0602 | 312 |
| MCC3 | SVOP | p.G167R | lyygilSaf | lyygilRaf | A2402 | 131 |
| MCC3 | SVOP | p.G167R | wtlyygilS | wtlyygilR | A3301 | 33 |
| MCC3 | SVOP | p.G167R | lyygilSaf | lyygilRaf | C0702 | 108 |
| MCC3 | SVOPL | p.P272S | vyPttmral | vySttmral | C0702 | 201 |
| MCC3 | SYNE1 | p.E1350K | etnkEtvvr | etnkKtvvr | A3301 | 37 |
| MCC3 | T | p.V175A | vrVggpqrm | vrAggpqrm | C0602 | 102 |
| MCC3 | TAF1L | p.S1040F | Srwevidvv | Frwevidvv | C0702 | 177 |
| MCC3 | TAF1L | p.S1040F | Srwevidvv | Frwevidvv | C0602 | 21 |
| MCC3 | TARBP1 | p.R475W | kfiRkmtsr | kfiWkmtsr | A3301 | 285 |
| MCC3 | TARS2 | p.S351F | rSvyavlgf | rFvyavlgf | A2402 | 354 |
| MCC3 | TAS2R20 | p.P272S | afgiiyPsf | afgiiySsf | A2402 | 126 |
| MCC3 | TAS2R20 | p.P272S | iiyPsfhsf | iiySsfhsf | A2402 | 166 |
| MCC3 | TAS2R20 | p.P272S | iyPsfhsfi | iySsfhsfi | A2402 | 13 |
| MCC3 | TBC1D12 | p.L571V | pyhdvLhsi | pyhdvVhsi | A2402 | 195 |
| MCC3 | TBX19 | p.P428L | wtavashPf | wtavashLf | A2402 | 217 |
| MCC3 | TDRD9 | p.H172P | yildHyvqr | yildPyvqr | A3301 | 56 |
| MCC3 | TENM2 | p.S39F | kSysssetl | kFysssetl | C0702 | 178 |
| MCC3 | TEP1 | p.G870E | Gktgvqslr | Ektgvqslr | A3301 | 439 |
| MCC3 | TEX36 | p.K178Q | vrftvdKkv | vrftvdQkv | C0602 | 133 |
| MCC3 | TLR4 | p.P339S | ffsldtfPy | ffsldtfSy | C0702 | 198 |
| MCC3 | TLR4 | p.P339S | fsldtfPyk | fsldtfSyk | A3301 | 330 |
| MCC3 | TLR4 | p.P339S | fPykclnsl | fSykclnsl | C0602 | 155 |
| MCC3 | TMC3 | p.G175R | fGstarkti | fRstarkti | C0602 | 19 |
| MCC3 | TMC3 | p.G175R | eliagqpfG | eliagqpfR | A3301 | 107 |
| MCC3 | TMEM17 | p.R172C | Rfhlqdfdr | Cfhlqdfdr | A3301 | 228 |
| MCC3 | TNFSF13B | p.L150F | sytfvpwLl | sytfvpwFl | A2402 | 15 |
| MCC3 | TNFSF13B | p.L150F | tfvpwLlsf | tfvpwFlsf | A2402 | 19 |
| MCC3 | TNFSF13B | p.L150F | vpwLlsfkr | vpwFlsfkr | A3301 | 155 |
| MCC3 | TNFSF13B | p.L150F | ytfvpwLls | ytfvpwFls | A3301 | 298 |
| MCC3 | TNFSF13B | p.L150F | tfvpwLlsf | tfvpwFlsf | C0702 | 64 |
| MCC3 | TRIP12 | p.G1396R | eGtkyiqnl | eRtkyiqnl | C0602 | 57 |
| MCC3 | TRPM3 | p.L1285F | psssayatL | psssayatF | A2402 | 282 |
| MCC3 | TTN | p.G2872S | mwlknGvei | mwlknSvei | A2402 | 174 |
| MCC3 | TTN | p.S2235L | Sysgeleci | Lysgeleci | A2402 | 126 |
| MCC3 | TTN | p.S265F | srSptppsi | srFptppsi | C0702 | 289 |
| MCC3 | TTN | p.S265F | srSptppsi | srFptppsi | C0602 | 31 |
| MCC3 | TUBG1 | p.T240I | msasTttlr | msasIttlr | A3301 | 34 |
| MCC3 | UBC | p.G123R | dqqrlifaG | dqqrlifaR | A3301 | 56 |
| MCC3 | UGCG | p.I31F | hfmaiIytr | hfmaiFytr | A3301 | 3 |
| MCC3 | UGT3A1 | p.S27L | syqvirwfS | syqvirwfL | A2402 | 45 |
| MCC3 | UGT3A1 | p.S27L | syqvirwfS | syqvirwfL | C0702 | 481 |
| MCC3 | UHRF1BP1L | p.S1021L | yredsnilS | yredsnilL | C0702 | 134 |
| MCC3 | UHRF1BP1L | p.S1021L | yredsnilS | yredsnilL | C0602 | 13 |
| MCC3 | UMODL1 | p.S1018F | tSsgftlew | tFsgftlew | A2402 | 458 |
| MCC3 | UMODL1 | p.S1018F | afqndlltS | afqndlltF | A2402 | 118 |
| MCC3 | UMODL1 | p.S1018F | afqndlltS | afqndlltF | C0702 | 179 |
| MCC3 | UQCRC1 | p.P283R | lPfahvaia | lRfahvaia | C0602 | 254 |
| MCC3 | UQCRC1 | p.P283R | rhrddalPf | rhrddalRf | C0602 | 296 |
| MCC3 | UQCRC1 | p.P283R | rhrddalPf | rhrddalRf | C0702 | 176 |
| MCC3 | VNN1 | p.G257S | hsawamGmr | hsawamSmr | A3301 | 208 |
| MCC3 | VNN1 | p.G257S | fhsawamGm | fhsawamSm | C0702 | 358 |
| MCC3 | VNN1 | p.G257S | fhsawamGm | fhsawamSm | C0602 | 17 |
| MCC3 | VPREB3 | p.G53S | yGvswyqqr | ySvswyqqr | A3301 | 106 |
| MCC3 | VWA2 | p.V700M | lryhqdVli | lryhqdMli | C0602 | 84 |
| MCC3 | VWA3A | p.E31K | mflEnhcir | mflKnhcir | A3301 | 17 |
| MCC3 | VWA3A | p.E31K | flEnhcirr | flKnhcirr | A3301 | 55 |
| MCC3 | WDR7 | p.S834L | tvSfgllsr | tvLfgllsr | A3301 | 49 |
| MCC3 | WDR7 | p.S834L | mlkphctvS | mlkphctvL | C0702 | 343 |
| MCC3 | WNK4 | p.R736Q | erdgflrRi | erdgflrQi | C0602 | 500 |
| MCC3 | WNT8A | p.L242F | hwvpaeafL | hwvpaeafF | A2402 | 475 |
| MCC3 | WNT8A | p.L242F | hwvpaeafL | hwvpaeafF | C0702 | 392 |
| MCC3 | WNT8A | p.L242F | fLpsaeael | fFpsaeael | C0702 | 373 |
| MCC3 | XAB2 | p.M613I | Mavyeratr | Iavyeratr | A3301 | 122 |
| MCC3 | XDH | p.P1013S | Pflnqagal | Sflnqagal | C0702 | 330 |
| MCC3 | ZAN | p.P1149L | Pyagtatcl | Lyagtatcl | A2402 | 162 |
| MCC3 | ZBTB11 | p.C656Y | eficsiCgr | eficsiYgr | A3301 | 7 |
| MCC3 | ZBTB11 | p.C656Y | iCgrtlpkl | iYgrtlpkl | A2402 | 71 |
| MCC3 | ZBTB17 | p.R28W | hllsRlpal | hllsWlpal | C0702 | 101 |
| MCC3 | ZFHX4 | p.L1959P | klfsnvLil | klfsnvPil | C0702 | 368 |
| MCC3 | ZFP69B | p.D265N | niDlvnhsr | niNlvnhsr | A3301 | 13 |
| MCC3 | ZMAT4 | p.H99Y | hyqgkiHak | hyqgkiYak | A3301 | 209 |
| MCC3 | ZMAT4 | p.H99Y | iHakrlkll | iYakrlkll | A2402 | 38 |
| MCC3 | ZNF251 | p.P236L | Pyecgrcgr | Lyecgrcgr | A3301 | 373 |
| MCC3 | ZNF251 | p.P236L | qrshtgekP | qrshtgekL | C0602 | 329 |
| MCC3 | ZNF45 | p.V92E | etlqeVglr | etlqeEglr | A3301 | 71 |
| MCC3 | ZNF615 | p.P455S | thtgekPyv | thtgekSyv | C0602 | 131 |
| MCC3 | ZNF791 | p.S146F | fsylkSfqr | fsylkFfqr | A3301 | 9 |
| MCC3 | ZNF883 | p.S329F | kSfslssal | kFfslssal | C0702 | 137 |
| MCC3 | ZSCAN4 | p.D147G | lfseDmplr | lfseGmplr | A3301 | 113 |
| MCC33 | A2ML1 | p.V137M | ytpgqqVyf | ytpgqqMyf | A2402 | 476 |
| MCC33 | ABCC6 | p.G741E | fpeGihtsi | fpeEihtsi | B0702 | 130 |
| MCC33 | ADAM15 | p.P466L | rpsgwqcrP | rpsgwqcrL | B0702 | 31 |
| MCC33 | ADAM22 | p.E867K | sarlwEtsi | sarlwKtsi | B0702 | 226 |
| MCC33 | ANO2 | p.S501F | kmlkesnqS | kmlkesnqF | A2402 | 350 |
| MCC33 | APP | p.P109S | Pyrclvgef | Syrclvgef | A2402 | 310 |
| MCC33 | ASAH1 | p.V104L | Vpsgkimqv | Lpsgkimqv | B0702 | 435 |
| MCC33 | BTN3A3 | p.S56L | apyrgrtSi | apyrgrtLi | B0702 | 16 |
| MCC33 | C17orf47 | p.F515L | qpiqrFtaf | qpiqrLtaf | B0702 | 48 |
| MCC33 | C19orf55 | p.S77F | wpsssgtpS | wpsssgtpF | B0702 | 11 |
| MCC33 | CASR | p.S497F | Spedgsivf | Fpedgsivf | B0702 | 246 |
| MCC33 | CASR | p.S497F | ysiinwhlS | ysiinwhlF | A2402 | 23 |
| MCC33 | CD8B | p.E173K | Eplspnacm | Kplspnacm | B0702 | 27 |
| MCC33 | CNTN3 | p.D543N | yfngalaDf | yfngalaNf | A2402 | 152 |
| MCC33 | COL16A1 | p.G788E | epgppgrGv | epgppgrEv | B0702 | 267 |
| MCC33 | COL6A6 | p.D879N | svlqnDqam | svlqnNqam | B0702 | 167 |
| MCC33 | CRX | p.G231D | vpqlgGpal | vpqlgDpal | B0702 | 29 |
| MCC33 | CSMD3 | p.P3151S | sfscnfPfi | sfscnfSfi | A2402 | 193 |
| MCC33 | CSMD3 | p.P3151S | vsfscnfPf | vsfscnfSf | A2402 | 353 |
| MCC33 | DIRAS3 | p.T184I | afmeisakT | afmeisakI | A2402 | 227 |
| MCC33 | DMBT1 | p.S1462F | tSssnfmsi | tFssnfmsi | A2402 | 44 |
| MCC33 | DNAH6 | p.S1795L | tyvlnpkSi | tyvlnpkLi | A2402 | 28 |
| MCC33 | DUSP4 | p.P269S | tpatPtsqf | tpatStsqf | B0702 | 35 |
| MCC33 | DUSP4 | p.P269S | Ptsqfvfsf | Stsqfvfsf | A2402 | 290 |
| MCC33 | ENPP1 | p.D622N | ftrnprDnl | ftrnprNnl | B0702 | 74 |
| MCC33 | ERGIC1 | p.A26T | tytgAiisi | tytgTiisi | A2402 | 166 |
| MCC33 | ERGIC1 | p.A26T | qptytgAii | qptytgTii | B0702 | 369 |
| MCC33 | EXO5 | p.P206S | rpmlPleaq | rpmlSleaq | B0702 | 370 |
| MCC33 | EXOSC2 | p.T128R | avslhTrsl | avslhRrsl | B0702 | 18 |
| MCC33 | FAM171A1 | p.S235N | vplatqsSl | vplatqsNl | B0702 | 77 |
| MCC33 | FAM71A | p.G35R | lykGeydif | lykReydif | A2402 | 69 |
| MCC33 | FCF1 | p.S53F | vpqhpSclf | vpqhpFclf | B0702 | 466 |
| MCC33 | FCHO1 | p.D29N | rvssDklal | rvssNklal | B0702 | 20 |
| MCC33 | FCHO2 | p.S476F | aplaraesS | aplaraesF | B0702 | 34 |
| MCC33 | FNDC1 | p.S1579L | rpptttepS | rpptttepL | B0702 | 14 |
| MCC33 | GALNT18 | p.P312S | rylnpPkaw | rylnpSkaw | A2402 | 369 |
| MCC33 | GALNT18 | p.P312S | npPkawwkl | npSkawwkl | B0702 | 146 |
| MCC33 | GPATCH4 | p.E234K | hpehaEqni | hpehaKqni | B0702 | 272 |
| MCC33 | GTF3C2 | p.E352V | lpqEekspl | lpqVekspl | B0702 | 20 |
| MCC33 | HCN1 | p.S871F | lpreSssvl | lpreFssvl | B0702 | 10 |
| MCC33 | HERC3 | p.S143F | vScgnwhcl | vFcgnwhcl | A2402 | 230 |
| MCC33 | HHIPL2 | p.S294L | kfyiyyScl | kfyiyyLcl | A2402 | 250 |
| MCC33 | HIST1H2AH | p.S20P | rsSraglqf | rsPraglqf | A2402 | 328 |
| MCC33 | HIST1H2AH | p.S20P | ktrsSragl | ktrsPragl | B0702 | 31 |
| MCC33 | HSD3B2 | p.G193E | iygeGgpfl | iygeEgpfl | A2402 | 228 |
| MCC33 | HYDIN | p.P861S | vpPetdvql | vpSetdvql | B0702 | 277 |
| MCC33 | ICAM3 | p.A13T | wprAcwtll | wprTcwtll | B0702 | 8 |
| MCC33 | ICAM3 | p.A13T | lwprAcwtl | lwprTcwtl | A2402 | 14 |
| MCC33 | IRAK4 | p.H166Y | fHsfsfyel | fYsfsfyel | A2402 | 54 |
| MCC33 | KEL | p.S507F | Svlscvrsl | Fvlscvrsl | B0702 | 384 |
| MCC33 | KIAA0195 | p.S1027F | pSrcswetf | pFrcswetf | A2402 | 415 |
| MCC33 | KIAA0391 | p.R326C | fpkvResql | fpkvCesql | B0702 | 97 |
| MCC33 | KIAA1107 | p.G717S | npertnGtl | npertnStl | B0702 | 31 |
| MCC33 | LDHC | p.L134F | hyspdckiL | hyspdckiF | A2402 | 326 |
| MCC33 | MGAM | p.M474I | gpydrgsdM | gpydrgsdI | B0702 | 116 |
| MCC33 | MMP13 | p.S146F | kvwSdvtpl | kvwFdvtpl | B0702 | 293 |
| MCC33 | MYO15A | p.R1923S | rfRslrhki | rfSslrhki | A2402 | 331 |
| MCC33 | NAALADL1 | p.E59K | rirEnlrel | rirKnlrel | B0702 | 18 |
| MCC33 | NR4A2 | p.S455T | rlayrSnpv | rlayrTnpv | B0702 | 71 |
| MCC33 | NYAP2 | p.D225N | lprDsslsq | lprNsslsq | B0702 | 69 |
| MCC33 | OR2Z1 | p.S275F | spqqdnvvS | spqqdnvvF | B0702 | 48 |
| MCC33 | OR51F2 | p.P44L | ywisiPfcl | ywisiLfcl | A2402 | 53 |
| MCC33 | OR51F2 | p.P44L | tqywisiPf | tqywisiLf | A2402 | 316 |
| MCC33 | OR6C2 | p.D264N | kpsakDeva | kpsakNeva | B0702 | 496 |
| MCC33 | OR6Q1 | p.N224S | sygNivwtl | sygSivwtl | A2402 | 10 |
| MCC33 | PC | p.H560Y | avrnHpgll | avrnYpgll | B0702 | 122 |
| MCC33 | PDIA4 | p.A451V | dfpeytfAi | dfpeytfVi | A2402 | 276 |
| MCC33 | PDPR | p.G723S | ffafwGqdi | ffafwSqdi | A2402 | 116 |
| MCC33 | PLA2G4A | p.E589K | sppfkElll | sppfkKlll | B0702 | 198 |
| MCC33 | PLXNA4 | p.S231L | kipSdtfti | kipLdtfti | A2402 | 413 |
| MCC33 | PPP1R14D | p.S51F | iprSrrpsr | iprFrrpsr | B0702 | 249 |
| MCC33 | PPP1R3A | p.E1005K | svEkslgpm | svKkslgpm | B0702 | 244 |
| MCC33 | PSAP | p.L114F | yLpvildii | yFpvildii | A2402 | 357 |
| MCC33 | PSAP | p.L114F | syLpvildi | syFpvildi | A2402 | 105 |
| MCC33 | RBM11 | p.Q83K | rpinvQyrf | rpinvKyrf | B0702 | 99 |
| MCC33 | RBM12 | p.S924T | gSrkvklvl | gTrkvklvl | B0702 | 478 |
| MCC33 | RC3H2 | p.R761K | lpRepcghl | lpKepcghl | B0702 | 107 |
| MCC33 | REG1A | p.S45F | ayrSycyyf | ayrFycyyf | A2402 | 31 |
| MCC33 | RPL3 | p.R212C | hpaRvafsv | hpaCvafsv | B0702 | 28 |
| MCC33 | RPL3 | p.R212C | awhpaRvaf | awhpaCvaf | A2402 | 446 |
| MCC33 | RYR2 | p.H3949Y | lHvfahmqm | lYvfahmqm | A2402 | 293 |
| MCC33 | SAMD13 | p.P25R | Ppdpadwav | Rpdpadwav | B0702 | 70 |
| MCC33 | SFMBT2 | p.Y220F | rlrlrYvgl | rlrlrFvgl | B0702 | 116 |
| MCC33 | SIAE | p.S181F | qypigliaS | qypigliaF | A2402 | 413 |
| MCC33 | SIGLEC12 | p.S439F | appalatpS | appalatpF | B0702 | 71 |
| MCC33 | SLC19A3 | p.S176F | syfylnviS | syfylnviF | A2402 | 204 |
| MCC33 | SLC1A3 | p.V96G | mlqmlVlpl | mlqmlGlpl | B0702 | 323 |
| MCC33 | SLC25A24 | p.S172F | kSgqwwrql | kFgqwwrql | A2402 | 201 |
| MCC33 | SLC4A11 | p.T258I | tfaTmfsdi | tfaImfsdi | A2402 | 193 |
| MCC33 | SLC5A4 | p.G220E | sfilmGfaf | sfilmEfaf | A2402 | 183 |
| MCC33 | SLCO1B1 | p.L263F | fLvsglfsi | fFvsglfsi | A2402 | 356 |
| MCC33 | SMC5 | p.E312A | ipvtcrieE | ipvtcrieA | B0702 | 407 |
| MCC33 | SPAM1 | p.D403N | npDnfaiql | npNnfaiql | B0702 | 182 |
| MCC33 | SPEF2 | p.M1176I | yywgMeski | yywgIeski | A2402 | 192 |
| MCC33 | SPHKAP | p.S769F | awtkatesS | awtkatesF | A2402 | 436 |
| MCC33 | ST8SIA5 | p.E145K | iyhinqEif | iyhinqKif | A2402 | 101 |
| MCC33 | TBRG1 | p.S18C | spraplqsS | spraplqsC | B0702 | 29 |
| MCC33 | TENM4 | p.C1145F | eyesCpdli | eyesFpdli | A2402 | 334 |
| MCC33 | TEX15 | p.P2364L | hprttgshP | hprttgshL | B0702 | 9 |
| MCC33 | THBS3 | p.R83P | tRwleasvv | tPwleasvv | B0702 | 382 |
| MCC33 | TRIOBP | p.P1030S | rasspPryl | rasspSryl | B0702 | 113 |
| MCC33 | TRIOBP | p.P1030S | aprasspPr | aprasspSr | B0702 | 473 |
| MCC33 | TRPS1 | p.P1102S | qyplfglPf | qyplfglSf | A2402 | 53 |
| MCC33 | TRPS1 | p.P1102S | yplfglPfv | yplfglSfv | B0702 | 109 |
| MCC33 | UNC79 | p.P1494S | svPshpsvl | svSshpsvl | B0702 | 259 |
| MCC33 | VPS4B | p.K255N | aarriKtef | aarriNtef | B0702 | 496 |
| MCC33 | ZC3HC1 | p.P301L | vpesPrrmm | vpesLrrmm | B0702 | 28 |
| MCC33 | ZEB2 | p.P486L | spnipPvgl | spnipLvgl | B0702 | 29 |
| MCC33 | ZEB2 | p.P486L | ipPvglpvv | ipLvglpvv | B0702 | 343 |
| MCC33 | ZNF438 | p.P711L | kpretcqgP | kpretcqgL | B0702 | 16 |
| MCC35 | ABCA4 | p.G2100R | lldepttGm | lldepttRm | A0201 | 288 |
| MCC35 | ABCA8 | p.V944A | rlncfpVlm | rlncfpAlm | A0201 | 239 |
| MCC35 | ABCA8 | p.V944A | Vlmdivsng | Almdivsng | A0201 | 335 |
| MCC35 | AGPS | p.P206S | gmferiPdi | gmferiSdi | A0201 | 30 |
| MCC35 | AKAP9 | p.P1381S | stvPpslpv | stvSpslpv | A0201 | 432 |
| MCC35 | APCS | p.H38Y | svtdHvnli | svtdYvnli | A0201 | 157 |
| MCC35 | APCS | p.H38Y | vtdHvnlit | vtdYvnlit | A0101 | 397 |
| MCC35 | API5 | p.P222S | nlgtlttPv | nlgtlttSv | A0201 | 63 |
| MCC35 | API5 | p.P222S | tlttPvegl | tlttSvegl | A0201 | 402 |
| MCC35 | AQPEP | p.S534F | ksylktfSy | ksylktfFy | A0101 | 478 |
| MCC35 | ATG2A | p.R1579C | clRvslmpl | clCvslmpl | A0201 | 230 |
| MCC35 | ATP6V1E1 | p.A17V | mmAfieqea | mmVfieqea | A0201 | 137 |
| MCC35 | C1orf112 | p.S268F | flSdscctl | flFdscctl | A0201 | 3 |
| MCC35 | C7orf10 | p.P63L | rvlagPfat | rvlagLfat | A0201 | 481 |
| MCC35 | C7orf10 | p.P63L | vlagPfatm | vlagLfatm | A0201 | 123 |
| MCC35 | C7orf63 | p.R882Q | aRlvggplv | aQlvggplv | A0201 | 247 |
| MCC35 | CCAR2 | p.P650L | aldPellll | aldLellll | A0201 | 122 |
| MCC35 | CCAR2 | p.P650L | maldPelll | maldLelll | A0201 | 186 |
| MCC35 | CDH7 | p.F482C | Famdyettv | Camdyettv | A0201 | 188 |
| MCC35 | CDIP1 | p.P7S | msseppPpy | msseppSpy | A0101 | 50 |
| MCC35 | COL6A5 | p.M1962I | syMdvvfli | syIdvvfli | A0201 | 276 |
| MCC35 | CPM | p.E277K | yiwaqcfEi | yiwaqcfKi | A0201 | 5 |
| MCC35 | CYBRD1 | p.S140L | fSvfllpwa | fLvfllpwa | A0201 | 7 |
| MCC35 | CYBRD1 | p.S140L | lqllsgfSv | lqllsgfLv | A0201 | 79 |
| MCC35 | CYBRD1 | p.S140L | llsgfSvfl | llsgfLvfl | A0201 | 8 |
| MCC35 | DCHS2 | p.R623G | tldRevqea | tldGevqea | A0201 | 426 |
| MCC35 | DHX32 | p.G398E | ssGkffcly | ssEkffcly | A0101 | 9 |
| MCC35 | DIP2C | p.S1202F | Silippsel | Filippsel | A0201 | 183 |
| MCC35 | DNAH14 | p.P1780T | fPevtvlkv | fTevtvlkv | A0101 | 220 |
| MCC35 | DNAH14 | p.P1780T | iigdifPev | iigdifTev | A0201 | 27 |
| MCC35 | DNAH9 | p.D2041N | kqdhyDwgl | kqdhyNwgl | A0201 | 26 |
| MCC35 | DNAJC19 | p.L40I | Lilgvspta | Iilgvspta | A0201 | 139 |
| MCC35 | DZIP3 | p.P444S | Plggswhll | Slggswhll | A0201 | 67 |
| MCC35 | FAM71F1 | p.D181Y | klhpDhpet | klhpYhpet | A0201 | 324 |
| MCC35 | FLVCR1 | p.P444S | ffmtgylPl | ffmtgylSl | A0201 | 447 |
| MCC35 | FLVCR1 | p.P444S | lPlgfefav | lSlgfefav | A0201 | 291 |
| MCC35 | FLVCR1 | p.P444S | ylPlgfefa | ylSlgfefa | A0201 | 31 |
| MCC35 | GCKR | p.R301Q | rtfeRahqv | rtfeQahqv | A0201 | 127 |
| MCC35 | HBE1 | p.P52L | nlssPsail | nlssLsail | A0201 | 299 |
| MCC35 | HEATR5A | p.P1793L | alkgilssP | alkgilssL | A0201 | 306 |
| MCC35 | HEATR5A | p.P1793L | ilssPmara | ilssLmara | A0201 | 75 |
| MCC35 | HHIPL2 | p.P585L | flatsyPsa | flatsyLsa | A0201 | 13 |
| MCC35 | INTS8 | p.I51L | lIvqfleqa | lLvqfleqa | A0201 | 215 |
| MCC35 | ITGA10 | p.G1132R | svlGgllll | svlRgllll | A0201 | 254 |
| MCC35 | KIAA1468 | p.A706V | athqvflpA | athqvflpV | A0201 | 430 |
| MCC35 | KIAA1468 | p.A706V | flpAyaawt | flpVyaawt | A0201 | 339 |
| MCC35 | LAMC2 | p.P299S | ritaPlmpl | ritaSlmpl | A0201 | 133 |
| MCC35 | LAMC2 | p.P299S | Plmplgktl | Slmplgktl | A0201 | 120 |
| MCC35 | LEPROT | p.S45F | lifhaiSpi | lifhaiFpi | A0201 | 5 |
| MCC35 | LEPROT | p.S45F | aiSpiphfi | aiFpiphfi | A0201 | 26 |
| MCC35 | LETMD1 | p.C7F | malsrvCwa | malsrvFwa | A0201 | 125 |
| MCC35 | LRRC16B | p.S302F | qllcfpSgl | qllcfpFgl | A0201 | 11 |
| MCC35 | LRRC8C | p.F248L | alfekvkkF | alfekvkkL | A0201 | 60 |
| MCC35 | MAP7 | p.S141L | lltpthSfl | lltpthLfl | A0201 | 70 |
| MCC35 | MDGA2 | p.E375K | slsnEnygv | slsnKnygv | A0201 | 16 |
| MCC35 | MDGA2 | p.E375K | lsnEnygvy | lsnKnygvy | A0101 | 105 |
| MCC35 | MFSD2A | p.S219F | fqdlnsStv | fqdlnsFtv | A0201 | 10 |
| MCC35 | MKL1 | p.P199L | sltngttiP | sltngttiL | A0201 | 160 |
| MCC35 | MYCBP2 | p.S4018F | ycfellSmv | ycfellFmv | A0201 | 8 |
| MCC35 | NAV3 | p.D2296N | llqlrpeDv | llqlrpeNv | A0201 | 376 |
| MCC35 | NR4A3 | p.N439S | Nlltasidv | Slltasidv | A0201 | 27 |
| MCC35 | OPRM1 | p.I150T | impvliItv | impvliTtv | A0201 | 28 |
| MCC35 | OPRM1 | p.I150T | liItvcygl | liTtvcygl | A0201 | 293 |
| MCC35 | OPRM1 | p.I150T | fimpvliIt | fimpvliTt | A0201 | 24 |
| MCC35 | OR1N2 | p.I238V | rIfwavfvi | rVfwavfvi | A0201 | 57 |
| MCC35 | OR2L8 | p.M58L | hlhtpMyfl | hlhtpLyfl | A0201 | 36 |
| MCC35 | OR2L8 | p.M58L | dthlhtpMy | dthlhtpLy | A0101 | 458 |
| MCC35 | OR5H2 | p.P292S | llnPiiysl | llnSiiysl | A0201 | 6 |
| MCC35 | OR5H2 | p.P292S | iiipllnPi | iiipllnSi | A0201 | 122 |
| MCC35 | OR7D2 | p.P108S | Pildtlllt | Sildtlllt | A0201 | 371 |
| MCC35 | OR7D2 | p.P108S | smffPildt | smffSildt | A0201 | 404 |
| MCC35 | OSBP2 | p.E171K | alqrsltEl | alqrsltKl | A0201 | 305 |
| MCC35 | PAPLN | p.H432Y | llHtaacsl | llYtaacsl | A0201 | 39 |
| MCC35 | PCDHB1 | p.R751W | gslsRpcpy | gslsWpcpy | A0101 | 325 |
| MCC35 | PDGFRA | p.S46F | vqlnssfSl | vqlnssfFl | A0201 | 25 |
| MCC35 | PGLYRP2 | p.R65C | Rlyhfllga | Clyhfllga | A0201 | 56 |
| MCC35 | PGLYRP2 | p.R65C | nsgphnRly | nsgphnCly | A0101 | 29 |
| MCC35 | PHLPP2 | p.A986T | Alwehlsyt | Tlwehlsyt | A0201 | 12 |
| MCC35 | PKD1L1 | p.F526V | fatdtditF | fatdtditV | A0201 | 176 |
| MCC35 | PSPH | p.E64K | altErlali | altKrlali | A0201 | 208 |
| MCC35 | RANBP17 | p.V310I | ylgnlikgV | ylgnlikgI | A0201 | 61 |
| MCC35 | RP1 | p.E2138K | fiweEedil | fiweKedil | A0201 | 46 |
| MCC35 | SCN9A | p.E364K | tqdywEnly | tqdywKnly | A0101 | 243 |
| MCC35 | SERPINC1 | p.G390S | vaeGrddly | vaeSrddly | A0101 | 209 |
| MCC35 | SLC12A1 | p.M486I | glmnnfqvM | glmnnfqvI | A0201 | 72 |
| MCC35 | SLC12A1 | p.T566I | iliaelnTi | iliaelnIi | A0201 | 95 |
| MCC35 | SLC12A1 | p.T566I | liaelnTia | liaelnIia | A0201 | 457 |
| MCC35 | SLC12A1 | p.T566I | filiaelnT | filiaelnI | A0201 | 209 |
| MCC35 | SLC2A12 | p.G282E | iGltlvffv | iEltlvffv | A0201 | 433 |
| MCC35 | SLC2A12 | p.G282E | rimiGltlv | rimiEltlv | A0201 | 43 |
| MCC35 | SLCO1B1 | p.G584E | algGilapi | algEilapi | A0201 | 16 |
| MCC35 | SUCNR1 | p.H103Y | vlHanlyts | vlYanlyts | A0201 | 299 |
| MCC35 | SUCNR1 | p.H103Y | yvlHanlyt | yvlYanlyt | A0201 | 276 |
| MCC35 | SUSD4 | p.P255S | vlyfPhirl | vlyfShirl | A0201 | 70 |
| MCC35 | SUSD4 | p.P255S | hlfpvlyfP | hlfpvlyfS | A0201 | 48 |
| MCC35 | TCTN2 | p.R416K | gimtqRfvv | gimtqKfvv | A0201 | 107 |
| MCC35 | TNFSF10 | p.R191Q | yiysqtyfR | yiysqtyfQ | A0201 | 436 |
| MCC35 | UNC80 | p.S498F | fsfgsfSgl | fsfgsfFgl | A0201 | 44 |
| MCC35 | USP25 | p.L452F | lvdvLqyal | lvdvFqyal | A0201 | 326 |
| MCC35 | USP31 | p.S1111L | sqdsvsspS | sqdsvsspL | A0201 | 241 |
| MCC35 | USP39 | p.A133V | kandyAnav | kandyVnav | A0201 | 113 |
| MCC35 | USP39 | p.A133V | yAnavlqal | yVnavlqal | A0201 | 88 |
| MCC35 | XPNPEP3 | p.P301L | ilayPpvva | ilayLpvva | A0201 | 280 |
| MCC35 | YIPF7 | p.P213S | vlgycllPm | vlgycllSm | A0201 | 215 |
| MCC35 | ZCCHC14 | p.S203F | flstsSppq | flstsFppq | A0201 | 431 |
| MCC35 | ZNF263 | p.V246M | tVqesyenv | tMqesyenv | A0201 | 66 |
| MCC35 | ZNF547 | p.S197F | ilfmerStl | ilfmerFtl | A0201 | 34 |
| MCC39 | A2M | p.L613F | aeLsassvy | aeFsassvy | B1801 | 32 |
| MCC39 | ABCA13 | p.S4514F | ydmlfylvS | ydmlfylvF | B1801 | 140 |
| MCC39 | ABCA7 | p.M370I | dhMealrsf | dhIealrsf | B1801 | 158 |
| MCC39 | ACLY | p.S564F | nfaSlrsay | nfaFlrsay | B1801 | 146 |
| MCC39 | AK9 | p.E1041K | tekkvgpEf | tekkvgpKf | B1801 | 41 |
| MCC39 | AK9 | p.R316C | delfRtlas | delfCtlas | B1801 | 30 |
| MCC39 | AKAP10 | p.A431T | Aqndamily | Tqndamily | B1801 | 443 |
| MCC39 | AKR1D1 | p.E63K | nehEvgeai | nehKvgeai | B1801 | 85 |
| MCC39 | ALS2CR11 | p.P98L | qetPnlvpf | qetLnlvpf | B1801 | 9 |
| MCC39 | AMER3 | p.S220N | Selladasf | Nelladasf | B1801 | 5 |
| MCC39 | ANKMY2 | p.P275S | resirkfPy | resirkfSy | B1801 | 136 |
| MCC39 | AP4E1 | p.S1047F | ddfgklwlS | ddfgklwlF | B1801 | 26 |
| MCC39 | ARHGEF26 | p.D705N | eesynvnDy | eesynvnNy | B1801 | 86 |
| MCC39 | ARMC12 | p.G235E | sGsllyevl | sEsllyevl | B1801 | 296 |
| MCC39 | ARMC3 | p.M437I | eevMvvpkf | eevIvvpkf | B1801 | 17 |
| MCC39 | ATP1A3 | p.H675Y | deilqnHte | deilqnYte | B1801 | 46 |
| MCC39 | BHMT2 | p.R127K | deaRikklf | deaKikklf | B1801 | 9 |
| MCC39 | C11orf24 | p.P315L | Pemeamspt | Lemeamspt | B1801 | 106 |
| MCC39 | C20orf194 | p.H791Y | secfHaahf | secfYaahf | B1801 | 88 |
| MCC39 | CACNB4 | p.R391Q | ierRslmts | ierQslmts | B1801 | 403 |
| MCC39 | CACNG7 | p.R174C | seqyfhyRy | seqyfhyCy | B1801 | 5 |
| MCC39 | CACNG7 | p.R174C | fhyRygwsf | fhyCygwsf | B1801 | 187 |
| MCC39 | CAPN8 | p.P126L | eellyrvvP | eellyrvvL | B1801 | 116 |
| MCC39 | CCDC87 | p.P543S | Pelvglysq | Selvglysq | B1801 | 87 |
| MCC39 | CCKAR | p.S353F | iSfilllsy | iFfilllsy | B1801 | 229 |
| MCC39 | CDC37 | p.R235W | dpracfRqf | dpracfWqf | B1801 | 162 |
| MCC39 | CDK12 | p.R882W | adfglaRly | adfglaWly | B1801 | 311 |
| MCC39 | CEP85L | p.K463I | neflKqrls | neflIqrls | B1801 | 207 |
| MCC39 | CILP | p.R737C | leiRerrlf | leiCerrlf | B1801 | 171 |
| MCC39 | CLTC | p.G1315E | mGmftelai | mEmftelai | B1801 | 137 |
| MCC39 | CLTC | p.G1315E | lerahmGmf | lerahmEmf | B1801 | 43 |
| MCC39 | CNBD1 | p.L338F | yelialLkw | yelialFkw | B1801 | 16 |
| MCC39 | CNPY4 | p.D99N | cerilDysv | cerilNysv | B1801 | 220 |
| MCC39 | CNTN6 | p.E820K | semEvswna | semKvswna | B1801 | 404 |
| MCC39 | CPA2 | p.S89F | lesqgiayS | lesqgiayF | B1801 | 28 |
| MCC39 | CPA6 | p.G171R | yeGrslfil | yeRrslfil | B1801 | 55 |
| MCC39 | CPAMD8 | p.A1430V | vemtayAll | vemtayVll | B1801 | 270 |
| MCC39 | CPAMD8 | p.A1430V | mtayAllty | mtayVllty | B1801 | 268 |
| MCC39 | CPNE8 | p.R94C | renlRfdly | renlCfdly | B1801 | 310 |
| MCC39 | CRIM1 | p.G634E | nGremcali | nEremcali | B1801 | 144 |
| MCC39 | CRLS1 | p.P110S | yenpwtiPn | yenpwtiSn | B1801 | 477 |
| MCC39 | CRYAB | p.S115F | dehgfiSre | dehgfiFre | B1801 | 156 |
| MCC39 | CST9L | p.R45C | dehnvmaRy | dehnvmaCy | B1801 | 5 |
| MCC39 | CYTIP | p.A16T | Adfcagpay | Tdfcagpay | B1801 | 85 |
| MCC39 | DAXX | p.E484K | Eespvsqlf | Kespvsqlf | B1801 | 375 |
| MCC39 | DLEC1 | p.G1072E | qGlqvaiti | qElqvaiti | B1801 | 102 |
| MCC39 | DNAH1 | p.L3700F | eemkfskkL | eemkfskkF | B1801 | 69 |
| MCC39 | DNAH3 | p.S2561F | iervinkiS | iervinkiF | B1801 | 61 |
| MCC39 | DNPEP | p.S228L | derhhSvlm | derhhLvlm | B1801 | 35 |
| MCC39 | DNTT | p.E358K | deEqllqkv | deKqllqkv | B1801 | 224 |
| MCC39 | DOCK4 | p.P1875L | neqsaPlpv | neqsaLlpv | B1801 | 24 |
| MCC39 | DOCK8 | p.G1734D | leafyGqcf | leafyDqcf | B1801 | 90 |
| MCC39 | DOK6 | p.G205R | dtGeglftf | dtReglftf | B1801 | 116 |
| MCC39 | DPEP2 | p.S164F | Selelvtsa | Felelvtsa | B1801 | 25 |
| MCC39 | DST | p.P1144S | aekagkpPf | aekagkpSf | B1801 | 495 |
| MCC39 | DYNC2H1 | p.D2714N | Dyqfvhptf | Nyqfvhptf | B1801 | 82 |
| MCC39 | DYRK3 | p.L388F | aelltgqpL | aelltgqpF | B1801 | 44 |
| MCC39 | ECT2 | p.P238S | nefkvPpfq | nefkvSpfq | B1801 | 28 |
| MCC39 | EIF4ENIF1 | p.S230F | Setieltgf | Fetieltgf | B1801 | 204 |
| MCC39 | EIF4G3 | p.R1124K | vefdsrRtl | vefdsrKtl | B1801 | 81 |
| MCC39 | ENTHD1 | p.P316S | tenlletPl | tenlletSl | B1801 | 359 |
| MCC39 | EPG5 | p.S1263F | iegelvinS | iegelvinF | B1801 | 11 |
| MCC39 | EPG5 | p.S1263F | gelvinSaf | gelvinFaf | B1801 | 31 |
| MCC39 | F13A1 | p.D272N | Degvlvgsw | Negvlvgsw | B1801 | 75 |
| MCC39 | FAT4 | p.P4143L | mefavngrP | mefavngrL | B1801 | 250 |
| MCC39 | FCAR | p.R57Q | Reigrrlkf | Qeigrrlkf | B1801 | 86 |
| MCC39 | FIGN | p.G412E | lGsrssesf | lEsrssesf | B1801 | 351 |
| MCC39 | FILIP1L | p.S419F | dedpndegS | dedpndegF | B1801 | 148 |
| MCC39 | FILIP1L | p.S419F | ndegSvlsf | ndegFvlsf | B1801 | 292 |
| MCC39 | FRMPD1 | p.G70E | yGfhisesl | yEfhisesl | B1801 | 25 |
| MCC39 | FRY | p.R2065K | feylmalRl | feylmalKl | B1801 | 41 |
| MCC39 | GABBR2 | p.M653I | centhMtiw | centhItiw | B1801 | 195 |
| MCC39 | GABRD | p.T86I | meyTmtvfl | meyImtvfl | B1801 | 34 |
| MCC39 | GABRD | p.T86I | seanmeyTm | seanmeyIm | B1801 | 196 |
| MCC39 | GAL3ST1 | p.D147N | Devrglvpt | Nevrglvpt | B1801 | 224 |
| MCC39 | GIMAP1-GIMAP5 | p.G279E | nGrkvlvvd | nErkvlvvd | B1801 | 109 |
| MCC39 | GLI1 | p.R40W | tevkltkkR | tevkltkkW | B1801 | 405 |
| MCC39 | GLP1R | p.G295S | yedeGcwtr | yedeScwtr | B1801 | 95 |
| MCC39 | GRAMD1B | p.R443C | dyfytinRy | dyfytinCy | B1801 | 167 |
| MCC39 | GTDC1 | p.T39I | Telaalrpd | Ielaalrpd | B1801 | 351 |
| MCC39 | HARBI1 | p.E323K | meqppEeey | meqppKeey | B1801 | 81 |
| MCC39 | HEATR5A | p.P686S | yellillpP | yellillpS | B1801 | 17 |
| MCC39 | HENMT1 | p.F266L | qerFfklvl | qerLfklvl | B1801 | 140 |
| MCC39 | HIF3A | p.E532K | pehEnfllf | pehKnfllf | B1801 | 457 |
| MCC39 | HIP1R | p.R113W | Reigdlwgh | Weigdlwgh | B1801 | 21 |
| MCC39 | HIVEP1 | p.T2288A | dendTipsv | dendAipsv | B1801 | 16 |
| MCC39 | HPN | p.R181C | wpwqvslRy | wpwqvslCy | B1801 | 149 |
| MCC39 | HSH2D | p.S142F | yedlflySn | yedlflyFn | B1801 | 389 |
| MCC39 | IBSP | p.R42Q | Ryylykhay | Qyylykhay | B1801 | 184 |
| MCC39 | IFNG | p.S74F | mqsqivSfy | mqsqivFfy | B1801 | 58 |
| MCC39 | IFT140 | p.S986F | yelardhfS | yelardhfF | B1801 | 15 |
| MCC39 | IGDCC4 | p.V879M | Veylilyss | Meylilyss | B1801 | 7 |
| MCC39 | IQCK | p.H104Y | aehyfpvsH | aehyfpvsY | B1801 | 49 |
| MCC39 | IRF2 | p.G102E | kGnnafrvy | kEnnafrvy | B1801 | 65 |
| MCC39 | ITGA6 | p.G416E | nGintkptq | nEintkptq | B1801 | 90 |
| MCC39 | ITIH2 | p.P218L | iePqglrfl | ieLqglrfl | B1801 | 174 |
| MCC39 | IZUMO2 | p.D179N | lqyqmDsky | lqyqmNsky | B1801 | 430 |
| MCC39 | JPH4 | p.P453S | yengltPse | yengltSse | B1801 | 176 |
| MCC39 | KCNH7 | p.G281E | eGfgvhpkn | eEfgvhpkn | B1801 | 214 |
| MCC39 | KCNH8 | p.V392A | weVgwlhel | weAgwlhel | B1801 | 10 |
| MCC39 | KCNT1 | p.M309I | eelvylwMe | eelvylwIe | B1801 | 202 |
| MCC39 | KDM7A | p.S805F | tedpdlrtS | tedpdlrtF | B1801 | 59 |
| MCC39 | KIAA1107 | p.P905L | deiPrkrpe | deiLrkrpe | B1801 | 458 |
| MCC39 | KIAA1199 | p.G104E | nGgelhags | nEgelhags | B1801 | 202 |
| MCC39 | KIAA1984 | p.G298S | meyqsGvta | meyqsSvta | B1801 | 86 |
| MCC39 | KLRG1 | p.V183A | cevplhwVc | cevplhwAc | B1801 | 80 |
| MCC39 | L3MBTL3 | p.D105N | Declsggny | Neclsggny | B1801 | 41 |
| MCC39 | LCP1 | p.G235E | iGlfadiel | iElfadiel | B1801 | 273 |
| MCC39 | LHCGR | p.G183E | yGngfeevq | yEngfeevq | B1801 | 394 |
| MCC39 | LILRB5 | p.M440I | veMdaraaa | veIdaraaa | B1801 | 464 |
| MCC39 | LNX2 | p.S478F | heSlgmtva | heFlgmtva | B1801 | 68 |
| MCC39 | LOXHD1 | p.K327E | nKnsgkifl | nEnsgkifl | B1801 | 147 |
| MCC39 | LPIN2 | p.V619M | qeleesitV | qeleesitM | B1801 | 35 |
| MCC39 | LRP1B | p.G1712E | dGntinman | dEntinman | B1801 | 125 |
| MCC39 | LRP1B | p.P647S | legemshPr | legemshSr | B1801 | 351 |
| MCC39 | LSS | p.G442E | Gdimidyty | Edimidyty | B1801 | 231 |
| MCC39 | MAGEL2 | p.P927L | wevqsPiqv | wevqsLiqv | B1801 | 132 |
| MCC39 | MAMDC2 | p.G256E | qGndnvfsl | qEndnvfsl | B1801 | 47 |
| MCC39 | MANBA | p.A823V | letsAvapf | letsVvapf | B1801 | 7 |
| MCC39 | MAVS | p.H206Y | telgstHta | telgstYta | B1801 | 82 |
| MCC39 | MAVS | p.H206Y | qdtelgstH | qdtelgstY | B1801 | 342 |
| MCC39 | MBIP | p.L283F | Lelegispe | Felegispe | B1801 | 92 |
| MCC39 | MGAT4A | p.H529Y | neiHikkat | neiYikkat | B1801 | 407 |
| MCC39 | MMP12 | p.S455F | nqfeydflL | nqfeydflF | B1801 | 29 |
| MCC39 | MMP12 | p.S455F | feydflLqr | feydflFqr | B1801 | 78 |
| MCC39 | MMP8 | p.W350G | nqyWalsgy | nqyGalsgy | B1801 | 31 |
| MCC39 | MON2 | p.S1518F | fetllqfSf | fetllqfFf | B1801 | 18 |
| MCC39 | MRPS27 | p.S151F | eeernfgaS | eeernfgaF | B1801 | 337 |
| MCC39 | MTBP | p.P449L | eeaklsfPf | eeaklsfLf | B1801 | 25 |
| MCC39 | MUC16 | p.S8855F | vektsppsS | vektsppsF | B1801 | 424 |
| MCC39 | MYOF | p.S729L | heaavrmrS | heaavrmrL | B1801 | 290 |
| MCC39 | MYOZ2 | p.P168S | lealyPklf | lealySklf | B1801 | 80 |
| MCC39 | NAALAD2 | p.G417E | eefGllgst | eefEllgst | B1801 | 174 |
| MCC39 | NAALAD2 | p.G417E | fGllgstew | fEllgstew | B1801 | 175 |
| MCC39 | NDST4 | p.G410E | lehGipinm | lehEipinm | B1801 | 74 |
| MCC39 | NDST4 | p.G410E | hGipinmgy | hEipinmgy | B1801 | 10 |
| MCC39 | NES | p.E566K | lenqshEtl | lenqshKtl | B1801 | 240 |
| MCC39 | NFATC2IP | p.L414F | mesgdLiev | mesgdFiev | B1801 | 290 |
| MCC39 | NGF | p.P112L | fevggaaPf | fevggaaLf | B1801 | 15 |
| MCC39 | NLRP8 | p.A516V | qeffaAlfy | qeffaVlfy | B1801 | 4 |
| MCC39 | NPHP3 | p.A1032T | releAlatl | releTlatl | B1801 | 35 |
| MCC39 | NPHP3 | p.P1024S | naygadhPy | naygadhSy | B1801 | 383 |
| MCC39 | NRSN1 | p.D97N | ieafgeaDf | ieafgeaNf | B1801 | 247 |
| MCC39 | NUP153 | p.S1018F | dnfkfglsS | dnfkfglsF | B1801 | 113 |
| MCC39 | OBSCN | p.R5061C | dehficiRf | dehficiCf | B1801 | 5 |
| MCC39 | OIT3 | p.E405K | feepyrEal | feepyrKal | B1801 | 320 |
| MCC39 | OLFM1 | p.M253I | reyksMvdf | reyksIvdf | B1801 | 171 |
| MCC39 | OR10A3 | p.H176Y | neinHlfce | neinYlfce | B1801 | 412 |
| MCC39 | OR14C36 | p.S200F | nevmivvSa | nevmivvFa | B1801 | 135 |
| MCC39 | OR2A14 | p.L25I | leiLlcglf | leiIlcglf | B1801 | 167 |
| MCC39 | OR2AG1 | p.G306E | lGkymlpah | lEkymlpah | B1801 | 142 |
| MCC39 | OR2M3 | p.G249E | vGmyygaal | vEmyygaal | B1801 | 49 |
| MCC39 | OR4L1 | p.F102I | tqmFfmhff | tqmIfmhff | B1801 | 393 |
| MCC39 | OR5B12 | p.G39E | vGnlgmiel | vEnlgmiel | B1801 | 230 |
| MCC39 | OR6C4 | p.P19L | Pelqvmifi | Lelqvmifi | B1801 | 205 |
| MCC39 | OR6C76 | p.G250E | yGscifmyv | yEscifmyv | B1801 | 93 |
| MCC39 | OR6K2 | p.P157S | Peiawistl | Seiawistl | B1801 | 161 |
| MCC39 | OR8D2 | p.L105F | tqlyffLif | tqlyffFif | B1801 | 170 |
| MCC39 | OR8I2 | p.S274F | tqaqvaSvf | tqaqvaFvf | B1801 | 349 |
| MCC39 | OSBPL1A | p.M202K | teyMehtyl | teyKehtyl | B1801 | 312 |
| MCC39 | OVCA2 | p.R71P | eeqpRgwwf | eeqpPgwwf | B1801 | 64 |
| MCC39 | PCDH15 | p.S1826L | lekSvanmy | lekLvanmy | B1801 | 56 |
| MCC39 | PCDH20 | p.S571L | eergqvSyf | eergqvLyf | B1801 | 42 |
| MCC39 | PCDHA12 | p.D560N | denDnapal | denNnapal | B1801 | 26 |
| MCC39 | PCDHGA12 | p.S280F | aevrySfry | aevryFfry | B1801 | 349 |
| MCC39 | PCDHGB6 | p.K135E | dKkeihlei | dEkeihlei | B1801 | 39 |
| MCC39 | PCGF6 | p.S205L | lekkfvrvS | lekkfvrvL | B1801 | 255 |
| MCC39 | PDE6A | p.E395K | Eeivgvatf | Keivgvatf | B1801 | 30 |
| MCC39 | PDIA5 | p.R124Q | teynRavtf | teynQavtf | B1801 | 10 |
| MCC39 | PIPOX | p.G83R | leheaGtql | leheaRtql | B1801 | 135 |
| MCC39 | PKD1 | p.L3476F | dedliqqvL | dedliqqvF | B1801 | 15 |
| MCC39 | PKD1L2 | p.A291V | weaslAlqh | weaslVlqh | B1801 | 101 |
| MCC39 | PKD2L2 | p.G259E | gGiltswqf | gEiltswqf | B1801 | 67 |
| MCC39 | PLCXD3 | p.G240R | terrkkGsf | terrkkRsf | B1801 | 325 |
| MCC39 | PLD1 | p.S230F | iegmimkrS | iegmimkrF | B1801 | 328 |
| MCC39 | PLEKHA1 | p.S264F | nlfeivttS | nlfeivttF | B1801 | 318 |
| MCC39 | PLEKHG4B | p.D1007Y | Defivccgr | Yefivccgr | B1801 | 424 |
| MCC39 | PPBP | p.E93K | nqvEviatl | nqvKviatl | B1801 | 464 |
| MCC39 | PREX1 | p.H152Y | Hekalrllv | Yekalrllv | B1801 | 428 |
| MCC39 | PRICKLE1 | p.G39S | eeyawvppG | eeyawvppS | B1801 | 86 |
| MCC39 | PTPRD | p.E1163K | wespdEmel | wespdKmel | B1801 | 67 |
| MCC39 | RALGAPA2 | p.L88F | eeldsilfL | eeldsilfF | B1801 | 46 |
| MCC39 | RASGRP3 | p.L362F | mdlinlltL | mdlinlltF | B1801 | 212 |
| MCC39 | RERGL | p.S157F | celsaaeqS | celsaaeqF | B1801 | 144 |
| MCC39 | RFX6 | p.D738N | teqqlsrDf | teqqlsrNf | B1801 | 62 |
| MCC39 | RGSL1 | p.R958W | tegyldRsv | tegyldWsv | B1801 | 170 |
| MCC39 | RMDN3 | p.E296K | eevsEkksy | eevsKkksy | B1801 | 224 |
| MCC39 | ROS1 | p.G192E | hGvpetapl | hEvpetapl | B1801 | 188 |
| MCC39 | RYR1 | p.E1092V | fefEavttg | fefVavttg | B1801 | 408 |
| MCC39 | RYR2 | p.L2488F | Levgflpdl | Fevgflpdl | B1801 | 74 |
| MCC39 | RYR2 | p.M2689L | mesnyvsmM | mesnyvsmL | B1801 | 188 |
| MCC39 | SAMD9 | p.N977T | teviecgNy | teviecgTy | B1801 | 142 |
| MCC39 | SAMM50 | p.S171L | ketSyglsf | ketLyglsf | B1801 | 64 |
| MCC39 | SART3 | p.D416N | Dyveiwqay | Nyveiwqay | B1801 | 471 |
| MCC39 | SDK1 | p.S2141F | yedalpkhS | yedalpkhF | B1801 | 294 |
| MCC39 | SERPINA9 | p.S180F | ievfiprfS | ievfiprfF | B1801 | 40 |
| MCC39 | SERPINB11 | p.S85F | Sefgvefsq | Fefgvefsq | B1801 | 26 |
| MCC39 | SERPINB6 | p.T328M | Teaaaataa | Meaaaataa | B1801 | 312 |
| MCC39 | SLC12A8 | p.P216L | Pellqnntl | Lellqnntl | B1801 | 490 |
| MCC39 | SLC24A1 | p.G1030E | nGlqpvpvs | nElqpvpvs | B1801 | 475 |
| MCC39 | SLC2A2 | p.G213E | vGlvllnkf | vElvllnkf | B1801 | 36 |
| MCC39 | SLC35F1 | p.G117E | qGeenllai | qEeenllai | B1801 | 299 |
| MCC39 | SLCO1B3 | p.S293L | kerkiSlsl | kerkiLlsl | B1801 | 338 |
| MCC39 | SNX24 | p.G160R | iegvlhGif | iegvlhRif | B1801 | 22 |
| MCC39 | SNX9 | p.F380L | delagvmiF | delagvmiL | B1801 | 7 |
| MCC39 | STXBP5L | p.M519I | dpfaiqMiy | dpfaiqIiy | B1801 | 44 |
| MCC39 | SULF1 | p.P115S | nencssPsw | nencssSsw | B1801 | 194 |
| MCC39 | SUN5 | p.M181I | deqkMaqki | deqkIaqki | B1801 | 420 |
| MCC39 | SYNE1 | p.R3659K | deshvnsRm | deshvnsKm | B1801 | 97 |
| MCC39 | T | p.E50K | eesElwlrf | eesKlwlrf | B1801 | 143 |
| MCC39 | TEX101 | p.A41V | veadpAnmf | veadpVnmf | B1801 | 432 |
| MCC39 | TIGD2 | p.R339Q | yRagllqky | yQagllqky | B1801 | 139 |
| MCC39 | TLN2 | p.S517N | Seldslppl | Neldslppl | B1801 | 11 |
| MCC39 | TLR1 | p.S642L | lqfhafiSy | lqfhafiLy | B1801 | 345 |
| MCC39 | TMPRSS3 | p.R106C | deyRcvrvg | deyCcvrvg | B1801 | 179 |
| MCC39 | TNR | p.R1007W | eefRlvdll | eefWlvdll | B1801 | 102 |
| MCC39 | TOPAZ1 | p.A1210T | feyvAtmkl | feyvTtmkl | B1801 | 55 |
| MCC39 | TP53BP1 | p.H905Y | setpfHftl | setpfYftl | B1801 | 41 |
| MCC39 | TRDMT1 | p.S322F | vqveniykS | vqveniykF | B1801 | 261 |
| MCC39 | TTC29 | p.E291K | eeyEtaltv | eeyKtaltv | B1801 | 107 |
| MCC39 | TTC40 | p.I2170F | nempptfwI | nempptfwF | B1801 | 18 |
| MCC39 | TVP23A | p.R122Q | teaeaRifw | teaeaQifw | B1801 | 85 |
| MCC39 | UNC50 | p.L54H | fefaawqmL | fefaawqmH | B1801 | 230 |
| MCC39 | USO1 | p.S67L | mehliHvlq | mehliLvlq | B1801 | 113 |
| MCC39 | USP20 | p.A307V | Aetellipd | Vetellipd | B1801 | 166 |
| MCC39 | USP28 | p.P985L | neliiPcih | neliiLcih | B1801 | 91 |
| MCC39 | UTS2B | p.K42I | neifpdKky | neifpdIky | B1801 | 21 |
| MCC39 | VPS13D | p.L1079F | qesLikley | qesFikley | B1801 | 13 |
| MCC39 | VWF | p.S958F | veSgryiil | veFgryiil | B1801 | 57 |
| MCC39 | WBSCR17 | p.G358E | ielGikvwl | ielEikvwl | B1801 | 78 |
| MCC39 | WDR96 | p.P215S | netdvvfPq | netdvvfSq | B1801 | 23 |
| MCC39 | YARS2 | p.H438Y | teggvsinH | teggvsinY | B1801 | 21 |
| MCC39 | ZNF112 | p.S93F | qevtvsyfS | qevtvsyfF | B1801 | 11 |
| MCC39 | ZNF175 | p.P155L | deqnqiqPm | deqnqiqLm | B1801 | 98 |
| MCC39 | ZNF491 | p.S114F | keceksfiS | keceksfiF | B1801 | 117 |
| MCC39 | ZNF491 | p.S114F | ceksfiSpa | ceksfiFpa | B1801 | 467 |
| MCC41 | CGN | p.E678G | veadrgrEl | veadrgrGl | C0303 | 29 |
| MCC41 | CGN | p.E678G | Eleeqnlql | Gleeqnlql | A0201 | 474 |
| MCC41 | CGN | p.E678G | veadrgrEl | veadrgrGl | C0701 | 213 |
| MCC41 | NOTCH2 | p.R1953H | aaRlavegm | aaHlavegm | C0303 | 72 |
| MCC41 | NOTCH2 | p.R1953H | lilaaRlav | lilaaHlav | A0201 | 75 |
| MCC42 | 1-Sep | p.E242K | eeEihiyqf | eeKihiyqf | B4402 | 82 |
| MCC42 | ABCA4 | p.I664V | wIysvsmtv | wVysvsmtv | A0201 | 22 |
| MCC42 | ABCA4 | p.I664V | mvlawIysv | mvlawVysv | A0201 | 3 |
| MCC42 | AHSA2 | p.M86I | yaMvalnfv | yaIvalnfv | A0201 | 50 |
| MCC42 | ARHGAP21 | p.R1296G | kmepRnlai | kmepGnlai | A0201 | 349 |
| MCC42 | ARHGAP21 | p.R1296G | kmepRnlai | kmepGnlai | C0501 | 443 |
| MCC42 | ARHGAP26 | p.S153F | lekhlnlsS | lekhlnlsF | B4402 | 211 |
| MCC42 | ASCC2 | p.P296L | Pvaedisll | Lvaedisll | A0201 | 126 |
| MCC42 | ASCC2 | p.P296L | fPvaedisl | fLvaedisl | A0201 | 13 |
| MCC42 | ATMIN | p.S173L | nSygtewdl | nLygtewdl | A0201 | 138 |
| MCC42 | B3GAT2 | p.Q272K | Qesdflkqi | Kesdflkqi | B4402 | 182 |
| MCC42 | C1orf112 | p.H219N | ilfsfHscl | ilfsfNscl | A0201 | 17 |
| MCC42 | C4orf3 | p.P173L | glPkhsywl | glLkhsywl | A0201 | 9 |
| MCC42 | CCR6 | p.L169I | iicLvvwgl | iicIvvwgl | A0201 | 237 |
| MCC42 | CNBD2 | p.E103K | rtedEiqav | rtedKiqav | C0501 | 150 |
| MCC42 | CREB3L4 | p.L282F | vlilLfsla | vlilFfsla | A0201 | 206 |
| MCC42 | CREB3L4 | p.L282F | lilLfslal | lilFfslal | A0201 | 114 |
| MCC42 | CREB3L4 | p.L282F | ilLfslali | ilFfslali | A0201 | 26 |
| MCC42 | CREB3L4 | p.L282F | cvlilLfsl | cvlilFfsl | A0201 | 458 |
| MCC42 | CXXC11 | p.S33I | vllpehSlv | vllpehIlv | A0201 | 13 |
| MCC42 | CYP4X1 | p.F18L | ylaFvfcla | ylaLvfcla | A0201 | 51 |
| MCC42 | CYP4X1 | p.F18L | Fvfclalgl | Lvfclalgl | A0201 | 257 |
| MCC42 | CYP4X1 | p.F18L | fylaFvfcl | fylaLvfcl | A0201 | 137 |
| MCC42 | DDHD1 | p.S819L | eSffnlpql | eLffnlpql | A0201 | 275 |
| MCC42 | DDHD1 | p.S819L | rlqeSffnl | rlqeLffnl | A0201 | 8 |
| MCC42 | DFNA5 | p.E164K | ellmvlEpv | ellmvlKpv | A0201 | 377 |
| MCC42 | DOT1L | p.P1037H | fsdPeseak | fsdHeseak | C0501 | 121 |
| MCC42 | EPHA8 | p.H41Y | Hgdwgwlty | Ygdwgwlty | C0501 | 78 |
| MCC42 | EPHA8 | p.H41Y | lldtstiHg | lldtstiYg | C0501 | 463 |
| MCC42 | ESRRG | p.G12E | gmlkeGvrl | gmlkeEvrl | A0201 | 276 |
| MCC42 | EYS | p.P1432L | siPgadiel | siLgadiel | A0201 | 115 |
| MCC42 | FAT4 | p.L4238F | gamLepfgv | gamFepfgv | A0201 | 132 |
| MCC42 | FRMD4A | p.L34V | llddrkleL | llddrkleV | A0201 | 19 |
| MCC42 | FRMD4A | p.L34V | leLlvqpkl | leVlvqpkl | B4402 | 465 |
| MCC42 | FRMD4A | p.L34V | llddrkleL | llddrkleV | C0501 | 14 |
| MCC42 | GIPC3 | p.E295D | gefafpdEf | gefafpdDf | B4402 | 274 |
| MCC42 | GIPC3 | p.E295D | fpdEfvvev | fpdDfvvev | A0201 | 246 |
| MCC42 | GIPC3 | p.E295D | fpdEfvvev | fpdDfvvev | B3503 | 282 |
| MCC42 | GIPC3 | p.E295D | fafpdEfvv | fafpdDfvv | A0201 | 38 |
| MCC42 | GIPC3 | p.E295D | fpdEfvvev | fpdDfvvev | C0501 | 114 |
| MCC42 | HECW1 | p.G18S | yqnrflGla | yqnrflSla | A0201 | 326 |
| MCC42 | IGSF9B | p.E866K | aemEpslks | aemKpslks | B4402 | 75 |
| MCC42 | KCNA5 | p.T479I | fwwavvtmT | fwwavvtmI | C0401 | 338 |
| MCC42 | KCNA5 | p.T479I | fwwavvtmT | fwwavvtmI | A0201 | 399 |
| MCC42 | KIAA0020 | p.E349D | miEaireav | miDaireav | A0201 | 296 |
| MCC42 | KIAA0020 | p.E349D | miEaireav | miDaireav | C0501 | 136 |
| MCC42 | KIAA1107 | p.E455K | kldhntttE | kldhntttK | C0501 | 454 |
| MCC42 | LNPEP | p.D713N | lsdkDranl | lsdkNranl | C0501 | 18 |
| MCC42 | LYVE1 | p.V74G | Vetalkasf | Getalkasf | B4402 | 39 |
| MCC42 | MELK | p.G339E | Gldkvitvl | Eldkvitvl | C0501 | 90 |
| MCC42 | MRPL11 | p.L138I | lsseeLaaf | lsseeIaaf | C0501 | 199 |
| MCC42 | MYO3B | p.G790R | flqkplGll | flqkplRll | A0201 | 96 |
| MCC42 | NBEA | p.L1750I | sLvaapvei | sIvaapvei | A0201 | 366 |
| MCC42 | NPAP1 | p.D791N | slpsahDfl | slpsahNfl | A0201 | 151 |
| MCC42 | NPHP3 | p.S865N | Sadelpwlf | Nadelpwlf | C0501 | 4 |
| MCC42 | OLFML3 | p.R124K | fpRlrdftl | fpKlrdftl | B3503 | 30 |
| MCC42 | OLFML3 | p.R124K | Rlrdftlam | Klrdftlam | A0201 | 154 |
| MCC42 | OR10J5 | p.V76L | ytlVivprm | ytlLivprm | A0201 | 330 |
| MCC42 | OR10J5 | p.V76L | setvytlVi | setvytlLi | B4402 | 49 |
| MCC42 | OR51B5 | p.V270A | kqvphiVhl | kqvphiAhl | A0201 | 224 |
| MCC42 | OR5K3 | p.D267E | gDkdipvai | gEkdipvai | B4402 | 372 |
| MCC42 | P4HA3 | p.H280N | laespnHvv | laespnNvv | C0501 | 39 |
| MCC42 | P4HA3 | p.H280N | llaespnHv | llaespnNv | A0201 | 19 |
| MCC42 | PLEKHM2 | p.D659V | vsynelDyv | vsynelVyv | A0201 | 418 |
| MCC42 | PROSER1 | p.T404I | fastsapfT | fastsapfI | C0501 | 134 |
| MCC42 | PRUNE2 | p.P755L | sPqgtnhli | sLqgtnhli | A0201 | 125 |
| MCC42 | PTCH1 | p.G141R | Gelitetgy | Relitetgy | B4402 | 369 |
| MCC42 | REV1 | p.S207F | fvdleqtSp | fvdleqtFp | C0501 | 33 |
| MCC42 | ROBO4 | p.R606C | Rlppqlaql | Clppqlaql | A0201 | 340 |
| MCC42 | SCN2A | p.A954T | tmwdcmevA | tmwdcmevT | A0201 | 99 |
| MCC42 | SKIV2L | p.P204L | tPapgllsl | tLapgllsl | A0201 | 17 |
| MCC42 | SMIM7 | p.R55Q | fllslryfR | fllslryfQ | A0201 | 230 |
| MCC42 | SMIM7 | p.R55Q | Rifialwni | Qifialwni | A0201 | 131 |
| MCC42 | SMIM7 | p.R55Q | llslryfRi | llslryfQi | A0201 | 127 |
| MCC42 | SMIM7 | p.R55Q | slryfRifi | slryfQifi | A0201 | 267 |
| MCC42 | SRL | p.L260M | kadnLatqm | kadnMatqm | C0501 | 5 |
| MCC42 | TATDN1 | p.L32V | hqddLqdvi | hqddVqdvi | C0501 | 462 |
| MCC42 | TP53 | p.K132N | alnKmfcql | alnNmfcql | A0201 | 102 |
| MCC42 | UNC5D | p.P854S | kafkiPysi | kafkiSysi | A0201 | 184 |
| MCC42 | ZNF77 | p.G390E | kafGcptyf | kafEcptyf | C0501 | 344 |
| MCC42 | ZP4 | p.P382L | sqpqwPilv | sqpqwLilv | A0201 | 37 |
| MCC44 | ABCA13 | p.D4888N | gmDpcskry | gmNpcskry | A0301 | 482 |
| MCC44 | ABCB1 | p.S696F | pvSfwrimk | pvFfwrimk | A0301 | 125 |
| MCC44 | ABCB4 | p.Q145P | laagrQirk | laagrPirk | A0301 | 201 |
| MCC44 | ABCC9 | p.G1339E | kayikpGqk | kayikpEqk | A0301 | 120 |
| MCC44 | ABCG1 | p.P116L | imgPsgagk | imgLsgagk | A0301 | 68 |
| MCC44 | ACSL5 | p.L355F | rlLnriydk | rlFnriydk | A0301 | 20 |
| MCC44 | ACSS2 | p.G118R | klGdkvafy | klRdkvafy | A0301 | 68 |
| MCC44 | ADAMTSL3 | p.R921K | rinltigsR | rinltigsK | A0301 | 40 |
| MCC44 | ADCY5 | p.H108Y | kqedmmfHk | kqedmmfYk | A0301 | 356 |
| MCC44 | ADCY5 | p.H108Y | mfHkiyiqk | mfYkiyiqk | A0301 | 60 |
| MCC44 | AHNAK | p.V4655G | kmpkVkmpk | kmpkGkmpk | A0301 | 43 |
| MCC44 | ALG3 | p.C303Y | giCfsrslh | giYfsrslh | A0301 | 126 |
| MCC44 | ALPL | p.N16S | ltNslvpek | ltSslvpek | A0301 | 363 |
| MCC44 | ANK2 | p.P1060L | lqlgpPgtk | lqlgpLgtk | A0301 | 439 |
| MCC44 | AP3D1 | p.S1022F | kgtlSfiak | kgtlFfiak | A0301 | 327 |
| MCC44 | ARFGEF2 | p.I798N | kmtkeqyIk | kmtkeqyNk | A0301 | 70 |
| MCC44 | ASB15 | p.P39S | vPlsaqnrk | vSlsaqnrk | A0301 | 322 |
| MCC44 | ASXL3 | p.S148F | vsSfqqhtk | vsFfqqhtk | A0301 | 50 |
| MCC44 | ATF6 | p.S133F | sqmsplSly | sqmsplFly | A0301 | 231 |
| MCC44 | ATP2C2 | p.E281K | mmqaeEtpk | mmqaeKtpk | A0301 | 186 |
| MCC44 | ATP2C2 | p.E281K | Etpktplqk | Ktpktplqk | A0301 | 139 |
| MCC44 | BAI3 | p.S966L | qSymavtgk | qLymavtgk | A0301 | 28 |
| MCC44 | BCMO1 | p.Y411C | rvnYahngk | rvnCahngk | A0301 | 152 |
| MCC44 | C10orf53 | p.E59K | mvnEevifh | mvnKevifh | A0301 | 331 |
| MCC44 | C11orf49 | p.G42R | Giarfftey | Riarfftey | A0301 | 103 |
| MCC44 | C16orf70 | p.P255S | lsmlgsPhk | lsmlgsShk | A0301 | 76 |
| MCC44 | C16orf70 | p.P255S | gsPhkvfyk | gsShkvfyk | A0301 | 50 |
| MCC44 | C4orf51 | p.G161E | linysrrGk | linysrrEk | A0301 | 345 |
| MCC44 | C6 | p.S584L | atykrSrtr | atykrLrtr | A0301 | 146 |
| MCC44 | CACNA1H | p.D1297A | kmfDhvvlv | kmfAhvvlv | A0301 | 259 |
| MCC44 | CAPRIN2 | p.P318S | siPvpknak | siSvpknak | A0301 | 228 |
| MCC44 | CASP8AP2 | p.D1137H | Dqldylfak | Hqldylfak | A0301 | 148 |
| MCC44 | CCDC146 | p.E244K | Eiekitrkk | Kiekitrkk | A0301 | 220 |
| MCC44 | CCDC175 | p.K259M | rmetyqkKk | rmetyqkMk | A0301 | 161 |
| MCC44 | CCR4 | p.R135K | fvmlmsidR | fvmlmsidK | A0301 | 254 |
| MCC44 | CD3D | p.G69R | ildprGiyr | ildprRiyr | A0301 | 279 |
| MCC44 | CD84 | p.S176F | lqskvlpSk | lqskvlpFk | A0301 | 109 |
| MCC44 | CDC42BPA | p.E274K | wslgvcmyE | wslgvcmyK | A0301 | 97 |
| MCC44 | CENPF | p.E2787Q | kEneraqgk | kQneraqgk | A0301 | 196 |
| MCC44 | CHMP4B | p.P195S | vPsialpsk | vSsialpsk | A0301 | 465 |
| MCC44 | CLCN1 | p.T295I | sTyfavrny | sIyfavrny | A0301 | 143 |
| MCC44 | CMYA5 | p.G1653S | rqsgsiGtk | rqsgsiStk | A0301 | 71 |
| MCC44 | CNTN5 | p.S21L | tmclseySk | tmclseyLk | A0301 | 176 |
| MCC44 | COL15A1 | p.G847S | Glpgfpglk | Slpgfpglk | A0301 | 72 |
| MCC44 | COL4A3 | p.P1163S | giPgpagek | giSgpagek | A0301 | 145 |
| MCC44 | COL6A6 | p.I722M | kgarpnIrk | kgarpnMrk | A0301 | 73 |
| MCC44 | CORO2B | p.F335I | rFyklvtlk | rIyklvtlk | A0301 | 18 |
| MCC44 | CSRNP3 | p.E201K | Elrairlsr | Klrairlsr | A0301 | 57 |
| MCC44 | CSTB | p.F43L | pvfkavsFk | pvfkavsLk | A0301 | 94 |
| MCC44 | CUX1 | p.P553S | ylslsPwdk | ylslsSwdk | A0301 | 317 |
| MCC44 | DALRD3 | p.E206K | lsvatikfE | lsvatikfK | A0301 | 490 |
| MCC44 | DEK | p.M309I | vtMkqickk | vtIkqickk | A0301 | 247 |
| MCC44 | DKK2 | p.E212K | Evctkqrkk | Kvctkqrkk | A0301 | 111 |
| MCC44 | DLG5 | p.R708Q | rRrkslggk | rQrkslggk | A0301 | 84 |
| MCC44 | DNAH10 | p.P635L | qvlPalmkk | qvlLalmkk | A0301 | 97 |
| MCC44 | DNAH14 | p.S856F | msqlySvak | msqlyFvak | A0301 | 153 |
| MCC44 | DNAH3 | p.E3758K | llsmfyckE | llsmfyckK | A0301 | 87 |
| MCC44 | DNAH6 | p.P1079L | ssrylgPlk | ssrylgLlk | A0301 | 87 |
| MCC44 | DNAH6 | p.R2691W | iisardRvk | iisardWvk | A0301 | 355 |
| MCC44 | DNAH7 | p.R3386C | rmliiRclr | rmliiCclr | A0301 | 279 |
| MCC44 | DNAH8 | p.P4016L | kPyrwildm | kLyrwildm | A0301 | 468 |
| MCC44 | DNAJB14 | p.S209F | vsnppySly | vsnppyFly | A0301 | 254 |
| MCC44 | DOCK9 | p.T1582I | vlmaTaqmk | vlmaIaqmk | A0301 | 23 |
| MCC44 | DSCAML1 | p.N1115S | evytldNlk | evytldSlk | A0301 | 229 |
| MCC44 | DTHD1 | p.E53K | ytnqvqlEk | ytnqvqlKk | A0301 | 115 |
| MCC44 | ECE2 | p.P552S | Pfyarnhpk | Sfyarnhpk | A0301 | 149 |
| MCC44 | ELAVL1 | p.F279L | virdFntnk | virdLntnk | A0301 | 182 |
| MCC44 | ENPP6 | p.D245N | mtDifwmdk | mtNifwmdk | A0301 | 66 |
| MCC44 | EPHA5 | p.R306K | qvcRpgffk | qvcKpgffk | A0301 | 84 |
| MCC44 | ETNK1 | p.R434C | ivRfnqyfk | ivCfnqyfk | A0301 | 86 |
| MCC44 | FAM186B | p.E498K | Emwqqrqkk | Kmwqqrqkk | A0301 | 44 |
| MCC44 | FAM227B | p.R351K | Raytlpisk | Kaytlpisk | A0301 | 26 |
| MCC44 | FAT3 | p.G674E | vlhGkvssk | vlhEkvssk | A0301 | 30 |
| MCC44 | FAT3 | p.E2860K | slhsdsqpE | slhsdsqpK | A0301 | 213 |
| MCC44 | FBN3 | p.R1386H | clnapggyR | clnapggyH | A0301 | 411 |
| MCC44 | FBXW7 | p.S289L | kvwSavtgk | kvwLavtgk | A0301 | 58 |
| MCC44 | FGA | p.E111K | hslttnimE | hslttnimK | A0301 | 83 |
| MCC44 | FILIP1L | p.E263K | tvmfvdErk | tvmfvdKrk | A0301 | 475 |
| MCC44 | FILIP1L | p.E263K | tltvmfvdE | tltvmfvdK | A0301 | 283 |
| MCC44 | FMN1 | p.M1114I | ttvryfgMk | ttvryfgIk | A0301 | 148 |
| MCC44 | FRAS1 | p.R2516C | gliRyvlhk | gliCyvlhk | A0301 | 44 |
| MCC44 | FRY | p.P1140S | imftPldry | imftSldry | A0301 | 430 |
| MCC44 | GALNT7 | p.P235S | svikrtPrk | svikrtSrk | A0301 | 38 |
| MCC44 | GPR110 | p.R280K | tlpcssgyR | tlpcssgyK | A0301 | 69 |
| MCC44 | GPRC5C | p.P310L | iPevsqvtk | iLevsqvtk | A0301 | 384 |
| MCC44 | GPX6 | p.F56L | iqFkqfagk | iqLkqfagk | A0301 | 135 |
| MCC44 | GRAMD3 | p.Q234E | ilhkvksQk | ilhkvksEk | A0301 | 38 |
| MCC44 | GRID1 | p.E446K | tlkvvtvlE | tlkvvtvlK | A0301 | 52 |
| MCC44 | GRIN2B | p.S1017F | tqsrSiskk | tqsrFiskk | A0301 | 89 |
| MCC44 | GRIN2B | p.S1017F | ttqsrSisk | ttqsrFisk | A0301 | 74 |
| MCC44 | GTF2F2 | p.P72L | kPasvsapr | kLasvsapr | A0301 | 78 |
| MCC44 | GUCY2C | p.E696K | rvEnsngmk | rvKnsngmk | A0301 | 68 |
| MCC44 | GUCY2C | p.L331F | ilLfghmlk | ilFfghmlk | A0301 | 12 |
| MCC44 | HCFC2 | p.P584S | Psnpvatvk | Ssnpvatvk | A0301 | 194 |
| MCC44 | HEATR3 | p.K429N | ltnylipKk | ltnylipNk | A0301 | 105 |
| MCC44 | HPSE2 | p.G168E | avngsqlGk | avngsqlEk | A0301 | 77 |
| MCC44 | HRG | p.P478L | Psfplphhk | Lsfplphhk | A0301 | 71 |
| MCC44 | HYDIN | p.E1871K | liEekilrk | liKekilrk | A0301 | 107 |
| MCC44 | HYDIN | p.R1059L | kqliyRlek | kqliyLlek | A0301 | 94 |
| MCC44 | IFI16 | p.E587K | yvcrngflE | yvcrngflK | A0301 | 262 |
| MCC44 | IFI16 | p.E587K | Evypftlva | Kvypftlva | A0301 | 498 |
| MCC44 | IL36B | p.I30L | slIaaplsr | slLaaplsr | A0301 | 262 |
| MCC44 | IMPDH2 | p.S485F | ammySgelk | ammyFgelk | A0301 | 38 |
| MCC44 | ISCA1 | p.S30L | tltpSavnk | tltpLavnk | A0301 | 125 |
| MCC44 | ITGA8 | p.Y422H | kvliYngnk | kvliHngnk | A0301 | 39 |
| MCC44 | ITPR2 | p.E607K | llEkhitak | llKkhitak | A0301 | 32 |
| MCC44 | JAKMIP2 | p.R510T | kRnqellek | kTnqellek | A0301 | 149 |
| MCC44 | KALRN | p.P37L | asdvlPilk | asdvlLilk | A0301 | 496 |
| MCC44 | KANSL3 | p.S788F | Slgatpggk | Flgatpggk | A0301 | 306 |
| MCC44 | KATNAL2 | p.P214L | sPwkgllly | sLwkgllly | A0301 | 77 |
| MCC44 | KIAA0368 | p.L212F | kfLppvllk | kfFppvllk | A0301 | 34 |
| MCC44 | KIAA0368 | p.H20Y | kisiskinH | kisiskinY | A0301 | 166 |
| MCC44 | KIAA0368 | p.H20Y | kinHssyrk | kinYssyrk | A0301 | 39 |
| MCC44 | KIF4B | p.E945Q | qmaEkqlek | qmaQkqlek | A0301 | 177 |
| MCC44 | KMT2C | p.R4584L | sRlywstry | sLlywstry | A0301 | 221 |
| MCC44 | LHCGR | p.E79K | glnevikiE | glnevikiK | A0301 | 129 |
| MCC44 | LRRC6 | p.T339A | pTyvrvmik | pAyvrvmik | A0301 | 231 |
| MCC44 | MAP4K3 | p.P258L | kmaltknPk | kmaltknLk | A0301 | 32 |
| MCC44 | MAP4K3 | p.P258L | maltknPkk | maltknLkk | A0301 | 154 |
| MCC44 | MIA2 | p.L10F | Lllaisltk | Fllaisltk | A0301 | 36 |
| MCC44 | MKI67 | p.R293T | lvsrksRpk | lvsrksTpk | A0301 | 141 |
| MCC44 | MLEC | p.G146D | rlnGhvvvk | rlnDhvvvk | A0301 | 51 |
| MCC44 | MROH9 | p.G215S | gtnkptnGk | gtnkptnSk | A0301 | 72 |
| MCC44 | MYH15 | p.N668K | aslhkenlN | aslhkenlK | A0301 | 403 |
| MCC44 | MYH15 | p.N668K | slhkenlNk | slhkenlKk | A0301 | 88 |
| MCC44 | MYH7 | p.E1772K | mmaeelkkE | mmaeelkkK | A0301 | 128 |
| MCC44 | MYO1A | p.D777N | ltlaDfiyk | ltlaNfiyk | A0301 | 30 |
| MCC44 | MYO1B | p.L189F | visnyLlek | visnyFlek | A0301 | 38 |
| MCC44 | NAALAD2 | p.S711F | kanSrlawk | kanFrlawk | A0301 | 42 |
| MCC44 | NID2 | p.D343H | siDvsfqsk | siHvsfqsk | A0301 | 84 |
| MCC44 | NID2 | p.D343H | alnghssiD | alnghssiH | A0301 | 161 |
| MCC44 | NRCAM | p.E965K | iltEytlky | iltKytlky | A0301 | 119 |
| MCC44 | NRCAM | p.E965K | giltEytlk | giltKytlk | A0301 | 63 |
| MCC44 | NRG3 | p.E325K | cvErpldlk | cvKrpldlk | A0301 | 464 |
| MCC44 | NUB1 | p.Q211H | klkekqiQr | klkekqiHr | A0301 | 302 |
| MCC44 | NXPH1 | p.R64C | llsqtfRgk | llsqtfCgk | A0301 | 139 |
| MCC44 | OBSCN | p.V3743M | kaapVewrk | kaapMewrk | A0301 | 387 |
| MCC44 | OLR1 | p.H144Y | rvancsglH | rvancsglY | A0301 | 42 |
| MCC44 | OR10K1 | p.E296K | miyslrnkE | miyslrnkK | A0301 | 22 |
| MCC44 | OR1L3 | p.G16R | rlsefillG | rlsefillR | A0301 | 63 |
| MCC44 | OR2AK2 | p.D286N | slrspsrDk | slrspsrNk | A0301 | 32 |
| MCC44 | OR4K14 | p.F77Y | wlasFatpk | wlasYatpk | A0301 | 98 |
| MCC44 | OR51A7 | p.S216F | vlSyvlilk | vlFyvlilk | A0301 | 18 |
| MCC44 | OR51E1 | p.S180F | rsnilshSy | rsnilshFy | A0301 | 218 |
| MCC44 | OR56A4 | p.G357R | kqGiqnllk | kqRiqnllk | A0301 | 93 |
| MCC44 | OR6Y1 | p.L91F | kmlvdfLsh | kmlvdfFsh | A0301 | 305 |
| MCC44 | OR7C2 | p.E173K | rlsfctnmE | rlsfctnmK | A0301 | 19 |
| MCC44 | OSBPL9 | p.R385K | ltfpngygR | ltfpngygK | A0301 | 108 |
| MCC44 | OTOGL | p.R1585K | Risglcfkk | Kisglcfkk | A0301 | 46 |
| MCC44 | OTUD4 | p.P423L | ssnPcvqrk | ssnLcvqrk | A0301 | 132 |
| MCC44 | PAPSS2 | p.M501I | rMiaganfy | rIiaganfy | A0301 | 182 |
| MCC44 | PARP14 | p.G310E | slgtalyGk | slgtalyEk | A0301 | 351 |
| MCC44 | PAXBP1 | p.P375S | ktdntvPfk | ktdntvSfk | A0301 | 44 |
| MCC44 | PCDHGA4 | p.E142K | vtnleillE | vtnleillK | A0301 | 60 |
| MCC44 | PCLO | p.G5103E | illfsnGgk | illfsnEgk | A0301 | 139 |
| MCC44 | PDE8B | p.E121K | kvllifakE | kvllifakK | A0301 | 40 |
| MCC44 | PDZRN3 | p.E566K | tilsnqhEk | tilsnqhKk | A0301 | 131 |
| MCC44 | PEX1 | p.G1063E | Gtmfrpgqk | Etmfrpgqk | A0301 | 234 |
| MCC44 | PIBF1 | p.E468Q | klksfesEr | klksfesQr | A0301 | 249 |
| MCC44 | PLCB1 | p.E475K | kssEgsgkk | kssKgsgkk | A0301 | 66 |
| MCC44 | PLTP | p.S8F | itnaSlglr | itnaFlglr | A0301 | 224 |
| MCC44 | POP1 | p.R141Q | hmrRramsh | hmrQramsh | A0301 | 91 |
| MCC44 | POSTN | p.I593M | tqgskIflk | tqgskMflk | A0301 | 268 |
| MCC44 | POSTN | p.D421N | lsmDqrllk | lsmNqrllk | A0301 | 29 |
| MCC44 | PRDM5 | p.S298L | klicSvcnk | klicLvcnk | A0301 | 45 |
| MCC44 | PRKG2 | p.R38C | ivklyRtfk | ivklyCtfk | A0301 | 82 |
| MCC44 | PSD2 | p.T581I | aTrasdysk | aIrasdysk | A0301 | 246 |
| MCC44 | PSMA8 | p.E197Q | elyvteiEk | elyvteiQk | A0301 | 156 |
| MCC44 | PTPRN2 | p.E620K | hssqhrlkE | hssqhrlkK | A0301 | 62 |
| MCC44 | PYHIN1 | p.S441F | ssSftkvtk | ssFftkvtk | A0301 | 68 |
| MCC44 | R3HDM2 | p.E24Q | klveEsvnk | klveQsvnk | A0301 | 54 |
| MCC44 | RAD17 | p.L6I | msktfLrpk | msktfIrpk | A0301 | 134 |
| MCC44 | RALGAPB | p.P1009S | vPkndvgfk | vSkndvgfk | A0301 | 228 |
| MCC44 | RGS18 | p.E15K | Eskektffk | Kskektffk | A0301 | 59 |
| MCC44 | RICTOR | p.T1091K | rlTpsrfmk | rlKpsrfmk | A0301 | 44 |
| MCC44 | RNF148 | p.E162K | misnlkgmE | misnlkgmK | A0301 | 72 |
| MCC44 | RNF43 | p.L639V | tssLfnlqk | tssVfnlqk | A0301 | 184 |
| MCC44 | RYR2 | p.L274I | svharsLwr | svharsIwr | A0301 | 326 |
| MCC44 | SAMD4A | p.R145W | klfRsfprk | klfWsfprk | A0301 | 13 |
| MCC44 | SEC14L5 | p.P8L | yqsPvrvyk | yqsLvrvyk | A0301 | 445 |
| MCC44 | SECISBP2 | p.L604F | vlgLrevlk | vlgFrevlk | A0301 | 56 |
| MCC44 | SEMA3A | p.D87N | lvnikDfqk | lvnikNfqk | A0301 | 244 |
| MCC44 | SEMA3E | p.D259N | fllptrDhk | fllptrNhk | A0301 | 246 |
| MCC44 | SFTPA2 | p.N234K | emytdgqwN | emytdgqwK | A0301 | 360 |
| MCC44 | SHANK2 | p.T352I | Tvatmmrek | Ivatmmrek | A0301 | 97 |
| MCC44 | SIX4 | p.E225K | Etvycfkek | Ktvycfkek | A0301 | 187 |
| MCC44 | SLC1A6 | p.M385K | mlqalitaM | mlqalitaK | A0301 | 42 |
| MCC44 | SLC7A13 | p.F216L | aifqgyFay | aifqgyLay | A0301 | 199 |
| MCC44 | SLTM | p.R564K | kihgRskek | kihgKskek | A0301 | 211 |
| MCC44 | SMG1 | p.E2286K | hvmkavleE | hvmkavleK | A0301 | 41 |
| MCC44 | SORBS2 | p.L34F | ssLaakgfr | ssFaakgfr | A0301 | 300 |
| MCC44 | SORBS2 | p.L34F | atyssLaak | atyssFaak | A0301 | 23 |
| MCC44 | SPHK2 | p.G283R | alhspvseG | alhspvseR | A0301 | 244 |
| MCC44 | SPINK5 | p.S421L | kSrnkrqsk | kLrnkrqsk | A0301 | 33 |
| MCC44 | SPTA1 | p.E364K | alatsryEk | alatsryKk | A0301 | 35 |
| MCC44 | SPTA1 | p.E364K | ralatsryE | ralatsryK | A0301 | 83 |
| MCC44 | SRGAP1 | p.S230L | rqakySenk | rqakyLenk | A0301 | 164 |
| MCC44 | STAT3 | p.E357K | Elnyqlkik | Klnyqlkik | A0301 | 57 |
| MCC44 | TC2N | p.S278F | pvhfkSsak | pvhfkFsak | A0301 | 437 |
| MCC44 | TDRD1 | p.M627I | lMkklvqnk | lIkklvqnk | A0301 | 252 |
| MCC44 | TLE6 | p.E95K | qlqgfqseE | qlqgfqseK | A0301 | 230 |
| MCC44 | TMEM108 | p.E563K | vlvnpfcqE | vlvnpfcqK | A0301 | 48 |
| MCC44 | TMPRSS15 | p.G992R | flagvtsfG | flagvtsfR | A0301 | 136 |
| MCC44 | TPH2 | p.H234Y | klyptHacr | klyptYacr | A0301 | 48 |
| MCC44 | TRIM44 | p.E236K | Elverlkfk | Klverlkfk | A0301 | 33 |
| MCC44 | TRIM44 | p.E236K | miElverlk | miKlverlk | A0301 | 174 |
| MCC44 | TRIP4 | p.R466Q | Rlwiaatak | Qlwiaatak | A0301 | 132 |
| MCC44 | TSHZ3 | p.S1061R | havklhlSk | havklhlRk | A0301 | 456 |
| MCC44 | TTC3 | p.E1090K | alydqhsnE | alydqhsnK | A0301 | 18 |
| MCC44 | TTF2 | p.F978L | vslngtFfk | vslngtLfk | A0301 | 23 |
| MCC44 | TTK | p.Q331E | ksvQnshfk | ksvEnshfk | A0301 | 117 |
| MCC44 | TTN | p.E23769K | ilErrevpk | ilKrrevpk | A0301 | 69 |
| MCC44 | TTN | p.A10001V | Amtlgvsyk | Vmtlgvsyk | A0301 | 22 |
| MCC44 | TTN | p.A10001V | kAmtlgvsy | kVmtlgvsy | A0301 | 128 |
| MCC44 | TTN | p.E3724K | ilkaalitE | ilkaalitK | A0301 | 38 |
| MCC44 | TTN | p.S3409L | vslsvSfkk | vslsvLfkk | A0301 | 45 |
| MCC44 | TTN | p.S3409L | hvslsvSfk | hvslsvLfk | A0301 | 25 |
| MCC44 | UCHL1 | p.S215F | rfSavalck | rfFavalck | A0301 | 50 |
| MCC44 | UHMK1 | p.D321H | ltgrmfDgk | ltgrmfHgk | A0301 | 211 |
| MCC44 | UHMK1 | p.D321H | klltgrmfD | klltgrmfH | A0301 | 33 |
| MCC44 | ULK4 | p.P367L | llssrptPr | llssrptLr | A0301 | 256 |
| MCC44 | UNC13A | p.L1405W | tmignlLrk | tmignlWrk | A0301 | 101 |
| MCC44 | UNC79 | p.S2294F | vlhmcSlfh | vlhmcFlfh | A0301 | 108 |
| MCC44 | USP8 | p.L1007F | klppvLlvh | klppvFlvh | A0301 | 261 |
| MCC44 | UTRN | p.S2823L | iShnkvpyy | iLhnkvpyy | A0301 | 116 |
| MCC44 | VIT | p.N498S | lqfvtNltk | lqfvtSltk | A0301 | 76 |
| MCC44 | VMP1 | p.L111I | riekqfLly | riekqfIly | A0301 | 329 |
| MCC44 | WDR17 | p.H547Y | htakvfHvk | htakvfYvk | A0301 | 46 |
| MCC44 | XRCC4 | p.S110F | rlgSfnlek | rlgFfnlek | A0301 | 32 |
| MCC44 | ZC3H4 | p.E401K | kvickyfvE | kvickyfvK | A0301 | 82 |
| MCC44 | ZCCHC6 | p.S1048F | klslfgsSk | klslfgsFk | A0301 | 14 |
| MCC44 | ZCCHC7 | p.A237V | tqryysAnk | tqryysVnk | A0301 | 194 |
| MCC44 | ZFP90 | p.R629K | hqRihtrnk | hqKihtrnk | A0301 | 343 |
| MCC44 | ZNF214 | p.R574K | ssalrihqR | ssalrihqK | A0301 | 116 |
| MCC44 | ZNF226 | p.S544L | sSylqihqk | sLylqihqk | A0301 | 22 |
| MCC44 | ZNF230 | p.H386Y | rvHtgerpy | rvYtgerpy | A0301 | 153 |
| MCC44 | ZNF236 | p.H1189Y | hvriHtgek | hvriYtgek | A0301 | 105 |
| MCC44 | ZNF236 | p.H1189Y | riHtgekpy | riYtgekpy | A0301 | 224 |
| MCC44 | ZNF334 | p.H286Y | riHtgerpy | riYtgerpy | A0301 | 138 |
| MCC44 | ZNF347 | p.H535Y | riHtgvkpy | riYtgvkpy | A0301 | 62 |
| MCC44 | ZNF347 | p.H535Y | hqriHtgvk | hqriYtgvk | A0301 | 201 |
| MCC44 | ZNF347 | p.H535Y | hlanhqriH | hlanhqriY | A0301 | 236 |
| MCC44 | ZNF486 | p.K111Q | kvilrKfek | kvilrQfek | A0301 | 57 |
| MCC44 | ZNF568 | p.E16K | isnscvtmE | isnscvtmK | A0301 | 51 |
| MCC44 | ZNF608 | p.P1350T | hayPypqmy | hayTypqmy | A0301 | 404 |
| MCC44 | ZNF790 | p.N609K | Niytfeksy | Kiytfeksy | A0301 | 152 |
| MCC44 | ZNF790 | p.N609K | hqNiytfek | hqKiytfek | A0301 | 432 |
| MCC44 | ZNF804B | p.L166F | cmksaLllk | cmksaFllk | A0301 | 25 |
| MCC44 | ZNF804B | p.L166F | ksaLllkgk | ksaFllkgk | A0301 | 93 |
| MCC44 | ZNF93 | p.L580H | kafnhsatL | kafnhsatH | A0301 | 246 |
| MCC44 | ZNF93 | p.L580H | hsatLsshk | hsatHsshk | A0301 | 28 |
| MCC47 | AQP4 | p.L132F | Ltaghgllv | Ftaghgllv | A0101 | 148 |
| MCC48 | ABCA8 | p.F970L | irterstFl | irterstLl | C0701 | 8 |
| MCC48 | ABCA8 | p.F970L | hirterstF | hirterstL | B0801 | 224 |
| MCC48 | ADAM32 | p.E309K | ypkEitlea | ypkKitlea | B0801 | 155 |
| MCC48 | AGGF1 | p.P449S | ehtlriPev | ehtlriSev | C0701 | 63 |
| MCC48 | AGPAT6 | p.D311Y | ehvqDkskl | ehvqYkskl | C0701 | 81 |
| MCC48 | ANLN | p.K69E | Krcsdntev | Ercsdntev | C0701 | 108 |
| MCC48 | ARHGAP29 | p.P1198L | dhdPhglvv | dhdLhglvv | C0701 | 225 |
| MCC48 | ASXL3 | p.L258F | Lvntnlral | Fvntnlral | C0701 | 339 |
| MCC48 | BRINP3 | p.M332I | klfmkrlpM | klfmkrlpI | B0801 | 28 |
| MCC48 | C10orf67 | p.S166F | mrkSfnqql | mrkFfnqql | C0701 | 89 |
| MCC48 | C10orf67 | p.S166F | mrkSfnqql | mrkFfnqql | B0801 | 346 |
| MCC48 | CCDC130 | p.E49K | irfEmpyni | irfKmpyni | C0701 | 50 |
| MCC48 | CD248 | p.P483S | srdhqiPvi | srdhqiSvi | C0701 | 33 |
| MCC48 | CEACAM5 | p.P334T | itsnnsnPv | itsnnsnTv | C0701 | 284 |
| MCC48 | CENPA | p.D108N | feDaylltl | feNaylltl | C0701 | 213 |
| MCC48 | CENPA | p.D108N | hlfeDayll | hlfeNayll | C0701 | 230 |
| MCC48 | CHPT1 | p.W95R | eeapyWtyl | eeapyRtyl | C0701 | 489 |
| MCC48 | COL11A1 | p.G578R | qGnpgpqgl | qRnpgpqgl | C0701 | 37 |
| MCC48 | COQ10B | p.R81K | yseRrilgy | yseKrilgy | A0101 | 10 |
| MCC48 | CPT1A | p.G134R | llsyhGwmf | llsyhRwmf | B0801 | 83 |
| MCC48 | CSMD3 | p.D3081N | tnfDwgfsi | tnfNwgfsi | C0701 | 483 |
| MCC48 | CSTL1 | p.G77E | tGveyivtv | tEveyivtv | C0701 | 405 |
| MCC48 | CTNND2 | p.G837R | eppkGiqml | eppkRiqml | B0801 | 156 |
| MCC48 | DAP3 | p.G292R | lgkeGfdal | lgkeRfdal | B0801 | 25 |
| MCC48 | EIF3H | p.E102D | yqmEmmrsl | yqmDmmrsl | C0701 | 53 |
| MCC48 | ENAM | p.E968K | lgqkEimpf | lgqkKimpf | B0801 | 299 |
| MCC48 | EPHA7 | p.G753E | mryladmGy | mryladmEy | C0701 | 336 |
| MCC48 | ERCC1 | p.T48I | frstqslpT | frstqslpI | C0701 | 82 |
| MCC48 | FAM194B | p.E136K | ehleEeeyl | ehleKeeyl | C0701 | 25 |
| MCC48 | FBXO39 | p.E391K | sqkErqcal | sqkKrqcal | B0801 | 16 |
| MCC48 | FPR2 | p.P239L | srPlrvlta | srLlrvlta | B0801 | 327 |
| MCC48 | GCC1 | p.E756K | fspeEkqvi | fspeKkqvi | C0701 | 172 |
| MCC48 | GNLY | p.D105N | wrDvcrnfm | wrNvcrnfm | C0701 | 135 |
| MCC48 | GPR107 | p.G451E | raGsriaai | raEsriaai | B0801 | 335 |
| MCC48 | GPR116 | p.G286R | feGdtvslv | feRdtvslv | C0701 | 93 |
| MCC48 | IMMP2L | p.G135R | snsfGpvsl | snsfRpvsl | B0801 | 421 |
| MCC48 | IMMP2L | p.G135R | fGpvslgll | fRpvslgll | C0701 | 189 |
| MCC48 | KIF21A | p.E179Q | ihEdstggi | ihQdstggi | C0701 | 65 |
| MCC48 | KIF21A | p.E179Q | hEdstggiy | hQdstggiy | A0101 | 213 |
| MCC48 | LCN6 | p.A20V | vsvpraqAv | vsvpraqVv | C0701 | 323 |
| MCC48 | LIG4 | p.R580Q | lrfpRieki | lrfpQieki | C0701 | 12 |
| MCC48 | LRP1B | p.D301N | Dhvgdrifv | Nhvgdrifv | C0701 | 378 |
| MCC48 | LRRC32 | p.D79N | ytalrhlDl | ytalrhlNl | B0801 | 155 |
| MCC48 | LRRC4C | p.P624S | ihssvhePl | ihssvheSl | C0701 | 34 |
| MCC48 | LRRC4C | p.P624S | vhePllirm | vheSllirm | C0701 | 165 |
| MCC48 | LYSMD4 | p.P66L | wrcsmaakP | wrcsmaakL | C0701 | 298 |
| MCC48 | MKL2 | p.S904F | Srpppqvqm | Frpppqvqm | C0701 | 113 |
| MCC48 | MORC1 | p.E416K | hnkqEflnv | hnkqKflnv | B0801 | 109 |
| MCC48 | MS4A7 | p.M108I | seMdylssl | seIdylssl | C0701 | 234 |
| MCC48 | NARF | p.P306S | fPfhfvevl | fSfhfvevl | C0701 | 225 |
| MCC48 | OR2D2 | p.T23I | phTeqllfi | phIeqllfi | C0701 | 107 |
| MCC48 | OR4X2 | p.S49F | Spmyfflsy | Fpmyfflsy | B0801 | 134 |
| MCC48 | OTOF | p.E413Q | Erqwarfyv | Qrqwarfyv | C0701 | 378 |
| MCC48 | OVCH1 | p.P215L | vlksmnlpP | vlksmnlpL | B0801 | 91 |
| MCC48 | PDE11A | p.T341A | lyferrTef | lyferrAef | C0701 | 235 |
| MCC48 | PDE11A | p.T341A | rrTeffelv | rrAeffelv | C0701 | 122 |
| MCC48 | PECR | p.L267F | dvdggrsLy | dvdggrsFy | A0101 | 218 |
| MCC48 | PECR | p.L267F | sLythsyev | sFythsyev | C0701 | 294 |
| MCC48 | PKNOX1 | p.P168L | wlfqhighP | wlfqhighL | B0801 | 203 |
| MCC48 | PLAGL1 | p.R30P | yshsreRpy | yshsrePpy | A0101 | 392 |
| MCC48 | PLXNA4 | p.G421R | sdmvrGipv | sdmvrRipv | B0801 | 258 |
| MCC48 | PROC | p.S28F | Ssserahqv | Fsserahqv | C0701 | 50 |
| MCC48 | RALGPS2 | p.S26F | Slsdkgsel | Flsdkgsel | C0701 | 101 |
| MCC48 | RASGRP3 | p.I420M | hIrklvesv | hMrklvesv | B0801 | 444 |
| MCC48 | RTKN2 | p.D542N | slDtklstl | slNtklstl | B0801 | 45 |
| MCC48 | SEC11C | p.S42F | famivSsal | famivFsal | B0801 | 16 |
| MCC48 | SLC24A1 | p.S84F | Semggkmlv | Femggkmlv | C0701 | 196 |
| MCC48 | SYT16 | p.R23W | fsswisRvy | fsswisWvy | A0101 | 10 |
| MCC48 | TAS2R7 | p.R214Q | rrhirRmql | rrhirQmql | B0801 | 181 |
| MCC48 | TAS2R7 | p.R214Q | slrrhirRm | slrrhirQm | C0701 | 482 |
| MCC48 | TAS2R7 | p.R214Q | hirRmqlsa | hirQmqlsa | B0801 | 454 |
| MCC48 | TAS2R7 | p.R214Q | rrhirRmql | rrhirQmql | C0701 | 65 |
| MCC48 | TAS2R7 | p.R214Q | slrrhirRm | slrrhirQm | B0801 | 14 |
| MCC48 | TIAM2 | p.R379K | slkaRmrri | slkaKmrri | B0801 | 79 |
| MCC48 | TTC32 | p.L132I | tlsLkqtil | tlsIkqtil | B0801 | 428 |
| MCC48 | UBQLN1 | p.D98N | ihDgltvhl | ihNgltvhl | C0701 | 31 |
| MCC48 | VWA8 | p.R1655Q | rrqvhslRi | rrqvhslQi | C0701 | 62 |
| MCC48 | WDR87 | p.D1245N | dDlcrlmal | dNlcrlmal | B0801 | 72 |
| MCC48 | WDR87 | p.D1245N | wrdDlcrlm | wrdNlcrlm | C0701 | 202 |
| MCC48 | ZBP1 | p.M52I | yrMkkelkv | yrIkkelkv | C0701 | 41 |
| MCC48 | ZBTB37 | p.R238K | grsddevRv | grsddevKv | C0701 | 229 |
| MCC48 | ZMYM4 | p.E1363A | ymfsrieeE | ymfsrieeA | C0701 | 313 |
| MCC48 | ZNF112 | p.H175Y | ahHswrkmy | ahYswrkmy | C0701 | 34 |
| MCC48 | ZNF112 | p.H175Y | hHswrkmyl | hYswrkmyl | C0701 | 353 |
| MCC48 | ZNF274 | p.S423F | svhkkIhtg | svhkkFhtg | B0801 | 168 |
| MCC48 | ZNF274 | p.S423F | Ihtgerpyv | Fhtgerpyv | C0701 | 13 |
| MCC48 | ZSCAN9 | p.P337L | qrihkgekP | qrihkgekL | C0701 | 114 |
| MCC53 | C10orf25 | p.L77I | rshLnyhqk | rshInyhqk | A1101 | 29 |
| MCC53 | C10orf25 | p.L77I | shLnyhqka | shInyhqka | C0701 | 133 |
| MCC53 | CR2 | p.V605L | hVhiangyk | hLhiangyk | A1101 | 496 |
| MCC53 | CR2 | p.V605L | Vhiangyki | Lhiangyki | C0701 | 83 |
| MCC53 | KCNA1 | p.V486I | tanqncVnk | tanqncInk | A1101 | 55 |
| MCC53 | OR10G9 | p.S118P | tvmSydryl | tvmPydryl | C0701 | 84 |
| MCC53 | OR10G9 | p.S118P | mSydrylai | mPydrylai | B5301 | 6 |
| MCC53 | OR5AN1 | p.S218F | lvimiSygy | lvimiFygy | A1101 | 94 |
| MCC53 | OR5AN1 | p.S218F | salvimiSy | salvimiFy | A1101 | 38 |
| MCC54 | ABCC12 | p.M1240I | fmrdtiMkl | fmrdtiIkl | C0702 | 160 |
| MCC54 | ABCC12 | p.M1240I | fmrdtiMkl | fmrdtiIkl | C0701 | 455 |
| MCC54 | ABCC3 | p.E1267K | rvkeysktE | rvkeysktK | A0301 | 187 |
| MCC54 | ACACA | p.G1862S | aGrphptqk | aSrphptqk | A0301 | 131 |
| MCC54 | ACSS2 | p.P148S | Pvrccivvk | Svrccivvk | A0301 | 90 |
| MCC54 | ADAM18 | p.E121K | glrgflqfE | glrgflqfK | A0301 | 27 |
| MCC54 | ADAM2 | p.I331M | ihfsgvkIf | ihfsgvkMf | C0701 | 49 |
| MCC54 | ADAM2 | p.I331M | ihfsgvkIf | ihfsgvkMf | C0702 | 494 |
| MCC54 | ADAR | p.S316F | satsffSgk | satsffFgk | A0301 | 247 |
| MCC54 | ADCY10 | p.E1429K | ilslpswEk | ilslpswKk | A0301 | 35 |
| MCC54 | ADCY10 | p.E1429K | tilslpswE | tilslpswK | A0301 | 64 |
| MCC54 | ADCY10 | p.E1429K | lpswEkiva | lpswKkiva | B0702 | 206 |
| MCC54 | AEBP1 | p.G591S | Gkssrglki | Skssrglki | C0701 | 271 |
| MCC54 | AEBP1 | p.G591S | lGkssrglk | lSkssrglk | A0301 | 145 |
| MCC54 | AEBP1 | p.G591S | itrtyslGk | itrtyslSk | A0301 | 32 |
| MCC54 | AK5 | p.P501S | asiPviayy | asiSviayy | A2601 | 97 |
| MCC54 | AK5 | p.P501S | yyrasiPvi | yyrasiSvi | C0701 | 159 |
| MCC54 | AK5 | p.P501S | yyrasiPvi | yyrasiSvi | C0702 | 105 |
| MCC54 | AKAP12 | p.K1358I | vleeKssek | vleeIssek | A0301 | 448 |
| MCC54 | ATAD2 | p.E440K | Emvvfplly | Kmvvfplly | A0301 | 50 |
| MCC54 | ATAD2 | p.E440K | nhiaalkEm | nhiaalkKm | C0701 | 225 |
| MCC54 | BAZ2A | p.P582S | yygPcgkrm | yygScgkrm | C0702 | 95 |
| MCC54 | BAZ2A | p.P582S | yygPcgkrm | yygScgkrm | C0701 | 271 |
| MCC54 | BBS7 | p.V314A | tysgwVtgl | tysgwAtgl | C0702 | 291 |
| MCC54 | BBS7 | p.V314A | tysgwVtgl | tysgwAtgl | C0701 | 244 |
| MCC54 | BRD4 | p.G691S | dviaGsskm | dviaSsskm | A2601 | 10 |
| MCC54 | BRD4 | p.G691S | viaGsskmk | viaSsskmk | A0301 | 75 |
| MCC54 | C12orf43 | p.S11L | apsgtvsdS | apsgtvsdL | B0702 | 19 |
| MCC54 | C17orf47 | p.G186R | Gvrvprril | Rvrvprril | B0702 | 8 |
| MCC54 | C2CD3 | p.H375Y | srnrfkdHi | srnrfkdYi | C0701 | 145 |
| MCC54 | CCDC125 | p.S297F | aaStrklll | aaFtrklll | C0701 | 198 |
| MCC54 | CD163 | p.P95L | qlgcPtaik | qlgcLtaik | A0301 | 482 |
| MCC54 | CDC42BPB | p.P356L | irnleaPyi | irnleaLyi | C0701 | 182 |
| MCC54 | CDH12 | p.S746F | Svaeslssi | Fvaeslssi | A2601 | 122 |
| MCC54 | CEACAM5 | p.S96N | gpaySgrei | gpayNgrei | B0702 | 28 |
| MCC54 | CELSR3 | p.V1913A | apqglVgci | apqglAgci | B0702 | 236 |
| MCC54 | CFTR | p.E528K | vikacqleE | vikacqleK | A0301 | 61 |
| MCC54 | CHRM3 | p.P421S | gsfPksfsk | gsfSksfsk | A0301 | 31 |
| MCC54 | CLDN8 | p.M102I | fmmailgMk | fmmailgIk | A0301 | 36 |
| MCC54 | CLK2 | p.D218H | kDpdnknlc | kHpdnknlc | C0701 | 239 |
| MCC54 | CPXM1 | p.P621L | gpfPcnfvl | gpfLcnfvl | B0702 | 337 |
| MCC54 | CPXM1 | p.P621L | fPcnfvltk | fLcnfvltk | A0301 | 59 |
| MCC54 | CR1 | p.K1937R | lKcedgytl | lRcedgytl | C0702 | 250 |
| MCC54 | CR1 | p.K1937R | lKcedgytl | lRcedgytl | C0701 | 75 |
| MCC54 | CRTC1 | p.S226F | vpginifpS | vpginifpF | B0702 | 263 |
| MCC54 | CTNND2 | p.N714K | vlrNatgcl | vlrKatgcl | B0702 | 423 |
| MCC54 | CYP4F8 | p.S414F | vvlpdsrvI | vvlpdsrvF | C0702 | 211 |
| MCC54 | DBF4 | p.L170F | Lhiddiryy | Fhiddiryy | C0701 | 7 |
| MCC54 | DBF4 | p.L170F | Lhiddiryy | Fhiddiryy | C0702 | 277 |
| MCC54 | DBNL | p.I170V | sIsspqpgk | sVsspqpgk | A0301 | 66 |
| MCC54 | DCAF6 | p.P80L | visnPysrk | visnLysrk | A0301 | 48 |
| MCC54 | DCC | p.P748S | iimswtpPl | iimswtpSl | B0702 | 351 |
| MCC54 | DCPS | p.P39S | Pvrlpfsgf | Svrlpfsgf | B0702 | 193 |
| MCC54 | DDB1 | p.H522Y | ylqiHpqel | ylqiYpqel | C0701 | 428 |
| MCC54 | DDB1 | p.H522Y | ylqiHpqel | ylqiYpqel | C0702 | 434 |
| MCC54 | DEPDC4 | p.D44V | gpssrnrrD | gpssrnrrV | B0702 | 237 |
| MCC54 | DHX32 | p.E593K | iraellEii | iraellKii | C0701 | 121 |
| MCC54 | DMGDH | p.R510C | rpsfRrtnw | rpsfCrtnw | B0702 | 30 |
| MCC54 | DMGDH | p.R510C | tqyrpsfRr | tqyrpsfCr | A0301 | 194 |
| MCC54 | DNAH14 | p.L2055R | kswLlktsk | kswRlktsk | A0301 | 73 |
| MCC54 | DNAH2 | p.G2419R | lvgpvGtgk | lvgpvRtgk | A0301 | 286 |
| MCC54 | DNAH5 | p.E3889K | lyEehkflf | lyKehkflf | C0702 | 65 |
| MCC54 | DNAH5 | p.E3889K | kyaarglyE | kyaarglyK | A0301 | 95 |
| MCC54 | DNAH7 | p.L3126F | Leeekqali | Feeekqali | C0701 | 486 |
| MCC54 | DNAH8 | p.E1514K | mssisgyyE | mssisgyyK | A0301 | 81 |
| MCC54 | DNMT1 | p.S977F | ypehyrkyS | ypehyrkyF | B0702 | 151 |
| MCC54 | DNMT1 | p.S977F | hyrkySdyi | hyrkyFdyi | C0702 | 326 |
| MCC54 | DNMT3B | p.P266L | kPtgieglk | kLtgieglk | A0301 | 47 |
| MCC54 | DPP4 | p.P264L | ypkagavnP | ypkagavnL | B0702 | 44 |
| MCC54 | DYNC2H1 | p.N2009K | kvvkqytmN | kvvkqytmK | A0301 | 74 |
| MCC54 | DYRK1A | p.P554S | vrqqfPapl | vrqqfSapl | C0702 | 330 |
| MCC54 | ECT2 | p.L280F | thLvveeni | thFvveeni | C0701 | 196 |
| MCC54 | ERF | p.M76I | kpqMnydkl | kpqInydkl | B0702 | 276 |
| MCC54 | EVI5 | p.V241A | slvdreVgy | slvdreAgy | A2601 | 205 |
| MCC54 | EVX2 | p.P57L | sprlpsaPl | sprlpsaLl | B0702 | 9 |
| MCC54 | EVX2 | p.P57L | lpsaPlhsa | lpsaLlhsa | B0702 | 240 |
| MCC54 | F11 | p.D182N | tritklDkv | tritklNkv | C0701 | 105 |
| MCC54 | FAM135B | p.L1271P | glwlmqkLk | glwlmqkPk | A0301 | 186 |
| MCC54 | FAM135B | p.L1271P | kLkksgsll | kPkksgsll | B0702 | 11 |
| MCC54 | FAM83B | p.E938K | rvysrfEpf | rvysrfKpf | B0702 | 190 |
| MCC54 | FAM83B | p.E938K | ysrfEpfck | ysrfKpfck | A0301 | 289 |
| MCC54 | FAM83B | p.E938K | rvysrfEpf | rvysrfKpf | C0702 | 321 |
| MCC54 | FANCL | p.R349K | wlrglltsR | wlrglltsK | A0301 | 190 |
| MCC54 | FANCM | p.S933F | ftnkStssl | ftnkFtssl | B0702 | 476 |
| MCC54 | FGFR4 | p.E27K | vlsleaseE | vlsleaseK | A0301 | 153 |
| MCC54 | FNBP1 | p.H382Y | kiHcfrslk | kiYcfrslk | A0301 | 13 |
| MCC54 | GAB2 | p.S434F | nSqsvyipm | nFqsvyipm | C0702 | 167 |
| MCC54 | GPR98 | p.G72R | vtaivslyG | vtaivslyR | A0301 | 234 |
| MCC54 | GPR98 | p.G72R | yGedagdff | yRedagdff | C0702 | 283 |
| MCC54 | GPR98 | p.G72R | lyGedagdf | lyRedagdf | C0702 | 145 |
| MCC54 | GPR98 | p.G72R | yGedagdff | yRedagdff | C0701 | 60 |
| MCC54 | GZMA | p.P104S | fpyPcydpa | fpyScydpa | B0702 | 304 |
| MCC54 | HCFC2 | p.G25E | arhGhrava | arhEhrava | C0701 | 215 |
| MCC54 | HCFC2 | p.G25E | rhGhravai | rhEhravai | C0701 | 97 |
| MCC54 | HCFC2 | p.G25E | rarhGhrav | rarhEhrav | B0702 | 44 |
| MCC54 | HDAC4 | p.P344L | gsaaplPly | gsaaplLly | A0301 | 213 |
| MCC54 | HECTD4 | p.P1796L | ktsPknslk | ktsLknslk | A0301 | 41 |
| MCC54 | HECTD4 | p.P1796L | ttltktsPk | ttltktsLk | A0301 | 33 |
| MCC54 | HERC1 | p.P4362L | hsaawtapP | hsaawtapL | B0702 | 135 |
| MCC54 | HHIPL2 | p.R387K | kvlridvnR | kvlridvnK | A0301 | 92 |
| MCC54 | HIBCH | p.S251F | Skidrdksf | Fkidrdksf | C0702 | 406 |
| MCC54 | HIVEP3 | p.G41S | ypgsGtaat | ypgsStaat | B0702 | 18 |
| MCC54 | HIVEP3 | p.G41S | vpypgsGta | vpypgsSta | B0702 | 49 |
| MCC54 | HMCN1 | p.L1564F | svlinsLik | svlinsFik | A0301 | 122 |
| MCC54 | HPS5 | p.L619H | gLrndlael | gHrndlael | C0701 | 138 |
| MCC54 | HPS5 | p.L619H | Lrndlaelt | Hrndlaelt | C0701 | 398 |
| MCC54 | HYI | p.P113L | ihlmagrvP | ihlmagrvL | C0701 | 46 |
| MCC54 | ITGA8 | p.E912K | Efhrqspak | Kfhrqspak | A0301 | 450 |
| MCC54 | ITGB8 | p.H349Y | Hwykdllpl | Ywykdllpl | C0702 | 61 |
| MCC54 | ITGB8 | p.H349Y | favqgkqfH | favqgkqfY | A2601 | 326 |
| MCC54 | KANSL1 | p.S531L | hiSeslstk | hiLeslstk | A0301 | 35 |
| MCC54 | KDM8 | p.G15R | Grgseasgl | Rrgseasgl | C0701 | 494 |
| MCC54 | KIAA0753 | p.S211N | niSeqksll | niNeqksll | C0701 | 340 |
| MCC54 | KIF16B | p.T169I | ksskTfnlr | ksskIfnlr | A0301 | 463 |
| MCC54 | KIF1A | p.P305L | Pyrdsvltw | Lyrdsvltw | C0702 | 382 |
| MCC54 | KPNA7 | p.S409F | vhSgvlepl | vhFgvlepl | C0701 | 191 |
| MCC54 | L3MBTL4 | p.G111S | svaevcGyr | svaevcSyr | A0301 | 290 |
| MCC54 | LAMA2 | p.P1232L | mredlhleP | mredlhleL | C0702 | 303 |
| MCC54 | LAMA2 | p.P1232L | mredlhleP | mredlhleL | C0701 | 13 |
| MCC54 | LARS | p.E1022K | vlmEnivyl | vlmKnivyl | C0701 | 380 |
| MCC54 | LEPREL2 | p.R386K | filrslgEk | filrslgKk | A0301 | 127 |
| MCC54 | LEPREL2 | p.R386K | rfilrslgE | rfilrslgK | A0301 | 445 |
| MCC54 | LGALS12 | p.P208S | lisaPflfy | lisaSflfy | A0301 | 45 |
| MCC54 | LGALS12 | p.P208S | Pflfypqrf | Sflfypqrf | C0702 | 243 |
| MCC54 | LGSN | p.G201E | lsssrslGk | lsssrslEk | A0301 | 106 |
| MCC54 | LPPR5 | p.R188G | raRktfpsk | raGktfpsk | A0301 | 212 |
| MCC54 | LRP1B | p.G2143R | rvrekGtnv | rvrekRtnv | B0702 | 49 |
| MCC54 | LRP1B | p.G2143R | vrekGtnvc | vrekRtnvc | C0701 | 369 |
| MCC54 | LRRC66 | p.H872Y | fHerdsdil | fYerdsdil | C0702 | 144 |
| MCC54 | LYSMD1 | p.R167W | rtsrtRtlr | rtsrtWtlr | A0301 | 455 |
| MCC54 | LYSMD1 | p.R167W | trtsrtRtl | trtsrtWtl | C0701 | 9 |
| MCC54 | LYSMD1 | p.R167W | trtsrtRtl | trtsrtWtl | C0702 | 101 |
| MCC54 | MAP3K13 | p.P20L | ergvctqaP | ergvctqaL | C0701 | 200 |
| MCC54 | MAP4 | p.P895L | Psrpsttpf | Lsrpsttpf | B0702 | 446 |
| MCC54 | MAP4 | p.P895L | srvkatpmP | srvkatpmL | C0701 | 71 |
| MCC54 | MAP4 | p.P895L | tpmPsrpst | tpmLsrpst | B0702 | 44 |
| MCC54 | MFSD5 | p.P448S | teepyaPel | teepyaSel | C0701 | 427 |
| MCC54 | MGA | p.P1177S | vyiptPsvi | vyiptSsvi | C0702 | 421 |
| MCC54 | MLLT3 | p.R440C | gsrsRrvsl | gsrsCrvsl | B0702 | 401 |
| MCC54 | MMRN1 | p.G236R | vtyvpggkG | vtyvpggkR | A0301 | 303 |
| MCC54 | MROH2B | p.G706R | mviyGaval | mviyRaval | B0702 | 49 |
| MCC54 | MROH2B | p.G706R | ktdvmviyG | ktdvmviyR | A0301 | 479 |
| MCC54 | MROH2B | p.G706R | viyGavalh | viyRavalh | A0301 | 174 |
| MCC54 | MTBP | p.W376R | ssrkWkeyi | ssrkRkeyi | C0701 | 328 |
| MCC54 | MTBP | p.E863K | rlfEiskfy | rlfKiskfy | A0301 | 31 |
| MCC54 | MTBP | p.E863K | tacsqrlfE | tacsqrlfK | A0301 | 195 |
| MCC54 | MTBP | p.E863K | sqrlfEisk | sqrlfKisk | A0301 | 338 |
| MCC54 | MUC16 | p.E11484K | Epettaiql | Kpettaiql | B0702 | 80 |
| MCC54 | MYO1E | p.H492Y | klqmqigsH | klqmqigsY | A0301 | 324 |
| MCC54 | MYO1E | p.V132M | yimsyisrV | yimsyisrM | A2601 | 23 |
| MCC54 | MYO1E | p.V132M | yimsyisrV | yimsyisrM | C0701 | 88 |
| MCC54 | MYO1E | p.V132M | yimsyisrV | yimsyisrM | C0702 | 121 |
| MCC54 | MYPN | p.S475F | vshpSvqtk | vshpFvqtk | A0301 | 197 |
| MCC54 | NAA35 | p.S398F | mvkdalrSf | mvkdalrFf | A2601 | 88 |
| MCC54 | NAA35 | p.S398F | rSfvsppvl | rFfvsppvl | C0702 | 455 |
| MCC54 | NBEAL1 | p.T970I | Tlmdvnvlm | Ilmdvnvlm | C0701 | 478 |
| MCC54 | NCKAP5L | p.P819T | klPsksptk | klTsksptk | A0301 | 40 |
| MCC54 | NCOA5 | p.P105L | Pmydryrdm | Lmydryrdm | C0702 | 353 |
| MCC54 | NCOA5 | p.P105L | frdqrdPmy | frdqrdLmy | C0701 | 94 |
| MCC54 | NEK9 | p.Q864L | aplehkpQv | aplehkpLv | B0702 | 458 |
| MCC54 | NEO1 | p.P439H | lPsaprdvv | lHsaprdvv | C0701 | 216 |
| MCC54 | NLGN1 | p.L232F | glLdliqal | glFdliqal | C0702 | 457 |
| MCC54 | NLRX1 | p.R470C | lhifRrdal | lhifCrdal | C0701 | 491 |
| MCC54 | NPR2 | p.G994S | srmesnGqa | srmesnSqa | C0701 | 231 |
| MCC54 | NTSR1 | p.R389K | clcpvwrrR | clcpvwrrK | A0301 | 149 |
| MCC54 | NTSR1 | p.R389K | rrRrkrpaf | rrKrkrpaf | C0702 | 337 |
| MCC54 | NUTM1 | p.E1034K | sprEhplsp | sprKhplsp | B0702 | 84 |
| MCC54 | NUTM1 | p.E1034K | slsprEhpl | slsprKhpl | C0702 | 105 |
| MCC54 | OPCML | p.R120W | hpktsRvhl | hpktsWvhl | B0702 | 36 |
| MCC54 | OR2T10 | p.M228I | kMnsvegrk | kInsvegrk | A0301 | 46 |
| MCC54 | OR4N5 | p.S145L | pracyalSl | pracyalLl | C0701 | 271 |
| MCC54 | OR4N5 | p.S145L | npracyalS | npracyalL | B0702 | 17 |
| MCC54 | OR51B2 | p.S163L | rlfSfsyck | rlfLfsyck | A0301 | 21 |
| MCC54 | OR51B2 | p.S163L | lpvilrlfS | lpvilrlfL | B0702 | 29 |
| MCC54 | OR51B2 | p.S163L | ilrlfSfsy | ilrlfLfsy | A0301 | 297 |
| MCC54 | OR6N1 | p.E296K | fiyslrnkE | fiyslrnkK | A0301 | 48 |
| MCC54 | OR6V1 | p.G249S | hltlvfiGy | hltlvfiSy | A0301 | 192 |
| MCC54 | OR6V1 | p.G249S | Gysstifly | Sysstifly | C0702 | 229 |
| MCC54 | OSCP1 | p.S174F | rvSmflkdk | rvFmflkdk | A0301 | 62 |
| MCC54 | OSCP1 | p.S174F | hirvSmflk | hirvFmflk | A0301 | 27 |
| MCC54 | OSTN | p.R115Q | rRfgipmdr | rQfgipmdr | A0301 | 365 |
| MCC54 | PCDH15 | p.S1755F | tplpppppS | tplpppppF | B0702 | 76 |
| MCC54 | PCDH15 | p.N1400Y | yhfqqsrgN | yhfqqsrgY | C0701 | 13 |
| MCC54 | PCDH15 | p.R1343Q | ktaRiqaal | ktaQiqaal | B0702 | 324 |
| MCC54 | PCDHA10 | p.R622C | aripfRvgl | aripfCvgl | C0702 | 172 |
| MCC54 | PCDHA10 | p.R622C | ripfRvgly | ripfCvgly | A0301 | 253 |
| MCC54 | PCDHB7 | p.E203K | vlnqvldrE | vlnqvldrK | A0301 | 191 |
| MCC54 | PCNX | p.E1348K | fEklhvwll | fKklhvwll | C0701 | 414 |
| MCC54 | PDCD6IP | p.T746P | rTmpptkpq | rPmpptkpq | B0702 | 12 |
| MCC54 | PDCD6IP | p.T746P | pptpaprTm | pptpaprPm | B0702 | 72 |
| MCC54 | PEAK1 | p.S1236P | iSslttlsi | iPslttlsi | B0702 | 19 |
| MCC54 | PEG3 | p.E454K | flhssaliE | flhssaliK | A0301 | 40 |
| MCC54 | PHF3 | p.S574F | ahSvlkktl | ahFvlkktl | C0701 | 72 |
| MCC54 | PHF3 | p.S574F | nqahSvlkk | nqahFvlkk | A0301 | 307 |
| MCC54 | PIK3C2G | p.V742A | irkvaVqql | irkvaAqql | C0701 | 84 |
| MCC54 | PIWIL1 | p.R146C | arrlRsall | arrlCsall | C0701 | 116 |
| MCC54 | PIWIL1 | p.R146C | rrlRsallf | rrlCsallf | C0702 | 402 |
| MCC54 | PKN3 | p.R375G | vhwRdwrql | vhwGdwrql | C0701 | 172 |
| MCC54 | PKN3 | p.R375G | vhwRdwrql | vhwGdwrql | C0702 | 393 |
| MCC54 | PLCE1 | p.V186I | hpdsrraVf | hpdsrraIf | B0702 | 18 |
| MCC54 | PLCE1 | p.V186I | ahpdsrraV | ahpdsrraI | C0701 | 39 |
| MCC54 | PNPLA7 | p.E28K | hswglwftE | hswglwftK | A0301 | 84 |
| MCC54 | POLQ | p.H2344Y | ahlsHdrrl | ahlsYdrrl | C0701 | 59 |
| MCC54 | POLR1B | p.E744K | atpfifseE | atpfifseK | A0301 | 279 |
| MCC54 | POM121L2 | p.H614Y | thasthhfH | thasthhfY | C0701 | 158 |
| MCC54 | POP1 | p.P41L | khhsggekP | khhsggekL | C0701 | 58 |
| MCC54 | POP1 | p.P41L | hhsggekPf | hhsggekLf | C0701 | 468 |
| MCC54 | PREX1 | p.R330G | frgRintev | frgGintev | C0701 | 45 |
| MCC54 | PRPF18 | p.R110K | qrlRkieil | qrlKkieil | C0702 | 311 |
| MCC54 | PRPF18 | p.R110K | qrlRkieil | qrlKkieil | C0701 | 59 |
| MCC54 | PRPF4 | p.H460Y | kfepiHgnf | kfepiYgnf | C0702 | 281 |
| MCC54 | PTCHD2 | p.S1248F | sSvdycvhl | sFvdycvhl | C0702 | 164 |
| MCC54 | PTCHD2 | p.S1248F | Svdycvhlv | Fvdycvhlv | C0701 | 277 |
| MCC54 | PTDSS1 | p.R102Q | rphpalwRm | rphpalwQm | B0702 | 15 |
| MCC54 | PTDSS1 | p.R102Q | hpalwRmvf | hpalwQmvf | B0702 | 25 |
| MCC54 | PTPN14 | p.E275K | ilvelinkE | ilvelinkK | A0301 | 142 |
| MCC54 | RFX6 | p.H522Y | sfHlirmll | sfYlirmll | C0702 | 158 |
| MCC54 | RNF144A | p.G256R | faGfgllll | faRfgllll | B0702 | 330 |
| MCC54 | RNF144A | p.G256R | aGfgllllv | aRfgllllv | C0701 | 24 |
| MCC54 | RNF144A | p.G256R | qvvgifaGf | qvvgifaRf | A2601 | 11 |
| MCC54 | RNF166 | p.S102F | fSydtfvdy | fFydtfvdy | C0702 | 149 |
| MCC54 | RNF166 | p.S102F | llhrhkfSy | llhrhkfFy | A0301 | 58 |
| MCC54 | RP1 | p.E536K | erkkEnsll | erkkKnsll | C0701 | 46 |
| MCC54 | RPL30 | p.R90K | gtacgkyyR | gtacgkyyK | A0301 | 281 |
| MCC54 | RPL30 | p.R90K | yyRvctlai | yyKvctlai | C0702 | 242 |
| MCC54 | RYR2 | p.E87K | emlantvEk | emlantvKk | A0301 | 344 |
| MCC54 | SAA2-SAA4 | p.G163R | qGlidcylf | qRlidcylf | C0702 | 356 |
| MCC54 | SAA2-SAA4 | p.G163R | srvylqGli | srvylqRli | C0701 | 184 |
| MCC54 | SART1 | p.G191R | klgkiktlG | klgkiktlR | A0301 | 342 |
| MCC54 | SCAND3 | p.C603F | flsCvvcek | flsFvvcek | A0301 | 151 |
| MCC54 | SCN2A | p.D322N | eyieDkshf | eyieNkshf | C0702 | 325 |
| MCC54 | SEL1L2 | p.E236K | Evalsyykk | Kvalsyykk | A0301 | 46 |
| MCC54 | SEMA6B | p.A184S | Alfsdgmlf | Slfsdgmlf | C0702 | 384 |
| MCC54 | SHANK2 | p.R479Q | srspslNrl | srspslQrl | C0702 | 85 |
| MCC54 | SHANK2 | p.R479Q | rsrspslNr | rsrspslQr | A0301 | 183 |
| MCC54 | SHANK2 | p.R479Q | srspslNrl | srspslQrl | C0701 | 15 |
| MCC54 | SLC12A5 | p.S361F | Syltkgviv | Fyltkgviv | C0702 | 406 |
| MCC54 | SLC12A5 | p.S361F | nlwsSyltk | nlwsFyltk | A0301 | 58 |
| MCC54 | SLC14A2 | p.N241S | lpfNiavtl | lpfSiavtl | B0702 | 170 |
| MCC54 | SLC17A1 | p.D4N | qmDnrlppk | qmNnrlppk | A0301 | 46 |
| MCC54 | SLC17A6 | p.H487Y | vHyggvify | vYyggvify | C0702 | 180 |
| MCC54 | SLC17A6 | p.H487Y | fliaalvHy | fliaalvYy | A2601 | 61 |
| MCC54 | SLC20A1 | p.E300K | Evgpatvpl | Kvgpatvpl | B0702 | 56 |
| MCC54 | SLC20A1 | p.E300K | hpvsEvgpa | hpvsKvgpa | B0702 | 38 |
| MCC54 | SLC25A20 | p.D259N | irDegvtsl | irNegvtsl | C0702 | 389 |
| MCC54 | SLC25A20 | p.D259N | irDegvtsl | irNegvtsl | C0701 | 26 |
| MCC54 | SLC2A7 | p.P220S | Pesprysli | Sesprysli | C0701 | 293 |
| MCC54 | SLC46A2 | p.G405R | likgssyGk | likgssyRk | A0301 | 173 |
| MCC54 | SLC46A2 | p.G405R | syGkvfvil | syRkvfvil | C0702 | 95 |
| MCC54 | SLC46A2 | p.G405R | klikgssyG | klikgssyR | A0301 | 52 |
| MCC54 | SLC6A1 | p.S340F | Sivgfmahv | Fivgfmahv | A2601 | 120 |
| MCC54 | SLC6A1 | p.S340F | fvifSivgf | fvifFivgf | A2601 | 33 |
| MCC54 | SMAD9 | p.S255F | qaSsrsvli | qaFsrsvli | C0701 | 114 |
| MCC54 | SPAG9 | p.Y497F | fgwslpqkY | fgwslpqkF | C0702 | 302 |
| MCC54 | SPATS2 | p.R353W | Rftcdvetl | Wftcdvetl | C0702 | 166 |
| MCC54 | STXBP6 | p.P139S | yltdrkPef | yltdrkSef | C0701 | 325 |
| MCC54 | STXBP6 | p.P139S | yltdrkPef | yltdrkSef | C0702 | 130 |
| MCC54 | TDRD5 | p.P251L | gyPsqqhyf | gyLsqqhyf | C0702 | 339 |
| MCC54 | TDRD6 | p.G551S | Gyyraivtk | Syyraivtk | A0301 | 478 |
| MCC54 | TERF2 | p.S431F | appSkptvl | appFkptvl | B0702 | 90 |
| MCC54 | TG | p.S1990F | vpmseksiS | vpmseksiF | B0702 | 60 |
| MCC54 | TMEM202 | p.Y194F | yYlnwcsdi | yFlnwcsdi | C0702 | 444 |
| MCC54 | TRIO | p.E455K | fhqkaEkym | fhqkaKkym | C0701 | 17 |
| MCC54 | TRIO | p.E455K | sifhqkaEk | sifhqkaKk | A0301 | 38 |
| MCC54 | TRIO | p.E455K | ssifhqkaE | ssifhqkaK | A0301 | 275 |
| MCC54 | TSPAN16 | p.S194N | sftlSgssl | sftlNgssl | C0702 | 492 |
| MCC54 | TTN | p.V10728A | vpevpkeVv | vpevpkeAv | B0702 | 248 |
| MCC54 | USH2A | p.E2321G | gplvEnrtl | gplvGnrtl | B0702 | 39 |
| MCC54 | USP21 | p.L214F | Lrnlgntcf | Frnlgntcf | C0702 | 133 |
| MCC54 | WDR35 | p.P644S | Pehpnkdyl | Sehpnkdyl | C0702 | 487 |
| MCC54 | WDR35 | p.P644S | Pehpnkdyl | Sehpnkdyl | C0701 | 248 |
| MCC54 | XKR9 | p.H34Y | ffHegqyvf | ffYegqyvf | C0702 | 28 |
| MCC54 | ZBBX | p.D259N | lhaavkDsl | lhaavkNsl | C0701 | 289 |
| MCC54 | ZNF10 | p.S125L | arndlwylS | arndlwylL | C0701 | 123 |
| MCC54 | ZNF10 | p.S125L | arndlwylS | arndlwylL | C0702 | 269 |
| MCC54 | ZNF202 | p.P388L | tPvhpllgr | tLvhpllgr | A0301 | 375 |
| MCC54 | ZNF236 | p.P1194L | vrihtgekP | vrihtgekL | C0701 | 125 |
| MCC54 | ZNF514 | p.T399I | qrshagkkT | qrshagkkI | C0701 | 384 |
| MCC54 | ZNF608 | p.S999R | yyhSydpyy | yyhRydpyy | C0702 | 92 |
| MCC54 | ZNF608 | p.S999R | shspyyhSy | shspyyhRy | C0701 | 64 |
| MCC54 | ZNF608 | p.S999R | shspyyhSy | shspyyhRy | C0702 | 197 |
| MCC54 | ZNF608 | p.S999R | pyyhSydpy | pyyhRydpy | C0702 | 384 |
| MCC54 | ZNF700 | p.P79L | eyqnPrrsf | eyqnLrrsf | C0702 | 282 |
| MCC54 | ZNF700 | p.P79L | Prrsfrsli | Lrrsfrsli | C0701 | 47 |
| MCC54 | ZNF700 | p.P79L | nPrrsfrsl | nLrrsfrsl | B0702 | 100 |
| MCC54 | ZNF816 | p.S215L | sSftqiqei | sLftqiqei | C0701 | 74 |
| MCC54 | ZUFSP | p.H172Y | ehsedmetH | ehsedmetY | C0701 | 281 |
| MCC56 | ABCC9 | p.A254T | Amravtnyv | Tmravtnyv | A0201 | 102 |
| MCC56 | ACOX1 | p.A394T | mmlqtArfl | mmlqtTrfl | A0201 | 38 |
| MCC56 | ACOX1 | p.A394T | vmmlqtArf | vmmlqtTrf | A2301 | 419 |
| MCC56 | ACOX1 | p.A394T | mlqtArflm | mlqtTrflm | A0201 | 242 |
| MCC56 | ADAM28 | p.G346E | hemghnfGm | hemghnfEm | B4403 | 68 |
| MCC56 | ADAM28 | p.G346E | hemghnfGm | hemghnfEm | C0701 | 391 |
| MCC56 | ADAMTSL2 | p.F453I | thfasqefF | thfasqefI | C0701 | 27 |
| MCC56 | ADAMTSL2 | p.F453I | qefFsanai | qefIsanai | B4403 | 263 |
| MCC56 | ADGB | p.L1261M | vLynswplt | vMynswplt | A0201 | 182 |
| MCC56 | ADGB | p.L1261M | svLynswpl | svMynswpl | A0201 | 8 |
| MCC56 | ADGB | p.L1261M | Lynswplte | Mynswplte | A2301 | 404 |
| MCC56 | AFF3 | p.D1191N | Dldllmgpv | Nldllmgpv | A0201 | 216 |
| MCC56 | AFF3 | p.D1191N | reffnDldl | reffnNldl | B4403 | 425 |
| MCC56 | AFF3 | p.D1191N | effnDldll | effnNldll | C0701 | 498 |
| MCC56 | AGPAT4 | p.G246E | vlnGkkyha | vlnEkkyha | A0201 | 266 |
| MCC56 | ANK2 | p.P303L | lllergaPl | lllergaLl | A0201 | 40 |
| MCC56 | ANPEP | p.P371L | rensllfdP | rensllfdL | B4403 | 71 |
| MCC56 | ANPEP | p.P371L | llfdPlsss | llfdLlsss | A0201 | 118 |
| MCC56 | AP3M1 | p.P348L | itPqklpsl | itLqklpsl | A0201 | 432 |
| MCC56 | AP4M1 | p.S84F | llSrlatll | llFrlatll | A0201 | 58 |
| MCC56 | AP4M1 | p.S84F | sllellSrl | sllellFrl | A0201 | 4 |
| MCC56 | ARHGAP39 | p.E1015K | fyEqciahy | fyKqciahy | A2301 | 324 |
| MCC56 | ARHGEF12 | p.R1266K | qhfpryRta | qhfpryKta | C0701 | 275 |
| MCC56 | ASB4 | p.G423E | ylllepeGi | ylllepeEi | A0201 | 34 |
| MCC56 | ASB4 | p.G423E | lllepeGii | lllepeEii | A0201 | 230 |
| MCC56 | ASH1L | p.P582L | sqlapnPll | sqlapnLll | A0201 | 436 |
| MCC56 | ASH1L | p.P582L | qlapnPlll | qlapnLlll | A0201 | 67 |
| MCC56 | ATP10A | p.S1428F | rvtplsSlf | rvtplsFlf | A2301 | 276 |
| MCC56 | ATP10A | p.S1428F | sSlfslptf | sFlfslptf | A2301 | 24 |
| MCC56 | ATP10A | p.S1428F | Slfslptfs | Flfslptfs | A0201 | 58 |
| MCC56 | ATP10A | p.R850C | llfqsaiRl | llfqsaiCl | A0201 | 49 |
| MCC56 | ATP13A5 | p.P101S | ylstlkfPv | ylstlkfSv | A0201 | 4 |
| MCC56 | ATP1A3 | p.M822I | aesdiMkrq | aesdiIkrq | B4403 | 107 |
| MCC56 | ATP6V0A1 | p.P205L | aeienPled | aeienLled | B4403 | 194 |
| MCC56 | ATP6V0A1 | p.P205L | lrqaeienP | lrqaeienL | C0701 | 295 |
| MCC56 | ATP6V1H | p.G39R | ylqGqmisa | ylqRqmisa | A0201 | 35 |
| MCC56 | BAI3 | p.G1019R | leGgllyaf | leRgllyaf | B4403 | 221 |
| MCC56 | BAI3 | p.G1019R | sleGgllya | sleRgllya | A0201 | 334 |
| MCC56 | BAZ1A | p.R1106C | hlstldRsv | hlstldCsv | A0201 | 104 |
| MCC56 | BCS1L | p.G371E | fypGqapsl | fypEqapsl | C0401 | 121 |
| MCC56 | BCS1L | p.G371E | fypGqapsl | fypEqapsl | C0701 | 237 |
| MCC56 | BICC1 | p.S859F | nymdciSsl | nymdciFsl | A0201 | 344 |
| MCC56 | BICC1 | p.S859F | ymdciSslt | ymdciFslt | A0201 | 37 |
| MCC56 | BICC1 | p.S859F | nymdciSsl | nymdciFsl | A2301 | 30 |
| MCC56 | BICC1 | p.S859F | nymdciSsl | nymdciFsl | C0701 | 83 |
| MCC56 | BRAF | p.P655L | yelmtgqlP | yelmtgqlL | B4403 | 266 |
| MCC56 | BTBD11 | p.A100V | kldavAiea | kldavViea | A0201 | 15 |
| MCC56 | BTBD11 | p.A100V | srkldavAi | srkldavVi | C0701 | 415 |
| MCC56 | BTRC | p.G520R | tlvehsGrv | tlvehsRrv | A0201 | 180 |
| MCC56 | BTRC | p.G520R | tlvehsGrv | tlvehsRrv | C0701 | 442 |
| MCC56 | BTRC | p.G520R | ehsGrvfrl | ehsRrvfrl | C0701 | 62 |
| MCC56 | C10orf62 | p.E45K | shfsrlseE | shfsrlseK | C0701 | 97 |
| MCC56 | C10orf62 | p.E45K | rlseEklal | rlseKklal | A0201 | 175 |
| MCC56 | C16orf46 | p.P202L | Pltsrallv | Lltsrallv | A0201 | 71 |
| MCC56 | C16orf46 | p.P202L | gPltsrall | gLltsrall | A0201 | 264 |
| MCC56 | C1RL | p.S17F | kylwrsphS | kylwrsphF | A2301 | 26 |
| MCC56 | C2orf62 | p.W293L | lvWeedmel | lvLeedmel | A0201 | 175 |
| MCC56 | C9orf153 | p.S49L | iSlneaqev | iLlneaqev | A0201 | 18 |
| MCC56 | C9orf153 | p.S49L | Slneaqevl | Llneaqevl | A0201 | 165 |
| MCC56 | CAD | p.L2067V | eeLgtvngm | eeVgtvngm | B4403 | 151 |
| MCC56 | CAND2 | p.P1004L | lisdqphPi | lisdqphLi | C0701 | 233 |
| MCC56 | CAND2 | p.P1004L | lisdqphPi | lisdqphLi | A0201 | 353 |
| MCC56 | CAND2 | p.P1004L | flisdqphP | flisdqphL | A0201 | 4 |
| MCC56 | CCDC170 | p.R470C | arteqlvRl | arteqlvCl | C0701 | 65 |
| MCC56 | CCDC28A | p.G17E | aGlplgawr | aElplgawr | B4403 | 179 |
| MCC56 | CCNA1 | p.E284K | kmEhlllkv | kmKhlllkv | A0201 | 91 |
| MCC56 | CCNA1 | p.E284K | qllkmEhll | qllkmKhll | A0201 | 453 |
| MCC56 | CD300LD | p.P175L | fllelPlll | fllelLlll | A0201 | 4 |
| MCC56 | CD300LD | p.P175L | flfllelPl | flfllelLl | A0201 | 23 |
| MCC56 | CD300LD | p.P175L | lflfllelP | lflfllelL | A2301 | 128 |
| MCC56 | CD300LD | p.P175L | lfllelPll | lfllelLll | A2301 | 235 |
| MCC56 | CDH10 | p.P467S | aeinnPket | aeinnSket | B4403 | 294 |
| MCC56 | CDH7 | p.P357L | gPfsdtttv | gLfsdtttv | A0201 | 8 |
| MCC56 | CEACAM5 | p.T276I | yswfvngTf | yswfvngIf | A2301 | 329 |
| MCC56 | CEACAM5 | p.T276I | Tfqqstqel | Ifqqstqel | A2301 | 230 |
| MCC56 | CEP120 | p.P124L | Prdgkvpai | Lrdgkvpai | C0701 | 175 |
| MCC56 | CEP128 | p.L294V | qelelsrrL | qelelsrrV | B4403 | 179 |
| MCC56 | CHD1 | p.P1008L | thenepgPl | thenepgLl | C0701 | 39 |
| MCC56 | CHD1 | p.P1008L | gPltvgdel | gLltvgdel | A0201 | 198 |
| MCC56 | COASY | p.E493K | avipEteav | avipKteav | A0201 | 364 |
| MCC56 | CPAMD8 | p.A1030V | vrahndArv | vrahndVrv | C0701 | 272 |
| MCC56 | CPAMD8 | p.A1030V | ahndArval | ahndVrval | C0701 | 40 |
| MCC56 | CPAMD8 | p.H593Y | Hlavtpsmv | Ylavtpsmv | A0201 | 22 |
| MCC56 | CPAMD8 | p.H593Y | lHlavtpsm | lYlavtpsm | A2301 | 250 |
| MCC56 | CPQ | p.S310L | iSwealsli | iLwealsli | A0201 | 7 |
| MCC56 | CPQ | p.S310L | fiSwealsl | fiLwealsl | A0201 | 31 |
| MCC56 | CREB3L2 | p.P235L | fslpqthsP | fslpqthsL | C0701 | 356 |
| MCC56 | CYP4X1 | p.Q114K | ylQkfsppl | ylKkfsppl | A0201 | 12 |
| MCC56 | DEFB110 | p.R52K | neiRiayci | neiKiayci | B4403 | 50 |
| MCC56 | DEFB110 | p.R52K | heneiRiay | heneiKiay | B4403 | 17 |
| MCC56 | DGKQ | p.L461P | rhvqrtmLm | rhvqrtmPm | C0701 | 271 |
| MCC56 | DGKQ | p.L461P | mLmdeqpll | mPmdeqpll | B5101 | 209 |
| MCC56 | DHX33 | p.V269L | qrcnlasVm | qrcnlasLm | C0701 | 66 |
| MCC56 | DHX33 | p.V269L | sVmlqllam | sLmlqllam | A0201 | 109 |
| MCC56 | DHX33 | p.V269L | nlasVmlql | nlasLmlql | A0201 | 49 |
| MCC56 | DLX6 | p.R185H | hRfqqtqyl | hHfqqtqyl | C0701 | 5 |
| MCC56 | DLX6 | p.R185H | qlqalnhRf | qlqalnhHf | A2301 | 192 |
| MCC56 | DMPK | p.P257S | sygPecdww | sygSecdww | A2301 | 335 |
| MCC56 | DPP10 | p.S294F | aqniSiltv | aqniFiltv | A0201 | 146 |
| MCC56 | DPP10 | p.S294F | nraqniSil | nraqniFil | C0701 | 36 |
| MCC56 | DYNC1I2 | p.F471Y | avdfshlFv | avdfshlYv | A0201 | 251 |
| MCC56 | DYNC1I2 | p.F471Y | shlFvtssf | shlYvtssf | A2301 | 240 |
| MCC56 | DYNC1I2 | p.F471Y | lFvtssfdw | lYvtssfdw | A2301 | 87 |
| MCC56 | DYSF | p.H1066Y | slfgwkfHl | slfgwkfYl | C0701 | 244 |
| MCC56 | DYSF | p.H1066Y | slfgwkfHl | slfgwkfYl | A0201 | 3 |
| MCC56 | ENPP5 | p.H76Y | Hytlvtglf | Yytlvtglf | A2301 | 35 |
| MCC56 | ENPP5 | p.H76Y | ktypnHytl | ktypnYytl | A0201 | 110 |
| MCC56 | ENPP5 | p.H76Y | ktypnHytl | ktypnYytl | A2301 | 80 |
| MCC56 | ENPP5 | p.H76Y | typnHytlv | typnYytlv | A2301 | 457 |
| MCC56 | EPHA6 | p.L457F | grkdLtysv | grkdFtysv | C0701 | 273 |
| MCC56 | EPHB4 | p.V689L | mpVmiltef | mpLmiltef | B5101 | 414 |
| MCC56 | EPN1 | p.P375L | tePdefsdf | teLdefsdf | A2301 | 469 |
| MCC56 | EPN1 | p.P375L | tePdefsdf | teLdefsdf | B4403 | 70 |
| MCC56 | FAT3 | p.G2137E | sGnvilkea | sEnvilkea | B4403 | 482 |
| MCC56 | FERMT1 | p.P92S | llftPqhkm | llftSqhkm | A0201 | 188 |
| MCC56 | FGL1 | p.Y80F | qYadcseif | qFadcseif | A2301 | 118 |
| MCC56 | FGL1 | p.Y80F | rqYadcsei | rqFadcsei | A0201 | 490 |
| MCC56 | FLT1 | p.P365L | kafpsPevv | kafpsLevv | A0201 | 495 |
| MCC56 | FLT3 | p.S102F | gniSclwvf | gniFclwvf | A2301 | 113 |
| MCC56 | FN3KRP | p.V231M | pVifdpasf | pMifdpasf | A2301 | 408 |
| MCC56 | FN3KRP | p.V231M | aedssgpVi | aedssgpMi | B4403 | 252 |
| MCC56 | FSHR | p.R608C | nfRrdffil | nfCrdffil | A2301 | 382 |
| MCC56 | FSTL4 | p.G451E | svGnmfyvf | svEnmfyvf | A2301 | 121 |
| MCC56 | FSTL4 | p.G451E | lsvGnmfyv | lsvEnmfyv | A0201 | 80 |
| MCC56 | FZR1 | p.T351I | klTghsyrv | klIghsyrv | A0201 | 3 |
| MCC56 | GATAD1 | p.S203F | qfdpaSyii | qfdpaFyii | A2301 | 169 |
| MCC56 | GNA12 | p.R110W | eafsRrsef | eafsWrsef | A2301 | 459 |
| MCC56 | GPCPD1 | p.S239N | feedlSehv | feedlNehv | C0701 | 383 |
| MCC56 | GPR116 | p.D751N | mlptylkDl | mlptylkNl | A0201 | 70 |
| MCC56 | GPR116 | p.D751N | ylkDlsisi | ylkNlsisi | A0201 | 45 |
| MCC56 | GPR15 | p.Y151H | rrtdcaYvv | rrtdcaHvv | C0701 | 13 |
| MCC56 | GPR15 | p.Y151H | frrtdcaYv | frrtdcaHv | C0701 | 399 |
| MCC56 | GPR31 | p.S292F | frsSyrrvf | frsFyrrvf | C0701 | 46 |
| MCC56 | GPR31 | p.S292F | Syrrvfhtl | Fyrrvfhtl | A2301 | 20 |
| MCC56 | GREB1 | p.D1397G | hylqlsDpw | hylqlsGpw | A2301 | 149 |
| MCC56 | GSDMC | p.L184F | ilgkiaLwi | ilgkiaFwi | A0201 | 46 |
| MCC56 | H2AFY | p.P217L | feveaiinP | feveaiinL | B4403 | 438 |
| MCC56 | HERC2 | p.G3118E | sGelytwgl | sEelytwgl | B4403 | 398 |
| MCC56 | HJURP | p.P43L | qPfedtpvv | qLfedtpvv | A0201 | 6 |
| MCC56 | HJURP | p.P43L | rliekynqP | rliekynqL | A0201 | 63 |
| MCC56 | HSPB2 | p.E116K | srEfcrtyv | srKfcrtyv | C0701 | 74 |
| MCC56 | IFI44L | p.S219F | ffnsvkSif | ffnsvkFif | A2301 | 27 |
| MCC56 | IKZF5 | p.S263F | tlagqlSsl | tlagqlFsl | A0201 | 9 |
| MCC56 | IL1RL1 | p.P450L | rrhifiltP | rrhifiltL | C0701 | 128 |
| MCC56 | ITGA7 | p.R1085W | qyhavkipR | qyhavkipW | A2301 | 316 |
| MCC56 | KCNJ3 | p.E363K | Emllmsspl | Kmllmsspl | A0201 | 21 |
| MCC56 | KDM5A | p.G474E | yvGmcfssf | yvEmcfssf | A2301 | 265 |
| MCC56 | KDM5A | p.G474E | pwlyvGmcf | pwlyvEmcf | A2301 | 49 |
| MCC56 | KIAA1549L | p.G1745E | Genelpsqw | Eenelpsqw | B4403 | 15 |
| MCC56 | KIF11 | p.E345K | Etlstleya | Ktlstleya | A0201 | 324 |
| MCC56 | KLRC1 | p.S105F | Slntrtqka | Flntrtqka | A0201 | 208 |
| MCC56 | KLRD1 | p.R140C | Ryickqqli | Cyickqqli | A2301 | 59 |
| MCC56 | KLRF1 | p.G65R | lillvsqGv | lillvsqRv | A0201 | 303 |
| MCC56 | LCT | p.R982W | fsiswsRif | fsiswsWif | A2301 | 22 |
| MCC56 | LCT | p.R982W | iswsRifpt | iswsWifpt | A0201 | 129 |
| MCC56 | LCT | p.R982W | yrfsiswsR | yrfsiswsW | C0701 | 397 |
| MCC56 | LIMS2 | p.E158K | cyEkfplel | cyKkfplel | A2301 | 261 |
| MCC56 | LPHN2 | p.P651L | smPtenivl | smLtenivl | A0201 | 42 |
| MCC56 | LRP1B | p.M4515I | glldpgfMi | glldpgfIi | A0201 | 34 |
| MCC56 | LRPPRC | p.P336L | iPdamnlil | iLdamnlil | A0201 | 59 |
| MCC56 | LRPPRC | p.P336L | yiPdamnli | yiLdamnli | A0201 | 6 |
| MCC56 | LRPPRC | p.P336L | vtcerryiP | vtcerryiL | C0701 | 389 |
| MCC56 | LRPPRC | p.P336L | ryiPdamnl | ryiLdamnl | A2301 | 244 |
| MCC56 | LRRC66 | p.D577N | srydsnelD | srydsnelN | C0701 | 201 |
| MCC56 | LRRC7 | p.S688L | ySdyspsqa | yLdyspsqa | A0201 | 73 |
| MCC56 | LRRC70 | p.P59S | Pestvflyl | Sestvflyl | B4403 | 38 |
| MCC56 | MAP1A | p.P2455S | flnPplpps | flnSplpps | A0201 | 200 |
| MCC56 | MAST3 | p.S348F | eeqppapeS | eeqppapeF | B4403 | 340 |
| MCC56 | MC3R | p.V140I | iaVdryvti | iaIdryvti | B5101 | 144 |
| MCC56 | MDM1 | p.P177L | vpPfkgnsv | vpLfkgnsv | B5101 | 303 |
| MCC56 | MED1 | p.P1052L | gsagrsqtP | gsagrsqtL | C0701 | 455 |
| MCC56 | MED1 | p.P1052L | Ppgvatppi | Lpgvatppi | B5101 | 158 |
| MCC56 | MEIS2 | p.S125F | vraekplfS | vraekplfF | C0701 | 80 |
| MCC56 | MEIS2 | p.S125F | plfSsnpel | plfFsnpel | A0201 | 402 |
| MCC56 | MFSD9 | p.F306Y | vmlyysnFv | vmlyysnYv | A0201 | 3 |
| MCC56 | MFSD9 | p.F306Y | lyysnFvla | lyysnYvla | A2301 | 275 |
| MCC56 | MFSD9 | p.F306Y | mlyysnFvl | mlyysnYvl | A0201 | 8 |
| MCC56 | MFSD9 | p.F306Y | yysnFvlal | yysnYvlal | C0701 | 57 |
| MCC56 | MFSD9 | p.F306Y | mlyysnFvl | mlyysnYvl | A2301 | 252 |
| MCC56 | MFSD9 | p.F306Y | Fvlaleerf | Yvlaleerf | A2301 | 271 |
| MCC56 | MFSD9 | p.F306Y | yysnFvlal | yysnYvlal | A2301 | 238 |
| MCC56 | MPP4 | p.G518E | vlveGkicv | vlveEkicv | A0201 | 61 |
| MCC56 | MPP4 | p.G518E | eGkicvmdl | eEkicvmdl | B4403 | 413 |
| MCC56 | MTFR1 | p.E55D | eegEcsarl | eegDcsarl | B4403 | 203 |
| MCC56 | MTUS1 | p.P1192S | krsPtssai | krsStssai | C0701 | 248 |
| MCC56 | MUC16 | p.S10353F | alheitSss | alheitFss | A0201 | 166 |
| MCC56 | MUC16 | p.S10353F | Sssatpyrv | Fssatpyrv | A0201 | 351 |
| MCC56 | MUC16 | p.P5898S | qrfPhseet | qrfShseet | C0701 | 82 |
| MCC56 | MUC16 | p.P5898S | Phseetttm | Shseetttm | C0701 | 11 |
| MCC56 | MYH8 | p.S1143F | aekqrsdlS | aekqrsdlF | B4403 | 464 |
| MCC56 | MYH8 | p.S1143F | qrsdlSrel | qrsdlFrel | C0701 | 36 |
| MCC56 | MYLK2 | p.G241E | sevGqalcl | sevEqalcl | B4403 | 79 |
| MCC56 | MYO16 | p.S20F | fqlcnvfrS | fqlcnvfrF | A2301 | 50 |
| MCC56 | MYO18B | p.S2357F | Srpsmgrkl | Frpsmgrkl | C0701 | 14 |
| MCC56 | MYO18B | p.S2357F | slleSrpsm | slleFrpsm | A0201 | 30 |
| MCC56 | NFASC | p.P49L | sakdhivdP | sakdhivdL | C0701 | 431 |
| MCC56 | NOC3L | p.L369V | niivlivpL | niivlivpV | A0201 | 62 |
| MCC56 | NOC3L | p.L369V | Lmndmskli | Vmndmskli | C0701 | 457 |
| MCC56 | NOC3L | p.L369V | Lmndmskli | Vmndmskli | A0201 | 416 |
| MCC56 | NR5A1 | p.L325P | ilLvtgqev | ilPvtgqev | A0201 | 462 |
| MCC56 | NUP210 | p.E1548K | lrtykEvvv | lrtykKvvv | C0701 | 354 |
| MCC56 | OLFM4 | p.P95S | fPvdrverl | fSvdrverl | C0701 | 68 |
| MCC56 | OLFM4 | p.P95S | slpdttfPv | slpdttfSv | A0201 | 5 |
| MCC56 | ONECUT2 | p.S460F | iSqqlglel | iFqqlglel | A2301 | 444 |
| MCC56 | ONECUT2 | p.S460F | kemqitiSq | kemqitiFq | B4403 | 250 |
| MCC56 | ONECUT2 | p.S460F | mqitiSqql | mqitiFqql | A0201 | 209 |
| MCC56 | OR2A12 | p.S274F | Slfyslfnp | Flfyslfnp | A0201 | 102 |
| MCC56 | OR2A12 | p.S274F | kilSlfysl | kilFlfysl | A0201 | 20 |
| MCC56 | OR2A12 | p.S274F | ilSlfyslf | ilFlfyslf | A2301 | 225 |
| MCC56 | OR2G3 | p.S217F | alisiSygf | alisiFygf | A0201 | 360 |
| MCC56 | OR2G3 | p.S217F | alisiSygf | alisiFygf | A2301 | 55 |
| MCC56 | OR4C6 | p.P251S | fvPciflym | fvSciflym | A0201 | 84 |
| MCC56 | OR4C6 | p.P251S | vlffvPcif | vlffvScif | A2301 | 450 |
| MCC56 | OR4C6 | p.P251S | ffvPcifly | ffvScifly | A2301 | 92 |
| MCC56 | OR4K1 | p.E196K | tyemEimtl | tyemKimtl | A2301 | 135 |
| MCC56 | OR4K1 | p.E196K | cmdtyemEi | cmdtyemKi | A0201 | 323 |
| MCC56 | OR4K1 | p.E196K | tyemEimtl | tyemKimtl | C0701 | 419 |
| MCC56 | OR4K1 | p.S288F | llnpiiySl | llnpiiyFl | A0201 | 5 |
| MCC56 | OR51G2 | p.S248F | Shicavllf | Fhicavllf | C0701 | 82 |
| MCC56 | OR51G2 | p.S248F | Shicavllf | Fhicavllf | A2301 | 71 |
| MCC56 | OR51G2 | p.S248F | alntcvShi | alntcvFhi | A0201 | 12 |
| MCC56 | OR5H15 | p.L63H | llLgnlafv | llHgnlafv | A0201 | 17 |
| MCC56 | OR5H15 | p.L63H | yllLgnlaf | yllHgnlaf | A2301 | 242 |
| MCC56 | OR5H15 | p.L63H | yllLgnlaf | yllHgnlaf | A0201 | 366 |
| MCC56 | OR6C70 | p.P56S | Pmyfflrnf | Smyfflrnf | A2301 | 392 |
| MCC56 | OR6C70 | p.P56S | sqlktPmyf | sqlktSmyf | A2301 | 270 |
| MCC56 | OR6N1 | p.S217F | fllilcSyv | fllilcFyv | A0201 | 2 |
| MCC56 | OR6N1 | p.S217F | ilcSyvqii | ilcFyvqii | A0201 | 249 |
| MCC56 | OR6N1 | p.S217F | Syvqiictv | Fyvqiictv | A2301 | 158 |
| MCC56 | OR6N1 | p.S217F | lilcSyvqi | lilcFyvqi | A0201 | 140 |
| MCC56 | OR6N1 | p.P138S | tlmtPtlca | tlmtStlca | A0201 | 42 |
| MCC56 | OR8B12 | p.F101Y | tqlFffcff | tqlYffcff | A2301 | 179 |
| MCC56 | OR8B12 | p.F101Y | mtqlFffcf | mtqlYffcf | A2301 | 86 |
| MCC56 | OR8B12 | p.F101Y | lFffcffvv | lYffcffvv | A2301 | 20 |
| MCC56 | OR8B12 | p.F101Y | qlFffcffv | qlYffcffv | A0201 | 5 |
| MCC56 | PAPOLB | p.V52I | gVfeeeeel | gIfeeeeel | A0201 | 321 |
| MCC56 | PCDHAC2 | p.E592K | Emvprtapa | Kmvprtapa | A0201 | 32 |
| MCC56 | PCDHAC2 | p.S876F | afvthppiS | afvthppiF | A2301 | 443 |
| MCC56 | PCDHGA10 | p.S758F | flqtyShev | flqtyFhev | A0201 | 2 |
| MCC56 | PCDHGB5 | p.L180I | slnpsfsLi | slnpsfsIi | A0201 | 233 |
| MCC56 | PDIA4 | p.E492K | kfamepeEf | kfamepeKf | A2301 | 177 |
| MCC56 | PIK3CA | p.M1004I | nlfinlfsM | nlfinlfsI | A0201 | 23 |
| MCC56 | PIK3CA | p.M1004I | finlfsMml | finlfsIml | A0201 | 112 |
| MCC56 | PIK3R4 | p.L337F | tfLsaderi | tfFsaderi | A2301 | 99 |
| MCC56 | PIK3R4 | p.L337F | Lsaderilv | Fsaderilv | C0701 | 396 |
| MCC56 | PKD1L1 | p.P1020L | iPpagdsav | iLpagdsav | A0201 | 130 |
| MCC56 | PKHD1L1 | p.E4020K | mqlselqEi | mqlselqKi | A2301 | 397 |
| MCC56 | PKHD1L1 | p.E4020K | mqlselqEi | mqlselqKi | A0201 | 307 |
| MCC56 | PLEKHG1 | p.P150S | irdqtklPl | irdqtklSl | C0701 | 129 |
| MCC56 | PLEKHG2 | p.P371S | Prdplgfkv | Srdplgfkv | C0701 | 324 |
| MCC56 | PNPLA8 | p.L136F | ehfrdkseL | ehfrdkseF | C0701 | 250 |
| MCC56 | PPM1F | p.S345F | geadaaSra | geadaaFra | B4403 | 316 |
| MCC56 | PPP1R3B | p.D87N | Dmpfnitel | Nmpfnitel | A0201 | 230 |
| MCC56 | PPP1R3B | p.D87N | sefddplDm | sefddplNm | B4403 | 117 |
| MCC56 | PPP1R3B | p.D87N | sefddplDm | sefddplNm | C0701 | 269 |
| MCC56 | PRG3 | p.G13R | flllGtvsa | flllRtvsa | A0201 | 29 |
| MCC56 | PRG3 | p.G13R | lGtvsalhl | lRtvsalhl | C0701 | 411 |
| MCC56 | PRG3 | p.G13R | lpflllGtv | lpflllRtv | B5101 | 120 |
| MCC56 | PRG3 | p.G13R | lllGtvsal | lllRtvsal | A0201 | 66 |
| MCC56 | PRKD3 | p.S91F | vcSivyqkf | vcFivyqkf | A2301 | 231 |
| MCC56 | PRMT10 | p.R82Q | alsRiqdll | alsQiqdll | A0201 | 183 |
| MCC56 | PRMT10 | p.R82Q | eeldalsRi | eeldalsQi | B4403 | 281 |
| MCC56 | PRSS41 | p.D230N | rsmiwDsmf | rsmiwNsmf | A2301 | 157 |
| MCC56 | PRSS41 | p.D230N | miwDsmfca | miwNsmfca | A0201 | 13 |
| MCC56 | PTRH1 | p.P26L | rPpgkrwmv | rLpgkrwmv | A0201 | 139 |
| MCC56 | QTRTD1 | p.M258T | llagvllmM | llagvllmT | A0201 | 196 |
| MCC56 | QTRTD1 | p.M258T | gvllmMhnf | gvllmThnf | A2301 | 236 |
| MCC56 | RASGRP3 | p.G246E | tlmavvGgl | tlmavvEgl | A0201 | 14 |
| MCC56 | RERGL | p.E31K | Eyasnfesi | Kyasnfesi | A2301 | 23 |
| MCC56 | REXO2 | p.P133S | vrqqtPpgl | vrqqtSpgl | C0701 | 138 |
| MCC56 | RGS3 | p.P332L | Plylqsvkl | Llylqsvkl | A0201 | 173 |
| MCC56 | RGS3 | p.P332L | grcdvlrnP | grcdvlrnL | C0701 | 155 |
| MCC56 | RNF44 | p.R390W | sdfeaRqll | sdfeaWqll | C0701 | 405 |
| MCC56 | ROS1 | p.G140E | kyaqllGsw | kyaqllEsw | A2301 | 57 |
| MCC56 | ROS1 | p.G140E | qllGswtyt | qllEswtyt | A0201 | 208 |
| MCC56 | RPS6KA2 | p.D160N | mfteeDvkf | mfteeNvkf | A2301 | 99 |
| MCC56 | RPS8 | p.S142F | eeilnkkrS | eeilnkkrF | B4403 | 32 |
| MCC56 | SCN1A | p.N1751S | flvvvNmyi | flvvvSmyi | A0201 | 15 |
| MCC56 | SCN1A | p.N1751S | vvvNmyiav | vvvSmyiav | A0201 | 210 |
| MCC56 | SCUBE3 | p.G665R | tfqereGql | tfqereRql | C0701 | 490 |
| MCC56 | SEMA6A | p.V914I | mhhsssygV | mhhsssygI | C0701 | 17 |
| MCC56 | SESTD1 | p.P206S | flpsvdPet | flpsvdSet | A0201 | 402 |
| MCC56 | SETBP1 | p.M1086I | tlgaaspfM | tlgaaspfI | A0201 | 60 |
| MCC56 | SGIP1 | p.S57L | areggkkvS | areggkkvL | C0701 | 188 |
| MCC56 | SLC12A1 | p.G243R | vrggGayyl | vrggRayyl | C0701 | 29 |
| MCC56 | SLC12A2 | p.E1142K | Ehsstanii | Khsstanii | C0701 | 95 |
| MCC56 | SLC15A1 | p.S15N | sffgyplSi | sffgyplNi | A2301 | 173 |
| MCC56 | SLC15A1 | p.S15N | ffgyplSif | ffgyplNif | A2301 | 109 |
| MCC56 | SLC15A1 | p.S15N | gyplSiffi | gyplNiffi | A2301 | 155 |
| MCC56 | SLC15A1 | p.S15N | yplSiffiv | yplNiffiv | B5101 | 115 |
| MCC56 | SLC15A1 | p.S15N | sffgyplSi | sffgyplNi | C0701 | 367 |
| MCC56 | SLC1A3 | p.R226Q | Riteelvpv | Qiteelvpv | A0201 | 89 |
| MCC56 | SLC1A3 | p.R226Q | tltRiteel | tltQiteel | A0201 | 193 |
| MCC56 | SLC35F1 | p.G211R | qGagenklv | qRagenklv | C0701 | 111 |
| MCC56 | SLC4A9 | p.V111M | vlfeekleV | vlfeekleM | A0201 | 62 |
| MCC56 | SLC4A9 | p.V111M | leVaagrws | leMaagrws | B4403 | 451 |
| MCC56 | SLC6A15 | p.G268E | illitisGf | illitisEf | A2301 | 150 |
| MCC56 | SLC6A15 | p.G268E | itisGfipl | itisEfipl | A0201 | 149 |
| MCC56 | SLC6A15 | p.G268E | llitisGfi | llitisEfi | A0201 | 26 |
| MCC56 | SLC6A16 | p.F667I | llmitlFai | llmitlIai | A0201 | 22 |
| MCC56 | SLC6A16 | p.F667I | alllmitlF | alllmitlI | A0201 | 89 |
| MCC56 | SLC6A16 | p.F667I | lllmitlFa | lllmitlIa | A0201 | 170 |
| MCC56 | SLC6A16 | p.F667I | lFaivilpi | lIaivilpi | A0201 | 136 |
| MCC56 | SLC6A16 | p.F667I | lmitlFaiv | lmitlIaiv | A0201 | 10 |
| MCC56 | SMARCC2 | p.P303L | nykkrkrsP | nykkrkrsL | C0701 | 443 |
| MCC56 | SNX4 | p.S202F | fqlkadSrl | fqlkadFrl | A0201 | 49 |
| MCC56 | SPTBN4 | p.D502N | laaegyyDi | laaegyyNi | A0201 | 491 |
| MCC56 | SPTBN4 | p.D502N | laaegyyDi | laaegyyNi | B5101 | 347 |
| MCC56 | SSPO | p.D3576Y | qlsqDglcv | qlsqYglcv | A0201 | 250 |
| MCC56 | SUPT6H | p.Q1692E | errkQkqrl | errkEkqrl | C0701 | 128 |
| MCC56 | SUPT6H | p.Q1692E | rrkQkqrlt | rrkEkqrlt | C0701 | 341 |
| MCC56 | SVEP1 | p.P2927S | yilhgaPkl | yilhgaSkl | A0201 | 141 |
| MCC56 | SWT1 | p.E737K | lknshnqEi | lknshnqKi | C0701 | 210 |
| MCC56 | SWT1 | p.E737K | shnqEitvf | shnqKitvf | C0701 | 21 |
| MCC56 | TAS2R7 | p.S239F | aviSflllf | aviFflllf | A2301 | 205 |
| MCC56 | TAS2R7 | p.S239F | viSflllfi | viFflllfi | A0201 | 79 |
| MCC56 | TAS2R7 | p.S239F | alkaviSfl | alkaviFfl | A0201 | 25 |
| MCC56 | TBR1 | p.S651L | sSsplksev | sLsplksev | A0201 | 59 |
| MCC56 | TDRD6 | p.N2060S | Nlsngmeei | Slsngmeei | A0201 | 56 |
| MCC56 | TGM5 | p.L412M | prsLhtpsl | prsMhtpsl | C0701 | 254 |
| MCC56 | THUMPD2 | p.L398F | qemervLhv | qemervFhv | B4403 | 226 |
| MCC56 | THUMPD2 | p.L398F | Lhvggtivl | Fhvggtivl | C0701 | 31 |
| MCC56 | THUMPD2 | p.L398F | qemervLhv | qemervFhv | C0701 | 433 |
| MCC56 | TMEM241 | p.L270M | llfdaiLts | llfdaiMts | A0201 | 71 |
| MCC56 | TMTC2 | p.P84S | glnPwsyhl | glnSwsyhl | A0201 | 10 |
| MCC56 | TNC | p.S1548Y | Swmasenaf | Ywmasenaf | A2301 | 66 |
| MCC56 | TNFRSF10D | p.D62E | atiprqDev | atiprqEev | C0701 | 370 |
| MCC56 | TPCN1 | p.P531S | mePfyfivv | meSfyfivv | B4403 | 224 |
| MCC56 | TPCN1 | p.P531S | nmePfyfiv | nmeSfyfiv | A0201 | 60 |
| MCC56 | TPCN2 | p.G584R | rafgGilvv | rafgRilvv | C0701 | 99 |
| MCC56 | TPCN2 | p.G584R | Gilvvvyyv | Rilvvvyyv | A0201 | 9 |
| MCC56 | TPCN2 | p.G584R | mrafgGilv | mrafgRilv | C0701 | 350 |
| MCC56 | TPRA1 | p.S198F | fwlvsScff | fwlvsFcff | A2301 | 42 |
| MCC56 | TPRA1 | p.S198F | lvsScfffl | lvsFcfffl | A0201 | 53 |
| MCC56 | TPRA1 | p.S198F | wlvsScfff | wlvsFcfff | A0201 | 125 |
| MCC56 | TPRA1 | p.S198F | qfwlvsScf | qfwlvsFcf | A2301 | 34 |
| MCC56 | TPRA1 | p.S198F | vsScfffll | vsFcfffll | A0201 | 180 |
| MCC56 | TPRA1 | p.S198F | wlvsScfff | wlvsFcfff | A2301 | 48 |
| MCC56 | TRIM11 | p.R398W | seralaplR | seralaplW | B4403 | 13 |
| MCC56 | TRIM34 | p.G101E | hhGeklllf | hhEeklllf | C0701 | 47 |
| MCC56 | TRIM34 | p.G101E | dhhGeklll | dhhEeklll | C0701 | 20 |
| MCC56 | TRPV6 | p.D383N | tpkdDirlv | tpkdNirlv | C0701 | 231 |
| MCC56 | TSNARE1 | p.S315F | tiaasasSv | tiaasasFv | A0201 | 244 |
| MCC56 | TSNARE1 | p.S315F | Svkqmaell | Fvkqmaell | C0701 | 463 |
| MCC56 | TTC27 | p.S740F | qeavqmlsS | qeavqmlsF | B4403 | 124 |
| MCC56 | TTC27 | p.S740F | mlsSvrlnl | mlsFvrlnl | A0201 | 120 |
| MCC56 | TXNDC2 | p.P263S | kegdiPksl | kegdiSksl | B4403 | 497 |
| MCC56 | TYK2 | p.S1157F | teaSfrptf | teaFfrptf | B4403 | 474 |
| MCC56 | TYK2 | p.S1157F | Sfrptfenl | Ffrptfenl | A2301 | 151 |
| MCC56 | UBC | p.L301F | rLrggmqif | rFrggmqif | A2301 | 419 |
| MCC56 | UBC | p.L301F | lrLrggmqi | lrFrggmqi | C0701 | 107 |
| MCC56 | UBC | p.L301F | Lrggmqifv | Frggmqifv | C0701 | 81 |
| MCC56 | UBQLNL | p.G291E | gGnpftall | gEnpftall | B4403 | 328 |
| MCC56 | UNC13C | p.E1477K | grEkfikll | grKkfikll | C0701 | 217 |
| MCC56 | UNC13C | p.E1477K | tnfgrEkfi | tnfgrKkfi | C0701 | 251 |
| MCC56 | USP2 | p.S360F | fyelasppS | fyelasppF | A2301 | 113 |
| MCC56 | VCAN | p.S1609F | sayqrtySm | sayqrtyFm | C0701 | 209 |
| MCC56 | VCAN | p.S1609F | ayqrtySmk | ayqrtyFmk | A2301 | 140 |
| MCC56 | VSTM1 | p.E40V | vEaesnvtl | vVaesnvtl | A0201 | 239 |
| MCC56 | VWA5A | p.P638L | lhsdrpPsa | lhsdrpLsa | C0701 | 78 |
| MCC56 | VWA5B2 | p.S248T | phasSaati | phasTaati | C0701 | 464 |
| MCC56 | WDR93 | p.P161S | iliaPvdem | iliaSvdem | A0201 | 91 |
| MCC56 | WDR96 | p.S734F | lliSlssam | lliFlssam | A0201 | 444 |
| MCC56 | WDR96 | p.S734F | yyqklliSl | yyqklliFl | A2301 | 79 |
| MCC56 | WDR96 | p.S734F | klliSlssa | klliFlssa | A0201 | 44 |
| MCC56 | WDR96 | p.S734F | dyyqklliS | dyyqklliF | A2301 | 48 |
| MCC56 | ZC3H12C | p.G10R | yGvlciqey | yRvlciqey | C0701 | 68 |
| MCC56 | ZER1 | p.Y54D | lvneYvelv | lvneDvelv | A0201 | 135 |
| MCC56 | ZER1 | p.Y54D | rlvneYvel | rlvneDvel | A0201 | 280 |
| MCC56 | ZFHX4 | p.L1535F | fLdpsrpyk | fFdpsrpyk | C0401 | 159 |
| MCC56 | ZNF382 | p.P71S | rifPsysyl | rifSsysyl | A0201 | 56 |
| MCC56 | ZNF415 | p.S209L | slltqeqkS | slltqeqkL | A0201 | 421 |
| MCC56 | ZNF425 | p.Q139H | illaQtatf | illaHtatf | A2301 | 112 |
| MCC56 | ZNF490 | p.S58F | siSledvav | siFledvav | A0201 | 212 |
| MCC56 | ZNF546 | p.E690K | ghtgEkpyi | ghtgKkpyi | C0701 | 56 |
| MCC56 | ZNF599 | p.A487T | kecakAfyy | kecakTfyy | B4403 | 78 |
| MCC56 | ZRANB2 | p.D10Y | frvsDgdwi | frvsYgdwi | C0701 | 93 |
| MCC56 | ZXDC | p.M752L | kMspphfha | kLspphfha | A0201 | 166 |
| MCC57 | CADPS2 | p.V515A | fvlvqVsqy | fvlvqAsqy | A3002 | 279 |
| MCC57 | CADPS2 | p.V515A | fvlvqVsqy | fvlvqAsqy | C1203 | 281 |
| MCC58 | DHX15 | p.L540Q | tlmraLell | tlmraQell | A0201 | 78 |
| MCC58 | F5 | p.F1334L | sldFsqtnl | sldLsqtnl | A0201 | 232 |
| MCC58 | IMPDH1 | p.T155M | mTprielvv | mMprielvv | A0201 | 53 |
| MCC58 | IMPDH1 | p.T155M | vmTprielv | vmMprielv | A0201 | 5 |
| MCC58 | LYPD6 | p.A138V | Atfattspi | Vtfattspi | A0201 | 230 |
| MCC58 | SLC28A2 | p.A335V | mtlseihAv | mtlseihVv | A0201 | 18 |
| MCC58 | TMPRSS11B | p.L350F | fvtdtmLca | fvtdtmFca | A0201 | 40 |
| MCC8 | ATP5F1 | p.G112R | vlgvmvyGi | vlgvmvyRi | A0201 | 137 |
| MCC8 | PHYH | p.N51I | fqytldnNv | fqytldnIv | A0201 | 59 |
| MCC8 | PHYH | p.N51I | tldnNvltl | tldnIvltl | A0201 | 33 |
| MCC8 | PHYH | p.N51I | ytldnNvlt | ytldnIvlt | A0201 | 239 |
| MCC8 | RPAP2 | p.T524S | Tlgdiytql | Slgdiytql | A0201 | 34 |
| MCC8 | RPAP2 | p.T524S | llvplqiTl | llvplqiSl | A0201 | 23 |
| MCC8 | TBCK | p.N394K | Nqiwkearv | Kqiwkearv | A0201 | 285 |
| MCC9 | B4GALT6 | p.R186Q | qRlefafyv | qQlefafyv | A0201 | 4 |
| MCC9 | DYNC2H1 | p.T664I | iTwdnpkel | iIwdnpkel | A0201 | 272 |
| MCC9 | HEATR1 | p.K1054N | avlKdeamv | avlNdeamv | A0201 | 338 |
| MCC9 | HEATR1 | p.K1054N | vlKdeamvl | vlNdeamvl | A0201 | 107 |
| MCC9 | MED13 | p.K1429N | seKlvaewf | seNlvaewf | B4001 | 73 |
| MCC9 | NFYA | p.V208A | gmVmmvpga | gmAmmvpga | A0201 | 50 |
| MCC9 | TAS2R46 | p.T16I | silivvTfv | silivvIfv | A0201 | 37 |
| MCC9 | TAS2R46 | p.T16I | ilivvTfvi | ilivvIfvi | A0201 | 34 |
| MCC9 | TMEM132D | p.T940N | lincvTfal | lincvNfal | A0201 | 100 |
| MCC9 | TMEM132D | p.T940N | flincvTfa | flincvNfa | A0201 | 5 |
| MCC9 | TMEM132D | p.T940N | vflincvTf | vflincvNf | A2402 | 183 |
| MCC9 | ZNF718 | p.R7M | lltfKdvai | lltfMdvai | A0201 | 488 |
| MCC9 | ZNF718 | p.R7M | melltfKdv | melltfMdv | B4001 | 202 |
| MCC9 | ZNF718 | p.R7M | tfKdvaief | tfMdvaief | A2402 | 244 |

Ka represents binding affinity of mutant nonamer to HLA allele.
